# Functional group divergence and the structural basis of acridine photocatalysis revealed by direct decarboxysulfonylation†

**DOI:** 10.1039/d2sc00789d

**Published:** 2022-03-21

**Authors:** Vu T. Nguyen, Graham C. Haug, Viet D. Nguyen, Ngan T. H. Vuong, Guna B. Karki, Hadi D. Arman, Oleg V. Larionov

**Affiliations:** Department of Chemistry, The University of Texas at San Antonio One UTSA Circle San Antonio TX 78249 USA oleg.larionov@utsa.edu

## Abstract

The reactivity of the sulfonyl group varies dramatically from nucleophilic sulfinates through chemically robust sulfones to electrophilic sulfonyl halides—a feature that has been used extensively in medicinal chemistry, synthesis, and materials science, especially as bioisosteric replacements and structural analogs of carboxylic acids and other carbonyls. Despite the great synthetic potential of the carboxylic to sulfonyl functional group interconversions, a method that can convert carboxylic acids directly to sulfones, sulfinates and sulfonyl halides has remained out of reach. We report herein the development of a photocatalytic system that for the first time enables direct decarboxylative conversion of carboxylic acids to sulfones and sulfinates, as well as sulfonyl chlorides and fluorides in one step and in a multicomponent fashion. A mechanistic study prompted by the development of the new method revealed the key structural features of the acridine photocatalysts that facilitate the decarboxylative transformations and provided an informative and predictive multivariate linear regression model that quantitatively relates the structural features with the photocatalytic activity.

## Abstract 2

Carboxylic acids can now serve as a single point of entry to several sulfonyl functional groups by a one-step organophotocatalytic sulfonylation, revealing structural effects that enable the photocatalysis.

## Graphical abstract

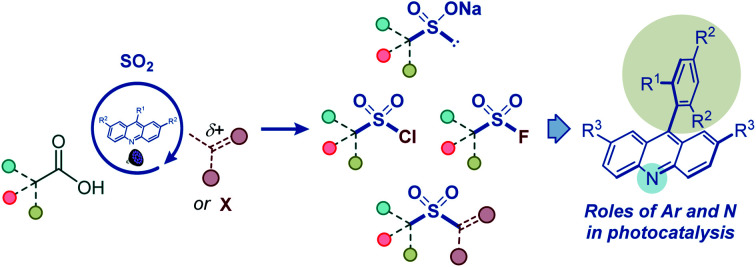

## Introduction

Functional group interconversions (FGI) are indispensable synthetic tools that can be used for a rapid entry to challenging functionalities and for structural diversification in the context of drug discovery. However, they should be applied judiciously and in the most direct manner, ideally by a direct, one-step conversion to a target functional group to maximize synthetic efficiency.^1^

**Fig. 1:**
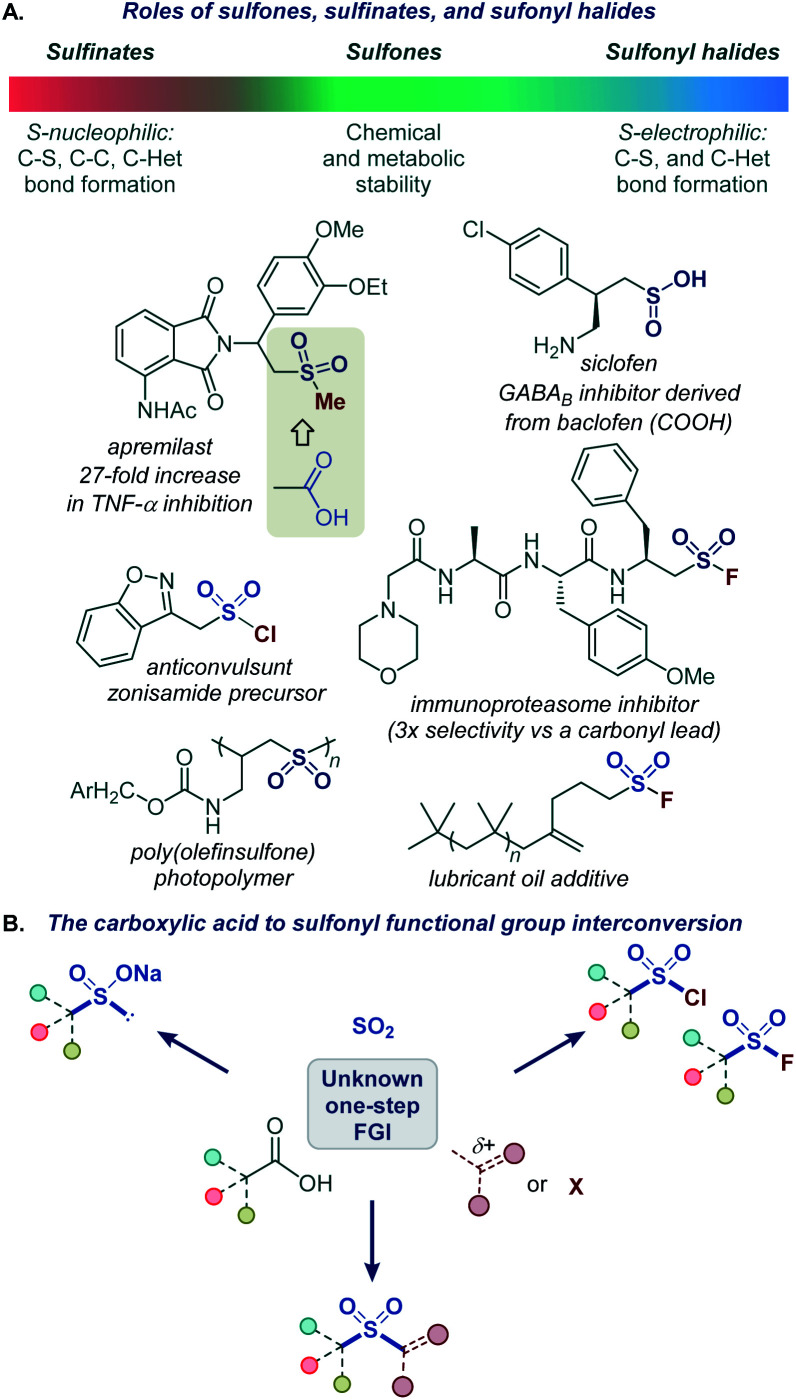
(A) Roles of sulfones, sulfinates, and sulfonyl halides. (B) The carboxylic acid to sulfonyl functional group interconversion.

The sulfonyl group occupies a preeminent position in chemistry, drug discovery, and materials science. Bearing structural and electronic resemblance to the carbonyl group, the sulfonyl group has been indispensable as a bioisosteric replacement in drug design (Fig. 1A).^2^ It also plays enabling roles in many classical and recently developed synthetic methods.^3^ The reactivity and properties of the sulfonyl group can be readily adjusted by selecting appropriate substituents on the sulfur atom, making it a chameleon functionality with a broad range of applications. For example, the robustness of sulfones makes them particularly suitable for applications in advanced materials^4^ and for the improvement of metabolic stability.^2^ By contrast, the reactive sulfonyl chlorides are used for the introduction of the sulfonyl group,^3*b*^ while sulfonyl science.^5^ On the other hand, sulfinate salts have recently emerged as versatile reagents for C–C and C–S bond-forming fluorides have found applications as covalent inhibitors and click chemistry reagents in chemical biology and materials reactions.^6–8^ Carboxylic acids are abundant naturally occurring and industrial feedstocks, as well as common synthetic intermediates and drugs. Given the central role of the sulfonyl group as a bioisosteric replacement for the carboxylic group, and the distinctive and complementary chemistries of sulfones, sulfinates, sulfonyl chlorides, and sulfonyl fluorides, a general platform that can directly convert carboxylic acids into a range of sulfonyl analogues without pre-functionalization and in one step will streamline their synthesis and enable rapid generation of bioisosteres and analogues for drug discovery and materials science applications (Fig. 1B).

**Fig. 2:**
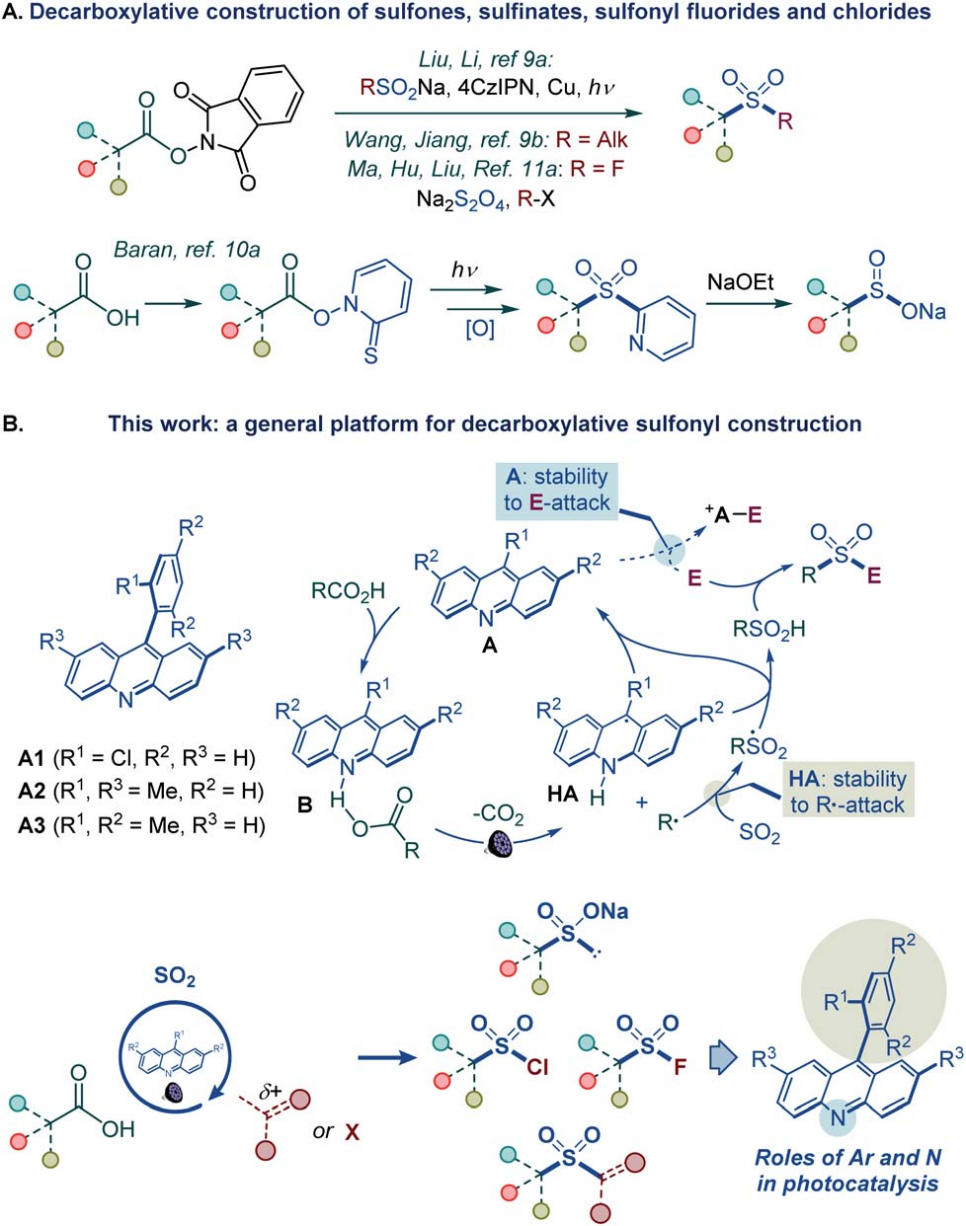
(A) Decarboxylative construction of sulfones, sulfinates, sulfonyl fluorides and chlorides. (B) Functional group-divergent decarboxysulfonylation by acridine photocatalysis.

However, such a general synthetic platform has remained elusive, and different stepwise methods have to be used for the conversion of carboxylic acids to each of the four sulfonyl classes, if available. For example, while no direct tricomponent conversion of carboxylic acids to sulfones is known, two recent reports demonstrated the feasibility of the decarboxylative construction of sulfones from *N*-hydroxyphthalimide esters, using a two-component approach with sulfinates,^9*a*^ and a tricomponent coupling with dithionite as a reductant and a sulfur dioxide donor,^9*b*^ while a four-step sequence is required to convert carboxylic acids to sulfinates *via* Barton esters (Fig. 2A).^10^ Critically, as for sulfones and sulfinates, no synthetic method is also available for decarboxylative construction of sulfonyl chlorides and fluorides directly from carboxylic acids in one step.^11^

The role of the photocatalytic system is particularly critical for the development of a single platform encompassing the direct decarboxylative construction of sulfones, sulfonates, and sulfonyl halides. Since sulfinates are more readily oxidized than carboxylates (*e.g.*, *E*_ox_ = 0.3 V *vs.* SCE for CH_3_SO_2_NBu_4_ and 1.28 V for CH_3_CO_2_NBu_4_ in MeCN), photocatalytic systems operating by a single electron oxidation of carboxylate anions may not be suitable for the development of the general platform. Additionally, the reaction should be compatible with reactive electrophilic reagents (*e.g.*, alkyl halides, *N*-haloimides) and sulfonyl halides that readily generate radicals in the presence of reducing forms of photocatalysts or upon photoinduced homolysis, leading to deleterious side reactions.

We recently described a new class of photocatalysts based on the 9-arylacridine framework that enables direct decarboxylative functionalization of carboxylic acids, obviating preactivation as redox-active esters.^12^ Mechanistic studies point to a proton-coupled electron transfer (PCET) occurring in the photoexcited acridine–carboxylic acid hydrogen bond complex B as an underlying process without the formation of acridinium carboxylate salts. However, the details of the PCET process and the roles of the C9 substituents in the photocatalysis have remained unclear.

The mildness of the reaction conditions has facilitated several challenging carbon–heteroatom and carbon–carbon bond-forming processes that directly replace the carboxylic group with a target functionality, providing a direct entry to alkenes, *N*-alkylated (hetero)aromatic amines, conjugate addition products, and sulfonamides. However, all of the developed methods required a transition metal co-catalyst that facilitated the photocatalyst turnover and the key bond-forming steps, and acridines have so far not demonstrated the ability to serve as standalone catalysts for a synthetic method.

We report herein the development of a broad-scope organophotocatalytic platform that, for the first time, enables decarboxylative construction of sulfones, sulfinates, sulfonyl chlorides, and fluorides with minimal adjustments in the reaction conditions (Fig. 2B). The reaction is catalyzed by acridines in the absence of transition metal co-catalysts. Importantly, the sulfone construction enables a direct coupling of carboxylic acids with a variety of electrophilic reagents, including alkyl halides, Michael acceptors, and heteroaryl halides without the electrophilic deactivation of the nucleophilic photocatalyst. Furthermore, our mechanistic and computational studies clarify the roles of the acridine excited states in the photoinduced decarboxylation process and establish a mechanistic and predictive framework for the effects of the C9 substituents on the photocatalytic activity.

## Results and discussion

We first focused on the direct conversion of carboxylic acids to sulfones. Radical sulfonylation has recently attracted attention as an efficient approach to aliphatic sulfones, as shown by Glorius, Jiang, Li, Wu, and others.^9^ After initial optimization studies, we found that cyclohexanecarboxylic acid can be efficiently converted to the corresponding allyl sulfone 1a in a tricomponent reaction with allyl bromide and DABSO (DABCO–bis(sulfur dioxide) adduct)^13^ as a stable and solid sulfur dioxide source in the presence of acridine catalyst A1 with 400 or 420 nm LED irradiation (Table 1, entries 1 and 2). The reaction did not proceed without light and the acridine catalyst (entries 3 and 4). Other acridine catalysts A2 and A3 provided comparable catalytic performance (entries 5 and 6). Importantly, other classes of photocatalysts (*e.g.*, Ir- and Ru-based photocatalysts, 4CzIPN, and the *N*-methyl 9-mesitylacridinium catalyst) failed to deliver the sulfone product (Table S1†). Trifluorotoluene was also a suitable solvent (entry 7). Sodium metabisulfite^14^ – a stable and readily available bulk commodity chemical – can also be used as a sulfur dioxide source (entry 8). An evaluation of the sensitivity of the reaction to various reaction parameters^15^ indicated that the reaction is generally insensitive to changes in the concentration, temperature, and moisture, and is moderately sensitive to scale-up, and when carried out under air or with reduced light intensity, with the yield in all cases remaining in the synthetically useful range (>70%, see Fig. S1†).

**Table 1:** Reaction conditions for the photocatalytic direct decarboxylative alkylsulfonylation^a^

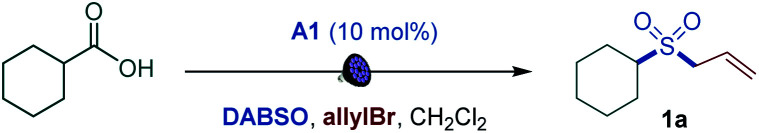		
Entry	Change from optimal conditions	Yield, %
1	None	96 (92^b^)
2	420 nm instead of 400 nm LED	95
3	No light	0
4	No A1	0
5	A2 instead of A1	94
6	A3 instead of A1	95
7	PhCF_3_ instead of CH_2_Cl_2_	96
8	Na_2_S_2_O_5_ instead of DABSO in PhCF_3_	83 (80^b^)

a Reaction conditions: carboxylic acid (0.3 mmol), DABSO (0.33 mmol), A1 (10 mol%), allyl bromide (0.75 mmol), CH_2_Cl_2_ (6 mL), LED light (400 nm), 12 h. Yield was determined by ^1^H NMR spectroscopy with 1,4-dimethoxybenzene as an internal standard.

b Isolated yield. DABSO = O_2_S–N(CH_2_CH_2_)_3_N–SO_2_.

**Scheme 1:**
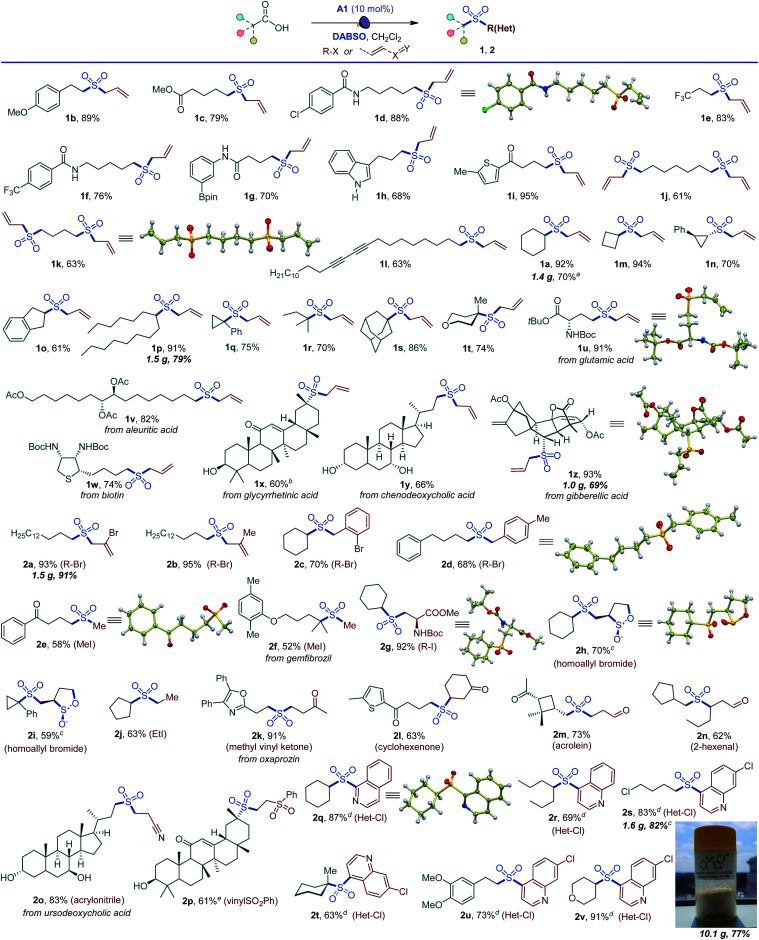
Scope of the visible light-induced decarboxylative alkylsulfonylation. Reaction conditions: carboxylic acid (0.15–0.3 mmol), DABSO (0.33–0.45 mmol), A1 (10 mol%), alkyl halide (0.75 mmol), CH_2_Cl_2_ (3–6 mL), LED light (400 nm), 12 h. ^*a*^With Na_2_S_2_O_5_. ^*b*^12 : 1 dr. ^*c*^*cis*/*trans* ratio: 9 : 1. ^*d*^Halogenated nitrogen heterocycle (0.3 mmol), carboxylic acid (0.75 mmol), DABSO (0.33 mmol), A1 (10 mol%), CH_2_Cl_2_ (6 mL), LED light (400 nm), 12 h. ^*e*^19 : 1 dr.

The simplicity of the direct decarboxylative alkylsulfonylation provided an opportunity to carry out an initial evaluation of the substrate scope that revealed broad compatibility and efficiency of the sulfonylation with a wide array of functionalized carboxylic acids (*e.g.*, sulfones 1a–1z, Scheme 1). Primary carboxylic acids bearing aryl (1b), ester (1c), amide (1d), the medicinally important trifluoromethyl (1e, 1f), and the synthetically versatile boryl (1g) groups were directly converted to corresponding sulfones. Unprotected electron rich indole (1h) and a thiophene-containing ketone (1i) were also well tolerated. The reaction can be readily performed with diacids, resulting in an efficient double alkylsulfonylation (1j, 1k). In addition, triple C–C bonds were compatible with the direct decarboxylative alkylsulfonylation (1l). A range of secondary carboxylic acids were converted to corresponding sulfones (1a, 1m–1p), including strained small rings (1m, 1n). Importantly, high diastereoselectivity can be achieved, as demonstrated for *trans*-sulfone 1n. Furthermore, both acyclic and cyclic tertiary acids readily produced sulfones 1q–1t in good yields, highlighting the broad scope of the reaction. To test the performance of the alkylsulfonylation reaction in more complex structural settings, the reaction was carried out with several natural products and their derivatives (1u–1z). A sulfone isostere of glutamic acid was produced (1u), revealing a straightforward route to biologically important cysteine sulfinic acid derivatives.^16^ Similarly, aleuritic acid and biotin sulfone isosteres 1v and 1w were readily synthesized. Derivatives of medicinally relevant glycyrrhetinic (1x), chenodeoxycholic (1y), and gibberellic (1z) acids were also efficiently converted to the corresponding sulfones revealing high diastereoselectivity (1x and 1z) and tolerance to several easily oxidizable and reactive functional groups (1w–1z). Other electrophiles can also be used (Scheme 1). Substituted allyl (2a, 2b) and benzyl (2c, 2d) halides are suitable electrophiles. Other alkyl halides also participate in the tricomponent sulfone synthesis (2e–2j), *e.g.*, allowing for a one-step access to sulfone isostere of aspartate 2g. Notably, reactions with homoallyl bromide resulted in a four-component radical cascade coupling process (Scheme 1 and Fig. S1†), producing previously inaccessible *cis*-sultines (2h, 2i).^17^ Furthermore, various reactive Michael acceptors can also be used as electrophiles, including α,β-unsaturated ketones, aldehydes and acrylonitrile. A range of conjugate addition-derived sulfones 2k–2p were readily accessed. Notably, the cyclobutane ring in *cis*-pinonic acid that typically readily undergoes ring cleavage upon decarboxylation^12*a*^ remained intact (2m), highlighting the functional group tolerance and efficiency of the transient alkyl radical capture. The reaction is not limited to alkyl electrophiles, as for example, nitrogenous heteroaryl sulfones 2q–2v can be prepared in a tricomponent manner with halogenated nitrogen heterocycles.

**Scheme 2:**
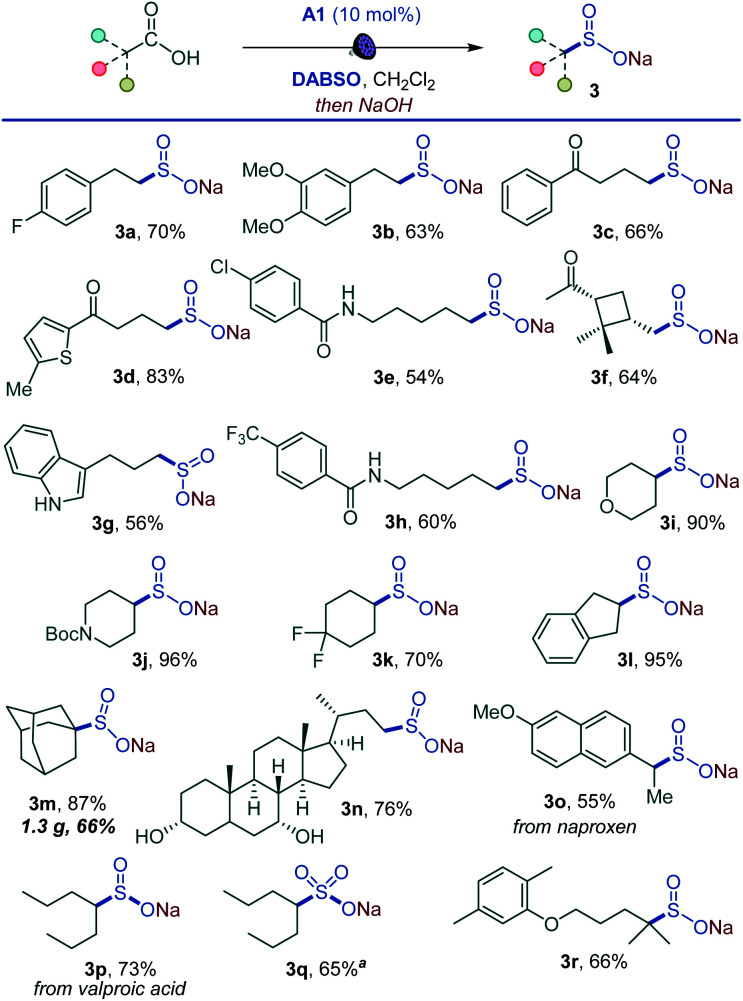
Direct decarboxylative synthesis of alkyl sulfinates. Reaction conditions: carboxylic acid (0.3 mmol), DABSO (0.33 mmol), A1 (10 mol%), CH_2_Cl_2_ (6 mL), LED light (400 nm), 12 h, then 1 M NaOH in MeOH (1 mL, 1.0 mmol), r.t., 10 min. ^*a*^Na_2_S_2_O_8_ (0.45 mmol) was added after treatment with NaOH.

Importantly, the decarboxysulfonylation reaction can be interrupted to access sulfinate salts (Scheme 2). In line with the reactivity observed in the alkylsulfonylation, a wide range of sulfinate salts is accessible after a simple basic work-up from primary, secondary and tertiary acids (3a–3r). Easily oxidizable unprotected indole (3g) and hydroxy groups (3n) were tolerated, and a variety of functionalized natural product- and small molecule therapeutics-derived sulfinates were readily accessed (3n–3r). A pre-workup treatment of the sulfinate reaction mixture of the anti-convulsant drug valproic acid (3p) with sodium persulfate^18^ also produced the corresponding sulfonate salt 3q, indicating that the method can be expanded to access sulfonates. Taken together, the procedure establishes a direct one-step route to sulfinate salts from carboxylic acids.

**Scheme 3:**
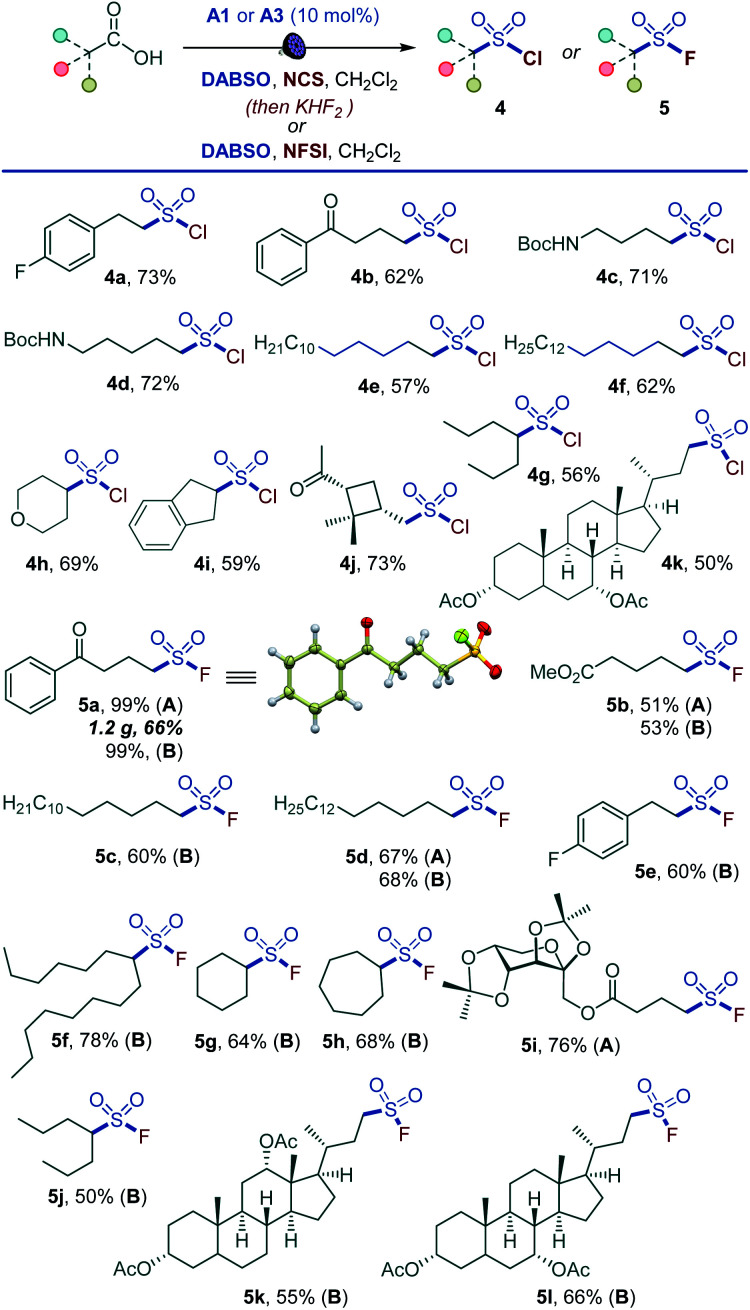
Scope of the direct decarboxylative chloro- and fluorosulfonylation. Reaction conditions: carboxylic acid (0.3 mmol), DABSO (0.3–0.36 mmol), A1 (10 mol%), *N*-chlorosuccinimide (NCS) (0.6–0.75 mmol), CH_2_Cl_2_ (6 mL), LED light (400 nm), 8–12 h, then purification for 4, or (method A) 2 M KHF_2_ (0.35 mL, 0.69 mmol), MeCN (2 mL), 50 °C, 3 h or 80 °C, 5 min. Method B: carboxylic acid (0.3 mmol), DABSO (0.45 mmol), A2 (10 mol%), *N*-fluorobenzenesulfonimide (NFSI) (0.45 mmol), CH_2_Cl_2_ (6 mL), LED light (400 nm), 12 h.

Given the synthetic importance of sulfonyl chlorides and fluorides,^3,5,11^ we questioned if the decarboxylative sulfonyl construction approach can be adapted to access sulfonyl halides. This would constitute the first example of a direct one-step conversion of carboxylic acids to sulfonyl halides. Indeed, a range of sulfonyl chlorides were readily available by carrying out the reaction in the presence of *N*-chlorosuccinimide (NCS) (4a–4k, Scheme 3). The tested substates included compounds that are susceptible to facile radical halogenation with NCS (*e.g.*, 4h, 4i) and the readily cleavable *cis*-pinonic acid (4j). Additionally, the reaction can be adapted to the synthesis of sulfonyl fluorides by a work-up with potassium bifluoride (5a, 5b, 5d, 5i). The nucleophilic fluoride work-up can be completed in 5 minutes. The short conversion time can be important for time-sensitive introduction of the short-lived ^18^F label for preparation of radiolabeled sulfonyl fluorides in chemical biology and radiotherapeutics.^19^ We also probed if a direct tricomponent synthesis of sulfonyl fluorides can be accomplished by carrying out the decarboxysulfonylation in the presence of a fluorinating reagent. Gratifyingly, *N*-fluorobenzenesulfonimide (NFSI) was identified as an optimal fluorinating reagent, and a range of functionalized sulfonyl fluorides became accessible in one step (5a–5h, 5j–5l), establishing a one-step reaction that converts carboxylic acids directly to sulfonyl fluorides. Notably, sulfonyl fluoride bioisosteres of palmitic (5c) and stearic (5d) acids,^20^ as well as fructose- (5i) and bile acids-derived (5k and 5l) sulfonyl fluorides can be accessed directly from the corresponding carboxylic acids. The applicability of nucleophilic and electrophilic fluorine sources provides synthetic flexibility and highlights the inherent adaptability of the acridine-photocatalyzed direct decarboxylative sulfonyl construction approach. The preparative utility of the functional group-divergent sulfonylation was tested in a series of gram scale syntheses, and the corresponding products encompassing sulfones (1a, 1p, 1z, 2a, 2s, 2v), sulfonates (3m), and sulfonyl halides (5a) were readily obtained in good yields, including on a decagram scale for heterocyclic sulfone 2v in 77% yield without additional scale-up optimization and with sodium metabisulfite for sulfone 1a. The sulfinate salt formation proceeded with quantum yield of 0.38, pointing to the efficiency of the photocatalytic process. The suitability of the method for the rapid access to diverse sulfone, sulfinates, and sulfonyl halide analogues of carboxylic acids in the context of drug discovery is readily demonstrated by the facile construction of libraries of the corresponding sulfonyl-derivatives of the anticonvulsant drug valproic acid (2r, 3q, 3p, 4g, 5j), lipid regulator gemfibrozil (2f, 3r), bile acids (1y, 2o, 3n, 5k, 5l), nonsteroidal anti-inflammatory drugs (2k, 3o), plant growth regulator gibberellic acid (1z, 6s) and others.

**Fig. 3:**
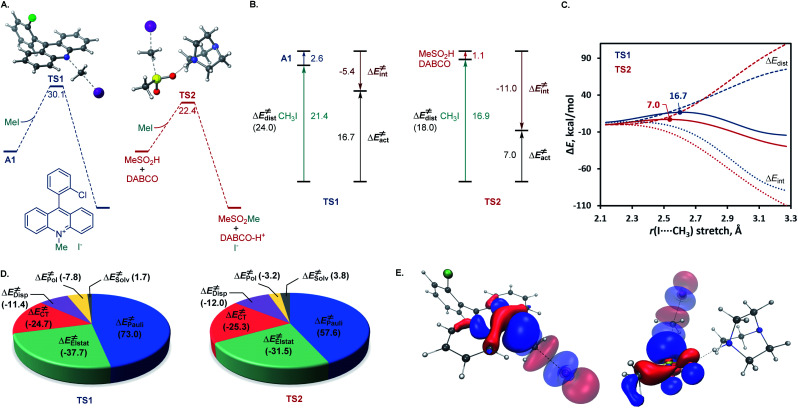
(A) Computed Gibbs free energy profile for the alkylation of acridine A1 and sulfinic acid in the presence of DABCO. (B) Activation strain model distortion–interaction analysis of TS1 and TS2. (C) Activation strain diagram of the alkylation of acridine A1 (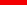) and sulfinic acid in the presence of DABCO (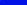). (D) The most significant complementary occupied-virtual pairs (COVP) for TS1 and TS2. Δ*G* and Δ*E* in kcal mol^−1^.

**Fig. 4:**
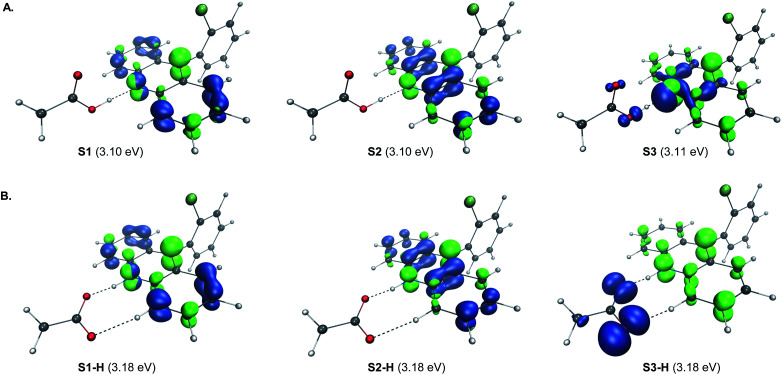
Electron–hole analysis of the three lowest singlet excited states of the hydrogen bond complex of acridine A1 and acetic acid before (A) and after (B) the O → N proton transfer.

A remarkable feature of the acridine-photocatalyzed sulfone construction is the resistance of the nucleophilic *N*-center of the photocatalyst to reactions with powerful electrophiles that are present in large excess *vis-à-vis* the acridine, permitting selective alkylation of the sulfinic acid product and the photocatalyst turnover that would not be possible if the acridine underwent *N*-alkylation. To probe the nature of the resistance of the photocatalyst to *N*-alkylation, computational studies were carried out for acridine and the sulfinic acid product. Although modestly exergonic, the alkylation of catalyst A1 was found to proceed over a prohibitively high barrier of 30.1 kcal mol^−1^ (TS1) (Fig. 3A). By contrast, the DABCO-assisted *S*-alkylation of the sulfinic acid was significantly exergonic and took place with an accessible barrier of 22.3 kcal mol^−1^ in agreement with the experimental observations (TS2). Further analysis by means of the activation strain model (AIM)^21^ revealed that the alkylation of acridine A1 proceeds with higher distortion energy and lower interaction energy than the alkylation of the sulfuric acid, accounting for the substantially higher activation barrier (Fig. 3B and C). In both cases, the distortion in the alkyl halide fragment was the major component of the distortion energy, with a greater CH_3_I distortion for catalyst A1 to accommodate the larger nucleophile, as evident from the longer C⋯I distance in TS1 (2.62 Å) *vis-à-vis*TS2 (2.52 Å). To gain insight into the factors responsible for the higher interaction energy in the case of TS2, the second-generation energy decomposition analysis based on absolutely localized molecular orbitals (ALMO-EDA(solv)) was carried out (Fig. 3D).^22^ It revealed that the acridine system has a substantially higher Pauli repulsion (steric) energy that cannot be compensated by the moderately higher electrostatic and polarization components, and a lower solute–solvent interaction energy. The sulfinic acid system also benefits from a somewhat stronger stabilizing dispersion and an increased charge transfer interaction, due to a smaller HOMO–LUMO gap (5.58 eV for TS2*vs.* 5.87 eV for TS1, Fig. S3†). The complementary occupied virtual pair (COVP)^23^ analysis indicates that the charge transfer is dominated by the donation from the *N*- or *S*-centered *n* orbital of the nucleophile to the σ* orbital of the alkyl halide in both cases (Fig. 3E). Taken together, these studies point to the stronger repulsive interactions around the more congested *N*-center in acridine and a weaker stabilizing interaction with the electrophile in TS1 in combination with a greater distortion required to accommodate the larger *N*-nucleophile as the underlying causes of the kinetically disfavored deactivation of the photocatalyst in the presence of highly reactive electrophiles. Substituents can have profound effects on photocatalytic activity.^24^ In the case of the acridine photocatalysts, the 9-aryl group was found to significantly improve the efficiency of all of the direct decarboxylative functionalizations developed to date. In particular, acridines bearing *ortho*-substituted 9-aryl groups (*e.g.*, A1–A3) displayed the best photocatalytic performance. On the contrary, the unsubstituted acridine (A4) is typically inefficient and large loadings (*e.g.*, 20–30 mol%) are required to effect a direct decarboxylative functionalization, resulting in low yields and slower rates. To examine the influence of the 9-aryl substitution on the lowest singlet excited states that may be involved in the photoinduced proton-coupled electron transfer (PCET), computational studies were carried out on acridine A1 that was selected because it demonstrated optimal or near-optimal catalytic performance across a wide range of the direct decarboxylative transformations. Our prior studies indicated that the photoinduced PCET takes place in the singlet excited state of the hydrogen bond acridine–carboxylic acid complex.^12*a*^ The three lowest singlet excited states of complex A1–AcOH prior to the proton transfer from the acid to the acridine catalyst were therefore first examined (Fig. 4A). Notably, the S1–S3 excited states were nearly isoenergetic. An electron–hole analysis further indicated that the S1 and S2 excited states had π → π* character, while S3 resulted from the *n* → π* transition. Significantly, the 9-aryl group was not involved in the transitions, indicating that it does not have a direct influence on the nature of the excited states. Additionally, no contribution of the carboxylate to the hole in S1 and S2 states and a minor (8.3%) contribution in the S3 state suggests that the electron transfer from the acid to the acridine does not occur before the O → N proton transfer.

The proton transfer results in a set of excited states S1-H to S3-H that are only slightly higher in energy (ΔΔ*G* < 2 kcal mol^−1^), indicating that they are readily thermally accessible on the singlet excited state surface (Fig. 4B). The π → π* character of S1-H and S2-H, and *n* → π* for S3-H remained unchanged. Significantly, while no involvement of the carboxylate fragment in the hole was observed for the S1-H and S2-H states, the hole in S3-H was predominantly localized on the carboxylate (92.3%), indicating that readily accessible S3-H singlet state can serve as a channel for the charge transfer that leads to a low-barrier decarboxylation^12*a*^ en route to the alkyl radical.

**Fig. 5:**
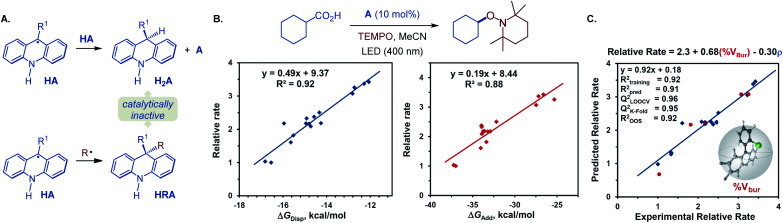
Mechanistic study of the acridine photocatalysis. (A) Disproportionation and cross-termination reactions of acridinyl radical HA. (B) Dependence of the relative rate (rate of the reaction catalyzed by a C9-substituted acridine *versus* unsubstituted acridine) of the visible light-induced, C9-alkyl and aryl-substituted acridine-catalyzed decarboxylative reaction with TEMPO on the Gibbs free energy of the acridinyl radical disproportionation (Δ*G*_disp_) and cyclohexyl radical addition to the acridinyl radical (Δ*G*_add_). (C) Multivariate linear regression model relating steric (percent buried volume, % *V*_bur_ of the C9 substituent) and electronic (sum of NBO spin densities on C9 and C9 substituent, *ρ*) parameters of acridinyl radical intermediate HA with the observed photocatalytic reactivity of 9-substituted acridines.

Our prior investigation revealed that dihydroacridine (H_2_A) was formed when acridine (A4) was used as a photocatalyst, resulting from the disproportionation of two acridinyl radicals (Fig. 5A),^25^ and that oxidation of dihydroacridine (H_2_A) was needed to turnover the catalyst, and in some cases a transition metal co-catalyst was required to effect the reoxidation.^12*a*^

Additionally, we observed formation of significant amounts of 9-alkyl by-product HRA, stemming from the cross-termination of the alkyl radical with acridinyl radical HA following the PCET and decarboxylation. Given the experimentally observed accumulation of substantial quantities of dihydroacridines H_2_A and HRA with unsubstituted acridine (A4) as a photocatalyst, we hypothesized that the photocatalytic activity may inversely depend on the propensity to undergo the catalyst deactivation reactions resulting in these by-products that may forestall or slow down the photocatalyst turnover. More sterically encumbered C9 substituents may disfavor the acridinyl radical disproportionation and the cross-termination with an alkyl radical, resulting in an improved catalytic performance. To probe the roles of the C9-substitution on the photocatalytic activity of acridines, the photocatalytic activities of a structurally and electronically diverse 9-aryl and alkyl-substituted acridines in reference to unsubstituted acridine (A4) were studied in the photoinduced reaction of cyclohexanecarboxylic acid with TEMPO (Fig. 5B). Computational studies were performed to derive Boltzmann-averaged Gibbs free energies for the cross-termination of the cyclohexyl radical with the corresponding C9-substituted acridinyl radical HA (Δ*G*_add_) and for the disproportionation of two HA radicals (Δ*G*_disp_). Interestingly, the relative reaction rates showed a strong correlation with the Gibbs free energies of the cross-termination and disproportionation processes (Fig. 5B). Multivariate linear regression analysis^26^ was then applied using a stepwise linear regression algorithm to gain further insights into the roles of various molecular parameters relevant to the photocatalysis, and to obtain a predictive statistical model that can be used for the development of new acridine photocatalysts. Evaluation of a range of computationally derived Boltzmann-averaged molecular steric (percent buried volume of the C9 substituent, % *V*_bur_), electronic (charge and spin density on C9 and on the C9 substituent), and photophysical (*e.g.*, *λ*_max_, oscillator strength *f*, molar absorptivity *ε*_max_ and *ε*_400 nm_) parameters revealed an accurate (*R*^2^ = 0.92) two-parameter statistical model composed of the steric (% *V*_bur_) term and a sum of NBO spin densities on C9 and the C9 substituent (*ρ*). Cross-validation and external validation (16 : 5 training/test set split) analyses indicate a robust model (*Q*_LOOCV_^2^ = 0.96, *R*_pred_^2^ = 0.91, out-of-sample *R*^2^ = 0.92). Suggesting that it can be used as a predictive tool for the development of new acridine photocatalysts. Furthermore, comparison of the magnitudes of the coefficient terms points to the predominant role of the steric repulsion of the C9 substituent in the photocatalytic activity of 9-substituted acridines. Additionally, the inverse relationship of the photocatalytic activity and the spin density term suggests that a more delocalized acridinyl radical may contribute to a more efficient photocatalytic system. Taken together, the results of the TD-DFT and DFT computational and kinetic studies, as well as statistical analysis point to the central role of the C9 substituents that safeguard against the deactivation of the photocatalyst by deleterious acridinyl radical attrition processes.

## Conclusions

In conclusion, we have developed a visible light-induced, organophotocatalytic reaction that enables access to sulfones, sulfinates, sulfonyl chlorides, and sulfonyl fluorides by a direct tricomponent decarboxylative coupling of carboxylic acids. Notably, the acridine photocatalyst does not undergo deactivation in the presence of a variety of electrophilic coupling partners. Mechanistic and computational studies provided insights into the reactivity of the acridine photocatalytic system that highlighted the steric effects, underlying the resistance of the catalyst to deactivation by electrophiles at N1 position and by radical intermediates at C9 position. A multivariate linear regression model has been developed to quantitatively describe the steric effects of the C9 substituents and the spin density in the key acridinyl radical intermediate. Additionally, computational studies also pointed to the important role of the excited state proton transfer for the acridine-photocatalyzed decarboxylation process.

## Data availability

All experimental procedures, characterization data, and NMR spectra for all new compounds can be found in the ESI.†

## Author contributions

VTN, VDN, NTHV, and GBK carried out the experiments, and GCH performed the computational studies. HDA performed the X-ray crystallography studies. OVL conceived the project, wrote the manuscript, and co-wrote the ESI. VTN, VDN, and GCH co-wrote the ESI and contributed to writing the manuscript.

## Conflicts of interest

There are no conflicts to declare.

## Supplementary Material

SC-013-D2SC00789D-s001

SC-013-D2SC00789D-s002
