# Supplementary material for: Functional group divergence and the structural basis of acridine photocatalysis revealed by direct decarboxysulfonylation
Source: Chem Sci. 2022 Mar 21;13(14):4170–9. doi: 10.1039/d2sc00789d (PMC8985579; doi:10.1039/d2sc00789d)
Supplement: SC-013-D2SC00789D-s001 [file SC-013-D2SC00789D-s001.pdf]

Electronic Supplementary Material (ESI) for Chemical Science.
This journal is © The Royal Society of Chemistry 2022

Supporting Information

Functional Group Divergence and the Structural Basis of Acridine Photocatalysis Revealed by Direct Decarboxysulfonylation

Vu T. Nguyen,[+] Graham C. Haug,[+] Viet D. Nguyen,[+] Ngan H. T. Vuong, Guna B. Karki, Hadi D. Arman, and Oleg V. Larionov*

Department of Chemistry, The University of Texas at San Antonio, San Antonio, Texas 78249, United States

oleg.larionov@utsa.edu

Contents

Materials and experimental details S1
General procedures S2
Additional experiemental and computational studies S5
Acridine synthesis S11
Sulfones S12
Sulfinate salts S50
Sulfonyl chlorides and sulfonyl fluorides S62
Computational data S80
X-ray crystallographic data S216
NMR spectroscopic data S226
References S410

Materials and experimental details

Materials: Acridines **A1**, **A2**,[1] and **A3**,[2] as well as DABSO,[3] compounds 6-(4-(trifluoromethyl)benzamido)hexanoic acid (**S8**),[4] 6-(4-chlorobenzamido)hexanoic acid (**S9**),[5] 5-oxo-5-((3-(4,4,5,5-tetramethyl-1,3,2-dioxaborolan-2-yl)phenyl)amino)pentanoic acid (**S10**),[6] 5-(5-methylthiophen-2-yl)-5-oxopentanoic acid (**S11)**,[7] 9,10,16-triacetoxyhexadecanoic acid (**S12**),[8] 5-((2*S*,3*S*,4*R*)-3,4-bis((*tert*-butoxycarbonyl)amino)-tetrahydrothiophen-2-yl)pentanoic acid (**S13)**,[9,10] 3,13-di-*O*-acetylgibberellic acid (**S14**),[11] 3*α*,7*α*-diacetoxy-5β-cholanic acid (**S15**),[12] 5-oxo-5-(((3a*S*,5a*R*,8a*R*,8b*S*)-2,2,7,7-tetramethyltetrahydro-3a*H*-bis([1,3]dioxolo)[4,5-b:4',5'-d]pyran-3a-yl)methoxy)penta-noic acid (**S16**),[13] deoxycholic acid 3,12-diacetate (**S17**),[12] and cholic acid 3,7,12-

[+] V.T.N., G.C.H., and V.D.N. contributed equally.

triacetate(**S18**)[12] were prepared as previously described. All other chemicals were used as commercially available.

Experimental equipment: Reactions were set up by purging vigorously stirred reaction mixtures with argon for 3 min prior to irradiation, or in a glovebox. Borosilicate glass test-tubes (9 and 10 mL capacity) fitted with GL14 and GL18 screw-caps were used, and the sealed reaction test-tubes were placed in a test-tube rack on a magnetic stirplate that was flanked by two 400 nm 36W LED lights. The temperature in the test-tube rack was 35 °C. Eight parallel reactions arranged in two rows of four tubes were typically carried out in one test-tube rack.

Glovebox work was carried out in a nitrogen-filled LC Technology Solutions LCPW-220 glovebox.

Purification: Column chromatography was performed using CombiFlash Rf-200 (Teledyne-Isco) automated flash chromatography system, as well as manually. Thin layer chromatography was carried out on silica gel-coated glass plates (Merck Kieselgel 60 F254). Plates were visualized under ultraviolet light (254 nm) and using a potassium permanganate stain.

Characterization: ^{1}H, ^{13}C, ^{11}B, and ^{19}F NMR spectra were recorded at 500 MHz (^{1}H), 125 MHz (^{13}C), 202 MHz (^{31}P), 470 MHz (^{19}F), and 160 MHz (^{11}B) on Bruker AVANCE III 500 instruments in $CDCl_3$ or other specified deuterated solvents with and without tetramethylsilane (TMS) as an internal standard at 25 °C, unless specified otherwise. Chemical shifts (δ) are reported in parts per million (ppm) from tetramethylsilane (^{1}H and ^{13}C), $BF_3{\cdot}OEt_2$ (^{11}B), and $CFCl_3$ (^{19}F). Coupling constants (*J*) are in Hz. Proton multiplicity is assigned using the following abbreviations: singlet (s), doublet (d), triplet (t), quartet (q), quintet (quint.), septet (sept.), multiplet (m), broad (br).

Infrared measurements were carried out neat on a Bruker Vector 22 FT-IR spectrometer fitted with a Specac diamond attenuated total reflectance (ATR) module. EPR Spectra were collected on a Bruker EMX X-band EPR spectrometer.

General Procedures

General procedure for the tricomponent, visible light-induced, direct decarboxylative allyl sulfone synthesis (GP1)

To a 10 mL test-tube, carboxylic acid (0.3 mmol), DABSO (79 mg, 0.33 mmol, 1.1 equiv.), acridine catalyst **A1** (9 mg, 0.03 mmol, 10 mol%), allyl bromide (91 mg, 0.75 mmol, 2.5 equiv.), and degassed dichloromethane (6 mL) were added. The test-tube was capped, and the reaction mixture was irradiated with LED light (λ = 400 nm) while stirring at room temperature for 12 h.

The reaction mixture was then quenched with a saturated solution of potassium hydrogen sulfate (2 mL) and extracted with ethyl acetate (3 × 10 mL). The organic layer was combined, dried over anhydrous sodium sulfate, concentrated under reduced pressure, and the remaining material was purified by flash chromatography on silica gel to give the sulfone product.

General procedure for the tricomponent, visible light-induced, direct decarboxylative sulfone synthesis with alkyl halides or Michael acceptors (GP2)

To a 10 mL test-tube, carboxylic acid (0.3 mmol), DABSO (86-108 mg, 0.36–0.45 mmol, 1.2–1.5 equiv.), acridine catalyst **A1** (9 mg, 0.03 mmol, 10 mol%), alkyl halides or Michael acceptors (0.75−1.5 mmol, 2.5−5 equiv.) and degassed dichloromethane (3 mL) were added. The test-tube was capped and the reaction mixture was irradiated with LED light (λ = 400 nm) while stirring at room temperature for 12 h. The reaction mixture was then quenched with a saturated solution of potassium hydrogen sulfate (2 mL) and extracted with ethyl acetate (3 × 10 mL). The organic layer was combined, dried over anhydrous sodium sulfate, concentrated under reduced pressure, and the remaining material was purified by flash chromatography on silica gel to give the sulfone product.

General procedure for the direct decarboxylative sulfinate salt synthesis (GP3)

To a 10 mL test-tube, carboxylic acid (0.3 mmol), DABSO (79 mg, 0.33 mmol, 1.1 equiv.), acridine catalyst **A1** (9 mg, 0.03 mmol, 10 mol%), and degassed dichloromethane (6 mL) were added. The test-tube was capped and the reaction mixture was irradiated with LED light (λ = 400 nm) while stirring at room temperature for 12 h. After completion, a 1M solution of sodium hydroxide (1 mL, 1 mmol, 3.3 equiv.) in MeOH was added. The mixture was stirred for 10 min and purified by flash chromatography on silica gel to give the desired sulfinate salt.

General procedure for the the visible light-induced, direct decarboxylative sulfonyl chloride synthesis (GP4)

To a 10 mL test-tube, carboxylic acid (0.3 mmol), DABSO (0.3–0.36 mmol, 1−1.2 equiv.), acridine catalyst **A1** (9 mg, 0.03 mmol, 10 mol%), *N*-chlorosuccinimide (0.6−0.75 mmol, 2−2.5 equiv.), and degassed dichloromethane (6 mL) were added. The test-tube was capped and the reaction mixture was irradiated with LED light (λ = 400 nm) while stirring at room temperature for 12 h. The reaction mixture was concentrated under reduced pressure, and the remaining material was purified by flash chromatography on silica gel to give the sulfonyl chloride product.

General procedure for the visible light-induced, direct decarboxylative sulfonyl fluoride synthesis with potassium bifluoride (Method A, GP5)

The reaction was carried out as described in the GP5. After completion, the reaction mixture was concentrated under reduced pressure and acetonitrile (2 mL), as well as a 2M aqueous solution of potassium bifluoride (0.35 mL, 0.69 mmol, 2.3 equiv.), were added. The reaction was stirred at 50 °C for 3 h before quenching with a saturated solution of potassium hydrogen sulfate (4 mL) and extracting with ethyl acetate (3 × 10 mL). The organic layer was combined, dried over anhydrous sodium sulfate, concentrated under reduced pressure, and the remaining material was purified by flash chromatography on silica gel to give the sulfonyl fluoride product.

General procedure for the visible light-induced, direct decarboxylative sulfonyl fluoride synthesis with NFSI (Method B, GP6)

To a 10 mL test-tube, carboxylic acid (0.3 mmol), DABSO (108 mg, 0.45 mmol, 1.5 equiv.), acridine catalyst **A3** (9 mg, 0.03 mmol, 10 mol%), *N*-fluorobenzenesulfonimide (142 mg, 0.45 mmol, 1.5 equiv.), and degassed dichloromethane (6 mL) were added. The test-tube was capped and the reaction mixture was irradiated with LED light (λ = 400 nm) while stirring at room temperature for 12 h. The reaction mixture was concentrated under reduced pressure, and the remaining material was purified by flash chromatography on silica gel to give the sulfonyl fluoride product.

Go back to table of contents

Additional experiemental and computational studies

Table S1. Catalyst performance in the photocatalytic direct decarboxylative sulfonylation. [a]

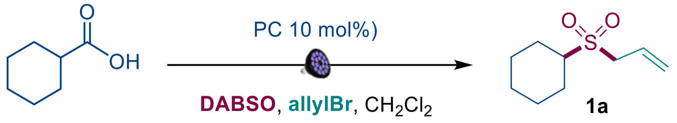

Entry	Photocatalyst	Yield, %
1	Eosin Y at 450 nm	0
2	Eosin Y at 420 nm	0
3	Eosin Y at 400 nm	0
4	Eosin Y disodium salt at 450 nm	0
5	4CzIPN at 450 nm	0
6	4CzIPN at 420 nm	0
7	4CzIPN at 400 nm	0
8	$[Acr\text{-}Mes]^+(ClO_4)^-$ at 400 nm	0
9	$[Acr\text{-}Mes]^+(ClO_4)^-$ at 450 nm	0[b]
10	$Ir(ppy)_3$ at 450 nm	0[b]
11	$Ir(ppy)_2(pq)$ at 450 nm	0[b]
12	$(Ir[dF(CF_3)ppy]_2(dtbpy))PF_6$ at 450 nm	0[b]
13	$Ru(bpm)_2Cl_2$ at 450 nm	0[b]
14	Ru(*p*-CF_3-bpy$)_3(BF_4)_2$ at 450 nm	0[b]
15	TiO_2, anatase	0[c]

[a] Reaction conditions: carboxylic acid (0.3 mmol), DABSO (0.33 mmol), **PC** (2-10 mol%), allyl bromide (0.75 mmol), CH_2Cl_2 (6 mL), LED light (400 nm), 12 h. Yield was determined by ^1H NMR spectroscopy with 1,4-dimethoxybenzene as an internal standard. [b] 2 mol% photocatalyst was used. [c] nanopowder, <25 nm particle size, 30 mg. 4CzIPN: 1,2,3,5-Tetrakis(carbazol-9-yl)-4,6-dicyanobenzene, $[Acr\text{-}Mes]^+(ClO_4)^-$: 10-Methyl-9-(2,4,6-trimethylphenyl)acridinium perchlorate, $Ir(ppy)_3$: Tris(2-phenylpyridine)iridium(III), $Ir(ppy)_2(pq)$: bis(2-phenylpyridine)(2-phenyl-quinoline)iridium(III), $(Ir[dF(CF_3)ppy]_2(dtbpy))PF_6$: [4,4′-Bis(1,1-dimethylethyl)-2,2′-bipyridine-*N1,N1′*]bis[3,5-difluoro-2-[5-(trifluoromethyl)-2-pyridinyl-*N*]phenyl-C]Iridium(III) hexafluoro-phosphate, $Ru(bpm)_2Cl_2$: Tris(2,2′-bipyrimide)ruthenium(II) dichloride, Ru(*p*-CF_3-bpy$)_3(BF_4)_2$: Tris(2,2′-(*p*CF_3)bipyridine)ruthenium(II) tetrafluoroborate.

Table S2. Reaction conditions for the photocatalytic direct decarboxylative chlorosulfonylation

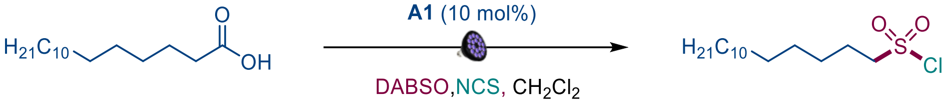

4e

Entry	Variations from standard conditions	Yield, %
1	none	67(57[b])
2	$PhCF_3$ instead of CH_2Cl_2	31
3	EtOAc instead of CH_2Cl_2	40
4	1,3-Dichloro-5,5-dimethylhydantoin instead of NCS	9
5	*N*-Chlorophthalimide instead of NCS	10
6	Trichloroisocyanuric acid instead of NCS	0
7	CCl_4 (0.5 mL) instead of NCS	30

[a] Reaction conditions: carboxylic acid (0.3 mmol), DABSO (0.36 mmol), acridine **A1** (10 mol%), NCS (0.75 mmol), CH_2Cl_2 (6 mL), LED light (400 nm), 9 h. Yield was determined by ^1H NMR spectroscopy with 1,4-dimethoxybenzene as an internal standard. [b] Isolated yield.

Table S3. Reaction conditions for the photocatalytic direct decarboxylative fluorosulfonylation

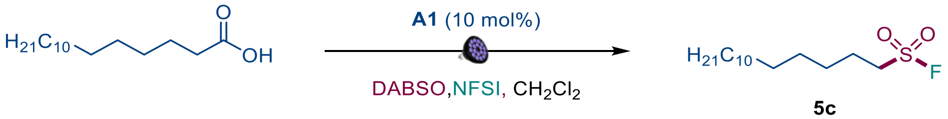

5c

Entry	Variations from standard conditions	Yield, %
1	none	68(60[b])
2	2.5 equiv. of DABSO instead of 1.5 equiv.	65
3	2.0 equiv. of NFSI instead of 1.5 equiv.	51
4	SelectFluor instead of NFSI	23
5	1-Fluoro-2,4,6-trimethylpyridinium tetrafluoroborate instead of NFSI	20
6	$PhCF_3$ instead of CH_2Cl_2	27
7	EtOAc instead of CH_2Cl_2	13

[a] Reaction conditions: carboxylic acid (0.3 mmol), DABSO (0.45 mmol), acridine **A1** (10 mol%), NFSI (0.45 mmol), CH_2Cl_2 (6 mL), LED light (400 nm), 12 h. Yield was determined by ^1H NMR spectroscopy with 1,4-dimethoxybenzene as an internal standard. [b] Isolated yield.

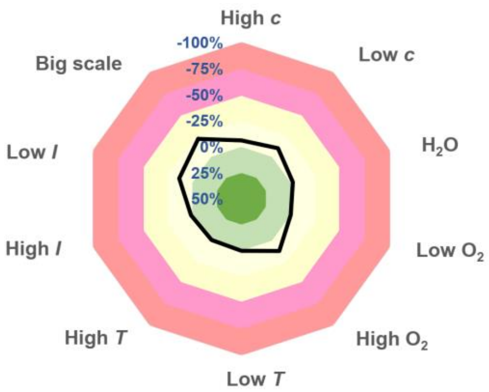

Figure S1. Evaluation of the sensitivity of the decarboxylative alkylsulfonylation reaction (see Table 1) to various reaction parameters. The screen was conducted as previously described.[14] The reaction was carried out at 0 °C for the low temperature experiment.

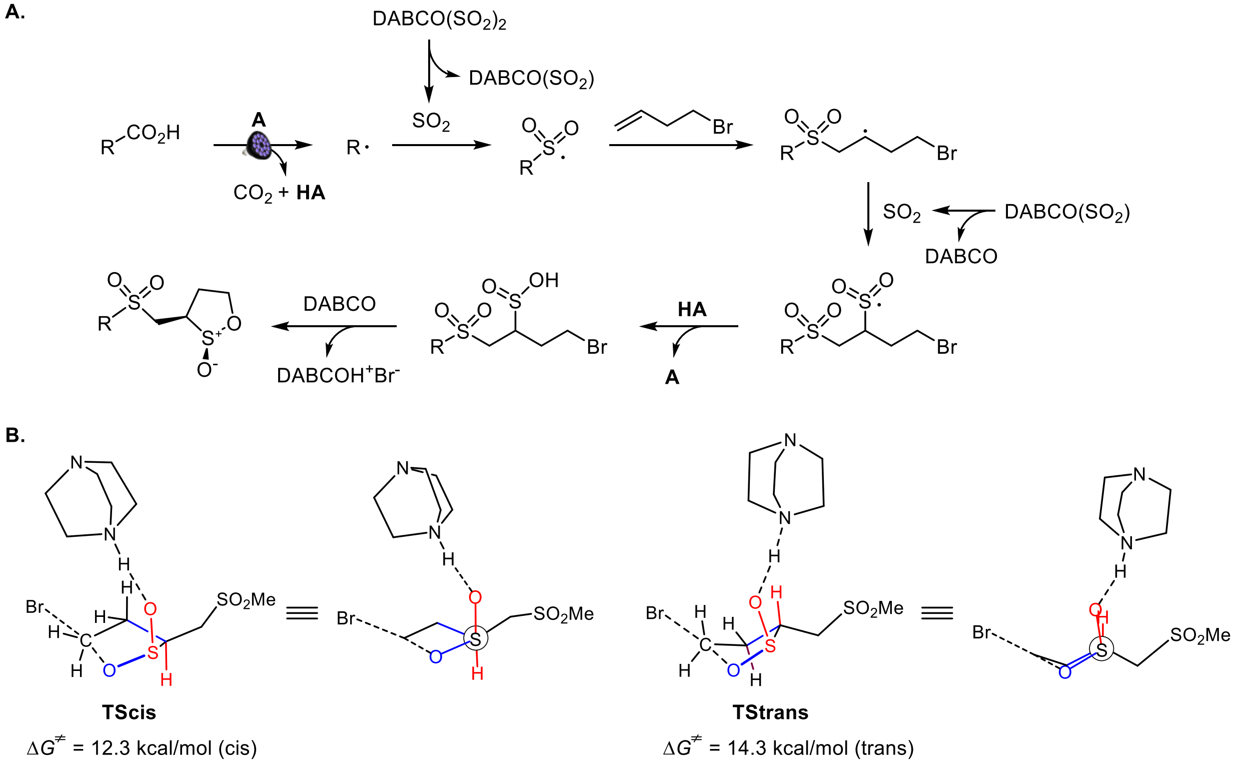

Figure S2. The *cis*-selective formation of 1,2-oxathiolane *S*-oxides in the decarboxylative sulfonylation with homoallyl bromide. **A.** Mechanism of formation of 1,2-oxathiolane *S*-oxides. **B.** Computed transition states **TScis** and **TStrans** for the DABCO-mediated *cis*- and *trans*-selective cyclization. Computational studies indicate that the *cis*-pathway proceeds over a lower barrier (cf., **TScis** and **TStrans**), in agreement with the experientally observed *cis*-selectivity. A noncovalent interactions analysis suggests that the *cis*-selectivity of the cyclization is due to the tortional strain caused by repulsive eclipsing interactions in **TStrans** that are relieved in staggered **TScis**.

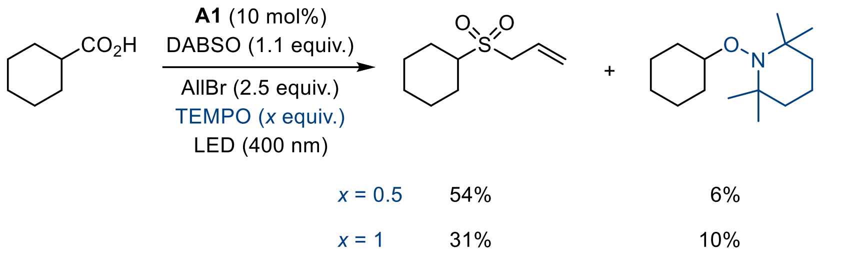

Figure S3. Alkyl radical trapping studies with TEMPO.

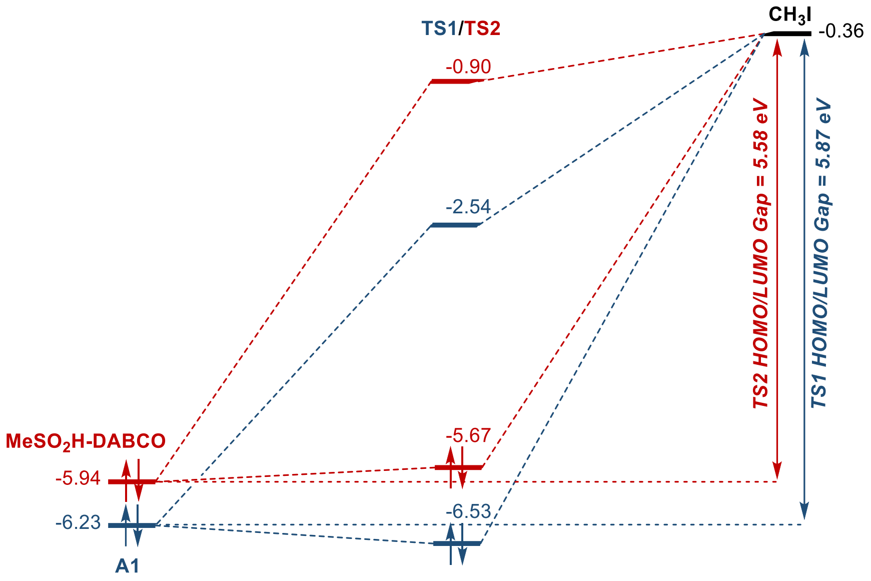

Figure S4. HOMO–LUMO gap for the alkylation of acridine **A1** and $MeSO_2H$-DABCO.

Quantum yield measurement

The photon flux of the photochemical setup was determined using the phenylglyoxylic acid chemical actinometer system.[15] Incident photon flux: 0.16 μmol photons per second. The direct decarboxylative sulfonylation reaction of palmitic acid was carried out as described in GP3. Yield was determined by ^{1}H NMR spectroscopy, using 1,4-dimethoxybenzene as an internal standard. Φ = 0.38.

Kinetic studies of the reactions of cyclohexanecarboxylic acid with TEMPO and various acridines

To a 10 mL test-tube, cyclohexanecarboxylic acid (38 mg, 0.3 mmol), TEMPO (37 mg, 0.24 mmol), acridine catalyst (0.03 mmol, 10 mol%), 1,3,5-trimethoxybenzene (17 mg, 0.1 mmol), and degassed dichloromethane (3 mL) were added. The test-tube was sealed with a rubber septum and the reaction mixture was irradiated with LED light (λ = 400 nm) while stirring at the rate of 1500 rpm at room temperature. A reaction aliquot was withdrawn after every 5 minutes, concentrated

under reduced pressure, and the product yield was determined by ^{1}H NMR with 1,3,5-trimethoxybenzene as an internal standard.

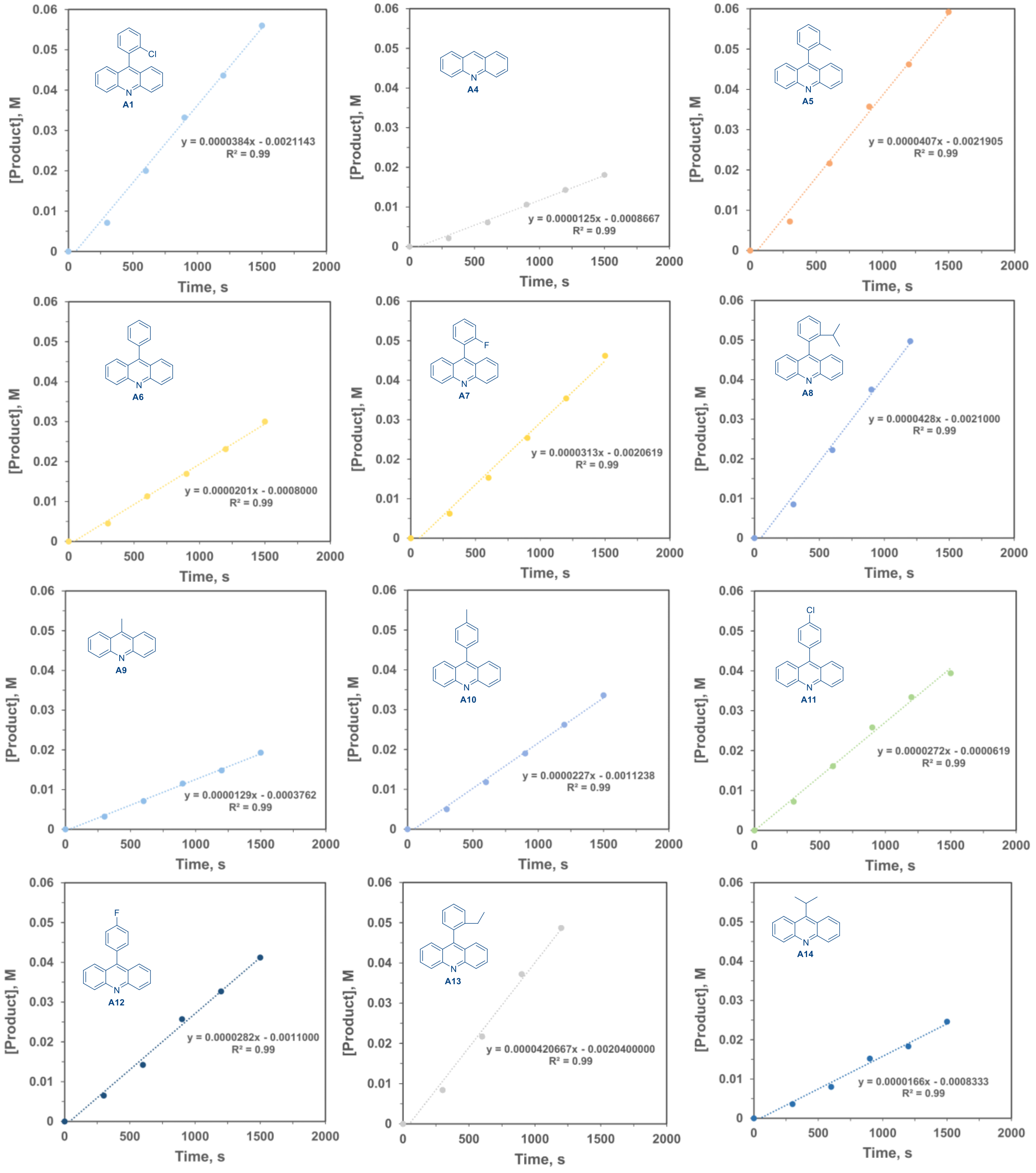

Figure S4. Kinetic plots of the reactions of cyclohexanecarboxylic acid with TEMPO and acridines **A1**, and **A4-A14**.

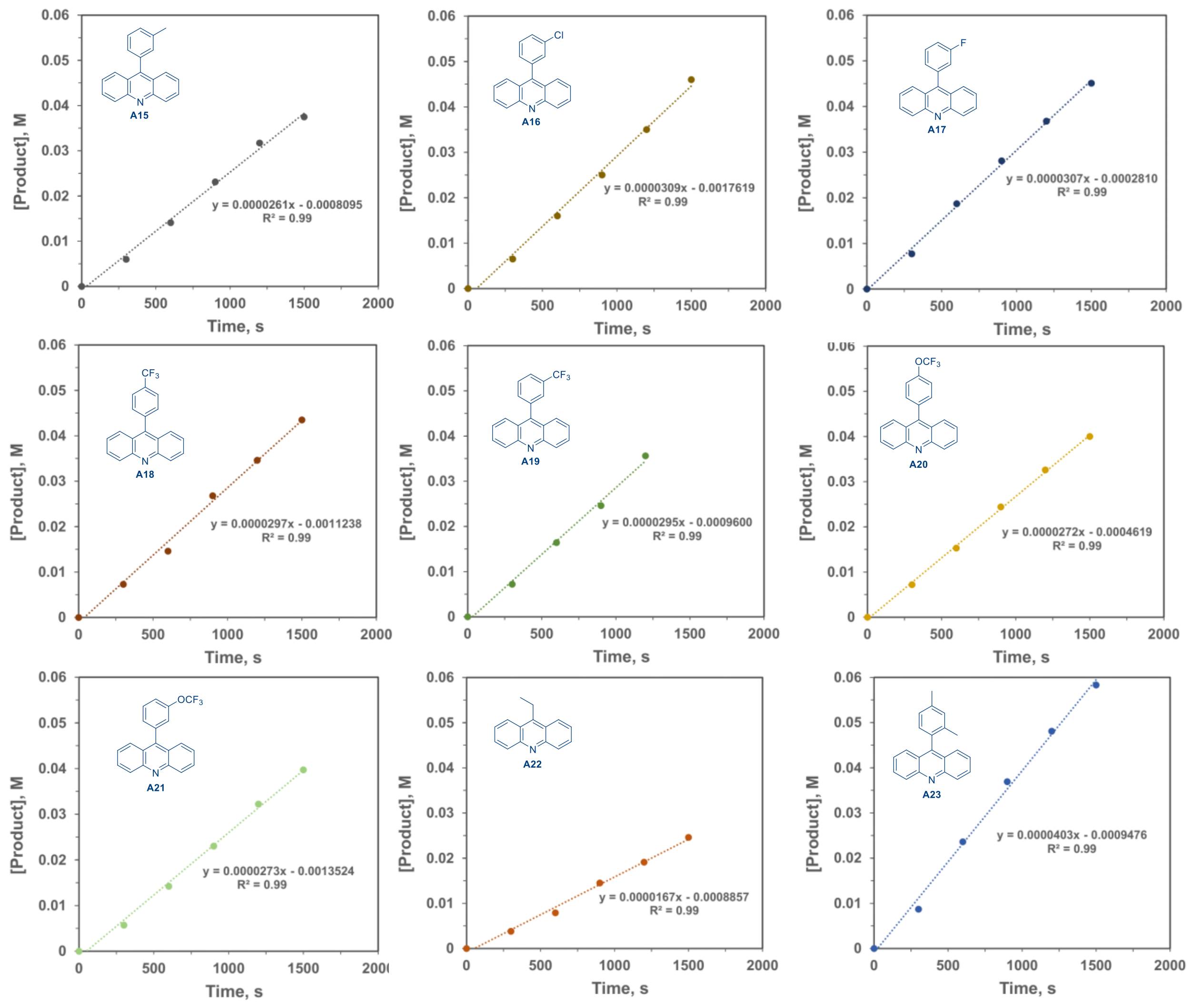

Figure S5. Kinetic plots of the reactions of cyclohexanecarboxylic acid with TEMPO and acridines **A15**-**A23**.

Acridine synthesis

9-(3-(Trifluoromethyl)phenyl)acridine (A19)

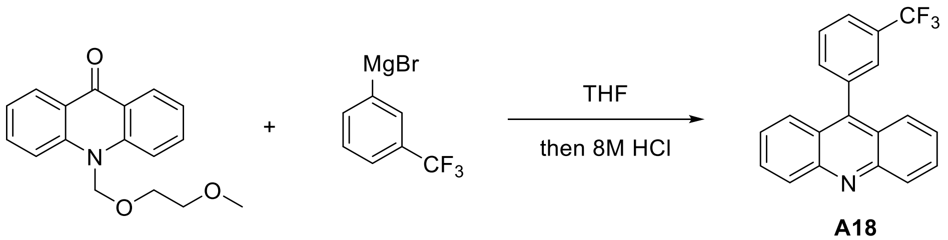

A18

To a 350 mL pressure vessel, 10-((2-methoxyethoxy)methyl)acridin-9(10*H*)-one (0.85 g, 3 mmol) and THF (60 mL) were added uner N_2 atmostphere. A solution of (3-(trifluoromethyl)phenyl)magnesium bromide (9 mmol, 3 equiv.) in THF was added slowly, and the vessel was capped and heated at 60 ˚C for 12 h. The reaction was allowed to cool down to room temperature before HCl 8 M (100 mL) was added, and the reaction was stirred for another 12 h at room temperature. After completion, the reaction was quenched with Na_2CO_3, and extracted with ethyl acetate (3 x 100 mL). The organic layer was washed with brine, separated, and dried over Na_2SO_4. Removal of the solvent and purification by silica gel chromatography (hexane/ ethyl acetate 9 : 1 v/v) afforded acridine **A19** as a yellow solid (0.59 g, 61%).

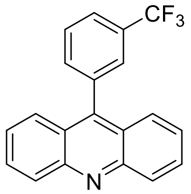

m.p.: 148–150 °C. – ^{1}H NMR (500 MHz, $CDCl_3$): 8.34 (2 H, d, *J* = 8.8 Hz), 7.95– 7.74 (5 H, m), 7.72–7.60 (3 H, m), 7.50 (2 H, ddd, *J* = 8.6, 6.6, 1.2 Hz) ppm. – ^{13}C NMR (125 MHz, $CDCl_3$): 148.6, 136.9, 133.8, 131.2 (q, *J* = 32.6 Hz), 130.3, 129.7, 129.2, 127.1 (q, *J* = 3.9 Hz), 126.3, 126.2, 125.4 (q, *J* = 4.2 Hz), 124.9, 124.0 (q, *J* = 272.4 Hz) ppm. – ^{19}F NMR (470 MHz, $CDCl_3$): –62.5 ppm. – IR: 3030, 1628, 1609, 1558, 1518, 1411, 1358, 1315, 1279, 1184, 1161, 1112, 1094 cm^{-1}. – HRMS: calcd for $C_{20}H_{12}F_3N$: 324.0995, found 324.0995 [M+H$^+$].

9-(3-(Trifluoromethoxy)phenyl)acridine (A21)

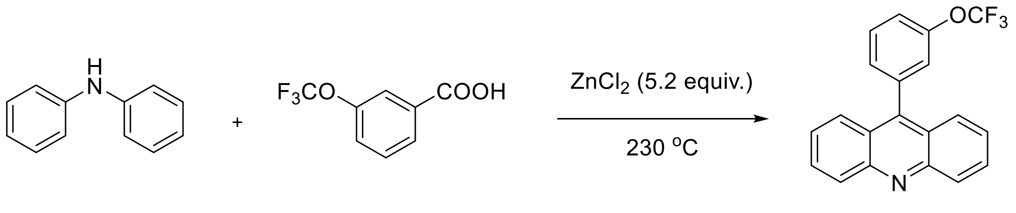

A20

According to the known procedure for synthesis of **A1**,[1] the reaction was carried out with diphenyl amine (0.61 g, 3.6 mmol), 3-(trifluoromethoxy)benzoic acid (2.1 g, 10 mmol, 3 equiv.), zinc chloride (2.6 g, 19 mmol, 5.2 equiv.) in a sand bath at 200 °C for 14 h. After completion, the

reaction was quenched with a saturated solution of ammonium hydroxide (50 mL) and then extracted with ethyl acetate (3 x 75 mL). The organic layer was washed with brine, separated, and dried over Na_2SO_4. Removal of the solvent and purification by silica gel chromatography (hexane/ ethyl acetate 9 : 1 v/v) afforded acridine **A21** as a yellow solid (0.44 g, 36%).

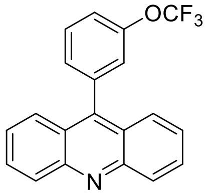

m.p.: 152–155 °C. – ^{1}H NMR (500 MHz, $CDCl_3$): 8.33 (2 H, d, *J* = 8.8 Hz), 7.82 (2 H, t, *J* = 7.7 Hz), 7.73–7.63 (3 H, m), 7.57–7.33 (5 H, m) ppm. – ^{13}C NMR (125 MHz, $CDCl_3$): 148.7, 143.7 (q, *J* = 1432.4 Hz), 130.2, 130.1, 129.7, 128.9, 126.3, 126.2, 124.8, 123.0, 120.8 ppm. – ^{19}F NMR (470 MHz, $CDCl_3$): –57.8 ppm. – IR: 3031, 1641, 1611, 1551, 1520, 1501, 1415, 1405, 1379, 1228, 1214, 1186 cm^{-1}. – HRMS: calcd for $C_{20}H_{12}F_3NO$: 340.0944, found 340.0942 [M+H$^+$].

Sulfones

(Allylsulfonyl)cyclohexane (1a)

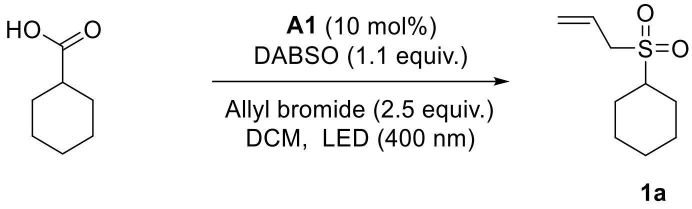

According to **GP1**, the reaction was carried out with cyclohexanecarboxylic acid (38 mg, 0.3 mmol), DABSO (79 mg, 0.33 mmol, 1.1 equiv.), acridine catalyst **A1** (9 mg, 0.03 mmol, 10 mol%), allyl bromide (91 mg, 0.75 mmol, 2.5 equiv.) in degassed dichloromethane (6 mL). The test-tube was capped, and the reaction mixture was irradiated with LED light (λ = 400 nm) while stirring at room temperature for 12 h. The reaction mixture was then quenched with a saturated solution of potassium hydrogen sulfate (2 mL) and extracted with ethyl acetate (3 × 10 mL). The organic layer was combined, dried over anhydrous sodium sulfate, concentrated under reduced pressure, and the remaining material was purified by flash chromatography on silica gel (hexane/ ethyl acetate 7 : 3 v/v) to give the sulfone product **1a** (52 mg, 92%) as a yellow liquid.

Synthesis with sodium metabisulfite

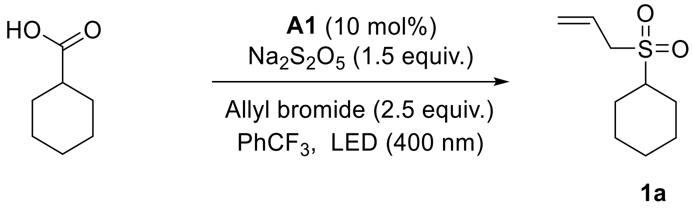

According to **GP1**, the reaction was carried out with cyclohexanecarboxylic acid (38 mg, 0.3 mmol), sodium metabisulfite (86 mg, 0.45 mmol, 1.5 equiv.), acridine catalyst **A1** (9 mg, 0.03 mmol, 10 mol%), allyl bromide (91 mg, 0.75 mmol, 2.5 equiv.) in degassed trifluorotoluene (3 mL). The test-tube was capped, and the reaction mixture was irradiated with LED light (λ = 400 nm) while stirring at room temperature for 12 h. The reaction mixture was then quenched with a saturated solution of potassium hydrogen sulfate (2 mL) and extracted with ethyl acetate (3 × 10 mL). The organic layer was combined, dried over anhydrous sodium sulfate, concentrated under reduced pressure, and the remaining material was purified by flash chromatography on silica gel (hexane/ ethyl acetate 7 : 3 v/v) to give the sulfone product **1a** (45 mg, 80%) as a yellow liquid.

Gram scale synthesis with sodium metabisulfite: According to **GP1**, the reaction was carried out with cyclohexanecarboxylic acid (1.28 g, 10 mmol), sodium metabisulfite (2.85 g, 15 mmol, 1.5 equiv.), acridine catalyst **A1** (289 mg, 1 mmol, 10 mol%), allyl bromide (3.02 g, 25 mmol, 2.5 equiv.) in degassed trifluorotoluene (80 mL). The test-tube was capped, and the reaction mixture was irradiated with LED light (λ = 400 nm) while stirring at room temperature for 12 h. The reaction mixture was then quenched with a saturated solution of potassium hydrogen sulfate (20 mL) and extracted with ethyl acetate (3 × 50 mL). The organic layer was combined, dried over anhydrous sodium sulfate, concentrated under reduced pressure, and the remaining material was purified by flash chromatography on silica gel (hexane/ ethyl acetate 7 : 3 v/v) to give the sulfone product **1a** (1.35 g, 70%) as a yellow liquid.

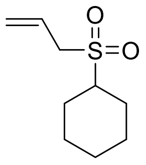

^{1}H NMR (500 MHz, $CDCl_3$): 5.89 (1 H, ddt, *J* = 17.4, 10.2, 7.3 Hz), 5.47–5.36 (2 H, m), 3.66 (2 H, d, *J* = 7.4 Hz), 2.89 (1 H, tt, *J* = 12.2, 3.5 Hz), 2.14–2.05 (2 H, m), 1.89 (2 H, dt, *J* = 11.8, 2.8 Hz), 1.71–1.65 (1 H, m), 1.52 (2 H, qd, *J* = 12.5, 3.5 Hz), 1.32–1.14 (3 H, m) ppm. – ^{13}C NMR (125 MHz, $CDCl_3$): 125.0, 124.3, 59.5, 54.7, 25.1, 25.0, 24.9 ppm. – IR: 2974, 2934, 2919, 2857, 2863, 1737, 1637, 1453, 1309, 1269, 1128, 1002, 938, 748 cm^{-1}. – HRMS: calcd for $C_9H_{17}O_2S$: 189.0944, found 189.0946 [M+H$^+$].

1-(2-(Allylsulfonyl)ethyl)-4-methoxybenzene (1b)

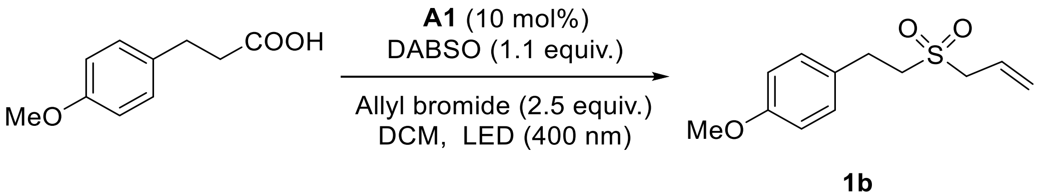

1b

According to **GP1**, the reaction was carried out with 3-(4-methoxyphenyl)propanoic acid (54 mg, 0.3 mmol), DABSO (79 mg, 0.33 mmol, 1.1 equiv.), acridine catalyst **A1** (9 mg, 0.03 mmol, 10

Go back to table of contents

mol%), allyl bromide (91 mg, 0.75 mmol, 2.5 equiv.) in degassed dichloromethane (6 mL). The test-tube was capped and the reaction mixture was irradiated with LED light (λ = 400 nm) while stirring at room temperature for 12 h. The reaction mixture was then quenched with a saturated solution of potassium hydrogen sulfate (2 mL) and extracted with ethyl acetate (3 × 10 mL). The organic layer was combined, dried over anhydrous sodium sulfate, concentrated under reduced pressure, and the remaining material was purified by flash chromatography on silica gel (hexane/ ethyl acetate 7 : 3 v/v) to give the sulfone product **1b** (64 mg, 89%) as a yellow liquid.

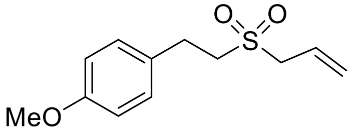

^{1}H NMR (500 MHz, $CDCl_3$): 7.22–7.00 (2 H, m), 6.91–6.70 (2 H, m), 5.90 (1 H, ddt, *J* = 17.4, 10.1, 7.4 Hz), 5.46 (1 H, dq, *J* = 10.3, 1.0 Hz), 5.34 (1 H, dq, *J* = 17.2, 1.2 Hz), 3.79 (3 H, s), 3.61 (2 H, dt, *J* = 7.4, 1.1 Hz), 3.26–3.14 (2 H, m), 3.14–3.04 (2 H, m) ppm. – ^{13}C NMR (125 MHz, $CDCl_3$): 158.8, 129.6, 129.6, 125.2, 124.7, 114.4, 58.2, 55.4, 52.9, 27.3 ppm. – IR: 3065, 2965, 2876, 1612, 1513, 1422, 1311, 1265, 1248, 1179, 1124, 1033, 937 cm^{-1}. – HRMS: calcd for $C_{12}H_{16}O_3SNa$: 263.0712, found 263.0710 [M+Na$^+$].

Methyl 5-(allylsulfonyl)pentanoate (1c)

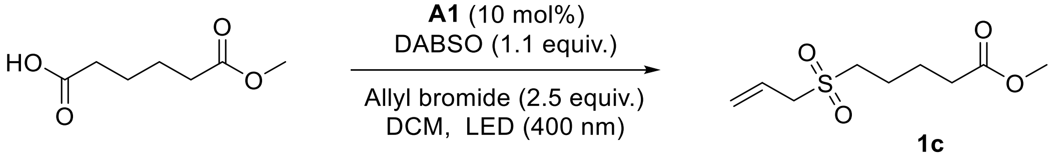

1c

According to **GP1**, the reaction was carried out with 6-methoxy-6-oxohexanoic acid (48 mg, 0.3 mmol), DABSO (79 mg, 0.33 mmol, 1.1 equiv.), acridine catalyst **A1** (9 mg, 0.03 mmol, 10 mol%), allyl bromide (91 mg, 0.75 mmol, 2.5 equiv.) in degassed dichloromethane (6 mL). The test-tube was capped, and the reaction mixture was irradiated with LED light (λ= 400 nm) while stirring at room temperature for 12 h. The reaction mixture was then quenched with a saturated solution of potassium hydrogen sulfate (2 mL) and extracted with ethyl acetate (3 × 10 mL). The organic layer was combined, dried over anhydrous sodium sulfate, concentrated under reduced pressure, and the remaining material was purified by flash chromatography on silica gel (hexane/ ethyl acetate 3 : 2 v/v) to give the sulfone product **1c** (52 mg, 79%) as a colorless oil.

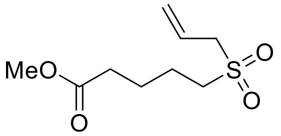

^{1}H NMR (300 MHz, $CDCl_3$): 5.90 (1 H, ddt, *J* = 17.4, 10.2, 7.4 Hz), 5.48–5.34 (2 H, m), 3.66 (5 H, m), 3.02–2.85 (2 H, m), 2.33 (2 H, t, *J* = 7.0 Hz), 1.93–1.65 (4 H, m) ppm. – ^{13}C NMR (75 MHz, $CDCl_3$): 173.2, 125.2, 124.6, 57.7, 51.7, 50.8, 33.3, 23.7, 21.3 ppm. – IR: 2953, 2929, 2887, 2848, 1731, 1666, 1639, 1460, 1438, 1309, 1289,

1199, 1176, 1123, 1083, 995, 940, 882 cm^{-1}. – HRMS: calcd for $C_9H_{17}O_4S$: 221.0842, found 221.0856 [M+H$^+$].

_N_-(5-(Allylsulfonyl)pentyl)-4-chlorobenzamide (1d)

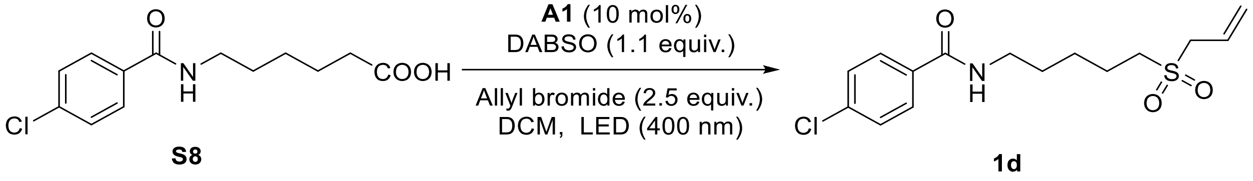

According to **GP1**, the reaction was carried out with acid **S1** (81 mg, 0.3 mmol), DABSO (79 mg, 0.33 mmol, 1.1 equiv.), acridine catalyst **A1** (9 mg, 0.03 mmol, 10 mol%), allyl bromide (91 mg, 0.75 mmol, 2.5 equiv.) in degassed dichloromethane (6 mL). The test-tube was capped and the reaction mixture was irradiated with LED light (λ = 400 nm) while stirring at room temperature for 12 h. The reaction mixture was then quenched with a saturated solution of potassium hydrogen sulfate (2 mL) and extracted with ethyl acetate (3 × 10 mL). The organic layer was combined, dried over anhydrous sodium sulfate, concentrated under reduced pressure, and the remaining material was purified by flash chromatography on silica gel (hexane/ ethyl acetate 3 : 7 v/v) to give the sulfone product **1d** (87 mg, 88%) as a white solid.

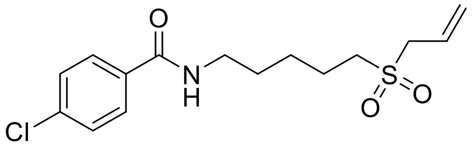

m.p.: 56–58 °C. – ^{1}H NMR (500 MHz, $CDCl_3$): 7.70 (2 H, d, *J* = 8.6 Hz), 7.36 (2 H, d, *J* = 8.5 Hz), 6.53 (1 H, d, *J* = 6.0 Hz), 5.90 (1 H, ddt, *J* = 17.5, 10.2, 7.4 Hz), 5.56–5.31 (2 H, m), 3.68 (2 H, dt, *J* = 7.3, 1.0 Hz), 3.42 (2 H, td, *J* = 7.0, 5.8 Hz), 3.03–2.88 (2 H, m), 1.93–1.76 (2 H, m), 1.68–1.42 (4 H, m) ppm. – ^{13}C NMR (125 MHz, $CDCl_3$): 166.7, 137.7, 133.0, 128.8, 128.5, 125.1, 124.8, 58.0, 51.0, 39.6, 29.1, 25.8, 21.4 ppm. – IR: 3053, 2980, 1737, 1421, 1264, 895, 735 cm^{-1}. – HRMS: calcd for $C_{15}H_{20}ClNO_3S$: 330.0925, found 330.0924 [M+H$^+$].

3-(3,3,3-Trifluoropropyl)sulfonyl)prop-1-ene (1e)

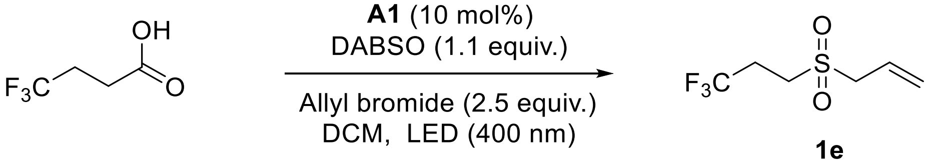

According to **GP1**, the reaction was carried out with 4,4,4-trifluorobutanoic acid (43 mg, 0.3 mmol), DABSO (79 mg, 0.33 mmol, 1.1 equiv.), acridine catalyst **A1** (9 mg, 0.03 mmol, 10 mol%), allyl bromide (91 mg, 0.75 mmol, 2.5 equiv.) in degassed dichloromethane (6 mL). The test-tube

was capped, and the reaction mixture was irradiated with LED light (λ= 400 nm) while stirring at room temperature for 12 h. The reaction mixture was then quenched with a saturated solution of potassium hydrogen sulfate (2 mL) and extracted with ethyl acetate (3 × 10 mL). The organic layer was combined, dried over anhydrous sodium sulfate, concentrated under reduced pressure, and the remaining material was purified by flash chromatography on silica gel (hexane/ ethyl acetate 7 : 3 v/v) to give the sulfone product **1e** (50 mg, 83%) as a slightly yellow solid.

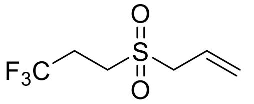

m.p.: 25–27 °C. – ^{1}H NMR (500 MHz, $CDCl_3$): 5.93 (1 H, ddt, *J* = 17.4, 10.2, 7.4 Hz), 5.58–5.46 (2 H, m), 3.77 (2 H, d, *J* = 7.4 Hz), 3.20–3.13 (2 H, m), 2.69–2.56 (2 H, m) ppm. – ^{13}C NMR (125 MHz, $CDCl_3$): 125.7 (q, $^{1}J_{C\text{-}F}$ = 276.1 Hz), 125.5, 124.6, 58.4, 44.3 (q, $^{3}J_{C\text{-}F}$ = 3.1 Hz), 27.0 (q, $^{2}J_{C\text{-}F}$ = 31.8 Hz) ppm. – ^{19}F NMR (470 MHz, $CDCl_3$): -66.07 (t, *J* = 10.0 Hz) ppm. – IR: 2991, 2974, 2926, 2859, 1641, 1445, 1386, 1294, 1276, 1250, 1214, 1134, 1093, 962, 884, 850 cm^{-1}. – HRMS: calcd for $C_6H_9F_3NaO_2S$: 225.0168, found 225.0173 [M+Na$^+$].

N-(5-(Allylsulfonyl)pentyl)-4-(trifluoromethyl)benzamide (1f)

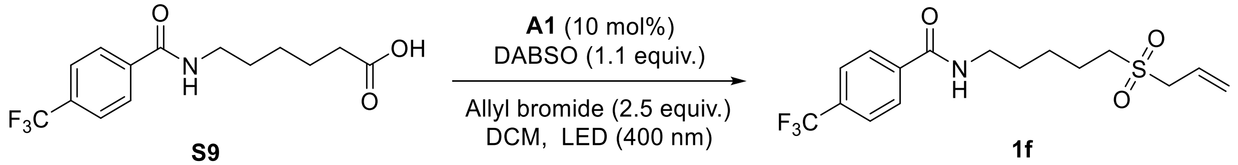

According to **GP1**, the reaction was carried out with acid **S9** (91 mg, 0.3 mmol), DABSO (79 mg, 0.33 mmol, 1.1 equiv.), acridine catalyst **A1** (9 mg, 0.03 mmol, 10 mol%), allyl bromide (91 mg, 0.75 mmol, 2.5 equiv.) in degassed dichloromethane (6 mL). The test-tube was capped, and the reaction mixture was irradiated with LED light (λ = 400 nm) while stirring at room temperature for 12 h. The reaction mixture was then quenched with a saturated solution of potassium hydrogen sulfate (2 mL) and extracted with ethyl acetate (3 × 10 mL). The organic layer was combined, dried over anhydrous sodium sulfate, concentrated under reduced pressure, and the remaining material was purified by flash chromatography on silica gel (hexane/ ethyl acetate 4 : 6 v/v) to give the sulfone product **1f** (83 mg, 76%) as a slightly yellow solid.

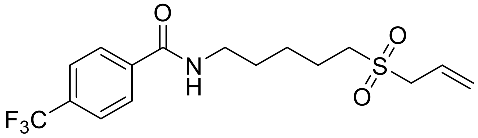

m.p.: 95–97 °C. – ^{1}H NMR (500 MHz, $CDCl_3$): 7.88 (2 H, d, *J* = 8.1 Hz), 7.69 (2 H, d, *J* = 8.1 Hz), 6.34 (1 H, brs), 5.93 (1 H, ddt, *J* = 17.4, 10.2, 7.4 Hz), 5.58–5.35 (2 H, m), 3.71 (2 H, d, *J* = 7.4 Hz), 3.49 (2 H, q, *J* = 6.6 Hz), 3.04–2.90 (2 H, m), 1.95–1.85 (2 H, m), 1.69 (2 H, quint., *J* = 7.1 Hz), 1.62–1.52 (2 H, m) ppm. – ^{13}C NMR (125 MHz, $CDCl_3$): 166.5, 137.9, 133.2 (q, $^{2}J_{C\text{-}F}$ = 32.7 Hz), 127.5, 125.7 (q, $^{3}J_{C\text{-}F}$ = 3.8 Hz), 124.8, 123.7 (q, $^{1}J_{C\text{-}F}$ = 271.2 Hz), 58.0, 50.9, 39.7, 29.0, 25.7, 21.4

ppm. – ^{19}F NMR (470 MHz, $CDCl_3$): -63.21 (d, *J* = 11.6 Hz) ppm. – IR: 3331, 2976, 2938, 2865, 1634, 1539, 1508, 1333, 1315, 1290, 1272, 1166, 1126, 1108, 1073, 1019, 933, 860, 774 cm^{-1}. – HRMS: calcd for $C_{16}H_{20}F_3NNaO_3S$: 386.1008, found 386.1013 [M+Na$^+$].

4-(Allylsulfonyl)-*N*-(3-(4,4,5,5-tetramethyl-1,3,2-dioxaborolan-2-yl)phenyl)butanamide (1g)

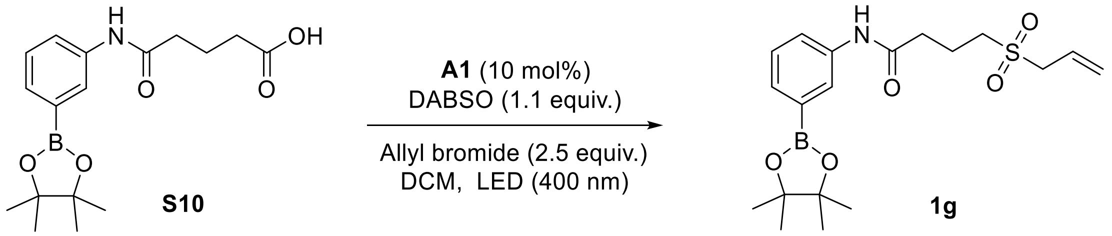

According to **GP1**, the reaction was carried out with acid **S10** (100 mg, 0.3 mmol), DABSO (79 mg, 0.33 mmol, 1.1 equiv.), acridine catalyst **A1** (9 mg, 0.03 mmol, 10 mol%), allyl bromide (91 mg, 0.75 mmol, 2.5 equiv.) in degassed dichloromethane (6 mL). The test-tube was capped, and the reaction mixture was irradiated with LED light (λ = 400 nm) while stirring at room temperature for 12 h. The reaction mixture was then quenched with a saturated solution of potassium hydrogen sulfate (2 mL) and extracted with ethyl acetate (3 × 10 mL). The organic layer was combined, dried over anhydrous sodium sulfate, concentrated under reduced pressure, and the remaining material was purified by flash chromatography on silica gel (hexane/ ethyl acetate 4 : 6 v/v) to give the sulfone product **1g** (83 mg, 70%) as a slightly yellow solid.

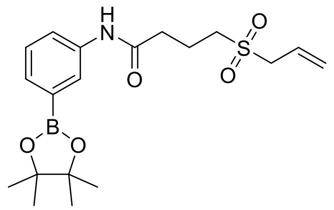

m.p.: 78–80 °C. – ^{1}H NMR (500 MHz, $CDCl_3$): 7.82–7.70 (3 H, m), 7.53 (1 H, d, *J* = 7.2 Hz), 7.32 (1 H, t, *J* = 7.7 Hz), 5.91 (1 H, ddt, *J* = 17.5, 10.2, 7.4 Hz), 5.50–5.41 (2 H, m), 3.72 (2 H, d, *J* = 7.3 Hz), 3.09 (2 H, t, *J* = 7.2 Hz), 2.58 (2 H, t, *J* = 6.9 Hz), 2.25 (2 H, quint., *J* = 7.1 Hz), 1.32 (12 H, s) ppm. – ^{13}C NMR (125 MHz, $CDCl_3$): 169.8, 137.3, 130.8, 128.6, 126.0, 125.2, 124.8, 123.2, 84.1, 58.1, 49.9, 35.0, 25.0, 18.1 ppm. – ^{11}B NMR (160 MHz, $CDCl_3$) 31.74 ppm. – IR: 3328, 3091, 2978, 2925, 1669, 1610, 1427, 1357, 1312, 1167, 1142, 1077, 851, 707 cm^{-1}. – HRMS: calcd for $C_{19}H_{29}BNO_5S$: 394.1854, found 394.1865 [M+H$^+$].

3-(3-(Allylsulfonyl)propyl)-1*H*-indole (1h)

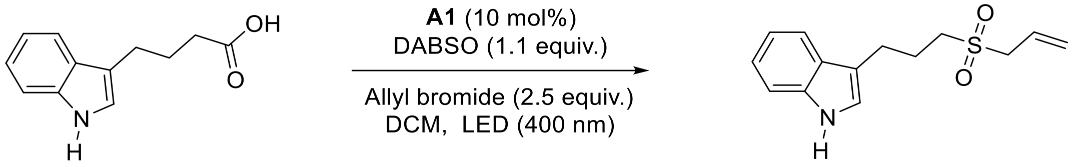

1h

According to **GP1**, the reaction was carried out with 4-(1H-indol-3-yl)butanoic acid (61 mg, 0.3 mmol), DABSO (79 mg, 0.33 mmol, 1.1 equiv.), acridine catalyst **A1** (9 mg, 0.03 mmol, 10 mol%), allyl bromide (91 mg, 0.75 mmol, 2.5 equiv.) in degassed dichloromethane (6 mL). The test-tube was capped, and the reaction mixture was irradiated with LED light (λ = 400 nm) while stirring at room temperature for 12 h. The reaction mixture was then quenched with a saturated solution of potassium hydrogen sulfate (2 mL) and extracted with ethyl acetate (3 × 10 mL). The organic layer was combined, dried over anhydrous sodium sulfate, concentrated under reduced pressure, and the remaining material was purified by flash chromatography on silica gel (hexane/ ethyl acetate 4 : 6 v/v) to give the sulfone product **1h** (54 mg, 68%) as a colorless liquid.

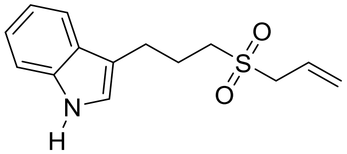

^{1}H NMR (300 MHz, $CDCl_3$): 8.16 (1 H, s), 7.57 (1 H, dd, *J* = 7.8, 1.1 Hz), 7.37 (1 H, dt, *J* = 8.1, 1.0 Hz), 7.21 (1 H, ddd, *J* = 8.1, 7.0, 1.3 Hz), 7.13 (1 H, ddd, *J* = 8.0, 7.0, 1.2 Hz), 6.99 (1 H, d, *J* = 2.2 Hz), 5.86 (1 H, ddt, *J* = 17.4, 10.2, 7.4 Hz), 5.38 (1 H, dq, *J* = 10.2, 1.0 Hz), 5.28 (1 H, dq, *J* = 17.0, 1.2 Hz), 3.64 (1 H, d, *J* = 7.4 Hz), 3.09–2.84 (4 H, m), 2.38–2.17 (2 H, m) ppm. – ^{13}C NMR (75 MHz, $CDCl_3$): 136.5, 127.2, 125.1, 124.6, 122.2, 122.0, 119.5, 118.6, 114.0, 111.4, 57.7, 50.7, 23.8, 22.5 ppm. – IR: 3378, 3081, 3056, 2951, 2920, 2872, 2852, 1658, 1643, 1457, 1422, 1307, 1288, 1242, 1126, 1093, 1015, 993, 885, 744 cm^{-1}. – HRMS: calcd for $C_{14}H_{18}NO_2S$: 264.1053, found 264.1058 [M+H$^+$].

4-(Allylsulfonyl)-1-(5-methylthiophen-2-yl)butan-1-one (1i)

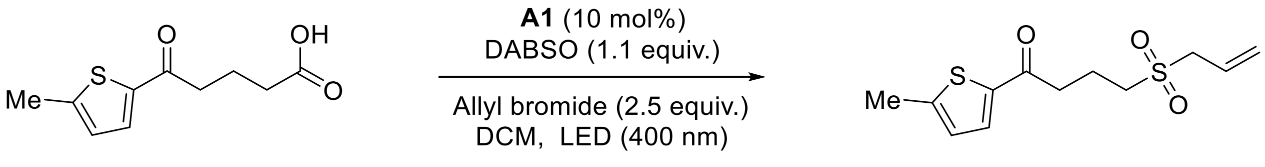

S11 **1i**

According to **GP1**, the reaction was carried out with acid **S11** (64 mg, 0.3 mmol), DABSO (79 mg, 0.33 mmol, 1.1 equiv.), acridine catalyst **A1** (9 mg, 0.03 mmol, 10 mol%), allyl bromide (91 mg, 0.75 mmol, 2.5 equiv.) in degassed dichloromethane (6 mL). The test-tube was capped, and the

reaction mixture was irradiated with LED light (λ = 400 nm) while stirring at room temperature for 12 h. The reaction mixture was then quenched with a saturated solution of potassium hydrogen sulfate (2 mL) and extracted with ethyl acetate (3 × 10 mL). The organic layer was combined, dried over anhydrous sodium sulfate, concentrated under reduced pressure, and the remaining material was purified by flash chromatography on silica gel (hexane/ ethyl acetate 1 : 1 v/v) to give the sulfone product **1i** (78 mg, 95%) as a slightly yellow liquid.

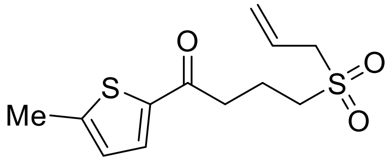

^{1}H NMR (300 MHz, $CDCl_3$): 7.51 (1 H, d, *J* = 3.8 Hz), 6.77 (1 H, dq, *J* = 3.8, 1.0 Hz), 5.90 (1 H, ddt, *J* = 16.3, 10.9, 7.3 Hz), 5.52–5.29 (2 H, m), 3.70 (2 H, dt, *J* = 7.3, 1.1 Hz), 3.14–2.84 (4 H, m), 2.49 (3 H, d, *J* = 1.1 Hz), 2.33–2.08 (2 H, m) ppm. – ^{13}C NMR (75 MHz, $CDCl_3$): 191.0, 150.1, 141.4, 132.9, 127.0, 124.9, 124.8, 57.7, 50.3, 36.4, 16.9, 16.0 ppm. – IR: 3091, 3023, 2972, 2925, 1738, 1656, 1456, 1365, 1311, 1289, 1238, 1122, 1079, 940, 888 cm^{-1}. – HRMS: calcd for $C_{12}H_{16}NaO_3S_2$: 295.0433, found 295.0436 [M+Na$^+$].

1,7-Bis(allylsulfonyl)heptane (1j)

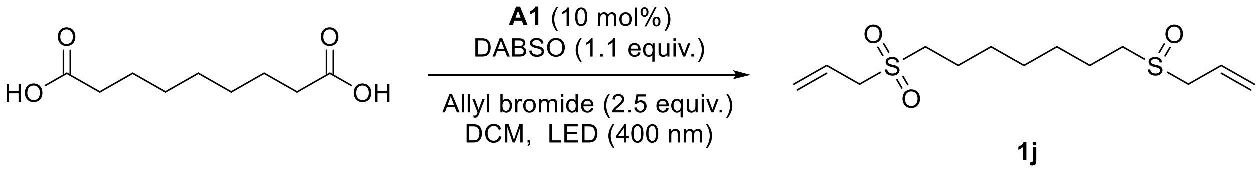

1j

According to **GP1**, the reaction was carried out with azelaic acid (28 mg, 0.15 mmol), DABSO (40 mg, 0.165 mmol, 1.1 equiv.), acridine catalyst **A1** (5 mg, 0.015 mmol, 10 mol%), allyl bromide (45 mg, 0.375 mmol, 2.5 equiv.) in degassed dichloromethane (6 mL). The test-tube was capped, and the reaction mixture was irradiated with LED light (λ = 400 nm) while stirring at room temperature for 12 h. The reaction mixture was then quenched with a saturated solution of potassium hydrogen sulfate (2 mL) and extracted with ethyl acetate (3 × 10 mL). The organic layer was combined, dried over anhydrous sodium sulfate, concentrated under reduced pressure, and the remaining material was purified by flash chromatography on silica gel (hexane/ ethyl acetate 3 : 7 v/v) to give the sulfone product **1j** (43 mg, 63%) as a yellow solid.

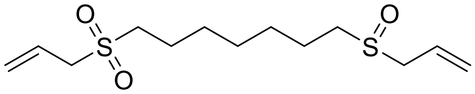

m.p.: 55–57 °C. – ^{1}H NMR (500 MHz, $CDCl_3$): 5.93 (2 H, ddt, *J* = 17.1, 9.3, 6.8 Hz), 5.47 (4 H, td, *J* = 17.4, 16.4, 5.0 Hz), 3.69 (4 H, d, *J* = 7.6 Hz), 2.93 (4 H, M, *J* = 9.3 Hz), 1.82 (4 H, quint., *J* = 7.8 Hz), 1.51–1.32 (6 H, m) ppm. – ^{13}C NMR (125 MHz, $CDCl_3$) 125.3, 124.6, 57.9, 51.1, 28.6, 28.1, 21.6 ppm. – IR: 2984, 2936,

2873, 1738, 1639, 1452, 1421, 1405, 1321, 1269, 1211, 1193, 1118, 989, 933, 921, 777, 750 cm^{-1}. – HRMS: calcd for $C_{13}H_{24}NaO_4S_2$: 331.1008, found 331.1005 [M+Na$^+$].

1,4-Bis(allylsulfonyl)butane (1k)

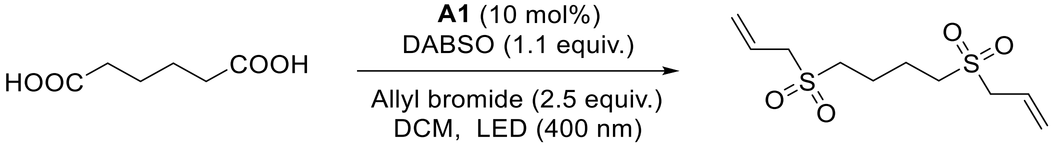

According to **GP1**, the reaction was carried out with adipic acid (44 mg, 0.3 mmol), DABSO (79 mg, 0.33 mmol, 1.1 equiv.), acridine catalyst **A1** (9 mg, 0.03 mmol, 10 mol%), allyl bromide (91 mg, 0.75 mmol, 2.5 equiv.) in degassed dichloromethane (6 mL). The test-tube was capped and the reaction mixture was irradiated with LED light (λ = 400 nm) while stirring at room temperature for 12 h. The reaction mixture was then quenched with a saturated solution of potassium hydrogen sulfate (2 mL) and extracted with ethyl acetate (3 × 10 mL). The organic layer was combined, dried over anhydrous sodium sulfate, concentrated under reduced pressure, and the remaining material was purified by flash chromatography on silica gel (hexane/ ethyl acetate 3 : 7 v/v) to give the sulfone product **1k** (50 mg, 63%) as a yellow solid.

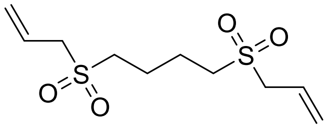

m.p.: 45–48 °C. – ^{1}H NMR (500 MHz, $CDCl_3$): 5.92 (2 H, ddt, *J* = 17.4, 10.1, 7.4 Hz), 5.58–5.34 (4 H, m), 3.71 (4 H, d, *J* = 7.4 Hz), 3.00 (4 H, t, *J* = 6.6 Hz), 2.11–1.90 (4 H, m) ppm. – ^{13}C NMR (125 MHz, $CDCl_3$): 125.0, 58.1, 50.3, 20.8 ppm. – IR: 3054, 2987, 2889, 2165, 1738, 1421, 1364, 1261, 1134 cm^{-1}. – HRMS: calcd for $C_{10}H_{18}NaO_4S_2$: 289.0539, found 289.0543 [M+Na$^+$].

1-(Allylsulfonyl)tetracosa-9,11-diyne (1l)

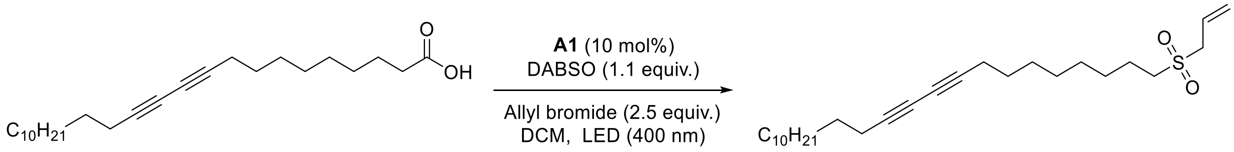

According to **GP1**, the reaction was carried out pentacosa-10,12-diynoic acid (56 mg, 0.15 mmol), DABSO (40 mg, 0.165 mmol, 1.1 equiv.), acridine catalyst **A1** (5 mg, 0.015 mmol, 10 mol%), allyl bromide (45 mg, 0.375 mmol, 2.5 equiv.) in degassed dichloromethane (6 mL). The test-tube was capped, and the reaction mixture was irradiated with LED light (λ = 400 nm) while stirring at room temperature for 12 h. The reaction mixture was then quenched with a saturated solution of

potassium hydrogen sulfate (2 mL) and extracted with ethyl acetate (3 × 10 mL). The organic layer was combined, dried over anhydrous sodium sulfate, concentrated under reduced pressure, and the remaining material was purified by flash chromatography on silica gel (hexane/ ethyl acetate 1 : 4 v/v) to give the sulfone product **1l** (41 mg, 63%) as a green solid.

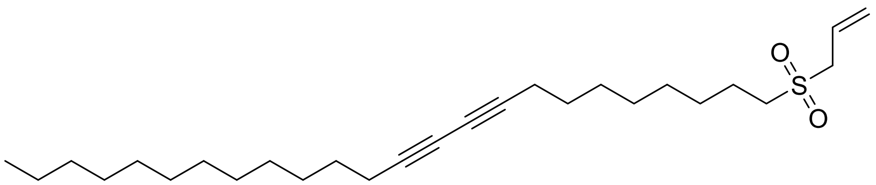

m.p.: 41–43°C. – ^{1}H NMR (300 MHz, $CDCl_3$): 5.93 (1 H, ddt, *J* = 17.4, 10.2, 7.4 Hz), 5.51–5.37 (2 H), 3.68 (2 H, d, *J* = 7.4 Hz), 2.96–2.85 (2 H, m), 2.22 (4 H, t, *J* = 6.9 Hz), 1.80 (2 H, tt, *J* = 8.0, 6.2 Hz), 1.55–1.15 (30 H, m), 0.93–0.78 (3 H, m) ppm. – ^{13}C NMR (75 MHz, $CDCl_3$): 125.4, 124.5, 77.7, 77.3, 65.5, 65.3, 57.7, 51.3, 32.0, 29.7, 29.6, 29.4, 29.2, 29.1, 29.0, 28.8, 28.7, 28.4, 28.3, 22.8, 21.8, 19.3, 19.2, 14.2 ppm. – IR: 2961, 2938, 2847, 1645, 1459, 1420, 1320, 1305, 1287, 1195, 1123, 1021, 921, 724 cm^{-1}. – HRMS: calcd for $C_{27}H_{47}O_2S$: 435.3291, found 435.3288 [M+H$^+$].

(Allylsulfonyl)cyclobutane (1m)

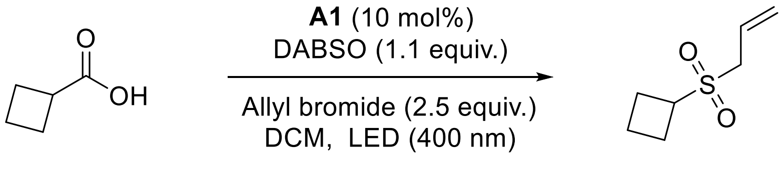

1m

According to **GP1**, the reaction was carried out with cyclobutanecarboxylic acid (30 mg, 0.3 mmol), DABSO (79 mg, 0.33 mmol, 1.1 equiv.), acridine catalyst **A1** (9 mg, 0.03 mmol, 10 mol%), allyl bromide (91 mg, 0.75 mmol, 2.5 equiv.) in degassed dichloromethane (6 mL). The test-tube was capped, and the reaction mixture was irradiated with LED light (λ = 400 nm) while stirring at room temperature for 12 h. The reaction mixture was then quenched with a saturated solution of potassium hydrogen sulfate (2 mL) and extracted with ethyl acetate (3 × 10 mL). The organic layer was combined, dried over anhydrous sodium sulfate, concentrated under reduced pressure, and the remaining material was purified by flash chromatography on silica gel (hexane/ ethyl acetate 7 : 3 v/v) to give the sulfone product **1m** (45 mg, 94%) as a colorless liquid.

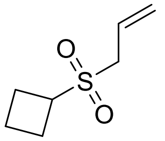

^{1}H NMR (500 MHz, $CDCl_3$): 5.85 (1 H, ddt, *J* = 17.4, 10.1, 7.4 Hz), 5.43–5.32 (2 H), 3.82 (1 H, quint., *J* = 8.3 Hz), 3.58 (2 H, dt, *J* = 7.4, 1.1 Hz), 2.60–2.49 (2 H, m), 2.26–2.17 (2 H, m), 2.08–1.95 (2 H, m) ppm. – ^{13}C NMR (125 MHz, $CDCl_3$): 125.0, 124.2,

55.8, 52.9, 22.4, 17.4 ppm. – IR: 2976, 2941, 2858, 1738, 1639, 1454, 1310, 1271, 1128, 1082, 994, 938, 750 cm^{-1}. – HRMS: calcd for $C_7H_{13}O_2S$: 161.0631, found 161.0638 [M+H$^+$].

((1*S**,2*R**)-2-(Allylsulfonyl)cyclopropyl)benzene (1n)

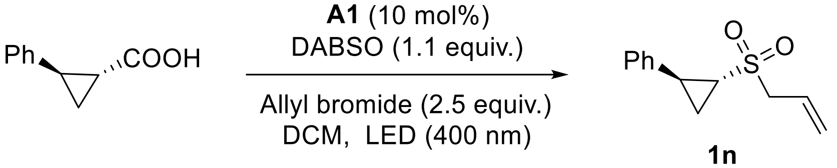

1n

According to **GP1**, the reaction was carried out with (1*R*,2*R*)-2-phenylcyclopropane-1-carboxylic acid (49 mg, 0.3 mmol), DABSO (79 mg, 0.33 mmol, 1.1 equiv.), acridine catalyst **A1** (9 mg, 0.03 mmol, 10 mol%), allyl bromide (91 mg, 0.75 mmol, 2.5 equiv.) in degassed dichloromethane (6 mL). The test-tube was capped and the reaction mixture was irradiated with LED light (λ = 400 nm) while stirring at room temperature for 12 h. The reaction mixture was then quenched with a saturated solution of potassium hydrogen sulfate (2 mL) and extracted with ethyl acetate (3 × 10 mL). The organic layer was combined, dried over anhydrous sodium sulfate, concentrated under reduced pressure, and the remaining material was purified by flash chromatography on silica gel (hexane/ ethyl acetate 7 : 3 v/v) to give the sulfone product **1n** (47 mg, 70%) as a yellow liquid.

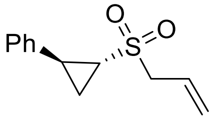

^{1}H NMR (500 MHz, $CDCl_3$): 7.40–7.22 (3 H, m), 7.20–7.06 (2 H, m), 5.98 (1 H, ddt, *J* = 17.4, 10.2, 7.4 Hz), 5.53–5.36 (2 H, m), 3.83 (2 H, d, *J* = 7.4 Hz), 2.86–2.58 (2 H, m), 1.84 (1 H, dt, *J* = 10.5, 5.6 Hz), 1.49 (1 H, dt, *J* = 8.3, 6.1 Hz) ppm. – ^{13}C NMR (125 MHz, $CDCl_3$): 137.4, 128.9, 127.4, 126.6, 124.9, 124.8, 58.8, 37.7, 22.8, 13.1 ppm. – IR: 3053, 2968, 2856, 1738, 1422, 1365, 1264, 1216, 1134, 895 cm^{-1}. – HRMS: calcd for $C_{12}H_{15}O_2S$: 223.0787, found 223.0782 [M+H$^+$].

2-(Allylsulfonyl)-2,3-dihydro-1*H*-indene (1o)

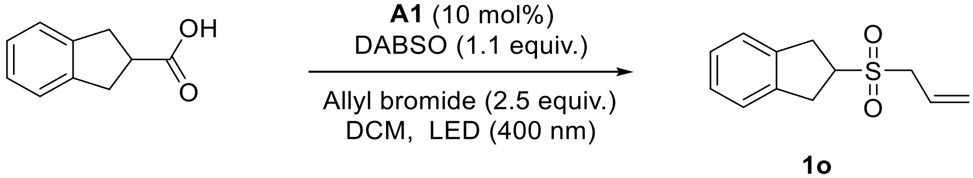

1o

According to **GP1**, the reaction was carried out with 2,3-dihydro-1*H*-indene-2-carboxylic acid (49 mg, 0.3 mmol), DABSO (79 mg, 0.33 mmol, 1.1 equiv.), acridine catalyst **A1** (9 mg, 0.03 mmol, 10 mol%), allyl bromide (91 mg, 0.75 mmol, 2.5 equiv.) in degassed dichloromethane (6 mL). The test-tube was capped, and the reaction mixture was irradiated with LED light (λ = 400 nm) while

stirring at room temperature for 12 h. The reaction mixture was then quenched with a saturated solution of potassium hydrogen sulfate (2 mL) and extracted with ethyl acetate (3 × 10 mL). The organic layer was combined, dried over anhydrous sodium sulfate, concentrated under reduced pressure, and the remaining material was purified by flash chromatography on silica gel (hexane/ ethyl acetate 7 : 3 v/v) to give the sulfone product **22** (41 mg, 61%) as a yellow solid.

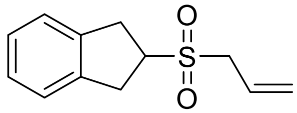

m.p.: 45–47 °C. –^{1}H NMR (300 MHz, $CDCl_3$): 7.30–7.12 (4 H, m), 5.96 (1 H, ddt, *J* = 17.4, 10.2, 7.3 Hz), 5.57–5.32 (2 H, m), 4.00 (1 H, tt, *J* = 9.1, 7.7 Hz), 3.71 (2 H, dt, *J* = 7.4, 1.1 Hz), 3.52 (2 H, dd, *J* = 16.3, 7.7 Hz), 3.31 (2 H, dd, *J* = 16.3, 9.1 Hz) ppm. – ^{13}C NMR (75 MHz, $CDCl_3$): 139.4, 127.4, 124.9, 124.6, 124.5, 59.5, 56.5, 33.3 ppm. – IR: 3072, 3024, 2970, 2916, 2851, 1735, 1643, 1490, 1424, 1394, 1313, 1282, 1243, 1128, 1079, 994, 936, 878, 748 cm^{-1}. – HRMS: calcd for $C_{12}H_{15}O_2S$: 223.0787, found 223.0784 [M+H$^+$].

7-(Allylsulfonyl)pentadecane (1p)

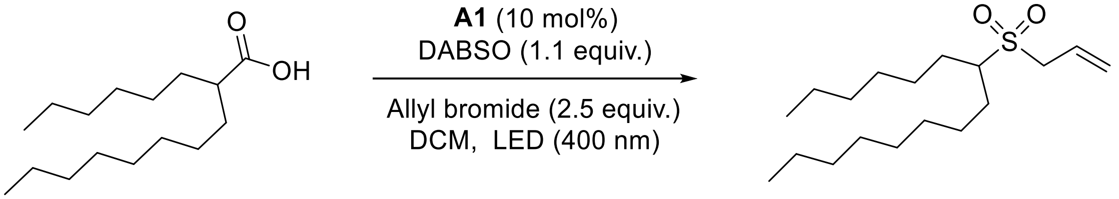

1p

According to **GP1**, the reaction was carried out with 2-hexyldecanoic acid (77 mg, 0.3 mmol), DABSO (79 mg, 0.33 mmol, 1.1 equiv.), acridine catalyst **A1** (9 mg, 0.03 mmol, 10 mol%), allyl bromide (91 mg, 0.75 mmol, 2.5 equiv.) in degassed dichloromethane (6 mL). The test-tube was capped, and the reaction mixture was irradiated with LED light (λ = 400 nm) while stirring at room temperature for 12 h. The reaction mixture was then quenched with a saturated solution of potassium hydrogen sulfate (2 mL) and extracted with ethyl acetate (3 × 10 mL). The organic layer was combined, dried over anhydrous sodium sulfate, concentrated under reduced pressure, and the remaining material was purified by flash chromatography on silica gel (hexane/ ethyl acetate 1 : 4 v/v) to give the sulfone product **1p** (86 mg, 91%) as a yellow liquid.

Gram scale for compound 1p: According to **GP1**, the reaction was carried out with 2-hexyldecanoic acid (1.54 g, 6 mmol), DABSO (1.58 g, 6.6 mmol, 1.1 equiv.), acridine catalyst **A1** (178 mg, 0.6 mmol, 10 mol%), allyl bromide (1.81 g, 15 mmol, 2.5 equiv.) in degassed dichloromethane (80 mL). The test-tube was capped, and the reaction mixture was irradiated with LED light (λ = 400 nm) while stirring at room temperature for 12 h. The reaction mixture was then quenched with a saturated solution of potassium hydrogen sulfate (20 mL) and extracted with

ethyl acetate (3 × 50 mL). The organic layer was combined, dried over anhydrous sodium sulfate, concentrated under reduced pressure, and the remaining material was purified by flash chromatography on silica gel (hexane/ ethyl acetate 1 : 4 v/v) to give the sulfone product **1p** (1.50 g, 79%) as a yellow liquid.

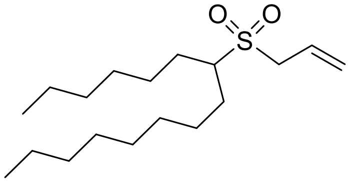

^{1}H NMR (500 MHz, CDCl$_3$): 5.91 (1 H, ddt, *J* = 17.4, 10.2, 7.3 Hz), 5.47–5.37 (2 H, m), 3.67 (2 H, d, *J* = 7.3 Hz), 2.85 (1 H, td, *J* = 7.5, 3.8 Hz), 1.86 (2 H, ddt, *J* = 15.4, 10.6, 5.1 Hz), 1.71–1.59 (2 H, m), 1.42 (4 H, m), 1.27 (16 H, m), 0.86 (6 H, m) ppm. – ^{13}C NMR (125 MHz, CDCl$_3$): 125.3, 124.2, 60.9, 55.8, 31.9, 31.6, 29.7, 29.33, 29.25, 27.7, 26.83, 26.80, 22.7, 22.6, 14.15, 14.08 ppm. – IR: 2959, 2925, 2874, 2855, 1738, 1642, 1466, 1377, 1307, 1293, 1126, 1083, 909, 733 cm^{-1}. – HRMS: calcd for $C_{18}H_{37}O_2S$: 317.2509, found 317.2509 [M+H$^+$].

(1-(Allylsulfonyl)cyclopropyl)benzene (1q)

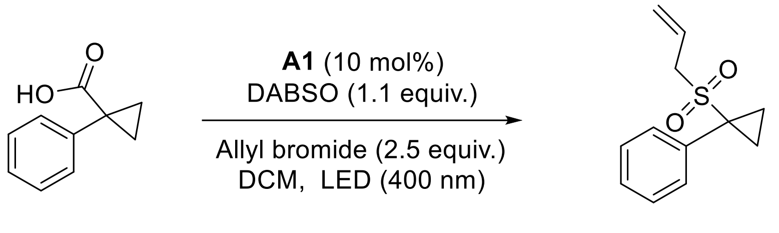

1q

According to **GP1**, the reaction was carried out with 1-phenylcyclopropane-1-carboxylic acid (49 mg, 0.3 mmol), DABSO (79 mg, 0.33 mmol, 1.1 equiv.), acridine catalyst **A1** (9 mg, 0.03 mmol, 10 mol%), allyl bromide (91 mg, 0.75 mmol, 2.5 equiv.) in degassed dichloromethane (6 mL). The test-tube was capped, and the reaction mixture was irradiated with LED light (λ = 400 nm) while stirring at room temperature for 12 h. The reaction mixture was then quenched with a saturated solution of potassium hydrogen sulfate (2 mL) and extracted with ethyl acetate (3 × 10 mL). The organic layer was combined, dried over anhydrous sodium sulfate, concentrated under reduced pressure, and the remaining material was purified by flash chromatography on silica gel (hexane/ ethyl acetate 7 : 3 v/v) to give the sulfone product **1q** (50 mg, 75%) as a yellow liquid.

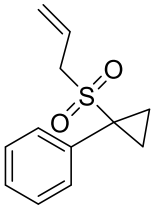

^{1}H NMR (300 MHz, CDCl$_3$): 7.61–7.54 (2H, m), 7.42–7.33 (3 H, m), 5.83 (1 H, ddt, *J* = 17.3, 10.2, 7.2 Hz), 5.50–5.26 (2 H, m), 3.60 (2 H, dt, *J* = 7.2, 1.2 Hz), 1.87–1.76 (2 H), 1.29–1.22 (2 H, m) ppm. – ^{13}C NMR (75 MHz, CDCl$_3$): 134.3, 132.0, 129.2, 128.8, 124.7, 124.2, 55.1, 44.7, 11.8 ppm. – IR: 3093, 3065, 3024, 2984, 2927, 1640, 1605, 1498, 1449,

1420, 1307, 1217, 1130, 1092, 932, 808, 701 cm^{-1}. – HRMS: calcd for $C_{12}H_{14}NaO_2S$: 245.0607, found 245.0608 [M+Na$^+$].

2-(Allylsulfonyl)-2-methylbutane (1r)

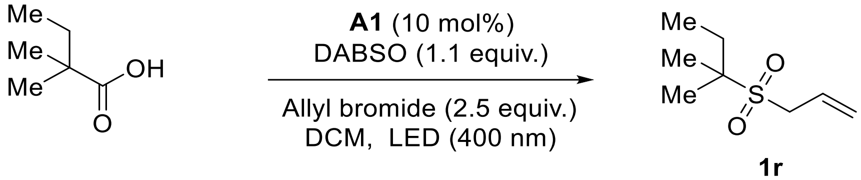

1r

According to **GP1**, the reaction was carried out with 2,2-dimethylbutanoic acid (35 mg, 0.3 mmol), DABSO (79 mg, 0.33 mmol, 1.1 equiv.), acridine catalyst **A1** (9 mg, 0.03 mmol, 10 mol%), allyl bromide (91 mg, 0.75 mmol, 2.5 equiv.) in degassed dichloromethane (6 mL). The test-tube was capped, and the reaction mixture was irradiated with LED light (λ = 400 nm) while stirring at room temperature for 12 h. The reaction mixture was then quenched with a saturated solution of potassium hydrogen sulfate (2 mL) and extracted with ethyl acetate (3 × 10 mL). The organic layer was combined, dried over anhydrous sodium sulfate, concentrated under reduced pressure, and the remaining material was purified by flash chromatography on silica gel (hexane/ ethyl acetate 7 : 3 v/v) to give the sulfone product **1r** (37 mg, 70%) as a colorless oil.

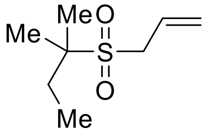

^{1}H NMR (300 MHz, $CDCl_3$): 5.94 (1 H, ddt, *J* = 17.3, 10.2, 7.2 Hz), 5.51–5.36 (2 H, m), 3.68 (2 H, dt, *J* = 7.2, 1.2 Hz), 1.82 (2 H, q, *J* = 7.5 Hz), 1.34 (6 H, s), 0.99 (3 H, t, *J* = 7.6 Hz) ppm. – ^{13}C NMR (75 MHz, $CDCl_3$): 124.5, 124.3, 63.5, 52.0, 28.2, 20.2, 8.2 ppm. – IR: 2975, 2941, 2890, 1645, 1470, 1420, 1287, 1169, 1110, 1014, 935, 772 cm^{-1}. – HRMS: calcd for $C_8H_{16}NaO_2S$: 199.0763, found 199.0761 [M+Na$^+$].

1-(Allylsulfonyl)adamantane (1s)

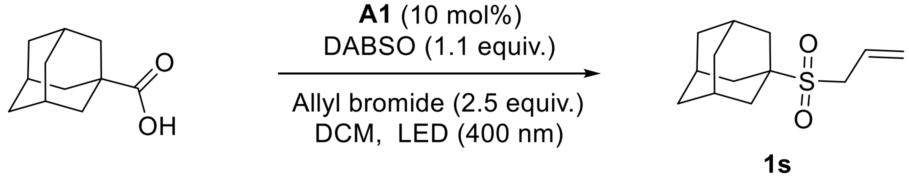

1s

According to **GP1**, the reaction was carried out with adamantane-1-carboxylic acid (54 mg, 0.3 mmol), DABSO (79 mg, 0.33 mmol, 1.1 equiv.), acridine catalyst **A1** (9 mg, 0.03 mmol, 10 mol%), allyl bromide (91 mg, 0.75 mmol, 2.5 equiv.) in degassed dichloromethane (6 mL). The test-tube was capped, and the reaction mixture was irradiated with LED light (λ = 400 nm) while stirring

at room temperature for 12 h. The reaction mixture was then quenched with a saturated solution of potassium hydrogen sulfate (2 mL) and extracted with ethyl acetate (3 × 10 mL). The organic layer was combined, dried over anhydrous sodium sulfate, concentrated under reduced pressure, and the remaining material was purified by flash chromatography on silica gel (hexane/ ethyl acetate 7 : 3 v/v) to give the sulfone product **1s** (62 mg, 86%) as a yellow solid.

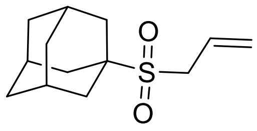

m.p.: 60–62°C. – ^{1}H NMR (500 MHz, $CDCl_3$): 5.92 (1 H, ddt, *J* = 17.2, 10.1, 7.2 Hz), 5.51–5.37 (2 H, m), 3.63 (2 H, d, *J* = 7.2 Hz), 2.21–2.12 (3 H, m), 2.04 (6 H, m), 1.80–1.63 (6 H, m) ppm. – ^{13}C NMR (125 MHz, $CDCl_3$): 124.4, 124.3, 61.5, 50.7, 35.9, 35.1, 28.3 ppm. – IR: 2938, 2911, 2854, 1640, 1455, 1397, 1317, 1301, 1258, 1132, 1102, 1045, 938, 870, 833, 777, 703 cm^{-1}. – HRMS: calcd for $C_{13}H_{21}O_2S$: 241.1257, found 241.1263 [M+H$^+$].

4-(Allylsulfonyl)-4-methyltetrahydro-2*H*-pyran (1t)

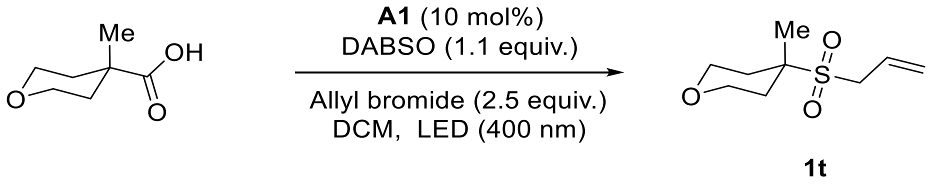

According to **GP1**, the reaction was carried out with 4-methyltetrahydro-2*H*-pyran-4-carboxylic acid (43 mg, 0.3 mmol), DABSO (79 mg, 0.33 mmol, 1.1 equiv.), acridine catalyst **A1** (9 mg, 0.03 mmol, 10 mol%), allyl bromide (91 mg, 0.75 mmol, 2.5 equiv.) in degassed dichloromethane (6 mL). The test-tube was capped, and the reaction mixture was irradiated with LED light (λ = 400 nm) while stirring at room temperature for 12 h. The reaction mixture was then quenched with a saturated solution of potassium hydrogen sulfate (2 mL) and extracted with ethyl acetate (3 × 10 mL). The organic layer was combined, dried over anhydrous sodium sulfate, concentrated under reduced pressure, and the remaining material was purified by flash chromatography on silica gel (hexane/ ethyl acetate 3 : 2 v/v) to give the sulfone product **1t** (45 mg, 74%) as a glassy solid.

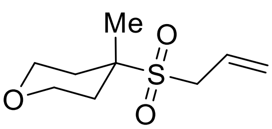

^{1}H NMR (300 MHz, $CDCl_3$): 5.93 (1 H, ddt, *J* = 17.3, 10.2, 7.2 Hz), 5.51–5.38 (2 H, m), 4.02–3.89 (2 H, m), 3.69 (2 H, dt, *J* = 7.2, 1.2 Hz), 3.53 (2 H, td, *J* = 12.0, 2.2 Hz), 2.35–2.19 (2 H, m), 1.62–1.51 (5 H, m) ppm. – ^{13}C NMR (75 MHz, $CDCl_3$): 124.7, 124.1, 63.1, 60.9, 51.5, 30.0, 17.3 ppm. – IR: 2960, 2942, 2859, 1641, 1458, 1304, 1284, 1171, 1130, 1100, 1038, 1018, 936, 848, 690 cm^{-1}. – HRMS: calcd for $C_9H_{17}O_3S$: 205.0893, found 205.0883 [M+H$^+$].

tert-Butyl (S)-4-(allylsulfonyl)-2-((tert-butoxycarbonyl)amino)butanoate (1u)

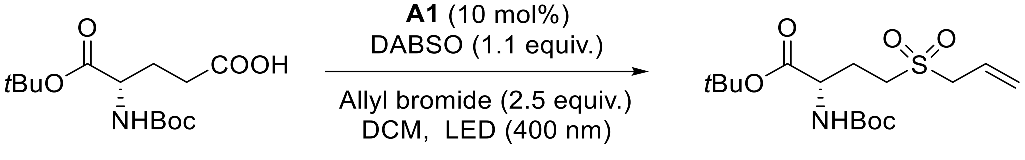

According to **GP1**, the reaction was carried out with (*S*)-5-(*tert*-butoxy)-4-((*tert*-butoxycarbonyl)amino)-5-oxopentanoic acid (91 mg, 0.3 mmol), DABSO (79 mg, 0.33 mmol, 1.1 equiv.), acridine catalyst **A1** (9 mg, 0.03 mmol, 10 mol%), allyl bromide (91 mg, 0.75 mmol, 2.5 equiv.) in degassed dichloromethane (6 mL). The test-tube was capped and the reaction mixture was irradiated with LED light (λ = 400 nm) while stirring at room temperature for 12 h. The reaction mixture was then quenched with a saturated solution of potassium hydrogen sulfate (2 mL) and extracted with ethyl acetate (3 × 10 mL). The organic layer was combined, dried over anhydrous sodium sulfate, concentrated under reduced pressure, and the remaining material was purified by flash chromatography on silica gel (hexane/ ethyl acetate 7 : 3 v/v) to give the sulfone product **1u** (99 mg, 91%) as a white solid.

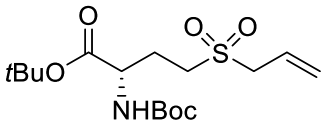

$[\alpha]_D^{23}$ = –19 (*c* 0.58, chloroform). – m.p.: 47–49 °C. – ^{1}H NMR (500 MHz, $CDCl_3$): 5.90 (1 H, ddt, *J* = 17.5, 10.2, 7.4 Hz), 5.51–5.37 (2 H, m), 5.20 (1 H, d, *J* = 7.0 Hz), 4.22 (1 H, d, *J* = 6.2 Hz), 3.70 (2 H, d, *J* = 7.5 Hz), 3.08 (1 H, td, *J* = 12.9, 12.3, 4.8 Hz), 2.94 (1 H, ddd, *J* = 13.8, 11.8, 4.6 Hz), 2.40–2.28 (1 H, m), 2.07 (1 H, tdd, *J* = 12.6, 7.8, 4.5 Hz), 1.43 (18 H, d, *J* = 16.6 Hz) ppm. – ^{13}C NMR (125 MHz, $CDCl_3$): 170.3, 155.5, 125.1, 124.9, 83.1, 80.3, 57.9, 52.7, 47.7, 28.4, 28.0, 25.5 ppm. – IR: 3359, 2977, 2931, 1713, 1515, 1454, 1392, 1367, 1250, 1153, 1053 cm^{-1}. – HRMS: calcd for $C_{16}H_{29}NO_6SNa$: 386.1608, found 386.1617 [M+Na$^+$].

Go back to table of contents

15-(Allylsulfonyl)pentadecane-1,7,8-triyl triacetate (1v)

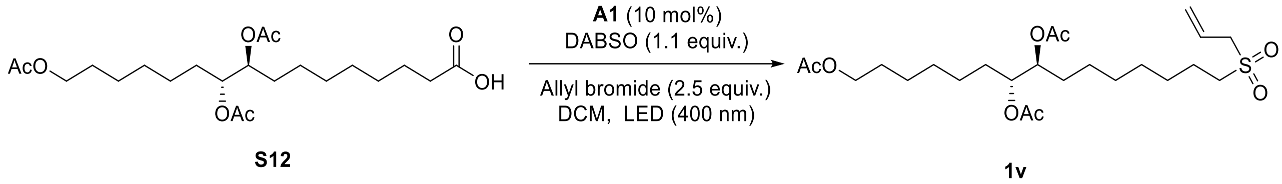

According to **GP1**, the reaction was carried out with acid **S12** (65 mg, 0.15 mmol), DABSO (40 mg, 0.165 mmol, 1.1 equiv.), acridine catalyst **A1** (5 mg, 0.015 mmol, 10 mol%), allyl bromide (45 mg, 0.375 mmol, 2.5 equiv.) in degassed dichloromethane (6 mL). The test-tube was capped, and the reaction mixture was irradiated with LED light (λ = 400 nm) while stirring at room temperature for 12 h. The reaction mixture was then quenched with a saturated solution of potassium hydrogen sulfate (2 mL) and extracted with ethyl acetate (3 × 10 mL). The organic layer was combined, dried over anhydrous sodium sulfate, concentrated under reduced pressure, and the remaining material was purified by flash chromatography on silica gel (hexane/ ethyl acetate 1 : 1 v/v) to give the sulfone product **1v** (60 mg, 82%) as a colorless oil.

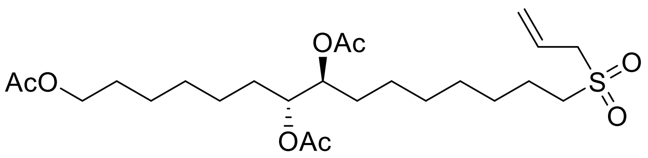

^{1}H NMR (500 MHz, $CDCl_3$): 5.90 (1 H, ddt, *J* = 17.3, 10.1, 7.3 Hz), 5.47–5.38 (2 H, m), 4.94 (2 H, tt, *J* = 5.2, 2.8 Hz), 3.99 (2 H, t, *J* = 6.7 Hz), 3.66 (2 H, d, *J* = 7.4 Hz), 2.94–2.83 (2 H, m), 2.04 (6 H, s), 2.00 (3 H, s), 1.76 (2 H, ddd, *J* = 11.8, 9.9, 6.6 Hz), 1.60–1.52 (2 H, m), 1.47–1.41 (4 H, m), 1.41–1.33 (2 H, m), 1.31–1.17 (12 H, m) ppm. – ^{13}C NMR (125 MHz, $CDCl_3$): 171.2, 170.6, 125.3, 124.5, 73.7, 64.5, 57.7, 51.2, 30.7, 29.0, 29.0, 28.9, 28.5, 28.3, 25.8, 25.12, 25.08, 21.7, 21.04, 20.99 ppm. – IR: 2961, 2933, 2858, 2851, 1733, 1635, 1372, 1312, 1229, 1130, 1025, 950, 850 cm^{-1}. – HRMS: calcd for $C_{24}H_{42}NaO_8S$: 513.2493, found 513.2506 [M+H$^+$].

Di-*tert*-butyl ((2*S*,3*S*,4*R*)-2-(4-(allylsulfonyl)butyl)tetrahydrothiophene-3,4-diyl)dicarbamate (1w)

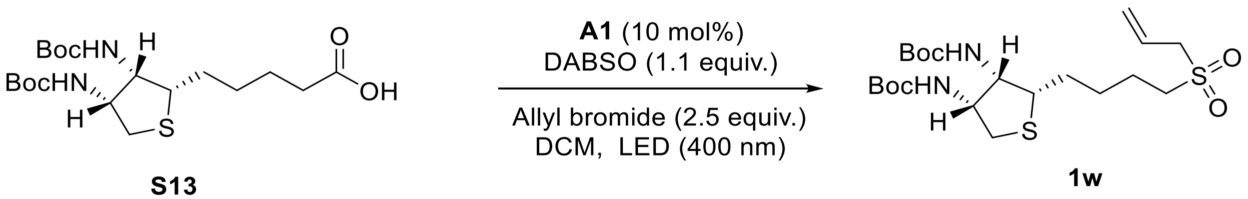

According to **GP1**, the reaction was carried out with acid **S13** (63 mg, 0.15 mmol), DABSO (40 mg, 0.165 mmol, 1.1 equiv.), acridine catalyst **A1** (5 mg, 0.015 mmol, 10 mol%), allyl bromide (45 mg, 0.375 mmol, 2.5 equiv.) in degassed dichloromethane (6 mL). The test-tube was capped, and the reaction mixture was irradiated with LED light (λ = 400 nm) while stirring at room temperature

for 12 h. The reaction mixture was then quenched with a saturated solution of potassium hydrogen sulfate (2 mL) and extracted with ethyl acetate (3 × 10 mL). The organic layer was combined, dried over anhydrous sodium sulfate, concentrated under reduced pressure, and the remaining material was purified by flash chromatography on silica gel (hexane/ ethyl acetate 1 : 1 v/v) to give the sulfone product **1w** (53 mg, 74%) as a colorless oil.

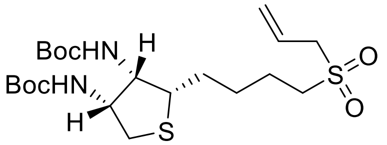

$[\alpha]_D^{23}$ = –51.4 (c 0.36, $CHCl_3$). – ^{1}H NMR (300 MHz, $CDCl_3$): 5.90 (1 H, ddt, *J* = 17.3, 10.1, 7.3 Hz), 5.52–5.32 (2 H, m), 4.94 (1 H, d, *J* = 8.0 Hz), 4.77 (1 H, d, *J* = 10.1 Hz), 4.40–4.04 (2 H, m), 3.67 (2 H, d, *J* = 7.3 Hz), 3.47 (1 H, q, *J* = 6.4, 5.9 Hz), 3.22–3.09 (1 H, m), 2.97–2.84 (2 H, m), 2.47 (1 H, t, *J* = 10.4 Hz), 1.95–1.57 (4 H, m), 1.42 (20 H, m) ppm. – ^{13}C NMR (75 MHz, $CDCl_3$): 156.2, 155.2, 125.2, 124.6, 80.4, 80.0, 57.8, 56.6, 56.4, 50.9, 48.1, 31.8, 30.5, 28.39, 28.35, 27.3, 21.7 ppm. – IR: 3360, 2975, 2933, 2875, 1713, 1647, 1507, 1456, 1366, 1391, 1248, 1165, 1130, 1045, 937, 861 cm^{-1}. – HRMS: calcd for $C_{21}H_{38}N_2NaO_6S_2$: 501.2063, found 501.2071 [M+H$^+$].

(2*R*,4a*S*,6a*S*,6b*R*,10*S*,12a*S*,14b*R*)-2-(Allylsulfonyl)-10-hydroxy-2,4a,6a,6b,9,9,12a-heptamethyl-1,3,4,4a,5,6,6a,6b,7,8,8a,9,10,11,12,12a,12b,14b-octadecahydropicen-13(2*H*)-one (1x)

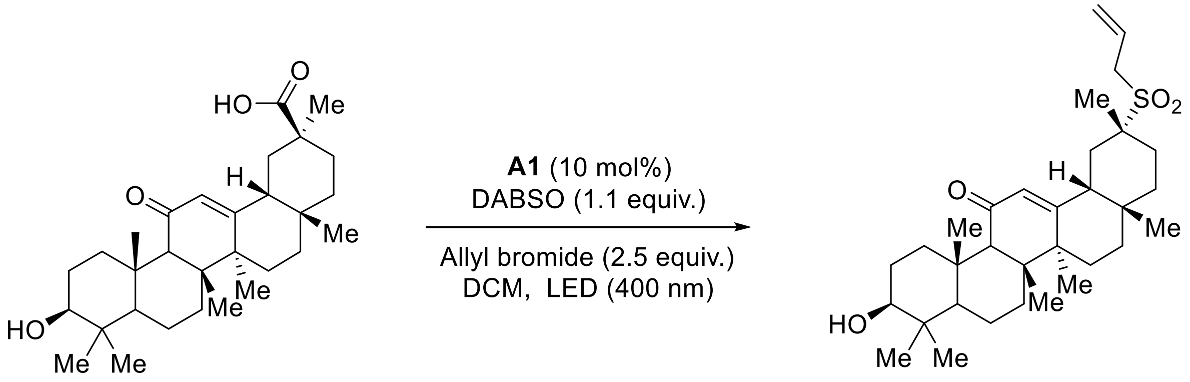

1x

According to **GP1**, the reaction was carried out with 18-β-glycyrrhetinic acid (71 mg, 0.15 mmol), DABSO (40 mg, 0.165 mmol, 1.1 equiv.), acridine catalyst **A1** (5 mg, 0.015 mmol, 10 mol%), allyl bromide (45 mg, 0.375 mmol, 2.5 equiv.) in degassed dichloromethane (6 mL). The test-tube was capped, and the reaction mixture was irradiated with LED light (λ = 400 nm) while stirring at room temperature for 12 h. The reaction mixture was then quenched with a saturated solution of potassium hydrogen sulfate (2 mL) and extracted with ethyl acetate (3 × 10 mL). The organic layer was combined, dried over anhydrous sodium sulfate, concentrated under reduced pressure, and the remaining material was purified by flash chromatography on silica gel (hexane/ ethyl acetate 2 : 3 v/v) to give the sulfone product **1x** (48 mg, 60%) as a colorless solid.

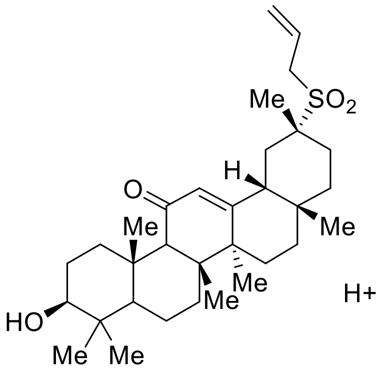

m.p.: > 250 °C. – $[\alpha]_D^{23}$ = + 48.9 (c 0.45, $CHCl_3$). – ^1H NMR (500 MHz, $CDCl_3$): 5.92 (1 H, ddt, *J* = 17.4, 10.2, 7.3 Hz), 5.59 (1 H, s), 5.52–5.37 (2 H, m), 3.75–3.59 (2 H, m), 3.21 (1 H, dd, *J* = 11.1, 5.3 Hz), 2.74 (1 H, dt, *J* = 13.5, 3.6 Hz), 2.47 (1 H, t, *J* = 13.4 Hz), 2.32 (1 H, s), 2.22–1.99 (3 H, m), 1.81 (1 H, td, *J* = 13.9, 4.7 Hz), 1.73–1.33 (17 H, m), 1.25–1.17 (1 H, m), 1.11 (6 H, s), 1.07–0.85 (8 H, m), 0.79 (3H, s), 0.69 (1H, dd, *J* = 11.8, 1.9 Hz) ppm. – ^{13}C NMR (125 MHz, $CDCl_3$): 200.0, 167.5, 129.1, 124.6, 124.3, 78.7, 64.0, 62.0, 55.0, 51.8, 46.4, 45.6, 43.4, 39.2, 39.2, 37.2, 35.0, 34.5, 32.8, 32.4, 28.2, 28.2, 27.4, 26.3, 26.3, 24.7, 23.7, 18.8, 17.6, 17.3, 16.5, 15.7 ppm. – IR: 3521, 2985, 2927, 2867, 1654, 1624, 1471, 1387, 1306, 1285, 1206, 1134, 1090, 1044, 994, 934, 880, 734 cm^{-1}. – HRMS: calcd for $C_{32}H_{51}O_4S$: 531.3503, found 531.3501 [M+H$^+$].

(3*R*,7*R*,8*R*,9*S*,10*S*,13*R*,14*S*,17*R*)-17-((*R*)-4-(Allylsulfonyl)butan-2-yl)-10,13-dimethylhexadecahydro-1*H*-cyclopenta[*a*]phenanthrene-3,7-diol (1y)

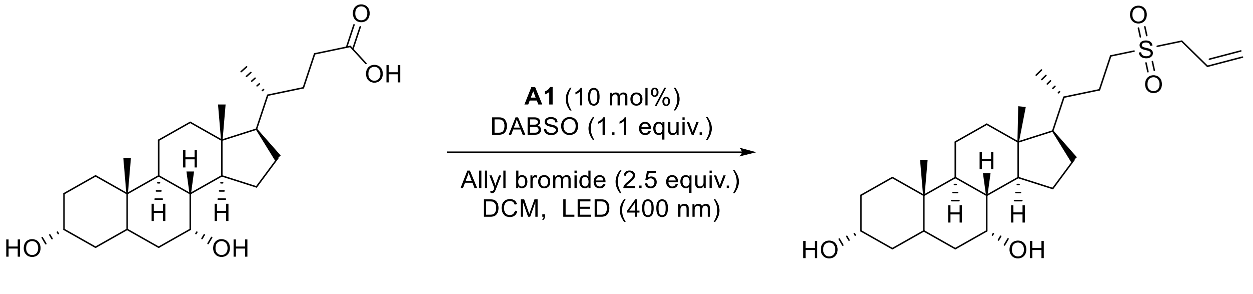

1y

According to **GP1**, the reaction was carried out with chenodeoxycholic acid (59 mg, 0.15 mmol), DABSO (40 mg, 0.165 mmol, 1.1 equiv.), acridine catalyst **A1** (5 mg, 0.015 mmol, 10 mol%), allyl bromide (45 mg, 0.375 mmol, 2.5 equiv.) in degassed dichloromethane (6 mL). The test-tube was capped, and the reaction mixture was irradiated with LED light (λ = 400 nm) while stirring at room temperature for 12 h. The reaction mixture was then quenched with a saturated solution of potassium hydrogen sulfate (2 mL) and extracted with ethyl acetate (3 × 10 mL). The organic layer was combined, dried over anhydrous sodium sulfate, concentrated under reduced pressure, and the remaining material was purified by flash chromatography on silica gel (hexane/ ethyl acetate 1 : 9 v/v) to give the sulfone product **1y** (45 mg, 66%) as a colorless solid.

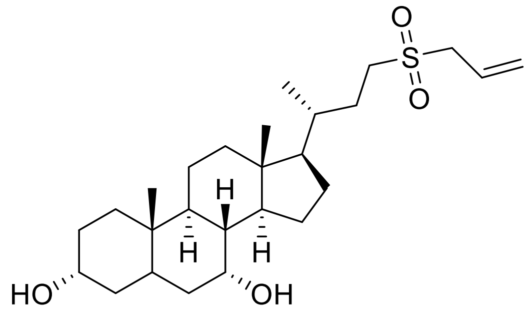

m.p.: 45–47 °C. –$[\alpha]_D^{23}$ = + 16.4 (c 0.11, $CHCl_3$). – ^1H NMR (300 MHz, $CDCl_3$): 5.90 (1 H, ddt, *J* = 17.4, 10.2, 7.3 Hz), 5.50–5.34 (2 H, m), 3.79 (1 H, t, *J* = 2.9 Hz), 3.67 (2 H, d, *J* = 7.4 Hz), 3.40 (1 H, tt, *J* = 11.1, 4.4 Hz,), 2.96 (1 H, ddd, *J* = 13.2, 12.5, 4.2 Hz), 2.81 (1 H, ddd, *J* = 13.6, 10.9, 4.8 Hz), 2.54–2.05 (3 H, m), 1.98–1.02 (21 H, m), 0.99–0.76 (8 H, m), 0.62 (3 H, s) ppm. – ^{13}C NMR (75 MHz, $CDCl_3$): 125.3, 124.5, 71.9, 68.3, 57.5, 55.5, 50.4, 48.7, 42.7, 41.5, 39.7, 39.6, 39.4, 35.4, 35.1, 35.0, 34.8, 32.8, 30.6, 28.2, 27.6, 23.6, 22.8, 20.6, 18.4, 11.8 ppm. – IR: 3391, 2972, 2925, 2863, 1738, 1639, 1475, 1309, 1289, 1128, 1078, 1001, 979, 931, 733 cm^{-1}. – HRMS: calcd for $C_{26}H_{44}NaO_4S$: 475.2853, found 475.2857 [M+Na^+].

((1*S*,2*S*,4a*R*,4b*R*,7*S*,9a*S*,10*S*,10a*R*)-10-(Allylsulfonyl)-1-methyl-8-methylene-13-oxo-1,2,5,6,8,9,10,10a-octahydro-4a,1-(epoxymethano)-7,9a-methanobenzo[*a*]azulene-2,7(4b*H*)-diyl diacetate (1z)

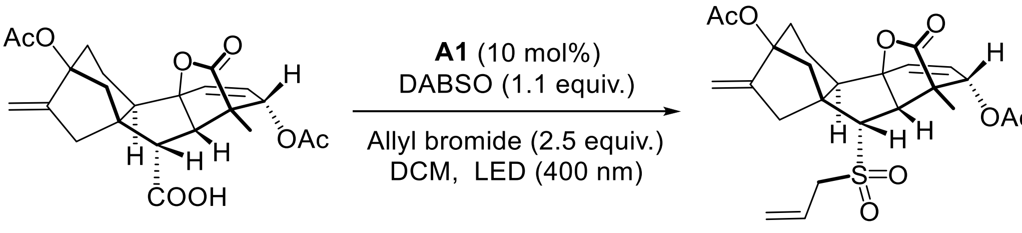

S14 **1z**

According to **GP1**, the reaction was carried out with acid **S14** (65 mg, 0.15 mmol), DABSO (40 mg, 0.165 mmol, 1.1 equiv.), acridine catalyst **A1** (5 mg, 0.015 mmol, 10 mol%), allyl bromide (46 mg, 0.375 mmol, 2.5 equiv.) in degassed dichloromethane (3 mL). The test-tube was capped and the reaction mixture was irradiated with LED light (λ = 400 nm) while stirring at room temperature for 12 h. The reaction mixture was then quenched with a saturated solution of potassium hydrogen sulfate (2 mL) and extracted with ethyl acetate (3 × 10 mL). The organic layer was combined, dried over anhydrous sodium sulfate, concentrated under reduced pressure, and the remaining material was purified by flash chromatography on silica gel (hexane/ ethyl acetate 7 : 3 v/v) to give the sulfone product **1z** (68 mg, 93%) as a white solid.

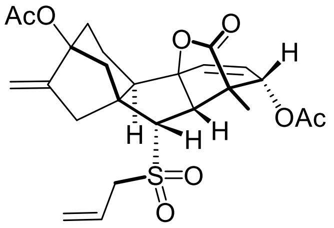

$[\alpha]_D^{23}$ = +86 (*c* 0.5, chloroform). – m.p.: 76–78 °C. – ^{1}H NMR (500 MHz, $CDCl_3$): 6.45 (1 H, dd, *J* = 9.2, 0.8 Hz), 5.97–5.80 (2 H, m), 5.62–5.50 (2H, m), 5.42 (1 H, dd, *J* = 3.8, 0.7 Hz), 5.11 (1 H, dt, *J* = 2.1, 1.1 Hz), 5.01 (1 H, dd, *J* = 3.3, 1.5 Hz), 3.96–3.77 (2 H, m), 3.49 (1 H, d, *J* = 7.9 Hz), 3.40 (1 H, d, *J* = 7.9 Hz), 3.15 (1 H, dt, *J* = 15.6, 3.1 Hz), 2.86–2.76 (1 H, m), 2.65 (1 H, dd, *J* = 10.3, 2.7 Hz), 2.23–2.04 (6 H, m), 2.04–1.89 (5 H, m), 1.74 (1 H, ddt, *J* = 11.8, 7.0, 2.6 Hz), 1.29 (3 H, s) ppm. – ^{13}C NMR (125 MHz, $CDCl_3$): 176.9, 170.1, 169.7, 148.7, 133.6, 129.8, 126.2, 124.6, 106.8, 89.8, 83.7, 71.4, 63.3, 59.7, 52.2, 52.2, 52.0, 47.6, 44.3, 42.5, 36.5, 22.2, 20.9, 17.8, 15.0 ppm. – IR: 2932, 2890, 1777, 1732, 1452, 1369, 1312, 1221, 1161, 1131, 1088, 1021, 951 cm^{-1}. – HRMS: calcd for $C_{25}H_{30}O_8S$: 491.1734, found 491.1732 [M+H$^+$].

1-((2-Bromoallyl)sulfonyl)pentadecane (2a)

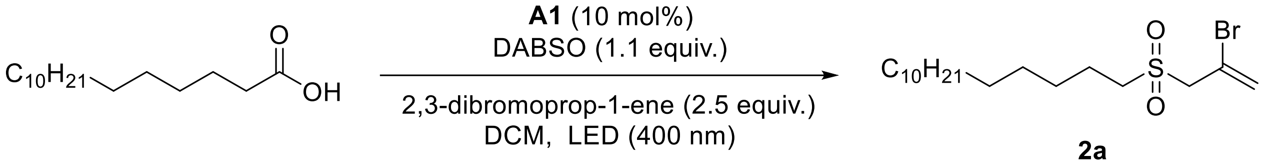

According to **GP1**, the reaction was carried out with palmitic acid (77 mg, 0.3 mmol), DABSO (79 mg, 0.33 mmol, 1.1 equiv.), acridine catalyst **A1** (9 mg, 0.03 mmol, 10 mol%), 2,3-dibromoprop-1-ene (178 mg, 0.75 mmol, 2.5 equiv.) in degassed dichloromethane (6 mL). The test-tube was capped, and the reaction mixture was irradiated with LED light (λ = 400 nm) while stirring at room temperature for 12 h. The reaction mixture was then quenched with a saturated solution of potassium hydrogen sulfate (2 mL) and extracted with ethyl acetate (3 × 10 mL). The organic layer was combined, dried over anhydrous sodium sulfate, concentrated under reduced pressure, and the remaining material was purified by flash chromatography on silica gel (hexane/ ethyl acetate 1 : 4 v/v) to give the sulfone product **2a** (110 mg, 93%) as a slightly yellow solid.

Gram scale for compound 2a: According to **GP1**, the reaction was carried out with palmitic acid (1.03 g, 4 mmol), DABSO (1.06 mg, 4.4 mmol, 1.1 equiv.), acridine catalyst **A1** (119 mg, 0.4 mmol, 10 mol%), 2,3-dibromoprop-1-ene (2.00 g, 10 mmol, 2.5 equiv.) in degassed dichloromethane (80 mL). The test-tube was capped, and the reaction mixture was irradiated with LED light (λ = 400 nm) while stirring at room temperature for 12 h. The reaction mixture was then quenched with a saturated solution of potassium hydrogen sulfate (20 mL) and extracted with ethyl acetate (3 × 50 mL). The organic layer was combined, dried over anhydrous sodium sulfate, concentrated under

reduced pressure, and the remaining material was purified by flash chromatography on silica gel (hexane/ ethyl acetate 1 : 4 v/v) to give the sulfone product **2a** (1.45 g, 91%) as a slightly yellow solid.

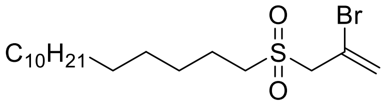

m.p.: 43–45 °C. – ^{1}H NMR (500 MHz, $CDCl_3$): 6.12 (1 H, d, *J* = 2.2 Hz), 5.92 (1 H, d, *J* = 2.3 Hz), 4.04 (2 H, s), 3.17–3.06 (2 H, m), 1.87 (2 H, ddd, *J* = 11.8, 9.9, 6.5 Hz), 1.45 (2 H, quint., *J* = 7.3 Hz), 1.36–1.23 (22 H, m), 0.88 (3 H, t, *J* = 6.8 Hz) ppm. – ^{13}C NMR (125 MHz, $CDCl_3$): 127.0, 117.1, 63.5, 53.1, 32.1, 29.8, 29.8, 29.7, 29.6, 29.5, 29.4, 29.2, 28.5, 22.8, 22.1, 14.3 ppm. – IR: 3055, 2993, 2924, 2853, 1771, 1623, 1371, 1264, 1225, 1127, 910, 737, 705 cm^{-1}. – HRMS: calcd for $C_{18}H_{35}BrNaO_2S$: 417.1433, found 417.1426 [M+Na$^+$].

1-((2-Methylallyl)sulfonyl)pentadecane (2b)

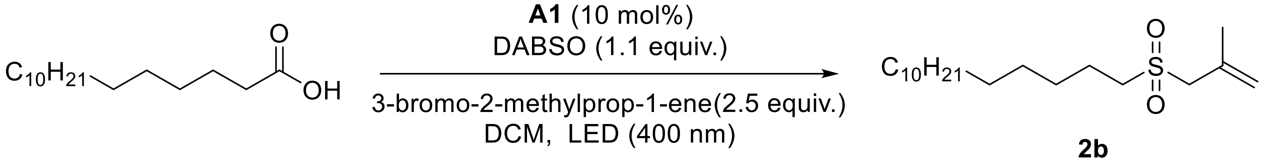

According to **GP1**, the reaction was carried out with palmitic acid (77 mg, 0.3 mmol), DABSO (79 mg, 0.33 mmol, 1.1 equiv.), acridine catalyst **A1** (9 mg, 0.03 mmol, 10 mol%), 3-bromo-2-methylprop-1-ene (101 mg, 0.75 mmol, 2.5 equiv.) in degassed dichloromethane (6 mL). The test-tube was capped, and the reaction mixture was irradiated with LED light (λ = 400 nm) while stirring at room temperature for 12 h. The reaction mixture was then quenched with a saturated solution of potassium hydrogen sulfate (2 mL) and extracted with ethyl acetate (3 × 10 mL). The organic layer was combined, dried over anhydrous sodium sulfate, concentrated under reduced pressure, and the remaining material was purified by flash chromatography on silica gel (hexane/ ethyl acetate 1 : 4 v/v) to give the sulfone product **2b** (94 mg, 95%) as a colorless solid.

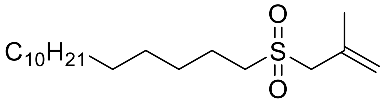

m.p.: 45–47 °C. – ^{1}H NMR (500 MHz, $CDCl_3$): 5.21 (1 H, t, *J* = 1.6 Hz), 5.08 (1 H, s,), 3.65 (2 H, s), 3.03–2.93 (2 H, m), 1.99 (3 H, s), 1.88–1.78 (2 H, m), 1.42 (2 H, quint., *J* = 7.2 Hz), 1.36–1.22 (22 H, s), 0.88 (3 H, t, *J* = 6.9 Hz) ppm. – ^{13}C NMR (125 MHz, $CDCl_3$): 134.5, 120.4, 61.3, 51.5, 32.1, 29.8, 29.8, 29.7, 29.6, 29.0, 29.4, 29.2, 28.6, 22.8, 22.1, 14.3 ppm. – IR: 2997, 2948, 2856, 1775, 1622, 1369, 1261, 1230, 1130, 911, 738, 705 cm^{-1}. – HRMS: calcd for $C_{19}H_{39}O_2S$: 331.2665, found 331.2653 [M+H$^+$].

1-Bromo-2-((cyclohexylsulfonyl)methyl)benzene (2c)

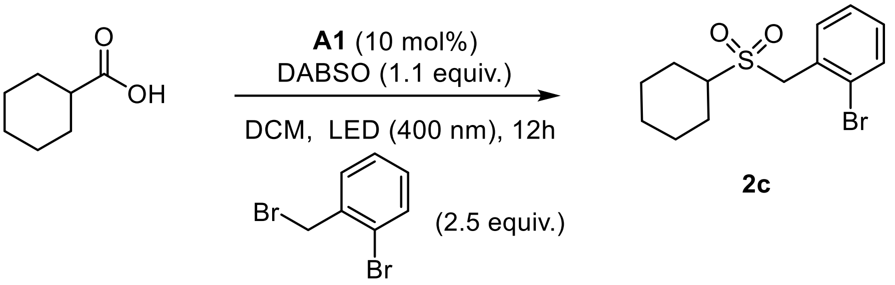

According to **GP2**, the reaction was carried out with cyclohexanecarboxylic acid (38 mg, 0.3 mmol), DABSO (108 mg, 0.45 mmol, 1.5 equiv.), acridine catalyst **A1** (9 mg, 0.03 mmol, 10 mol%), 1-bromo-2-(bromomethyl)benzene (187 mg, 0.75 mmol, 2.5 equiv.) in degassed dichloromethane (3 mL). The test-tube was capped and the reaction mixture was irradiated with LED light (λ = 400 nm) while stirring at room temperature for 12 h. The reaction mixture was then quenched with a saturated solution of potassium hydrogen sulfate (2 mL) and extracted with ethyl acetate (3 × 10 mL). The organic layer was combined, dried over anhydrous sodium sulfate, concentrated under reduced pressure, and the remaining material was purified by flash chromatography on silica gel (hexane/ethyl acetate 3 : 2 v/v) to give the sulfone product **2c** (67 mg, 70%) as a white solid.

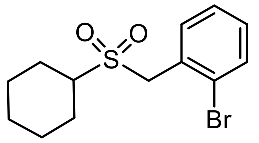

m.p.: 71–73°C. – ^{1}H NMR (500 MHz, CDCl$_3$): 7.61 (2 H, dt, *J* = 7.7, 1.8 Hz), 7.35 (1 H, td, *J* = 7.6, 1.3 Hz), 7.23 (1 H, td, *J* = 7.8, 1.8 Hz), 4.44 (2 H, s), 2.83 (1 H, tt, *J* = 12.2, 3.5 Hz), 2.24–2.15 (2 H, m), 1.91 (2 H, ddd, *J* = 11.1, 5.3, 2.5 Hz), 1.74–1.67 (1 H, m), 1.65–1.50 (2 H, m), 1.33–1.16 (3 H, m) ppm. – ^{13}C NMR (125 MHz, CDCl$_3$): 133.4, 133.3, 130.6, 128.4, 128.1, 125.4, 61.0, 55.7, 25.5, 25.3, 25.2 ppm. – IR: 2932, 2855, 1719, 1588, 1474, 1442, 1409, 1349, 1310, 1270, 1127, 1047, 1026 cm^{-1}. – HRMS: calcd for $C_{13}H_{18}BrO_2S$: 317.0205, found 317.0208 [M+H$^+$].

1-Methyl-4-(((4-phenylbutyl)sulfonyl)methyl)benzene (2d)

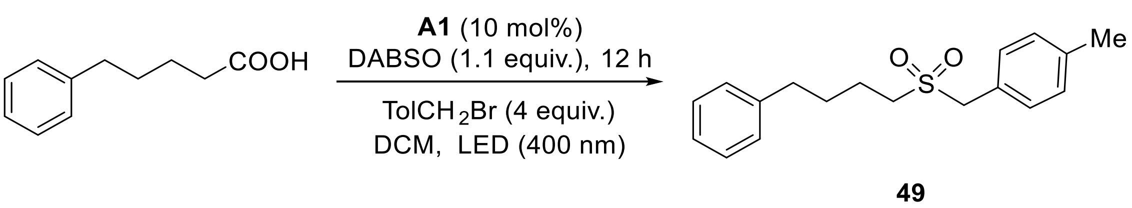

According to **GP2**, the reaction was carried out with 5-phenylpentanoic acid (53 mg, 0.3 mmol), DABSO (79 mg, 0.33 mmol, 1.1 equiv.), acridine catalyst **A1** (9 mg, 0.03 mmol, 10 mol%), 1-(bromomethyl)-4-methylbenzene (220 mg, 1.2 mmol, 4 equiv.) in degassed dichloromethane (3 mL). The test-tube was capped and the reaction mixture was irradiated with LED light (λ = 400

nm) while stirring at room temperature for 12 h. The reaction mixture was then quenched with a saturated solution of potassium hydrogen sulfate (2 mL) and extracted with ethyl acetate (3 × 10 mL). The organic layer was combined, dried over anhydrous sodium sulfate, concentrated under reduced pressure, and the remaining material was purified by flash chromatography on silica gel (hexane/ethyl acetate 4 : 1 v/v) to give the sulfone product **49** (62 mg, 68%) as a yellow solid.

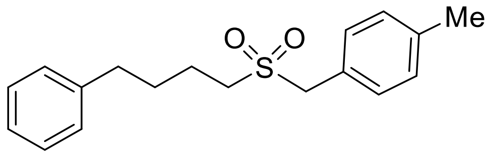

m.p.: 68–70°C. – ^{1}H NMR (500 MHz, $CDCl_3$): 7.36–7.08 (9 H, m), 4.15 (2 H, s), 2.86–2.75 (2 H, m), 2.62 (2 H, t, *J* = 7.5 Hz), 2.37 (3 H, s), 1.83 (2 H, tt, *J* = 8.0, 6.2 Hz), 1.77–1.65 (2 H, m) ppm. – ^{13}C NMR (125 MHz, $CDCl_3$): 141.3, 139.1, 130.4, 130.0, 128.6, 128.5, 126.2, 125.1, 59.3, 50.8, 35.4, 30.2, 21.5, 21.3 ppm. – IR: 2954, 2923, 2858, 1510, 1454, 1378, 1300, 1125, 890 cm^{-1}. – HRMS: calcd for $C_{18}H_{23}O_2S$: 303.1413, found 303.1416 [M+H^{+}].

4-(Methylsulfonyl)-1-phenylbutan-1-one (2e)

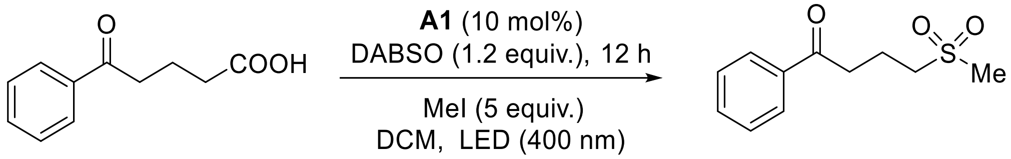

2e

According to **GP2**, the reaction was carried out with 5-oxo-5-phenylpentanoic acid (58 mg, 0.3 mmol), DABSO (86 mg, 0.33 mmol, 1.2 equiv.), acridine catalyst **A1** (9 mg, 0.03 mmol, 10 mol%), iodomethane (213 mg, 1.5 mmol, 5 equiv.) in degassed dichloromethane (3 mL). The test-tube was capped and the reaction mixture was irradiated with LED light (λ = 400 nm) while stirring at room temperature for 12 h. The reaction mixture was then quenched with a saturated solution of potassium hydrogen sulfate (2 mL) and extracted with ethyl acetate (3 × 10 mL). The organic layer was combined, dried over anhydrous sodium sulfate, concentrated under reduced pressure, and the remaining material was purified by flash chromatography on silica gel (hexane/ethyl acetate 1 : 1 v/v) to give the sulfone product **2e** (39 mg, 58%) as a white solid.

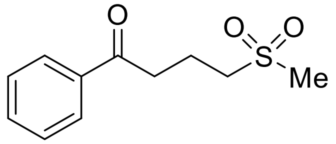

m.p.: 81–83°C. – ^{1}H NMR (500 MHz, $CDCl_3$): 8.03–7.82 (2 H, m), 7.52 (3 H, dt, *J* = 33.9, 7.4 Hz), 3.20 (4 H, dt, *J* = 26.4, 7.1 Hz), 2.95 (3 H, s), 2.46–2.22 (2 H, m) ppm. – ^{13}C NMR (125 MHz, $CDCl_3$): 198.6, 136.5, 133.6, 128.9, 128.1, 53.8, 40.7, 36.3, 17.3 ppm. – IR: 2955, 2924, 2858, 1560, 1458, 1378, 1264, 891 cm^{-1}. – HRMS: calcd for $C_{11}H_{15}O_3S$: 227.0742, found 227.0749 [M+H^{+}].

1,4-Dimethyl-2-((4-methyl-4-(methylsulfonyl)pentyl)oxy)benzene (2f)

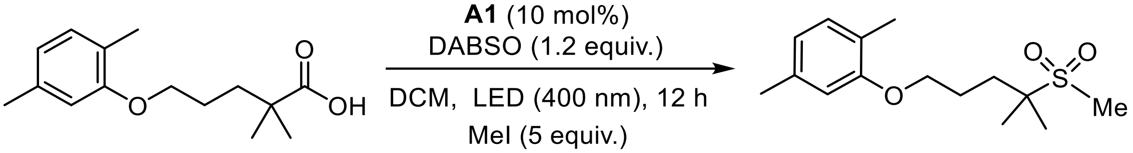

2f

According to **GP2**, the reaction was carried out with gemfibrozil (75 mg, 0.3 mmol), DABSO (86 mg, 0.36 mmol, 1.2 equiv.), acridine catalyst **A1** (9 mg, 0.03 mmol, 10 mol%), MeI (213 mg, 1.5 mmol, 5 equiv.) in degassed dichloromethane (3 mL). The test-tube was capped and the reaction mixture was irradiated with LED light (λ = 400 nm) while stirring at room temperature for 12 h. The reaction mixture was then quenched with a saturated solution of potassium hydrogen sulfate (2 mL) and extracted with ethyl acetate (3 × 10 mL). The organic layer was combined, dried over anhydrous sodium sulfate, concentrated under reduced pressure, and the remaining material was purified by flash chromatography on silica gel (hexane/ethyl acetate 3 : 2 v/v) to give the sulfone product **2f** (44 mg, 52%) as a yellow liquid.

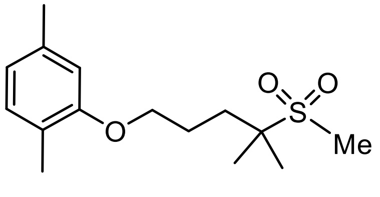

^{1}H NMR (500 MHz, $CDCl_3$): 7.01 (1 H, d, *J* = 7.4 Hz), 6.68 (1 H, dd, *J* = 7.5, 1.5 Hz), 6.62 (1 H, d, *J* = 1.6 Hz), 3.98 (2 H, t, *J* = 5.6 Hz), 2.82 (3 H, s), 2.31 (3 H, s), 2.17 (3 H, ,s), 2.08–1.83 (4 H, m), 1.43 (6 H, s) ppm. – ^{13}C NMR (125 MHz, $CDCl_3$): 156.8, 136.7, 130.5, 123.6, 121.1, 112.1, 67.5, 61.7, 34.6, 32.3, 24.4, 21.5, 20.8, 15.9 ppm. – IR: 3063, 3012, 2996, 2974, 2897, 2786, 1785, 1236, 1189, 1085, 963 cm^{-1}. – HRMS: calcd for $C_{15}H_{25}O_3S$: 285.1519, found 285.1518 [M+H^{+}].

Methyl (*tert*-butoxycarbonyl)(cyclohexylsulfonyl)-D-alaninate (2g)

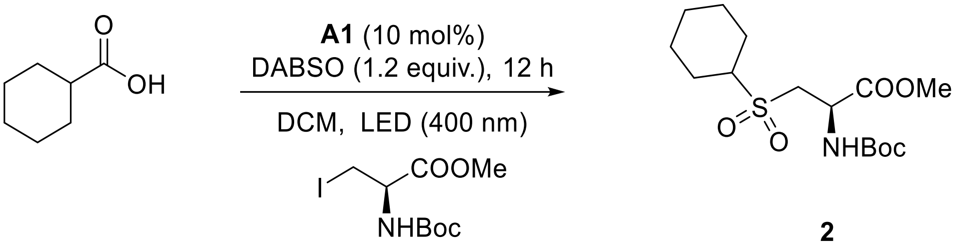

2

According to **GP3**, the reaction was carried out with cyclohexanecarboxylic acid (38 mg, 0.3 mmol, 2 equiv.), DABSO (43 mg, 0.18 mmol, 1.2 equiv.), acridine catalyst **A1** (5 mg, 0.015 mmol, 10 mol%), methyl (*R*)-2-((*tert*-butoxycarbonyl)amino)-3-iodopropanoate (49 mg, 0.15 mmol) in degassed dichloromethane (3 mL). The test-tube was capped and the reaction mixture was irradiated with LED light (λ = 400 nm) while stirring at room temperature for 12 h. The reaction mixture was then quenched with a saturated solution of potassium hydrogen sulfate (2 mL) and

extracted with ethyl acetate (3 × 10 mL). The organic layer was combined, dried over anhydrous sodium sulfate, concentrated under reduced pressure, and the remaining material was purified by flash chromatography on silica gel (hexane/ethyl acetate 4 : 1 v/v) to give the sulfone product **2g** (48 mg, 92 %) as a white solid.

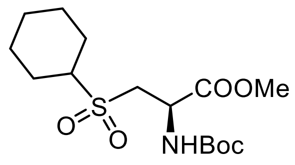

m.p.: 81–83°C. – $[\alpha]_D^{23}$ = + 1.5 (c 0.32, $CHCl_3$). – ^1H NMR (500 MHz, $CDCl_3$): 5.66 (1 H, d, *J* = 7.3 Hz), 4.65 (1 H, ,q, *J* = 5.1 Hz), 3.80 (3 H, s), 3.64–3.51 (2 H, m), 2.86 (1 H, tt, *J* = 12.2, 3.4 Hz), 2.17–2.09 (2 H, m), 1.96–1.87 (2 H, m), 1.78–1.68 (1 H, m), 1.60–1.43 (11 H, m), 1.36–1.11 (3 H, m) ppm. – ^{13}C NMR (125 MHz, $CDCl_3$): 170.0, 155.4, 80.9, 63.1, 53.3, 50.4, 49.8, 28.4, 25.18, 25.15, 25.0, 24.7 ppm. – IR: 3033, 2986, 2932, 2873, 1763, 1560, 1532, 1123, 1098, 987 cm^{-1}. – HRMS: calcd for $C_{15}H_{27}NO_6SNa$: 372.1451, found 372.1447 [M+Na$^+$].

cis-3-((Cyclohexylsulfonyl)methyl)-1,2-oxathiolane 2-oxide (2h)

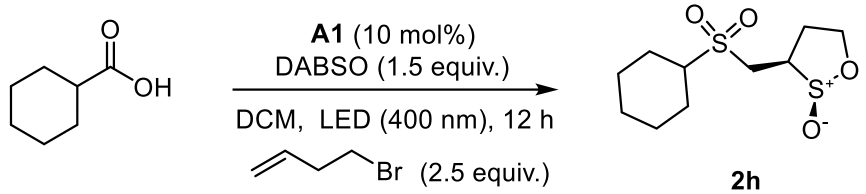

2h

According to **GP2**, the reaction was carried out with cyclohexanecarboxylic acid (38 mg, 0.3 mmol), DABSO (108 mg, 0.45 mmol, 1.5 equiv.), acridine catalyst **A1** (9 mg, 0.03 mmol, 10 mol%), 4-bromobut-1-ene (100 mg, 0.75 mmol, 2.5 equiv.) in degassed DCM (3 mL). The test-tube was capped and the reaction mixture was irradiated with LED light (λ = 400 nm) while stirring at room temperature for 12 h. The reaction mixture was then quenched with a saturated solution of potassium hydrogen sulfate (2 mL) and extracted with ethyl acetate (3 × 10 mL). The organic layer was combined, dried over anhydrous sodium sulfate, concentrated under reduced pressure, and the remaining material was purified by flash chromatography on silica gel (hexane/ethyl acetate 3 : 2 v/v) to give inseparable mixture of sulfone products **2h** (cis/trans = 9/1, 56 mg, 70%) as a white solid.

m.p.: 88–89°C. – IR: 2964, 2931, 2856, 1719, 1583, 1475, 1348, 1285, 1123 cm^{-1}. – HRMS: calcd for $C_{10}H_{18}O_4S_2Na$: 289.0539, found 289.0536 [M+Na$^+$].

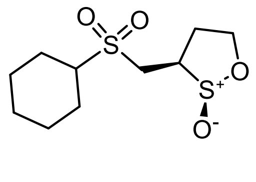

cis isomer: ^1H NMR (500 MHz, $CDCl_3$): 4.80 (1 H, td, *J* = 8.7, 2.7 Hz), 4.42 (1 H, ddd, *J* = 9.7, 8.7, 6.8 Hz), 3.59 (1 H, dq, *J* = 11.4, 7.0 Hz), 3.50 (1 H, dd, *J* = 13.5, 6.9 Hz), 3.15 (1 H, dd, *J* = 13.5, 6.7 Hz), 2.90 (1 H, tt, *J* = 12.3, 3.6 Hz), 2.57 (1 H, dtd, *J* = 12.9, 7.1, 2.7 Hz), 2.31 (1 H, dddd, *J* = 12.9, 11.5, 9.7, 8.7 Hz),

2.20 (2 H, tdq, *J* = 14.1, 4.9, 3.0, 2.4 Hz), 1.97–1.90 (2 H, m), 1.73 (1 H, dddd, *J* = 13.3, 6.7, 3.3, 1.6 Hz), 1.61–1.49 (2 H, m), 1.40–1.14 (3 H, m) ppm. – ^{13}C NMR (125 MHz, $CDCl_3$): 75.4, 62.7, 60.3, 46.9, 28.0, 25.5, 25.1, 25.1 ppm.

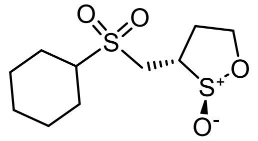

trans isomer: ^{1}H NMR (500 MHz, $CDCl_3$): 4.88 (1 H, td, *J* = 8.7, 4.7 Hz), 4.57 (1 H, dt, *J* = 9.1, 7.4 Hz), 3.85–3.74 (1 H, m), 3.26 (1 H, dd, *J* = 13.7, 5.0 Hz), 2.90 (1 H, tt, *J* = 12.3, 3.6 Hz), 2.86–2.75 (2 H, m), 2.41 (1 H, dddd, *J* = 13.5, 7.5, 4.7, 3.1 Hz), 2.20 (2 H, tdq, *J* = 14.1, 4.9, 3.0, 2.4 Hz), 1.99–1.89 (2 H, m), 1.73 (1 H, dddd, *J* = 13.3, 6.7, 3.3, 1.6 Hz), 1.61–1.46 (2 H, m), 1.40–1.10 (3 H, m) ppm. – ^{13}C NMR (125 MHz, $CDCl_3$): 75.3, 65.4, 60.3, 46.6, 28.2, 25.10, 25.07, 25.0 ppm.

cis-3-(((1-Phenylcyclopropyl)sulfonyl)methyl)-1,2-oxathiolane 2-oxide (2i)

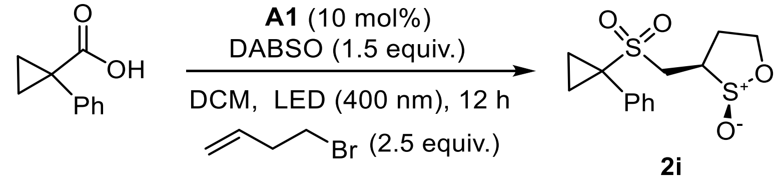

2i

According to **GP2**, the reaction was carried out with 1-phenylcyclopropane-1-carboxylic acid (49 mg, 0.3 mmol), DABSO (108 mg, 0.45 mmol, 1.5 equiv.), acridine catalyst **A1** (9 mg, 0.03 mmol, 10 mol%), 4-bromobut-1-ene (100 mg, 0.75 mmol, 2.5 equiv.) in degassed dichloromethane (3 mL). The test-tube was capped and the reaction mixture was irradiated with LED light (λ = 400 nm) while stirring at room temperature for 12 h. The reaction mixture was then quenched with a saturated solution of potassium hydrogen sulfate (2 mL) and extracted with ethyl acetate (3 × 10 mL). The organic layer was combined, dried over anhydrous sodium sulfate, concentrated under reduced pressure, and the remaining material was purified by flash chromatography on silica gel (hexane/ethyl acetate 3 : 2 v/v) to give inseparable mixture of sulfone products **2i** (cis/trans = 9/1, 53 mg, 59%) as a white solid.

m.p.: 108-110°C. – IR: 3035, 3002, 2987, 2873, 1765, 1563, 1165, 1087, 985 cm^{-1}. – HRMS: calcd for $C_{13}H_{16}O_4S_2Na$: 323.0382, found 323.0384 [M+Na$^+$].

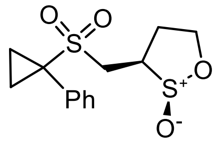

cis isomer: ^{1}H NMR (500 MHz, $CDCl_3$): 7.60 – 7.51 (2 H, m), 7.44–7.37 (3 H, m), 4.76 (1 H, td, *J* = 8.8, 2.7 Hz), 4.37 (1 H, td, *J* = 9.3, 6.9 Hz), 3.52 (1 H, dd, *J* = 13.6, 6.3 Hz), 3.40 (1 H, dq, *J* = 11.6, 7.2 Hz), 3.11 (1 H, dd, *J* = 13.6, 7.4 Hz), 2.48 (1 H, dtd, *J* = 12.9, 7.1, 2.6 Hz), 2.22 (1 H, ddt, *J* = 13.0, 11.6, 9.2 Hz), 1.97–1.77 (2 H, m), 1.44–1.26 (2 H, m) ppm. – ^{13}C NMR (125 MHz, $CDCl_3$): 133.6, 132.0, 129.6, 129.1, 75.6, 60.6, 47.7, 45.9, 27.8, 12.5, 12.4 ppm.

Go back to table of contents

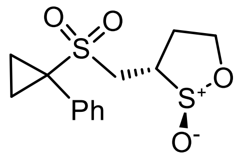

trans isomer: ^{1}H NMR (500 MHz, $CDCl_3$): 7.61–7.51 (2 H, m), 7.47–7.34 (3 H, m), 4.81 (1 H, dt, *J* = 8.7, 4.3 Hz), 4.46 (1 H, dt, *J* = 9.0, 7.3 Hz), 3.68–3.59 (1 H, m), 3.25 (1 H, dd, *J* = 13.9, 4.9 Hz), 2.79–2.66 (2 H, m), 2.32–2.25 (1 H, m), 1.93–1.81 (2 H, m), 1.39–1.29 (2 H, m) ppm. – ^{13}C NMR (125 MHz, $CDCl_3$): 133.5, 131.9, 129.8, 129.2, 75.2, 66.0, 47.5, 46.0, 28.0, 12.4, 12.3 ppm. – IR: 3035, 3002, 2987, 2873, 1765, 1563, 1165, 1087, 985 cm^{-1}.

(Ethylsulfonyl)cyclopentane (2j)

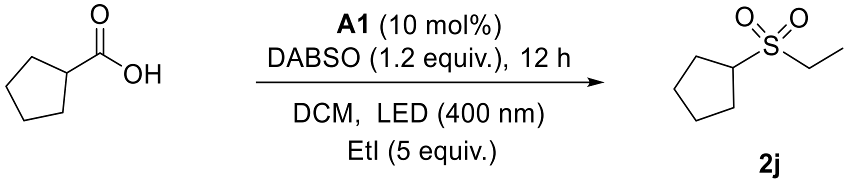

2j

According to **GP2**, the reaction was carried out with cyclopentanecarboxylic acid (34 mg, 0.3 mmol), DABSO (86 mg, 0.36 mmol, 1.2 equiv.), acridine catalyst **A1** (9 mg, 0.015 mmol, 10 mol%), ethyl iodide (234 mg, 1.5 mmol, 5 equiv.) in degassed dichloromethane (3 mL). The test-tube was capped and the reaction mixture was irradiated with LED light (λ = 400 nm) while stirring at room temperature for 12 h. The reaction mixture was then quenched with a saturated solution of potassium hydrogen sulfate (2 mL) and extracted with ethyl acetate (3 × 10 mL). The organic layer was combined, dried over anhydrous sodium sulfate, concentrated under reduced pressure, and the remaining material was purified by flash chromatography on silica gel (hexane/ethyl acetate 4 : 1 v/v) to give the sulfone product **2j** (65 mg, 63 %) as a yellow liquid.

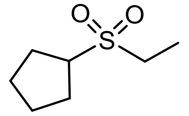

^{1}H NMR (500 MHz, $CDCl_3$): 3.45–3.30 (1 H, m), 2.95 (2 H, q, *J* = 7.5 Hz), 2.16–1.91 (4 H, m), 1.80 (2 H, td, *J* = 10.9, 10.2, 4.6 Hz), 1.65 (2 H, tdd, *J* = 12.3, 7.6, 4.1 Hz), 1.38 (3 H, t, *J* = 7.5 Hz) ppm. – ^{13}C NMR (125 MHz, $CDCl_3$): 60.21, 45.90, 26.79, 26.12, 6.40 ppm. – IR: 3063, 3006, 2946, 2935, 2867, 1422, 1256, 1213, 1156, 1086, 987 cm^{-1}. – HRMS: calcd for $C_7H_{15}O_2S$: 163.0787, found 163.0785 [M+H$^+$].

4-((2-(4,5-diphenyloxazol-2-yl)ethyl)sulfonyl)butan-2-one (2k)

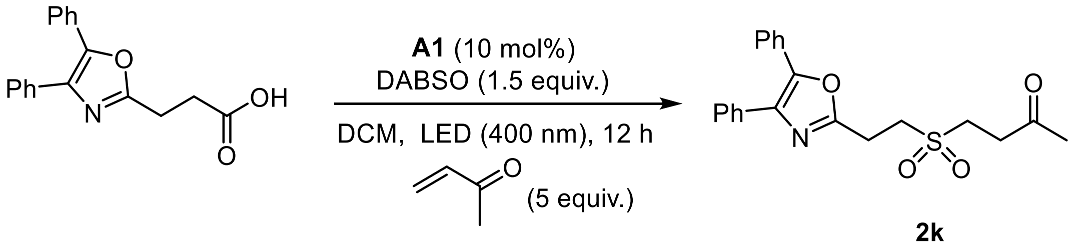

2k

According to **GP2**, the reaction was carried out with oxaprozin (88 mg, 0.3 mmol), DABSO (108 mg, 0.45 mmol, 1.5 equiv.), acridine catalyst **A1** (9 mg, 0.03 mmol, 10 mol%), but-3-en-2-one (105 mg, 1.5 mmol, 5 equiv.) in degassed dichloromethane (3 mL). The test-tube was capped and the reaction mixture was irradiated with LED light (λ = 400 nm) while stirring at room temperature for 12 h. The reaction mixture was then quenched with a saturated solution of potassium hydrogen sulfate (2 mL) and extracted with ethyl acetate (3 × 10 mL). The organic layer was combined, dried over anhydrous sodium sulfate, concentrated under reduced pressure, and the remaining material was purified by flash chromatography on silica gel (hexane/ethyl acetate 3 : 2 v/v) to give the sulfone product **2k** (104 mg, 91%) as a white solid.

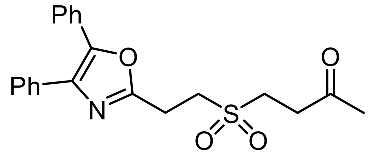

m.p.: 110–113°C. – ^{1}H NMR (500 MHz, $CDCl_3$): 7.64–7.58 (2 H, m), 7.60–7.54 (2 H, m), 7.41–7.29 (6 H, m), 3.60 (2 H, dd, *J* = 8.5, 6.9 Hz), 3.42 (2 H, dd, *J* = 8.6, 6.9 Hz), 3.36 (2 H, t, *J* = 7.2 Hz), 3.05 (2 H, t, *J* = 7.3 Hz), 2.20 (3 H, s) ppm. – ^{13}C NMR (125 MHz, $CDCl_3$): 203.9, 159.3, 146.3, 135.4, 132.2, 128.9, 128.84, 128.76, 128.6, 128.4, 128.0, 126.7, 50.5, 47.7, 35.5, 29.9, 21.6 ppm. – IR: 3053, 2996, 2897, 1421, 1264, 895, 735, 704 cm^{-1}. – HRMS: calcd for $C_{21}H_{22}NO_4S$: 384.1264, found 384.1259 [M+H$^+$].

3-((5-(5-Methylthiophen-2-yl)-5-oxopentyl)sulfonyl)cyclohexan-1-one (2l)

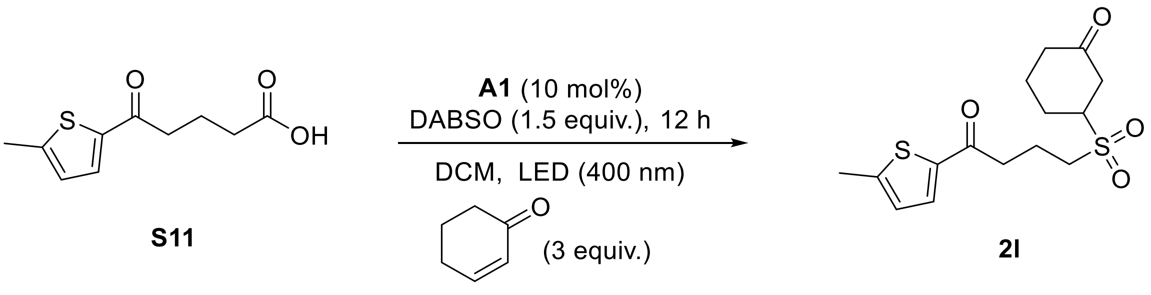

According to **GP2**, the reaction was carried out with acid **S11** (64 mg, 0.3 mmol), DABSO (108 mg, 0.45 mmol, 1.5 equiv.), acridine catalyst **A1** (9 mg, 0.015 mmol, 10 mol%), cyclohex-2-en-1-one (86 mg, 0.9 mmol, 3 equiv.) in degassed dichloromethane (3 mL). The test-tube was capped and the

reaction mixture was irradiated with LED light (λ = 400 nm) while stirring at room temperature for 12 h. The reaction mixture was then quenched with a saturated solution of potassium hydrogen sulfate (2 mL) and extracted with ethyl acetate (3 × 10 mL). The organic layer was combined, dried over anhydrous sodium sulfate, concentrated under reduced pressure, and the remaining material was purified by flash chromatography on silica gel (hexane/ethyl acetate 4 : 1 v/v) to give the sulfone product **2l** (65 mg, 63 %) as a yellow solid.

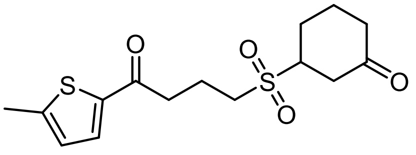

m.p.: 68–70°C. – ^{1}H NMR (500 MHz, $CDCl_3$): 7.53 (1 H, d, *J* = 3.8 Hz), 6.79 (1 H, dd, *J* = 3.8, 1.2 Hz), 3.32 (1 H, tt, *J* = 12.0, 4.3 Hz), 3.14–3.02 (4 H, m), 2.78 (1 H, ddt, *J* = 14.5, 4.4, 2.0 Hz), 2.65 (1 H, dd, *J* = 14.4, 12.4 Hz), 2.52 (3 H, d, *J* = 1.0 Hz), 2.44 (1 H, dtd, *J* = 14.7, 3.2, 1.7 Hz), 2.34 (2 H, dddd, *J* = 12.0, 9.6, 7.1, 5.7 Hz), 2.29–2.20 (3 H, m), 2.03–1.88 (1 H, m), 1.78–1.63 (1 H, m) ppm. – ^{13}C NMR (125 MHz, $CDCl_3$): 206.3, 191.0, 150.4, 141.4, 133.0, 127.0, 59.5, 49.4, 40.6, 39.7, 36.4, 23.5, 23.4, 16.8, 16.2 ppm. – IR: 3647, 2983, 2875, 1715, 1656, 1454, 1265, 1124, 894, 733 cm^{-1}. – HRMS: calcd for $C_{15}H_{21}O_4S_2$: 329.0876, found 329.0870 [M+H$^+$].

3-((((1*S,3*R**)-3-acetyl-2,2-dimethylcyclobutyl)methyl)sulfonyl)propanal (2m)**

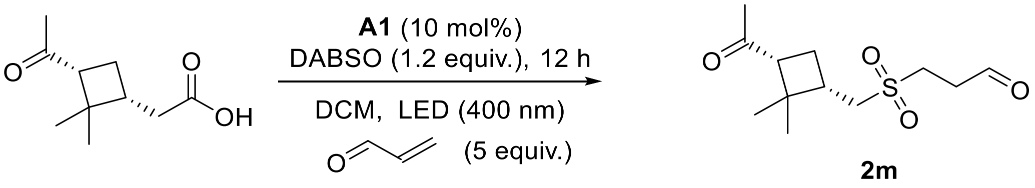

According to **GP3**, the reaction was carried out with *cis*-pinonic acid (55 mg, 0.3 mmol, 2.5 equiv.), DABSO (86 mg, 0.36 mmol, 1.2 equiv.), acridine catalyst **A1** (9 mg, 0.015 mmol, 10 mol%), (*E*)-hex-2-enal (29 mg, 0.3 mmol, 1 equiv.) in degassed dichloromethane (3 mL). The test-tube was capped and the reaction mixture was irradiated with LED light (λ = 400 nm) while stirring at room temperature for 12 h. The reaction mixture was then quenched with a saturated solution of potassium hydrogen sulfate (2 mL) and extracted with ethyl acetate (3 × 10 mL). The organic layer was combined, dried over anhydrous sodium sulfate, concentrated under reduced pressure, and the remaining material was purified by flash chromatography on silica gel (hexane/ethyl acetate 4 : 1 v/v) to give the sulfone product **2m** (57 mg, 73 %) as a yellow liquid.

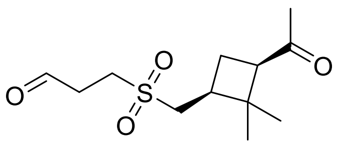

^{1}H NMR (500 MHz, $CDCl_3$): δ 9.81 (1 H, s), 3.26 (2 H, td, *J* = 7.1, 0.8 Hz), 3.06 (2 H, t, *J* = 6.8 Hz), 3.05–2.88 (3 H, m), 2.58 (1 H, dtd, *J* = 10.4, 8.1, 6.5 Hz), 2.25–1.99 (5 H, m), 1.37 (3 H, s), 0.91 (3 H, s) ppm. – ^{13}C NMR (125 MHz, $CDCl_3$): 206.9, 197.2, 54.5, 54.3, 46.5, 43.8, 35.8, 34.9, 30.3, 29.9, 23.4, 17.6 ppm. –

IR: 3025, 2987, 2930, 2847, 1732, 1568, 1487, 1385, 1270, 1173, 1048 cm^{-1}. – HRMS: calcd for $C_{12}H_{20}O_4SNa$: 283.0975, found 283.0977 [M+Na$^+$].

3-((Cyclopentylmethyl)sulfonyl)pentanal (2n)

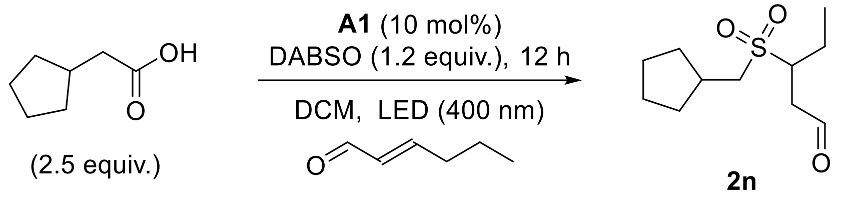

According to **GP3**, the reaction was carried out with 2-cyclopentylacetic acid (96 mg, 0.75 mmol, 2.5 equiv.), DABSO (86 mg, 0.36 mmol, 1.2 equiv.), acridine catalyst **A1** (9 mg, 0.015 mmol, 10 mol%), (*E*)-hex-2-enal (29 mg, 0.3 mmol, 1 equiv.) in degassed dichloromethane (3 mL). The test-tube was capped and the reaction mixture was irradiated with LED light (λ = 400 nm) while stirring at room temperature for 12 h. The reaction mixture was then quenched with a saturated solution of potassium hydrogen sulfate (2 mL) and extracted with ethyl acetate (3 × 10 mL). The organic layer was combined, dried over anhydrous sodium sulfate, concentrated under reduced pressure, and the remaining material was purified by flash chromatography on silica gel (hexane/ethyl acetate 4 : 1 v/v) to give the sulfone product **2n** (65 mg, 63 %) as a yellow liquid.

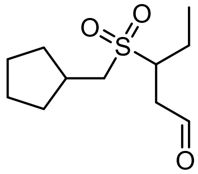

^{1}H NMR (500 MHz, $CDCl_3$): 9.83 (1 H, s), 3.58 (1 H, dtd, *J* = 10.0, 5.8, 4.4 Hz), 3.19 (1 H, ddd, *J* = 18.7, 6.0, 1.0 Hz), 3.05–2.92 (2 H, m), 2.70 (1 H, dd, *J* = 18.7, 5.6 Hz), 2.42 (1 H, tt, *J* = 9.1, 7.1 Hz), 2.07–1.87 (3 H, m), 1.71–1.53 (5 H, m), 1.52–1.21 (4 H, m), 0.96 (3 H, t, *J* = 7.3 Hz) ppm. – ^{13}C NMR (125 MHz, $CDCl_3$): 197.9, 56.7, 56.5, 41.7, 33.6, 33.1, 33.0, 31.1, 24.9, 24.9, 20.2, 14.0 ppm. – IR: 3065, 2968, 2956, 1783, 1325, 1289, 1187, 1098 cm^{-1}. – HRMS: calcd for $C_{12}H_{23}O_3S$: 247.1362, found 247.1357 [M+H$^+$].

Go back to table of contents

3-(((3*R*)-3-((3*R*,7*S*,8*R*,9*S*,10*S*,13*R*,14*S*,17*R*)-3,7-dihydroxy-10,13-dimethylhexadecahydro-1*H*-cyclopenta[a]phenanthren-17-yl)butyl)sulfonyl)propanenitrile (2o)

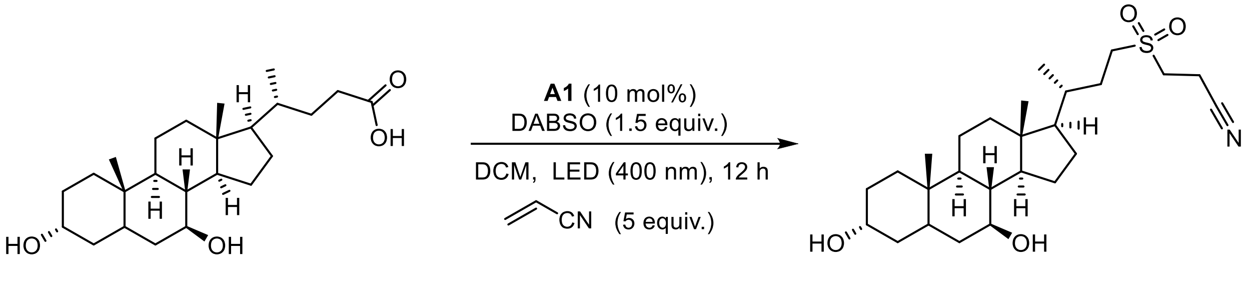

2o

According to **GP2**, the reaction was carried out with ursodeoxycholic acid (59 mg, 0.15 mmol), DABSO (54 mg, 0.225 mmol, 1.5 equiv.), acridine catalyst **A1** (5 mg, 0.015 mmol, 10 mol%), 4-bromobut-1-ene (100 mg, 0.75 mmol, 2.5 equiv.) in degassed dichloromethane (3 mL). The test-tube was capped and the reaction mixture was irradiated with LED light (λ = 400 nm) while stirring at room temperature for 12 h. The reaction mixture was then quenched with a saturated solution of potassium hydrogen sulfate (2 mL) and extracted with ethyl acetate (3 × 10 mL). The organic layer was combined, dried over anhydrous sodium sulfate, concentrated under reduced pressure, and the remaining material was purified by flash chromatography on silica gel (hexane/ethyl acetate 3 : 2 v/v) to give the sulfone product **2o** (58 mg, 83 %) as a white solid.

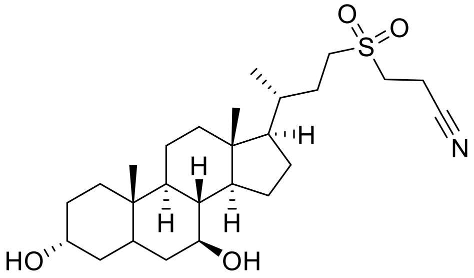

m.p.: 71–73°C. –$[\alpha]_D^{23}$ = + 29.4 (c 0.53, $CHCl_3$). – ^{1}H NMR (500 MHz, $CDCl_3$): 3.63–3.51 (2 H, m), 3.26 (2 H, t, *J* = 7.4 Hz), 3.14 (1 H, ddd, *J* = 13.9, 12.0, 4.2 Hz), 3.03–2.87 (3 H, m), 2.02–1.74 (6 H, m), 1.72–1.54 (7 H, m), 1.55–1.19 (11 H, m), 1.19–1.00 (2 H, m), 0.98 (3 H, d, *J* = 6.1 Hz), 0.94 (3 H, s), 0.68 (3 H, s) ppm. – ^{13}C NMR (125 MHz, $CDCl_3$): 116.70, 71.49, 71.36, 55.83, 54.70, 51.36, 47.81, 43.96, 43.86, 42.55, 40.21, 39.28, 37.42, 37.18, 35.03, 34.97, 34.19, 30.46, 28.73, 27.79, 26.95, 23.49, 21.27, 18.59, 12.30, 11.42 ppm. – IR: 3053, 2986, 2963, 2847, 1422, 1264, 895, 785 cm^{-1}. – HRMS: calcd for $C_{26}H_{43}NNaO_4S$: 488.2805, found 488.2789 [M+Na$^+$].

(2*R*,4a*S*,6a*S*,6b*R*,10*S*,12a*S*,14b*R*)-10-Hydroxy-2,4a,6a,6b,9,9,12a-heptamethyl-2-((2-(phenylsulfonyl)ethyl)sulfonyl)-1,3,4,4a,5,6,6a,6b,7,8,8a,9,10,11,12,12a,12b,14b-octadecahydropicen-13(2*H*)-one (2p)

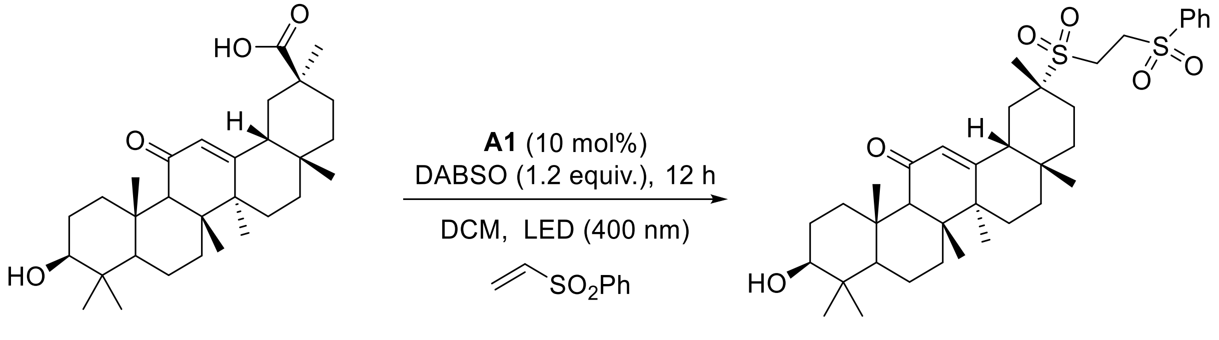

2

According to **GP2**, the reaction was carried out with enoxolone (71 mg, 0.15 mmol), DABSO (43 mg, 0.18 mmol, 1.2 equiv.), acridine catalyst **A1** (5 mg, 0.015 mmol, 10 mol%), (vinylsulfonyl)benzene (63 mg, 0.375 mmol, 2.5 equiv.) in degassed dichloromethane (3 mL). The test-tube was capped and the reaction mixture was irradiated with LED light (λ = 400 nm) while stirring at room temperature for 12 h. The reaction mixture was then quenched with a saturated solution of potassium hydrogen sulfate (2 mL) and extracted with ethyl acetate (3 × 10 mL). The organic layer was combined, dried over anhydrous sodium sulfate, concentrated under reduced pressure, and the remaining material was purified by flash chromatography on silica gel (hexane/ethyl acetate 1 : 4 v/v) to give the sulfone product **2p** (60 mg, 61 %) as a yellow solid.

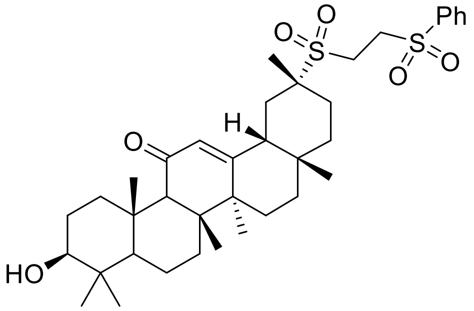

m.p.: 162–164°C. –$[\alpha]_D^{23}$ = + 24.5 (c 0.39, $CHCl_3$). – ^1H NMR (500 MHz, $CDCl_3$): 7.95–7.90 (2 H, m), 7.75–7.68 (1 H, m), 7.61 (2 H, t, *J* = 7.8 Hz), 5.60 (1 H, s), 3.57–3.46 (2 H, m), 3.39–3.28 (2 H, m), 3.23 (1 H, dd, *J* = 10.8, 5.5 Hz), 2.75 (1 H, dt, *J* = 13.6, 3.6 Hz), 2.43 (1 H, t, *J* = 13.3 Hz), 2.33 (1 H, s), 2.19–1.98 (3 H, m), 1.82 (1 H, td, *J* = 13.8, 4.9 Hz), 1.70–1.31 (15 H, m), 1.29–1.17 (2 H, m), 1.12 (6 H, brs), 1.06– 0.92 (5 H, m), 0.92–0.81 (4 H, m), 0.80 (3 H, s), 0.70 (1 H, d, *J* = 11.3 Hz) ppm. – ^{13}C NMR (125 MHz, $CDCl_3$): 199.1, 166.7, 138.6, 134.7, 129.8, 129.4, 128.1, 78.8, 64.2, 62.0, 55.0, 48.1, 46.3, 45.6, 43.4, 39.3, 39.2, 39.0, 37.2, 35.0, 33.9, 32.8, 32.4, 28.2, 27.4, 26.25, 26.19, 24.6, 23.7, 18.8, 17.6, 17.1, 16.5, 15.7 ppm. – IR: 335, 2933, 2857, 1754, 1712, 1505, 1453, 1292, 1366, 1298, 1268, 1163, 1124, 1053 cm^{-1}. – HRMS: calcd for $C_{37}H_{55}O_6S_2$: 659.3435, found 659.3410 $[M+H^+]$.

1-(Cyclohexylsulfonyl)isoquinoline (2q)

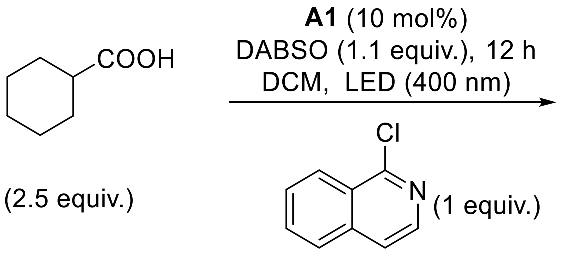

According to **GP3**, the reaction was carried out with 1-chloroisoquinoline (49 mg, 0.3 mmol), cyclohexanecarboxylic acid (96 mg, 0.75 mmol, 2.5 equiv.), DABSO (79 mg, 0.33 mmol, 1.1 equiv.), acridine catalyst **A1** (9 mg, 0.03 mmol, 10 mol%), in degassed dichloromethane (3 mL). The test-tube was capped and the reaction mixture was irradiated with LED light (λ = 400 nm) while stirring at room temperature for 12 h. The reaction mixture was then quenched with a saturated solution of potassium hydrogen sulfate (2 mL) and extracted with ethyl acetate (3 × 10 mL). The organic layer was combined, dried over anhydrous sodium sulfate, concentrated under reduced pressure, and the remaining material was purified by flash chromatography on silica gel (hexane/ethyl acetate 7 : 3 v/v) to give the sulfone product **2q** (72 mg, 87%) as a yellow solid.

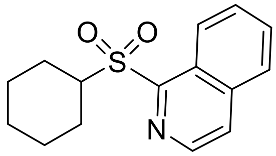

m.p.: 85–87 °C. – ^{1}H NMR (300 MHz, $CDCl_3$): ^{1}H NMR (500 MHz, Chloroform-*d*) δ 9.14–9.09 (1 H, m), 8.56 (1 H, d, *J* = 5.5 Hz), 7.92 (1 H, dd, *J* = 8.0, 1.3 Hz), 7.86 (1 H, d, *J* = 5.5 Hz), 7.78 (1 H, ddd, *J* = 8.1, 6.8, 1.3 Hz), 7.73 (1 H, ddd, *J* = 8.4, 6.9, 1.4 Hz), 3.93 (1 H, tt, *J* = 12.2, 3.4 Hz), 2.18–2.07 (2 H, m), 1.90 (2 H, dt, *J* = 12.4, 3.1 Hz), 1.75–1.62 (3 H, m), 1.40 –1.19 (3 H, m) ppm. – ^{13}C NMR (125 MHz, $CDCl_3$): 155.4, 140.6, 137.8, 131.3, 129.4, 127.6, 125.49, 125.46, 125.2, 60.2, 25.4, 25.2, 25.1 ppm. – IR: 3056, 3004, 2987, 2963, 2855, 1789, 1588, 1476, 1312, 1287, 1127 cm^{-1}. – HRMS: calcd for $C_{15}H_{18}NO_2S$: 276.1053, found 276.1054 [M+H$^+$].

4-(Heptan-4-ylsulfonyl)quinoline (2r)

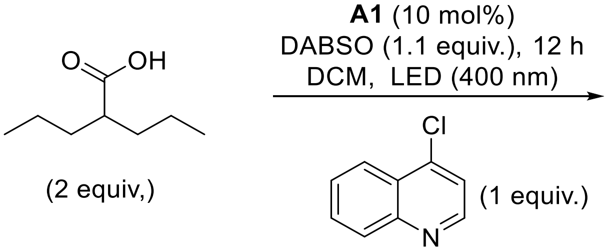

According to **GP3**, the reaction was carried out with 4-chloroquinoline (48 mg, 0.3 mmol), valproic acid (108 mg, 0.6 mmol, 2 equiv.), DABSO (79 mg, 0.33 mmol, 1.1 equiv.), acridine catalyst **A1** (9 mg, 0.03 mmol, 10 mol%), in degassed dichloromethane (6 mL). The test-tube was

capped and the reaction mixture was irradiated with LED light (λ = 400 nm) while stirring at room temperature for 12 h. The reaction mixture was then quenched with a saturated solution of potassium hydrogen sulfate (2 mL) and extracted with ethyl acetate (3 × 10 mL). The organic layer was combined, dried over anhydrous sodium sulfate, concentrated under reduced pressure, and the remaining material was purified by flash chromatography on silica gel (hexane/ethyl acetate 7 : 3 v/v) to give the sulfone product **2r** (60 mg, 69%) as a colorless liquid.

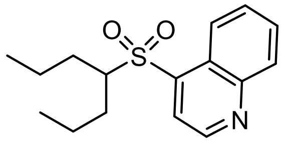

^{1}H NMR (300 MHz, $CDCl_3$): 9.14 (1 H, d, *J* = 4.4 Hz), 8.66 (1 H, ddd, *J* = 8.5, 1.4, 0.6 Hz), 8.27 (1 H, ddd, *J* = 8.5, 1.4, 0.6 Hz), 8.06 (1 H, d, *J* = 4.4 Hz), 7.86 (1 H, ddd, *J* = 8.4, 6.9, 1.4 Hz), 7.75 (1 H, ddd, *J* = 8.4, 6.9, 1.4 Hz), 3.22 (1 H, tt, *J* = 7.0, 4.8 Hz), 1.87–1.59 (4 H, m), 1.55–1.19 (4 H, m), 0.81 (3 H, t, *J* = 7.3 Hz) ppm. – ^{13}C NMR (125 MHz, $CDCl_3$): 149.6, 149.6, 142.4, 130.9, 130.5, 129.1, 124.2, 123.1, 123.1, 64.1, 29.6, 19.9, 13.9 ppm. – IR: 3087, 3066, 2961, 2934, 2873, 2145, 1739, 1580, 1499, 1459, 1313, 1217, 1158, 1127, 1093, 764, 710 cm^{-1}. – HRMS: calcd for $C_{16}H_{21}NNaO_2S$: 314.1185, found 314.1192 [M+Na$^+$].

7-Chloro-4-((4-chlorobutyl)sulfonyl)quinoline (2s)

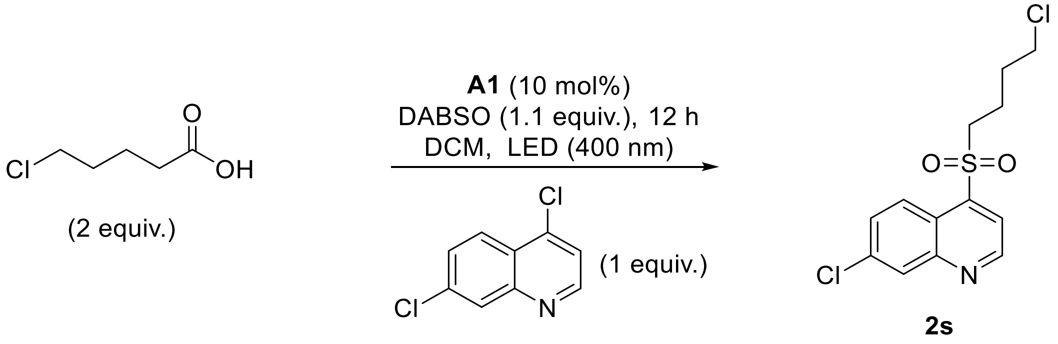

According to **GP3**, the reaction was carried out with 4,7-dichloroquinoline (59 mg, 0.3 mmol), 5-chlorovaleric acid (82 mg, 0.6 mmol, 2 equiv.), DABSO (79 mg, 0.33 mmol, 1.1 equiv.), acridine catalyst **A1** (9 mg, 0.03 mmol, 10 mol%) in degassed dichloromethane (6 mL). The test-tube was capped and the reaction mixture was irradiated with LED light (λ = 400 nm) while stirring at room temperature for 12 h. The reaction mixture was then quenched with a saturated solution of potassium hydrogen sulfate (2 mL) and extracted with ethyl acetate (3 × 10 mL). The organic layer was combined, dried over anhydrous sodium sulfate, concentrated under reduced pressure, and the remaining material was purified by flash chromatography on silica gel (hexane/ ethyl acetate 4 : 1 v/v) to give the sulfone product **2s** (79 mg, 83%) as a colorless solid.

Gram scale for compound 2s: According to **GP3**, the reaction was carried out with 4,7-dichloroquinoline (1.19 g, 6 mmol), 5-chlorovaleric acid (1.64 g, 12 mmol, 2 equiv.), DABSO (1.58 g, 6.6 mmol, 1.1 equiv.), acridine catalyst **A1** (179 mg, 0.6 mmol, 10 mol%) in degassed dichloromethane (80 mL). The test-tube was capped and the reaction mixture was irradiated with LED light (λ = 400 nm) while stirring at room temperature for 16 h. The reaction mixture was then quenched with a saturated solution of potassium hydrogen sulfate (20 mL) and extracted with ethyl acetate (3 × 50 mL). The organic layer was combined, dried over anhydrous sodium sulfate, concentrated under reduced pressure, and the remaining material was purified by flash chromatography on silica gel (hexane/ ethyl acetate 4 : 1 v/v) to give the sulfone product **2s** (1.56 g, 82%) as a colorless solid.

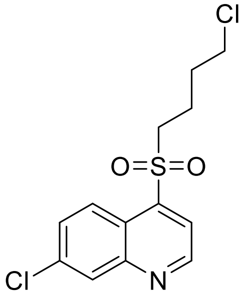

m.p.: 62– 64°C. – ^{1}H NMR (300 MHz, $CDCl_3$): 9.13 (1 H, d, *J* = 4.4 Hz), 8.61 (1 H, d, *J* = 9.1 Hz), 8.24 (1 H, d, *J* = 2.2 Hz), 8.02 (1 H, d, *J* = 4.4 Hz), 7.69 (1 H, dd, *J* = 9.1, 2.2 Hz), 3.47 (2 H, t, *J* = 5.8 Hz), 3.29 (2 H, dd, *J* = 8.8, 6.0 Hz), 1.86 (4 H, m) ppm. – ^{13}C NMR (75 MHz, $CDCl_3$): 151.0, 149.9, 142.8, 136.9, 130.3, 129.9, 125.3, 122.5, 121.1, 55.4, 43.8, 30.7, 20.1 ppm. – IR: 3113, 3074, 2943, 2913, 2874, 1736, 1606, 1577, 1556, 1488, 1403, 1156, 1196, 1077, 1046, 981, 970, 819, 780 cm^{-1}. – HRMS: calcd for $C_{13}H_{14}Cl_2NO_2S$: 318.0117, found 318.0120 [M+H$^+$].

7-Chloro-4-((1-methylcyclohexyl)sulfonyl)quinoline (2t)

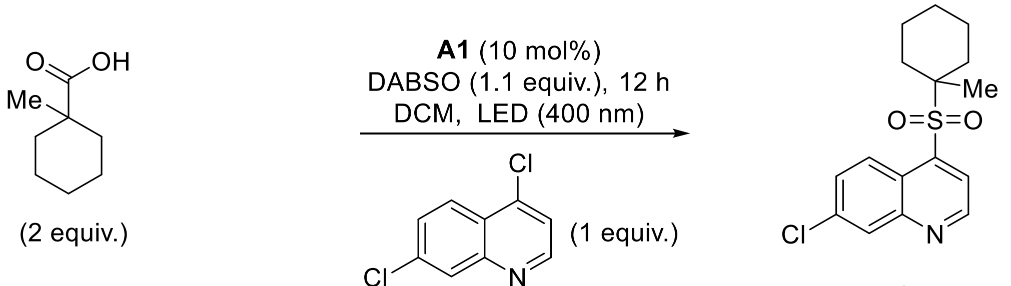

2t

According to **GP3**, the reaction was carried out with 4,7-dichloroquinoline (59 mg, 0.3 mmol), 1-methylcyclohexane-1-carboxylic acid (85 mg, 0.6 mmol, 2 equiv.), DABSO (79 mg, 0.33 mmol, 1.1 equiv.), acridine catalyst **A1** (9 mg, 0.03 mmol, 10 mol%) in degassed dichloromethane (6 mL). The test-tube was capped and the reaction mixture was irradiated with LED light (λ = 400 nm) while stirring at room temperature for 12 h. The reaction mixture was then quenched with a saturated solution of potassium hydrogen sulfate (2 mL) and extracted with ethyl acetate (3 × 10 mL). The organic layer was combined, dried over anhydrous sodium sulfate, concentrated under

reduced pressure, and the remaining material was purified by flash chromatography on silica gel (hexane/ ethyl acetate 3 : 2 v/v) to give the sulfone product **2t** (61 mg, 63%) as a colorless solid.

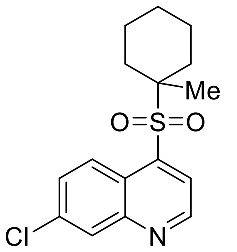

m.p.: 85–87 °C. – ^{1}H NMR (300 MHz, $CDCl_3$): 9.11 (1 H, d, *J* = 4.5 Hz), 8.91 (1 H, d, *J* = 9.3 Hz), 8.21 (1 H, d, *J* = 2.2 Hz), 7.96 (1 H, d, *J* = 4.4 Hz), 7.64 (1 H, dd, *J* = 9.3, 2.3 Hz), 1.86 (2 H, td, *J* = 12.8, 12.2, 4.4 Hz), 1.78–1.58 (5 H, m), 1.47–1.25 (5 H, m), 1.20–1.01 (1 H, m) ppm. – ^{13}C NMR (75 MHz, $CDCl_3$): 150.5, 150.1, 140.4, 136.6, 129.8, 129.3, 127.4, 125.9, 123.6, 65.9, 30.1, 25.0, 21.5, 17.1 ppm. – IR: 3131, 3083, 3003, 2938, 2861, 1605, 1556, 1488, 1464, 1439, 1307, 1288, 1175, 1134, 1092, 1080, 973, 922, 875, 823, 698 cm^{-1}. – HRMS: calcd for $C_{16}H_{19}ClNO_2S$: 324.0820, found 324.0819 [M+H$^+$].

7-Chloro-4-((3,4-dimethoxyphenethyl)sulfonyl)quinoline (2u)

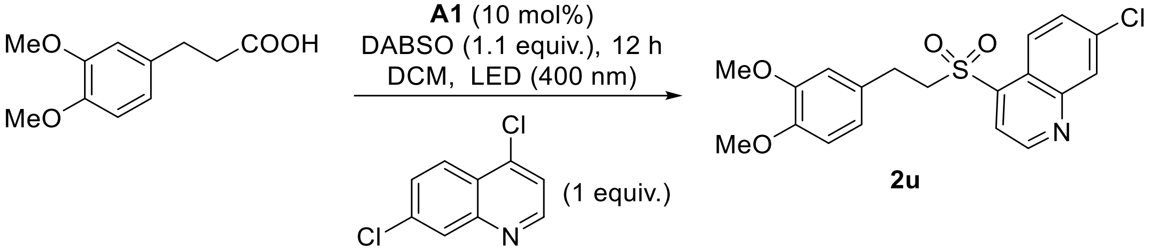

According to **GP3**, the reaction was carried out with 4,7-dichloroquinoline (59 mg, 0.3 mmol), 3-(3,4-dimethoxyphenyl)propanoic acid (126 mg, 0.6 mmol, 2 equiv.), DABSO (79 mg, 0.33 mmol, 1.1 equiv.), acridine catalyst **A1** (9 mg, 0.03 mmol, 10 mol%), in degassed dichloromethane (6 mL). The test-tube was capped and the reaction mixture was irradiated with LED light (λ = 400 nm) while stirring at room temperature for 12 h. The reaction mixture was then quenched with a saturated solution of potassium hydrogen sulfate (2 mL) and extracted with ethyl acetate (3 × 10 mL). The organic layer was combined, dried over anhydrous sodium sulfate, concentrated under reduced pressure, and the remaining material was purified by flash chromatography on silica gel (hexane/ethyl acetate 2 : 3 v/v) to give the sulfone product **2u** (86 mg, 73%) as a white solid.

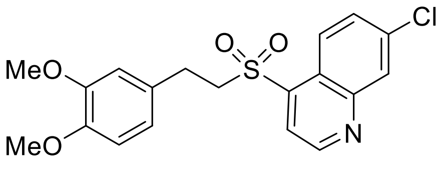

m.p.: 119–121°C. – ^{1}H NMR (500 MHz, $CDCl_3$): 9.08 (1 H, t, *J* = 4.7 Hz), 8.70–8.56 (1 H, m), 8.23 (1 H, dt, *J* = 5.1, 2.5 Hz), 7.97 (1 H, d, *J* = 4.7 Hz), 7.68 (1 H, dd, *J* = 9.6, 2.7 Hz), 6.68–6.58 (1 H, m), 6.58–6.44 (2 H, m), 3.78 (3 H, d, *J* = 3.2 Hz), 3.73 (3 H, d, *J* = 2.7 Hz), 3.61–3.49 (2 H, m), 3.08–2.95 (2 H, m) ppm. – ^{13}C NMR (125 MHz, $CDCl_3$): 150.9, 149.8, 149.1, 148.1, 143.0, 136.8, 130.2, 129.8, 129.0, 125.3, 122.5, 121.0, 120.3, 111.5, 111.4, 57.6, 56.0, 55.9, 28.3 ppm. – IR: 3042, 3006, 2932, 2834, 1606, 1591, 1338, 1438, 1314, 1156 cm^{-1}. – HRMS: calcd for $C_{19}H_{19}ClNO_4S$: 392.0718, found 392.0716 [M+H$^+$].

7-Chloro-4-((tetrahydro-2*H*-pyran-4-yl)sulfonyl)quinoline (2v)

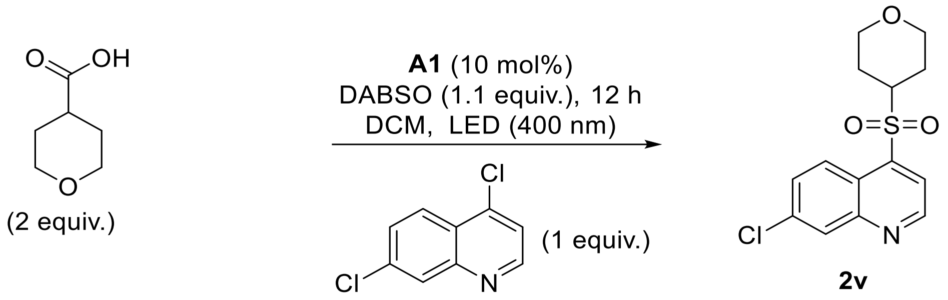

According to **GP3**, the reaction was carried out with 4,7-dichloroquinoline (59 mg, 0.3 mmol), tetrahydro-2*H*-pyran-4-carboxylic acid (78 mg, 0.6 mmol, 2 equiv.), DABSO (79 mg, 0.33 mmol, 1.1 equiv.), acridine catalyst **A1** (9 mg, 0.03 mmol, 10 mol%) in degassed dichloromethane (6 mL). The test-tube was capped and the reaction mixture was irradiated with LED light (λ = 400 nm) while stirring at room temperature for 12 h. The reaction mixture was then quenched with a saturated solution of potassium hydrogen sulfate (2 mL) and extracted with ethyl acetate (3 × 10 mL). The organic layer was combined, dried over anhydrous sodium sulfate, concentrated under reduced pressure, and the remaining material was purified by flash chromatography on silica gel (hexane/ ethyl acetate 2 : 3 v/v) to give the sulfone product **2v** (85 mg, 91%) as a colorless solid.

Gram scale for compound 2v: According to **GP3**, the reaction was carried out with 4,7-dichloroquinoline (0.99 g, 5 mmol), tetrahydro-2*H*-pyran-4-carboxylic acid (1.30 g, 10 mmol, 2 equiv.), DABSO (1.32 g, 5.5 mmol, 1.1 equiv.), acridine catalyst **A1** (149 mg, 0.5 mmol, 10 mol%) in degassed dichloromethane (80 mL). The test-tube was capped and the reaction mixture was irradiated with LED light (λ = 400 nm) while stirring at room temperature for 16 h. The reaction mixture was then quenched with a saturated solution of potassium hydrogen sulfate (20 mL) and extracted with ethyl acetate (3 × 50 mL). The organic layer was combined, dried over anhydrous sodium sulfate, concentrated under reduced pressure, and the remaining material was purified by flash chromatography on silica gel (hexane/ ethyl acetate 3 : 2 v/v) to give the sulfone product **2v** (1.46 g, 92%) as a colorless solid.

Decagram scale for compound 2v: According to **GP3**, the reaction was carried out with 4,7-dichloroquinoline (8.3 g, 42 mmol), tetrahydro-2*H*-pyran-4-carboxylic acid (10.9 g, 82 mmol, 2 equiv.), DABSO (11.1 g, 46 mmol, 1.1 equiv.), acridine catalyst **A1** (1.3 g, 4.2 mmol, 10 mol%), degassed dichloromethane (500 mL) in a round bottom 1L flask. The flask was capped, and the reaction mixture was irradiated with LED light (λ = 400 nm) while stirring at room temperature for 24 h. The reaction mixture was then quenched with a saturated solution of potassium hydrogen sulfate (200 mL) and extracted with ethyl acetate (3 × 150 mL). The organic layer was

combined, dried over anhydrous sodium sulfate, concentrated under reduced pressure, and the remaining material was purified by flash chromatography on silica gel (hexane/ ethyl acetate 3 : 2 v/v) to give the sulfone product **2v** (10.1 g, 77%) as a colorless solid.

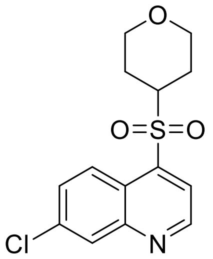

m.p.: 145–147°C °C. – ^{1}H NMR (300 MHz, $CDCl_3$): 9.13 (1 H, d, *J* = 4.4 Hz), 8.69 (1 H. d, *J* = 9.2 Hz), 8.25 (1 H, d, *J* = 2.2 Hz), 8.00 (1 H, d, *J* = 4.4 Hz), 7.71 (1 H, d, *J* = 2.2 Hz), 4.03 (ddd, *J* = 11.9, 4.6, 1.9 Hz, 1H)), 3.46–3.16 (3 H, m), 1.92 (2 H, dtd, *J* = 13.1, 11.8, 4.7 Hz), 1.77 (2 H, ddq, *J* = 13.0, 4.4, 2.3 Hz). ppm. – ^{13}C NMR (75 MHz, $CDCl_3$): 150.9, 150.0, 141.1, 137.0, 130.3, 129.8, 125.7, 123.7, 121.7, 66.3, 61.1, 25.2 ppm. – IR: 3077, 2959, 2926, 2854, 1605, 1489, 1365, 1314, 1299, 1157, 1123, 1060, 1021, 989, 880, 817, 781, 692 cm^{-1}. – HRMS: calcd for $C_{14}H_{15}ClNO_3S$: 312.0456, found 312.0458 [M+H^+].

Sulfinate Salts

Sodium 2-(4-fluorophenyl)ethane-1-sulfinate (3a)

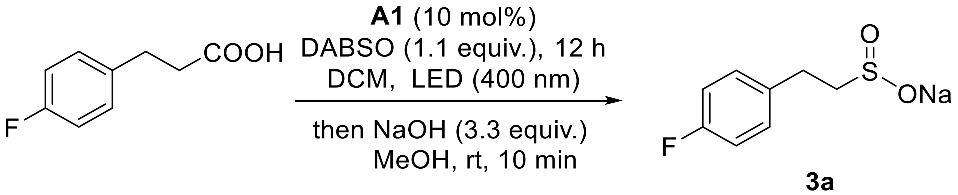

3a

According to **GP3**, the reaction was carried out with 3-(4-fluorophenyl)propanoic acid (50 mg, 0.3 mmol), DABSO (79 mg, 0.33 mmol, 1.1 equiv.), acridine catalyst **A1** (9 mg, 0.03 mmol, 10 mol%) in degassed dichloromethane (6 mL). The test-tube was capped and the reaction mixture was irradiated with LED light (λ = 400 nm) while stirring at room temperature for 12 h. After completion, a 1M solution of sodium hydroxide (1 mL, 1 mmol, 3.3 equiv.) in MeOH was added. The mixture was stirred for 10 min and purified by flash chromatography on silica gel (DCM/ MeOH 7 : 3 v/v) to give the sulfinate salt **3a** (44 mg, 70%) as a white solid.

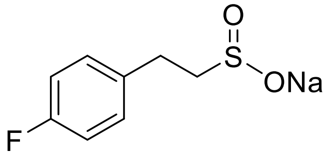

m.p.: >200 °C. – ^{1}H NMR (500 MHz, MeOD): 7.22 (2 H, dd, *J* = 8.6, 5.5 Hz), 6.96 (2 H, t, *J* = 8.8 Hz), 3.03–2.73 (2 H, m), 2.60–2.38 (2 H, m) ppm. – ^{13}C NMR (125 MHz, MeOD): 162.7 (d, *J* = 242.1 Hz), 138.3, 131.0 (d, *J* = 8.0 Hz), 115.9 (d, *J* = 21.5 Hz), 64.4, 28.9 ppm. – ^{19}F NMR (470 MHz, $CDCl_3$): –117.54 (td, *J* = 9.0, 8.5, 4.6 Hz) ppm. – IR: 3083, 3003, 2576, 185, 1606, 1510, 1242, 1177, 1133, 1091 cm^{-1}. – HRMS: calcd for $C_8H_8FO_2S$: 187.0235, found 187.0228 [M^-].

Sodium 2-(3,4-dimethoxyphenyl)ethane-1-sulfinate (3b)

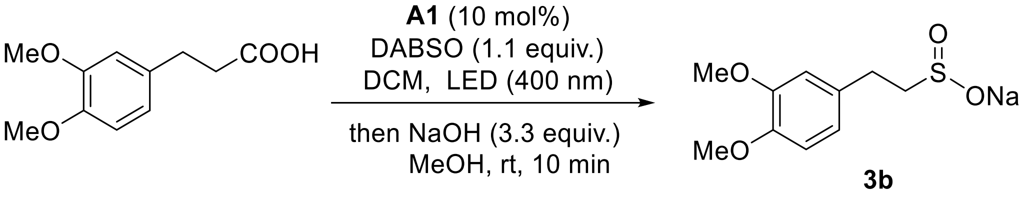

According to **GP3**, the reaction was carried out with 3-(3,4-dimethoxyphenyl)propanoic acid (63 mg, 0.3 mmol), DABSO (79 mg, 0.33 mmol, 1.1 equiv.), acridine catalyst **A1** (9 mg, 0.03 mmol, 10 mol%) in degassed dichloromethane (6 mL). The test-tube was capped and the reaction mixture was irradiated with LED light (λ = 400 nm) while stirring at room temperature for 12 h. After completion, a 1M solution of sodium hydroxide (1 mL, 1 mmol, 3.3 equiv.) in MeOH was added. The mixture was stirred for 10 min and purified by flash chromatography on silica gel (DCM/ MeOH 1 : 1 v/v) to give the sulfinate salt **3b** (48 mg, 63%) as a white solid.

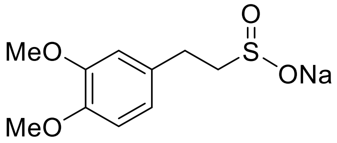

m.p.: >200 °C. – ^{1}H NMR (500 MHz, MeOD): 6.89–6.80 (2 H, m), 6.77 (1 H, dd, *J* = 8.2, 2.0 Hz), 3.80 (6 H, d, *J* = 13.3 Hz), 2.89–2.79 (2 H, m), 2.57–2.39 (2 H, m) ppm. – ^{13}C NMR (125 MHz, MeOD): 148.9, 147.2, 134.1, 120.3, 112.1, 111.8, 63.3, 55.2, 55.0, 27.9 ppm. – IR: 2938, 2835, 2012, 1606, 1514, 1462, 1449, 1418, 1233, 1140, 1020 cm^{-1}. – HRMS: calcd for $C_{10}H_{13}Na_2O_4S$: 275.0324, found 275.0321 [M+Na$^+$].

Sodium 4-oxo-4-phenylbutane-1-sulfinate (3c)

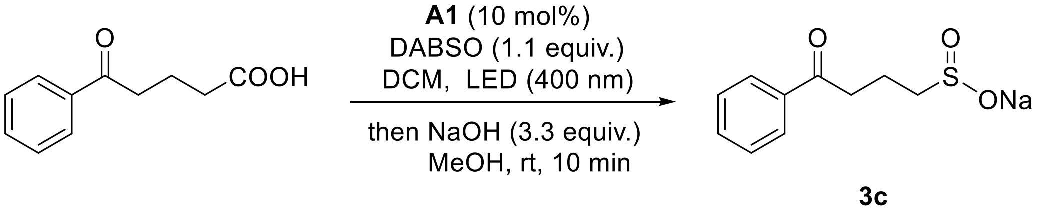

According to **GP3**, the reaction was carried out with 5-oxo-5-phenylpentanoic acid (58 mg, 0.3 mmol), DABSO (79 mg, 0.33 mmol, 1.1 equiv.), acridine catalyst **A1** (9 mg, 0.03 mmol, 10 mol %) in degassed dichloromethane (6 mL). The test-tube was capped and the reaction mixture was irradiated with LED light (λ = 400 nm) while stirring at room temperature for 12 h. After completion, a 1M solution of sodium hydroxide (1 mL, 1 mmol, 3.3 equiv.) in MeOH was added. The mixture was stirred for 10 min and purified by flash chromatography on silica gel (DCM/ MeOH 3 : 2 v/v) to give the sulfinate salt **3c** (46 mg, 66%) as a white solid.

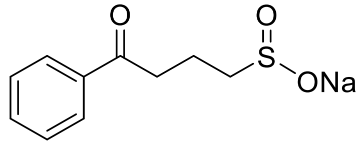

m.p.: >200 °C. – ^{1}H NMR (500 MHz, MeOD): 8.07–7.80 (2 H, m), 7.69–7.55 (1 H, m), 7.51 (2 H, dd, *J* = 8.4, 7.0 Hz), 3.18 (2 H, t, *J* = 7.3 Hz), 2.38 (2 H, dd, *J* = 8.4, 6.9 Hz), 2.04 (2 H, p, *J* = 7.5 Hz) ppm. – ^{13}C NMR (125 MHz, MeOD): 202.0, 138.3, 134.2, 129.7, 129.2, 62.2, 38.8, 18.8 ppm. – IR: 3169, 3045, 2983, 2939, 1978, 1895, 1606, 1510, 1243, 1177, 1050, 978 cm^{-1}. – HRMS: calcd for $C_{10}H_{11}NaO_3S$: 211.0434, found 211.0430 [M$^-$].

Sodium 4-(5-methylthiophen-2-yl)-4-oxobutane-1-sulfinate (3d)

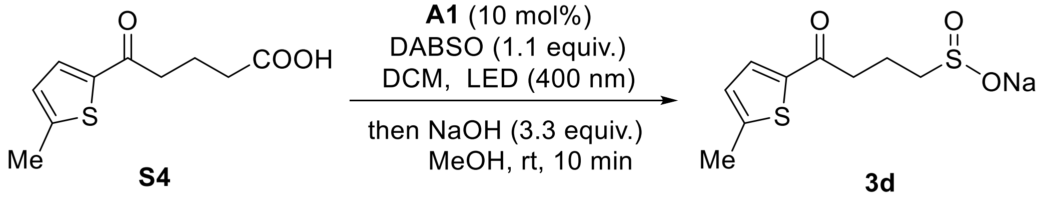

According to **GP3**, the reaction was carried out with acid **S11** (64 mg, 0.3 mmol), DABSO (79 mg, 0.33 mmol, 1.1 equiv.), acridine catalyst **A1** (9 mg, 0.03 mmol, 10 mol%) in degassed dichloromethane (6 mL). The test-tube was capped and the reaction mixture was irradiated with LED light (λ = 400 nm) while stirring at room temperature for 12 h. After completion, a 1M solution of sodium hydroxide (1 mL, 1 mmol, 3.3 equiv.) in MeOH was added. The mixture was stirred for 10 min and purified by flash chromatography on silica gel (DCM/ MeOH 3 : 2 v/v) to give the sulfinate salt **3d** (63 mg, 83%) as a white solid.

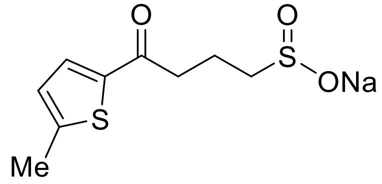

m.p.: >200 °C. – ^{1}H NMR (500 MHz, MeOD): 7.67 (1 H, d, *J* = 3.7 Hz), 6.85 (1 H, d, *J* = 4.4 Hz), 2.98 (2 H, t, *J* = 7.4 Hz), 2.48 (3 H, s), 2.30 (2 H, dd, *J* = 8.6, 6.7 Hz), 1.95 (2 H, p, *J* = 7.5 Hz) ppm. – ^{13}C NMR (125 MHz, MeOD): 195.0, 151.8, 142.8, 135.1, 128.3, 61.0, 38.8, 19.3, 15.9 ppm. – IR: 3320, 2944, 2831, 2206, 2071, 1671, 1427, 1278, 1023, 980 cm^{-1}. – HRMS: calcd for $C_9H_{12}NaO_3S_2$: 255.0120, found 255.0120 [M+H$^+$].

Sodium 5-(4-chlorobenzamido)pentane-1-sulfinate (3e)

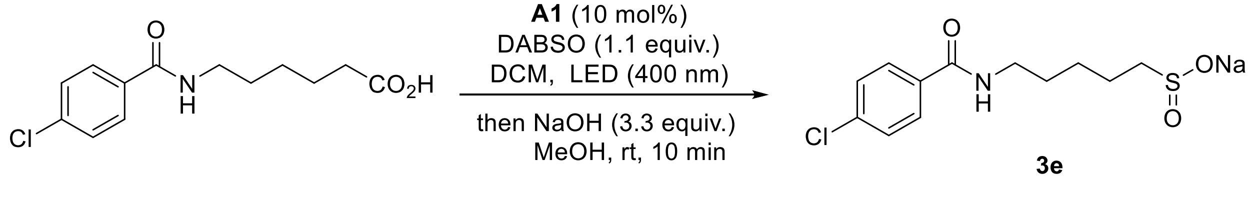

According to **GP3**, the reaction was carried out with tetrahydro-2*H*-pyran-4-carboxylic acid (81 mg, 0.3 mmol), DABSO (79 mg, 0.33 mmol, 1.1 equiv.), acridine catalyst **A1** (9 mg, 0.03 mmol, 10

mol%) in degassed dichloromethane (6 mL). The test-tube was capped and the reaction mixture was irradiated with LED light (λ = 400 nm) while stirring at room temperature for 12 h. After completion, a 1M solution of sodium hydroxide (1 mL, 1 mmol, 3.3 equiv.) in MeOH was added. The mixture was stirred for 10 min and purified by flash chromatography on silica gel (DCM/ MeOH 1 : 1 v/v) to give sulfinate salt **3e** (54 mg, 54%) as a white solid.

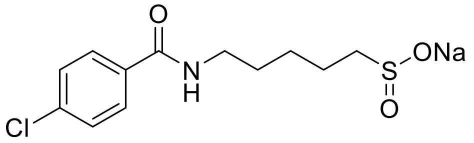

m.p.: >200 °C. – ^{1}H NMR (500 MHz, CD_3OD): 7.84–7.77 (2 H, m), 7.48–7.42 (2 H, m), 3.38 (2 H, t, *J* = 7.1 Hz), 2.33–2.24 (2 H, m), 1.72–1.60 (4 H, m), 1.54–1.43 (2 H, m) ppm. – ^{13}C NMR (125 MHz, CD_3OD): 168.9, 138.5, 134.5, 130.0, 129.6, 63.1, 40.9, 30.3, 27.7, 23.5 ppm. – IR: 2463, 2213, 2136, 2069, 1634, 1458, 1121, 975 cm^{-1}. – HRMS: calcd for $C_{12}H_{15}ClNNa_2O_3S$: 334.0251, found 334.0245 [M+Na$^+$].

Sodium ((1*R,3*S**)-3-acetyl-2,2-dimethylcyclobutyl)methanesulfinate (3f)**

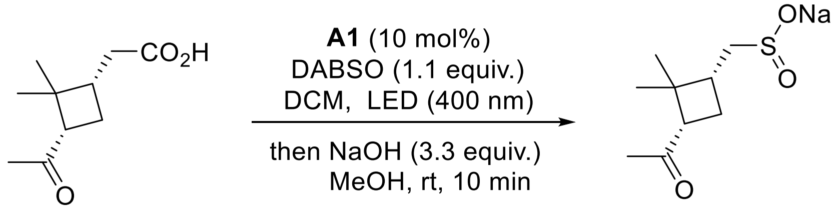

According to **GP3**, the reaction was carried out with *cis*-pinonic acid (55 mg, 0.3 mmol), DABSO (79 mg, 0.33 mmol, 1.1 equiv.), acridine catalyst **A1** (9 mg, 0.03 mmol, 10 mol%) in degassed dichloromethane (6 mL). The test-tube was capped and the reaction mixture was irradiated with LED light (λ = 400 nm) while stirring at room temperature for 12 h. After completion, a 1M solution of sodium hydroxide (1 mL, 1 mmol, 3.3 equiv.) in MeOH was added. The mixture was stirred for 10 min and purified by flash chromatography on silica gel (DCM/ MeOH 3 : 2 v/v) to give sulfinate salt **3f** (43.7 mg, 64%) as a colorless liquid.

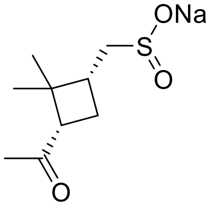

^{1}H NMR (500 MHz, CD_3OD): 3.06–2.92 (1 H, m), 2.34 (2 H, q, *J* = 7.0, 6.4 Hz), 2.21 (1 H, dt, *J* = 12.7, 6.7 Hz), 2.11–1.88 (5 H, m), 1.34 (3 H, s), 0.89 (3 H, s) ppm. – ^{13}C NMR (125 MHz, CD_3OD): 210.7, 64.8, 55.4, 44.8, 37.7, 30.6, 30.2, 24.4, 18.1 ppm. – IR: 3388, 2951, 2496, 1692, 1456, 1369, 1183, 1020, 954 cm^{-1}. – HRMS: calcd for $C_9H_{15}O_3SNa_2$: 249.0532, found 249.0525 [M+Na$^+$].

Sodium 3-(1*H*-indol-2-yl)propane-1-sulfinate (3g)

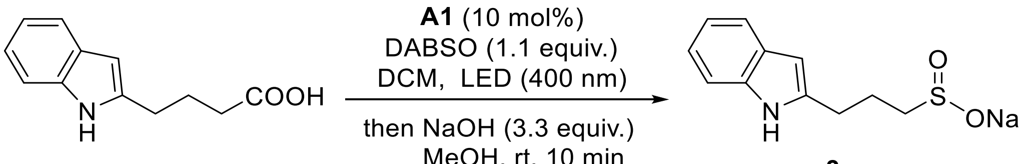

According to **GP3**, the reaction was carried out with 4-(1*H*-indol-2-yl)butanoic acid (61 mg, 0.3 mmol), DABSO (79 mg, 0.33 mmol, 1.1 equiv.), acridine catalyst **A1** (9 mg, 0.03 mmol, 10 mol%) in degassed dichloromethane (6 mL). The test-tube was capped and the reaction mixture was irradiated with LED light (λ = 400 nm) while stirring at room temperature for 12 h. After completion, a 1M solution of sodium hydroxide (1 mL, 1 mmol, 3.3 equiv.) in MeOH was added. The mixture was stirred for 10 min and purified by flash chromatography on silica gel (DCM/ MeOH 7 : 3 v/v) to give the sulfinate salt **3g** (41 mg, 56%) as a white solid.

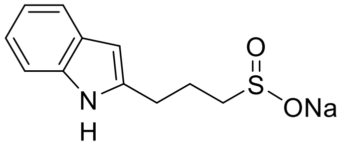

m.p.: >200 °C. – ^{1}H NMR (500 MHz, MeOD): 7.73–7.51 (1 H, m), 7.46–7.28 (1 H, m), 7.03 (3 H, dddd, *J* = 31.8, 14.6, 9.7, 5.0 Hz), 2.96–2.79 (2 H, m), 2.40 (2 H, dt, *J* = 15.5, 8.1 Hz), 2.04 (2 H, hept, *J* = 7.8 Hz) ppm. – ^{13}C NMR (125 MHz, MeOD): 136.8, 127.4, 121.5, 120.7, 118.0, 117.9, 114.5, 110.7, 61.5, 24.3, 23.2 ppm. – IR: 3054, 2986, 2302, 2027, 1546, 1421, 1264, 1082 cm^{-1}. – HRMS: calcd for $C_{11}H_{13}NNaO_2S$: 246.0559, found 246.0554 [M+H$^+$].

Sodium 5-(4-(trifluoromethyl)benzamido)pentane-1-sulfinate (3h)

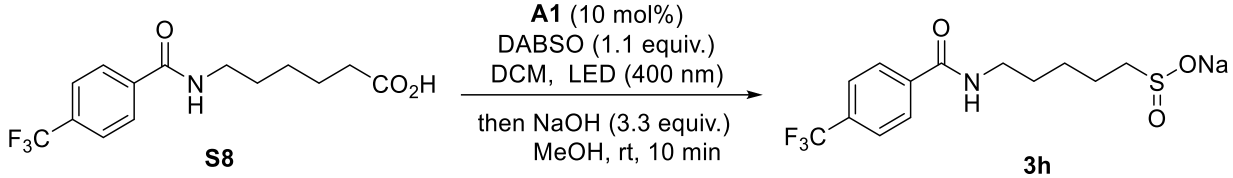

According to **GP3**, the reaction was carried out with acid **S8** (91 mg, 0.3 mmol), DABSO (79 mg, 0.33 mmol, 1.1 equiv.), acridine catalyst **A1** (9 mg, 0.03 mmol, 10 mol%) in degassed dichloromethane (6 mL). The test-tube was capped and the reaction mixture was irradiated with LED light (λ = 400 nm) while stirring at room temperature for 12 h. After completion, a 1M solution of sodium hydroxide (1 mL, 1 mmol, 3.3 equiv.) in MeOH was added. The mixture was stirred for 10 min and purified by flash chromatography on silica gel (DCM/ MeOH 1 : 1 v/v) to give sulfinate salt **3h** (62 mg, 60%) as a white solid.

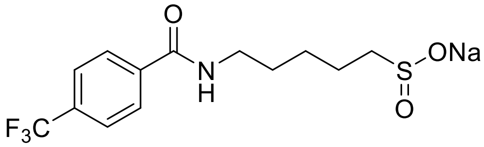

m.p.: >200 °C. – ^{1}H NMR (500 MHz, CD_3OD): 7.99 (2 H, d, J = 8.1 Hz), 7.75 (2 H, d, J = 8.1 Hz), 3.41 (2 H, t, J = 7.1 Hz), 2.30 (2 H, t, J = 7.7 Hz), 1.67 (4 H, p, J = 8.1, 7.5 Hz), 1.51 (2 H, q, J = 8.1 Hz) ppm. – ^{13}C NMR (125 MHz, CD_3OD): 168.7, 139.6, 133.9 (q, $^{2}J_{C\text{-}F}$ = 32.4 Hz), 129.0, 126.4 (q, $^{3}J_{C\text{-}F}$ = 4.0 Hz), 125.3 (q, $^{1}J_{C\text{-}F}$ = 271.3 Hz), 63.0, 41.0, 30.2, 27.7, 23.5 ppm. – ^{19}F NMR (470 MHz, CD_3OD): –64.4 ppm. – IR: 3332, 2944, 2835, 2478, 2217, 2071, 1646, 1457, 1327, 1171, 1120, 1027, 976 cm^{-1}. – HRMS: calcd for $C_{13}H_{17}F_3NO_3S$: 324.0876, found 324.0876 [M-Na+H^{+}].

Sodium tetrahydro-2*H*-pyran-4-sulfinate (3i)

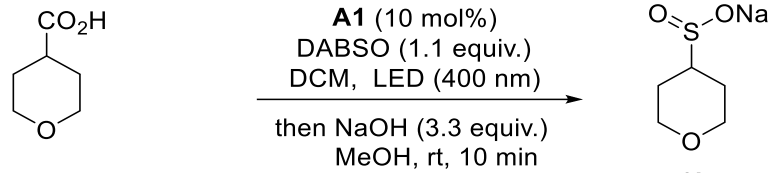

3i

According to **GP3**, the reaction was carried out with tetrahydro-2*H*-pyran-4-carboxylic acid (39 mg, 0.3 mmol), DABSO (79 mg, 0.33 mmol, 1.1 equiv.), acridine catalyst **A1** (9 mg, 0.03 mmol, 10 mol%) in degassed dichloromethane (6 mL). The test-tube was capped and the reaction mixture was irradiated with LED light (λ = 400 nm) while stirring at room temperature for 12 h. After completion, a 1M solution of sodium hydroxide (1 mL, 1 mmol, 3.3 equiv.) in MeOH was added. The mixture was stirred for 10 min and purified by flash chromatography on silica gel (DCM/ MeOH 3 : 2 v/v) to give sulfinate salt **3i** (46.3 mg, 90%) as a white solid.

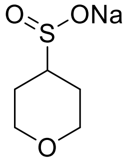

m.p.: >200 °C. – ^{1}H NMR (500 MHz, CD_3OD): 4.01 (2 H, ddd, J = 11.3, 4.7, 2.0 Hz), 3.40 (2 H, td, J = 11.7, 2.3 Hz), 2.04 (1 H, tt, J = 12.1, 4.2 Hz), 1.81 (2 H, ddq, J = 13.0, 4.2, 2.1 Hz), 1.57 (2 H, dtd, J = 13.4, 11.9, 4.7 Hz) ppm. – ^{13}C NMR (125 MHz, CD_3OD): 68.4, 64.4, 26.3 ppm. – IR: 3332, 2944, 2833, 2496, 2071, 1449, 1121, 1028, 980 cm^{-1}. – HRMS: calcd for $C_5H_9O_3SNa_2$: 195.0062, found 195.0062 [M+Na^{+}].

Sodium 1-(*tert*-butoxycarbonyl)piperidine-4-sulfinate (3j)

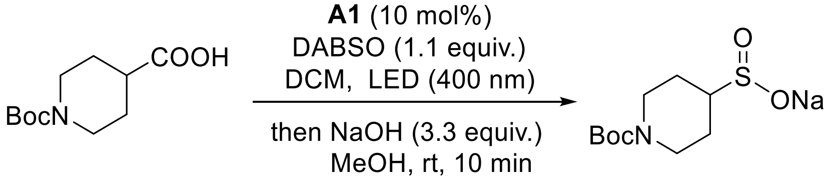

3j

According to **GP3**, the reaction was carried out with 1-(*tert*-butoxycarbonyl)piperidine-4-carboxylic acid (69 mg, 0.3 mmol), DABSO (79 mg, 0.33 mmol, 1.1 equiv.), acridine catalyst **A1** (9 mg, 0.03 mmol, 10 mol%) in degassed dichloromethane (6 mL). The test-tube was capped and the reaction mixture was irradiated with LED light (λ = 400 nm) while stirring at room temperature for 12 h. After completion, a 1M solution of sodium hydroxide (1 mL, 1 mmol, 3.3 equiv.) in MeOH was added. The mixture was stirred for 10 min and purified by flash chromatography on silica gel (DCM/ MeOH 3 : 2 v/v) to give the sulfinate salt **3j** (78 mg, 96%) as a white solid.

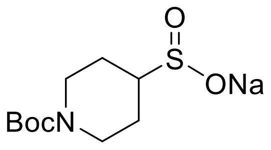

m.p.: >200 °C. – ^{1}H NMR (500 MHz, D_2O): 4.14 (2 H, d, *J* = 13.4 Hz), 2.87 (2 H, t, *J* = 12.7 Hz), 2.19 (1 H, tt, *J* = 12.0, 4.0 Hz), 1.87 (2 H, dd, *J* = 13.4, 3.7 Hz), 1.46 (11 H, m) ppm. – ^{13}C NMR (125 MHz, D_2O): 156.5, 81.7, 63.2, 43.2, 27.7, 23.8 ppm. – IR: 3370, 2976, 2947, 2925, 2853, 1689, 1423, 1394, 1291, 1166, 1111 cm^{-1}. – HRMS: calcd for $C_{10}H_{19}NNaO_4S$: 272.0927, found 272.0925 [M+H$^+$].

Sodium 4,4-difluorocyclohexane-1-sulfinate (3k)

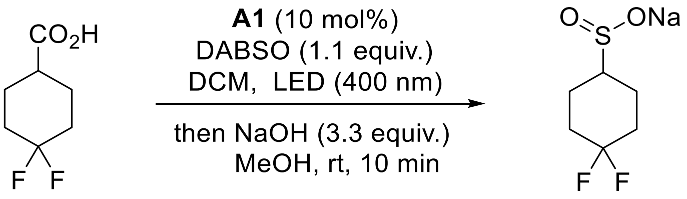

3k

According to **GP3**, the reaction was carried out with 4,4-difluorocyclohexane-1-carboxylic acid (49 mg, 0.3 mmol), DABSO (79 mg, 0.33 mmol, 1.1 equiv.), acridine catalyst **A1** (9 mg, 0.03 mmol, 10 mol%) in degassed dichloromethane (6 mL). The test-tube was capped and the reaction mixture was irradiated with LED light (λ = 400 nm) while stirring at room temperature for 12 h. After completion, a 1M solution of sodium hydroxide (1 mL, 1 mmol, 3.3 equiv.) in MeOH was added. The mixture was stirred for 10 min and purified by flash chromatography on silica gel (DCM/ MeOH 3 : 2 v/v) to give sulfinate salt **3k** (54 mg, 54%) as a white solid.

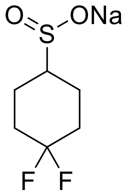

m.p.: >200 °C. – ^{1}H NMR (500 MHz, CD_3OD): 2.16–2.05 (2 H, m), 2.05–1.96 (2 H, m), 1.94–1.70 (3 H, m), 1.69–1.53 (2 H, m) ppm. – ^{13}C NMR (125 MHz, CD_3OD): 124.5(dd, $^{1}J_{C\text{-}F}$ = 240.4, 239.7 Hz), 65.08, 33.90 (d, $^{2}J_{C\text{-}F}$ = 24.5 Hz), 33.71 (d, $^{2}J_{C\text{-}F}$ = 24.3 Hz), 22.72 (d, $^{3}J_{C\text{-}F}$ = 8.9 Hz) ppm. – ^{19}F NMR (470 MHz, CD_3OD): –94.2 (d, *J* = 236.9 Hz), –102.57 (d, *J* = 236.4 Hz) ppm. – IR: 3366, 2912, 2487, 2221, 2137, 2072, 1646, 1451, 1169, 1119, 1043, 974 cm^{-1}. – HRMS: calcd for $C_6H_9F_2O_2S$: 183.0297, found 183.0298 [M^-].

Sodium 2,3-dihydro-1*H*-indene-2-sulfinate (3l)

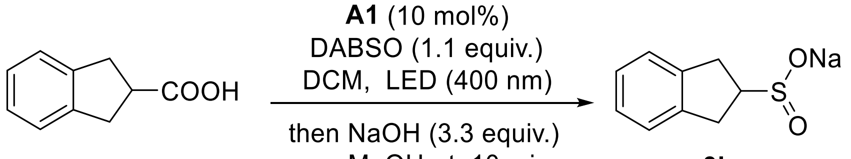

According to **GP3**, the reaction was carried out with 2,3-dihydro-1*H*-indene-2-carboxylic acid (49 mg, 0.3 mmol), DABSO (79 mg, 0.33 mmol, 1.1 equiv.), acridine catalyst **A1** (9 mg, 0.03 mmol, 10 mol%) in degassed dichloromethane (6 mL). The test-tube was capped and the reaction mixture was irradiated with LED light (λ = 400 nm) while stirring at room temperature for 12 h. After completion, a 1M solution of sodium hydroxide (1 mL, 1 mmol, 3.3 equiv.) in MeOH was added. The mixture was stirred for 10 min and purified by flash chromatography on silica gel (DCM/ MeOH 3 : 2 v/v) to give the sulfinate salt **3l** (58 mg, 95%) as a white solid.

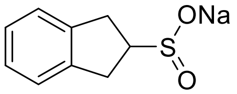

m.p.: >200 °C. – ^{1}H NMR (500 MHz, D_2O): 7.32 (2 H, dt, *J* = 7.3, 3.6 Hz), 7.24 (2 H, dd, *J* = 5.6, 3.2 Hz), 3.29–3.15 (4 H, m), 3.12–3.03 (1 H, m) ppm. – ^{13}C NMR (125 MHz, D_2O): 141.9, 126.7, 124.2, 66.0, 32.0 ppm. – IR: 3338, 2946, 2835, 2483, 1656, 1516, 1449, 1234, 1141, 1022, 977 cm^{-1}. – HRMS: calcd for $C_9H_9Na_2O_2S$: 227.0113, found 227.0115 [$M+Na^+$].

Sodium adamantane-1-sulfinate (3m)

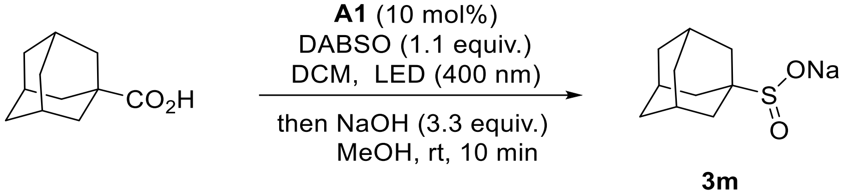

According to **GP3**, the reaction was carried out with 1-adamantanecarboxylic acid (54 mg, 0.3 mmol), DABSO (79 mg, 0.33 mmol, 1.1 equiv.), acridine catalyst **A1** (9 mg, 0.03 mmol, 10 mol%)

in degassed dichloromethane (6 mL). The test-tube was capped and the reaction mixture was irradiated with LED light (λ = 400 nm) while stirring at room temperature for 12 h. After completion, a 1M solution of sodium hydroxide (1 mL, 1 mmol, 3.3 equiv.) in MeOH was added. The mixture was stirred for 10 min and purified by flash chromatography on silica gel (DCM/ MeOH 3 : 2 v/v) to give sulfinate salt **3m** (53 mg, 87%) as a white solid.

Gram scale for compound 3m: According to **GP3**, 5 identical reactions were carried out with 1-adamantanecarboxylic acid (324 mg, 1.8 mmol), DABSO (480 mg, 2 mmol, 1.1 equiv.), acridine catalyst **A1** (53 mg, 0.18 mmol, 10 mol%) in degassed dichloromethane (36 mL). The test-tube was capped, and the reaction mixture was irradiated with LED light (λ = 400 nm) while stirring at room temperature for 12 h. After completion, the reactions were combined, and a 1M solution of sodium hydroxide (30 mL, 30 mmol, 3.3 equiv.) in MeOH was added. The mixture was stirred for 10 min and purified by flash chromatography on silica gel (DCM/ MeOH 3 : 2 v/v) to give sulfinate salt **3m** (1.3 g, 66%) as a white solid.

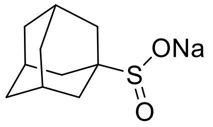

m.p.: >200 °C. – ^{1}H NMR (500 MHz, CD_3OD): 2.11–1.99 (9 H, m), 1.81–1.66 (6 H, m) ppm. – ^{13}C NMR (125 MHz, CD_3OD): 56.9, 37.9, 37.4, 29.9 ppm. – IR: 3420, 2907, 2848, 2529, 1633, 1450, 1341, 1212, 1170, 1112, 1093, 1022 cm^{-1}. – HRMS: calcd for $C_{10}H_{15}O_2S$: 199.0798, found 199.0792 [M$^-$].

Sodium (3*R*)-3-((3*R*,7*R*,8*R*,9*S*,10*S*,13*R*,14*S*,17*R*)-3,7-dihydroxy-10,13-dimethylhexadecahydro-1*H*-cyclopenta[*a*]phenanthren-17-yl)butane-1-sulfinate (3n)

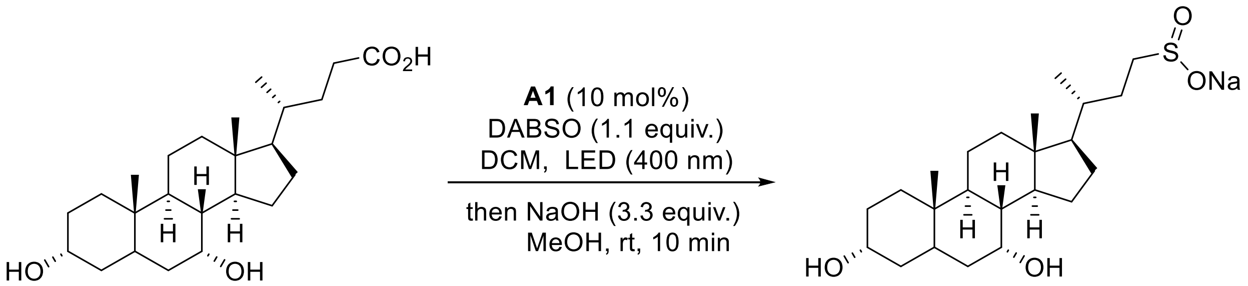

3n

According to **GP3**, the reaction was carried out with chenodeoxycholic acid (118 mg, 0.3 mmol), DABSO (79 mg, 0.33 mmol, 1.1 equiv.), acridine catalyst **A1** (9 mg, 0.03 mmol, 10 mol%) in degassed dichloromethane (6 mL). The test-tube was capped and the reaction mixture was irradiated with LED light (λ = 400 nm) while stirring at room temperature for 12 h. After completion, a 1M solution of sodium hydroxide (1 mL, 1 mmol, 3.3 equiv.) in MeOH was added.

The mixture was stirred for 10 min and purified by flash chromatography on silica gel (DCM/ MeOH 1 : 1 v/v) to give sulfinate salt **3n** (178.6 mg, 76%) as a white solid.

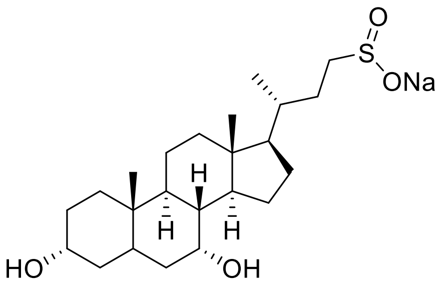

m.p.: >200 °C. – $[\alpha]_D^{23}$ = + 87.2 (c 0.14, MeOH). – ^{1}H NMR (500 MHz, CD_3OD): 3.80 (1 H, q, *J* = 2.8 Hz), 3.41–3.33 (1 H, m), 2.87 (1 H, ddd, *J* = 13.4, 12.1, 4.2 Hz), 2.68 (1 H, ddd, *J* = 13.4, 11.2, 4.6 Hz), 2.27 (1 H, td, *J* = 13.2, 11.4 Hz), 2.06–1.81 (6 H, m), 1.75 (1 H, tdd, *J* = 12.2, 8.0, 3.3 Hz), 1.69–1.45 (8 H, m), 1.42–1.26 (4 H, m), 1.25–1.05 (3 H, m), 1.03–0.94 (4 H, m), 0.93 (3 H, s), 0.70 (3 H, s) ppm. – ^{13}C NMR (125 MHz, CD_3OD): 72.8, 69.0, 57.2, 51.5, 49.9, 43.7, 43.2, 41.0, 40.8, 40.5, 36.6, 36.4, 36.2, 35.9, 34.0, 32.0, 31.3, 29.2, 24.6, 23.4, 21.8, 19.0, 12.2 ppm. – IR: 3302, 2942, 2830, 1449, 1116, 1028 cm^{-1}. – HRMS: calcd for $C_{23}H_{39}O_4S$: 411.2575, found 411.2572 $[M^-]$.

Sodium 1-(6-methoxynaphthalen-2-yl)ethane-1-sulfinate (3o)

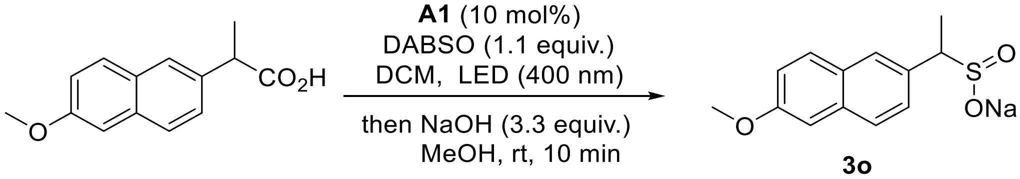

According to **GP3**, the reaction was carried out with naproxen (69 mg, 0.3 mmol), DABSO (79 mg, 0.33 mmol, 1.1 equiv.), acridine catalyst **A1** (9 mg, 0.03 mmol, 10 mol%) in degassed dichloromethane (6 mL). The test-tube was capped and the reaction mixture was irradiated with LED light (λ = 400 nm) while stirring at room temperature for 12 h. After completion, the reaction mixture was quickly extracted with a 0.5M aqueous solution of HCl (1 mL). A 1M solution of sodium hydroxide (1 mL, 1 mmol, 3.3 equiv.) in MeOH was then added to the organic phase. The mixture was stirred for 10 min and purified by flash chromatography on silica gel (DCM/ MeOH 3 : 2 v/v) to give sulfinate salt **3o** (45 mg, 55%) as a white solid.

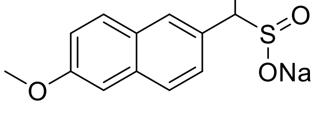

m.p.: >200 °C. – ^{1}H NMR (500 MHz, D_2O): 7.74 (2 H, t, *J* = 9.7 Hz), 7.67 (1 H, s), 7.39 (1 H, d, *J* = 8.3 Hz), 7.23 (1 H, s), 7.12 (1 H, d, *J* = 8.8 Hz), 3.87 (3 H, s), 3.45 (1 H, q, *J* = 7.3 Hz), 1.53 (3 H, d, *J* = 7.2 Hz) ppm. – ^{13}C NMR (125 MHz, D_2O): 156.7, 133.8, 133.4, 129.4, 128.7, 127.5, 126.8, 118.3, 106.1, 68.3, 55.3, 11.8 ppm. – IR: 3389, 2463, 1462, 1204, 1029, 854 cm^{-1}. – HRMS: calcd for C13H13O3S: 249.0591, found 249.0587 $[M^-]$.

Sodium heptane-4-sulfinate (3p)

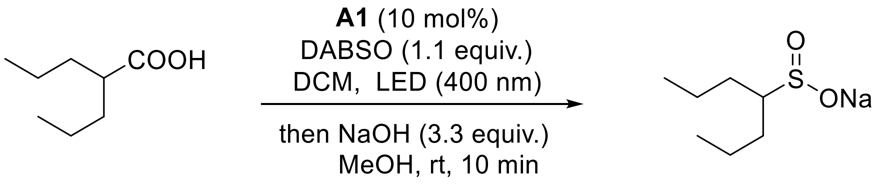

3p

According to **GP3**, the reaction was carried out with 2-propylpentanoic acid (34 mg, 0.3 mmol), DABSO (79 mg, 0.33 mmol, 1.1 equiv.), acridine catalyst **A1** (9 mg, 0.03 mmol, 10 mol%) in degassed dichloromethane (6 mL). The test-tube was capped and the reaction mixture was irradiated with LED light (λ = 400 nm) while stirring at room temperature for 12 h. After completion, a 1M solution of sodium hydroxide (1 mL, 1 mmol, 3.3 equiv.) in MeOH was added. The mixture was stirred for 10 min and purified by flash chromatography on silica gel (DCM/ MeOH 7 : 3 v/v) to give the sulfinate salt **3p** (40 mg, 72%) as a white solid.

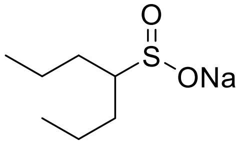

m.p.: >200 °C. – ^{1}H NMR (500 MHz, MeOD): 1.87 (1 H, h, *J* = 5.4 Hz), 1.78–1.65 (2 H, m), 1.44 (6 H, tddd, *J* = 22.0, 17.0, 13.9, 8.6 Hz), 0.94 (6 H, t, *J* = 7.0 Hz) ppm. – ^{13}C NMR (125 MHz, MeOD): 67.9, 30.0, 21.5, 14.8 ppm. – IR: 2459, 2211, 2137, 2069, 1898, 1406, 1122, 1092, 974 cm^{-1}. – HRMS: calcd for $C_7H_{15}O_2S$: 163.0798, found 163.0794 [M$^-$].

Sodium heptane-4-sulfonate (3q)

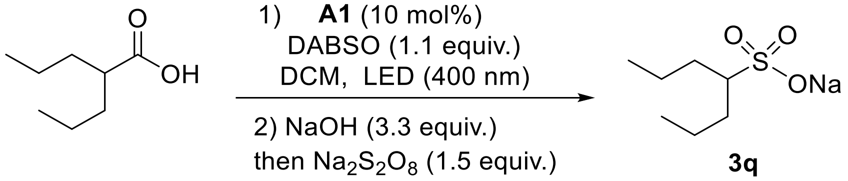

3q

According to **GP3**, the reaction was carried out with valproic acid (43 mg, 0.3 mmol), DABSO (79 mg, 0.33 mmol, 1.1 equiv.), acridine catalyst **A1** (9 mg, 0.03 mmol, 10 mol%) in degassed dichloromethane (6 mL). The test-tube was capped and the reaction mixture was irradiated with LED light (λ = 400 nm) while stirring at room temperature for 12 h. After completion, a 1M aqueous solution of sodium hydroxide (1 mL, 1 mmol, 3.3 equiv.) was then added. The mixture was stirred for 10 min and the organic phase was then removed. Sodium persulfate (107 mg, 0.45 mmol, 1.5 equiv.) was added to the aqueous phase, and the mixture was stirred for 30 min. The reaction was then concentrated and purified by flash chromatography on silica gel (DCM/ MeOH 3 : 2 v/v) to give sulfonate salt **3q** (40 mg, 65%) as a white solid.

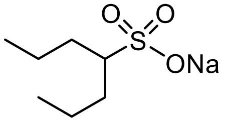

m.p.: >200 °C. – ^{1}H NMR (500 MHz, MeOD): 2.70–2.62 (1 H, m), 1.91 (2 H, dddd, *J* = 13.5, 10.0, 5.1, 2.3 Hz), 1.63–1.43 (5 H, m), 0.96 (6 H, t, *J* = 7.2 Hz) ppm. – ^{13}C NMR (125 MHz, MeOD): 61.1, 33.5, 21.5, 14.5 ppm. – IR: 2943, 2832, 1447, 1216, 1175, 1123, 1026, 1010 cm^{-1}. – HRMS: calcd for $C_7H_{15}O_3S$: 179.0747, found 179.0747 [M$^-$].

Sodium 5-(2,5-dimethylphenoxy)-2-methylpentane-2-sulfinate (3r)

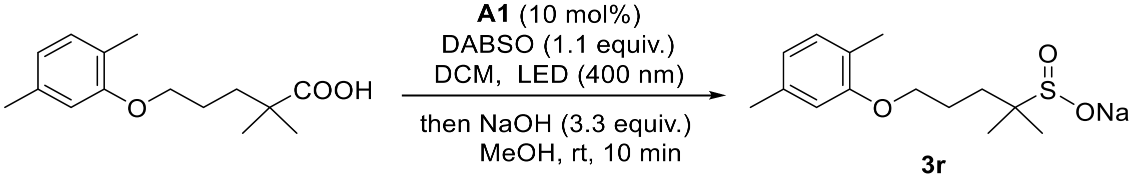

According to **GP3**, the reaction was carried out with 5-(2,5-dimethylphenoxy)-2,2-dimethylpentanoic acid (75 mg, 0.3 mmol), DABSO (79 mg, 0.33 mmol, 1.1 equiv.), acridine catalyst **A1** (9 mg, 0.03 mmol, 10 mol%) in degassed dichloromethane (6 mL). The test-tube was capped and the reaction mixture was irradiated with LED light (λ = 400 nm) while stirring at room temperature for 12 h. After completion, a 1M solution of sodium hydroxide (1 mL, 1 mmol, 3.3 equiv.) in MeOH was added. The mixture was stirred for 10 min and purified by flash chromatography on silica gel (DCM/ MeOH 7 : 3 v/v) to give the sulfinate salt **3r** (58 mg, 66%) as a white solid.

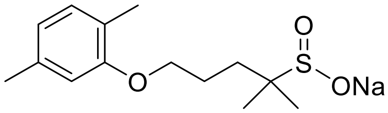

m.p.: >200 °C. – ^{1}H NMR (500 MHz, MeOD): 6.92 (1 H, d, *J* = 7.3 Hz), 6.64 (1 H, s), 6.63–6.43 (1 H, m), 3.92 (2 H, t, *J* = 5.9 Hz), 2.25 (3 H, s), 2.11 (3 H, s), 2.00–1.74 (4 H, m), 1.32 (6 H, s) ppm. – ^{13}C NMR (125 MHz, MeOD): 158.3, 137.5, 131.1, 124.4, 121.7, 113.0, 69.4, 58.3, 35.1, 25.8, 22.9, 21.4, 16.0 ppm. – IR: 3053, 2987, 2323, 2166, 2009, 1421, 1264, 896 cm^{-1}. – HRMS: calcd for $C_{14}H_{21}O_3S$: 269.1217, found 269.1212 [M$^-$].

Go back to table of contents

Sulfonyl Chlorides and Sulfonyl Fluorides

2-(4-Fluorophenyl)ethane-1-sulfonyl chloride (4a)

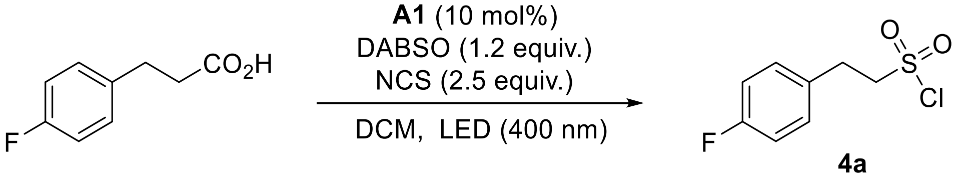

4a

According to **GP4**, the reaction was carried out with 3-(4-fluorophenyl)propanoic acid (50 mg, 0.3 mmol), DABSO (87 mg, 0.36 mmol, 1.2 equiv.), acridine catalyst **A1** (9 mg, 0.03 mmol, 10 mol%), *N*-chlorosuccinimide (100 mg, 0.75 mmol, 2.5 equiv.) in degassed dichloromethane (6 mL). The test-tube was capped and the reaction mixture was irradiated with LED light (λ = 400 nm) while stirring at room temperature for 8 h. The reaction mixture was concentrated under reduced pressure, and the remaining material was purified by flash chromatography on silica gel (hexane/ethyl acetate 3 : 2 v/v) to give the sulfonyl chloride **4a** (48 mg, 73%) as a colorless oil.

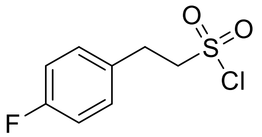

^{1}H NMR (500 MHz, $CDCl_3$): 7.24–7.17 (2 H, m), 7.11–6.96 (2 H, m), 3.95–3.80 (2 H, m), 3.46–3.22 (2 H, m) ppm. – ^{13}C NMR (125 MHz, $CDCl_3$): 162.3 (d, $^1J_{C\text{-}F}$ = 246.5 Hz), 131.4 (d, $^4J_{C\text{-}F}$ = 3.6 Hz), 130.3 (d, $^3J_{C\text{-}F}$ = 8.2 Hz), 116.3 (d, $^2J_{C\text{-}F}$ = 21.6 Hz), 66.3, 29.9 ppm. – ^{19}F NMR (470 MHz, $CDCl_3$): –114.5 (ddd, J = 13.8, 8.7, 5.2 Hz) ppm. – IR: 2935, 2860, 2323, 1510, 1265, 1125, 896 cm^{-1}. – HRMS: calcd for $C_8H_8O_2FSCl$: 221.9918, found 221.9918 [M$^+$].

4-Oxo-4-phenylbutane-1-sulfonyl chloride (4b)

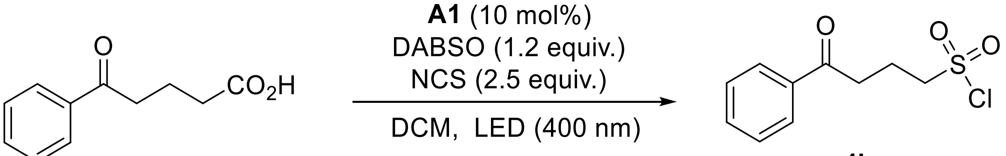

4b

According to **GP4**, the reaction was carried out with 5-oxo-5-phenylpentanoic acid (58 mg, 0.3 mmol), DABSO (87 mg, 0.36 mmol, 1.2 equiv.), acridine catalyst **A1** (9 mg, 0.03 mmol, 10 mol%), *N*-chlorosuccinimide (100 mg, 0.75 mmol, 2.5 equiv.) in degassed dichloromethane (6 mL). The test-tube was capped and the reaction mixture was irradiated with LED light (λ = 400 nm) while stirring at room temperature for 8 h. The reaction mixture was concentrated under reduced pressure, and the remaining material was purified by flash chromatography on silica gel (hexane/ethyl acetate 3 : 2 v/v) to give the sulfonyl chloride **4b** (46 mg, 62%) as a white solid.

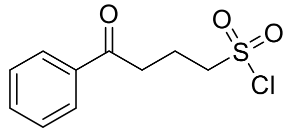

m.p.: 90–92 °C. – ^{1}H NMR (500 MHz, $CDCl_3$): 7.96 (2 H, dd, *J* = 8.4, 1.4 Hz, 2H), 7.67–7.56 (1 H, m), 7.49 (2 H, t, *J* = 7.8 Hz), 3.88 (2 H, t, *J* = 7.2 Hz), 3.30 (2 H, t, *J* = 6.5 Hz), 2.50 (2 H, p, *J* = 6.8 Hz) ppm. – ^{13}C NMR (125 MHz, $CDCl_3$): 197.9, 136.4, 133.8, 129.0, 128.1, 64.7, 35.5, 19.1 ppm. – IR: 1672, 1589, 1438, 1417, 1358, 1263, 1187, 1052 cm^{-1}. – HRMS: calcd for $C_{10}H_{11}ClO_3SNa$: 269.0010, found 269.0012 [M+H$^+$].

tert-Butyl (4-(chlorosulfonyl)butyl)carbamate (4c)

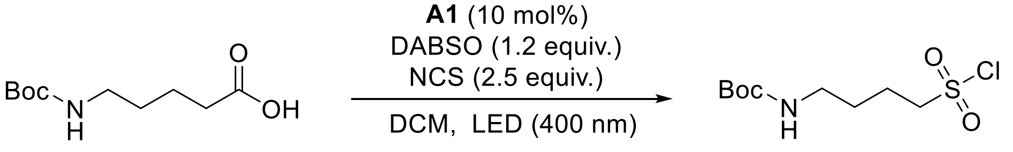

4c

According to **GP4**, the reaction was carried out with 5-((*tert*-butoxycarbonyl)amino)pentanoic acid (65 mg, 0.3 mmol), DABSO (87 mg, 0.36 mmol, 1.2 equiv.), acridine catalyst **A1** (9 mg, 0.03 mmol, 10 mol%), *N*-chlorosuccinimide (100 mg, 0.75 mmol, 2.5 equiv.) in degassed dichloromethane (6 mL). The test-tube was capped and the reaction mixture was irradiated with LED light (λ = 400 nm) while stirring at room temperature for 8 h. The reaction mixture was concentrated under reduced pressure, and the remaining material was purified by flash chromatography on silica gel (hexane/ ethyl acetate 4 : 1 v/v) to give the sulfonyl chloride **4c** (61 mg, 72%) as a colorless oil.

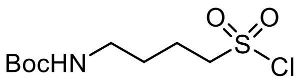

^{1}H NMR (500 MHz, $CDCl_3$): 4.61 (1 H, s), 3.73 (2 H, t, *J* = 7.7 Hz), 3.19 (2 H, q, *J* = 6.6 Hz), 2.14–2.01 (2 H, m), 1.70 (2 H, quint., *J* = 7.0 Hz), 1.44 (9 H, s) ppm. – ^{13}C NMR (125 MHz, $CDCl_3$): 156.0, 79.6, 64.9, 39.3, 28.4, 28.2, 21.6 ppm. – IR: 2975, 2930, 2875, 2854, 1720, 1472, 1400, 1381, 1205, 1189, 1128, 892, 774 cm^{-1}. – HRMS: calcd for $C_9H_{18}ClNaNO_4S$: 294.0537, found 294.0537 [M+Na$^+$].

tert-Butyl (5-(chlorosulfonyl)pentyl)carbamate (4d)

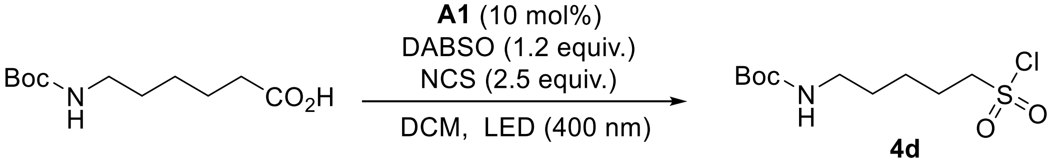

4d

According to **GP4**, the reaction was carried out with 6-((*tert*-butoxycarbonyl)amino)hexanoic acid (69 mg, 0.3 mmol), DABSO (87 mg, 0.36 mmol, 1.2 equiv.), acridine catalyst **A1** (9 mg, 0.03 mmol, 10 mol%), *N*-chlorosuccinimide (100 mg, 0.75 mmol, 2.5 equiv.) in degassed dichloromethane (6

Go back to table of contents

mL). The test-tube was capped and the reaction mixture was irradiated with LED light (λ = 400 nm) while stirring at room temperature for 8 h. The reaction mixture was concentrated under reduced pressure, and the remaining material was purified by flash chromatography on silica gel (hexane/ ethyl acetate 4 : 1 v/v) to give the sulfonyl chloride **4d** (61 mg, 72%) as a colorless oil.

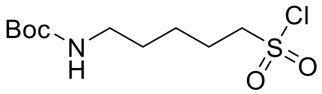

^{1}H NMR (500 MHz, $CDCl_3$): 4.55 (1 H, brs), 3.72–3.60 (2 H, m), 3.14 (2 H, q, *J* = 6.3 Hz), 2.13–1.98 (2 H, m), 1.64–1.48 (4 H, m), 1.44 (9 H, s) ppm. – ^{13}C NMR (125 MHz, $CDCl_3$): 156.1, 79.5, 65.4, 40.2, 29.7, 28.6, 25.0, 24.2 ppm. – IR: IR: 2972, 2922, 2863, 2851, 1458, 1380, 1375, 1208, 1180 cm^{-1}. – HRMS: calcd for $C_{10}H_{20}ClNO_4SNa$: 308.0694, found 308.0698 [$M+Na^+$].

Pentadecane-1-sulfonyl chloride (4e)

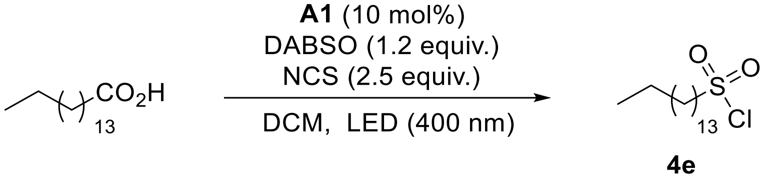

4e

According to **GP4**, the reaction was carried out with palmitic acid (77 mg, 0.3 mmol), DABSO (87 mg, 0.36 mmol, 1.2 equiv.), acridine catalyst **A1** (9 mg, 0.03 mmol, 10 mol%), *N*-chlorosuccinimide (100 mg, 0.75 mmol, 2.5 equiv.) in degassed dichloromethane (6 mL). The test-tube was capped and the reaction mixture was irradiated with LED light (λ = 400 nm) while stirring at room temperature for 8 h. The reaction mixture was concentrated under reduced pressure, and the remaining material was purified by flash chromatography on silica gel (hexane/ ethyl acetate 9 : 1 v/v) to give the sulfonyl chloride **4e** (53 mg, 57%) as a colorless oil.

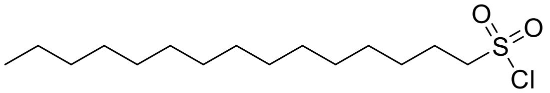

^{1}H NMR (500 MHz, $CDCl_3$): 3.70–3.62 (2 H, m), 2.10–1.97 (2 H, m), 1.49 (2 H, p, *J* = 7.7, 7.2 Hz), 1.40–1.17 (22 H, m), 0.88 (3 H, t, *J* = 6.9 Hz) ppm. – ^{13}C NMR (125 MHz, $CDCl_3$): 65.7, 32.1, 29.82, 29.80, 29.76, 29.7, 29.6, 29.5, 29.3, 29.0, 27.7, 24.4, 22.8, 14.3 ppm. – IR: 2992, 1727, 1362, 1236, 1055, 915 cm^{-1}. – HRMS: calcd for $C_{15}H_{32}O_2SCl$: 309.1655, found 309.1663 [$M+H^+$].

Heptadecane-1-sulfonyl chloride (4f)

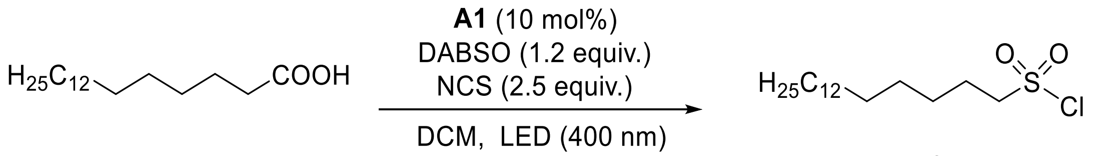

According to **GP4**, the reaction was carried out with stearic acid (85 mg, 0.3 mmol), DABSO (87 mg, 0.36 mmol, 1.2 equiv.), acridine catalyst **A1** (9 mg, 0.03 mmol, 10 mol%), *N*-chlorosuccinimide (100 mg, 0.75 mmol, 2.5 equiv.) in degassed dichloromethane (6 mL). The test-tube was capped and the reaction mixture was irradiated with LED light (λ = 400 nm) while stirring at room temperature for 8 h. The reaction mixture was concentrated under reduced pressure, and the remaining material was purified by flash chromatography on silica gel (hexane/ ethyl acetate 19 : 1 v/v) to give the sulfonyl chloride **4f** (63 mg, 62%) as a colorless oil.

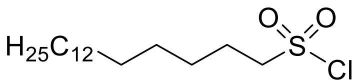

^{1}H NMR (500 MHz, $CDCl_3$): 3.76–3.61 (2 H, m), 2.11–1.96 (2 H, m), 1.62–1.45 (2 H, m), 1.29 (26 H, d, *J* = 10.6 Hz), 0.90 (3 H, t, *J* = 6.8 Hz) ppm. – ^{13}C NMR (125 MHz, $CDCl_3$): 65.6, 32.1, 29.8, 29.8, 29.8, 29.7, 29.6, 29.6, 29.5, 29.3, 29.0, 27.7, 24.4, 22.8, 14.3 ppm. – IR: 2984, 1735, 1373, 1243, 1047, 911, 733 cm^{-1}. – HRMS: calcd for $C_{17}H_{34}ClO_2S$: 337.1968, found 337.1962 [M–H]$^+$.

Heptane-4-sulfonyl chloride (4g)

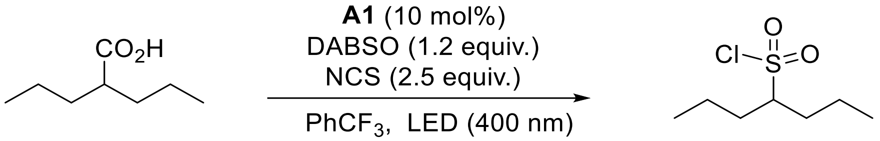

According to **GP4**, the reaction was carried out with 2-propylpentanoic acid (43 mg, 0.3 mmol), DABSO (108 mg, 0.45 mmol, 1.5 equiv.), acridine catalyst **A1** (9 mg, 0.03 mmol, 10 mol%), *N*-chlorosuccinimide (100 mg, 0.75 mmol, 2.5 equiv.) in degassed benzotrifluoride (6 mL). The test-tube was capped and the reaction mixture was irradiated with LED light (λ = 400 nm) while stirring at room temperature for 8 h. The reaction mixture was concentrated under reduced pressure, and the remaining material was purified by flash chromatography on silica gel (hexane/ ethyl acetate 4 : 1 v/v) to give the sulfonyl chloride **4g** (33 mg, 56%) as a colorless oil.

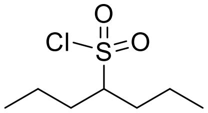

^{1}H NMR (500 MHz, $CDCl_3$): 3.47 (1 H, p, *J* = 6.1 Hz), 2.10 (2 H, ddt, *J* = 15.1, 10.9, 5.8 Hz), 1.82 (2 H, dq, *J* = 14.6, 7.1 Hz), 1.55 (4 H, dt, *J* = 15.4, 8.0 Hz), 0.99 (6 H, t, *J* = 7.5 Hz) ppm. – ^{13}C NMR (125 MHz, $CDCl_3$): 76.5, 32.0, 20.0, 14.0 ppm. – IR:

2941, 2902, 2884, 1475, 1396, 1312, 1174, 842 cm^{-1}. – HRMS: calcd for $C_7H_{16}O_2SCl$: 199.0560, found 199.0560 [M$^+$].

Tetrahydro-2*H*-pyran-4-sulfonyl chloride (4h)

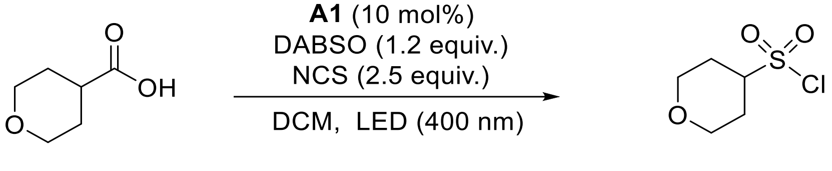

4h

According to **GP4**, the reaction was carried out with tetrahydro-2*H*-pyran-4-carboxylic acid (39 mg, 0.3 mmol), DABSO (87 mg, 0.36 mmol, 1.2 equiv.), acridine catalyst **A1** (9 mg, 0.03 mmol, 10 mol%), *N*-chlorosuccinimide (100 mg, 0.75 mmol, 2.5 equiv.) in degassed dichloromethane (6 mL). The test-tube was capped and the reaction mixture was irradiated with LED light (λ = 400 nm) while stirring at room temperature for 8 h. The reaction mixture was concentrated under reduced pressure, and the remaining material was purified by flash chromatography on silica gel (hexane/ethyl acetate 4 : 1 v/v) to give the sulfonyl chloride **4h** (38 mg, 69%) as a colorless oil.

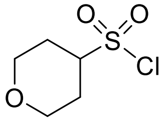

^{1}H NMR (500 MHz, $CDCl_3$): 4.19–4.14 (2 H, m), 3.75 (1 H, tt, *J* = 11.9, 3.9 Hz), 3.44 (2 H, td, *J* = 11.9, 2.2 Hz), 2.26 (1 H, ddd, *J* = 12.8, 4.2, 2.1 Hz), 2.11 (2 H, dtd, *J* = 13.2, 11.8, 4.8 Hz) ppm. – ^{13}C NMR (125 MHz, $CDCl_3$): 71.5, 66.3, 27.4 ppm. – IR: 2986, 2917, 2858, 1115, 1070, 1050, 953, 931, 910, 803, 773, 713 cm^{-1}. – HRMS: calcd for $C_5H_{10}O_3S$: 185.0039, found 185.0040 [M+H$^+$].

2,3-Dihydro-1*H*-indene-2-sulfonyl chloride (4i)

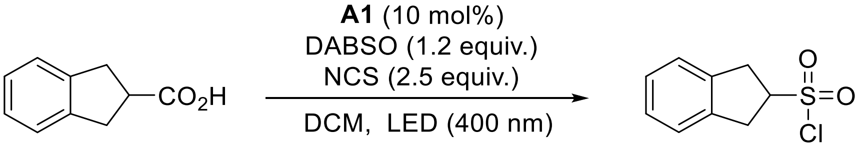

4i

According to **GP4**, the reaction was carried out with 2,3-dihydro-1*H*-indene-2-carboxylic acid (49 mg, 0.3 mmol), DABSO (87 mg, 0.36 mmol, 1.2 equiv.), acridine catalyst **A1** (9 mg, 0.03 mmol, 10 mol%), *N*-chlorosuccinimide (100 mg, 0.75 mmol, 2.5 equiv.) in degassed dichloromethane (6 mL). The test-tube was capped and the reaction mixture was irradiated with LED light (λ = 400 nm) while stirring at room temperature for 8 h. The reaction mixture was concentrated under reduced

pressure, and the remaining material was purified by flash chromatography on silica gel (hexane/ ethyl acetate 4 : 1 v/v) to give the sulfonyl chloride **4i** (38 mg, 59%) as a colorless oil.

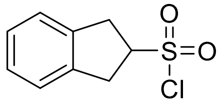

^{1}H NMR (500 MHz, $CDCl_3$): 7.28–7.23 (4 H, m), 4.56 (1 H, tt, *J* = 9.0, 6.7 Hz), 3.69 (2 H, dd, *J* = 17.1, 6.7 Hz), 3.57 (2 H, dd, *J* = 17.1, 9.0 Hz) ppm. – ^{13}C NMR (125 MHz, $CDCl_3$): 138.4, 127.9, 124.7, 73.2, 35.4 ppm. – IR: 3062, 3013, 2961, 2910, 2842, 1742, 1654, 1420, 1414, 1370, 1306, 1275, 1216, 1120, 1016 cm^{-1}. – HRMS: calcd for $C_9H_9O_2SCl$: 216.0012, found 216.0007 [M$^+$].

((1*S**,3*R**)-3-Acetyl-2,2-dimethylcyclobutyl)methanesulfonyl chloride (4j)

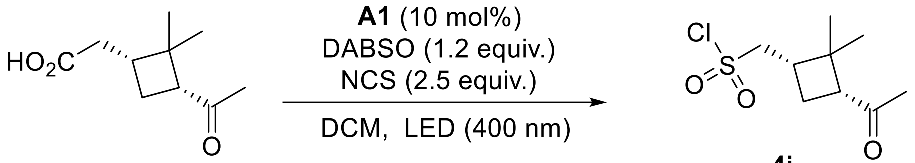

4j

According to **GP4**, the reaction was carried out with *cis*-pinonic acid (55 mg, 0.3 mmol), DABSO (87 mg, 0.36 mmol, 1.2 equiv.), acridine catalyst **A1** (9 mg, 0.03 mmol, 10 mol%), *N*-chlorosuccinimide (100 mg, 0.75 mmol, 2.5 equiv.) in degassed dichloromethane (6 mL). The test-tube was capped and the reaction mixture was irradiated with LED light (λ = 400 nm) while stirring at room temperature for 8 h. The reaction mixture was concentrated under reduced pressure, and the remaining material was purified by flash chromatography on silica gel (hexane/ ethyl acetate 4 : 1 v/v) to give the sulfonyl chloride **4j** (52 mg, 73%) as a colorless oil.

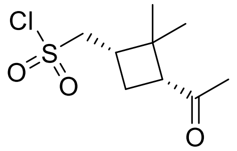

^{1}H NMR (500 MHz, $CDCl_3$): 3.72 (1 H, dd, *J* = 14.0, 6.4 Hz), 3.64 (1 H, dd, *J* = 14.0, 7.7 Hz), 2.98 (1 H, dd, *J* = 10.2, 7.5 Hz), 2.70 (1 H, dtd, *J* = 10.4, 8.0, 6.4 Hz), 2.25 (1 H, dt, *J* = 11.6, 10.5 Hz), 2.13 (1 H, dt, *J* = 11.7, 7.9 Hz), 2.07 (3 H, s), 1.40 (3 H, s), 0.95 (3 H, s) ppm. – ^{13}C NMR (125 MHz, $CDCl_3$): 206.4, 66.5, 54.0, 43.9, 36.9, 30.4, 29.9, 23.2, 17.6 ppm. – IR: 3040, 2950, 2924, 2867, 2257, 2136, 1700, 1351, 1184, 1150, 715 cm^{-1}. – HRMS: calcd for $C_9H_{15}ClO_3S$: 239.0503, found 239.0507 [M+H$^+$].

(3*R*,7*R*,8*R*,9*S*,10*S*,13*R*,14*S*,17*R*)-17-((*R*)-4-(Chlorosulfonyl)butan-2-yl)-10,13-dimethylhexadecahydro-1*H*-cyclopenta[*a*]phenanthrene-3,7-diyl diacetate (4k)

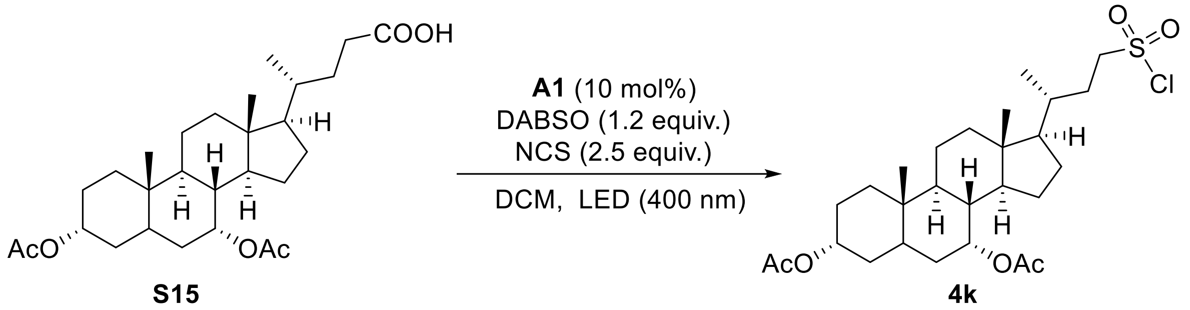

According to **GP4**, the reaction was carried out with acid **S15** (71 mg, 0.15 mmol), DABSO (44 mg, 0.18 mmol, 1.2 equiv.), acridine catalyst **A1** (5 mg, 0.015 mmol, 10 mol%), *N*-chlorosuccinimide (50 mg, 0.375 mmol, 2.5 equiv.) in degassed dichloromethane (3 mL). The test-tube was capped and the reaction mixture was irradiated with LED light (λ = 400 nm) while stirring at room temperature for 8 h. The reaction mixture was concentrated under reduced pressure, and the remaining material was purified by flash chromatography on silica gel (hexane/ ethyl acetate 9 : 1 v/v) to give the sulfonyl chloride **4k** (40 mg, 50%) as a colorless oil.

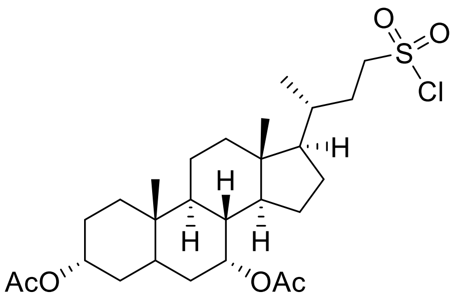

$[\alpha]_D^{23}$ = + 70.5. – ^{1}H NMR (500 MHz, $CDCl_3$): 4.88 (1 H, t, *J* = 3.2 Hz), 4.59 (1 H, tt, *J* = 11.4, 4.5 Hz), 3.64 (2 H, dddd, *J* = 63.6, 13.8, 11.4, 4.8 Hz), 2.17 (3 H, s), 2.04 (6 H, d, *J* = 13.4 Hz), 1.99–1.77 (3 H, m), 1.77–0.81 (24 H, m), 0.67 (3 H, s) ppm. – ^{13}C NMR (125 MHz, $CDCl_3$): 170.8, 170.5, 74.3, 71.3, 63.3, 55.4, 50.5, 43.0, 41.1, 39.6, 38.0, 35.0, 34.9, 34.8, 34.7, 34.2, 31.4, 30.3, 28.2, 26.9, 23.6, 22.8, 21.7, 21.6, 20.8, 18.6, 11.9 ppm. – IR: 2950, 2102, 1720, 1682, 1543, 1432, 1265, 1035, 891 cm^{-1}. – HRMS: calcd for $C_{27}H_{43}ClO_6S$: 548.2807, found 548.2820 [M+H$^+$].

4-Oxo-4-phenylbutane-1-sulfonyl fluoride (5a)

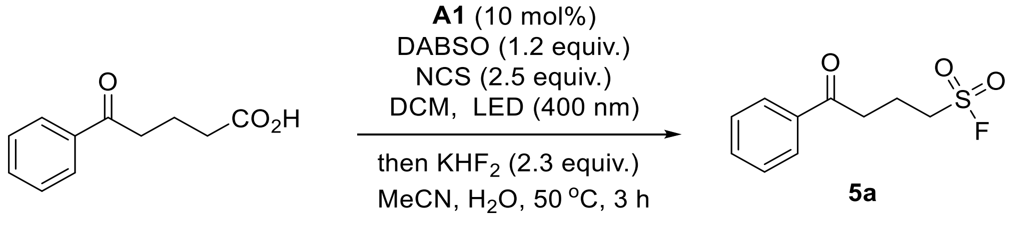

According to **GP5**, the reaction was carried out with 5-oxo-5-phenylpentanoic acid (58 mg, 0.3 mmol), DABSO (87 mg, 0.36 mmol, 1.2 equiv.), acridine catalyst **A1** (9 mg, 0.03 mmol, 10 mol%),

N-chlorosuccinimide (100 mg, 0.75 mmol, 2.5 equiv.), in degassed dichloromethane (6 mL). The test-tube was capped and the reaction mixture was irradiated with LED light (λ = 400 nm) while stirring at room temperature for 8 h. After completion, the reaction mixture was concentrated under reduced pressure and acetonitrile (2 mL), as well as a 2M aqueous solution of potassium bifluoride (0.35 mL, 0.69 mmol, 2.3 equiv.), were added. The reaction was stirred at 50 °C for 3 h before quenching with a saturated solution of potassium hydrogen sulfate (4 mL) and extracting with ethyl acetate (3 × 10 mL). The organic layer was combined, dried over anhydrous sodium sulfate, concentrated under reduced pressure, and purified by flash chromatography on silica gel (hexane/ ethyl acetate 3 : 2 v/v) to give the sulfonyl fluoride **5a** (69 mg, 99%) as a white solid.

Five-minute fluorination: According to **GP5**, the reaction was carried out with 5-oxo-5-phenylpentanoic acid (58 mg, 0.3 mmol), DABSO (87 mg, 0.36 mmol, 1.2 equiv.), acridine catalyst **A1** (9 mg, 0.03 mmol, 10 mol%), *N*-chlorosuccinimide (100 mg, 0.75 mmol, 2.5 equiv.), in degassed dichloromethane (6 mL). The test-tube was capped and the reaction mixture was irradiated with LED light (λ = 400 nm) while stirring at room temperature for 8 h. After completion, the reaction mixture was concentrated under reduced pressure and acetonitrile (2 mL), as well as a 2M aqueous solution of potassium bifluoride (0.35 mL, 0.69 mmol, 2.3 equiv.), were added. The reaction was stirred at 80 °C for 5 min before quenching with a saturated solution of potassium hydrogen sulfate (4 mL) and extracting with ethyl acetate (3 × 10 mL). The organic layer was combined, dried over anhydrous sodium sulfate, concentrated under reduced pressure, and purified by flash chromatography on silica gel (hexane/ ethyl acetate 3 : 2 v/v) to give the sulfonyl fluoride **5a** (59 mg, 86%) as a white solid.

Gram scale for compound 5a: According to **GP5**, 4 identical the reactions were carried out with 5-oxo-5-phenylpentanoic acid (270 mg, 1.4 mmol), DABSO (403 mg, 1.68 mmol, 1.2 equiv.), acridine catalyst **A1** (41 mg, 0.14 mmol, 10 mol%), *N*-chlorosuccinimide (466 mg, 3.5 mmol, 2.5 equiv.), in degassed dichloromethane (28 mL). The test-tube was capped and the reaction mixture was irradiated with LED light (λ = 400 nm) while stirring at room temperature for 8 h. After completion, the reaction mixture was concentrated under reduced pressure and acetonitrile (9 mL), as well as a 2M aqueous solution of potassium bifluoride (1.6 mL, 3.2 mmol, 2.3 equiv.), were added. The reaction was stirred at 50 °C for 3 h before quenching with a saturated solution of potassium hydrogen sulfate (20 mL) and extracting with ethyl acetate (3 × 40 mL). The organic layer was combined, dried over anhydrous sodium sulfate, concentrated under reduced pressure, and purified by flash chromatography on silica gel (hexane/ ethyl acetate 3 : 2 v/v) to give the sulfonyl fluoride **5a** (1.2 g, 66%) as a white solid.

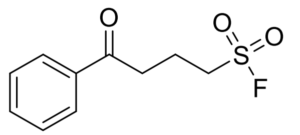

m.p.: 87–89 °C. – ^{1}H NMR (500 MHz, $CDCl_3$): 7.98–7.94 (2 H, m), 7.63–7.57 (1 H, m), 7.49 (2 H, t, J = 7.7 Hz), 3.58 (2 H, td, J = 7.2, 4.4 Hz), 3.26 (2 H, t, J = 6.5 Hz), 2.41 (2 H, p, J = 6.9 Hz) ppm. – ^{13}C NMR (125 MHz, $CDCl_3$): 197.9, 136.4, 133.8, 129.0, 128.1, 50.23, 50.16 (d, J = 16.5 Hz), 35.5, 18.1 ppm. – ^{19}F NMR (470 MHz, $CDCl_3$): 54.0 ppm. – IR: 1679, 1595, 1449, 1409, 1384, 1264, 1193, 1068 cm^{-1}. – HRMS: calcd for $C_{10}H_{11}O_3FS$: 230.0413, found 230.0421 [M^+].

Methyl 5-(fluorosulfonyl)pentanoate (5b)

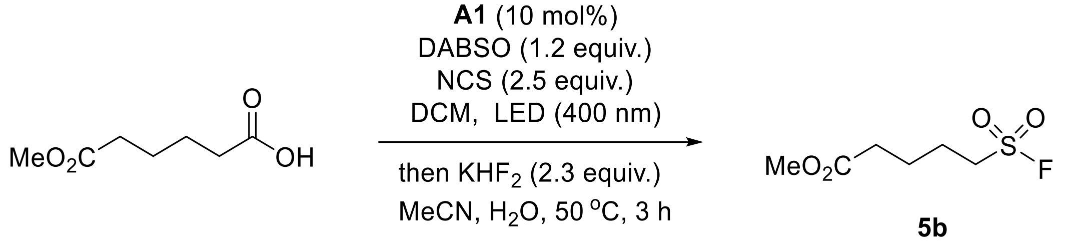

5b

According to **GP5**, the reaction was carried out with 6-methoxy-6-oxohexanoic acid (48 mg, 0.3 mmol), DABSO (87 mg, 0.36 mmol, 1.2 equiv.), acridine catalyst **A1** (9 mg, 0.03 mmol, 10 mol%), *N*-chlorosuccinimide (100 mg, 0.75 mmol, 2.5 equiv.) in degassed dichloromethane (6 mL). The test-tube was capped and the reaction mixture was irradiated with LED light (λ = 400 nm) while stirring at room temperature for 8 h. After completion, the reaction mixture was concentrated under reduced pressure and acetonitrile (2 mL), as well as a 2M aqueous solution of potassium bifluoride (0.35 mL, 0.69 mmol, 2.3 equiv.), were added. The reaction was stirred at 50 °C for 3 h before quenching with a saturated solution of potassium hydrogen sulfate (4 mL) and extracting with ethyl acetate (3 × 10 mL). The organic layer was combined, dried over anhydrous sodium sulfate, concentrated under reduced pressure, and purified by flash chromatography on silica gel (hexane/ ethyl acetate 19 : 1 v/v) to give the sulfonyl fluoride **5b** (30 mg, 51%) as a colorless oil.

Tricomponent synthesis of compound 5b:

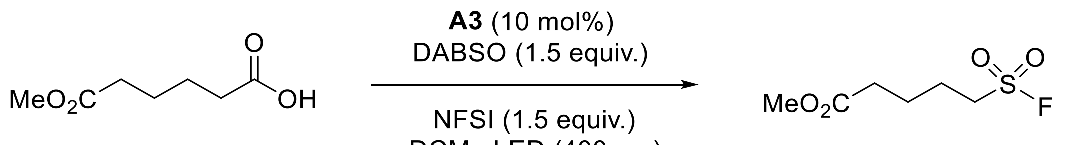

84

According to **GP7**, the reaction was carried out with 6-methoxy-6-oxohexanoic acid (48 mg, 0.3 mmol), DABSO (108 mg, 0.45 mmol, 1.5 equiv.), acridine catalyst **A3** (9 mg, 0.03 mmol, 10 mol%), *N*-fluorobenzenesulfonimide (142 mg, 0.45 mmol, 1.5 equiv.), and degassed dichloromethane (6 mL). The test-tube was capped and the reaction mixture was irradiated with LED light (λ = 400

nm) while stirring at room temperature for 12 h. The reaction mixture was concentrated under reduced pressure, and the remaining material was purified by flash chromatography on silica gel (hexane/ ethyl acetate 9 : 1 v/v) to give the sulfonyl chloride **5b** (31 mg, 53%) as a colorless oil.

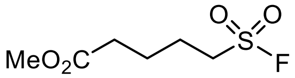

^{1}H NMR (300 MHz, $CDCl_3$): 3.68 (3 H, s), 3.45–3.33 (2 H, m), 2.39 (2 H, t, *J* = 7.1 Hz), 2.07–1.93 (2 H, m), 1.88–1.72 (2 H, m) ppm. – ^{13}C NMR (75 MHz, $CDCl_3$): 173.0, 51.9, 50.7 (d, $^{2}J_{C-F}$ = 17.1 Hz), 33.1, 23.2, 23.1 ppm. – ^{19}F NMR (470 MHz, $CDCl_3$): 53.6 (s) ppm. – IR: 2954, 2925, 2857, 1732, 1471, 1400, 1248, 1195, 1248, 1195, 891, 825 cm^{-1}. – HRMS: calcd for $C_6H_{12}O_4FS$: 199.0440 found 199.0443 [M+H$^+$].

Pentadecane-1-sulfonyl fluoride (5c)

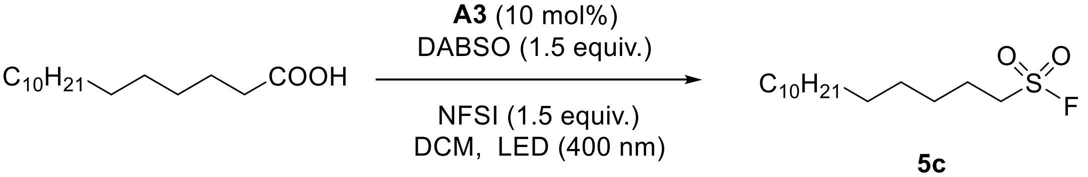

5c

According to **GP7**, the reaction was carried out with palmitic acid (77 mg, 0.3 mmol), DABSO (108 mg, 0.45 mmol, 1.5 equiv.), acridine catalyst **A3** (9 mg, 0.03 mmol, 10 mol%), *N*-fluorobenzenesulfonimide (142 mg, 0.45 mmol, 1.5 equiv.), and degassed dichloromethane (6 mL). The test-tube was capped and the reaction mixture was irradiated with LED light (*λ* = 400 nm) while stirring at room temperature for 12 h. The reaction mixture was concentrated under reduced pressure, and the remaining material was purified by flash chromatography on silica gel (hexane/ ethyl acetate 49 : 1 v/v) to give the sulfonyl chloride **5c** (53 mg, 60%) as a green yellow solid.

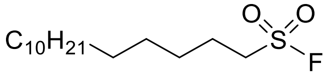

m.p.: 35–37 °C.–^{1}H NMR (300 MHz, $CDCl_3$): 3.39–3.30 (2 H, m), 1.94 (2 H, tt, *J* = 7.8, 6.5 Hz), 1.47 (2 H, quint., *J* = 7.1 Hz), 1.39– 1.13 (22H, m), 0.91–0.83 (3 H, m) ppm. – ^{13}C NMR (75 MHz, $CDCl_3$): 51.0 (d, $^{2}J_{C-F}$ = 16.0 Hz), 32.1, 29.8, 29.7, 29.6, 29.5, 29.3, 28.9, 28.0, 23.5, 22.8, 14.2 ppm. – ^{19}F NMR (470 MHz, $CDCl_3$): 53.2 (s) ppm. – IR: 2952, 2916, 2847, 1465, 1386, 1302, 1196, 844, 813, 759, 737, 720 cm^{-1}. – HRMS: calcd for $C_{15}H_{30}O_2SF$: 293.1951, found 293.1947 [M–H$^+$].

Heptadecane-1-sulfonyl fluoride (5d)

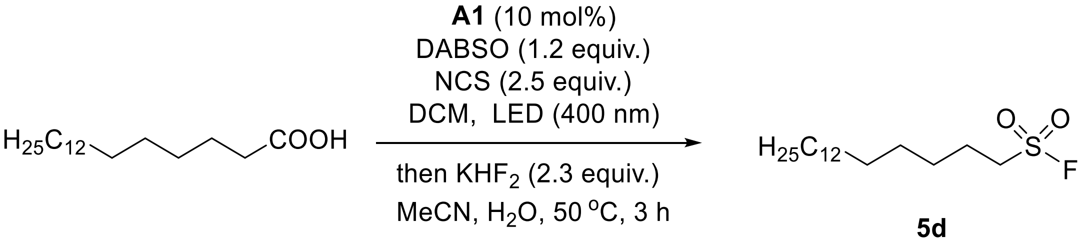

According to **GP5**, the reaction was carried out with stearic acid (85 mg, 0.3 mmol), DABSO (87 mg, 0.36 mmol, 1.2 equiv.), acridine catalyst **A1** (9 mg, 0.03 mmol, 10 mol%), *N*-chlorosuccinimide (100 mg, 0.75 mmol, 2.5 equiv.) in degassed dichloromethane (6 mL). The test-tube was capped and the reaction mixture was irradiated with LED light (λ = 400 nm) while stirring at room temperature for 8 h. After completion, the reaction mixture was concentrated under reduced pressure and acetonitrile (2 mL), as well as a 2M aqueous solution of potassium bifluoride (0.35 mL, 0.69 mmol, 2.3 equiv.), were added. The reaction was stirred at 50 °C for 3 h before quenching with a saturated solution of potassium hydrogen sulfate (4 mL) and extracting with ethyl acetate (3 × 10 mL). The organic layer was combined, dried over anhydrous sodium sulfate, concentrated under reduced pressure, and purified by flash chromatography on silica gel (hexane/ ethyl acetate 19 : 1 v/v) to give the sulfonyl fluoride **5d** (65 mg, 67%) as a green yellow solid.

Tricomponent synthesis of compound 5d:

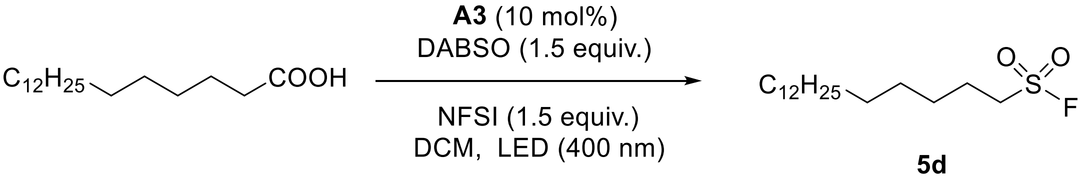

According to **GP7**, the reaction was carried out with stearic acid (77 mg, 0.3 mmol), DABSO (108 mg, 0.45 mmol, 1.5 equiv.), acridine catalyst **A3** (9 mg, 0.03 mmol, 10 mol%), *N*-fluorobenzenesulfonimide (142 mg, 0.45 mmol, 1.5 equiv.), and degassed dichloromethane (6 mL). The test-tube was capped and the reaction mixture was irradiated with LED light (λ = 400 nm) while stirring at room temperature for 12 h. The reaction mixture was concentrated under reduced pressure, and the remaining material was purified by flash chromatography on silica gel (hexane/ ethyl acetate 19 : 1 v/v) to give the sulfonyl chloride **5d** (65 mg, 68%) as a green yellow solid.

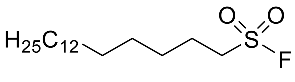

m.p.: 32–35 °C. – ^{1}H NMR (500 MHz, CDCl$_3$): 3.44–3.27 (2 H, m), 2.00–1.86 (2 H, m), 1.48 (2 H, quint., *J* = 7.3 Hz), 1.38–1.19 (26 H, m), 0.88 (3 H, t, *J* = 6.9 Hz) ppm. – ^{13}C NMR (125 MHz, CDCl$_3$): 51.05 (d, $^{2}J_{C\text{-}F}$ = 16.2 Hz), 51.1, 51.0, 32.1, 29.8, 29.8,

Go back to table of contents

29.8, 29.7, 29.6, 29.5, 29.3, 29.0, 28.0, 23.5, 22.8, 14.3 ppm. – ^{19}F NMR (470 MHz, $CDCl_3$): 43.95 ppm. – IR: 2980, 2953, 2925, 2862, 1714, 1453, 1403, 1380, 1301, 1202, 1188, 1130, 1113, 965, 940, 862, 808 cm^{-1}. – HRMS: calcd for $C_{17}H_{34}O_2SF$: 321.2264, found 321.2261 [M–H$^+$].

2-(4-Fluorophenyl)ethane-1-sulfonyl fluoride (5e)

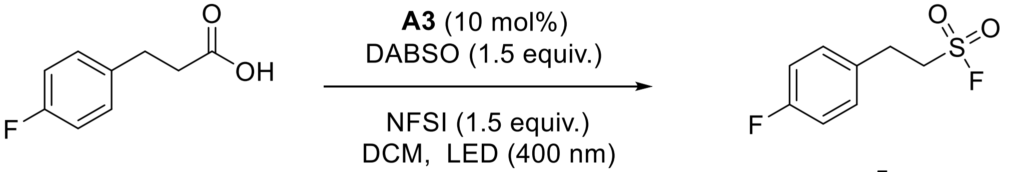

5e

According to **GP7**, the reaction was carried out with 3-(4-fluorophenyl)propanoic acid (50 mg, 0.3 mmol), DABSO (108 mg, 0.45 mmol, 1.5 equiv.), acridine catalyst **A3** (9 mg, 0.03 mmol, 10 mol%), *N*-fluorobenzenesulfonimide (142 mg, 0.45 mmol, 1.5 equiv.), and degassed dichloromethane (6 mL). The test-tube was capped and the reaction mixture was irradiated with LED light (λ = 400 nm) while stirring at room temperature for 12 h. The reaction mixture was concentrated under reduced pressure, and the remaining material was purified by flash chromatography on silica gel (hexane/ ethyl acetate 19 : 1 v/v) to give the sulfonyl chloride **5e** (37 mg, 60%) as a colorless oil.

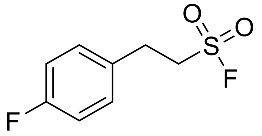

^{1}H NMR (300 MHz, $CDCl_3$): 7.25–7.15 (2 H, m), 7.10–6.99 (2 H, m), 3.66–3.53 (2 H, m), 3.26–3.18 (2 H, m) ppm. – ^{13}C NMR (75 MHz, $CDCl_3$): 162.3 (d, $^1J_{C-F}$ = 246.6 Hz), 131.8 (d, $^4J_{C-F}$ = 3.7 Hz), 130.1 (d, $^3J_{C-F}$ = 8.2 Hz), 116.2 (d, $^3J_{C-F}$ = 21.8 Hz), 52.3 (d, $^2J_{C-F}$ = 15.6 Hz), 29.0 ppm. –^{19}F NMR (470 MHz, $CDCl_3$): 54.0 ppm. – ^{19}F NMR (470 MHz, $CDCl_3$): 53.67 (t, *J* = 4.1 Hz), –114.63 (tt, *J* = 8.9, 5.4 Hz) ppm. – IR: 3035, 2964, 2933, 2841, 1735, 1511, 1418, 1402, 1258, 1227, 1194, 1165, 1016, 892, 861, 838 cm^{-1}. – HRMS: calcd for $C_8H_8O_2F_2S$: 206.0213, found 206.0213 [M$^+$].

Pentadecane-7-sulfonyl fluoride (5f)

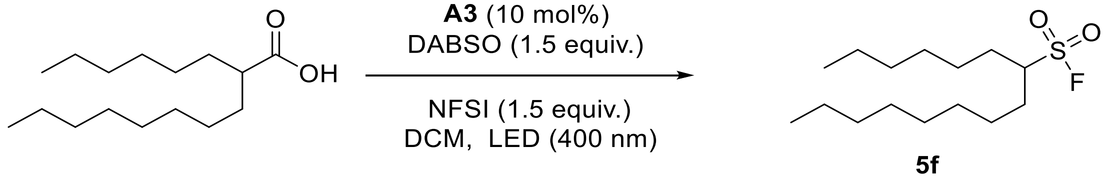

5f

According to **GP7**, the reaction was carried out with 2-hexyldecanoic acid (77 mg, 0.3 mmol), DABSO (108 mg, 0.45 mmol, 1.5 equiv.), acridine catalyst **A3** (9 mg, 0.03 mmol, 10 mol%), *N*-fluorobenzenesulfonimide (142 mg, 0.45 mmol, 1.5 equiv.), and degassed dichloromethane (6 mL). The test-tube was capped and the reaction mixture was irradiated with LED light (λ = 400

nm) while stirring at room temperature for 12 h. The reaction mixture was concentrated under reduced pressure, and the remaining material was purified by flash chromatography on silica gel (hexane/ ethyl acetate 49 : 1 v/v) to give the sulfonyl chloride **5f** (69 mg, 78%) as a colorless oil.

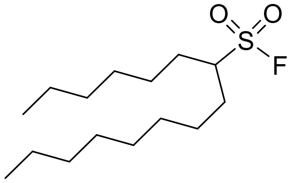

^{1}H NMR (300 MHz, CDCl$_3$): 3.30 (1 H, pd, *J* = 6.2, 1.9 Hz), 2.07–1.90 (2 H, m), 1.88–1.70 (2 H, m), 1.60–1.40 (4 H, m), 1.40–1.22 (16 H, m), 0.88 (6 H, td, *J* = 6.8, 3.0 Hz) ppm. – ^{13}C NMR (75 MHz, CDCl$_3$): 63.0 (d, $^{2}J_{C\text{-}F}$ = 10.2 Hz), 31.9, 31.5, 29.4, 29.3, 29.1, 29.0, 26.3, 22.8, 22.6, 14.2, 14.1 ppm. – ^{19}F NMR (470 MHz, CDCl$_3$): 46.91 (d, *J* = 42.9 Hz) ppm. – IR: 2961, 2925, 2872, 2856, 1722, 1473, 1408, 1384, 1206, 1188, 1118, 893, 775, 681 cm^{-1}. – HRMS: calcd for $C_{15}H_{30}O_2FS$: 293.1951, found 293.1963 [M-H$^+$].

Cyclohexanesulfonyl fluoride (5g)

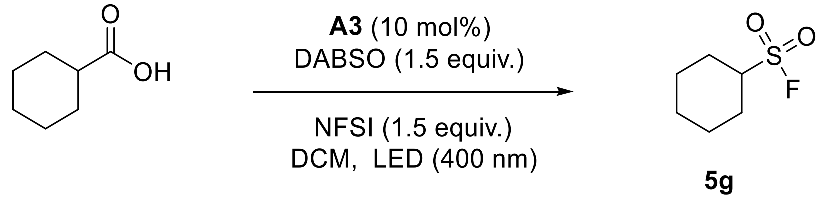

5g

According to **GP7**, the reaction was carried out with cyclohexanecarboxylic acid (38 mg, 0.3 mmol), DABSO (108 mg, 0.45 mmol, 1.5 equiv.), acridine catalyst **A3** (9 mg, 0.03 mmol, 10 mol%), *N*-fluorobenzenesulfonimide (142 mg, 0.45 mmol, 1.5 equiv.), and degassed dichloromethane (6 mL). The test-tube was capped and the reaction mixture was irradiated with LED light (λ = 400 nm) while stirring at room temperature for 12 h. The reaction mixture was concentrated under reduced pressure, and the remaining material was purified by flash chromatography on silica gel (hexane/ ethyl acetate 49 : 1 v/v) to give the sulfonyl chloride **5g** (32 mg, 64%) as a colorless oil.

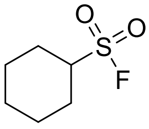

^{1}H NMR (500 MHz, CDCl$_3$): 3.30 (1 H, ttd, *J* = 12.2, 3.6, 1.5 Hz), 2.32–2.24 (2 H, m), 1.95 (2 H, dt, *J* = 13.0, 4.0 Hz), 1.80–1.61 (3 H, m), 1.40–1.19 (3 H, m) ppm. – ^{13}C NMR (125 MHz, CDCl$_3$): 61.1 (d, $^{2}J_{C\text{-}F}$ = 12.6 Hz), 26.6, 24.9, 24.8 ppm. – ^{19}F NMR (470 MHz, CDCl$_3$): 39.09 (s) ppm. – IR: 2972, 2941. 2917, 2863, 1740, 1689, 1454, 1399, 1335, 1238, 1199, 1184, 1016, 827, 715 m^{-1}. – HRMS: calcd for $C_6H_{10}O_2FS$: 165.0386, found 165.0388 [M-H$^+$].

Cycloheptanesulfonyl fluoride (5h)

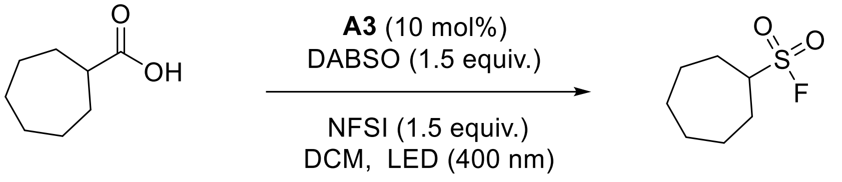

5h

According to **GP7**, the reaction was carried out with cycloheptanecarboxylic acid (43 mg, 0.3 mmol), DABSO (108 mg, 0.45 mmol, 1.5 equiv.), acridine catalyst **A3** (9 mg, 0.03 mmol, 10 mol%), *N*-fluorobenzenesulfonimide (142 mg, 0.45 mmol, 1.5 equiv.), and degassed dichloromethane (6 mL). The test-tube was capped and the reaction mixture was irradiated with LED light (λ = 400 nm) while stirring at room temperature for 12 h. The reaction mixture was concentrated under reduced pressure, and the remaining material was purified by flash chromatography on silica gel (hexane/ ethyl acetate 49 : 1 v/v) to give the sulfonyl chloride **5h** (35 mg, 68%) as a colorless oil.

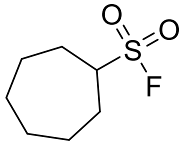

^{1}H NMR (500 MHz, $CDCl_3$): 3.44 (1 H, ttd, *J* = 9.6, 4.6, 2.6 Hz), 2.34 (2 H, ddd, *J* = 16.2, 8.5, 4.3 Hz), 2.00–1.80 (4 H, m), 1.65–1.50 (6 H, m) ppm. – ^{13}C NMR (125 MHz, $CDCl_3$): 63.0 (d, $^2J_{C-F}$ = 10.4 Hz), 28.3, 28.2, 25.5 ppm. – ^{19}F NMR (470 MHz, $CDCl_3$): 39.87 ppm. – IR: 3008, 2970, 2938, 2862, 1748, 1720, 1473, 1397, 1366, 1225, 1196, 817, 793, 775 m^{-1}. – HRMS: calcd for $C_7H_{14}FO_2S$: 181.0699, found 181.070 [M+H$^+$].

((3a*S*,5a*R*,8a*R*,8b*S*)-2,2,7,7-Tetramethyltetrahydro-3a*H*-bis([1,3]dioxolo)[4,5-*b*:4',5'-*d*]pyran-3a-yl)methyl 4-(fluorosulfonyl)butanoate (5i)

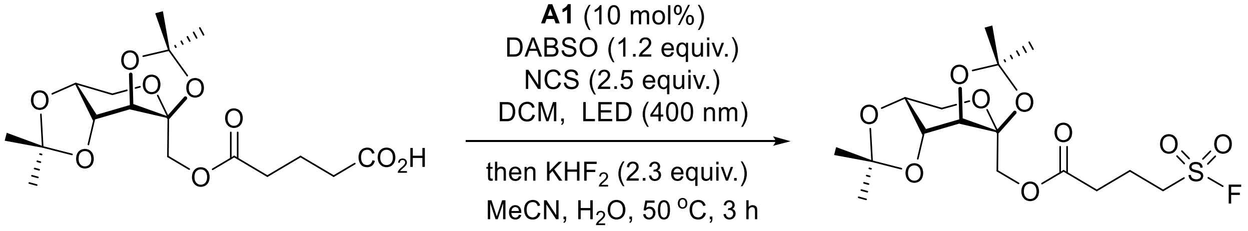

S16 **5i**

According to **GP5**, the reaction was carried out with acid **S16** (112 mg, 0.3 mmol), DABSO (87 mg, 0.36 mmol, 1.2 equiv.), acridine catalyst **A1** (9 mg, 0.03 mmol, 10 mol%), *N*-chlorosuccinimide (100 mg, 0.75 mmol, 2.5 equiv.) in degassed dichloromethane (6 mL). The test-tube was capped and the reaction mixture was irradiated with LED light (λ = 400 nm) while stirring at room temperature for 8 h. After completion, the reaction mixture was concentrated under reduced pressure and acetonitrile (2 mL), as well as a 2M aqueous solution of potassium bifluoride (0.35 mL, 0.69 mmol, 2.3 equiv.), were added. The reaction was stirred at 50 °C for 3 h before quenching

with a saturated solution of potassium hydrogen sulfate (4 mL) and extracting with ethyl acetate (3 × 10 mL). The organic layer was combined, dried over anhydrous sodium sulfate, concentrated under reduced pressure, and purified by flash chromatography on silica gel (hexane/ ethyl acetate 3 : 2 v/v) to give the sulfonyl fluoride **5i** (93 mg, 76%) as a colorless oil.

Five-minute fluorination: According to **GP5**, the reaction was carried out with acid **S16** (112 mg, 0.3 mmol), DABSO (87 mg, 0.36 mmol, 1.2 equiv.), acridine catalyst **A1** (9 mg, 0.03 mmol, 10 mol%), *N*-chlorosuccinimide (100 mg, 0.75 mmol, 2.5 equiv.) in degassed dichloromethane (6 mL). The test-tube was capped and the reaction mixture was irradiated with LED light (λ = 400 nm) while stirring at room temperature for 8 h. After completion, the reaction mixture was concentrated under reduced pressure and acetonitrile (2 mL), as well as a 2M aqueous solution of potassium bifluoride (0.35 mL, 0.69 mmol, 2.3 equiv.), were added. The reaction was stirred at 80 °C for 5 min before quenching with a saturated solution of potassium hydrogen sulfate (4 mL) and extracting with ethyl acetate (3 × 10 mL). The organic layer was combined, dried over anhydrous sodium sulfate, concentrated under reduced pressure, and purified by flash chromatography on silica gel (hexane/ ethyl acetate 3 : 2 v/v) to give the sulfonyl fluoride **5i** (87 mg, 71%) as a colorless oil.

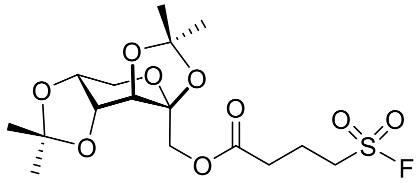

$[\alpha]_D^{23}$ = –13 (c 0.08, $CHCl_3$). – ^{1}H NMR (500 MHz, $CDCl_3$): 4.60 (1 H, dd, *J* = 7.9, 2.6 Hz), 4.45 (1 H, d, *J* = 11.6 Hz), 4.29–4.21 (2 H, m), 4.07 (1 H, d, *J* = 11.6 Hz), 3.89 (1 H, dd, *J* = 13.0, 1.9 Hz), 3.76 (1 H, dd, *J* = 12.9, 0.8 Hz), 3.56–3.49 (2 H, m), 2.62 (2H, t, *J* = 6.8 Hz), 2.27 (2 H, p, *J* = 7.0 Hz), 1.54 (3 H, s), 1.48 (3 H, s), 1.38 (3 H, s), 1.34 (3 H, s) ppm. – ^{13}C NMR (125 MHz, $CDCl_3$): 171.2, 109.3, 109.0, 101.5, 70.9, 70.1, 66.1, 61.4, 49.9, 49.7, 31.4, 26.6, 26.0, 25.3, 24.2, 19.0 ppm. – ^{19}F NMR (470 MHz, $CDCl_3$): 53.8 ppm. – IR: 2989, 2937, 1741, 1452, 1403, 1383, 1266, 1252, 1206, 1165, 1146, 1103, 1071, 1019 cm^{-1}. – HRMS: calcd for $C_{16}H_{25}FO_9SNa$: 435.1096, found 435.1094 [M+Na$^+$].

Heptane-4-sulfonyl fluoride (5j)

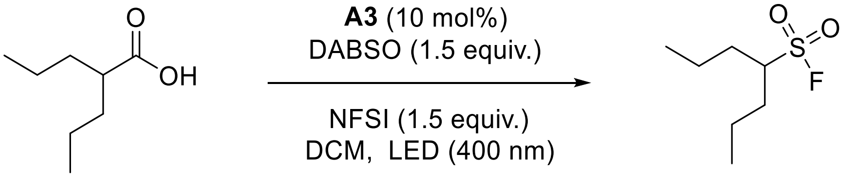

5j

According to **GP7**, the reaction was carried out with valproic acid (43 mg, 0.3 mmol), DABSO (108 mg, 0.45 mmol, 1.5 equiv.), acridine catalyst **A3** (9 mg, 0.03 mmol, 10 mol%), *N*-

fluorobenzenesulfonimide (142 mg, 0.45 mmol, 1.5 equiv.), and degassed dichloromethane (6 mL). The test-tube was capped and the reaction mixture was irradiated with LED light (λ = 400 nm) while stirring at room temperature for 12 h. The reaction mixture was concentrated under reduced pressure, and the remaining material was purified by flash chromatography on silica gel (hexane/ ethyl acetate 49 : 1 v/v) to give the sulfonyl chloride **5j** (34 mg, 62%) as a colorless oil.

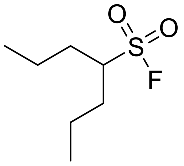

^{1}H NMR (500 MHz, $CDCl_3$): 3.37–3.25 (1 H, m), 2.03–1.89 (2 H, m), 1.78 (2 H, ddtd, *J* = 14.3, 9.9, 6.5, 1.7 Hz), 1.60–1.47 (4 H, m), 0.98 (6 H, t, *J* = 7.3 Hz) ppm. – ^{13}C NMR (125 MHz, $CDCl_3$): 62.6 (d, $^2J_{C\text{-}F}$ = 10.4 Hz), 31.1, 19.7, 13.9 ppm. – ^{19}F NMR (470 MHz, $CDCl_3$): 47.42 ppm. – IR: 2974, 2885, 2255, 1737, 1471, 1405, 1365, 1264, 1216, 1107, 905, 729 cm^{-1}. – HRMS: calcd for $C_7H_{14}O_2FS$: 181.0699, found 181.0696 [M-H$^+$].

(3*R*,5*R*,8*R*,9*S*,10*S*,12*S*,13*R*,14*S*,17*R*)-17-((*R*)-4-(Fluorosulfonyl)butan-2-yl)-10,13-dimethylhexadecahydro-1*H*-cyclopenta[*a*]phenanthrene-3,12-diyl diacetate (5k)

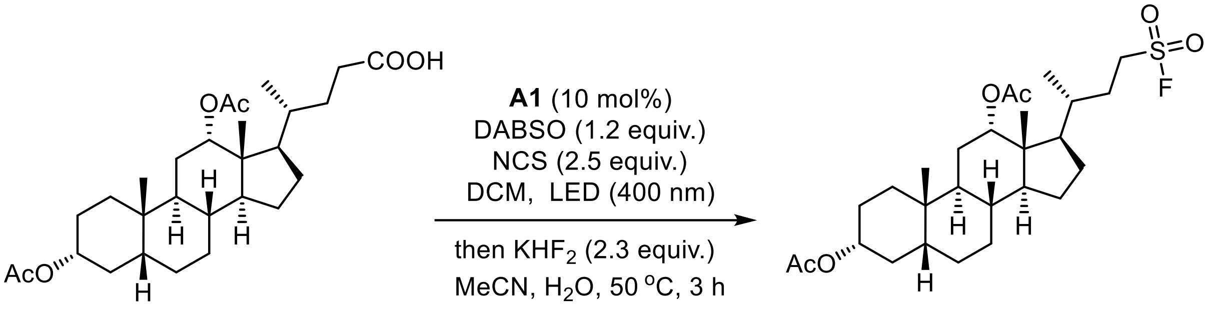

S17 **5k**

According to **GP7**, the reaction was carried out with acid **S17** (118 mg, 0.3 mmol), DABSO (108 mg, 0.45 mmol, 1.5 equiv.), acridine catalyst **A3** (9 mg, 0.03 mmol, 10 mol%), *N*-fluorobenzenesulfonimide (142 mg, 0.45 mmol, 1.5 equiv.), and degassed dichloromethane (6 mL). The test-tube was capped and the reaction mixture was irradiated with LED light (λ = 400 nm) while stirring at room temperature for 12 h. The reaction mixture was concentrated under reduced pressure, and the remaining material was purified by flash chromatography on silica gel (hexane/ ethyl acetate 4 : 1 v/v) to give the sulfonyl fluoride **5k** (84 mg, 55%) as a colorless solid.

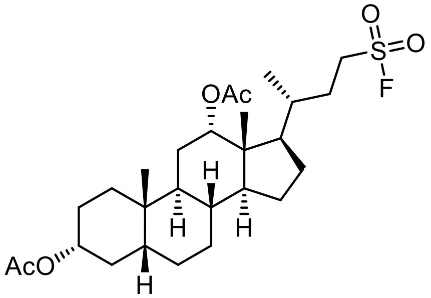

m.p.: 55–58°C. – $[\alpha]_D^{23}$ = + 35.6 (c 0.13, $CHCl_3$). – ^1H NMR (500 MHz, $CDCl_3$): 5.07 (1 H, t, *J* = 2.9 Hz), 4.70 (1 H, tt, *J* = 11.3, 4.6 Hz), 3.39 (1 H, ddt, *J* = 15.4, 11.4, 4.3 Hz), 3.24 (1 H, dddd, *J* = 14.5, 10.9, 5.3, 3.7 Hz), 2.07 (7 H, d, *J* = 37.9 Hz), 1.94–1.75 (3 H, m), 1.74–1.52 (10 H, m), 1.51–1.39 (4 H, m), 1.38–0.94 (6 H, m), 0.93–0.82 (6 H, m), 0.74 (3 H, s) ppm. – ^{13}C NMR (125 MHz, $CDCl_3$): 170.7, 170.5, 75.8, 74.3, 49.5, 48.7 (d, *J* = 16.3 Hz), 47.5, 45.3, 41.9, 35.8, 34.9, 34.5, 34.21, 34.18, 32.4, 29.3, 27.5, 27.0, 26.8, 26.0, 25.8, 23.5, 23.2, 21.6, 21.5, 17.6, 12.6 ppm. – ^{19}F NMR (470 MHz, $CDCl_3$): 52.26 ppm. – IR: 2938, 2868, 1727, 1448, 1400, 1379, 1362, 1240, 1195, 1161, 1089, 1026, 971 cm^{-1}. – HRMS: calcd for $C_{27}H_{43}FO_6SNa$: 537.2657, found 537.2653 [M+Na$^+$].

(3*R*,7*R*,8*R*,9*S*,10*S*,13*R*,14*S*,17*R*)-17-((*R*)-4-(Fluorosulfonyl)butan-2-yl)-10,13-dimethylhexadecahydro-1*H*-cyclopenta[*a*]phenanthrene-3,7-diyl diacetate (5l)

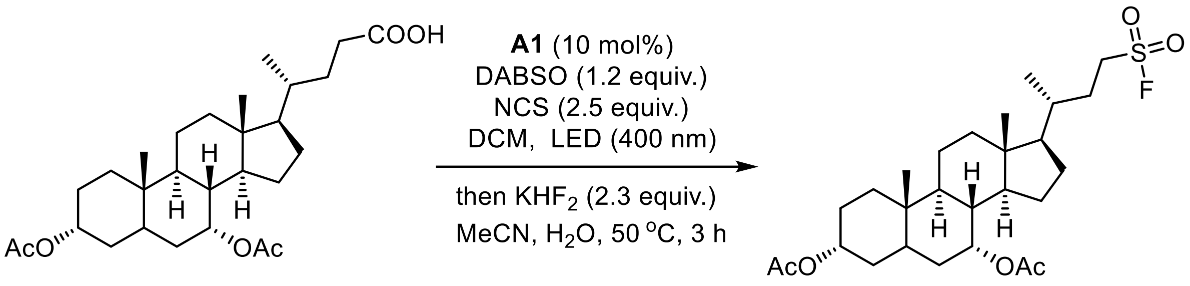

S15 **5l**

According to **GP7**, the reaction was carried out with acid **S15** (118 mg, 0.3 mmol), DABSO (108 mg, 0.45 mmol, 1.5 equiv.), acridine catalyst **A3** (9 mg, 0.03 mmol, 10 mol%), *N*-fluorobenzenesulfonimide (142 mg, 0.45 mmol, 1.5 equiv.), and degassed dichloromethane (6 mL). The test-tube was capped and the reaction mixture was irradiated with LED light (*λ* = 400 nm) while stirring at room temperature for 12 h. The reaction mixture was concentrated under reduced pressure, and the remaining material was purified by flash chromatography on silica gel (hexane/ ethyl acetate 4 : 1 v/v) to give the sulfonyl fluoride **5l** (102 mg, 66%) as a colorless solid.

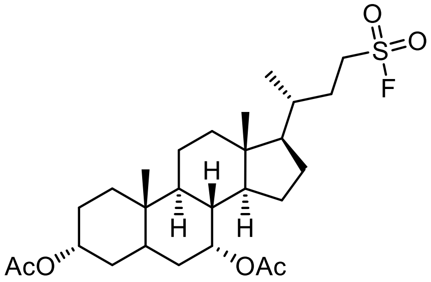

m.p.: 86–90°C. – $[\alpha]_D^{23}$ = + 75.1 (c 0.16, $CHCl_3$). – ^1H NMR (500 MHz, $CDCl_3$): 4.88 (1 H, q, *J* = 3.1 Hz), 4.59 (1 H, tt, *J* = 11.4, 4.5 Hz), 3.40 (1 H, ddt, *J* = 18.7, 11.4, 4.3 Hz), 3.32–3.22 (1 H, m), 2.11–1.91 (10 H, m), 1.85 (3 H, qd, *J* = 10.8, 4.1 Hz), 1.76–1.55 (6 H, m), 1.54–1.23 (7 H, m), 1.23–1.03 (4 H, m), 0.99 (3 H, d, *J* = 6.5 Hz), 0.93 (3 H, s), 0.67 (3 H, s) ppm. – ^{13}C NMR (125 MHz, $CDCl_3$): 170.6, 170.4, 74.1, 71.1, 55.3, 50.4, 48.4 (d, *J* = 16.2 Hz), 42.8, 40.9, 39.4, 37.9, 34.9, 34.8, 34.6,

Go back to table of contents

34.5, 34.0, 31.3, 29.2, 28.0, 26.8, 23.5, 22.7, 21.6, 21.5, 20.6, 18.2, 11.7 ppm. – ^{19}F NMR (470 MHz, $CDCl_3$): 52.1 ppm. – IR: 2939, 2812, 1730, 1504, 1462, 1403, 1250, 891 cm^{-1}. – HRMS: calcd for $C_{27}H_{43}FO_6SNa$: 537.2657, found 537.2654 [M+Na$^+$].

Computational Data

1. Software

Quantum chemical calculations were performed using the Stampede2 and Frontera supercomputers at the Texas Advanced Computing Center (TACC)[16] hosted by the University of Texas and the Expanse supercomputer at the San Diego Supercomputer Center (SDSC) hosted by UC San Diego.[17] DFT/TD-DFT geometry optimization, vibrational frequency, and IRC calculations were conducted using Gaussian 16 (rA.03).[18] The CREST utility[19] of the xTB software suite[20,21] was used in conjunction with manual conformational searching to locate initial starting geometries for optimization via DFT. General day-to-day visualization and monitoring of calculations was performed with Chemcraft (v1.8-610b).[22] Energy decomposition analysis was performed using the Absolutely Localized Molecular Orbital Energy Decomposition Analysis (ALMO-EDA2) and Complementary Occupied-Virtual orbital Pairs (COVP) methods of Head-Gordon and co-workers[23,24] as implemented in Q-Chem 5.3.1.[25] Activation Strain/Distortion Interaction calculations were performed using the Bash script pASDI.[26] Electron-hole[27] analyses were performed using Multiwfn 3.8(dev) using formatted checkpoint and log files derived from Gaussian 16.[28] NBO calculations were performed with the NBO 7.0 program suite.[29] Images of optimized minima and transition states were prepared using CYLview.[30]

2. Details of Computational Methods

Gaussian 16 DFT calculations

Geometries of ground state minima and transition states were optimized without constraints using the D3(BJ)[31,32] dispersion-corrected PW6B95[33] DFA. The PW6B95(D3BJ) DFA was selected on the basis of its excellent general performance in both thermochemical and NCI benchmarking studies.[34] DFT calculations were performed using the def2-TZVP[35] basis set in the SMD solvation model[36] using the "dichloromethane" keyword. Convergence criteria for these calculations was set to "tight" and an ultrafine grid was selected. Frequency calculations at the same level of theory were used to confirm the nature of the isolated stationary points. Geometries with zero imaginary frequencies were deemed minima whereas those with exactly one imaginary frequency along the chemical path of interest were deemed transition states. IRC calculations were performed to further corroborate that the located transition states connected reactants to products. The quasi-harmonic approximation from Grimme *et al*[37] was applied via GoodVibes[38] to all structures to correct for potential errors associated with low magnitude vibrational frequencies using a cut-off frequency of 50 cm^{-1}. Single point calculations were performed at the def2-TZVP-optimized

stationary points at the PW6B95(D3BJ) / def2-TZVPPD / SMD (DCM) level of theory. The electronic energy calculated at the def2-TZVPPD level replaced that calculated at the def2-TZVP level and is reported in the final calculated thermodynamic values. The def2-TZVPPD basis set was implemented in G16 by appending diffuse functions obtained from the EMSL BSE[39] to the G16-available def2-TZVPP basis set.

3. Distortion/Interaction-Activation Strain Analysis of TS1 and TS2

Given a reaction coordinate ζ connecting reactants to products through a transition state geometry, the Distortion/Interaction-Activation/Strain model[40] allows for the partitioning of the electronic energy along the reaction coordinate, $\Delta \boldsymbol{E}^{\zeta}$, into the sum of two terms:

$$\Delta \boldsymbol{E}^{\zeta} = \Delta \boldsymbol{E}^{\zeta}_{dist} + \Delta \boldsymbol{E}^{\zeta}_{int} \qquad (1)$$

where $\Delta \boldsymbol{E}^{\zeta}_{dist}$ is the sum of the strain energy required for two non-interacting equilibrium reactant geometries (**R1** and **R2**) to deform over the course of a chemical reaction:

$$\Delta \boldsymbol{E}^{\zeta}_{dist} = \Delta \boldsymbol{E}^{\zeta}_{dist}(\mathbf{R1}) + \Delta \boldsymbol{E}^{\zeta}_{dist}(\mathbf{R2}) \qquad (2)$$

$$\Delta \boldsymbol{E}^{\zeta}_{dist}(\boldsymbol{R1}) = \Delta \boldsymbol{E}_{dist}(\boldsymbol{R1}) - \Delta \boldsymbol{E}(\boldsymbol{R1}) \qquad (3)$$

$$\Delta \boldsymbol{E}^{\zeta}_{dist}(\boldsymbol{R2}) = \Delta \boldsymbol{E}_{dist}(\boldsymbol{R2}) - \Delta \boldsymbol{E}(\boldsymbol{R2}) \qquad (4)$$

and $\Delta \boldsymbol{E}^{\zeta}_{int}$ is the interaction energy between the two distorted fragments along the reaction coordinate:

$$\Delta \boldsymbol{E}^{\zeta}_{int} = \Delta \boldsymbol{E}^{\zeta} - \Delta \boldsymbol{E}^{\zeta}_{dist} \qquad (5)$$

A distortion/interaction-activation strain analysis was performed for **TS1** and **TS2** at the PW6B95(D3BJ) / def2-TZVP / SMD (DCM) level of theory. IRC calculations were performed for **TS1** and **TS2** at the same level of theory using a step-size of 3 to generate a high density of nonstationary points along the reaction coordinate through each transition state. The Bash script pASDI was used to extract and create single point energy calculations for intermediate geometries obtained along the IRCs of **TS1** and **TS2**. In total, 79 intermediate geometries were extracted from each of the **TS1** and **TS2** IRC calculations. Additionally, pASDI can be used to define and create G16 single point calculations for molecular fragments to investigate how $\Delta \boldsymbol{E}^{\zeta}_{dist}$ and $\Delta \boldsymbol{E}^{\zeta}_{int}$ fluctuate over the course of a reaction at the level of individual reagents. Fragment definitions were created for **TS1** and **TS2** according to Figure S6, with the green fragment consistently representing **CH_3I**. In total, 474 single point calculations were performed to obtain representative

electronic energies along the intrinsic reaction coordinates of **TS1** and **TS2**. These electronic energies were plotted with respect to the infinitely separated reagent equilibrium energies and projected along the I—CH_3 bond coordinate of each reaction.[41]

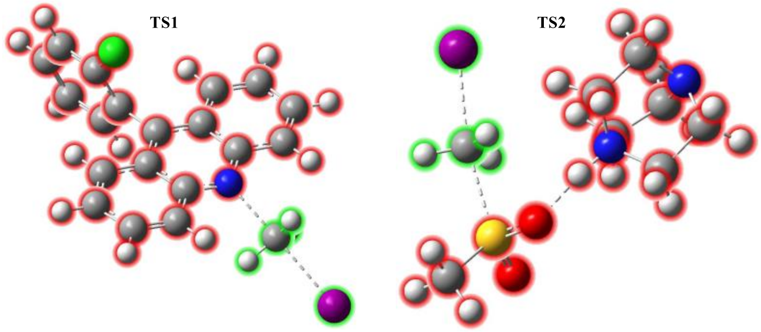

Figure S6. Fragment definitions for **TS1** and **TS2**.

4. Energy Decomposition Analysis via ALMO-EDA2

The second generation Absolutely Localized Molecular Orbital Energy Decomposition Analysis (ALMO-EDA2) method of Head-Gordon and co-workers was employed to gain quantitative insight into the intermolecular forces governing the interaction energy of **TS1** and **TS2**. This method decomposes the interaction energy, $\Delta\boldsymbol{E}_{int}$, into three initial terms:

$$\Delta\boldsymbol{E}_{int} = \Delta\boldsymbol{E}_{Frz} + \Delta\boldsymbol{E}_{Pol} + \Delta\boldsymbol{E}_{CT} \quad (6)$$

where $\Delta\boldsymbol{E}_{Frz}$ is the difference between the energy of the isolated, non-interacting fragments and the "frozen density" energy, the energy associated with bringing the fragments together in the transition state geometry without allowing intrafragment orbital relaxation nor interfragment delocalization, thus isolating the $\Delta\boldsymbol{E}_{Frz}$ term from polarization and charge transfer. The $\Delta\boldsymbol{E}_{Pol}$ term is the energy contribution associated with polarization obtained by allowing the frozen fragment-localized orbitals to relax without interfragment orbital delocalization. Finally, the $\Delta\boldsymbol{E}_{CT}$ term arises from the energy lowering effects of donor/acceptor interactions resulting from interfragment orbital delocalization(s).

Using ALMO-EDA2, the $\Delta\boldsymbol{E}_{Frz}$ term can be further decomposed into three constituent terms:

$$\Delta\boldsymbol{E}_{Frz} = \Delta\boldsymbol{E}_{Pauli} + \Delta\boldsymbol{E}_{Elec} + \Delta\boldsymbol{E}_{Disp} \quad (7)$$

Where $\Delta\boldsymbol{E}_{Pauli}$ represents Pauli repulsion, $\Delta\boldsymbol{E}_{Elec}$ represents permanent electrostatics, and $\Delta\boldsymbol{E}_{Disp}$ represents attractive interactions associated with dispersion. ALMO-EDA2 was employed at the PW6B95(D3BJ) / def2-TZVP / SMD (DCM) level of theory in Q-Chem 5.3 using the geometries of

TS1 and **TS2** optimized at the same level of theory. The results of the ALMO-EDA2 analysis are tabulated in **Table S1**.

Table S4. ALMO-EDA2 energy decomposition analysis of **TS1** and **TS2**.

**Structure**	**prep**	**ΔE_{Pauli}**	**ΔE_{Disp}**	**ΔE_{Elec}**	**ΔE_{CT}**	**ΔE_{Pol}**	**ΔE_{Sol}**	**Total $\Delta E^{\ddagger}_{int}$**
**TS1**	0.0	73.0	-11.4	-37.7	-24.7	-7.8	1.7	-6.9
**TS2**	0.0	57.6	-12.0	-31.5	-25.3	-3.2	3.8	-10.6

Energies reported in kcal/mol.

5. Decomposition of the ΔE_{CT} terms via Complementary Occupied-Virtual orbtial Pairs (COVPs)

To gain insight into the dominant donor/acceptor orbital interactions contributing to the **ΔE_{CT}** terms of **TS1** and **TS2**, Charge Decomposition Analysis (CDA) was performed using the Complementary Occupied-Virtual orbital Pairs (COVP) method in tandem with the ALMO-EDA2 method at the PW6B95(D3BJ) / def2-TZVP / SMD (DCM) level of theory.

COVP analysis of **TS1** identified a dominant stabilizing (-19.0 of **ΔE_{CT}** = -24.7 kcal/mol) interaction between an acridinyl N-centered orbital donor and **CH_3I** σ* orbital acceptor. Similarly, COVP analysis of **TS2** indicated a single dominant stabilizing interaction (-18.9 of **ΔE_{CT}** = -25.3 kcal/mol) between a S-centered orbital donor and a **CH_3I** σ* orbital acceptor.

6. Optimized Geometries (PW6B95(D3BJ) / def2-TZVP / SMD (DCM))

A1

E(RPW6B95D3) = -1247.96069158

Charge = 0 Multiplicity = 1

C	-7.9348919406	-1.3268358786	-0.7669072648
C	-6.8039455570	-1.7766712762	-0.1667079500
C	-5.7210673330	-0.8965378580	0.0935284215
C	-5.8476853263	0.4782942227	-0.2802285898
C	-7.0441570563	0.9052932370	-0.9141807869
C	-8.0552355729	0.0302729443	-1.1495558175
C	-4.7771352669	1.3344054275	-0.0155293525
C	-3.6313061258	0.8173627722	0.5922391302
C	-3.6084164929	-0.5742938721	0.9175386060
C	-2.4471013676	-1.1124496658	1.5313570506
H	-2.4498243572	-2.1650981159	1.7680242398

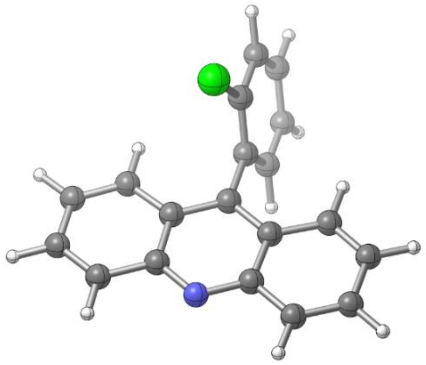

C	-1.3781526629	-0.3224620816	1.8048332166
C	-1.4024031830	1.0563611654	1.4869669203
C	-2.4950326221	1.6094238809	0.9010795748
H	-8.7510118798	-2.0055952898	-0.9611647163
H	-6.6899700475	-2.8085619794	0.1270841847
H	-7.1371269320	1.9374240220	-1.2119434320
H	-8.9583321822	0.3663373179	-1.6346609732
H	-0.4998808535	-0.7425149737	2.2703352705
H	-0.5437407930	1.6681656429	1.7155169445
H	-2.5109820953	2.6615757758	0.6639162039
N	-4.6302078094	-1.3923717232	0.6731609312
C	-4.8670007089	2.7731536129	-0.3526496461
C	-4.2073320626	3.3244570550	-1.4440680747
C	-5.6328697428	3.6238074190	0.4374169308
C	-4.2958326234	4.6716789680	-1.7432080096
C	-5.7289481256	4.9725730252	0.1533648052
H	-6.1534046370	3.2098125915	1.2872213838
C	-5.0584602812	5.4966693867	-0.9383348215
H	-3.7728990441	5.0641399376	-2.6000391298
H	-6.3262051146	5.6122136452	0.7836478519
H	-5.1275931826	6.5480364836	-1.1692743129
Cl	-3.2539759499	2.3090115106	-2.4797310886

MeI

E(RPW6B95D3) = -337.872505071

Charge = 0 Multiplicity = 1

C	0.3100809855	1.4039550538	-0.0249881567
H	0.6887772070	0.3924050242	0.0096085016
H	0.6888817987	1.9399155254	-0.8835697043
H	-0.7698756656	1.4241780832	0.0098892933
I	1.0210596844	2.4099573733	1.7171443460

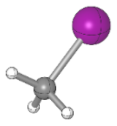

TS1

E(RPW6B95D3) = -1585.80657288

Charge = 0 Multiplicity = 1

C	-7.8518572324	-1.3469130248	-0.9939093828
C	-6.7301316937	-1.8162395071	-0.3880450053
C	-5.6757890085	-0.9337130596	-0.0458703581
C	-5.8113095871	0.4528268730	-0.3626073808
C	-7.0014403813	0.8983106820	-0.9932973555
C	-7.9952045830	0.0252284786	-1.2961289583
C	-4.7565243212	1.3127041598	-0.0521227019
C	-3.5915296321	0.7898417706	0.5123395747
C	-3.5372083791	-0.6098706874	0.7895695805

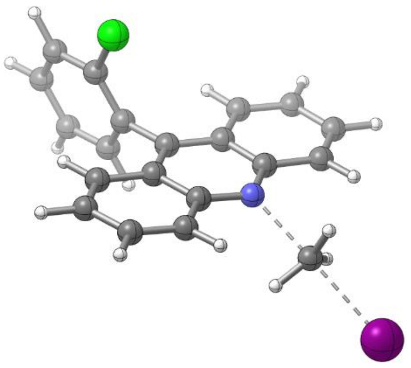

C	-2.3415525071	-1.1515711628	1.3236144147
H	-2.2772179792	-2.2137030299	1.4894679745
C	-1.2783228978	-0.3478001077	1.5856714638
C	-1.3403553903	1.0421440027	1.3428058661
C	-2.4640238349	1.5934599533	0.8189535422
H	-8.6415581397	-2.0324544628	-1.2590546821
H	-6.6158395202	-2.8684818510	-0.1890903712
H	-7.0986223197	1.9433224296	-1.2388854369
H	-8.8936903011	0.3724014769	-1.7809660521
H	-0.3703961998	-0.7762075588	1.9806718014
H	-0.4860221681	1.6604034387	1.5680401068
H	-2.5116501362	2.6526239172	0.6227805275
N	-4.5770741430	-1.4125320510	0.5442320754
C	-4.8784858520	2.7650678793	-0.3111706377
C	-4.2557114618	3.3797677775	-1.3898745224
C	-5.6381921367	3.5579619320	0.5414808546
C	-4.3749966318	4.7381585211	-1.6178384427
C	-5.7627450620	4.9173756691	0.3283385219
H	-6.1300350336	3.0921206433	1.3813403163
C	-5.1299254430	5.5070187465	-0.7523426749
H	-3.8814639813	5.1826800710	-2.4665904875
H	-6.3535796538	5.5140287824	1.0049330089
H	-5.2228958066	6.5671516110	-0.9279115844
Cl	-3.3133636023	2.4318336701	-2.4958600191
C	-4.7540762940	-3.1903772335	1.6571510652
H	-4.3812013386	-3.7761289952	0.8432968504
H	-4.0860657666	-2.7940488714	2.3922490408
H	-5.8033403730	-3.0114141847	1.7639129847
I	-5.0089232106	-5.3557066969	3.1034404855

A1-Me$^+$ / I$^-$

E(RPW6B95D3) = -1585.86089491

Charge = 0 Multiplicity = 1

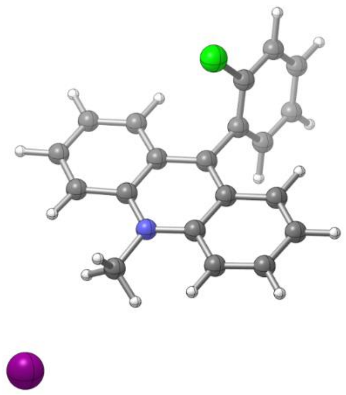

C	-7.9064665720	-1.2922607260	-0.9534207530
C	-6.8108624730	-1.7867201100	-0.3099895140
C	-5.7516231990	-0.9283132600	0.0403111860
C	-5.8400295950	0.4485751770	-0.3082856540
C	-7.0019355550	0.9165338570	-0.9705181610
C	-8.0153389700	0.0700884990	-1.2824176110
C	-4.7765056850	1.3014941050	-0.0101169310
C	-3.6189506450	0.7828133550	0.5724082410
C	-3.5621830170	-0.5992666870	0.8989503730
C	-2.3704688700	-1.1250371540	1.4333003560
H	-2.2759879410	-2.1743518730	1.6420559860

C	-1.3041929060	-0.3051390080	1.6559453120
C	-1.3632959460	1.0717443820	1.3771419420
C	-2.4948019170	1.5993893460	0.8460988890
H	-8.7010015350	-1.9694894530	-1.2241190550
H	-6.7583740510	-2.8397888110	-0.1067426260
H	-7.0576704890	1.9593301840	-1.2338049260
H	-8.8931805030	0.4315575950	-1.7925773690
H	-0.3952088090	-0.7285441430	2.0529858940
H	-0.5090948030	1.6975941830	1.5775643690
H	-2.5547333640	2.6506640980	0.6183403770
N	-4.6484507930	-1.3895487940	0.6873408430
C	-4.8797403280	2.7470799400	-0.3078622650
C	-4.2548899300	3.3188396750	-1.4083893950
C	-5.6229207440	3.5701085550	0.5301229590
C	-4.3581967240	4.6714828570	-1.6728078970
C	-5.7275832090	4.9245773550	0.2796252450
H	-6.1153692380	3.1345083420	1.3855086890
C	-5.0951065940	5.4738600360	-0.8222333070
H	-3.8667166510	5.0854874030	-2.5379418670
H	-6.3041606020	5.5485493690	0.9435004270
H	-5.1747396600	6.5298408310	-1.0264742850
Cl	-3.3355585560	2.3237341900	-2.4902214290
C	-4.6197174090	-2.7711644720	1.1753392100
H	-4.1645079290	-3.4264814380	0.4401052650
H	-4.0630398800	-2.8103853740	2.0989202190
H	-5.6228848930	-3.1006560720	1.3921049950
I	-4.4060775060	-6.2977141400	2.6240177360

MeSO$_2$H

E(RPW6B95D3) = -589.775039374

Charge = 0 Multiplicity = 1

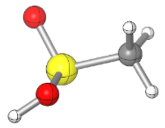

S	-0.5874389593	2.1694317384	0.0412973061
O	0.9734947852	1.8586404008	-0.3561569044
O	-1.2766042293	2.6862306970	-1.1449597032
C	-0.9928226249	0.4408940070	0.1283504227
H	-2.0616776009	0.3735451344	0.3073192712
H	-0.7356324869	-0.0129970869	-0.8248159422
H	-0.4441679617	-0.0189518925	0.9442267836
H	1.4949784577	2.6563745617	-0.1920464938

DABCO

E(RPW6B95D3) = -345.911628501

Charge = 0 Multiplicity = 1

C	-1.2483988393	0.8851799918	-0.0375017948

C	-1.7729443768	2.3369918865	0.0379336382
H	-1.5627935762	0.4001806773	-0.9597323737
H	-1.6168913339	0.2889287388	0.7950763160
H	-2.4375222353	2.5596413913	-0.7947162955
H	-2.3245012202	2.5092039231	0.9602537429
C	0.7167452385	1.5947708627	-1.1683681456
H	1.8031108317	1.6164948795	-1.1058030842
H	0.4452967866	1.0333032144	-2.0603150962
C	0.1301241634	3.0241985622	-1.2065088136
H	0.9183936384	3.7722382282	-1.2664878750
H	-0.5234524227	3.1588766090	-2.0662705792
C	0.1920492100	3.0471128713	1.1680797132
H	1.0409663051	3.7253314495	1.1057089596
H	-0.3760902632	3.3050186800	2.0598500962
C	0.6550130356	1.5730376982	1.2063274682
H	1.7393934688	1.5018084317	1.2660179906
H	0.2387124682	1.0518133936	2.0662638985
N	-0.6536075066	3.2734419033	-0.0003810737
N	0.2113634279	0.8798450076	0.0002173084

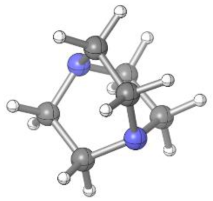

TS2

E(RPW6B95D3) = -1273.56815581

Charge = 0 Multiplicity = 1

S	3.7684230226	1.7852294724	-0.9410340133
O	2.7314687213	1.7333429231	0.1472061759
O	5.0565457618	2.3394814574	-0.4835438674
C	4.0916674837	0.0483012938	-1.2390455506
H	4.8560418893	-0.0359637241	-2.0053418876
H	4.4410938409	-0.3773857923	-0.3011723814
H	3.1741073391	-0.4337401536	-1.5616794263
H	1.8924107033	3.1015064737	0.5140952230
C	2.5753965054	3.1528663399	-2.6402538511
H	2.8642638975	2.5313824679	-3.4658343017
H	1.7390917882	2.8658040868	-2.0365224752
H	3.2446398925	3.9243728914	-2.3151796508
I	1.1610251498	4.7446112941	-3.9904076600
C	2.0460032090	5.1790671583	0.3525843668
C	1.3957831134	6.3733027053	1.0659622600
H	3.1052772024	5.0820853824	0.5645975152
H	1.8957510432	5.2150189321	-0.7212420619
H	2.0445360751	6.7575742133	1.8477242951
H	1.2127004289	7.1715617020	0.3533162808
C	1.3576561913	3.9419111265	2.3311934632
H	1.0299793340	2.9597319359	2.6530898476

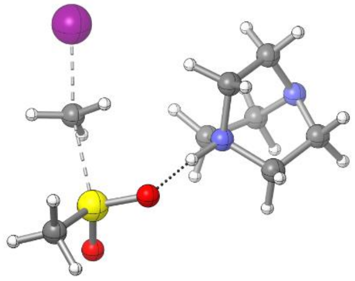

H	2.3819520356	4.0963428807	2.6527742121
C	0.4163586660	5.0661014070	2.7872108194
H	-0.5264069995	4.6592839789	3.1415641485
H	0.8715808743	5.6235377338	3.6001891942
C	-0.6716883967	5.2667497352	0.6865067280
H	-1.6709620178	5.1088279662	1.0806024644
H	-0.7495775862	5.8950107372	-0.1961328418
C	-0.0274277214	3.9175043715	0.3325594992
H	-0.5215201919	3.0777336454	0.8089425897
H	0.0131480447	3.7485488517	-0.7379566724
N	0.1356040394	5.9682223657	1.6773439598
N	1.3676726609	3.9498341405	0.8438585986

$MeSO_2Me$

E(RPW6B95D3) = -629.165402607

Charge = 0 Multiplicity = 1

S	-0.4741290925	2.1836350634	-0.0424785456
O	0.9518885156	2.1414875539	-0.2525557848
O	-1.2920766677	2.8184854338	-1.0461886152
C	-1.0516381107	0.5359385065	0.1846557657
H	-2.1119446527	0.5555396159	0.4128336743
H	-0.8787855511	0.0180035462	-0.7545536487
H	-0.4875801811	0.0648605354	0.9824759825
C	-0.7859950644	2.9654690223	1.5037743582
H	-0.4438691549	3.9914588438	1.4026431549
H	-1.8510219393	2.9435285644	1.7084854210
H	-0.2263863114	2.4584505144	2.2824802478

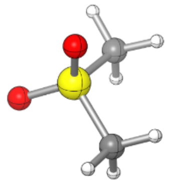

DABCO-H^+ / I^-

E(RPW6B95D3) = -644.466095368

Charge = 0 Multiplicity = 1

C	-0.4042369438	4.0933905521	0.7399047152
C	-0.5537952300	5.6072885004	0.9558822934
H	-0.7205248254	3.7762319009	-0.2476338317
H	-0.9237770837	3.5033469720	1.4860880877
H	-1.2267996181	6.0219824412	0.2118385653
H	-0.9641304313	5.8162477840	1.9395005222
C	1.7779151192	4.3609007105	-0.3077698547
H	2.8347492615	4.2070748644	-0.1198294000
H	1.4894417282	3.8017412965	-1.1904134891
C	1.3947344421	5.8464307845	-0.3813365176
H	2.2827355069	6.4494887644	-0.5433766103
H	0.7078902562	6.0256285250	-1.2033361851
C	1.5694184785	5.8626700539	1.9883174300

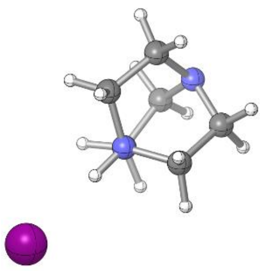

H	2.5734387765	6.2456606916	1.8307019452
H	1.1750263657	6.3077895368	2.8965280343
C	1.5864514913	4.3323337616	2.1208895596
H	2.5827342284	3.9272632249	2.2557040122
H	0.9453912507	3.9710976628	2.9176051162
N	0.7443412387	6.2631059363	0.8550566809
N	1.0454761649	3.7819806948	0.8498099825
H	1.1729273924	2.7552592576	0.8471963934
I	1.5437053411	0.3070986736	0.8370819802

7. TD-DFT and Hole/Electron of Acridinyl excited states

DFT and TD-DFT calculations to obtain the excitation energies of substituted acridinyl photocatalysts were performed using G16 at the ωB97X-D[42] / 6-311+G** / SMD (DCM) level of theory. The selection of the ωB97X-D DFA for ground state DFT and TD-DFT optimizations was motivated due to its accuracy across ground state geometries and energies relative to the GMTKN55 database[43] and its ability to generate physically sound geometries for low-lying singlet excited states.[44] Additionally, the range-separated hybrid nature of the ωB97X-D DFA serves as a bulwark against errors associated with long-range charge transfer excited states[45] by providing for accurate long-range interactions between hole and electron.[46] The dispersion-corrected ωB97X-D DFA also serves to appropriately treat noncovalent interactions.[47]

8. Benchmarking ωB97X-D / 6-311+G / SMD (DCM)**

To verify the use of TD-DFT at the ωB97X-D / 6-311+G** / SMD (DCM) level of theory for the calculation of excited state properties of acridinyl photocatalysts, benchmarking calculations were performed. The ground state and three lowest singlet excited states of unsubstituted acridine (**A4**) were first obtained via DFT and TD-DFT calculations, respectively, in the gas phase at the ωB97X-D / 6-311+G** level of theory and verified as minima on their respective energy surfaces via frequency calculations. This process was repeated in the solvent phase using the ωB97X-D / 6-311+G** / SMD (DCM) level of theory for optimization and frequency calculations. The differences in free energy between the optimized ground state and each of the optimized singlet excited states, the band origin E_0, were calculated and compared with theoretical CASSCF/MS-CASPT2[48] values, with both DFT/TD-DFT-based approaches demonstrating excellent agreement with the CASSCF/MS-CASPT2 literature values for unsubstituted acridine. The results from these calculations are summarized in Table S5.

Table S5. Benchmarking performance of selected TD-DFT method in comparision with previously reported CASSCF/MS-CASPT2 calculations for the three lowest singlet excited states of acridine (**A4**).

**State**	E_0 ωB97X-D / 6-311+G** / Gas Phase	E_0 **(Sol.)** ωB97X-D/6-311+G**/ SMD(DCM)	E_0 (CASSCF/MS-CASPT2)
S1	76.96	72.82	75.64
S2	77.52	72.41	77.02
S3	71.80	72.41	73.33

GoodVibes-corrected free energies (kcal/mol); cutoff frequency: 50 cm^{-1}, 1 atm, T = 298.15 K (25° C).

The referenced CASSCF/MS-CASPT2 calculations had identified that, after adiabatic relaxation, the lowest-lying singlet excited state for acridine corresponded to a ^{1}n- π* transition with the next two singlet excited states corresponding to close-lying ^{1}π–π* transitions. To determine whether the chosen TD-DFT method reliably reproduced these data, the nature of charge transfer between ground and each of the three low-lying singlet excited states was investigated by comparing the electron density difference between the ground and respective excited states[49a]:

$$\Delta\boldsymbol{\rho}(\boldsymbol{r}) = \boldsymbol{\rho}_{\boldsymbol{ES}}(\boldsymbol{r}) - \boldsymbol{\rho}_{\boldsymbol{GS}}(\boldsymbol{r})$$

Plots of the charge difference densities (Figure S7) revealed that the first and second absorptions were characterized as ^{1}π–π* transitions whereas the third absorption is of ^{1}n- π* character, in agreement with the literature-obtained CASSCF/MS-CASPT2 results.

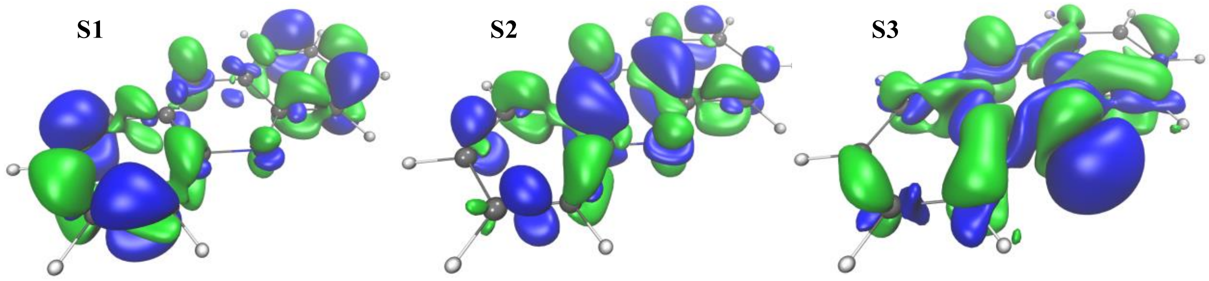

Figure S7. Plots of Ground/Excited State Charge Density Differences for acridine (**A4**). Green surfaces correspond to regions of increasing electronic density. Blue surfaces correspond to regions of decreased electronic density.

9. Electron-hole Analysis of Acid-Complexed Acridines

Electron-hole analysis can provide quantitative insight into the magnitude and direction of change in electronic density upon single electron excitation.[49] The electronic density difference $\Delta\boldsymbol{\rho}(\boldsymbol{r})$ between ground state density $\boldsymbol{\rho_{GS}}(\boldsymbol{r})$and excited state density $\boldsymbol{\rho_{ES}}(\boldsymbol{r})$**:**

$$\Delta\boldsymbol{\rho}(\boldsymbol{r}) = \boldsymbol{\rho_{ES}}(\boldsymbol{r}) - \boldsymbol{\rho_{GS}}(\boldsymbol{r}) \tag{8}$$

can be partitioned into regions corresponding to a decrease (hole) or increase (electron) of electronic density upon excitation:

$$\boldsymbol{\rho_{electron}}(\boldsymbol{r}) = \begin{Bmatrix} \Delta\boldsymbol{\rho}(\boldsymbol{r}) & \textbf{if } \Delta\boldsymbol{\rho}(\mathbf{r}) > \mathbf{0} \\ \mathbf{0} & \textbf{if } \Delta\boldsymbol{\rho}(\mathbf{r}) < \mathbf{0} \end{Bmatrix} \tag{9}$$

$$\boldsymbol{\rho_{hole}}(\boldsymbol{r}) = \begin{Bmatrix} \Delta\boldsymbol{\rho}(\boldsymbol{r}) & \textbf{if } \Delta\boldsymbol{\rho}(\mathbf{r}) < \mathbf{0} \\ \mathbf{0} & \textbf{if } \Delta\boldsymbol{\rho}(\mathbf{r}) > \mathbf{0} \end{Bmatrix} \tag{10}$$

The degree to which these two regions overlap is represented via the **S_r index** which has a theoretical upper limit of 1.0 representing perfect hole/electron overlap:

$$\boldsymbol{S_r} \text{ index } = \int \boldsymbol{S_r}(\boldsymbol{r})\boldsymbol{dr} = \int \sqrt{\boldsymbol{\rho_{hole}}(\boldsymbol{r})\boldsymbol{\rho_{electron}}(\boldsymbol{r})}\boldsymbol{dr} \tag{11}$$

By superimposing a three-dimensional grid of X/Y/Z coordinates atop a molecule, it is possible to discretize the density functions $\boldsymbol{\rho_{electron}}(\boldsymbol{r})$ and $\boldsymbol{\rho_{hole}}(\boldsymbol{r})$ to obtain the respective barycenters of electron ($\mathbf{R}_{ele}$)and hole ($\mathbf{R}_{hole}$) density distributions:

$$\boldsymbol{R_{ele}} = \frac{\int \boldsymbol{r}\boldsymbol{\rho_{electron}}(\boldsymbol{r})\boldsymbol{dr}}{\int \boldsymbol{\rho_{electron}}\,\boldsymbol{dr}} = (\boldsymbol{X_{ele}}, \boldsymbol{Y_{ele}}, \boldsymbol{Z_{ele}}) \tag{12}$$

$$\boldsymbol{R_{hole}} = \frac{\int \boldsymbol{r}\boldsymbol{\rho_{hole}}(\boldsymbol{r})\boldsymbol{dr}}{\int \boldsymbol{\rho_{hole}}\,\boldsymbol{dr}} = (\boldsymbol{X_{hole}}, \boldsymbol{Y_{hole}}, \boldsymbol{Z_{hole}}) \tag{13}$$

The magnitude of the vector connecting $\mathbf{R}_{ele}$ and $\mathbf{R}_{hole}$ is captured by the **D index**:

$$\boldsymbol{D} \text{ index} = |\boldsymbol{R_{ele}} - \boldsymbol{R_{hole}}| \tag{14}$$

Two centroids of charge $\boldsymbol{\sigma}_{\mathbf{hole},\lambda}$ and $\boldsymbol{\sigma}_{\mathbf{electron},\lambda}$, representing explicit regions of electron depletion and increment, respectively, can be derived for visualization purposes by characterizing the RMSD of hole and electron with respect to the X/Y/Z direction. For instance, the X component of $\boldsymbol{\sigma}_{\mathbf{hole},\lambda}$ is expressed by:

$$\boldsymbol{\sigma_{hole,X}} = \sqrt{\int (\boldsymbol{x} - \boldsymbol{X_{hole}})^2\, \boldsymbol{\rho_{hole}}(\boldsymbol{r})\, \boldsymbol{dr}} \tag{15}$$

The overall difference between RMSD of hole and electron, the **Δσ index**, represents the breadth of distribution between hole and electrons:

$$\Delta\boldsymbol{\sigma} \text{ index} = |\boldsymbol{\sigma_{electron}}| - |\boldsymbol{\sigma_{hole}}| \tag{16}$$

The half distance between these two centroids is termed the **H index**:

$$\boldsymbol{H}\text{ index} = \frac{(|\sigma_{electron}| - |\sigma_{hole}|)}{\mathbf{2}} \tag{17}$$

The **H index** can also be expressed in the direction of CT via $\mathbf{H}_{CT}$ by multiplying the **H** index by $\mathbf{u}_{CT}$**,** a CT-orineted unit vector:

$$\boldsymbol{H_{CT}} = |\boldsymbol{H}\ \cdot \boldsymbol{u_{CT}}| \tag{18}$$

Finally, the ***t* index** measures the degree of separation between hole and electron in the direction of charge transfer. A large-valued **t index** indicates a significant degree of CT character.

$$\boldsymbol{t}\text{ index} = \boldsymbol{D}\text{ index} - \boldsymbol{H_{CT}} \tag{19}$$

Table S5 summarizes the quantitative characterization of Hole and Electron for the series of acid-complexed acridines.

Table S5. Quantitative characterization of electron-hole analysis.

Transition	D, Å	S_r, a.u.	Δσ	H_{CT}	H, Å	t, Å	Δ*E*, eV
**S1**	0.133	0.86349	-0.139	1.401	2.909	-1.268	3.577
**S2**	0.107	0.91301	0.128	1.18	2.829	-1.073	4.082
**S3**	1.24	0.49028	0.593	1.562	2.424	-0.322	4.598
**S1-H**	0.24	0.81995	-0.283	1.502	2.879	-1.261	3.426
**S2-H**	0.175	0.87277	0.029	1.305	2.823	-1.131	4.032
**S3-H**	4.208	0.17133	0.471	1.63	2.529	2.578	4.425

10. Optimized Geometries (ωB97X-D / 6-311+G** / Gas Phase)

A4

E(RwB97XD) = -555.497377004

Charge = 0 Multiplicity = 1

C	-7.9215651133	-1.3436806848	-0.8286625556
C	-6.7966759325	-1.8031517101	-0.2172129502
C	-5.7108713166	-0.9179317200	0.0686668983
C	-5.8372232317	0.4600627935	-0.3038019602
C	-7.0349453722	0.9021649052	-0.9440492528
C	-8.0449844604	0.0277602709	-1.1983107269
C	-4.7716310962	1.3077539606	-0.0184225788
C	-3.6336808165	0.8093585134	0.6078197420
C	-3.6110026645	-0.5850699028	0.9373980023
C	-2.4491808581	-1.1140061728	1.5813777341
H	-2.4498034451	-2.1701721363	1.8229964469
C	-1.3922797517	-0.3086874779	1.8725508155
C	-1.4171423046	1.0783762614	1.5436763148

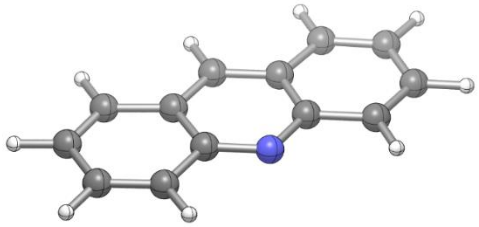

C	-2.5033259345	1.6204970158	0.9307150345
H	-8.7391434404	-2.0235739900	-1.0408640814
H	-6.6834759789	-2.8412750320	0.0714949848
H	-0.5160397279	-0.7200842495	2.3610943980
H	-2.5297847730	2.6753736748	0.6772046253
N	-4.6253963186	-1.4126678896	0.6704565605
H	-8.9520968994	0.3680503542	-1.6844613814
H	-0.5616332266	1.6980692496	1.7867351444
H	-4.8281794960	2.3597301388	-0.2848093688
H	-7.1213840776	1.9475338231	-1.2223737741

A4-S1

E(TD-DFT) = -555.370014962

Charge = 0 Multiplicity = 1

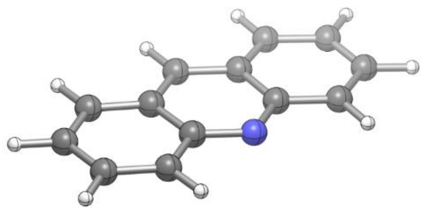

C	-7.9590404898	-1.3346379438	-0.8475980637
C	-6.7882889686	-1.7873713137	-0.2170529696
C	-5.7241124180	-0.9178382549	0.0626868244
C	-5.8514482639	0.4652824305	-0.3113988880
C	-7.0278829540	0.8890966942	-0.9378779966
C	-8.0795859972	-0.0049861845	-1.2063817847
C	-4.7726701322	1.3270832632	-0.0233172437
C	-3.6202525729	0.8189616244	0.6116631778
C	-3.5979952727	-0.5808156436	0.9422771231
C	-2.4591082995	-1.1011288942	1.5739609104
H	-2.4583320492	-2.1575289474	1.8162627329
C	-1.3564015897	-0.2880170610	1.8839622058
C	-1.3796346687	1.0570602307	1.5654373520
C	-2.5088759344	1.6054295404	0.9316684407
H	-8.7632409294	-2.0321684326	-1.0497398360
H	-6.6764392609	-2.8261644976	0.0712007590
H	-0.4914332622	-0.7209583582	2.3723678276
H	-2.5235778203	2.6620745644	0.6830437951
N	-4.6251387624	-1.4174592351	0.6716698495
H	-8.9782668163	0.3547515792	-1.6931922990
H	-0.5344878681	1.6932217743	1.8000611483
H	-4.8291634526	2.3780347930	-0.2894445670
H	-7.1260684534	1.9325082679	-1.2210404270

A4-S2

E(TD-DFT) = -555.315308291

Charge = 0 Multiplicity = 1

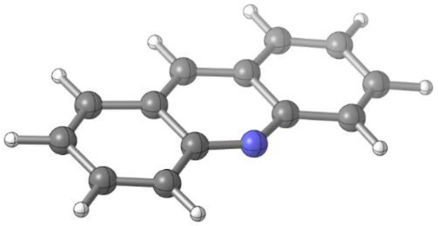

C	-0.0295445165	3.6062575459	0.7878267740
C	-0.0399657524	2.4333807822	1.4600111029
C	-0.0293405155	1.1986623365	0.7284168284

C	-0.0057663646	1.2252266921	-0.7713474480
C	0.0029799224	2.5091509750	-1.3816697475
C	-0.0078088678	3.6597166453	-0.6592673228
C	0.0047514980	0.0000001954	-1.4219618104
C	-0.0057475076	-1.2252265075	-0.7713473427
C	-0.0293214065	-1.1986625073	0.7284167719
C	-0.0399306768	-2.4333811132	1.4600112706
H	-0.0562467445	-2.3944033888	2.5419508598
C	-0.0294944910	-3.6062578934	0.7878269043
C	-0.0077586693	-3.6597164818	-0.6592672351
C	0.0030156340	-2.5091506359	-1.3816697086
H	-0.0381258992	4.5351534933	1.3466082806
H	-0.0562807897	2.3944029109	2.5419507082
H	-0.0380636986	-4.5351540310	1.3466082868
H	0.0186340334	-2.5432297477	-2.4675672302
N	-0.0379508564	-0.0000001758	1.2254832747
H	-0.0007464276	4.6214056196	-1.1553322022
H	-0.0006850800	-4.6214053445	-1.1553321940
H	0.0221136426	0.0000003529	-2.5098945022
H	0.0185987126	2.5432302777	-2.4675673186

A4-S3

E(TD-DFT) = -555.378915320

Charge = 0 Multiplicity = 1

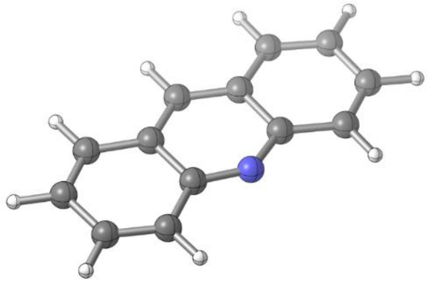

C	-3.5977352574	-0.7803511486	-0.0333089059
C	-2.4282356753	-1.4504588174	-0.0377167639
C	-1.1956475904	-0.7214617824	-0.0326543814
C	-1.2224379284	0.7765426752	-0.0227261035
C	-2.5038547499	1.3845667594	-0.0187191698
C	-3.6510959281	0.6643525218	-0.0236317054
C	-0.0000000138	1.4244517250	-0.0183052145
C	1.2224379119	0.7765426902	-0.0227259482
C	1.1956476078	-0.7214617583	-0.0326541437
C	2.4282357079	-1.4504587600	-0.0377176791
H	2.3903451122	-2.5310480186	-0.0446447366
C	3.5977352779	-0.7803510741	-0.0333091354
C	3.6510959149	0.6643526022	-0.0236319893
C	2.5038547197	1.3845668118	-0.0187199203
H	-4.5252994213	-1.3384064978	-0.0366529211
H	-2.3903450605	-2.5310480749	-0.0446439259
H	4.5252994520	-1.3384064084	-0.0366526307
H	2.5377236698	2.4690471135	-0.0110581352
N	0.0000000128	-1.2150126276	-0.0361907709
H	-4.6114143176	1.1597264350	-0.0200262592

H	4.6114142883	1.1597265404	-0.0200259231
H	-0.0000000248	2.5109990321	-0.0111920867
H	-2.5377237256	2.4690470615	-0.0110576904

11. Optimized Geometries (ωB97X-D / 6-311+G / SMD(DCM))**

A4

E(RwB97XD) = -555.518373780

Charge = 0 Multiplicity = 1

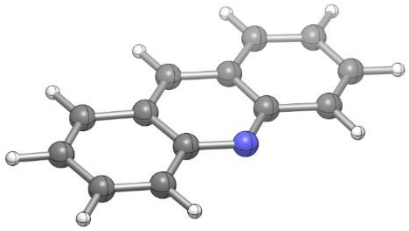

C	-7.9253978612	-1.3412708994	-0.8309392667
C	-6.8010032446	-1.8053714149	-0.2186519067
C	-5.7136440854	-0.9224194902	0.0684469725
C	-5.8371663790	0.4560110378	-0.3028483822
C	-7.0335863148	0.9030808059	-0.9436474326
C	-8.0464956480	0.0308379862	-1.1996956852
C	-4.7714933163	1.3051908262	-0.0177735288
C	-3.6333047890	0.8053573518	0.6089053650
C	-3.6077983507	-0.5886102126	0.9396508395
C	-2.4446900465	-1.1148280679	1.5835869001
H	-2.4342521253	-2.1707890434	1.8301360354
C	-1.3887686536	-0.3051135787	1.8733123075
C	-1.4159847390	1.0818770156	1.5433954393
C	-2.5047596624	1.6209702166	0.9299614589
H	-8.7443755568	-2.0196386496	-1.0441199319
H	-6.6986987522	-2.8467701111	0.0659031047
H	-0.5113155325	-0.7145706789	2.3619575469
H	-2.5371053253	2.6751229332	0.6739677106
N	-4.6251942071	-1.4164277914	0.6714086621
H	-8.9532307433	0.3724967325	-1.6859900017
H	-0.5609925287	1.7027969203	1.7859406651
H	-4.8280278771	2.3569095549	-0.2840951261
H	-7.1141604977	1.9495885525	-1.2195936743

A4-S1

E(TD-DFT): -555.397834

Charge = 0 Multiplicity = 1

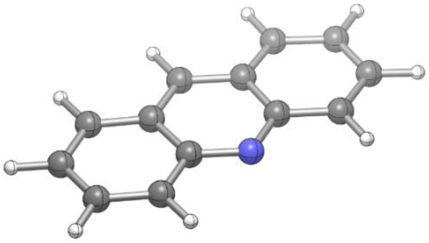

C	-7.92540000	-1.34127000	-0.83094000
C	-6.80100000	-1.80537000	-0.21865000
C	-5.71364000	-0.92242000	0.06845000
C	-5.83717000	0.45601000	-0.30285000
C	-7.03359000	0.90308000	-0.94365000
C	-8.04650000	0.03084000	-1.19970000
C	-4.77149000	1.30519000	-0.01777000
C	-3.63330000	0.80536000	0.60891000
C	-3.60780000	-0.58861000	0.93965000

C	-2.44469000	-1.11483000	1.58359000
H	-2.43425000	-2.17079000	1.83014000
C	-1.38877000	-0.30511000	1.87331000
C	-1.41598000	1.08188000	1.54340000
C	-2.50476000	1.62097000	0.92996000
H	-8.74438000	-2.01964000	-1.04412000
H	-6.69870000	-2.84677000	0.06590000
H	-0.51132000	-0.71457000	2.36196000
H	-2.53711000	2.67512000	0.67397000
N	-4.62519000	-1.41643000	0.67141000
H	-8.95323000	0.37250000	-1.68599000
H	-0.56099000	1.70280000	1.78594000
H	-4.82803000	2.35691000	-0.28410000
H	-7.11416000	1.94959000	-1.21959000

A4-S2

E(TD-DFT) = -555.397833684

Charge = 0 Multiplicity = 1

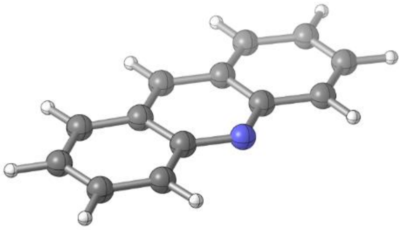

C	-0.0436149371	3.6167367918	0.7550726876
C	-0.0860935657	2.3733105783	1.4115572906
C	-0.0399829545	1.1679064546	0.6963740332
C	0.0521039937	1.2216660889	-0.7387218114
C	0.0930801686	2.4700912668	-1.3658390007
C	0.0452241972	3.6656335301	-0.6219881812
C	0.0970155295	-0.0000080111	-1.4465630998
C	0.0516299330	-1.2216728436	-0.7387350033
C	-0.0404479162	-1.1678968486	0.6963605450
C	-0.0870166709	-2.3732931924	1.4115291415
H	-0.1567729458	-2.3247290575	2.4929793166
C	-0.0450147932	-3.6167282172	0.7550300546
C	0.0437990331	-3.6656443415	-0.6220318770
C	0.0921198283	-2.4701074644	-1.3658596180
H	-0.0809449065	4.5304435124	1.3366973597
H	-0.1558751965	2.3247600564	2.4930064445
H	-0.0826932405	-4.5304278146	1.3366430306
H	0.1620117761	-2.5131721060	-2.4480896197
N	-0.0847556977	0.0000091726	1.3836169811
H	0.0787447283	4.6177195158	-1.1387420988
H	0.0769513123	-4.6177359792	-1.1387990039
H	0.1664799396	-0.0000171931	-2.5300042469
H	0.1630098930	2.5131561016	-2.4483623242

A4-S3

E(TD-DFT) = -555.397833875

Charge = 0 Multiplicity = 1

C	-0.0393350108	3.6167892340	0.7553139966
C	-0.0782301444	2.3734399706	1.4120669314
C	-0.0355852400	1.1680799434	0.6966631188
C	0.0498054218	1.2216854051	-0.7387481635
C	0.0867878225	2.4701149090	-1.3661493324
C	0.0424544226	3.6656231798	-0.6222044862
C	0.0922250712	-0.0000000307	-1.4467647299
C	0.0498062756	-1.2216854065	-0.7387480175
C	-0.0355853484	-1.1680799316	0.6966632024
C	-0.0782319162	-2.3734399464	1.4120669303
H	-0.1425083598	-2.3250228857	2.4938006982
C	-0.0393364302	-3.6167892192	0.7553140329
C	0.0424550659	-3.6656231595	-0.6222043382
C	0.0867894908	-2.4701149122	-1.3661491643
H	-0.0738424060	4.5304165019	1.3372226109
H	-0.1425054082	2.3250228998	2.4938007650
H	-0.0738450768	-4.5304164530	1.3372226196
H	0.1508310945	-2.5131912455	-2.4489402774
N	-0.0762442098	0.0000000245	1.3836763181
H	0.0728130956	4.6175495420	-1.1394407118
H	0.0728139632	-4.6175495290	-1.1394405452
H	0.1571554973	-0.0000000916	-2.5305106101
H	0.1508285121	2.5131912139	-2.4489395807

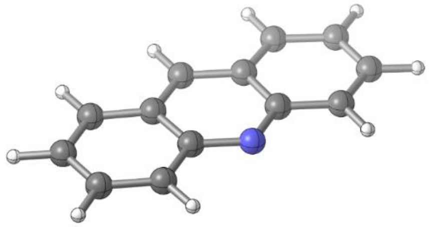

S0

E(RwB97XD) = -1475.27259352

Charge = 0 Multiplicity = 1

C	-7.8681769074	-1.4444278296	-0.8197639091
C	-6.7449224488	-1.8834330768	-0.1889798598
C	-5.6782544563	-0.9791217621	0.1011042784
C	-5.8134720227	0.3938803358	-0.2765803495
C	-7.0092449978	0.8119387636	-0.9388917265
C	-8.0033846677	-0.0794697258	-1.2015006510
C	-4.7593159480	1.2680677970	0.0168984417
C	-3.6187121215	0.7758947242	0.6636427837
C	-3.5796646509	-0.6135734861	1.0013053591
C	-2.4256391399	-1.1310827681	1.6647241860
H	-2.4067937536	-2.1844052733	1.9197713106
C	-1.3783628572	-0.3146413931	1.9631568357
C	-1.4116162888	1.0673954646	1.6224605915
C	-2.4961003886	1.5969799665	0.9936663712
H	-8.6729558356	-2.1380608191	-1.0365211897
H	-6.6351415539	-2.9214490740	0.1030153218

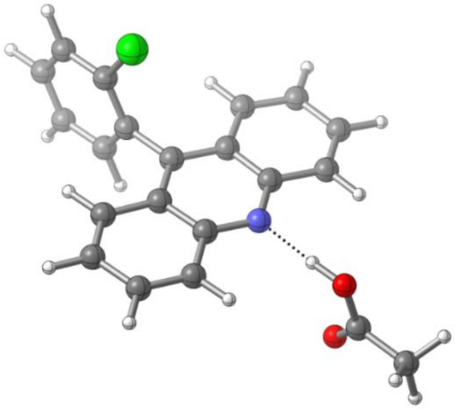

Go back to table of contents

H	-7.1131185240	1.8508960532	-1.2298343834
H	-8.9068604419	0.2455117438	-1.7045474278
H	-0.5056425530	-0.7161831392	2.4660483367
H	-0.5640549218	1.6967635432	1.8683686396
H	-2.5206728349	2.6493380249	0.7352211027
N	-4.5873924833	-1.4493321689	0.7195517053
C	-4.8636880184	2.7132623706	-0.3279774247
C	-4.4358868229	3.2188112251	-1.5536164772
C	-5.4073844309	3.6063855174	0.5965481511
C	-4.5402938436	4.5682037461	-1.8635656837
C	-5.5186400519	4.9583765526	0.3026309817
H	-5.7438520155	3.2246524532	1.5542739008
C	-5.0848092789	5.4382081105	-0.9282057726
H	-4.1993051730	4.9312894704	-2.8255489439
H	-5.9437897420	5.6357150802	1.0340504508
H	-5.1680410897	6.4923857387	-1.1663893926
Cl	-3.7448298831	2.1351741311	-2.7473790983
C	-5.0503334973	-4.2689255464	2.6912807165
O	-5.5752242927	-3.3971604451	3.3523655962
O	-4.4767682338	-4.0436176801	1.5164321247
H	-4.5435383601	-3.0712022691	1.2667944175
C	-4.9772164936	-5.7122266385	3.0987113869
H	-5.4972133278	-6.3268541519	2.3594092535
H	-3.9342692319	-6.0371400393	3.1225669711
H	-5.4316753854	-5.8513399865	4.0780428450

S1

E(TD-DFT) = -1475.15474245

Charge = 0 Multiplicity = 1

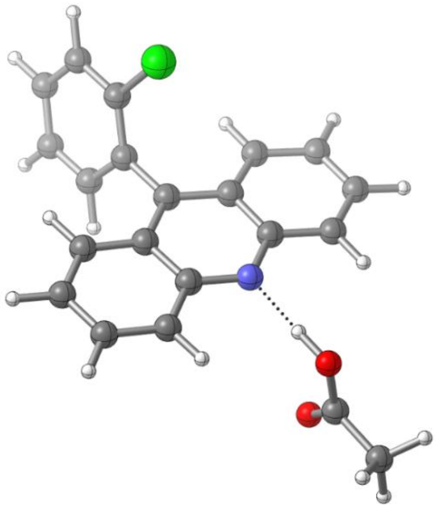

C	-7.8116197010	-1.5087381887	-1.0169594352
C	-6.6511723037	-1.9291236334	-0.3503071573
C	-5.6345606128	-1.0226518417	-0.0128861143
C	-5.7866135331	0.3594635562	-0.3671494799
C	-6.9626850050	0.7519882899	-1.0124646630
C	-7.9677928990	-0.1764758574	-1.3426443546
C	-4.7455452477	1.2674586155	-0.0189763318
C	-3.6094975177	0.7725170919	0.6808798745
C	-3.5615358940	-0.6251829674	1.0046183499
C	-2.4525932929	-1.1255748370	1.7021997353
H	-2.4276850069	-2.1816119342	1.9471653541
C	-1.3868295100	-0.2915694771	2.0751820390
C	-1.4201466378	1.0478520322	1.7455758150
C	-2.5226882746	1.5742299556	1.0456965386
H	-8.5790188326	-2.2320501999	-1.2657118802

H	-6.5263206850	-2.9717791881	-0.0801221408
H	-7.1115710539	1.7966768035	-1.2621999529
H	-8.8612275851	0.1665610716	-1.8509949147
H	-0.5436811359	-0.7074522641	2.6138269132
H	-0.6016295734	1.7038663379	2.0173583100
H	-2.5240588897	2.6259930522	0.7833060741
N	-4.5483157647	-1.4856398243	0.6527007332
C	-4.8596416785	2.7077739331	-0.3347019925
C	-4.7014082771	3.2183990261	-1.6262709784
C	-5.1372039732	3.6226524366	0.6885268345
C	-4.8296791221	4.5729106284	-1.9028330506
C	-5.2702337362	4.9788649606	0.4287827823
H	-5.2595387730	3.2463341623	1.6982791005
C	-5.1193853907	5.4541615872	-0.8693902027
H	-4.6967859861	4.9335603872	-2.9156229987
H	-5.4940202486	5.6633045786	1.2390151563
H	-5.2213355148	6.5119855081	-1.0829835509
Cl	-4.2920774855	2.1389288687	-2.9482682249
C	-5.0772339947	-4.1981909279	2.7200987927
O	-5.6011801605	-3.2951015256	3.3395188508
O	-4.4887710080	-4.0290152602	1.5446926899
H	-4.5371651989	-3.0627913335	1.2490998820
C	-5.0197550307	-5.6229126452	3.1925141029
H	-5.4965554504	-6.2745950174	2.4561949704
H	-3.9769042024	-5.9368669281	3.2840648353
H	-5.5205938119	-5.7226730319	4.1539266888

S2

E(TD-DFT) = -1475.15473250

Charge = 0 Multiplicity = 1

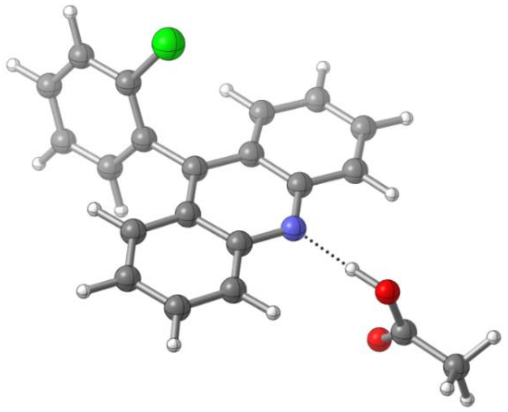

C	-7.8326700225	-1.4938926816	-0.9323174608
C	-6.6699944918	-1.9087005602	-0.2658268946
C	-5.6477471074	-1.0016235853	0.0522512318
C	-5.7966270178	0.3754424389	-0.3227846744
C	-6.9744620913	0.7627870975	-0.9681222498
C	-7.9854507678	-0.1663787017	-1.2779084361
C	-4.7492837994	1.2841295887	0.0028230743
C	-3.6107312940	0.7961205589	0.7030892558
C	-3.5652887863	-0.5967434422	1.0476844111
C	-2.4516625356	-1.0907462152	1.7423543546
H	-2.4273381044	-2.1434393848	2.0014417689
C	-1.3801440375	-0.2548077738	2.0942818497
C	-1.4121664732	1.0799865181	1.7462497909
C	-2.5187707850	1.5997461191	1.0479581087

H	-8.6043444780	-2.2179282653	-1.1652078344
H	-6.5486964355	-2.9478693183	0.0186515192
H	-7.1198774974	1.8039550147	-1.2341856183
H	-8.8803157528	0.1723680414	-1.7865982067
H	-0.5335731674	-0.6657623272	2.6313569476
H	-0.5894721004	1.7372820923	2.0018407357
H	-2.5192617591	2.6476904792	0.7707674790
N	-4.5585678758	-1.4586307141	0.7172653927
C	-4.8573018826	2.7185748514	-0.3413142671
C	-4.6941245644	3.2011959209	-1.6429833682
C	-5.1334988769	3.6555009078	0.6618634545
C	-4.8165352677	4.5499320180	-1.9485596131
C	-5.2604652098	5.0065347444	0.3731291411
H	-5.2594327796	3.3011167781	1.6790973352
C	-5.1049604717	5.4537550186	-0.9343933857
H	-4.6803981743	4.8885848768	-2.9684971797
H	-5.4831660534	5.7088390852	1.1682397957
H	-5.2024497654	6.5072239806	-1.1704244069
Cl	-4.2873013875	2.0916823816	-2.9406696102
C	-5.1092496646	-4.2734355404	2.6669577886
O	-5.7534652285	-3.4336707671	3.2615774410
O	-4.4285780116	-4.0193168377	1.5586055318
H	-4.5176021753	-3.0491588044	1.2876036003
C	-4.9941880033	-5.7083324743	3.0961704692
H	-5.3875380119	-6.3578979519	2.3100377234
H	-3.9425121200	-5.9668646793	3.2416183868
H	-5.5470409712	-5.8717614874	4.0196156185

S3

E(TD-DFT) = -1475.15456262

Charge = 0 Multiplicity = 1

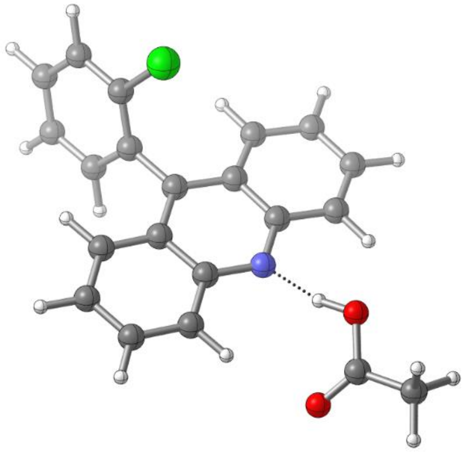

C	-8.1290107943	-1.2461436725	-0.6996493737
C	-7.0565470199	-1.6698984709	0.0986479912
C	-5.9654331866	-0.8279617697	0.3630779269
C	-5.9504100938	0.4905050381	-0.2036504269
C	-7.0409300580	0.8903475376	-0.9813079965
C	-8.1228746582	0.0269437332	-1.2335042702
C	-4.8357435124	1.3351510583	0.0715126352
C	-3.8031193350	0.8500882267	0.9210818187
C	-3.9229978494	-0.4763444568	1.4569638209
C	-2.9236845198	-0.9603699548	2.3139299612
H	-3.0483677849	-1.9512347224	2.7383566676
C	-1.7978346294	-0.1838461406	2.6325303037
C	-1.6622996823	1.0783476006	2.0924020519

C	-2.6566748670	1.5882597754	1.2354323134
H	-8.9566097511	-1.9199243258	-0.8871574155
H	-7.0617569045	-2.6632442814	0.5330833036
H	-7.0594474591	1.8899229472	-1.4011534872
H	-8.9460247714	0.3718622578	-1.8481382507
H	-1.0421871286	-0.5845464979	3.2978275395
H	-0.7939586195	1.6860182621	2.3183915019
H	-2.5233702676	2.5749342563	0.8070099021
N	-4.9677699355	-1.2888920560	1.1596080838
C	-4.7755201432	2.7096240912	-0.4712847015
C	-4.4958673916	2.9839290892	-1.8133194467
C	-5.0054787349	3.8027937822	0.3731537125
C	-4.4597031294	4.2807879973	-2.3075632836
C	-4.9749187275	5.1047050626	-0.1051613802
H	-5.2223363569	3.6113839921	1.4184185481
C	-4.7043853433	5.3438364716	-1.4481237466
H	-4.2343778905	4.4551832006	-3.3527418014
H	-5.1644600430	5.9313667299	0.5698768909
H	-4.6775838242	6.3573731454	-1.8315609094
Cl	-4.1405321755	1.6674588219	-2.9182003537
C	-4.5078109085	-4.5218052831	2.5682686399
O	-4.1461777331	-3.8642850310	3.5239087174
O	-4.9034687251	-3.9839629493	1.4252678023
H	-4.8883047791	-2.9759464732	1.4546084450
C	-4.5548961314	-6.0218881558	2.5466636082
H	-5.5758274837	-6.3541554095	2.3429091874
H	-3.9186487205	-6.3973387007	1.7413513291
H	-4.2189039303	-6.4235497264	3.5009741415

S1-H

E(TD-DFT) = -1475.15123655

Charge = 0 Multiplicity = 1

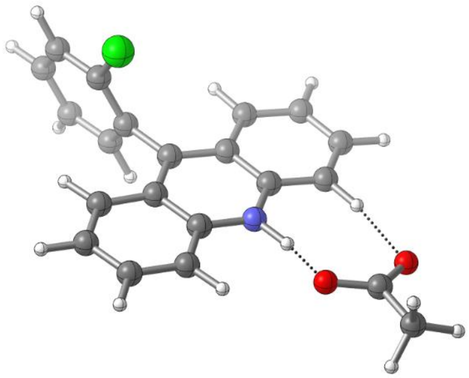

C	-7.7371286542	-1.3981319152	-1.2656877776
C	-6.6236008876	-1.8463552764	-0.5374953726
C	-5.6268431795	-0.9419877226	-0.1525309086
C	-5.7267532206	0.4427213880	-0.4765138467
C	-6.8401079510	0.8471724458	-1.2212993018
C	-7.8420207875	-0.0656082104	-1.6064918178
C	-4.6936269468	1.3317788801	-0.0650680912
C	-3.5822428033	0.8099608839	0.6639914994
C	-3.5306339234	-0.5861114311	0.9449600929
C	-2.4557984426	-1.1354471513	1.6523859941
H	-2.4502544369	-2.2025968077	1.8460782512
C	-1.4136185404	-0.3149092268	2.1017466734

Go back to table of contents

C	-1.4551705140	1.0429786285	1.8503738307
C	-2.5338888632	1.6012156553	1.1416000854
H	-8.5029198807	-2.1061971392	-1.5577238637
H	-6.5133426101	-2.8960878710	-0.2620305970
H	-6.9356935982	1.8856437576	-1.5154184521
H	-8.6910341000	0.2929661019	-2.1760094803
H	-0.5856738207	-0.7515054760	2.6464278640
H	-0.6585605503	1.6906380798	2.1964081802
H	-2.5502790320	2.6708753417	0.9667520184
N	-4.5443818716	-1.4000349341	0.5341284488
C	-4.7928497358	2.7791841793	-0.3538051820
C	-4.0269431892	3.4061315496	-1.3404000749
C	-5.6810683891	3.5757172599	0.3780394064
C	-4.1206200893	4.7689425045	-1.5870243606
C	-5.7850468874	4.9394892968	0.1449812460
H	-6.2854102547	3.1059351946	1.1463107061
C	-5.0018846412	5.5365562622	-0.8365986883
H	-3.5149434131	5.2214152416	-2.3628247266
H	-6.4756062256	5.5350372394	0.7308058732
H	-5.0764788743	6.6011858446	-1.0261809275
Cl	-2.9342847218	2.4549517247	-2.3297036035
H	-4.4569350042	-2.4545924193	0.7570629228
C	-4.8414939777	-4.8827995966	0.9490776216
O	-5.9793783646	-4.8157300770	0.4513721644
O	-4.0751410870	-3.8877484653	1.1583927134
C	-4.2752296680	-6.2409419665	1.3367278811
H	-3.4446373556	-6.4853015393	0.6671746433
H	-3.8756866262	-6.2072203246	2.3534548979
H	-5.0316098805	-7.0232129096	1.2633830584

S2-H

E(TD-DFT) = -1475.15128689

Charge = 0 Multiplicity = 1

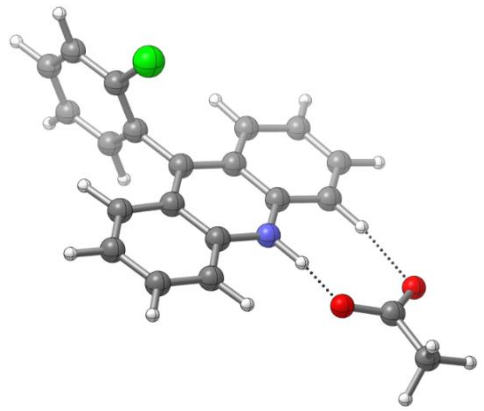

C	-7.7481287320	-1.3895666360	-1.2540573343
C	-6.6242673869	-1.8413149498	-0.5441917354
C	-5.6244861128	-0.9380645173	-0.1648033861
C	-5.7305313584	0.4486256733	-0.4777170108
C	-6.8542824939	0.8568170260	-1.2047343927
C	-7.8597778534	-0.0546482514	-1.5832502022
C	-4.6934624077	1.3364718835	-0.0733816214
C	-3.5734605479	0.8118058077	0.6402164920
C	-3.5158329462	-0.5863206635	0.9100135401
C	-2.4322917350	-1.1381607428	1.6022267875
H	-2.4227653771	-2.2065484407	1.7888615997

C	-1.3878999820	-0.3182687126	2.0476212489
C	-1.4362448930	1.0417078136	1.8088209412
C	-2.5236314665	1.6023801612	1.1154877238
H	-8.5163024807	-2.0966922843	-1.5419307913
H	-6.5073152978	-2.8934486625	-0.2809976673
H	-6.9548151906	1.8975861312	-1.4893778088
H	-8.7168369282	0.3068726996	-2.1387314536
H	-0.5531607917	-0.7570320971	2.5801252347
H	-0.6386299416	1.6891285031	2.1530523479
H	-2.5457164629	2.6735934080	0.9511854200
N	-4.5325890088	-1.3992143830	0.5048280411
C	-4.7990585932	2.7842506679	-0.3564028998
C	-4.0399111052	3.4192073465	-1.3434468557
C	-5.6927028322	3.5726174213	0.3778152845
C	-4.1451842390	4.7815886961	-1.5873369366
C	-5.8079523979	4.9359618895	0.1476346123
H	-6.2922753103	3.0965982147	1.1460027231
C	-5.0312217038	5.5409697033	-0.8341634386
H	-3.5450094677	5.2401761549	-2.3638444848
H	-6.5022511392	5.5249760556	0.7356449539
H	-5.1144852840	6.6052271826	-1.0222746211
Cl	-2.9418034609	2.4800203722	-2.3382255116
H	-4.4496611112	-2.4525783963	0.7355040565
C	-4.8549320537	-4.8746764735	0.9707298087
O	-5.9595196663	-4.8200083397	0.4017240768
O	-4.0893286790	-3.8786639475	1.1790255966
C	-4.3367971744	-6.2150188793	1.4716081171
H	-3.3872987902	-6.4464134963	0.9802705873
H	-4.1411414241	-6.1559584823	2.5459879247
H	-5.0496656815	-7.0168044537	1.2757819127

S3-H

E(TD-DFT) = -1475.15127963

Charge = 0   Multiplicity = 1

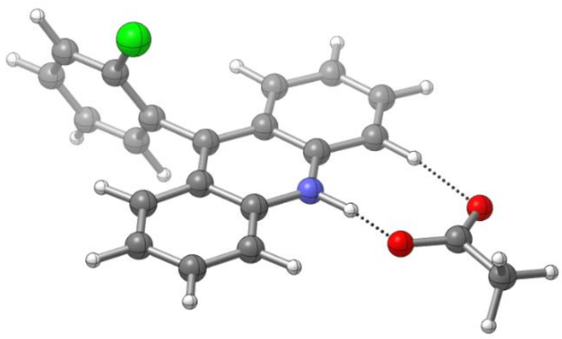

C	-7.66242000	-1.41464000	-1.39803000
C	-6.56331000	-1.86064000	-0.64695000
C	-5.58951000	-0.95030000	-0.21980000
C	-5.69841000	0.43788000	-0.52472000
C	-6.79630000	0.84050000	-1.29320000
C	-7.77555000	-0.07810000	-1.72001000
C	-4.68874000	1.33334000	-0.07118000
C	-3.59169000	0.81404000	0.68098000
C	-3.52956000	-0.58567000	0.94149000
C	-2.46804000	-1.13189000	1.67127000

H	-2.45470000	-2.20156000	1.85035000
C	-1.44977000	-0.30516000	2.16211000
C	-1.50262000	1.05603000	1.93118000
C	-2.56867000	1.61122000	1.20141000
H	-8.41043000	-2.12742000	-1.72272000
H	-6.44663000	-2.91342000	-0.38629000
H	-6.89669000	1.88283000	-1.57193000
H	-8.61348000	0.27932000	-2.30644000
H	-0.63148000	-0.73970000	2.72286000
H	-0.72503000	1.70863000	2.30971000
H	-2.59510000	2.68324000	1.04296000
N	-4.52058000	-1.40582000	0.48964000
C	-4.80020000	2.78286000	-0.34312000
C	-4.00916000	3.43727000	-1.29160000
C	-5.73231000	3.55290000	0.36236000
C	-4.12008000	4.80105000	-1.52495000
C	-5.85392000	4.91735000	0.14207000
H	-6.35707000	3.06192000	1.10048000
C	-5.04483000	5.54201000	-0.80055000
H	-3.49400000	5.27474000	-2.27138000
H	-6.57866000	5.49185000	0.70725000
H	-5.13278000	6.60729000	-0.98052000
Cl	-2.86140000	2.52144000	-2.25134000
H	-4.43434000	-2.46064000	0.71262000
C	-4.83026000	-4.88625000	0.92408000
O	-5.91961000	-4.83545000	0.32609000
O	-4.07426000	-3.88736000	1.15292000
C	-4.31853000	-6.22575000	1.43350000
H	-3.37725000	-6.46915000	0.93185000
H	-4.10886000	-6.15979000	2.50462000
H	-5.03952000	-7.02346000	1.25114000

12. Parameterization of acridinyl photocatalst structural/electronic properties

A set of 22 substituted acridinyl photocatalysts was devised to probe the role of substitution on photocatalyst performance (Figure S8). The CREST utility and manual conformational construction were used to generate multiple thermally accessible conformers for each photocatalyst and protonated radical, **HA**, each of which was initially optimized at the ωB97X-D / 6-311+G** / SMD (DCM) level of theory using Gaussian 16. For thermochemical calculations, Boltzmann averaging was applied across obtained geometries to obtain representative free energies across the range of thermally accessible conformers. In total, the structural dataset consisted of 222 unique geometries.

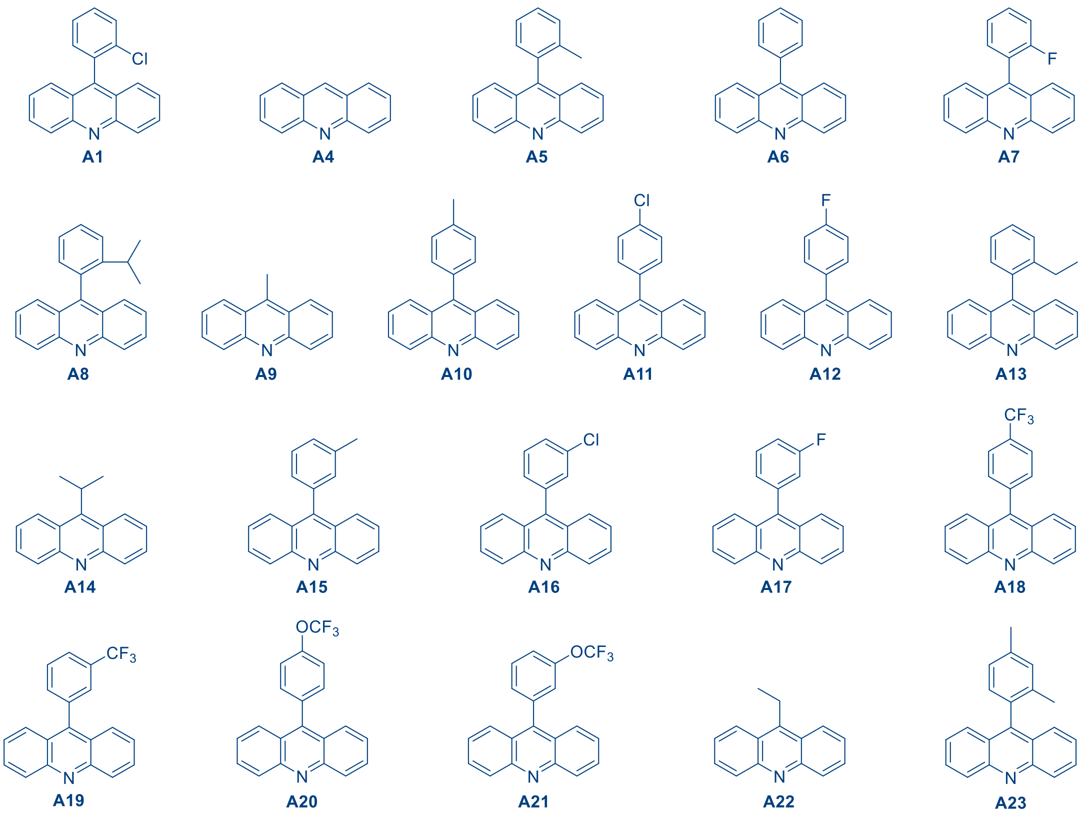

Figure S8. Set of substituted acridinyl photocatalysts.

13. Description of parameters

13a. Buried Volume

To investigate the role of bulky substituents proximal to the 9-acridinyl carbon atom, truncated C9-Ligand geometries were generated from the lowest energy ground state conformation obtained for each photocatalyst (Figure S9). The $\%V_{bur}$ parameter reflects the buried volumes calculated with respect to the central C9 atom using a sphere of radius 4.5 Å calculated using Paton's DBSTEP.[50]

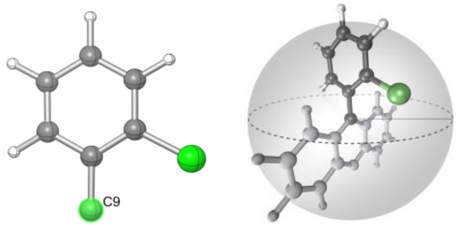

Figure S8. Percent buried volume parameter (%V_{bur}) calculation. Percent buried were calculated with respect to a sphere of 4.5 Å radius centered on the C9 atom of truncated C9-substituted acridines.

13b. Mulliken and NBO Charges/Spin Densities

To probe the capability of C9 substitution to stabilize acridinyl radical **HA**, the charge and spin density of two regions of the photocatalysts was parameterized: the C9 carbon and the sum of atomic contributions for each of the atoms comprising the substituent in the C9 position (Figure S10).

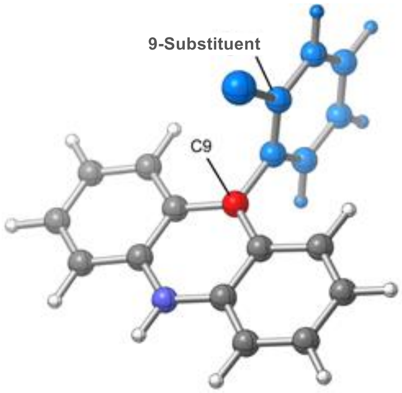

Figure S10. Atomic/Ligand Charges and Spin Densities. The charge and spin density of C9 (red) and the sum of atoms comprising the 9-substituent (blue) were parameterized to probe the ability of substitution to stabilize acridinyl radical **HA**. Both Mulliken- and NBO-derived values were obtained from the lowest energy **HA** conformer of each photocatalyst.

Mulliken charges and spin densities were extracted from population analyses performed on the lowest energy conformer of **HA**, the protonated acridine radical form of each photocatalyst. The ***Mulliken C9 Charge*** parameter reflects the Mulliken charge of the C9 carbon, shown in red in Figure S10. The ***Mulliken 9-Substituent Charge*** parameter was calculated as the sum of atom-centered charges comprising the substituent connected to the C9 position of

acridine, shown in blue in Figure S10. The ***Mulliken C9 Spin Density*** and ***Mulliken 9-Substituent Spin Density*** parameters represent the value of spin densities for the same atom groups.

NBO calculations for the photocatalysts proved challenging for several photocatalysts at the ωB97X-D / 6-311+G** / SMD (DCM) level of theory; for several photocatalysts, there existed sufficiently strong linear dependence within the employed 6-311+G** basis set such that problematic overlap matrices contained a negative eigenvalue leading to a failure of reliable NOs and natural charge/spin density values. Accordingly, the geometries of each of the **HA** structures were re-optimized using the ωB97X-D / def2-TZVP / SMD (DCM) level of theory. NBO analyses of the newly obtained def2-TZVP geometries exhibited no such symptoms. The ***NBO C9 Spin Density*** parameter reflects the spin density of the C9 atom, red in Figure S10, while the ***NBO 9-Substituent Spin Density*** parameter reflects the sum of atom spin densities comprising the substituent in the C9 position, blue in **Figure S10**. The ***ρ*** parameter represents the sum of the ***NBO C9 Spin Density*** and ***NBO 9-Substituent Spin Density*** parameters and replaced the individual spin density parameters during model development.

Table S6 DFT-derived Steric and Electronic Parameters.

Acridine	Relative Rate	%V_{bur}	Mulliken C9 Charge	Mulliken 9-Substituent Charge	Mulliken C9 Spin Density	Mulliken 9-Substituent Spin Density	NBO C9 Spin Density	NBO 9-Substituent Spin Density	ϱ
**A1**	3.072	22.19	1.288287	-0.237581	0.546839	-0.003808	0.49746	0.00613	0.50359
**A4**	1	5.68	0.421182	0.170555	0.56774	-0.028802	0.50757	-0.01586	0.49171
**A5**	3.256	22.28	1.233517	-0.48252	0.558353	-0.016807	0.4988	0.00496	0.50376
**A6**	1.608	18.63	1.10213	-0.278397	0.557421	-0.021294	0.49848	0.01184	0.51032
**A7**	2.504	20	1.274619	-0.47678	0.548	-0.014058	0.4964	0.01185	0.50825
**A8**	3.424	25.32	1.08768	0.251052	0.547804	-0.025879	0.49925	0.00681	0.50606
**A9**	1.032	9.64	1.053403	0.064338	0.535712	0.012849	0.49542	0.01728	0.5127
**A10**	1.816	18.6	1.059682	-0.248811	0.561815	-0.027815	0.49884	0.01239	0.51123
**A11**	2.176	18.63	1.096072	-0.30736	0.549092	-0.010586	0.49711	0.01367	0.51078
**A12**	2.168	18.64	1.151487	-0.364224	0.549413	-0.010594	0.49826	0.01159	0.50985
**A13**	3.368	24.77	1.165824	-0.262085	0.555929	-0.016946	0.49828	0.00748	0.50576
**A14**	1.328	17.65	0.98782	0.112906	0.57543	-0.005209	0.51756	0.01025	0.52781
**A15**	2.088	18.84	1.208316	-0.472292	0.550003	-0.00922	0.4988	0.01132	0.51012
**A16**	2.472	18.8	1.183475	-0.405737	0.543719	-0.004252	0.49645	0.01389	0.51034
**A17**	2.456	18.71	1.129639	-0.316671	0.54625	-0.007614	0.49718	0.01277	0.50995
**A18**	2.376	18.64	1.240661	-0.376506	0.539958	-0.000769	0.49564	0.01551	0.51115
**A19**	2.328	19.07	1.281995	-0.677112	0.543711	-0.001127	0.49601	0.01513	0.51114

**A20**	2.176	18.64	1.132015	-0.353046	0.548798	-0.010869	0.49714	0.0126	0.50974
**A21**	2.184	19.18	1.200109	-0.411723	0.538469	0.000754	0.49549	0.0173	0.51279
**A22**	1.336	13.63	0.904242	0.126729	0.547268	0.002331	0.49813	0.01432	0.51245
**A23**	3.224	22.25	1.128624	-0.341664	0.56348	-0.027045	0.49923	0.00503	0.50426

14. Photophysical Parameters derived using TD-DFT

TD-DFT calculations were performed at the ωB97X-D / 6-311+G** / SMD (DCM) level of theory to obtain parameters describing the photophysical properties of each photocatalyst. From the most stable ground state conformer of each photocatalyst, the 7 lowest singlet excited states were calculated using Gaussian 16. The λ_{max}, excitation energy E_{abs}, and oscillator strength f of the first singlet excited state were captured as parameters from these calculations. A second series of TD-DFT calculations considering the first 40 excited states of each photocatalyst was performed to obtain the full simulated spectrum for each photocatalyst. The spectral data were extracted from each calculation using Gaussum[51] and plotted to obtain the values of molar absorptivity at 400 nm, ε_{400}, and molar absorptivity at λ_{max}, ε_{max}.

Table S7. TD-DFT-derived Photophysical Parameters.

Structure	λ_{max}, nm	E_{abs}, eV	f	ε_{400}	ε_{max}
**A1**	343.54	3.6090	0.1768	65.4675	12905.4535
**A4**	336.71	3.6823	0.1183	8.7107	8753.0673
**A5**	342.47	3.6203	0.1820	53.2809	13248.5212
**A6**	341.94	3.6259	0.1818	53.2809	13248.5212
**A7**	342.98	3.6149	0.1776	58.2083	12957.9753
**A8**	343.21	3.6125	0.1823	62.8002	13274.3325
**A9**	341.73	3.6281	0.1534	38.0709	11174.2945
**A10**	341.91	3.6263	0.1897	48.9196	13823.7131
**A11**	342.36	3.6214	0.1892	54.1215	13777.0616
**A12**	343.39	3.6106	0.1859	66.6053	13538.4248
**A13**	343.12	3.6134	0.1835	62.0455	13369.3868
**A14**	342.37	3.6214	0.1634	46.7413	11874.2568
**A15**	341.86	3.6267	0.186	47.5591	13557.1836
**A16**	342.59	3.6190	0.1845	55.5072	13428.2855
**A17**	342.27	3.6224	0.1788	50.0794	13033.9929

**A18**	342.49	3.6201	0.1844	54.2109	13425.4739
**A19**	342.55	3.6195	0.1809	53.8563	13172.8029
**A20**	342.37	3.6214	0.1830	52.3479	13331.6264
**A21**	344.36	3.6004	0.1896	83.6667	13777.0570
**A22**	342.24	3.6227	0.1605	44.6701	11678.3894
**A23**	342.45	3.6205	0.1899	55.3604	13810.0392

15. Correlation-based Parameter Exclusion

A correlation matrix was calculated amongst collected parameters to perform a preliminary exclusion of highly correlated parameters prior to stepwise linear regression. Using an exclusionary cutoff of r = 0.89, two pairs of highly correlated parameters were observed: oscillator strength f with molar absorptivity at λ_{max}, ε_{max} (correlation coefficient = 0.99) and λ_{max} with the molar absorptivity at 400nm, ε_{400} (correlation coefficient = 0.94). Because the ε_{max} and ε_{400} parameters exhibited greater univariate correlation with the desired Relative Rate regressand than their correlated counterparts, parameters reflecting oscillator strength f and λ_{max} were excluded prior to executing the forward stepwise linear regression paradigm.

Table S8: Correlation Matrix of Collected Parameters

	Relative Rate	%V_{bur}	Mulli ken C9 Charge	Mulliken 9-Substituent Charge	Mulliken C9 SD	Mulliken 9-Substituent SD	NBO C9 SD	NBO 9-Substituent SD	ρ	λ_{max}, nm	E_{abs}, eV	f	ε_{400}	ε_{max}
**Relative Rate**	1													
%V_{bur}	0.8809	1												
Mulliken C9 Charge	0.6041	0.7436	1											
Mulliken 9-Substituent Charge	-0.3800	-0.4060	-0.7042	1										
Mulliken C9 SD	-0.1113	-0.0392	-0.4850	0.2901	1									
Mulliken 9-Substituent SD	-0.2908	-0.2373	0.3136	-0.0993	-0.6609	1								
NBO C9 SD	-0.3519	-0.2449	-0.5493	0.5193	0.8131	-0.24295	1							
NBO 9-Substituent SD	-0.0090	0.2723	0.6885	-0.3739	-0.6128	0.6388	-0.4778	1						
ρ	-0.2832	0.1100	0.3326	-0.0089	-0.0440	0.51545	0.2499	0.7311	1					
λ_{max}, nm	0.5445	0.7542	0.8576	-0.3919	-0.4412	0.3458	-0.4112	0.7339	0.4896	1				
E_{abs}, eV	-0.5433	-0.7540	-0.8591	0.3925	0.4414	-0.3471	0.4123	-0.7371	-0.4922	-0.99997	1			
f	0.6171	0.8218	0.8404	-0.6448	-0.2735	-0.02495	-0.4905	0.6243	0.3072	0.8064	-0.8078	1		
ε_{400}	0.5985	0.7707	0.7902	-0.4516	-0.4085	0.1838	-0.4332	0.5823	0.3054	0.9414	-0.9394	0.8145	1	
ε_{max}	0.6215	0.8227	0.8383	-0.6500	-0.2716	-0.0362	-0.4944	0.6144	0.29325	0.7992	-0.8006	0.9998	0.8113	1

SD = Spin Density.

16. Parameter Standardization

To enable the relative comparison of regressor coefficients in the multivariate linear regression model, each of the dependent variables was standardized/z-score normalized to ensure a standard normal distribution, that is, $\mu = 0$ and $\sigma = 1$:

$$z = \frac{x - \mu}{\sigma} \tag{20}$$

17. Model Development

The ***Relative Rate = 2.3 + 0.68(%V_{Bur}) − 0.30ρ*** model was developed using forward stepwise multivariate linear regression[52] performed in Microsoft Excel. The relative rate of the reaction was treated as regressand with the remaining parameters serving as regressors. After each regression, the regressor exhibiting the highest p-value was systematically eliminated to produce a reduced dataset. This process was repeated until all regressors exhibited p-values < 0.05. The resulting two parameter model contained only **%V_{Bur}** and ρ parameters:

Table S9. Regression Statistics of Initial Stepwise Multivariate Linear Regression Model.

Regression Statistics				
Multiple R	0.9603			
R Square	0.9223			
Adjusted R Square	0.9136			
Standard Error	0.2162			
Observations	21			
	Coefficients	*Standard Error*	*t Stat*	*p-value*
Intercept	22.0131	3.8634	5.6978	2.1048E-05
ρ	-44.3432	7.6197	-5.8196	1.6342E-05
%V_{bur}	0.15217	0.010897	13.9637	4.2441E-11

18. Developing the %V_{bur} + ρ model with unified ωB97X-D / def2-TZVP / SMD (DCM) parameters

Models were developed initially using parameters obtained from both ωB97X-D / 6-311+G** / SMD (DCM) and ωB97X-D / def2-TZVP / SMD (DCM) levels of theory owing to the need to calculate NBO parameters using the def2-TZVP basis set. Because a model relying on a minimal number of DFT calculations is favored over one in which multiple calculations using

different computational methods is required, the geometries of all structures were re-optimized using ωB97X-D / def2-TZVP / SMD (DCM) to unify the parameters under a single computational method. Because NBO spin density parameters were previously calculated using ωB97X-D / def2-TZVP / SMD (DCM), only the **%V_{Bur}** parameter needed to be reassessed using the def2-TZVP-obtained geometries resulting in the final reduced dataset used for final model development and validation:

Table S10. Final ωB97X-D / def2-TZVP / SMD (DCM) parameters.

Structure	Relative Rate	%V_{bur}	ϱ
**A1**	3.072	22.17	0.50359
**A4**	1	5.68	0.49171
**A5**	3.256	22.27	0.50376
**A6**	1.608	18.62	0.51032
**A7**	2.504	19.98	0.50825
**A8**	3.424	25.31	0.50606
**A9**	1.032	9.63	0.5127
**A10**	1.816	18.59	0.51123
**A11**	2.176	18.61	0.51078
**A12**	2.168	18.63	0.50985
**A13**	3.368	24.72	0.50576
**A14**	1.328	17.62	0.52781
**A15**	2.088	18.84	0.51012
**A16**	2.472	18.8	0.51034
**A17**	2.456	18.71	0.50995
**A18**	2.376	18.63	0.51115
**A19**	2.328	19.07	0.51114
**A20**	2.176	18.63	0.50974
**A21**	2.184	19.19	0.51279
**A22**	1.336	13.61	0.51245
**A23**	3.224	22.23	0.50426

19. Regression model development and statistical analyses

The predictive ability of regression model based on $\%V_{bur}$ and ρ parameters was assessed using Python 3.9.5 and scikit-learn.[53] A .csv file containing the final data table, above, was read in as data input to the Python script. The script was used to perform a 16/5 training/test splits holding out 5 data points as a test set for external validation to measure the predictive performance against unseen photocatalysts. The 16-photocatalyst training set was subjected to regression analysis to produce candidate models. Owing to the moderate size of the training set, internal validation was performed with both K-fold (k=4) and leave-one-out-cross validation methods.

Finally, the produced model was externally validated against the withheld test set by evaluating the out-of-sample R^2. Each of the test set photocatalysts were standardized with respect to the mean and standard deviation of the training set to allow for out-of-sample validation. The out-of-sample R^2 was calculated according to

$$R^2_{OOS} = 1 - \frac{RSS_{Test}}{TSS_{OOS}} \quad (21)$$

where RSS_{Test} is the residual sum of squares for the model-predicted test set:

$$RSS_{Test} = \sum_{i=1}^{n}\left(y_i - y_{i,pred}\right)^2 \quad (22)$$

and TSS_{OOS} is the total sum of squares with respect to the training mean:

$$TSS_{OOS} = \sum_{i=1}^{n}(y_i - \bar{y}_{train})^2 \quad (23)$$

21. Optimized Geometries (ωB97X-D / def2-TZVP / SMD(DCM))

*For each structure, the lowest energy conformer obtained is provided.

Cyclohexyl radical

E(UwB97XD) = -235.231765447

Charge = 0 Multiplicity = 2

C	-2.8273745454	0.0732658977	-0.0050886389
C	-1.2947503419	0.0326932811	0.0216842870
C	-0.7020349048	1.3868861359	0.1982107120
C	-1.2931933721	2.3041114646	1.2108605044
C	-2.8258183368	2.3045596523	1.1631515954
C	-3.3815761531	0.8832783910	1.1635937627
H	0.2845438289	1.5945602598	-0.1999543442
H	-0.9879443986	-0.6109936761	0.8642462615

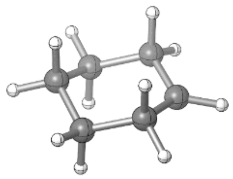

H	-0.9071707951	-0.4468499529	-0.8801563300
H	-3.1579310730	0.5298252769	-0.9439841076
H	-3.2249562567	-0.9443412946	0.0129479986
H	-0.9859768159	1.9781825546	2.2197298362
H	-0.9046375424	3.3180841223	1.0909080714
H	-3.2223970976	2.8699544087	2.0099035624
H	-3.1562677388	2.8164246490	0.2531857118
H	-3.1133690720	0.3899184689	2.1053438383
H	-4.4732571347	0.9074634309	1.1188112788

A1

E(RwB97XD) = -1246.25095314

Charge = 0 Multiplicity = 1

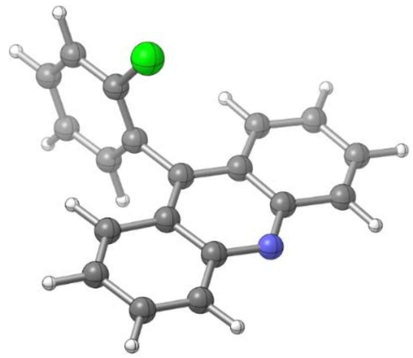

C	-7.8923968537	-1.3541599907	-0.8746353959
C	-6.7765811468	-1.7993137861	-0.2434424072
C	-5.7047456253	-0.9067558325	0.0567088271
C	-5.8301528164	0.4675410418	-0.3159031034
C	-7.0196767625	0.8930630100	-0.9794860573
C	-8.0156400861	0.0113370164	-1.2498916122
C	-4.7732946597	1.3313821053	-0.0158452906
C	-3.6426353504	0.8228237332	0.6293094855
C	-3.6213669266	-0.5687706403	0.9559100398
C	-2.4725245764	-1.1015519466	1.6131721190
H	-2.4752602027	-2.1573517670	1.8538380401
C	-1.4177942703	-0.3043610198	1.9191968320
C	-1.4367916151	1.0786897928	1.5915047885
C	-2.5122564089	1.6249661468	0.9690269174
H	-8.6998835830	-2.0402215016	-1.0988423074
H	-6.6645809605	-2.8361381917	0.0482828637
H	-7.1176391602	1.9319495491	-1.2676767594
H	-8.9130591447	0.3443923692	-1.7558025430
H	-0.5498314528	-0.7192178971	2.4167281447
H	-0.5835064733	1.6959451462	1.8428318622
H	-2.5220597335	2.6787593250	0.7213397037
N	-4.6286617117	-1.3960818519	0.6740675821
C	-4.8625050067	2.7769903876	-0.3547015361

C	-4.4259147906	3.2858702970	-1.5733118384
C	-5.4008730193	3.6694668794	0.5688435736
C	-4.5177352354	4.6359227768	-1.8725778332
C	-5.4993415087	5.0210466102	0.2847437476
H	-5.7449742061	3.2865775987	1.5217593355
C	-5.0569267808	5.5039180396	-0.9378440663
H	-4.1691443954	5.0004722363	-2.8295718943
H	-5.9213756825	5.6962704070	1.0178886608
H	-5.1295046353	6.5588521022	-1.1695663415
Cl	-3.7434941486	2.2147651843	-2.7670058363

HA1

E(UwB97XD) = -1246.84912241

Charge = 0 Multiplicity = 2

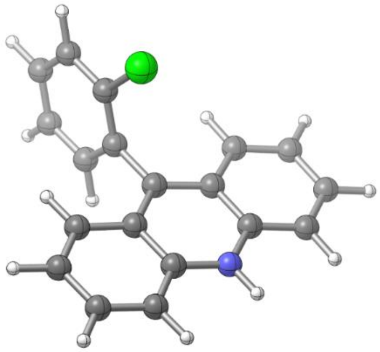

C	-7.9401443960	-1.3463511450	-0.9060268950
C	-6.8016375170	-1.7977297220	-0.2608931940
C	-5.7652624840	-0.9157047920	0.0300853940
C	-5.8565901250	0.4531893600	-0.3241631750
C	-7.0290284760	0.8756062670	-0.9809860650
C	-8.0516469600	-0.0051904340	-1.2671703500
C	-4.7752202460	1.3248661260	-0.0101980430
C	-3.6171883990	0.8178094440	0.6453299520
C	-3.5619966890	-0.5571302470	0.9838173690
C	-2.4464967920	-1.0893139970	1.6235932830
H	-2.4328508690	-2.1447279100	1.8708539870
C	-1.3694428170	-0.2778072860	1.9360723300
C	-1.3989981450	1.0765778350	1.6100924500
C	-2.5020051870	1.6119999410	0.9775049440
H	-8.7408399220	-2.0402982210	-1.1286506850
H	-6.7039117440	-2.8394163980	0.0226467390
H	-7.1215965140	1.9163082820	-1.2650575530
H	-8.9407322060	0.3484343290	-1.7739644560
H	-0.5056695710	-0.7013877640	2.4327766040
H	-0.5560434250	1.7116058550	1.8520266340
H	-2.5181882460	2.6650020430	0.7260733120
N	-4.6324110900	-1.3588366400	0.6687889740

C	-4.8640337160	2.7658956260	-0.3478314040
C	-4.4407323450	3.2783676120	-1.5719651740
C	-5.3879181920	3.6697621520	0.5757977650
C	-4.5299390830	4.6279757230	-1.8750794570
C	-5.4850660250	5.0217693490	0.2914171440
H	-5.7231106370	3.2913508940	1.5339486830
C	-5.0549342900	5.5012017310	-0.9369311450
H	-4.1904859310	4.9887375910	-2.8369059050
H	-5.8964255030	5.7001163060	1.0280844910
H	-5.1259180470	6.5559947040	-1.1703570890
Cl	-3.7745781360	2.2043607840	-2.7745630850
H	-4.5796427820	-2.3350049660	0.9138379390

H_2A1

E(RwB97XD) = -1247.47156146

Charge = 0 Multiplicity = 1

C	-8.1369853546	-0.9303755915	-0.6784809564
C	-7.1286446617	-1.2432464709	0.2127233989
C	-5.8857849352	-0.6108581857	0.1253050665
C	-5.6572176809	0.3401094697	-0.8715508700
C	-6.6886543453	0.6421755924	-1.7519041408
C	-7.9237996245	0.0202616400	-1.6692923451
C	-4.3203621212	1.0522184141	-0.9931048444
C	-3.3448881099	0.6708127888	0.1078610046
C	-3.6692183908	-0.2944134174	1.0637978723
C	-2.7501784523	-0.6190188292	2.0650627229
H	-3.0120222046	-1.3751729938	2.7966372147
C	-1.5228665373	0.0131821340	2.1199799331
C	-1.1929593850	0.9817621721	1.1798561559
C	-2.1073550540	1.2972539131	0.1880797477
H	-9.0945428919	-1.4303065813	-0.5976892899
H	-7.2900542515	-1.9836719663	0.9881523507
H	-6.5121720335	1.3812282352	-2.5259186170
H	-8.7101767607	0.2725959752	-2.3689673102
H	-0.8214286598	-0.2507979919	2.9020720275
H	-0.2346573424	1.4834891217	1.2186797759

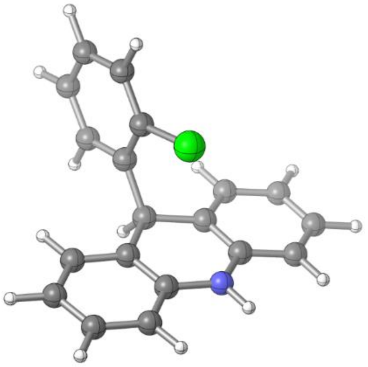

H	-1.8550972386	2.0474549669	-0.5535192268
N	-4.8886432686	-0.9370682231	1.0202258322
H	-5.1009197356	-1.6015027866	1.7448572608
H	-4.5216517742	2.1210493690	-0.8779213176
C	-3.7132627799	0.9185173432	-2.3831255257
C	-3.5520376153	2.0651108763	-3.1582148263
C	-3.3002101486	-0.2859695521	-2.9509474229
C	-3.0082820013	2.0232733957	-4.4320349336
C	-2.7539585909	-0.3486052900	-4.2229457108
C	-2.6069196354	0.8104240895	-4.9671772636
H	-2.8996428376	2.9369832444	-5.0024999419
H	-2.4458093831	-1.3042951011	-4.6259317745
H	-2.1802943609	0.7584807832	-5.9607569628
H	-3.8656847487	3.0155030061	-2.7422649746
Cl	-3.4542433643	-1.7890845797	-2.0780511087

HCyA1

E(RwB97XD) = -1482.15959572

Charge = 0 Multiplicity = 1

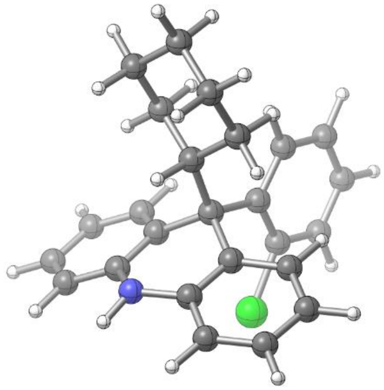

C	-8.2104130981	-0.9679736826	-0.5728118736
C	-7.0165990871	-1.5410209531	-0.1763115157
C	-5.8411049619	-0.7904608938	-0.1816945170
C	-5.8543893831	0.5423792945	-0.5983220016
C	-7.0694845507	1.0992034370	-0.9764062487
C	-8.2432083362	0.3625462922	-0.9682286530
C	-4.5265498699	1.2967079902	-0.7233788788
C	-3.5468703668	0.7703469771	0.3305767800
C	-3.6179927943	-0.5706910976	0.7135165253
C	-2.6642459262	-1.1109437834	1.5758795839
H	-2.7409947603	-2.1540316732	1.8619820492
C	-1.6444266515	-0.3194334853	2.0700175499
C	-1.5737979357	1.0212467369	1.7160866651
C	-2.5220063892	1.5483060393	0.8538826147
H	-9.1178312290	-1.5594075586	-0.5630692241
H	-6.9834235955	-2.5732565129	0.1541188770
H	-7.0969700234	2.1324141863	-1.3003021816

H	-9.1737696064	0.8222463084	-1.2757723601
H	-0.9102606192	-0.7486060880	2.7409229275
H	-0.7822729822	1.6510029895	2.1017343570
H	-2.4545022054	2.5909691091	0.5683501327
N	-4.6495379126	-1.3661510140	0.2353940695
C	-4.7635127756	2.7969064383	-0.5031896665
C	-5.2953366452	3.3024988308	0.6937884839
C	-4.4674296192	3.7521865190	-1.4728355899
C	-5.5105769848	4.6560873423	0.8987552256
C	-4.6762588338	5.1098313042	-1.2860453893
H	-4.0560474908	3.4360465841	-2.4149631950
C	-5.2011089749	5.5697275512	-0.0933889090
H	-5.9224999467	4.9890491718	1.8419811462
H	-4.4248442584	5.8012223659	-2.0803282072
H	-5.3715809650	6.6257670148	0.0724691981
Cl	-5.7356121356	2.2724208837	2.0360054978
H	-4.7212621304	-2.2863776130	0.6394928746
C	-3.9194190410	0.8604344186	-2.1247607584
C	-4.8176603905	1.0770993414	-3.3521841220
C	-2.4802150492	1.3135784506	-2.4146910667
H	-3.8528199177	-0.2287507515	-2.0158035116
C	-4.2405698793	0.3787127378	-4.5826016462
H	-4.9362899897	2.1391799304	-3.5772148159
H	-5.8166782923	0.6877520202	-3.1591946274
C	-1.9248897608	0.6105764758	-3.6523853979
H	-2.4253655662	2.3925841996	-2.5742418017
H	-1.8380925696	1.0929236926	-1.5627018784
C	-2.8133331426	0.8280139535	-4.8711713823
H	-4.8831251569	0.5697486316	-5.4457311423
H	-4.2486535322	-0.7043418590	-4.4154426838
H	-0.9107629209	0.9665723282	-3.8503367524
H	-1.8495376235	-0.4638793438	-3.4502014051
H	-2.4127675158	0.2914284692	-5.7349861346
H	-2.8153067498	1.8930377847	-5.1311252751

A5

E(RwB97XD) = -825.960922436

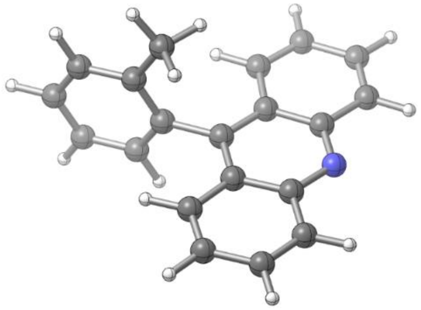

Charge = 0 Multiplicity = 1

C	-7.8591547547	-1.3483511406	-0.9067473052
C	-6.7468440723	-1.7936730528	-0.2691786298
C	-5.6826748882	-0.8985181496	0.0505899325
C	-5.8123310996	0.4790935530	-0.3076028728
C	-6.9984065677	0.9043857621	-0.9778500930
C	-7.9868405680	0.0203209198	-1.2685323485
C	-4.7645360597	1.3500082456	0.0083870120
C	-3.6354147819	0.8354820662	0.6538693686
C	-3.6077645537	-0.5590946315	0.9663151801
C	-2.4585376706	-1.0925210286	1.6227157434
H	-2.4569094569	-2.1505837832	1.8535540300
C	-1.4082588063	-0.2935186627	1.9398187587
C	-1.4326303938	1.0925106700	1.6255742067
C	-2.5096336437	1.6391734468	1.0056392572
H	-8.6603989608	-2.0368020949	-1.1458159252
H	-6.6320405976	-2.8329887826	0.0126635015
H	-7.0987566562	1.9464847297	-1.2528221291
H	-8.8815755545	0.3539645802	-1.7788833841
H	-0.5401778048	-0.7093997842	2.4364145301
H	-0.5834501521	1.7116158684	1.8862645274
H	-2.5266122423	2.6954291864	0.7695860653
N	-4.6083150100	-1.3900424327	0.6699240370
C	-4.8495702958	2.7960517692	-0.3365968962
C	-4.3808435624	3.2619123299	-1.5703687452
C	-5.4015246564	3.6816504724	0.5821369376
C	-4.4838741714	4.6225852168	-1.8436989604
C	-5.4961977887	5.0343105838	0.2930389956
C	-5.0346044758	5.5048999956	-0.9260219147
H	-4.1241850918	4.9950643885	-2.7960880323
H	-5.9279862492	5.7141410543	1.0168762807
C	-3.7813432258	2.3194131037	-2.5745907336
H	-2.9047437165	1.8120160328	-2.1645884057
H	-4.4951831063	1.5444082600	-2.8638628930

H	-3.4755123694	2.8527833438	-3.4743244402
H	-5.1022689129	6.5592096543	-1.1650568584
H	-5.7595629018	3.3029761697	1.5323003732

HA5

E(UwB97XD) = -826.557958096

Charge = 0 Multiplicity = 2

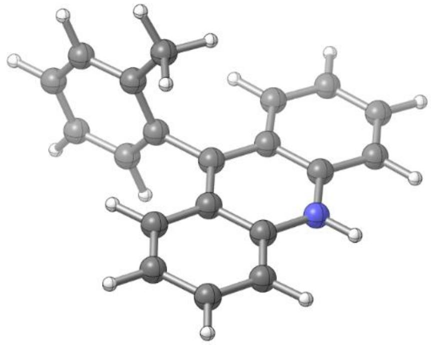

C	-7.9209290700	-1.3379366170	-0.9198110390
C	-6.7824994480	-1.7901483220	-0.2738623540
C	-5.7516400950	-0.9063769700	0.0298959470
C	-5.8472396620	0.4664542760	-0.3099356980
C	-7.0188553750	0.8887085200	-0.9691309160
C	-8.0363914870	0.0057880680	-1.2689105080
C	-4.7725388370	1.3432730540	0.0147587130
C	-3.6113187090	0.8292225910	0.6601006880
C	-3.5486901860	-0.5491627700	0.9844797210
C	-2.4282443000	-1.0842766040	1.6125770140
H	-2.4098548860	-2.1422199770	1.8487841730
C	-1.3517120810	-0.2723657010	1.9282885130
C	-1.3882061720	1.0851470670	1.6174076530
C	-2.4970612000	1.6233955290	0.9965705200
H	-8.7175743160	-2.0334077640	-1.1521268740
H	-6.6810601460	-2.8341649490	-0.0001057090
H	-7.1142538510	1.9320792400	-1.2415677200
H	-8.9248886230	0.3604697090	-1.7761516600
H	-0.4840979570	-0.6982831610	2.4162748540
H	-0.5469757540	1.7210876710	1.8632324640
H	-2.5212804160	2.6794288720	0.7592827850
N	-4.6179960240	-1.3514464380	0.6662468580
C	-4.8574225230	2.7859853700	-0.3303022790
C	-4.3656936960	3.2597297850	-1.5542895750
C	-5.4321323130	3.6771512280	0.5712337830
C	-4.4670249530	4.6194360960	-1.8335003520
C	-5.5263682810	5.0298734530	0.2796676700
C	-5.0405623920	5.5017680480	-0.9294555250
H	-4.0889057090	4.9920184490	-2.7789740710

H	-5.9762951540	5.7089763720	0.9934386350
C	-3.7407249340	2.3183841680	-2.5442029920
H	-2.8593862140	1.8305062580	-2.1204739990
H	-4.4383614970	1.5278761650	-2.8309973100
H	-3.4351608130	2.8474166320	-3.4470358440
H	-5.1071529550	6.5556484300	-1.1713380480
H	-5.8097720530	3.2993351600	1.5144685610
H	-4.5616058390	-2.3295787910	0.9022254600

H_2A5

E(RwB97XD) = -827.180801213

Charge = 0 Multiplicity = 1

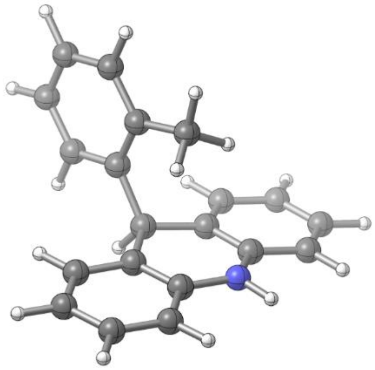

C	-7.8255658639	-1.2326565652	-1.1189647433
C	-6.5204045510	-1.6149294304	-0.8750416950
C	-5.6205704207	-0.7223041411	-0.2873510211
C	-6.0370107346	0.5670020810	0.0511628117
C	-7.3563898322	0.9250035294	-0.1977248915
C	-8.2547519668	0.0442253940	-0.7773924186
C	-5.0842741762	1.5681691336	0.6817141644
C	-3.7381885900	0.9546395163	1.0275166055
C	-3.4133216778	-0.3502444639	0.6507819197
C	-2.1626624287	-0.8804490993	0.9775587793
H	-1.9225694428	-1.8928629633	0.6725706112
C	-1.2438383867	-0.1231293763	1.6785053930
C	-1.5588435225	1.1733829298	2.0672165941
C	-2.7995732210	1.6935777847	1.7374431817
H	-8.5113451964	-1.9359138706	-1.5755455832
H	-6.1807728157	-2.6102505244	-1.1387150111
H	-7.6801134569	1.9263225393	0.0650309711
H	-9.2766813042	0.3493863976	-0.9624320296
H	-0.2781957573	-0.5481038185	1.9241014217
H	-0.8452148220	1.7713647969	2.6193505225
H	-3.0513991397	2.7073301432	2.0295414383
N	-4.3156143691	-1.1145206309	-0.0627927095
C	-4.9541307869	2.8521631326	-0.1334746479
C	-4.4139489207	2.8900920636	-1.4294699181

C	-5.3961921873	4.0367537367	0.4485047359
C	-4.3420821823	4.1174152828	-2.0828284273
C	-5.3183580335	5.2520135455	-0.2157466185
C	-4.7864564884	5.2915958104	-1.4926835879
H	-3.9254179591	4.1500560600	-3.0833985353
H	-5.6711194041	6.1567871758	0.2640017783
C	-3.9139681702	1.6605389431	-2.1346132301
H	-3.1103484855	1.1768063656	-1.5766210838
H	-4.7079323456	0.9229349818	-2.2645669728
H	-3.5299441839	1.9175539266	-3.1217434140
H	-4.7153349279	6.2289572834	-2.0309284266
H	-5.8120075880	4.0037730949	1.4495488082
H	-4.0743079859	-2.0730213403	-0.2506393396
H	-5.5382505046	1.8751054463	1.6281096082

HCyA5

E(RwB97XD) = -1061.87342793

Charge = 0 Multiplicity = 1

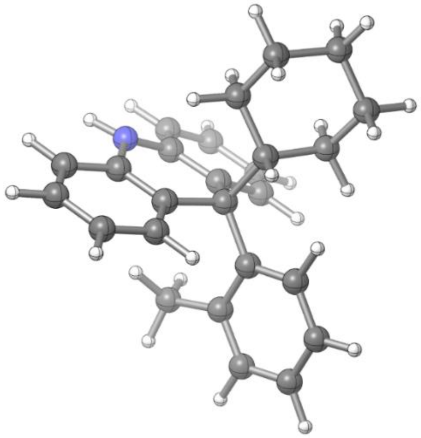

C	-7.9890854483	3.1042041773	-2.3439513041
C	-7.9709772815	1.7527463414	-2.0601792170
C	-7.0614492648	1.2364269280	-1.1345218350
C	-6.1702655798	2.0829833901	-0.4720126379
C	-6.1962339362	3.4355504627	-0.7937804258
C	-7.0903208645	3.9559017328	-1.7143936819
C	-5.2194182126	1.5430750019	0.5957494467
C	-5.0517665497	0.0247313938	0.4559671164
C	-5.9808241909	-0.7408780582	-0.2529928703
C	-5.8576879773	-2.1305158579	-0.3029091232
H	-6.5885688292	-2.7052975868	-0.8605028622
C	-4.8185903423	-2.7642552755	0.3513990289
C	-3.8857201984	-2.0170196882	1.0578588565
C	-4.0124277341	-0.6374115888	1.0979653910
H	-8.6976934420	3.4907581347	-3.0663461954
H	-8.6528996819	1.0755434307	-2.5617270226
H	-5.4892542614	4.0991955003	-0.3120849255
H	-7.0856064425	5.0150704181	-1.9378031989

H	-4.7357061035	-3.8433168512	0.3052744770
H	-3.0667575947	-2.5027024051	1.5728489591
H	-3.2826996065	-0.0561821891	1.6485689281
N	-7.0380683675	-0.1251174170	-0.8952690312
C	-3.8188152010	2.1802344925	0.4348161380
C	-3.1425739807	2.1507855590	-0.8039170261
C	-3.1498352477	2.7328921270	1.5252562359
C	-1.8675897725	2.7059018765	-0.8889079318
C	-1.8784943203	3.2780410036	1.4228621237
C	-1.2317303851	3.2750938463	0.2019791694
H	-1.3565302084	2.6784024217	-1.8447928647
H	-1.4028173409	3.6978065517	2.3008429922
C	-3.7018551846	1.5382153564	-2.0622195191
H	-4.1810899516	0.5773974087	-1.8787014729
H	-4.4449443440	2.1868235888	-2.5295253190
H	-2.8990263207	1.3783621729	-2.7823987871
H	-0.2394765987	3.6966743995	0.0969571082
H	-3.6154374112	2.7394577423	2.4988855738
H	-7.6228862564	-0.7030940859	-1.4761609545
C	-5.8538101172	1.8047245840	2.0145737563
C	-6.1888785772	3.2706748796	2.3204459377
C	-7.1124966389	0.9694631522	2.2850988802
H	-5.1073080919	1.4646799110	2.7405206149
C	-6.6141944008	3.4460980862	3.7759970384
H	-7.0155062039	3.5861134546	1.6780876897
H	-5.3529281317	3.9322177448	2.0944669722
C	-7.5842014740	1.1304536817	3.7292769699
H	-7.9107705229	1.2829482772	1.6037059626
H	-6.9235215958	-0.0868991019	2.0941954989
C	-7.8338947540	2.5889800628	4.0950191978
H	-6.8291962571	4.4992691853	3.9735300918
H	-5.7850824807	3.1628986691	4.4347530949
H	-8.4903959226	0.5401158796	3.8869527414
H	-6.8199042415	0.7179390009	4.3978089848
H	-8.0984106261	2.6757396122	5.1519808158
H	-8.6894734144	2.9620587076	3.5208551120

A4

E(RwB97XD) = -555.579887252

Charge = 0 Multiplicity = 1

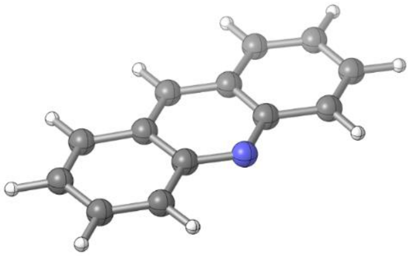

C	-7.9182292462	-1.3381821905	-0.8281515839
C	-6.7969918765	-1.8010604037	-0.2174438494
C	-5.7117354845	-0.9205439452	0.0692354778
C	-5.8346513941	0.4548910351	-0.3012737997
C	-7.0281530866	0.9005464270	-0.9411049617
C	-8.0385363187	0.0308604201	-1.1965370943
C	-4.7714897312	1.3013677156	-0.0163491279
C	-3.6359006745	0.8035604398	0.6087269606
C	-3.6101198852	-0.5874274238	0.9386431836
C	-2.4491561903	-1.1119758410	1.5807960248
H	-2.4386848827	-2.1665953153	1.8268113405
C	-1.3959703315	-0.3044846658	1.8693156740
C	-1.4235084839	1.0794881093	1.5402725637
C	-2.5098123100	1.6170367355	0.9291956495
H	-8.7364630747	-2.0151764465	-1.0412599595
H	-6.6950228372	-2.8410694012	0.0669146753
H	-0.5192009155	-0.7129937022	2.3568045947
H	-2.5424464548	2.6698751062	0.6739800105
N	-4.6255587694	-1.4122043173	0.6706137096
H	-8.9438005419	0.3719032537	-1.6828312692
H	-0.5695723333	1.6995103883	1.7825218317
H	-4.8281286002	2.3515765751	-0.2824231446
H	-7.1083128135	1.9455274421	-1.2172388348

HA4

E(UwB97XD) = -556.176479437

Charge = 0 Multiplicity = 2

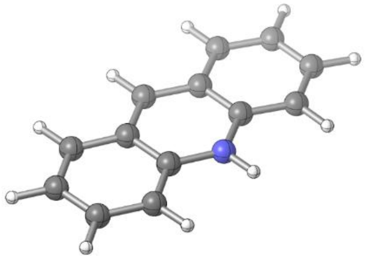

C	-7.9691149771	-1.3325817634	-0.8533047875
C	-6.8233539445	-1.7998916375	-0.2298177849
C	-5.7733761670	-0.9293685684	0.0442221773
C	-5.8599078710	0.4411094197	-0.3088761260
C	-7.0387574011	0.8806567928	-0.9414143024

C	-8.0758644672	0.0114759601	-1.2096148406
C	-4.7715116305	1.2943706691	-0.0135796482
C	-3.6100378944	0.7978641177	0.6223227529
C	-3.5491011107	-0.5767686299	0.9645106675
C	-2.4232184999	-1.1025265621	1.5902145322
H	-2.3993317536	-2.1567696277	1.8418480201
C	-1.3459488532	-0.2828250648	1.8860398032
C	-1.3842673037	1.0722588869	1.5588359466
C	-2.4972447098	1.6008312340	0.9384943182
H	-8.7811779086	-2.0174898120	-1.0624986862
H	-6.7342446145	-2.8436477722	0.0491292830
H	-0.4739029129	-0.7009511479	2.3728761194
H	-2.5327183130	2.6533686648	0.6819624913
N	-4.6281667184	-1.3765644606	0.6632055300
H	-8.9726367894	0.3731798579	-1.6970002546
H	-0.5406437884	1.7098687275	1.7919199850
H	-4.8279634141	2.3429043846	-0.2790815266
H	-7.1161506997	1.9262989749	-1.2162615197
H	-4.5762972253	-2.3519612335	0.9115456716

H_2A4

E(RwB97XD) = -556.803677528

Charge = 0 Multiplicity = 1

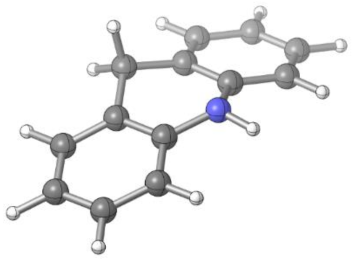

C	-7.8049730120	-1.4003101288	-1.0508915674
C	-6.8045497131	-1.7797090432	-0.1719508045
C	-5.8677859252	-0.8450413917	0.2655701258
C	-5.9395131985	0.4796472628	-0.1749098845
C	-6.9362975519	0.8326570936	-1.0730610710
C	-7.8721441847	-0.0928763771	-1.5133688136
C	-4.9678588025	1.4831051014	0.3885925970
C	-3.6591696261	0.8411062790	0.7677124108
C	-3.6612456550	-0.4948913963	1.1787757225
C	-2.4821101592	-1.0936994098	1.6182882550
H	-2.4964668032	-2.1305066903	1.9351366129
C	-1.3023054843	-0.3691180312	1.6406947984
C	-1.2831839214	0.9513097114	1.2119243433

C	-2.4615526274	1.5415087468	0.7766228197
H	-8.5284178083	-2.1344212744	-1.3842002000
H	-6.7382323531	-2.8042875687	0.1765825389
H	-0.3916386078	-0.8440400894	1.9850087148
H	-2.4584591559	2.5746556281	0.4465342809
N	-4.8517061761	-1.2151460575	1.1420551859
H	-8.6474290513	0.2042518364	-2.2080926466
H	-0.3603749379	1.5173461271	1.2187264888
H	-4.8046213771	2.2972573600	-0.3191096752
H	-6.9845483931	1.8581107935	-1.4230549093
H	-4.7710465575	-2.2027289418	1.3263277169
H	-5.4102103874	1.9360392997	1.2854008603

HCyA4

E(RwB97XD) = -791.507294218

Charge = 0 Multiplicity = 1

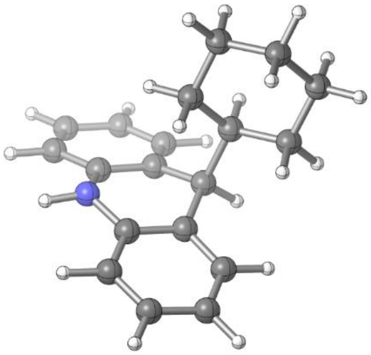

C	-1.5953500123	3.2767110619	-1.3984227381
C	-2.8064217375	3.8981595973	-1.6454224522
C	-3.9902620378	3.3448065897	-1.1571470925
C	-3.9574250759	2.1686046292	-0.4032122729
C	-2.7316460025	1.5526421967	-0.1857534747
C	-1.5511575550	2.0922853770	-0.6735416944
C	-5.2281486846	1.6343103599	0.2161619079
C	-6.4277737017	2.0416171434	-0.6086258182
C	-6.3890382114	3.2255283368	-1.3527483215
C	-7.5212332004	3.6603535831	-2.0419013181
H	-7.4773480626	4.5844838529	-2.6073360344
C	-8.6819029468	2.9080393048	-2.0172066489
C	-8.7201857225	1.7055084422	-1.3234685587
C	-7.5934626604	1.2854853077	-0.6326401822
H	-0.6827326211	3.7147439998	-1.7840501849
H	-2.8467392539	4.8134697363	-2.2251313651
H	-9.5543665472	3.2546762017	-2.5577199850
H	-7.6166678752	0.3492889607	-0.0868832533
N	-5.2101282114	3.9558998720	-1.4127138395
H	-0.6064925479	1.5972404442	-0.4875393542

H	-9.6191398703	1.1022934611	-1.3185756561
H	-2.7072464836	0.6340814657	0.3910615471
H	-5.1956027834	4.7265804084	-2.0613163489
H	-5.1734758717	0.5419378907	0.2195256374
C	-5.3253691322	2.0572515989	1.7099364785
C	-5.4615966533	3.5654096373	1.9215241908
C	-6.4343658763	1.3176988927	2.4581879037
H	-4.3713049265	1.7486248163	2.1561536205
C	-5.4718652150	3.9246117834	3.4056239787
H	-6.3947462270	3.9095758089	1.4615529376
H	-4.6461133748	4.0920050831	1.4209899362
C	-6.4362514241	1.6651137435	3.9447285867
H	-7.4051719738	1.5895801818	2.0314240412
H	-6.3182976308	0.2383825276	2.3217061057
C	-6.5625486606	3.1703217877	4.1594919800
H	-5.6045792597	5.0027802588	3.5259162449
H	-4.4970386683	3.6760756444	3.8405657519
H	-7.2513672035	1.1403258891	4.4494601580
H	-5.5031905279	1.3132767466	4.3994224751
H	-6.5197290543	3.4066492423	5.2257715420
H	-7.5430539752	3.5022066141	3.7993256592

A6

E(RwB97XD) = -786.639268742

Charge = 0 Multiplicity = 1

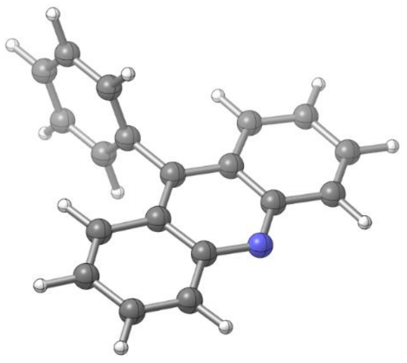

C	-7.8917746311	-1.3526724928	-0.8772484570
C	-6.7663768619	-1.8012706471	-0.2655468185
C	-5.6990080609	-0.9063059694	0.0436702191
C	-5.8367235165	0.4740503949	-0.3021549470
C	-7.0367338694	0.9025088319	-0.9457868606
C	-8.0294389865	0.0190171053	-1.2232107221
C	-4.7850385292	1.3433895423	0.0060720494
C	-3.6472564096	0.8279469059	0.6358492568
C	-3.6131413447	-0.5695114095	0.9361390804
C	-2.4570077164	-1.1057725217	1.5777945624
H	-2.4498066501	-2.1663320247	1.7966946301

C	-1.4083981523	-0.3059225814	1.8981039352
C	-1.4412841229	1.0836449130	1.6012053230
C	-2.5229745911	1.6326053839	0.9916006013
H	-8.6959664858	-2.0407557749	-1.1072966661
H	-6.6438099607	-2.8427865308	0.0046245161
H	-7.1451352380	1.9460924569	-1.2113735120
H	-8.9349934823	0.3556297058	-1.7120607174
H	-0.5356069343	-0.7236683312	2.3847622720
H	-0.5942661030	1.7039080982	1.8661313511
H	-2.5442149344	2.6913297106	0.7679786409
N	-4.6151075369	-1.4001411691	0.6440479026
C	-4.8753806290	2.7900206230	-0.3304125198
C	-4.4025050339	3.2577907444	-1.5523925578
C	-5.4340823055	3.6877387761	0.5737682033
C	-4.4878466492	4.6064010725	-1.8656581857
C	-5.5183078120	5.0361883041	0.2594655898
H	-5.8036705269	3.3269798076	1.5262305479
C	-5.0455981315	5.4981738985	-0.9605953452
H	-4.1172732110	4.9604383114	-2.8197550935
H	-5.9545648822	5.7269773722	0.9704293290
H	-5.1117576815	6.5510166438	-1.2055583367
H	-3.9669189025	2.5614747614	-2.2590619484

HA6

E(UwB97XD) = -787.236349923

Charge = 0 Multiplicity = 2

C	-7.9910488190	-1.3265346580	-0.7912198560
C	-6.8271810980	-1.7835604570	-0.1975656380
C	-5.7788993120	-0.9049356450	0.0591776100
C	-5.8815370040	0.4696731130	-0.2719180740
C	-7.0774611740	0.8941324990	-0.8857516790
C	-8.1122150290	0.0169493940	-1.1387197870
C	-4.7850445810	1.3396438590	0.0070260810
C	-3.6019599870	0.8150383520	0.6087395090
C	-3.5350998680	-0.5665456290	0.9193492450
C	-2.3979899110	-1.1146712560	1.5054811400

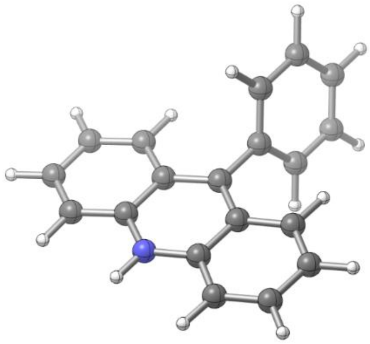

H	-2.3780231680	-2.1759564330	1.7261860580
C	-1.3107513000	-0.3115674040	1.8035553370
C	-1.3561196240	1.0517127860	1.5210658160
C	-2.4794620480	1.6016199220	0.9381280060
H	-8.8010251550	-2.0178279970	-0.9869535170
H	-6.7177217990	-2.8281449970	0.0707548510
H	-7.1797729840	1.9342802640	-1.1656378460
H	-9.0178503490	0.3762740010	-1.6111494310
H	-0.4306324250	-0.7470660220	2.2595970040
H	-0.5107165910	1.6844021250	1.7608620920
H	-2.5062604410	2.6628039250	0.7285641300
N	-4.6191150260	-1.3615120270	0.6364932720
C	-4.8747342340	2.7818977710	-0.3275166300
C	-4.1472468400	3.3131595970	-1.3915315880
C	-5.6888224130	3.6359172210	0.4151819890
C	-4.2310715880	4.6617214030	-1.7053401320
C	-5.7736187070	4.9848346790	0.1032672120
H	-6.2597993790	3.2362032140	1.2450750140
C	-5.0447196780	5.5019611350	-0.9584093180
H	-3.6603331990	5.0563689850	-2.5372343820
H	-6.4096000460	5.6337600810	0.6931525030
H	-5.1105448320	6.5551196110	-1.2026348020
H	-3.5107653920	2.6605301520	-1.9776084930
H	-4.5587877350	-2.3412360570	0.8648509340

H_2A6

E(RwB97XD) = -787.862049949

Charge = 0 Multiplicity = 1

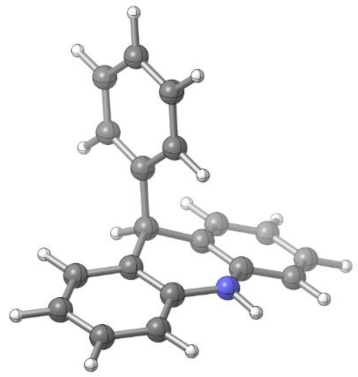

C	-7.8625699613	-1.2088645119	-1.0143568448
C	-6.5048212999	-1.4686400331	-1.0022669193
C	-5.6245336990	-0.5781218691	-0.3853777828
C	-6.1138668897	0.5856660830	0.2115638940
C	-7.4833206024	0.8181434407	0.2014444207
C	-8.3627518018	-0.0644828154	-0.4042894098
C	-5.1566952387	1.5992718950	0.8056117381
C	-3.8363975938	0.9562531112	1.1769110456

C	-3.4250961193	-0.2209745196	0.5481992898
C	-2.1718774425	-0.7644917011	0.8361497744
H	-1.8629201496	-1.6771209145	0.3390231681
C	-1.3398812863	-0.1478607224	1.7518643282
C	-1.7480743419	1.0120278092	2.4000020960
C	-2.9905803498	1.5495115523	2.1056608193
H	-8.5350827230	-1.9086190054	-1.4953622882
H	-6.1122701754	-2.3677970059	-1.4636358279
H	-7.8608842190	1.7208483402	0.6697099823
H	-9.4260709814	0.1382438796	-0.4048398317
H	-0.3702573622	-0.5802378455	1.9668368512
H	-1.1024495192	1.4931605453	3.1234921101
H	-3.3144895579	2.4612971114	2.5959886890
N	-4.2638573539	-0.8447847671	-0.3630357981
C	-4.9559678351	2.7781559299	-0.1385672996
C	-4.3417995451	2.6095174549	-1.3783216813
C	-5.3946671387	4.0464430888	0.2216952542
C	-4.1732059624	3.6847278736	-2.2349523650
C	-5.2271015196	5.1272950511	-0.6354009580
H	-5.8737306376	4.1912882910	1.1836853039
C	-4.6158811817	4.9495435059	-1.8662834539
H	-3.6940568330	3.5366690691	-3.1952602256
H	-5.5752112254	6.1089951040	-0.3372993375
H	-4.4834802288	5.7897946626	-2.5367508169
H	-3.9929086282	1.6277000168	-1.6780799437
H	-5.6077735652	1.9996124315	1.7159270072
H	-3.9767510315	-1.7519515360	-0.6932979880

HCyA6

E(RwB97XD) = -1022.55668700

Charge = 0 Multiplicity = 1

C	-8.0786222053	2.6833076863	-2.3717874936
C	-7.9347956084	1.3593482289	-2.0096463656
C	-7.0070059889	0.9897661562	-1.0316191686
C	-6.2221846631	1.9560036983	-0.3981565136
C	-6.3725142188	3.2800936907	-0.8027469871

C	-7.2871987958	3.6550767112	-1.7714673733
C	-5.2694081895	1.5781587956	0.7379967900
C	-4.9423217111	0.0790063704	0.6791379147
C	-5.7656242975	-0.8121971328	-0.0131682892
C	-5.4973975449	-2.1830409498	0.0061323425
H	-6.1479773352	-2.8546267054	-0.5430647029
C	-4.4214057917	-2.6750947547	0.7180305595
C	-3.5939728909	-1.8026364753	1.4135049116
C	-3.8614422125	-0.4440945576	1.3815995551
H	-8.8002029432	2.9560373991	-3.1322203863
H	-8.5317894217	0.5897694505	-2.4860871737
H	-5.7532845942	4.0393210627	-0.3422656901
H	-7.3817040904	4.6953805583	-2.0553546484
H	-4.2258494631	-3.7404661636	0.7257997054
H	-2.7466285860	-2.1761344956	1.9741575516
H	-3.2119373647	0.2327407133	1.9231191712
N	-6.8693561500	-0.3448678554	-0.7005543924
C	-3.9369651582	2.3330021576	0.5686303280
C	-3.3370304583	2.3523666463	-0.6917242772
C	-3.2459831711	2.9263745744	1.6194715244
C	-2.1078581422	2.9531027891	-0.8993244807
C	-2.0112857144	3.5331213825	1.4174346191
H	-3.6537482889	2.9200618583	2.6205219938
C	-1.4369751982	3.5536634030	0.1583401641
H	-1.6707913599	2.9505216418	-1.8906048847
H	-1.5000133172	3.9896951938	2.2564328531
H	-0.4758897182	4.0275003837	0.0005775569
H	-3.8418524852	1.8833682054	-1.5276320216
H	-7.3689325901	-1.0096599008	-1.2680768573
C	-5.9516177830	1.8623296604	2.1226574494
C	-6.3715566384	3.3192894172	2.3483152744
C	-7.1614065311	0.9623959672	2.4002979371
H	-5.2002485122	1.6067047286	2.8784892402
C	-6.8607218551	3.5356833185	3.7781212019
H	-7.1861788998	3.5661249414	1.6616921709
H	-5.5561881027	4.0078655508	2.1245896481

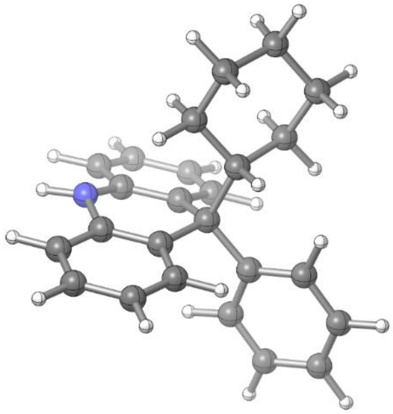

Go back to table of contents

C	-7.6905140864	1.1646338303	3.8187591492
H	-7.9547173223	1.1898833193	1.6798589546
H	-6.8978155828	-0.0872861290	2.2659320744
C	-8.0391381822	2.6223720496	4.0977816397
H	-7.1437410789	4.5819991289	3.9177818074
H	-6.0414227837	3.3328929845	4.4775198496
H	-8.5646418185	0.5286274690	3.9795805002
H	-6.9259972798	0.8349336732	4.5315229069
H	-8.3484774208	2.7463205654	5.1387282553
H	-8.8928187786	2.9130780272	3.4751754859

A7

E(RwB97XD) = -885.893707126

Charge = 0 Multiplicity = 1

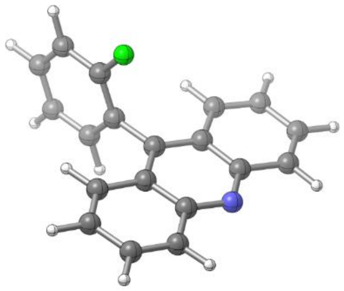

C	-7.8899184365	-1.3553691840	-0.8800413932
C	-6.7689147204	-1.8027598618	-0.2597407362
C	-5.7007171774	-0.9083317113	0.0478303084
C	-5.8347545924	0.4702531582	-0.3062988320
C	-7.0296706448	0.8977738104	-0.9590253945
C	-8.0221496403	0.0143393141	-1.2364809485
C	-4.7824455677	1.3365181435	0.0033510834
C	-3.6449827356	0.8244880536	0.6340255109
C	-3.6151672419	-0.5712720868	0.9424296370
C	-2.4608103978	-1.1069371448	1.5875938031
H	-2.4571522381	-2.1658813694	1.8140175373
C	-1.4090371221	-0.3084372090	1.9002562898
C	-1.4367920125	1.0788597969	1.5916952777
C	-2.5174465730	1.6277927565	0.9806593400
H	-8.6946943769	-2.0427794924	-1.1098148619
H	-6.6499475970	-2.8427455806	0.0176318496
H	-7.1340215102	1.9398364054	-1.2327738304
H	-8.9237045515	0.3492661012	-1.7337523004
H	-0.5369830105	-0.7253881058	2.3888012911
H	-0.5860712189	1.6973304724	1.8487116024
H	-2.5343519055	2.6848299191	0.7479060575
N	-4.6195812944	-1.3999274170	0.6543647783

C	-4.8782321749	2.7845153652	-0.3218100193
C	-4.4232950037	3.2811715855	-1.5323300990
C	-5.4289445651	3.6935366050	0.5770370106
C	-4.4958242591	4.6171585621	-1.8700763368
C	-5.5149362178	5.0413245186	0.2657546999
H	-5.7920803351	3.3291430065	1.5302179895
C	-5.0483966385	5.5020037496	-0.9573786184
H	-4.1240272945	4.9471072167	-2.8313129802
H	-5.9463921943	5.7316893371	0.9788593476
H	-5.1131542698	6.5536693863	-1.2057153211
F	-3.8870006315	2.4231626192	-2.4193179124

HA7

E(UwB97XD) = -886.491816817

Charge = 0 Multiplicity = 2

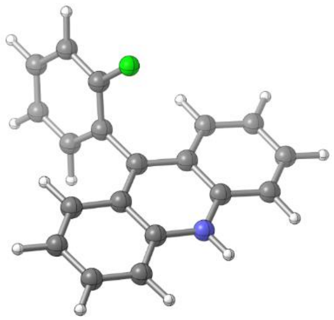

C	2.1380810262	3.5981134092	0.1087500541
C	2.7955068269	2.3807295547	0.0887930828
C	2.0711785103	1.1947091305	0.0079797126
C	0.6557927936	1.2086349904	-0.0490410575
C	0.0229861696	2.4672148703	-0.0348174525
C	0.7467374848	3.6388232849	0.0440685748
C	-0.0500172440	-0.0282516345	-0.1296154034
C	0.6715171012	-1.2582967978	-0.1500794132
C	2.0876341053	-1.2296085739	-0.0977159388
C	2.8283528947	-2.4080799355	-0.1168404026
H	3.9103601702	-2.3516383193	-0.0792505339
C	2.1874359453	-3.6328073681	-0.1794494116
C	0.7957072981	-3.6884005872	-0.2197597000
C	0.0551783796	-2.5246613666	-0.2035034982
H	2.7094342642	4.5156012617	0.1716988376
H	3.8777396080	2.3358776069	0.1340205831
H	-1.0573957391	2.5098327262	-0.0895075881
H	0.2301074241	4.5902924721	0.0544336583
H	2.7717112172	-4.5442108863	-0.1930029141
H	0.2914777847	-4.6456883395	-0.2612034446
H	-1.0253405805	-2.5786785563	-0.2293969845

N	2.7224197287	-0.0141126673	-0.0226400771
C	-1.5295004301	-0.0363294599	-0.2028237071
C	-2.3174285684	0.2457659090	0.9048726994
C	-2.2023982743	-0.3295072720	-1.3890173914
C	-3.6973652879	0.2503961794	0.8731737392
C	-3.5867153970	-0.3348501349	-1.4538680472
H	-1.6172619083	-0.5557880028	-2.2722975211
C	-4.3349943347	-0.0435451411	-0.3218285213
H	-4.2519185124	0.4788511740	1.7741804702
H	-4.0814017255	-0.5638707502	-2.3891069665
H	-5.4167652643	-0.0446550844	-0.3649441343
F	-1.7107073006	0.5270309947	2.0741475386
H	3.7289148347	-0.0092904244	0.0238611583

H_2A7

E(RwB97XD) = -887.116972775

Charge = 0 Multiplicity = 1

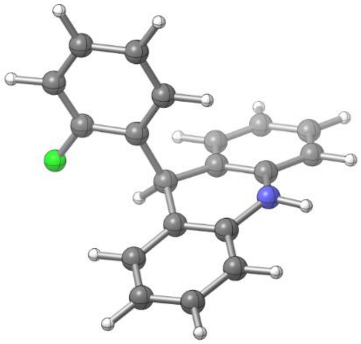

C	-7.9616944904	-1.1065612644	-0.8692914362
C	-7.1242910060	-1.1492594365	0.2299076256
C	-5.9384620658	-0.4131707596	0.2385649340
C	-5.6021166761	0.3787866323	-0.8613506434
C	-6.4492613537	0.3935940937	-1.9620447575
C	-7.6249126302	-0.3392661236	-1.9781411649
C	-4.3622798295	1.2512508544	-0.8167208622
C	-3.3474407254	0.7153675422	0.1695426094
C	-3.7579367772	-0.0969866225	1.2282676827
C	-2.8290242212	-0.5276955526	2.1773456840
H	-3.1598607841	-1.1564915961	2.9962067512
C	-1.5008053131	-0.1616369252	2.0655848250
C	-1.0774582422	0.6288255040	1.0031995599
C	-2.0047414945	1.0569262398	0.0675278586
H	-8.8784704988	-1.6834432207	-0.8635648508
H	-7.3758563907	-1.7597159528	1.0900099275
H	-6.1822570263	1.0058057862	-2.8163673516
H	-8.2738952052	-0.3105891112	-2.8439875016
H	-0.7896814785	-0.5035014820	2.8076966413

H	-0.0365350871	0.9102544552	0.9086839745
H	-1.6893395089	1.6835409147	-0.7598191873
N	-5.0897061346	-0.4680648717	1.3341645139
C	-4.7454854831	2.6917894218	-0.5015316019
C	-4.8000287111	3.6572557964	-1.4940070528
C	-5.0878639123	3.1015503478	0.7853207957
C	-5.1661233673	4.9684676016	-1.2597308146
C	-5.4640059114	4.4070599465	1.0540627212
H	-5.0576302918	2.3783044216	1.5916399619
C	-5.5027241003	5.3439857448	0.0301130502
H	-5.1829869613	5.6725034773	-2.0817445798
H	-5.7251012220	4.6943711260	2.0645890897
H	-5.7927042172	6.3671353748	0.2327307901
F	-4.4800073727	3.3144896565	-2.7610721369
H	-3.9076838035	1.2447105991	-1.8070070694
H	-5.3131371764	-1.1482780178	2.0426979244

HCyA7

E(RwB97XD) = -1121.81062167

Charge = 0 Multiplicity = 1

C	-7.3251152040	-2.0449618240	-1.3765336060
C	-5.9911571120	-2.2712641030	-1.0995958000
C	-5.2167335460	-1.2799413030	-0.4931556510
C	-5.7770447790	-0.0399990550	-0.1787643010
C	-7.1290425460	0.1501323220	-0.4428531700
C	-7.9060885580	-0.8298944620	-1.0379743190
C	-4.9139731900	1.0835289740	0.3996244180
C	-3.6458728280	0.5086613270	1.0433106860
C	-3.1798595820	-0.7617703850	0.6977064930
C	-1.9801036390	-1.2393882910	1.2281517650
H	-1.6372348660	-2.2295925960	0.9495057990
C	-1.2389555940	-0.4592610760	2.0948121100
C	-1.6902025360	0.8052068130	2.4487744080
C	-2.8852669200	1.2697285230	1.9229955730
H	-7.9147685780	-2.8236742190	-1.8447922370
H	-5.5338625750	-3.2254909460	-1.3363046190

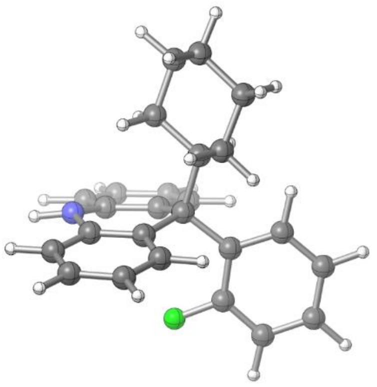

H	-7.5857534890	1.0958841050	-0.1788397090
H	-8.9541540620	-0.6457673120	-1.2364598460
H	-0.3092594890	-0.8422574650	2.4976708320
H	-1.1180115680	1.4244463990	3.1276716460
H	-3.2348147470	2.2571578620	2.1999950960
N	-3.8920189750	-1.5411714260	-0.1937028850
C	-5.6898130560	1.8179129940	1.5044105640
C	-6.2419322040	1.1001579740	2.5638411190
C	-5.8324204650	3.2009534490	1.5837216340
C	-6.9230633850	1.6788862290	3.6133224040
C	-6.5073769030	3.8170055630	2.6288778050
H	-5.3999891540	3.8285770990	0.8186898750
C	-7.0626092110	3.0572904910	3.6439550710
H	-7.3251881030	1.0464687660	4.3942705160
H	-6.5938554750	4.8959969660	2.6441975090
H	-7.5932912030	3.5285318200	4.4613653310
F	-6.0977162120	-0.2391499550	2.5994135600
H	-3.5744446070	-2.4872015090	-0.3270290340
C	-4.4674882950	2.0532583550	-0.7548045520
C	-3.4614439390	1.4207725440	-1.7243440380
C	-5.6155600910	2.6476859960	-1.5806843030
H	-3.9391150360	2.8766911270	-0.2606694550
C	-2.9385077530	2.4464452750	-2.7282872900
H	-3.9444174890	0.5976429240	-2.2618344320
H	-2.6167212630	0.9974661630	-1.1806175460
C	-5.1035889770	3.7062448480	-2.5541983950
H	-6.0892931250	1.8473378530	-2.1556869870
H	-6.3929257810	3.0715473640	-0.9447720470
C	-4.0656137540	3.1186618290	-3.5036847180
H	-2.2407848450	1.9629822220	-3.4165952130
H	-2.3685107020	3.2106911200	-2.1876630290
H	-5.9409051200	4.1234345620	-3.1191002190
H	-4.6570025540	4.5340576540	-1.9913641330
H	-3.6646411690	3.8942684350	-4.1611187110
H	-4.5523400230	2.3765168520	-4.1464350550

A8

E(RwB97XD) = -904.595421282

Charge = 0 Multiplicity = 1

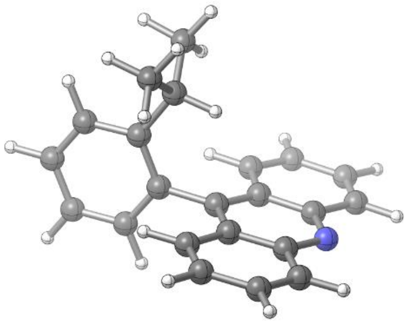

C	-7.9177322581	-1.5137448201	-0.4804910470
C	-6.7378359408	-1.8996688487	0.0683409730
C	-5.6763230364	-0.9631492128	0.2469564278
C	-5.8810660977	0.3924006879	-0.1577457144
C	-7.1367866128	0.7549284542	-0.7310627153
C	-8.1207415162	-0.1673432307	-0.8878715220
C	-4.8341842546	1.3033342249	0.0157373854
C	-3.6352192720	0.8499816254	0.5750740625
C	-3.5354968645	-0.5271914989	0.9444947789
C	-2.3129467642	-0.9985457373	1.5091767644
H	-2.2558178799	-2.0442816244	1.7847693684
C	-1.2624225852	-0.1577854487	1.6880107548
C	-1.3591133491	1.2109101816	1.3174382696
C	-2.5065506618	1.6988964065	0.7802937511
H	-8.7160045121	-2.2337629116	-0.6127841189
H	-6.5658005793	-2.9215967691	0.3827801153
H	-7.2941968255	1.7801398212	-1.0400976333
H	-9.0691440796	0.1187905493	-1.3248911420
H	-0.3385699347	-0.5271467238	2.1160316283
H	-0.5087515516	1.8640190075	1.4677870570
H	-2.5788496570	2.7417057548	0.4998110917
N	-4.5336370046	-1.3972988547	0.7814866249
C	-4.9962536469	2.7305987703	-0.3794441138
C	-4.6573754795	3.1745029252	-1.6603185869
C	-5.4990049033	3.6257191798	0.5631261776
C	-4.8383991355	4.5252536846	-1.9567720169
C	-5.6707162552	4.9625829050	0.2500982587
H	-5.7562609468	3.2620372082	1.5510439781
C	-5.3375019069	5.4132205045	-1.0195776313
H	-4.5818247625	4.8881415883	-2.9454227304
H	-6.0621966449	5.6477929011	0.9916209695
H	-5.4668550961	6.4568585596	-1.2793291866
C	-4.1049223083	2.2349816480	-2.7145851044

H	-4.0516336667	1.2358478653	-2.2797522714
C	-5.0291399357	2.1536436293	-3.9297627870
H	-6.0352637963	1.8440367900	-3.6388955162
H	-5.1045557003	3.1192322358	-4.4359158460
H	-4.6454675692	1.4272797972	-4.6496651217
C	-2.6853142631	2.6306078535	-3.1223110790
H	-2.0203539265	2.6565133321	-2.2563565676
H	-2.2817920654	1.9113246179	-3.8384683044
H	-2.6688017535	3.6172379721	-3.5920846807

HA8

E(UwB97XD) = -905.192376173

Charge = 0 Multiplicity = 2

C	-7.9814860190	-1.5061726280	-0.4874172800
C	-6.7746195350	-1.8969501040	0.0684545590
C	-5.7471143860	-0.9733093300	0.2319060380
C	-5.9155919210	0.3784953870	-0.1598813810
C	-7.1557403480	0.7379155320	-0.7240162920
C	-8.1695736290	-0.1846715000	-0.8857611400
C	-4.8418188320	1.2969757320	0.0201744990
C	-3.6106505420	0.8450373020	0.5762902560
C	-3.4754849730	-0.5143202090	0.9540440870
C	-2.2841688000	-0.9899542140	1.4927666650
H	-2.2110635630	-2.0348634120	1.7722618520
C	-1.2076783970	-0.1360437640	1.6667819860
C	-1.3152762750	1.2039352910	1.3017112210
C	-2.4943738670	1.6830387740	0.7675799740
H	-8.7748267100	-2.2325602590	-0.6111067030
H	-6.6174279560	-2.9231595460	0.3807772080
H	-7.3076811160	1.7638926760	-1.0337915810
H	-9.1117913060	0.1220518230	-1.3224152740
H	-0.2846584950	-0.5158761800	2.0863699420
H	-0.4745173050	1.8728431650	1.4365311970
H	-2.5733009720	2.7252566060	0.4861601640
N	-4.5454571940	-1.3582050170	0.7763411090
C	-5.0042867380	2.7209266100	-0.3744124990

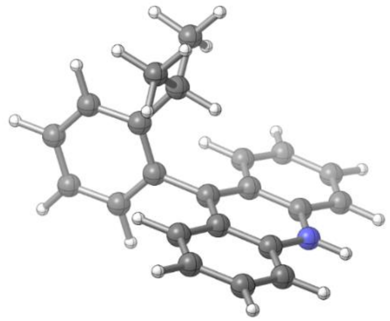

C	-4.6516100480	3.1742711030	-1.6509522400
C	-5.5226301000	3.6209743740	0.5572449820
C	-4.8328624710	4.5241588410	-1.9510693350
C	-5.6956887500	4.9581026230	0.2436480190
H	-5.7922370710	3.2573645890	1.5422021080
C	-5.3475166050	5.4112535610	-1.0210532560
H	-4.5646968500	4.8879152980	-2.9366133130
H	-6.0993296230	5.6417221460	0.9804696380
H	-5.4770534590	6.4546170190	-1.2824539990
C	-4.0848357250	2.2367197280	-2.6989469740
H	-4.0276245040	1.2421386610	-2.2540300270
C	-5.0018156030	2.1417178590	-3.9187139730
H	-6.0067963010	1.8251587190	-3.6313209800
H	-5.0828933210	3.1046466490	-4.4295076470
H	-4.6090885460	1.4155176210	-4.6341882850
C	-2.6666443750	2.6409126190	-3.1030222180
H	-2.0064540690	2.6796158560	-2.2339321200
H	-2.2526964080	1.9194002660	-3.8112234210
H	-2.6554626130	3.6236823600	-3.5813589090
H	-4.4373851300	-2.3227967740	1.0473138110

H_2A8

E(RwB97XD) = -905.813383741

Charge = 0 Multiplicity = 1

C	-8.0503628149	-1.5945725574	-0.1772777891
C	-7.1079834011	-1.4829072906	0.8276536672
C	-5.9574702979	-0.7163458995	0.6311126865
C	-5.7611902838	-0.0497331599	-0.5797594508
C	-6.7141987005	-0.1900241873	-1.5809176982
C	-7.8558189962	-0.9518694222	-1.3944176270
C	-4.5541805354	0.8427269819	-0.7843443240
C	-3.4402067422	0.5010183127	0.1872059411
C	-3.7210445509	-0.1769509708	1.3757087721
C	-2.7017824767	-0.4194075187	2.2986032588
H	-2.9340068445	-0.9450849317	3.2180479088
C	-1.4108122690	0.0000731967	2.0378267177

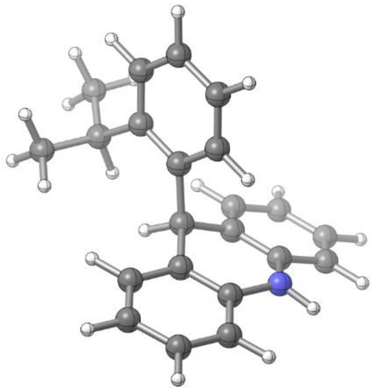

C	-1.1158406004	0.6593623494	0.8504981970
C	-2.1325055151	0.9012849303	-0.0589177508
H	-8.9383651623	-2.1923561226	-0.0115997437
H	-7.2494034551	-1.9925385039	1.7742124429
H	-6.5568152097	0.3254175898	-2.5223703209
H	-8.5878705103	-1.0421388717	-2.1867050455
H	-0.6297948715	-0.1944719992	2.7627214899
H	-0.1053851427	0.9848660449	0.6387874915
H	-1.9138084319	1.4281530763	-0.9812147595
N	-5.0121020838	-0.6075830229	1.6386483428
C	-4.9240388072	2.3231406304	-0.6760571569
C	-4.5682824951	3.2703724108	-1.6458167434
C	-5.6347796934	2.7362489972	0.4519780444
C	-4.9762291058	4.5934039897	-1.4551671666
C	-6.0155599474	4.0523794187	0.6309419980
H	-5.8979985607	2.0047723797	1.2073523687
C	-5.6891766936	4.9896173555	-0.3385618992
H	-4.7288427466	5.3356744802	-2.2041293580
H	-6.5667569086	4.3433654503	1.5167736643
H	-5.9860279641	6.0251935861	-0.2245168682
C	-3.8006173541	2.9173033368	-2.9102129926
H	-3.2941973321	1.9660878306	-2.7523317334
C	-4.7627911395	2.7341708821	-4.0855294781
H	-5.5036816822	1.9605046696	-3.8721579793
H	-5.2992180064	3.6638008346	-4.2934389788
H	-4.2193178167	2.4464947647	-4.9887422839
C	-2.7067558005	3.9291274809	-3.2513364490
H	-2.0440976143	4.0997016391	-2.4002295303
H	-2.1032833756	3.5543923808	-4.0810509561
H	-3.1181882658	4.8928648336	-3.5583574186
H	-4.1891508218	0.6531085153	-1.7913215615
H	-5.1387538522	-1.1963128901	2.4457427617

HCyA8

E(RwB97XD) = -1140.50794089

Charge = 0 Multiplicity = 1

C	-7.8720162919	-1.8564170427	-0.5236715375
C	-6.5652679840	-2.2388090618	-0.2848914942
C	-5.5793129446	-1.2775907136	-0.0569137432
C	-5.9065990417	0.0803988034	-0.0510099885
C	-7.2278669250	0.4369126716	-0.2883299248
C	-8.2109140775	-0.5102697997	-0.5296461019
C	-4.8275609387	1.1523867303	0.1378088341
C	-3.5638919285	0.5488646500	0.7534268225
C	-3.3172039515	-0.8247731359	0.6802336444
C	-2.1223048256	-1.3551017989	1.1700246228
H	-1.9531321133	-2.4240672374	1.1027687424
C	-1.1762608186	-0.5303179403	1.7481301952
C	-1.4203042280	0.8316907903	1.8588581769
C	-2.6082369641	1.3492899287	1.3682715437
H	-8.6268522846	-2.6127766509	-0.7010593014
H	-6.2927775239	-3.2882694812	-0.2743277120
H	-7.4944329069	1.4870043153	-0.2839811725
H	-9.2311348095	-0.1996709661	-0.7153324800
H	-0.2538230334	-0.9551916227	2.1248638036
H	-0.6943165384	1.4859449859	2.3243123632
H	-2.8005254167	2.4104252162	1.4633614980
N	-4.2706365756	-1.6739496344	0.1477643234
C	-5.4111375052	2.2378918623	1.0762662446
C	-5.7401768540	1.9497731226	2.4177079475
C	-5.6899557624	3.5192212474	0.6008520588
C	-6.2721235501	2.9650533401	3.2112788462
C	-6.2281922337	4.5129237254	1.4029275511
H	-5.4946326887	3.7673732663	-0.4307050154
C	-6.5117471260	4.2390115785	2.7277216983
H	-6.5202482610	2.7450399510	4.2430760346
H	-6.4221587526	5.4934141434	0.9853750680
H	-6.9269054008	5.0005257768	3.3767253848
C	-5.6044646405	0.5790162040	3.0668973647
H	-5.1793045651	-0.1125202550	2.3495785072
C	-4.6567938830	0.6021255021	4.2662688653
H	-3.6721392066	0.9767121053	3.9802036853

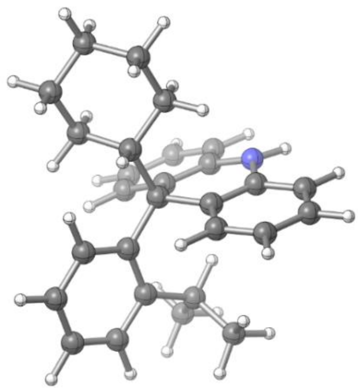

H	-5.0400187057	1.2330576539	5.0717177996
H	-4.5320626831	-0.4072485202	4.6655017178
C	-6.9736051314	0.0154377037	3.4526409852
H	-7.6369335415	-0.0282824641	2.5862134258
H	-6.8647654010	-0.9974409281	3.8478747386
H	-7.4573118992	0.6237693066	4.2206042831
H	-4.0970339538	-2.6623420541	0.2266824420
C	-4.4700400964	1.7039111278	-1.2956166740
C	-3.3033763814	2.7006667553	-1.3405605609
C	-4.1793637094	0.6006684199	-2.3224421508
H	-5.3723372389	2.2137242224	-1.6506646851
C	-3.1623905398	3.3221734501	-2.7275070673
H	-2.3771885114	2.1704405251	-1.1030174671
H	-3.4092741576	3.4838785831	-0.5905867250
C	-4.0121007535	1.1807944871	-3.7259798387
H	-3.2657942279	0.0683107357	-2.0365410852
H	-4.9857598739	-0.1323349080	-2.3410945624
C	-2.9260844381	2.2488646862	-3.7838520087
H	-2.3394497858	4.0413359399	-2.7277575659
H	-4.0728022483	3.8820042681	-2.9710149914
H	-3.7895680698	0.3757909806	-4.4309735975
H	-4.9654453652	1.6201481010	-4.0411565750
H	-2.8835351261	2.6938353247	-4.7812735285
H	-1.9519014941	1.7810844588	-3.6021166535

A9

E(RwB97XD) = -594.898606658

Charge = 0 Multiplicity = 1

C	-3.5721413665	-1.0360243446	0.0009688539
C	-2.3714946954	-1.6691904960	0.0051842929
C	-1.1539636002	-0.9257751104	0.0056930676
C	-1.2124729776	0.5000195122	0.0017747855
C	-2.4967952024	1.1231964823	-0.0025730016
C	-3.6357402154	0.3823556035	-0.0030174971
C	-0.0126421028	1.2303548272	0.0022979128
C	1.1932733647	0.5137985009	0.0062613656

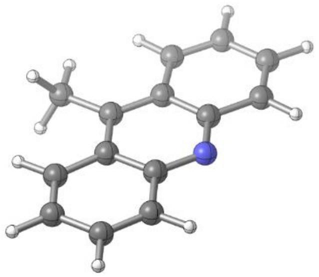

C	1.1360538481	-0.9182819125	0.0101166947
C	2.3545226121	-1.6626810744	0.0144353381
H	2.2791276382	-2.7430016755	0.0174047152
C	3.5556773720	-1.0335672875	0.0146922486
C	3.6202920944	0.3852994785	0.0106086876
C	2.4845270048	1.1287889568	0.0066022041
H	-4.4905574956	-1.6101723519	0.0005895135
H	-2.2997340890	-2.7498183965	0.0082530474
H	-2.5628328729	2.2027577574	-0.0056326521
H	-4.6014409124	0.8721020100	-0.0064301287
H	4.4731685273	-1.6091616598	0.0179239801
H	4.5863463217	0.8742996630	0.0107215669
H	2.5651132599	2.2063423846	0.0034691919
N	-0.0055369515	-1.6055787077	0.0098770758
C	-0.0626987199	2.7290961847	-0.0013456180
H	-0.5958582408	3.0902173950	-0.8833105682
H	-0.6015247824	3.0942395083	0.8754832011
H	0.9213735998	3.1857577525	0.0008567227

HA9

E(UwB97XD) = -595.494334362

Charge = 0 Multiplicity = 2

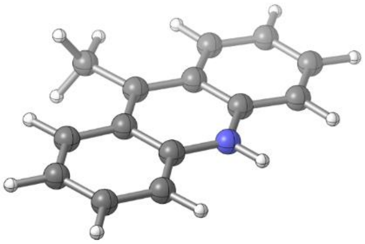

C	-3.6257589669	-1.0188894493	0.0006178836
C	-2.4003464269	-1.6649582859	0.0050923363
C	-1.2202602016	-0.9279111181	0.0056866818
C	-1.2401003696	0.4885736520	0.0017169245
C	-2.5062546141	1.1079111759	-0.0028995717
C	-3.6757696978	0.3717095243	-0.0034550296
C	-0.0115639464	1.2191778454	0.0025785841
C	1.2236213521	0.5026513027	0.0062999684
C	1.2047123303	-0.9181954930	0.0103985751
C	2.3833919115	-1.6576539593	0.0145158253
H	2.3246154395	-2.7403660763	0.0177031541
C	3.6112233320	-1.0184898393	0.0144500117
C	3.6628052300	0.3724788299	0.0101336992
C	2.4971614589	1.1119722086	0.0062150216

H	-4.5393391397	-1.6000787613	0.0002060714
H	-2.3464009461	-2.7478501417	0.0081835581
H	-2.5644646141	2.1879879257	-0.0061273019
H	-4.6313311060	0.8811779457	-0.0070739080
H	4.5229522814	-1.6023722870	0.0176018060
H	4.6189092728	0.8809654046	0.0098893384
H	2.5705189211	2.1903156687	0.0028750263
N	-0.0058207378	-1.5669138376	0.0102981347
C	-0.0684175960	2.7147664302	-0.0007176390
H	-0.5995575362	3.0874786129	-0.8823417170
H	-0.6071255563	3.0908400506	0.8748280125
H	0.9157018470	3.1744802057	0.0027336562
H	-0.0009724211	-2.5742358943	0.0135502138

H_2A9

E(RwB97XD) = -596.122373057

Charge = 0 Multiplicity = 1

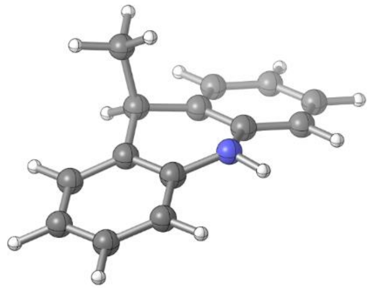

C	-3.3389583578	-1.5256319842	0.1224515272
C	-2.0338596963	-1.9054622097	-0.1380651232
C	-1.0160342740	-0.9521359953	-0.1426937699
C	-1.3120051043	0.3914822851	0.1045140581
C	-2.6248020625	0.7456662762	0.3839786334
C	-3.6418451492	-0.1975469234	0.3944913755
C	-0.2159171867	1.4229392473	-0.0202821918
C	1.1235281654	0.8199673768	0.3303881018
C	1.3488757165	-0.5358139409	0.0758265850
C	2.6112502639	-1.0880754177	0.2903448339
H	2.7732880016	-2.1409214119	0.0883204337
C	3.6421418996	-0.2980497424	0.7682735829
C	3.4236797078	1.0444696076	1.0502628471
C	2.1667423686	1.5879916979	0.8293267076
H	-4.1216830882	-2.2745435699	0.1240593756
H	-1.7921733876	-2.9447582409	-0.3306974671
H	-2.8516196573	1.7874076194	0.5846084094
H	-4.6603565565	0.1000919378	0.6090990083
H	4.6185861723	-0.7376282010	0.9319326449

H	4.2255161943	1.6620424023	1.4345347621
H	1.9881261254	2.6380185282	1.0354190409
N	0.3009891209	-1.3234481428	-0.3856929822
C	-0.1855011225	2.0006272461	-1.4445610941
H	0.0194216110	1.2145199869	-2.1752235853
H	-1.1461629270	2.4566577057	-1.6929203830
H	0.5932679803	2.7610979811	-1.5324989071
H	-0.4241926446	2.2438329860	0.6697858469
H	0.4803193369	-2.3137605943	-0.4359264405

HCyA9

E(RwB97XD) = -830.818794147

Charge = 0 Multiplicity = 1

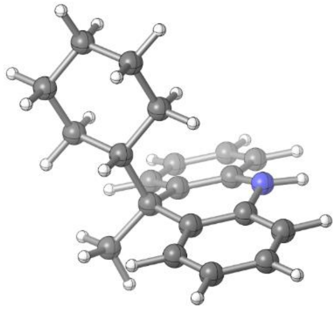

C	-3.1243157550	-2.1414616919	-0.0895172558
C	-1.7733685879	-2.4184367655	-0.1546645483
C	-0.8357299635	-1.3835093544	-0.1382190268
C	-1.2438441622	-0.0459243856	-0.0656021717
C	-2.6136248105	0.1959098430	0.0191625791
C	-3.5514652679	-0.8246704873	0.0047466911
C	-0.2233119665	1.0939390523	-0.1402624087
C	1.1743411057	0.5766739569	0.2188774893
C	1.4842560422	-0.7848721855	0.1522106263
C	2.7803835697	-1.2358103678	0.4120292965
H	2.9892621016	-2.2984116534	0.3554233077
C	3.7809803647	-0.3406034910	0.7345283591
C	3.4950353876	1.0160804043	0.8046260899
C	2.2049729313	1.4531609448	0.5489610595
H	-3.8410188726	-2.9535868557	-0.1033689168
H	-1.4241351834	-3.4433099696	-0.2131761705
H	-2.9706005262	1.2141323136	0.0852981447
H	-4.6067018115	-0.5901696888	0.0637433262
H	4.7826213774	-0.7025050954	0.9322326679
H	4.2701055312	1.7301522920	1.0524133825
H	2.0031358617	2.5154459867	0.5939571866
N	0.5083400437	-1.6983874964	-0.1960808835
C	-0.6063887119	2.2078222485	0.8494328912

H	-0.4935809571	1.8562234139	1.8763958403
H	0.0232966516	3.0878821012	0.7163585968
H	-1.6341124043	2.5385482104	0.7219195394
H	0.7508607780	-2.6731574519	-0.1286083861
C	-0.1433332068	1.6841216682	-1.5940098118
C	0.2603467451	0.6473280970	-2.6460706751
C	-1.4043423658	2.4162202055	-2.0621587043
H	0.6607358813	2.4299917349	-1.5462367458
C	0.4620350026	1.2794820918	-4.0209303286
H	-0.5224888880	-0.1164450085	-2.7138470471
H	1.1772865484	0.1379637116	-2.3445909695
C	-1.1988435169	3.0645997035	-3.4301898058
H	-2.2337568978	1.7058196272	-2.1324141427
H	-1.6974383641	3.1811143503	-1.3411532851
C	-0.7778893017	2.0397228860	-4.4770294162
H	0.7233067451	0.5066797067	-4.7483487916
H	1.3115312799	1.9705449741	-3.9756694933
H	-2.1174364214	3.5691555429	-3.7405062370
H	-0.4254194874	3.8370764193	-3.3501878117
H	-0.5938792794	2.5288202090	-5.4370482770
H	-1.5978495892	1.3294640428	-4.6333288030

A10

E(RwB97XD) = -825.959738491

Charge = 0 Multiplicity = 1

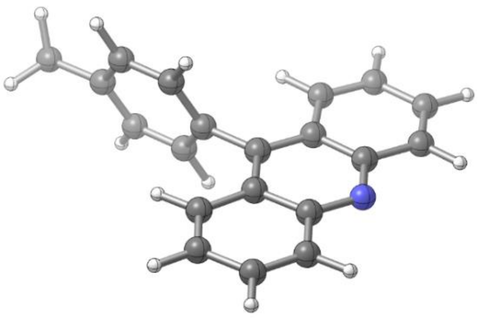

C	-7.9426054034	-1.3429813216	-0.7527099921
C	-6.8000894517	-1.7952709304	-0.1767783466
C	-5.7141283533	-0.9072798698	0.0844789043
C	-5.8528056560	0.4724998063	-0.2635733871
C	-7.0685208309	0.9025349458	-0.8764963320
C	-8.0777126801	0.0253647326	-1.1116633507
C	-4.7826331213	1.3361122329	0.0003082297
C	-3.6252287742	0.8107282573	0.5877123734
C	-3.5903554680	-0.5867803199	0.8880308579
C	-2.4144901612	-1.1332916917	1.4836917408
H	-2.4072658707	-2.1945584724	1.6991499559

C	-1.3492749018	-0.3421051299	1.7682995378
C	-1.3863387471	1.0493682985	1.4821413652
C	-2.4855285826	1.6069898845	0.9128626990
H	-8.7603467779	-2.0260433011	-0.9472572324
H	-6.6761784345	-2.8352457723	0.0986791191
H	-7.1753594742	1.9421036954	-1.1567540415
H	-8.9936109549	0.3646798300	-1.5789247726
H	-0.4620690820	-0.7674733320	2.2211192483
H	-0.5294981012	1.6651248345	1.7251279013
H	-2.5096096775	2.6679299327	0.7018401431
N	-4.6108643829	-1.4093082841	0.6410031457
C	-4.8738838239	2.7812996197	-0.3366951699
C	-4.2165752945	3.2927710642	-1.4506445781
C	-5.6232462894	3.6475031516	0.4526062163
C	-4.3095734086	4.6392928459	-1.7660669214
C	-5.7079850449	4.9939051020	0.1349606235
H	-6.1455494831	3.2640021978	1.3211812197
C	-5.0529909771	5.5138170102	-0.9781716809
H	-3.7956434166	5.0166563355	-2.6430164922
H	-6.2975631326	5.6522429534	0.7631096948
H	-3.6313427488	2.6307070907	-2.0779290187
C	-5.1238800584	6.9787486511	-1.3030711560
H	-4.3187321534	7.5230702835	-0.8017943027
H	-5.0199326558	7.1523418008	-2.3749238758
H	-6.0685116257	7.4124598683	-0.9722443251

HA10

E(UwB97XD) = -826.556629695

Charge = 0 Multiplicity = 2

C	-8.0037392967	-1.3327872753	-0.7517265373
C	-6.8333815262	-1.7907761255	-0.1715086369
C	-5.7804512968	-0.9136443284	0.0707457576
C	-5.8844632449	0.4607057373	-0.2612595660
C	-7.0870762556	0.8859080717	-0.8613893700
C	-8.1265156746	0.0102010457	-1.1002995329
C	-4.7833313938	1.3300564251	0.0026105527

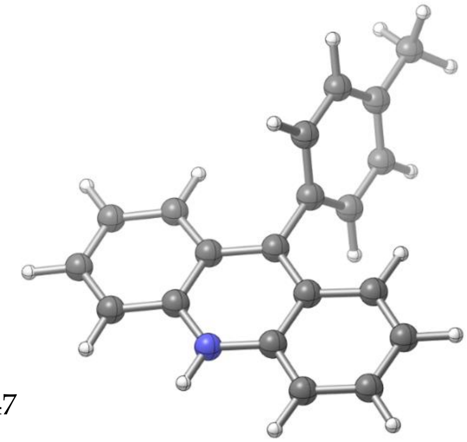

C	-3.5938571636	0.8030738931	0.5902088567
C	-3.5253637853	-0.5782386535	0.9019339413
C	-2.3820010009	-1.1276223301	1.4744772693
H	-2.3612707722	-2.1886457078	1.6964503645
C	-1.2895442993	-0.3261466658	1.7579412442
C	-1.3360884348	1.0367650424	1.4741898566
C	-2.4657552749	1.5878055052	0.9044863376
H	-8.8172566380	-2.0230270231	-0.9362248913
H	-6.7225768145	-2.8350833203	0.0973754250
H	-7.1904756675	1.9257327011	-1.1419496464
H	-9.0370901610	0.3704189506	-1.5624852903
H	-0.4046221040	-0.7626805188	2.2036094929
H	-0.4866993934	1.6683346042	1.7026563067
H	-2.4937956804	2.6486802419	0.6936592666
N	-4.6143575632	-1.3717100134	0.6341420215
C	-4.8742106751	2.7714619972	-0.3330573265
C	-4.1540575083	3.3070144334	-1.3991875855
C	-5.6859628579	3.6303735314	0.4042825328
C	-4.2434217995	4.6536365845	-1.7145395356
C	-5.7726645843	4.9773333915	0.0866857564
H	-6.2592895263	3.2372243256	1.2359291009
C	-5.0522251237	5.5140194628	-0.9764304390
H	-3.6769546139	5.0429264110	-2.5534738436
H	-6.4131356901	5.6232725018	0.6771279457
H	-3.5187752964	2.6581592715	-1.9910716530
C	-5.1223517641	6.9792312705	-1.3019270409
H	-4.3142775834	7.5240216054	-0.8057264345
H	-5.0223188118	7.1519055335	-2.3746171673
H	-6.0654036080	7.4146185587	-0.9689344249
H	-4.5529022152	-2.3512965049	0.8627265133

H_2A10

E(RwB97XD) = -827.182256028

Charge = 0 Multiplicity = 1

C	-7.8026050638	-1.1795334621	-1.1866576158
C	-6.4341514683	-1.3745154001	-1.2115586471

C	-5.5893776619	-0.5162669172	-0.5055052916
C	-6.1251987716	0.5501162326	0.2198279977
C	-7.5040200071	0.7167414698	0.2438474651
C	-8.3483674810	-0.1343862417	-0.4506203519
C	-5.2096590175	1.5373136718	0.9151193457
C	-3.8572932511	0.9194132000	1.2066769575
C	-3.3982326700	-0.1593696038	0.4477276753
C	-2.1180348173	-0.6715844592	0.6657992172
H	-1.7730611104	-1.5081020681	0.0685052185
C	-1.3055192266	-0.1222715892	1.6400842287
C	-1.7604280532	0.9370916015	2.4164783040
C	-3.0298859478	1.4443439410	2.1913800432
H	-8.4472433308	-1.8527277767	-1.7385845103
H	-6.0056750348	-2.1978324574	-1.7719067317
H	-7.9178778472	1.5436615104	0.8109686756
H	-9.4198484854	0.0170075080	-0.4226922810
H	-0.3145123897	-0.5294283725	1.7996433901
H	-1.1300700415	1.3645887165	3.1856914750
H	-3.3903751522	2.2792871981	2.7826387336
N	-4.2174749863	-0.7173199329	-0.5220990050
C	-5.0683850766	2.8203211718	0.1065226432
C	-4.5026345005	2.8120741482	-1.1680857013
C	-5.5044243467	4.0321345415	0.6212113073
C	-4.3808381975	3.9810450099	-1.8960461219
C	-5.3812649481	5.2068670005	-0.1127776291
H	-5.9477646744	4.0640945355	1.6104896499
C	-4.8185307859	5.2028556755	-1.3818635115
H	-3.9377228609	3.9493006160	-2.8856928328
H	-5.7309136600	6.1405598510	0.3138618964
H	-4.1533475830	1.8794679070	-1.5972203975
C	-4.6775800898	6.4648293017	-2.1854911846
H	-3.6280319180	6.6710700495	-2.4088902138
H	-5.2013053606	6.3814121144	-3.1407058630
H	-5.0841363155	7.3233889537	-1.6501806129
H	-5.6691316190	1.8138873606	1.8666117074
H	-3.8898261285	-1.5646702855	-0.9568647980

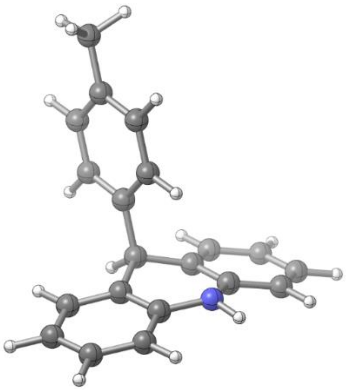

HCyA10

E(RwB97XD) = -1061.87704671

Charge = 0 Multiplicity = 1

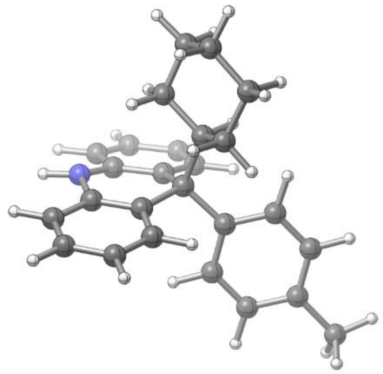

C	-7.7844415465	-0.7962585114	-1.4983964086
C	-6.5259685389	-1.2763233495	-1.1942204779
C	-5.6770634323	-0.5532148158	-0.3530059615
C	-6.0869738553	0.6695349579	0.1840556289
C	-7.3566555790	1.1323526664	-0.1460336995
C	-8.2079983240	0.4174616517	-0.9722752750
C	-5.2014708488	1.4360851556	1.1772188044
C	-3.7501998096	0.9646610237	1.0612030549
C	-3.4389450943	-0.2792524312	0.5069793360
C	-2.1181828973	-0.7364254336	0.4866722174
H	-1.9044920251	-1.7065156549	0.0519218770
C	-1.1011282899	0.0417158367	1.0012997255
C	-1.3881993273	1.2939175646	1.5309997985
C	-2.6993901356	1.7372055123	1.5493382121
H	-8.4331542053	-1.3682628568	-2.1505826496
H	-6.1832778224	-2.2207882692	-1.6017188510
H	-7.6899043142	2.0798788318	0.2592143191
H	-9.1916586802	0.8052304596	-1.2044727052
H	-0.0816979723	-0.3237826154	0.9779795951
H	-0.5980686134	1.9191598924	1.9266720422
H	-2.9180354193	2.7154804724	1.9580099055
N	-4.4359510825	-1.0694648704	-0.0331008384
C	-5.2937650828	2.9272866389	0.8044864482
C	-4.6514273486	3.3706455165	-0.3534652960
C	-6.0564006500	3.8567278980	1.4986722349
C	-4.7528510387	4.6796376075	-0.7836357134
C	-6.1581995400	5.1756080587	1.0674933231
H	-6.5996210736	3.5714286717	2.3889747489
C	-5.5075104374	5.6150068258	-0.0756528097
H	-4.2391178133	4.9833481321	-1.6894853603
H	-6.7630443194	5.8710991676	1.6389749948
H	-4.0618259765	2.6719133014	-0.9350518597

C	-5.5980445832	7.0416044101	-0.5375272182
H	-4.6413396602	7.5543619138	-0.4077833718
H	-5.8533637722	7.0966704224	-1.5979006491
H	-6.3524163105	7.5930151848	0.0247289613
H	-4.1447873563	-1.8979025381	-0.5255771692
C	-5.7421857559	1.1436378432	2.6215586263
C	-5.8621066478	-0.3519441135	2.9367324852
C	-4.9528847888	1.8130310215	3.7524881497
H	-6.7627852813	1.5426023557	2.6394494348
C	-6.5385468058	-0.5834583346	4.2864824316
H	-4.8627140885	-0.8003606019	2.9495937461
H	-6.4331731497	-0.8633271571	2.1610599313
C	-5.6468327352	1.6200445494	5.0985352159
H	-3.9576996246	1.3626696245	3.8069425534
H	-4.8033342990	2.8755929754	3.5590369691
C	-5.8184203465	0.1393304138	5.4192542509
H	-6.5897080396	-1.6554506363	4.4932089545
H	-7.5723911271	-0.2239130187	4.2303603149
H	-5.0711893522	2.1138110861	5.8854656300
H	-6.6298705930	2.1043526022	5.0737574857
H	-6.3627244686	0.0113032874	6.3584010028
H	-4.8291371203	-0.3103793253	5.5607779040

A11

E(RwB97XD) = -1246.25146268

Charge = 0 Multiplicity = 1

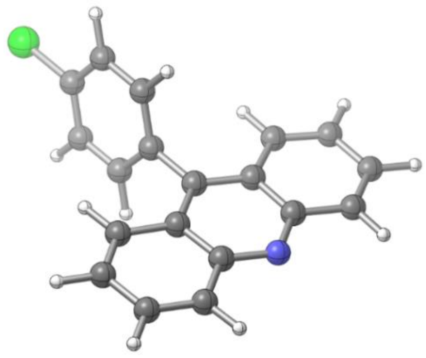

C	-2.7644238463	-3.5617619350	0.1467242425
C	-3.4049139338	-2.3661575189	0.1038804659
C	-2.6675478144	-1.1457663317	0.0509856169
C	-1.2388130538	-1.2037342076	0.0509185931
C	-0.6066172367	-2.4833092768	0.0900483892
C	-1.3445309910	-3.6217756115	0.1368144188
C	-0.5270361023	0.0000000824	0.0000015880
C	-1.2388113547	1.2037351148	-0.0509215420
C	-2.6675461715	1.1457687133	-0.0510011530
C	-3.4049105628	2.3661606705	-0.1039024351

H	-4.4858363744	2.3006513055	-0.1063011523
C	-2.7644188605	3.5617644311	-0.1467404143
C	-1.3445260318	3.6217766407	-0.1368179850
C	-0.6066138685	2.4833095356	-0.0900456123
H	-3.3340358189	-4.4821227251	0.1853424455
H	-4.4858396982	-2.3006470392	0.1062696048
H	0.4741391919	-2.5361463964	0.0785241459
H	-0.8529648825	-4.5860485268	0.1645809166
H	-3.3340295352	4.4821258160	-0.1853635458
H	-0.8529586787	4.5860490512	-0.1645799680
H	0.4741425145	2.5361455314	-0.0785118044
N	-3.3482103340	0.0000015446	-0.0000108020
C	0.9602303285	-0.0000007030	0.0000078550
C	1.6650476645	-0.2020472817	1.1817909037
C	1.6650575715	0.2020451004	-1.1817694172
C	3.0506937228	-0.2013159582	1.1899331993
C	3.0507036908	0.2013120650	-1.1899003585
H	1.1275446303	0.3613431152	-2.1083998994
C	3.7291181359	-0.0000024198	0.0000191739
H	3.5939763907	-0.3562453685	2.1124848223
H	3.5939941604	0.3562408847	-2.1124474830
H	1.1275269929	-0.3613446265	2.1084170149
Cl	5.4724451551	-0.0000036853	0.0000261749

HA11

E(UwB97XD) = -1246.84906160

Charge = 0 Multiplicity = 2

C	-2.7522521200	-3.6175316618	-0.0883018131
C	-3.3998239173	-2.3946681677	-0.0685131918
C	-2.6651606792	-1.2128367615	-0.0361143029
C	-1.2479067587	-1.2342515521	-0.0328274586
C	-0.6256766525	-2.4991167881	-0.0411237073
C	-1.3601303533	-3.6667775458	-0.0701874139
C	-0.5314514537	0.0000000914	0.0000022774
C	-1.2479058413	1.2342524310	0.0328257258
C	-2.6651598205	1.2128390218	0.0360989607

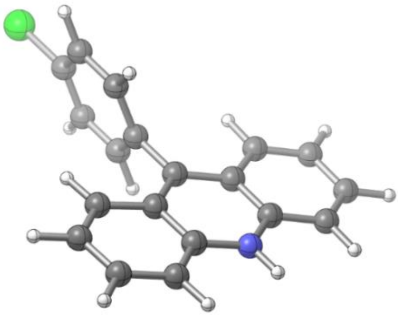

C	-3.3998222087	2.3946711501	0.0684908895
H	-4.4827059771	2.3424849117	0.0745188943
C	-2.7522494133	3.6175340095	0.0882858781
C	-1.3601274251	3.6667785394	0.0701850760
C	-0.6256745889	2.4991170638	0.0411281834
H	-3.3318848188	-4.5316707924	-0.1125837654
H	-4.4827075744	-2.3424808426	-0.0745516140
H	0.4549016886	-2.5502948028	-0.0203973221
H	-0.8502129905	-4.6219449050	-0.0760851927
H	-3.3318814700	4.5316736927	0.1125623938
H	-0.8502091778	4.6219453947	0.0760879931
H	0.4549039961	2.5502940462	0.0204121634
N	-3.3084850766	0.0000014507	-0.0000109091
C	0.9507054981	-0.0000006960	0.0000080662
C	1.6647489162	0.3634235194	1.1401307944
C	1.6647568980	-0.3634257559	-1.1401094121
C	3.0504812051	0.3662382482	1.1492368875
C	3.0504892209	-0.3662420639	-1.1492053498
H	1.1291891981	-0.6483049272	-2.0375505809
C	3.7298090709	-0.0000022628	0.0000182491
H	3.5939092710	0.6474757106	2.0415382844
H	3.5939234398	-0.6474801600	-2.0415027930
H	1.1291749827	0.6483032623	2.0375680636
Cl	5.4744818117	-0.0000031174	0.0000245919
H	-4.3159895728	0.0000019987	-0.0000166662

H_2A11

E(RwB97XD) = -1247.47497639

Charge = 0 Multiplicity = 1

C	-2.6554620689	-3.5811304069	0.1755707995
C	-3.1033410657	-2.3685698404	0.6652721409
C	-2.3793668158	-1.2005540523	0.4189192644
C	-1.1925829228	-1.2577129371	-0.3147531904
C	-0.7702711534	-2.4846602101	-0.8110248003
C	-1.4865663641	-3.6463254782	-0.5739352029
C	-0.3548587167	-0.0107753301	-0.5123415683

C	-1.1880340378	1.2450126108	-0.3549343003
C	-2.3751539753	1.2156093965	0.3798297161
C	-3.0951913844	2.3934438480	0.5876051339
H	-4.0143401159	2.3583860450	1.1616484491
C	-2.6426169537	3.5881933935	0.0599970323
C	-1.4729762370	3.6252669128	-0.6902031369
C	-0.7608644195	2.4540192846	-0.8896327537
H	-3.2266240931	-4.4798783590	0.3739665457
H	-4.0221013017	-2.3115653268	1.2381248692
H	0.1495993049	-2.5244364024	-1.3845690041
H	-1.1369329449	-4.5930232797	-0.9652986072
H	-3.2107211692	4.4948081283	0.2292171378
H	-1.1195263122	4.5577828378	-1.1110871465
H	0.1595034482	2.4723056675	-1.4634359962
N	-2.8332652606	0.0164727889	0.9035494554
C	0.8407632720	0.0030682835	0.4307347978
C	0.6652821641	0.0166920812	1.8126362922
C	2.1343830507	0.0036591260	-0.0736468117
C	1.7506094398	0.0305391143	2.6706094187
C	3.2362421401	0.0175556962	0.7711328499
H	2.2923506001	-0.0068226291	-1.1458140605
C	3.0305460802	0.0308494068	2.1376833246
H	1.6039143347	0.0409228378	3.7425085600
H	4.2396650624	0.0179145876	0.3664519214
H	-0.3339931883	0.0166719684	2.2322700470
Cl	4.4048402929	0.0484448409	3.2134683258
H	0.0465599350	-0.0277320957	-1.5277061282
H	-3.7521253245	0.0245373240	1.3156977760

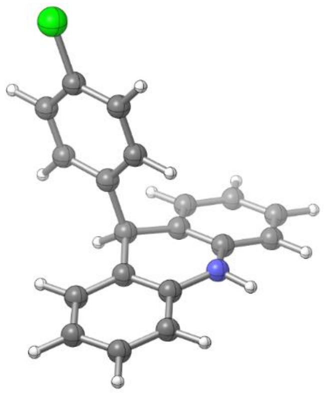

HCyA11

E(RwB97XD) = -1482.16989647

Charge = 0 Multiplicity = 1

C	-3.1091680900	-3.5277317690	0.2393481430
C	-3.5436581340	-2.2893114990	0.6659975380
C	-2.7770840430	-1.1467583330	0.4199050530
C	-1.5538889830	-1.2449467400	-0.2471010160

C	-1.1541684410	-2.5042849480	-0.6873071170
C	-1.9084172770	-3.6404498040	-0.4510287320
C	-0.6590760600	-0.0183993240	-0.4359051770
C	-1.4881562760	1.2675764510	-0.3063759470
C	-2.7239965750	1.2644754590	0.3450330230
C	-3.4299557950	2.4560193900	0.5267271420
H	-4.3885367320	2.4278753920	1.0327633820
C	-2.9118306260	3.6521860620	0.0714292910
C	-1.6842452090	3.6727195570	-0.5782809660
C	-0.9940730970	2.4860148320	-0.7612060310
H	-3.7136530710	-4.4049067220	0.4355552200
H	-4.4884801200	-2.1854858580	1.1876208020
H	-0.2212751980	-2.5957096980	-1.2289378360
H	-1.5624941730	-4.6044258420	-0.8013860630
H	-3.4689886520	4.5690799800	0.2202927930
H	-1.2684762560	4.6040350700	-0.9409466700
H	-0.0378871620	2.5078582910	-1.2695010160
N	-3.2442915770	0.0840424420	0.8388909540
C	-0.0664625940	-0.0264415010	-1.8566518540
C	-0.9183374300	-0.2736321180	-2.9335871490
C	1.2568632930	0.2846964310	-2.1446421730
C	-0.4744138080	-0.2314422300	-4.2421090090
C	1.7255421910	0.3313369220	-3.4516732820
H	1.9581121450	0.5075402680	-1.3533109890
C	0.8552120790	0.0688123090	-4.4904065540
H	-1.1559182620	-0.4273008490	-5.0592731020
H	2.7614220780	0.5726394090	-3.6500989840
H	-1.9606768600	-0.5020329880	-2.7490439290
Cl	1.4328774110	0.1212828870	-6.1361093060
H	-4.1800048620	0.1155229440	1.2092169670
C	0.4478529340	0.0124178270	0.6765224810
C	-0.1141316810	0.2393142230	2.0847235430
C	1.3602203770	-1.2191286730	0.7128675190
H	1.0678212440	0.8875493380	0.4498584000
C	1.0053217330	0.4177926540	3.1082408990
H	-0.7337818750	-0.6180137790	2.3693577750

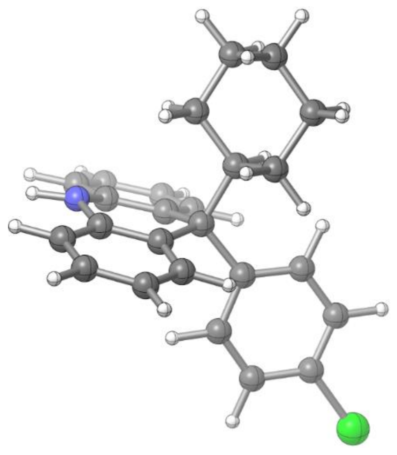

H	-0.7567794120	1.1201988150	2.1035534390
C	2.5047678570	-1.0284230390	1.7050653560
H	0.7732123290	-2.0879478280	1.0234841950
H	1.7587984860	-1.4511367750	-0.2752838220
C	1.9735297720	-0.7597932810	3.1087981160
H	0.5749224430	0.5550725830	4.1033774010
H	1.5559714990	1.3359540260	2.8738424990
H	3.1445993790	-1.9143795510	1.7051917000
H	3.1289995680	-0.1866207300	1.3838924010
H	2.7983233960	-0.5732448720	3.8012225120
H	1.4517734270	-1.6539044140	3.4683030250

A12

E(RwB97XD) = -885.894402865

Charge = 0 Multiplicity = 1

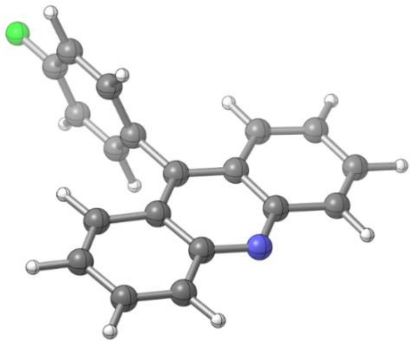

C	-2.4297672309	-3.5643864482	-0.0500505313
C	-3.0695651821	-2.3676842846	-0.0410772109
C	-2.3316654241	-1.1465900463	-0.0203022126
C	-0.9029280647	-1.2045253791	-0.0174935398
C	-0.2717947386	-2.4853094602	-0.0184671413
C	-1.0099840004	-3.6245021173	-0.0348514609
C	-0.1898037024	0.0000000582	0.0000003347
C	-0.9029279934	1.2045255326	0.0174945669
C	-2.3316653541	1.1465902739	0.0203039728
C	-3.0695650383	2.3676845507	0.0410793442
H	-4.1504484250	2.3016085422	0.0471216304
C	-2.4297670206	3.5643866814	0.0500523289
C	-1.0099837950	3.6245022767	0.0348525269
C	-0.2717946006	2.4853095812	0.0184678403
H	-2.9998365288	-4.4851762734	-0.0646838890
H	-4.1504485685	-2.3016082201	-0.0471189408
H	0.8088070427	-2.5382331810	-0.0022414810
H	-0.5186568806	-4.5893108607	-0.0335352481
H	-2.9998362633	4.4851765362	0.0646859711
H	-0.5186566253	4.5893109944	0.0335360533
H	0.8088071752	2.5382332457	0.0022416273

N	-3.0125320530	0.0000001315	0.0000010569
C	1.2975140629	0.0000000207	-0.0000000277
C	2.0021693658	0.2743550367	1.1681734498
C	2.0021688014	-0.2743550261	-1.1681738384
C	3.3882471903	0.2743239635	1.1780456408
C	3.3882466216	-0.2743240066	-1.1780466849
H	1.4627587808	-0.4898142165	-2.0822657506
C	4.0520760995	-0.0000000330	-0.0000006791
H	3.9464489212	0.4835448217	2.0811200070
H	3.9464479169	-0.4835448847	-2.0811213160
H	1.4627597829	0.4898142459	2.0822656159
F	5.3981757287	-0.0000000559	-0.0000009993

HA12

E(UwB97XD) = -886.491765998

Charge = 0 Multiplicity = 2

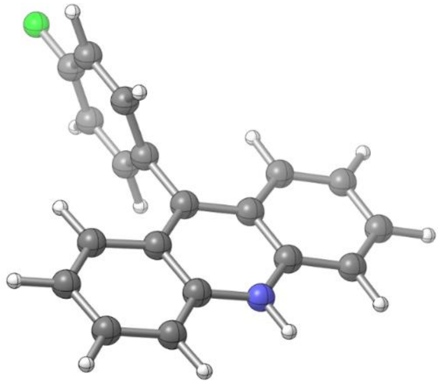

C	-2.4159280059	-3.6179941220	-0.0776891720
C	-3.0641337015	-2.3951729326	-0.0606011744
C	-2.3300183044	-1.2130237476	-0.0318914607
C	-0.9126343479	-1.2338797793	-0.0286128879
C	-0.2898141803	-2.4984360781	-0.0354609278
C	-1.0238185750	-3.6666192562	-0.0611215604
C	-0.1957862822	0.0000000636	0.0000003040
C	-0.9126341832	1.2338799940	0.0286138603
C	-2.3300181393	1.2130241340	0.0318932199
C	-3.0641333766	2.3951734081	0.0606033380
H	-4.1470692600	2.3436112073	0.0662252266
C	-2.4159275239	3.6179945196	0.0776909658
C	-1.0238180964	3.6666194848	0.0611225641
C	-0.2898138580	2.4984362171	0.0354615386
H	-2.9952393677	-4.5324199530	-0.0992042752
H	-4.1470695817	-2.3436106005	-0.0662224589
H	0.7909162323	-2.5480897215	-0.0168912111
H	-0.5135628263	-4.6216375262	-0.0658904361
H	-2.9952387634	4.5324204205	0.0992063843
H	-0.5135622285	4.6216376926	0.0658911401

H	0.7909165505	2.5480897282	0.0168912192
N	-2.9734743019	0.0000002322	0.0000010645
C	1.2872261776	-0.0000000209	-0.0000000402
C	2.0002399900	0.3401884315	1.1483683659
C	2.0002394438	-0.3401885451	-1.1483687630
C	3.3866893066	0.3435825340	1.1590319168
C	3.3866887566	-0.3435827828	-1.1590329301
H	1.4625219484	-0.6068158580	-2.0501338048
C	4.0513128371	-0.0000001559	-0.0000006547
H	3.9447528127	0.6048859620	2.0487297716
H	3.9447518423	-0.6048862640	-2.0487310330
H	1.4625229201	0.6068157978	2.0501336457
F	5.3991905774	-0.0000002196	-0.0000009556
H	-3.9809589098	0.0000002933	0.0000013917

H_2A12

E(RwB97XD) = -887.117523089

Charge = 0 Multiplicity = 1

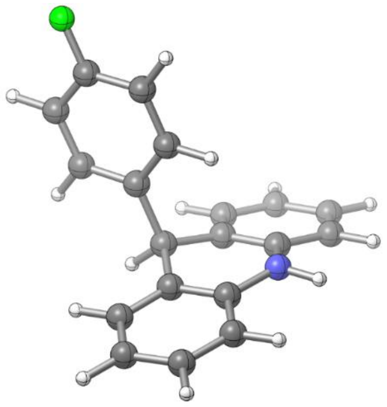

C	-2.2699157717	-3.6021357944	-0.0697660870
C	-2.7337702476	-2.4110032116	-0.5958006287
C	-2.0263706521	-1.2261972913	-0.3840832419
C	-0.8400679653	-1.2444925181	0.3524534298
C	-0.4017380305	-2.4502265793	0.8855194743
C	-1.1013708699	-3.6284345482	0.6825925807
C	-0.0195171797	0.0193166962	0.5125664368
C	-0.8709535371	1.2574771938	0.3183360570
C	-2.0561893631	1.1898810552	-0.4169947244
C	-2.7926389908	2.3505870129	-0.6610769057
H	-3.7098022120	2.2849858419	-1.2355381393
C	-2.3587947419	3.5665855976	-0.1672784052
C	-1.1917061228	3.6421733109	0.5840929189
C	-0.4630737219	2.4876463484	0.8187156870
H	-2.8282931674	-4.5142523452	-0.2418354775
H	-3.6522278707	-2.3843254593	-1.1714654663
H	0.5179110277	-2.4603119203	1.4607187500
H	-0.7390985461	-4.5580426145	1.1024545228

H	-2.9394425208	4.4596399859	-0.3638572004
H	-0.8529224262	4.5914561944	0.9787865603
H	0.4556685160	2.5359015997	1.3934507179
N	-2.4961906346	-0.0306545072	-0.9058738196
C	1.1786507331	0.0199648337	-0.4283185657
C	2.4713043038	0.0271513976	0.0798394048
C	1.0050486157	0.0114706085	-1.8110754888
C	3.5766372704	0.0259985911	-0.7618278609
C	2.0918149403	0.0102410732	-2.6684751991
H	0.0057725144	0.0055755981	-2.2306908082
C	3.3607006611	0.0175743353	-2.1219972960
H	4.5856788195	0.0317381635	-0.3705696189
H	1.9639614416	0.0038130849	-3.7431409221
H	2.6248884518	0.0338106275	1.1526677277
F	4.4240625432	0.0165842261	-2.9516717003
H	0.3800869135	0.0380888288	1.5286938653
H	-3.4145767900	-0.0473401322	-1.3188465271

HCyA12

E(RwB97XD) = -1121.81232721

Charge = 0 Multiplicity = 1

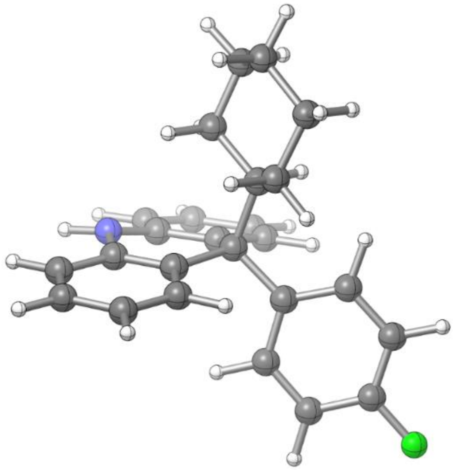

C	-2.5727263510	-3.5154032416	0.5209598808
C	-2.9509035285	-2.2721217108	0.9855617805
C	-2.2400541970	-1.1291499733	0.6086957500
C	-1.1287957614	-1.2311351932	-0.2314386648
C	-0.7869271568	-2.4958834573	-0.7037395636
C	-1.4870615427	-3.6325610164	-0.3384877633
C	-0.2847051444	-0.0017727867	-0.5744478300
C	-1.0998261230	1.2795231108	-0.3472238709
C	-2.2247453034	1.2802691830	0.4810561575
C	-2.9099719120	2.4693185596	0.7412224975
H	-3.7829919773	2.4440705302	1.3838816027
C	-2.4784547627	3.6600604452	0.1911302943
C	-1.3616815783	3.6768669011	-0.6346644158
C	-0.6932624562	2.4921069138	-0.8949099140
H	-3.1333845648	-4.3928673651	0.8194139631

H	-3.8090966820	-2.1658981932	1.6397188456
H	0.0553026066	-2.5905483456	-1.3772172333
H	-1.1876572801	-4.6005608910	-0.7193938019
H	-3.0180382655	4.5750831170	0.4031075861
H	-1.0148166394	4.6036716511	-1.0734203643
H	0.1752845543	2.5099325935	-1.5417963423
N	-2.6518564531	0.1062609735	1.0712979060
C	0.0945189508	-0.0342510397	-2.0668743462
C	-0.9048871816	-0.3089142120	-3.0017663122
C	1.3582223199	0.2831651852	-2.5516794689
C	-0.6606189249	-0.2880527467	-4.3630148802
C	1.6302498603	0.3098241536	-3.9146291865
H	2.1654588990	0.5272511497	-1.8761092384
C	0.6140625624	0.0194301218	-4.7951685285
H	-1.4426058976	-0.5026020658	-5.0797536572
H	2.6177493756	0.5548279262	-4.2835543954
H	-1.9055718710	-0.5420924207	-2.6598192200
H	-3.5257732167	0.1381479707	1.5704358860
C	0.9729697487	0.0591728671	0.3629021058
C	0.6244574338	0.3123932294	1.8342595602
C	1.8916306112	-1.1659452995	0.2881886507
H	1.5458219875	0.9324743516	0.0303976544
C	1.8825348521	0.5201345643	2.6751511992
H	0.0619707000	-0.5424020573	2.2253548445
H	-0.0160379647	1.1895481281	1.9319288837
C	3.1699492446	-0.9462487863	1.0931919548
H	1.3657247285	-2.0311103845	0.7015224321
H	2.1392330539	-1.4165966705	-0.7438317057
C	2.8521087991	-0.6500739039	2.5547101606
H	1.6044751132	0.6757239605	3.7205642804
H	2.3825697784	1.4369809907	2.3426232870
H	3.8112518352	-1.8277904436	1.0162000282
H	3.7308030476	-0.1078088625	0.6642851429
H	3.7693020069	-0.4421209635	3.1115769857
H	2.3996413624	-1.5397390579	3.0070887421
F	0.8672110549	0.0398085269	-6.1195699959

A13

E(RwB97XD) = -865.277995173

Charge = 0 Multiplicity = 1

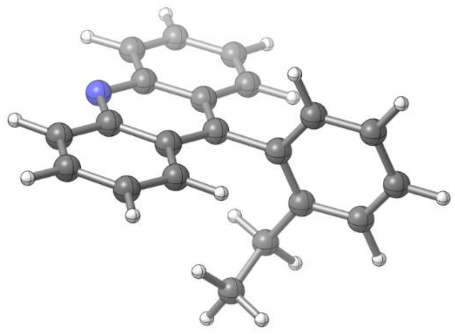

C	-8.0804420106	-1.3302305847	-0.7820135225
C	-6.9895338219	-1.8072031198	-0.1304513167
C	-5.8882003140	-0.9513441375	0.1699983726
C	-5.9538672946	0.4195363677	-0.2276221291
C	-7.1207422802	0.8797337191	-0.9078992074
C	-8.1479384339	0.0334756472	-1.1764843858
C	-4.8663167784	1.2507500613	0.0644958737
C	-3.7704064093	0.7068239186	0.7427682848
C	-3.8081783997	-0.6782218426	1.0955561062
C	-2.6913576715	-1.2433007671	1.7814185638
H	-2.7392146530	-2.2939020380	2.0396913831
C	-1.6121441304	-0.4823642718	2.0936059777
C	-1.5722949389	0.8946496368	1.7427369484
C	-2.6145549878	1.4696034766	1.0906020306
H	-8.9107909369	-1.9887153188	-1.0056519373
H	-6.9207619717	-2.8427715878	0.1787909457
H	-7.1762050653	1.9182104086	-1.2077196217
H	-9.0282705471	0.3945461773	-1.6932036783
H	-0.7698100118	-0.9219420850	2.6135694785
H	-0.7006982927	1.4835913669	1.9992293733
H	-2.5789023189	2.5172622797	0.8220973798
N	-4.8405245750	-1.4727469445	0.8107638850
C	-4.8844658563	2.6907638039	-0.3177299675
C	-4.5326652448	3.1098125289	-1.6053180604
C	-5.2659448174	3.6270995808	0.6401437316
C	-4.5720864251	4.4736487112	-1.8885445281
C	-5.3008291564	4.9784455996	0.3392922959
C	-4.9499867476	5.4032270201	-0.9335863864
H	-4.3024597194	4.8074686333	-2.8845770195
H	-5.6025765114	5.6937802161	1.0941241233
C	-4.0812084775	2.1430925692	-2.6706602256
H	-4.5940675887	1.1870340054	-2.5538872362

H	-4.3708151970	2.5389593949	-3.6464001060
H	-4.9743725145	6.4568139742	-1.1838314158
H	-5.5388522472	3.2856705889	1.6318222369
C	-2.5706264462	1.9113593146	-2.6515388151
H	-2.2769258226	1.2288754040	-3.4515136189
H	-2.0309006630	2.8507813150	-2.7910590051
H	-2.2504299315	1.4769643977	-1.7027781872

HA13

E(UwB97XD) = -865.875068097

Charge = 0 Multiplicity = 2

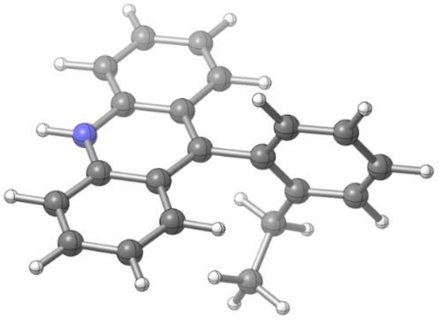

C	-7.8104489780	-1.3499899732	-1.0532317747
C	-6.6944279514	-1.7967244695	-0.3664332585
C	-5.6826176844	-0.9065542309	-0.0189171627
C	-5.7725837158	0.4660789084	-0.3589647918
C	-6.9249807542	0.8837157416	-1.0536989077
C	-7.9240664224	-0.0045251461	-1.3961655037
C	-4.7151311793	1.3495486737	0.0082089882
C	-3.5921922447	0.8445421544	0.7263086624
C	-3.5369144659	-0.5340140606	1.0515151497
C	-2.4530085411	-1.0619307398	1.7468211600
H	-2.4414762722	-2.1203783184	1.9812597307
C	-1.4044862152	-0.2431273076	2.1298777710
C	-1.4322035296	1.1144407320	1.8175539406
C	-2.5040930070	1.6449021139	1.1295874007
H	-8.5923825045	-2.0499709117	-1.3194677997
H	-6.5961238560	-2.8409077307	-0.0921777905
H	-7.0207176968	1.9284044823	-1.3205357576
H	-8.7973300440	0.3476480155	-1.9308522468
H	-0.5652652906	-0.6632756585	2.6697506592
H	-0.6112879122	1.7561796422	2.1121511781
H	-2.5162551955	2.6993504503	0.8862726052
N	-4.5745960957	-1.3465931143	0.6636948887
C	-4.8010181149	2.7964580877	-0.3218225139
C	-4.4880903095	3.2820912607	-1.5987038621
C	-5.2075195918	3.6894695156	0.6688046680

C	-4.5923068204	4.6510458186	-1.8365462025
C	-5.3052099114	5.0483888139	0.4169460950
C	-4.9948129746	5.5317801921	-0.8452724271
H	-4.3524877845	5.0298642841	-2.8242693281
H	-5.6253141052	5.7238552104	1.2008041204
C	-4.0059381417	2.3723010979	-2.6999388288
H	-4.5179917063	1.4103865817	-2.6415208184
H	-4.2714337700	2.8147133113	-3.6626552089
H	-5.0688930889	6.5912049980	-1.0591310872
H	-5.4504100259	3.3043120342	1.6525527945
C	-2.4956063071	2.1431539282	-2.6518821496
H	-2.1779210674	1.4953213691	-3.4715457421
H	-1.9555062095	3.0889407434	-2.7377257746
H	-2.1994815613	1.6710804601	-1.7132551355
H	-4.5249792012	-2.3238644240	0.9046476293

H_2A13

E(RwB97XD) = -866.498015991

Charge = 0 Multiplicity = 1

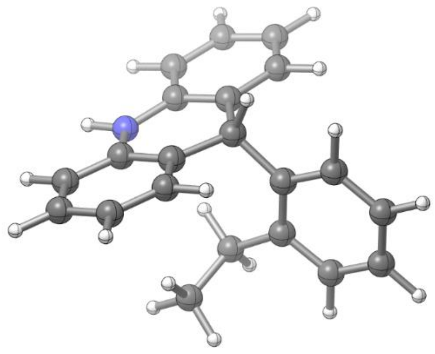

C	-7.8615344094	-1.5008730060	-0.9209179820
C	-6.7442691834	-1.9219123770	-0.2258552196
C	-5.7097107868	-1.0270392624	0.0579342531
C	-5.8079341848	0.3045594697	-0.3491768504
C	-6.9354557105	0.7006247716	-1.0580770183
C	-7.9620493128	-0.1823238760	-1.3487963441
C	-4.7244417533	1.3159301821	-0.0231972721
C	-3.5016775429	0.6770946260	0.6146121853
C	-3.4851261150	-0.6747031190	0.9661235631
C	-2.3434985583	-1.2316723335	1.5478547437
H	-2.3482072775	-2.2813118738	1.8196556734
C	-1.2242272216	-0.4539801088	1.7741101862
C	-1.2250891963	0.8902418329	1.4213125256
C	-2.3610325863	1.4363312425	0.8469344398
H	-8.6562588184	-2.2060845217	-1.1315858991
H	-6.6592162514	-2.9501489629	0.1072647122
H	-7.0120493099	1.7352203983	-1.3744790173

H	-8.8323278222	0.1533266377	-1.8979776318
H	-0.3467601207	-0.9000814671	2.2261619351
H	-0.3509950989	1.5050457446	1.5935361856
H	-2.3724797447	2.4860343593	0.5744173755
N	-4.6027058501	-1.4608216052	0.7613654832
C	-5.2490719735	2.5122633640	0.7699356408
C	-5.8274231370	2.4115742438	2.0439873582
C	-5.1306027013	3.7711734556	0.1824461082
C	-6.2545764147	3.5794058906	2.6742493604
C	-5.5640208917	4.9220988883	0.8206509828
C	-6.1307605958	4.8248145263	2.0810328248
H	-6.6946997547	3.5020927649	3.6629372037
H	-5.4557675160	5.8850695418	0.3366952552
C	-6.0465868480	1.1070309603	2.7718533198
H	-5.3779458058	0.3336780017	2.4024209943
H	-5.7887218749	1.2542196155	3.8237141703
H	-6.4718227687	5.7113940131	2.6017252040
H	-4.6846419979	3.8475600180	-0.8030846695
C	-7.4911199837	0.6140248881	2.6824792071
H	-7.6078404220	-0.3279157520	3.2226403676
H	-8.1793347599	1.3412366687	3.1191000981
H	-7.7885817207	0.4494685867	1.6453711004
H	-4.3912238498	1.7334892821	-0.9772807493
H	-4.5372650890	-2.4403208686	0.9810836762

HCyA13

E(RwB97XD) = -1101.18822459

Charge = 0 Multiplicity = 1

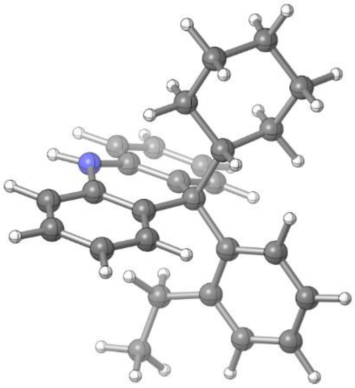

C	-7.5206743825	-1.8853718098	-0.4396475153
C	-6.4650215400	-2.0520698525	0.4352902697
C	-5.4852656741	-1.0642808356	0.5576150747
C	-5.5708622928	0.1120549417	-0.1896487395
C	-6.6326712656	0.2435387262	-1.0774926399
C	-7.6050883930	-0.7334222867	-1.2111350676
C	-4.5458638937	1.2316618276	-0.0121244609
C	-3.2664284607	0.6994224596	0.6462350062

C	-3.2664262996	-0.5084607733	1.3486678379
C	-2.1129500075	-0.9438566888	2.0030422609
H	-2.1335475465	-1.8871326588	2.5373355341
C	-0.9608698255	-0.1811883003	1.9698899638
C	-0.9451967151	1.0217315963	1.2769377395
C	-2.0929718445	1.4429418572	0.6237740654
H	-8.2715071004	-2.6611759849	-0.5277614217
H	-6.3773706036	-2.9566149591	1.0266097571
H	-6.6934963389	1.1358817437	-1.6876757278
H	-8.4201302097	-0.5968739804	-1.9103272646
H	-0.0730234791	-0.5299825920	2.4831680960
H	-0.0482359794	1.6268875906	1.2441190233
H	-2.0784863780	2.3805285619	0.0813911508
N	-4.4165525655	-1.2718099136	1.4103430272
C	-4.1501212791	1.8247586649	-1.3877403254
C	-3.6951449871	1.0019837901	-2.4464083121
C	-4.1586527086	3.2024189563	-1.5925833213
C	-3.3260524264	1.6053594374	-3.6461104126
C	-3.7807222338	3.7836700676	-2.7939872658
C	-3.3708991059	2.9775616451	-3.8361706100
H	-2.9804300261	0.9859898133	-4.4625721959
H	-3.8079164536	4.8611547178	-2.9016215822
C	-3.5768673001	-0.5058193580	-2.3283007831
H	-3.1975494748	-0.7532127343	-1.3373317876
H	-4.5760677373	-0.9450082788	-2.3763071021
H	-3.0745592159	3.4042508398	-4.7867747632
H	-4.4579612517	3.8652157588	-0.7954968057
C	-2.6903460907	-1.1957184008	-3.3571202857
H	-2.6051896502	-2.2546924987	-3.1062425766
H	-3.0984090338	-1.1347241421	-4.3676383645
H	-1.6828536933	-0.7735848458	-3.3709759783
H	-4.3434452019	-2.1790380944	1.8403315521
C	-5.1500127299	2.3073731945	0.9705023522
C	-6.4979895031	2.9018309340	0.5403082331
C	-5.2993406537	1.8042863322	2.4127800218
H	-4.4144737942	3.1176614711	1.0123241302

C -6.9021456142 4.0628510355 1.4452358625
H -7.2651164742 2.1260110969 0.6104028285
H -6.4834893138 3.2278589875 -0.4993949014
C -5.7216264860 2.9333278864 3.3516781295
H -6.0473745341 1.0048810314 2.4445799108
H -4.3617502178 1.3818261119 2.7739358634
C -7.0092325459 3.6105266256 2.8970915028
H -7.8540331164 4.4800943728 1.1072428246
H -6.1583870035 4.8640579808 1.3649645216
H -5.8368729994 2.5425325836 4.3658582308
H -4.9183140493 3.6778794584 3.3909458052
H -7.2432313559 4.4574917627 3.5471419317
H -7.8395497585 2.9010413075 2.9870787317

A14

E(RwB97XD) = -673.526043790

Charge = 0 Multiplicity = 1

C -3.5700890447 -0.8500180830 0.0136468960
C -2.4009259951 -1.5314799642 -0.0553895338
C -1.1490524232 -0.8450397299 -0.0682796995
C -1.1219691767 0.5861418474 -0.0051659964
C -2.3872840550 1.2543906386 0.0669365364
C -3.5585219382 0.5674001799 0.0753630993
C 0.1262021342 1.2430573436 -0.0191455794
C 1.2817712448 0.4409356322 -0.1016966892
C 1.1325655027 -0.9815310563 -0.1572815765
C 2.2909499978 -1.8123976377 -0.2355146148
H 2.1317217896 -2.8828578224 -0.2736315479
C 3.5348786653 -1.2751862322 -0.2609222343
C 3.6969581775 0.1334617342 -0.2109090515
C 2.6177992854 0.9538737215 -0.1347617157
H -4.5145557980 -1.3799418931 0.0219095373
H -2.3738611758 -2.6129656412 -0.1040250524
H -2.4324493696 2.3316356374 0.1144615039
H -4.4960863177 1.1064052409 0.1294590191
H 4.4075011175 -1.9139523955 -0.3198663254

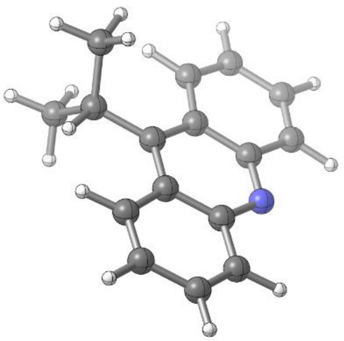

H	4.6932540082	0.5569401567	-0.2342733489
H	2.7859531758	2.0170822776	-0.1005567656
N	-0.0504336819	-1.5934347971	-0.1395652223
C	0.1908963550	2.7608317438	0.0545868821
H	-0.8319059208	3.1160718334	0.1235912788
C	0.7399810798	3.4050542002	-1.2235042247
H	1.7984552570	3.2089042529	-1.3886342939
H	0.1934779237	3.0476658970	-2.0986619695
H	0.6086736868	4.4877878831	-1.1654807471
C	0.8654016298	3.2784820328	1.3305337129
H	0.7186258992	4.3585312931	1.4003267015
H	0.4161368823	2.8217721909	2.2147497848
H	1.9372382442	3.0886621651	1.3650339371

HA14

E(UwB97XD) = -674.121805531

Charge = 0 Multiplicity = 2

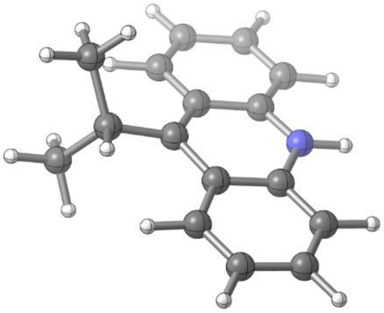

C	-3.6124562657	-0.8125876281	-0.1180101299
C	-2.4251050995	-1.5165214833	-0.0277111665
C	-1.2073024275	-0.8432257550	-0.0006192931
C	-1.1401705077	0.5738185084	-0.0424844272
C	-2.3757824154	1.2487054094	-0.1569978465
C	-3.5813042908	0.5764111312	-0.1922708818
C	0.1361849491	1.2282217412	0.0223045252
C	1.3176891626	0.4218654669	-0.0872281472
C	1.2066023781	-0.9925099095	-0.0414492912
C	2.3277580142	-1.8150811433	-0.1097295510
H	2.1923188650	-2.8896415956	-0.0588765650
C	3.5905819177	-1.2682739511	-0.2462243538
C	3.7325079020	0.1133824532	-0.3276057362
C	2.6236289178	0.9322573372	-0.2523871021
H	-4.5553302358	-1.3442902826	-0.1396165835
H	-2.4272182688	-2.5995304168	0.0197741799
H	-2.3914144103	2.3263966257	-0.2352843036
H	-4.5033244715	1.1372798319	-0.2817006199
H	4.4570829345	-1.9152819860	-0.2993711071

H	4.7137287876	0.5536361481	-0.4543244662
H	2.7709431415	1.9965157412	-0.3388702413
N	-0.0382122663	-1.5567444663	0.0833687506
C	0.1866235353	2.7342143449	0.1915218828
H	-0.8203559077	3.0408160887	0.4683365743
C	0.5074494478	3.4787779628	-1.1101305119
H	1.5150561365	3.2682234658	-1.4718286963
H	-0.1940602202	3.1978429123	-1.8987202505
H	0.4276720204	4.5578933285	-0.9559694241
C	1.0551373446	3.2116816413	1.3631154696
H	0.8425573048	4.2649125903	1.5616323959
H	0.8258609737	2.6480231524	2.2699415289
H	2.1248713838	3.1260979069	1.1784916639
H	-0.0993705196	-2.5623455405	0.1057376947

H_2A14

E(RwB97XD) = -674.757102489

Charge = 0 Multiplicity = 1

C	-3.4955787384	-0.9045407640	0.0250902096
C	-2.3290199809	-1.3615667951	-0.5635900494
C	-1.1666444761	-0.5966515321	-0.4899548737
C	-1.1787165664	0.6460820335	0.1507699714
C	-2.3503818161	1.0667143940	0.7649832655
C	-3.5086187122	0.3054189172	0.7073809238
C	0.0691397508	1.4934639141	0.0849243287
C	1.2723422695	0.5886765481	0.2005733499
C	1.2282851962	-0.6524662127	-0.4419253247
C	2.3561568659	-1.4703427814	-0.4704110625
H	2.3085061830	-2.4273458778	-0.9778767164
C	3.5178783279	-1.0680297102	0.1658385879
C	3.5595253497	0.1400969295	0.8502203453
C	2.4364922137	0.9544298535	0.8624017088
H	-4.3942727053	-1.5064621785	-0.0340173260
H	-2.3055348379	-2.3206778670	-1.0686635932
H	-2.3561177630	2.0211447987	1.2794059620
H	-4.4153693204	0.6561886826	1.1836763496

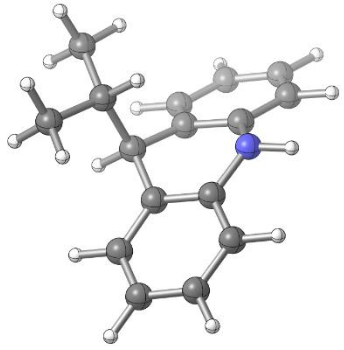

H	4.3893769787	-1.7109704690	0.1417336183
H	4.4615193586	0.4482270381	1.3635860532
H	2.4658626725	1.9077991230	1.3780097859
N	0.0318700623	-1.0610143329	-1.0266795848
C	0.1156777748	2.3283933372	-1.2309715892
H	0.1170251507	1.6190832384	-2.0655637766
C	1.3848043324	3.1704532687	-1.3174777786
H	1.4493934930	3.8624814583	-0.4719034872
H	2.2877178920	2.5596175165	-1.3231435067
H	1.3804690600	3.7652102314	-2.2335188670
C	-1.1090810951	3.2272025651	-1.3699280552
H	-1.0397933953	3.8194545524	-2.2849676684
H	-2.0378301670	2.6580256047	-1.4129946938
H	-1.1771120682	3.9229669377	-0.5276938431
H	0.0687973769	2.2030272954	0.9176165795
H	0.0166092841	-2.0097413274	-1.3673366330

HCyA14

E(RwB97XD) = -909.446517454

Charge = 0 Multiplicity = 1

C	-3.6229505706	-0.7774156978	-0.1963500888
C	-2.4298954751	-1.4671694299	-0.1405915975
C	-1.2137024188	-0.7819246574	-0.0544625660
C	-1.1765624670	0.6156231432	-0.0026069931
C	-2.4005076760	1.2783430131	-0.0917493107
C	-3.6109377146	0.6102732929	-0.1805968172
C	0.1435118756	1.3991667120	0.1018768593
C	1.3484889753	0.4428326351	0.1399540788
C	1.2021922786	-0.9450328797	0.0400356872
C	2.3172377936	-1.7893001194	0.0509933793
H	2.1635203192	-2.8591122120	-0.0383547028
C	3.5889943259	-1.2730576260	0.1855753345
C	3.7581191032	0.0974044812	0.3247354022
C	2.6469076621	0.9245515803	0.3059294687
H	-4.5573189900	-1.3211937649	-0.2640963113
H	-2.4184580615	-2.5510146514	-0.1735827895

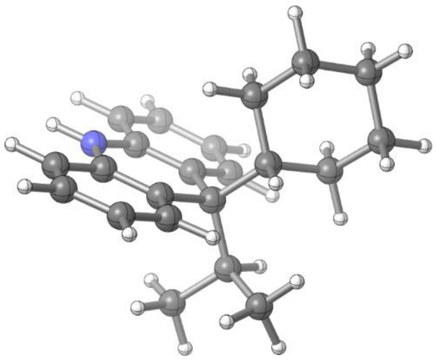

H	-2.4185440829	2.3578753559	-0.1065840494
H	-4.5345500707	1.1715118366	-0.2432418495
H	4.4433178348	-1.9389592647	0.1937384293
H	4.7457456552	0.5221994395	0.4518115232
H	2.8064726932	1.9843709146	0.4383402667
N	-0.0479906221	-1.5118201462	-0.0492603108
C	0.1846634111	2.2269647672	1.4385624900
H	1.1904518568	2.6463250331	1.4869754217
C	0.0151971334	1.3269041212	2.6610304037
H	-0.9865772566	0.8905482394	2.6902657480
H	0.7397372894	0.5109348015	2.6736715341
H	0.1504426915	1.9074668588	3.5765023456
C	-0.7735103951	3.4136519786	1.5572192129
H	-0.4444003963	4.0492001307	2.3829211195
H	-0.8035910531	4.0340405833	0.6606691142
H	-1.7910251339	3.0955510868	1.7876252208
H	-0.1132876570	-2.5141287884	-0.0962499281
C	0.2137326790	2.3171122032	-1.1698260970
C	1.2975128095	3.3998199908	-1.2102455194
C	0.2829195910	1.5030553662	-2.4664597586
H	-0.7386665666	2.8541201647	-1.1924986468
C	1.0852927253	4.3212775359	-2.4108957295
H	2.2837390523	2.9406581695	-1.3083832128
H	1.3052778968	3.9876747123	-0.2905451535
C	0.0990179436	2.3926313693	-3.6936450429
H	1.2546555657	1.0002079443	-2.5231398875
H	-0.4788093627	0.7203621318	-2.4646948745
C	1.1085317296	3.5359354380	-3.7186974545
H	1.8549612920	5.0972149197	-2.4235201811
H	0.1210901969	4.8326410684	-2.3094465602
H	0.1834486750	1.7941215390	-4.6043525029
H	-0.9153126375	2.8076719963	-3.6818977615
H	0.9107542550	4.1987202531	-4.5650909247
H	2.1130447272	3.1233048510	-3.8664618580

A15

E(RwB97XD) = -825.959880405

Charge = 0 Multiplicity = 1

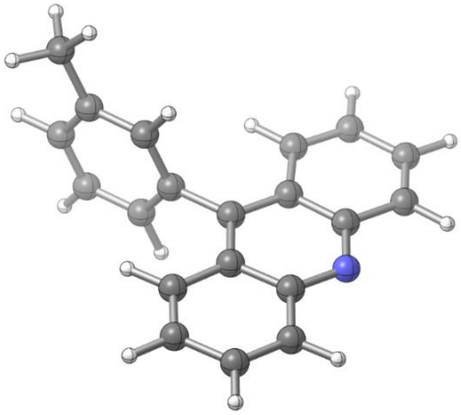

C	-7.8980324880	-1.3640684836	-0.8574059565
C	-6.7670019897	-1.8120660143	-0.2556815119
C	-5.6983969843	-0.9160734265	0.0463423287
C	-5.8409539346	0.4645511584	-0.2962323707
C	-7.0469991657	0.8924546638	-0.9288078383
C	-8.0407145923	0.0080470998	-1.1996567480
C	-4.7883180116	1.3353836274	0.0046313635
C	-3.6445989526	0.8203241965	0.6239571619
C	-3.6059593599	-0.5773532890	0.9223525333
C	-2.4436758179	-1.1124753236	1.5538123017
H	-2.4331439797	-2.1731714620	1.7719642391
C	-1.3933681039	-0.3114431731	1.8656363628
C	-1.4303639880	1.0782307106	1.5696556345
C	-2.5181336342	1.6261228290	0.9699581138
H	-8.7030633721	-2.0530028160	-1.0819649662
H	-6.6408201049	-2.8538606099	0.0117824967
H	-7.1589555688	1.9365625987	-1.1908576311
H	-8.9509584513	0.3442481653	-1.6800172043
H	-0.5159526463	-0.7284160750	2.3446036114
H	-0.5816818088	1.6992917067	1.8273044994
H	-2.5430937207	2.6848390303	0.7466429100
N	-4.6090013048	-1.4092625736	0.6373723494
C	-4.8834071888	2.7822338033	-0.3293077842
C	-4.4339273988	3.2434988575	-1.5634632343
C	-5.4203083786	3.6802900593	0.5838730497
C	-4.5114492710	4.5892226701	-1.9029016521
C	-5.5029146594	5.0267003908	0.2565053373
H	-5.7722068808	3.3269423983	1.5455004067
C	-5.0533152180	5.4760433213	-0.9738397674
H	-5.9217001646	5.7280730242	0.9679134625
H	-4.0161236216	2.5367899785	-2.2721104991
H	-5.1223609094	6.5296856936	-1.2208150345
C	-4.0236087887	5.0832346270	-3.2353390671

H	-4.8247514037	5.5866538770	-3.7811728666
H	-3.2136446767	5.8055388676	-3.1095291615
H	-3.6544920397	4.2635846916	-3.8520196280

HA15

E(UwB97XD) = -826.556742721

Charge = 0 Multiplicity = 2

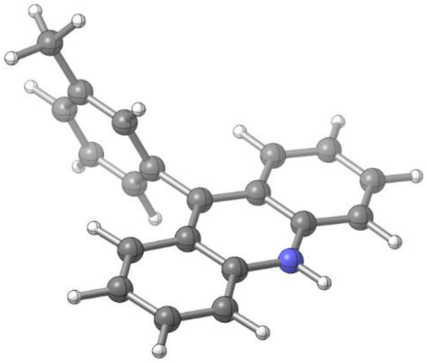

C	-2.7559243577	-3.6179106867	-0.0749672715
C	-3.4024627168	-2.3939935640	-0.0557778693
C	-2.6666138158	-1.2129588887	-0.0300977881
C	-1.2492118510	-1.2358992872	-0.0319762086
C	-0.6280537258	-2.5012533947	-0.0411198102
C	-1.3638756001	-3.6684805915	-0.0638195522
C	-0.5299732122	-0.0036052927	-0.0051859309
C	-1.2446893475	1.2312180378	0.0271005191
C	-2.6621343502	1.2132202405	0.0340555307
C	-3.3936755953	2.3967893048	0.0655942264
H	-4.4767203847	2.3475333718	0.0739557316
C	-2.7428322936	3.6184589048	0.0821260148
C	-1.3507116516	3.6641996487	0.0624194556
C	-0.6191313184	2.4944406954	0.0339650966
H	-3.3366515402	-4.5315155107	-0.0941692411
H	-4.4853627880	-2.3409856533	-0.0574952739
H	0.4527545930	-2.5512464067	-0.0275229810
H	-0.8550095849	-4.6242558254	-0.0706639854
H	-3.3202874900	4.5340258829	0.1058095245
H	-0.8384819835	4.6181917482	0.0670677988
H	0.4617347940	2.5406964152	0.0137412962
N	-3.3079837844	0.0012873155	0.0033946116
C	0.9534568776	-0.0059123383	-0.0096342876
C	1.6639329338	0.3226335648	1.1428709510
C	1.6621348794	-0.3379315533	-1.1620926917
C	3.0551290286	0.3285099266	1.1691870873
C	3.0485948567	-0.3391232797	-1.1509934780
H	1.1236181629	-0.5984504265	-2.0654991618
C	3.7400391101	-0.0094201663	0.0049585001

H	3.5941345396	-0.6000972710	-2.0500120069
H	1.1153591992	0.5786286478	2.0432125010
H	-4.3154246010	0.0030941991	0.0069927397
H	4.8243777450	-0.0162875664	0.0056514695
C	3.7972170284	0.7166972007	2.4171308057
H	4.7552769242	0.1993947325	2.4855797150
H	4.0024672851	1.7908266237	2.4244228204
H	3.2162906052	0.4872443723	3.3113573926

H_2A15

E(RwB97XD) = -827.182688513

Charge = 0 Multiplicity = 1

C	-2.2351047410	-3.6216669974	-0.0737216689
C	-2.7103477480	-2.4343571164	-0.5985135166
C	-2.0148902724	-1.2429001486	-0.3847385004
C	-0.8289425245	-1.2501065993	0.3526924016
C	-0.3792030881	-2.4521683192	0.8843114521
C	-1.0668930539	-3.6371911037	0.6792689715
C	-0.0209495938	0.0214114365	0.5158274040
C	-0.8830090001	1.2516087665	0.3185865409
C	-2.0670348454	1.1731212557	-0.4176853414
C	-2.8130392833	2.3271276160	-0.6642107756
H	-3.7290156194	2.2527853644	-1.2394901739
C	-2.3899063343	3.5476874576	-0.1721295445
C	-1.2241057328	3.6341830069	0.5798961045
C	-0.4860559394	2.4860139579	0.8170863184
H	-2.7842976924	-4.5390659779	-0.2473716650
H	-3.6286523577	-2.4160581168	-1.1748042375
H	0.5402628754	-2.4538338989	1.4598765108
H	-0.6954536582	-4.5636721396	1.0980878458
H	-2.9778714861	4.4354996605	-0.3707626978
H	-0.8933212303	4.5869326973	0.9730800876
H	0.4320284058	2.5428904999	1.3920655286
N	-2.4963314092	-0.0515819973	-0.9054899107
C	1.1796336949	0.0327264502	-0.4222442113
C	2.4691207289	0.0437511906	0.0886833514

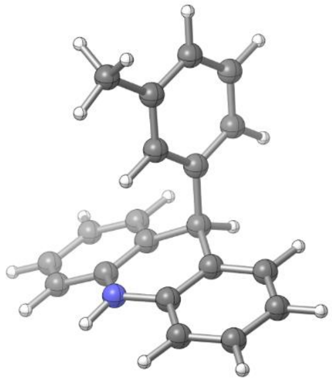

C	1.0026942487	0.0296796424	-1.8053546111
C	3.5618379790	0.0514240042	-0.7712988350
C	2.0818419207	0.0375495618	-2.6776170042
H	-0.0025472815	0.0209162235	-2.2140989565
C	3.3707627507	0.0484027438	-2.1410563238
H	2.6227911213	0.0464383924	1.1618512811
H	0.3752271377	0.0437807748	1.5333129553
H	-3.4142743486	-0.0766870288	-1.3189507237
H	4.2264447887	0.0547068313	-2.8073417375
H	4.5665174251	0.0601489100	-0.3654986366
C	1.8781650994	0.0353191567	-4.1666632661
H	0.8183800208	0.0224887657	-4.4221932916
H	2.3270263157	0.9207643131	-4.6230045283
H	2.3487623345	-0.8383675387	-4.6236756964

HCyA15

E(RwB97XD) = -1061.87720017

Charge = 0 Multiplicity = 1

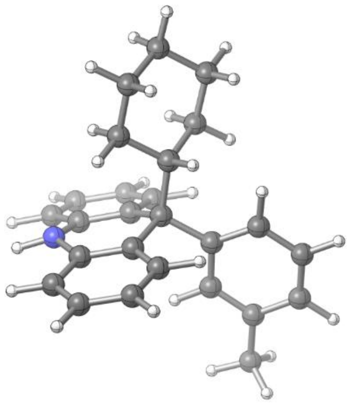

C	-7.5057614090	-1.7389912050	-1.5053579460
C	-6.1700095690	-2.0279071700	-1.3074039150
C	-5.3254902070	-1.0920067950	-0.7055517960
C	-5.8237574610	0.1444473210	-0.2881131830
C	-7.1747324980	0.4052052710	-0.4934287460
C	-8.0170067330	-0.5136618600	-1.0971850870
C	-4.8945727170	1.2164562280	0.2993661110
C	-3.5953177450	0.5757519110	0.7919875290
C	-3.1797740970	-0.6694398930	0.3151187380
C	-1.9511850840	-1.2062547740	0.7106486470
H	-1.6515150520	-2.1747611380	0.3259343040
C	-1.1377159160	-0.5182743410	1.5879461410
C	-1.5470241620	0.7097659160	2.0934053490
C	-2.7644413200	1.2340308420	1.6951798590
H	-8.1492918420	-2.4732865550	-1.9744398140
H	-5.7615830810	-2.9826352910	-1.6193848510
H	-7.5783992780	1.3579977820	-0.1735214970
H	-9.0626865240	-0.2761520900	-1.2455759380

H	-0.1886884730	-0.9461892030	1.8873659330
H	-0.9237250070	1.2537076170	2.7915757770
H	-3.0836859130	2.1872659800	2.0968173520
N	-3.9844018670	-1.3872016150	-0.5498983140
C	-5.6414665490	1.8520446230	1.4878955350
C	-5.7898121910	1.0987425180	2.6546612100
C	-6.2545393880	3.0966930220	1.4396433020
C	-6.5047089380	1.5547665250	3.7514299140
C	-6.9759888410	3.5728101830	2.5297766850
H	-6.1924884400	3.7138766930	0.5546819510
C	-7.0996621000	2.8146052640	3.6780203180
H	-7.6632486970	3.1936875100	4.5234804540
H	-3.6975445730	-2.3286558370	-0.7624742150
C	-4.5811604520	2.2507699800	-0.8389624840
C	-3.6336850470	3.3865487440	-0.4357766000
C	-4.0406041930	1.6020467730	-2.1186398840
H	-5.5467552030	2.6954943830	-1.1057213240
C	-3.5249407550	4.4324857880	-1.5424790490
H	-2.6387513490	2.9718696820	-0.2507035680
H	-3.9528114140	3.8587003880	0.4938809230
C	-3.9030016560	2.6244330820	-3.2448881240
H	-3.0624362450	1.1539802510	-1.9123642300
H	-4.6986618500	0.7972720320	-2.4482568420
C	-3.0290211470	3.8066420000	-2.8414717910
H	-2.8530675210	5.2350325820	-1.2279254860
H	-4.5075003140	4.8888975090	-1.7091618650
H	-3.4957169060	2.1386580860	-4.1351927250
H	-4.9004650180	2.9901193110	-3.5143255580
H	-3.0049495320	4.5516219210	-3.6408954120
H	-1.9993901530	3.4597758990	-2.6982892270
H	-5.3338175960	0.1164380440	2.7069513200
H	-7.4454260570	4.5477236060	2.4719896050
C	-6.6361963710	0.7198601910	4.9943332860
H	-6.1833299920	-0.2630789830	4.8620090460
H	-6.1475774090	1.2054210760	5.8428151190
H	-7.6856571500	0.5787412200	5.2624390660

A16

E(RwB97XD) = -1246.25142432

Charge = 0 Multiplicity = 1

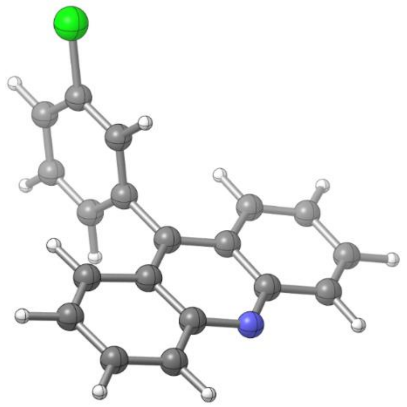

C	-7.9010522831	-1.3592726731	-0.8516986471
C	-6.7708119056	-1.8077820109	-0.2491120829
C	-5.7001063746	-0.9130786714	0.0488624369
C	-5.8401533847	0.4668336618	-0.2980916836
C	-7.0451634380	0.8954460359	-0.9321075140
C	-8.0405700644	0.0119278000	-1.1990947259
C	-4.7843289448	1.3336497518	0.0000735672
C	-3.6404491117	0.8204215550	0.6191248144
C	-3.6051614687	-0.5767076064	0.9211933821
C	-2.4435253812	-1.1133176362	1.5523938908
H	-2.4354228956	-2.1735167398	1.7728108724
C	-1.3910325419	-0.3140853109	1.8609554222
C	-1.4252966940	1.0750786915	1.5620535669
C	-2.5120425286	1.6247037092	0.9623227325
H	-8.7077929490	-2.0469796668	-1.0736112615
H	-6.6465375752	-2.8488757249	0.0217661590
H	-7.1558797503	1.9383770228	-1.1994614989
H	-8.9499233288	0.3481960997	-1.6810253985
H	-0.5138787258	-0.7318351661	2.3396030071
H	-0.5748951448	1.6946543777	1.8174745815
H	-2.5331031912	2.6832009810	0.7374591476
N	-4.6109760663	-1.4061665819	0.6399412082
C	-4.8782252089	2.7806017398	-0.3349117919
C	-4.4088743780	3.2380713303	-1.5608867020
C	-5.4331414140	3.6788397986	0.5697933292
C	-4.5034964425	4.5862773036	-1.8599511413
C	-5.5182197635	5.0251047172	0.2509801135
H	-5.7985516311	3.3207989903	1.5241026393
C	-5.0535468477	5.4918691169	-0.9689702606
H	-5.9505727467	5.7217273198	0.9578629799
H	-3.9753297791	2.5470767466	-2.2721441003
H	-5.1179942354	6.5413863446	-1.2227885916

Cl	-3.9133159450	5.1541319245	-3.3991037708

HA16

E(UwB97XD) = -1246.84913946

Charge = 0 Multiplicity = 2

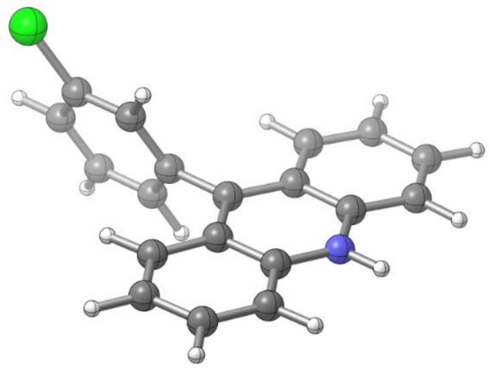

C	-2.7540831763	-3.6175146434	-0.0865673713
C	-3.4010408175	-2.3944394698	-0.0674839409
C	-2.6656993977	-1.2129296751	-0.0359151807
C	-1.2485436753	-1.2354261152	-0.0328599267
C	-0.6267646036	-2.5004731189	-0.0403786747
C	-1.3619016049	-3.6676060249	-0.0685977065
C	-0.5321648520	-0.0013949161	-0.0008818734
C	-1.2469554390	1.2335745164	0.0329293565
C	-2.6641495506	1.2127668177	0.0360718966
C	-3.3981279628	2.3950613173	0.0691839400
H	-4.4810305336	2.3435033109	0.0752417525
C	-2.7498014841	3.6173727436	0.0900920536
C	-1.3575792718	3.6658766538	0.0725047206
C	-0.6237601309	2.4979678852	0.0425898803
H	-3.3341719618	-4.5313787148	-0.1101143934
H	-4.4838867405	-2.3416714345	-0.0733502370
H	0.4538131223	-2.5520959840	-0.0199161654
H	-0.8526175780	-4.6231018359	-0.0738254541
H	-3.3288385898	4.5318581936	0.1152054676
H	-0.8471138478	4.6207197100	0.0799051263
H	0.4568607285	2.5488987195	0.0229909867
N	-3.3081304057	0.0003314873	-0.0007386463
C	0.9502444386	-0.0027809037	-0.0031576902
C	1.6530622966	0.3599160291	1.1440822794
C	1.6642826558	-0.3631216260	-1.1443281111
C	3.0366653048	0.3580233365	1.1291219795
C	3.0501624242	-0.3605632182	-1.1404392645
H	1.1261626014	-0.6458283333	-2.0406660694
C	3.7523705404	0.0015390181	-0.0008408770
H	3.5941509228	-0.6398890330	-2.0340053579
H	1.1178739688	0.6416380006	2.0416922613

H	-4.3156487134	0.0009763760	0.0001422468
H	4.8340651513	0.0069244893	0.0085038318
Cl	3.8996232117	0.8160034520	2.5757872011

H_2A16

E(RwB97XD) = -1247.47523953

Charge = 0 Multiplicity = 1

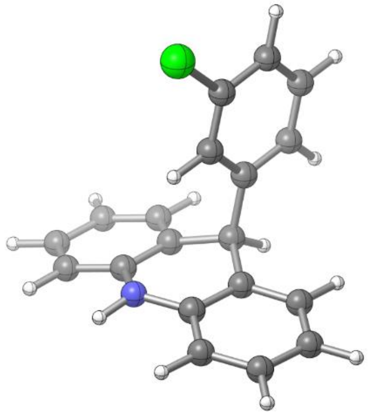

C	-2.2660602098	-3.6017727155	-0.0709519623
C	-2.7275313550	-2.4114990388	-0.6009160436
C	-2.0214373069	-1.2264192096	-0.3870024911
C	-0.8392337231	-1.2442808045	0.3560412424
C	-0.4031002173	-2.4489045736	0.8932433540
C	-1.1016400493	-3.6272576184	0.6878176847
C	-0.0204241240	0.0201928812	0.5180095747
C	-0.8710159235	1.2581399977	0.3208925693
C	-2.0520889071	1.1894764224	-0.4209264233
C	-2.7879410375	2.3497577106	-0.6683422524
H	-3.7018475714	2.2839362880	-1.2478551280
C	-2.3572127481	3.5656702466	-0.1717227446
C	-1.1943052188	3.6419435070	0.5860689698
C	-0.4660758594	2.4879860228	0.8243061212
H	-2.8232676016	-4.5141963419	-0.2451011002
H	-3.6428145246	-2.3857327253	-1.1815714901
H	0.5133748977	-2.4580010392	1.4734254026
H	-0.7415681503	-4.5564203680	1.1104819490
H	-2.9372046157	4.4584762116	-0.3712755434
H	-0.8582876089	4.5913919996	0.9826553625
H	0.4494626973	2.5363709367	1.4040494453
N	-2.4878775336	-0.0313334579	-0.9124519521
C	1.1770909794	0.0203194831	-0.4237696435
C	2.4709350021	0.0222339514	0.0805621446
C	0.9888807719	0.0160663034	-1.8038687010
C	3.5641986219	0.0197736648	-0.7754037293
C	2.0873993740	0.0137372498	-2.6411628398
H	-0.0093256188	0.0144600836	-2.2237385943
C	3.3831631094	0.0155082366	-2.1480512532

H	2.6260513504	0.0257637439	1.1529568634
H	0.3810503244	0.0394596384	1.5331489554
H	-3.4020621278	-0.0485907581	-1.3346344635
H	4.2287365099	0.0138048069	-2.8225847676
H	4.5685900822	0.0214405328	-0.3707347353
Cl	1.8356002717	0.0086418519	-4.3688507303

HCyA16

E(RwB97XD) = -1482.17018021

Charge = 0 Multiplicity = 1

C	-7.8783624130	-1.4727231180	-1.5847168440
C	-6.9102236700	-1.9912162950	-0.7478235620
C	-5.9417059340	-1.1570644570	-0.1844043620
C	-5.9352407540	0.2104191910	-0.4692355520
C	-6.9188565790	0.7043980310	-1.3200562120
C	-7.8876267780	-0.1144877600	-1.8757428810
C	-4.9295627320	1.1542152180	0.2055317650
C	-3.7410067490	0.3599348230	0.7505621160
C	-3.8476538420	-1.0090313840	1.0058151220
C	-2.7848594100	-1.7061454670	1.5874622570
H	-2.8939379220	-2.7679262090	1.7782214870
C	-1.6105461300	-1.0535726010	1.9030945630
C	-1.4762702310	0.3022410630	1.6293812670
C	-2.5349450950	0.9852772370	1.0566418700
H	-8.6242362740	-2.1307461610	-2.0136174880
H	-6.8919832270	-3.0502133160	-0.5156641370
H	-6.9302924120	1.7626632250	-1.5506032680
H	-8.6412535020	0.3034967820	-2.5307803120
H	-0.7943235070	-1.6058281360	2.3526609280
H	-0.5559273640	0.8228153510	1.8610345000
H	-2.4243945960	2.0403667570	0.8407894030
N	-5.0049577210	-1.6894887990	0.6798125100
C	-4.4397639320	2.1297330780	-0.8810159730
C	-3.5734538080	1.6561779100	-1.8670606160
C	-4.8849503810	3.4419073290	-0.9816975820
C	-3.1534342090	2.4660735620	-2.9056858240

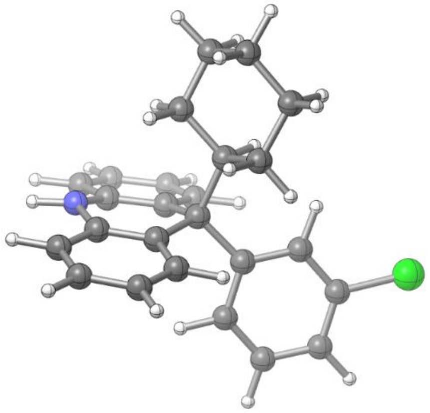

C	-4.4544967900	4.2438333490	-2.0280657610
H	-5.5741573450	3.8590254290	-0.2627452770
C	-3.5882394430	3.7813006450	-2.9978764930
H	-3.2632691740	4.4242259290	-3.8046674300
H	-4.9888415200	-2.6912086400	0.7776332540
C	-5.6708608360	1.8823526580	1.3815003950
C	-6.3455400370	0.9156128040	2.3616755260
C	-4.8035573340	2.8587352590	2.1846940140
H	-6.4800243600	2.4538627360	0.9122484200
C	-7.1821667200	1.6669062780	3.3952967250
H	-5.5776998820	0.3253623340	2.8735045200
H	-6.9870763270	0.2137938920	1.8276508260
C	-5.6438039480	3.6423088670	3.1898417560
H	-4.0447591120	2.2929951280	2.7324062760
H	-4.2666721410	3.5470919220	1.5308743780
C	-6.3605955030	2.7032231110	4.1537209140
H	-7.6308269720	0.9549388390	4.0925417680
H	-8.0099067530	2.1696880000	2.8822687790
H	-5.0062099460	4.3373568730	3.7417594960
H	-6.3826460380	4.2480838880	2.6526807070
H	-6.9999735110	3.2697345220	4.8354738210
H	-5.6142218870	2.1893646790	4.7699726420
H	-3.2246681030	0.6323351480	-1.8216011920
Cl	-5.0350129060	5.8894380530	-2.1142832080
H	-2.4800807740	2.0735057160	-3.6573503700

A17

E(RwB97XD) = -885.894390043

Charge = 0 Multiplicity = 1

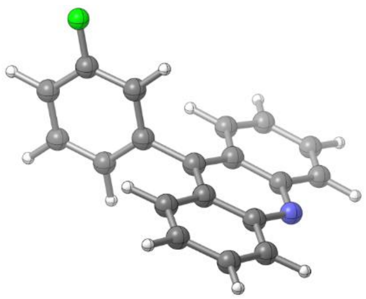

C	-7.9003672382	-1.3595692503	-0.8532186554
C	-6.7702877512	-1.8082867383	-0.2504789251
C	-5.6996403009	-0.9136959844	0.0481710519
C	-5.8394279583	0.4662425918	-0.2986490192
C	-7.0443771823	0.8951004429	-0.9326208157
C	-8.0398259659	0.0117805640	-1.2000953393
C	-4.7837654651	1.3332379950	-0.0001538713

C	-3.6405749339	0.8197149875	0.6200530804
C	-3.6054189607	-0.5774047328	0.9220012900
C	-2.4442373240	-1.1139523842	1.5541491841
H	-2.4361936526	-2.1741827825	1.7744482000
C	-1.3921543823	-0.3146248433	1.8638762861
C	-1.4263528727	1.0746122564	1.5652498577
C	-2.5126349947	1.6240904517	0.9645496924
H	-8.7070840485	-2.0471974895	-1.0754969770
H	-6.6461796421	-2.8494562148	0.0202062594
H	-7.1549215040	1.9382719902	-1.1991046941
H	-8.9492190900	0.3483048135	-1.6817806992
H	-0.5154112016	-0.7323550534	2.3433092540
H	-0.5763666808	1.6943049519	1.8217832468
H	-2.5340778627	2.6826441247	0.7399766176
N	-4.6108670030	-1.4070192271	0.6397700473
C	-4.8769877457	2.7801596445	-0.3360944155
C	-4.4104663776	3.2367593796	-1.5637515870
C	-5.4291317117	3.6789654591	0.5707848815
C	-4.5109848947	4.5830355038	-1.8486029073
C	-5.5149824082	5.0263862391	0.2534374322
H	-5.7918790472	3.3204815436	1.5257940593
C	-5.0539944346	5.4954637602	-0.9675769906
H	-5.9454220470	5.7210670382	0.9633926618
H	-3.9763347839	2.5592469493	-2.2878460548
H	-5.1116455381	6.5421649068	-1.2355493014
F	-4.0587899759	5.0213762565	-3.0382623895

HA17

E(UwB97XD) = -886.491934946

Charge = 0 Multiplicity = 2

C	-2.7529267698	-3.6176614231	-0.0807875674
C	-3.4007220349	-2.3948878229	-0.0631976765
C	-2.6661540930	-1.2129116775	-0.0334633240
C	-1.2489400953	-1.2346000327	-0.0302742356
C	-0.6262707846	-2.4992105101	-0.0369351115
C	-1.3607190037	-3.6668711127	-0.0634507326

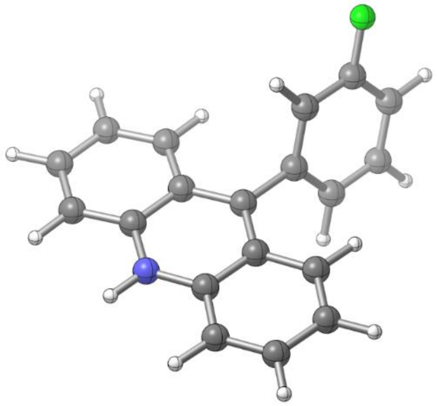

C	-0.5331064604	-0.0004242720	0.0004211100
C	-1.2484047384	1.2341362880	0.0312971131
C	-2.6656263517	1.2130074793	0.0335868983
C	-3.3997872897	2.3952250250	0.0634085784
H	-4.4826991953	2.3435900335	0.0686555853
C	-2.7515936906	3.6177649936	0.0820586783
C	-1.3593839384	3.6664742136	0.0657441510
C	-0.6253296232	2.4985589486	0.0390686018
H	-3.3324760535	-4.5319082490	-0.1029924839
H	-4.4836135225	-2.3428820184	-0.0690500669
H	0.4544203421	-2.5494591385	-0.0178483469
H	-0.8508766798	-4.6220832680	-0.0681284056
H	-3.3308202927	4.5322053811	0.1045605267
H	-0.8491431843	4.6214604450	0.0715662957
H	0.4553515243	2.5489747128	0.0206682662
N	-3.3091598191	0.0001886684	-0.0004518851
C	0.9497634845	-0.0013539578	-0.0008575515
C	1.6527886179	0.3485659372	1.1502816145
C	1.6629848868	-0.3493247448	-1.1472771569
C	3.0317263672	0.3410841025	1.1210318811
C	3.0495771156	-0.3476716593	-1.1458238154
H	1.1234972282	-0.6220576503	-2.0457263477
C	3.7551374794	0.0004708061	-0.0031688213
H	3.5904199827	-0.6176117586	-2.0441610491
H	1.1340991366	0.6234465590	2.0599911620
H	-4.3166676262	0.0004233264	-0.0003472729
H	4.8368867433	0.0093528496	0.0208807038
F	3.6990793686	0.6801217761	2.2422071546

H_2A17

E(RwB97XD) = -887.118133871

Charge = 0 Multiplicity = 1

C	-2.2675474782	-3.6019784554	-0.0704298712
C	-2.7304791693	-2.4113704167	-0.5984209380
C	-2.0240278743	-1.2262898285	-0.3855182355
C	-0.8400376884	-1.2443509036	0.3546757056

C	-0.4024418508	-2.4493335269	0.8898760717
C	-1.1011984456	-3.6277470022	0.6853331092
C	-0.0210387499	0.0200795438	0.5165357525
C	-0.8717112190	1.2581388217	0.3199931580
C	-2.0545710582	1.1896994905	-0.4189963374
C	-2.7907128949	2.3501456162	-0.6649081215
H	-3.7061433409	2.2843787138	-1.2420397367
C	-2.3584672176	3.5661383405	-0.1697489864
C	-1.1936339578	3.6422680672	0.5850612627
C	-0.4652497491	2.4880866579	0.8219200666
H	-2.8251044492	-4.5143555625	-0.2437267711
H	-3.6472701136	-2.3853479487	-1.1767016680
H	0.5154404292	-2.4585950053	1.4678304579
H	-0.7398884616	-4.5571087414	1.1065161442
H	-2.9387698693	4.4590254034	-0.3680543511
H	-0.8563403657	4.5917114905	0.9805907793
H	0.4516894947	2.5363308970	1.3994605899
N	-2.4922896106	-0.0310414746	-0.9089991930
C	1.1761903076	0.0203903459	-0.4252524028
C	2.4701936750	0.0233506293	0.0811914686
C	0.9888907829	0.0153191587	-1.8056996392
C	3.5659739745	0.0211413511	-0.7726894704
C	2.0935095480	0.0132745952	-2.6275275846
H	-0.0020477855	0.0128538792	-2.2433883077
C	3.3888325846	0.0160676448	-2.1463915200
H	2.6232984909	0.0274922197	1.1537245236
H	0.3801913375	0.0391495860	1.5317528831
H	-3.4080695831	-0.0481486685	-1.3276986046
H	4.2255244613	0.0145030405	-2.8324243341
F	1.9019375802	0.0084709062	-3.9621348894
H	4.5688171159	0.0236423448	-0.3642240804

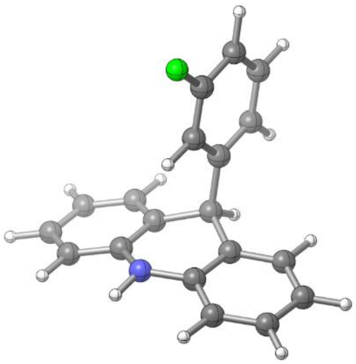

HCyA17

E(RwB97XD) = -1121.81287881

Charge = 0 Multiplicity = 1

C	-7.8184469890	-1.9207219370	-1.1210941870
C	-6.5118690880	-2.3020996360	-0.8934125570

C	-5.5754039850	-1.3755567750	-0.4260884840
C	-5.9461055250	-0.0490041910	-0.1950840830
C	-7.2770303280	0.2997287760	-0.4103477770
C	-8.2113591710	-0.6113655670	-0.8712342920
C	-4.9107946300	0.9956076150	0.2264909850
C	-3.6811886260	0.3079473260	0.8375173040
C	-3.4094229190	-1.0383083940	0.5823150000
C	-2.2463972500	-1.6309918470	1.0796641060
H	-2.0594906330	-2.6786568220	0.8720310830
C	-1.3470405960	-0.8914087250	1.8217498510
C	-1.6018639660	0.4486561800	2.0837002430
C	-2.7611227930	1.0267582270	1.5939806680
H	-8.5344033400	-2.6487867480	-1.4825671580
H	-6.1987652680	-3.3260296580	-1.0642776940
H	-7.5885241060	1.3172866470	-0.2115784330
H	-9.2360077830	-0.3028910730	-1.0345039040
H	-0.4484187800	-1.3631277490	2.2002201270
H	-0.9050402290	1.0385311270	2.6652894820
H	-2.9545960090	2.0719367260	1.8021285790
N	-4.2781294380	-1.7866982300	-0.1880083020
C	-5.5059638680	1.8963649350	1.3248445890
C	-6.1736364680	1.2979062950	2.3951725600
C	-5.3370836750	3.2754951400	1.3522729850
C	-6.6675115130	2.0478126570	3.4471235110
C	-5.8419089970	4.0020532640	2.4139976260
H	-4.8135891810	3.8114164600	0.5737892280
C	-6.5104011370	3.4277924260	3.4701270110
H	-6.8892194770	4.0375084600	4.2795994310
H	-4.0904629500	-2.7730944050	-0.2616601130
C	-4.4312875040	1.8099251680	-1.0263923100
C	-3.6377491630	0.9664102890	-2.0309752940
C	-5.5443966180	2.5431135740	-1.7839577160
H	-3.7328091380	2.5609764520	-0.6397945060
C	-3.0593856490	1.8320041570	-3.1485372450
H	-4.2959474130	0.2052655780	-2.4638900600
H	-2.8236565810	0.4403094380	-1.5312628350

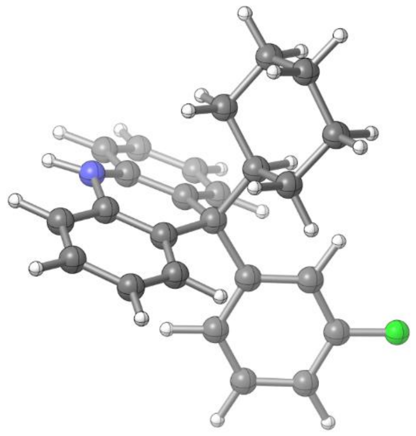

C	-4.9684227350	3.4415091410	-2.8755557700
H	-6.2018891850	1.8052599180	-2.2521755530
H	-6.1675585480	3.1306678270	-1.1089738840
C	-4.1377813870	2.6347403740	-3.8674702210
H	-2.5189425030	1.2017429450	-3.8592323040
H	-2.3249146600	2.5218909640	-2.7175363840
H	-5.7795150950	3.9593478500	-3.3934778510
H	-4.3406852250	4.2144386570	-2.4173511100
H	-3.6868272860	3.2934367940	-4.6139993030
H	-4.7982576120	1.9467129320	-4.4071799890
H	-6.3056216460	0.2236264860	2.4057472640
F	-5.6628844830	5.3399187340	2.4062699530
H	-7.1810775730	1.5558493780	4.2636195130

A18

E(RwB97XD) = -1123.74416498

Charge = 0 Multiplicity = 1

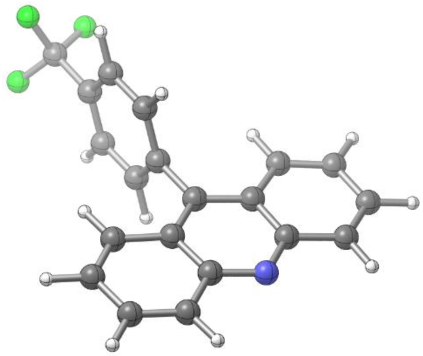

C	-7.8991952260	-1.3529393900	-0.8569489060
C	-6.7697099710	-1.8030172240	-0.2541049150
C	-5.6988488430	-0.9093666480	0.0463562620
C	-5.8378548760	0.4710831000	-0.2987264350
C	-7.0421046620	0.9014488200	-0.9330730280
C	-8.0377612090	0.0188800480	-1.2022072920
C	-4.7816748230	1.3365797050	0.0018572030
C	-3.6390074000	0.8224283900	0.6221214150
C	-3.6049480030	-0.5751911960	0.9221729410
C	-2.4446600590	-1.1131766610	1.5546330070
H	-2.4374233700	-2.1736802580	1.7735762730
C	-1.3924809770	-0.3147923040	1.8664026720
C	-1.4256412690	1.0748538530	1.5696204710
C	-2.5109825100	1.6258622980	0.9686326070
H	-8.7061646440	-2.0398096470	-1.0805594500
H	-6.6462981690	-2.8445767000	0.0153385610
H	-7.1526814740	1.9448476590	-1.1987788170
H	-8.9466697120	0.3564737030	-1.6840341110
H	-0.5164478990	-0.7335703860	2.3461797290

H	-0.5755916610	1.6937281230	1.8278634330
H	-2.5309221110	2.6848448270	0.7458028100
N	-4.6106536140	-1.4037512450	0.6380435060
C	-4.8739551370	2.7827846700	-0.3343468000
C	-4.4075197340	3.2495371810	-1.5587539860
C	-5.4311384340	3.6789559400	0.5707149370
C	-4.4996389270	4.5931877420	-1.8772521610
C	-5.5254047800	5.0245170600	0.2577711860
H	-5.7962808720	3.3214702480	1.5252617160
C	-5.0633564880	5.4787262950	-0.9689481300
H	-4.1354321940	4.9461893370	-2.8331008560
H	-5.9615159670	5.7132342940	0.9689871220
H	-3.9726400880	2.5562745790	-2.2677062530
C	-5.1247000410	6.9429048720	-1.3007243260
F	-3.9816062300	7.5783485530	-0.9743555170
F	-6.1103233040	7.5764026530	-0.6495878650
F	-5.3183998830	7.1621645780	-2.6100523150

HA18

E(UwB97XD) = -1124.34208533

Charge = 0 Multiplicity = 2

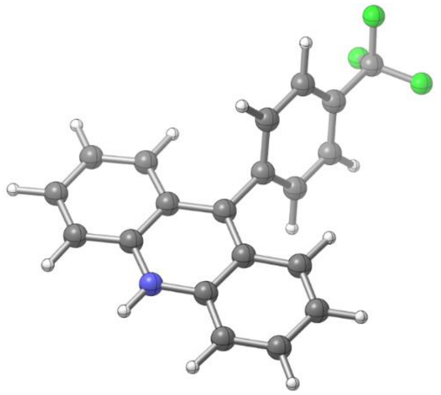

C	-7.9956726009	-1.3304078492	-0.7757142846
C	-6.8282030066	-1.7873997875	-0.1905084199
C	-5.7783445001	-0.9084690707	0.0601050971
C	-5.8843936731	0.4663709622	-0.2677945433
C	-7.0838218994	0.8906222489	-0.8749984449
C	-8.1194254386	0.0133433632	-1.1220256218
C	-4.7842765010	1.3342482153	0.0045077318
C	-3.5952388999	0.8093830496	0.5946606177
C	-3.5276643236	-0.5725360873	0.9022659902
C	-2.3865689767	-1.1228230492	1.4791692891
H	-2.3661464227	-2.1845271523	1.6975878596
C	-1.2971943160	-0.3213762191	1.7712259172
C	-1.3437905281	1.0428746928	1.4927614555
C	-2.4705022369	1.5948424971	0.9191716200
H	-8.8066434139	-2.0217437872	-0.9669024958

H	-6.7162827217	-2.8321672537	0.0758385772
H	-7.1894928129	1.9299683871	-1.1564161183
H	-9.0276378772	0.3724673451	-1.5894923673
H	-0.4142268218	-0.7582858672	2.2202644188
H	-0.4968091459	1.6746459566	1.7290998628
H	-2.4966845583	2.6570901207	0.7150932449
N	-4.6143419372	-1.3661098199	0.6272929704
C	-4.8761634156	2.7758587567	-0.3248021188
C	-4.1236436068	3.3177625187	-1.3678059614
C	-5.7150170079	3.6192734272	0.3988121928
C	-4.2077999655	4.6619271199	-1.6782492320
C	-5.8046941566	4.9685611611	0.0952418692
H	-6.3050273916	3.2150756741	1.2119808214
C	-5.0497711129	5.4888248744	-0.9437242003
H	-3.6208086945	5.0668149149	-2.4934859417
H	-6.4598083340	5.6071351752	0.6720086805
H	-3.4674962506	2.6752211691	-1.9416883450
C	-5.1242887992	6.9436884051	-1.3058882082
H	-4.5525306623	-2.3463712789	0.8533172685
F	-5.9312126572	7.6430358010	-0.4984441468
F	-3.9169719238	7.5375848932	-1.2562322956
F	-5.5787650092	7.1269995134	-2.5603588590

H_2A18

E(RwB97XD) = -1124.96818226

Charge = 0 Multiplicity = 1

C	-7.7831851752	-1.1897011373	-1.2130402745
C	-6.4136739660	-1.3769319024	-1.2274016641
C	-5.5794942062	-0.5142601848	-0.5141294064
C	-6.1283314655	0.5475860413	0.2078559845
C	-7.5082322060	0.7069417959	0.2214521263
C	-8.3414491663	-0.1484205736	-0.4807504222
C	-5.2245783123	1.5374707132	0.9138439163
C	-3.8685557394	0.9306339621	1.2126981173
C	-3.3973981814	-0.1429999997	0.4541815815
C	-2.1149282264	-0.6458536719	0.6801958392

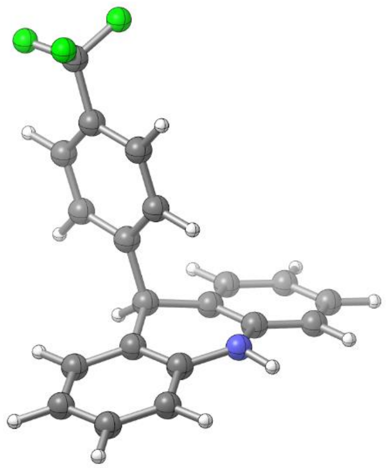

H	-1.7596604650	-1.4789153171	0.0842366939
C	-1.3133499239	-0.0918697186	1.6607140341
C	-1.7808863297	0.9627339140	2.4361416460
C	-3.0524237453	1.4609219908	2.2038850056
H	-8.4193860547	-1.8661558744	-1.7706690680
H	-5.9759763633	-2.1972670043	-1.7848955245
H	-7.9317116660	1.5301785581	0.7867202760
H	-9.4139449079	-0.0035121541	-0.4616328148
H	-0.3204417406	-0.4918759304	1.8261506414
H	-1.1588004552	1.3933225950	3.2102517493
H	-3.4233530306	2.2912833791	2.7950123996
N	-4.2063650357	-0.7056064459	-0.5212599586
C	-5.0784218787	2.8204394613	0.1070993621
C	-4.5527323660	2.7978081024	-1.1848243253
C	-5.4632549216	4.0381790135	0.6498070275
C	-4.4179237606	3.9632859486	-1.9115776835
C	-5.3316377651	5.2161267146	-0.0727732456
H	-5.8728572381	4.0724431516	1.6522982736
C	-4.8078448684	5.1771097241	-1.3530492896
H	-4.0096446147	3.9315908721	-2.9141416952
H	-5.6384233299	6.1551550851	0.3677400671
H	-4.2444592362	1.8586425229	-1.6282635131
C	-4.6725437573	6.4252079812	-2.1745365924
H	-5.6925212557	1.8117445401	1.8614762409
H	-3.8692119284	-1.5492036508	-0.9561864740
F	-4.9193635927	7.5405881480	-1.4756266345
F	-5.5204840649	6.4318591576	-3.2223398371
F	-3.4382826590	6.5579057928	-2.6937581188

HCyA18

E(RwB97XD) = -1359.66307374

Charge = 0 Multiplicity = 1

C	-7.6797900188	-1.1922623636	-1.6470026956
C	-6.3061648605	-1.2845193118	-1.7427061965
C	-5.4856354992	-0.5497398156	-0.8821315760
C	-6.0451105266	0.2988917409	0.0757783568

C	-7.4339194486	0.3518837950	0.1646904814
C	-8.2537855534	-0.3747584230	-0.6809671489
C	-5.1608516380	1.1795694459	0.9607592956
C	-3.7321369695	0.6194728194	1.0099804054
C	-3.2689617640	-0.2567525720	0.0256532719
C	-1.9423750819	-0.6939320567	0.0376745233
H	-1.6064616472	-1.3756975943	-0.7355023641
C	-1.0717644627	-0.2603881842	1.0176767286
C	-1.5166721856	0.6111149928	2.0036110699
C	-2.8349693783	1.0350007889	1.9887036408
H	-8.3038933900	-1.7687235581	-2.3189428535
H	-5.8478357939	-1.9349566120	-2.4790427637
H	-7.8857090415	0.9836869825	0.9188946922
H	-9.3298269614	-0.3035692151	-0.5876626696
H	-0.0455005500	-0.6067588951	1.0141885780
H	-0.8437880910	0.9572103262	2.7776576904
H	-3.1763378575	1.7149859194	2.7597042740
N	-4.1136341657	-0.6757210485	-0.9836324673
C	-5.6938999961	1.1560225254	2.4046125975
C	-6.0258989014	-0.0766113942	2.9726826811
C	-5.7855102657	2.2830251239	3.2106418580
C	-6.4465275737	-0.1784626634	4.2818727829
C	-6.2090660019	2.1949230491	4.5306475045
H	-5.5195926099	3.2584947375	2.8304433425
C	-6.5423415714	0.9657768455	5.0674291993
H	-6.6974525859	-1.1479135962	4.6944216998
H	-6.2717117207	3.0925958568	5.1307442645
H	-5.9492240066	-0.9768222574	2.3763338383
C	-7.0221397637	0.8370244480	6.4826442572
H	-3.7636053525	-1.3825552990	-1.6093164707
C	-5.0858017769	2.6315426084	0.3701210924
C	-4.3596138627	2.6994924897	-0.9782524448
C	-6.4378181465	3.3400906043	0.2282046997
H	-4.4747401711	3.2060515235	1.0759006786
C	-4.1745857937	4.1445179003	-1.4372336125
H	-4.9381337046	2.1501960154	-1.7289761742

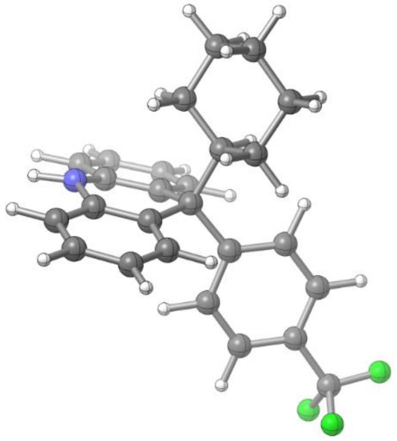

H	-3.3823074507	2.2200859314	-0.9119315322
C	-6.2553695368	4.7968056831	-0.1904091277
H	-7.0282774753	2.8298454476	-0.5378355905
H	-7.0150345784	3.2879082611	1.1520787072
C	-5.4987010189	4.8966463206	-1.5102725504
H	-3.6756531150	4.1601500650	-2.4094283446
H	-3.5085306266	4.6564760453	-0.7333547545
H	-7.2311822537	5.2813381295	-0.2756373281
H	-5.7020990310	5.3331002433	0.5891705228
H	-5.3268270508	5.9430499886	-1.7748516614
H	-6.1145943623	4.4636451242	-2.3065337758
F	-6.9354760541	1.9830326815	7.1702209516
F	-6.3225167356	-0.0828291897	7.1722774872
F	-8.3110630233	0.4477005877	6.5438389283

A19

E(RwB97XD) = -1123.74410704

Charge = 0 Multiplicity = 1

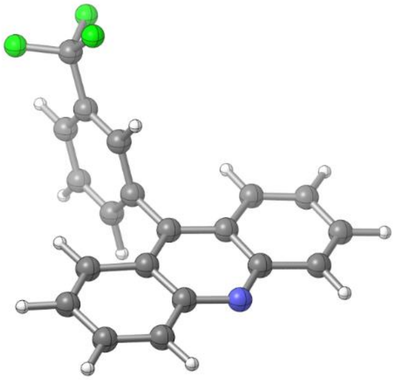

C	-7.9046750731	-1.3659389688	-0.8431113320
C	-6.7742188456	-1.8128008860	-0.2397103843
C	-5.7032638054	-0.9173730579	0.0550442505
C	-5.8432831274	0.4615650380	-0.2959201678
C	-7.0486513822	0.8884814336	-0.9304388262
C	-8.0442343156	0.0042516310	-1.1943959895
C	-4.7870322193	1.3288975116	-0.0010436977
C	-3.6427542562	0.8173955466	0.6185886009
C	-3.6076246091	-0.5788136912	0.9250201632
C	-2.4456692517	-1.1138109213	1.5569352544
H	-2.4378022277	-2.1732726229	1.7808432302
C	-1.3924817583	-0.3140299582	1.8616921030
C	-1.4263097181	1.0740744193	1.5579167425
C	-2.5134599243	1.6222415145	0.9575787514
H	-8.7115715889	-2.0541977725	-1.0626853730
H	-6.6499248420	-2.8531047547	0.0341510370
H	-7.1597865213	1.9306015842	-1.2007932428
H	-8.9537901661	0.3391920137	-1.6768572849

H	-0.5150199200	-0.7305411641	2.3408337852
H	-0.5752029774	1.6940202881	1.8100471516
H	-2.5338130209	2.6799730991	0.7290487288
N	-4.6139059298	-1.4087715299	0.6470477749
C	-4.8802765039	2.7744284921	-0.3413265204
C	-4.4195737344	3.2309434685	-1.5713881283
C	-5.4266836799	3.6769868139	0.5621167405
C	-4.5102267368	4.5772031653	-1.8834007324
C	-5.5137804336	5.0247023334	0.2434500860
H	-5.7861619088	3.3219763693	1.5201754226
C	-5.0564134013	5.4805243527	-0.9796849413
H	-5.9406975353	5.7210338124	0.9534746522
H	-3.9935798517	2.5298132866	-2.2765947643
H	-5.1233893911	6.5315257321	-1.2316882067
C	-4.0177982407	5.0956551420	-3.2054070473
F	-4.9957038613	5.7073736709	-3.8984221080
F	-3.0398635244	6.0071601227	-3.0514832318
F	-3.5283184664	4.1330117153	-3.9948859361

HA19

E(UwB97XD) = -1124.34195793

Charge = 0 Multiplicity = 2

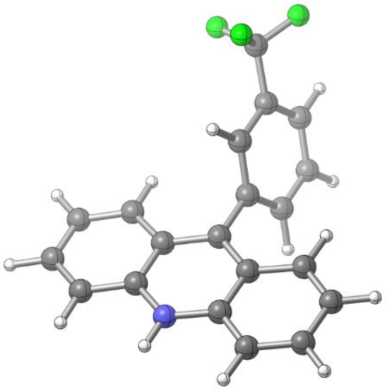

C	-2.7736719390	-3.6189814720	-0.1243127590
C	-3.4132703840	-2.3923702700	-0.0962912310
C	-2.6710429520	-1.2155847630	-0.0507893250
C	-1.2540480640	-1.2461912300	-0.0432107490
C	-0.6400663050	-2.5150248170	-0.0590417030
C	-1.3819079670	-3.6774300290	-0.1008538790
C	-0.5307129780	-0.0163511200	0.0031244850
C	-1.2391941690	1.2223602410	0.0477302560
C	-2.6564270140	1.2089730170	0.0470535860
C	-3.3845522440	2.3945612380	0.0914529140
H	-4.4676808230	2.3482214050	0.0948116530
C	-2.7303842060	3.6132604360	0.1267497590
C	-1.3379199460	3.6548767150	0.1119952640
C	-0.6100015780	2.4836838140	0.0711679710

H	-3.3589940050	-4.5291540980	-0.1584565780
H	-4.4957205940	-2.3328661530	-0.1054598940
H	0.4399389950	-2.5744607070	-0.0330667000
H	-0.8782090450	-4.6358173210	-0.1121323800
H	-3.3048938020	4.5302999810	0.1606118860
H	-0.8225311460	4.6069127660	0.1300699710
H	0.4707878780	2.5306072880	0.0531648100
N	-3.3068932570	0.0006769990	-0.0052938530
C	0.9510382590	-0.0244842240	0.0059351630
C	1.6569808050	0.3504448430	1.1458014370
C	1.6681545290	-0.4040548750	-1.1270415770
C	3.0436463150	0.3430905580	1.1463535010
C	3.0542718260	-0.4078131870	-1.1240237230
H	1.1303952490	-0.6981592700	-2.0203036470
C	3.7510060820	-0.0319937030	0.0122871660
H	3.5956289630	-0.7031039590	-2.0137238200
H	1.1131421770	0.6460244640	2.0337933410
H	-4.3144071480	0.0070229010	-0.0076328760
H	4.8330960720	-0.0330694770	0.0148161670
C	3.7894932840	0.7986531930	2.3685374460
F	3.0961608830	0.6051452290	3.4995426990
F	4.0740570910	2.1159725800	2.3175541450
F	4.9627185390	0.1649285850	2.5183809150

H_2A19

E(RwB97XD) = -1124.96813164

Charge = 0 Multiplicity = 1

C	-2.1309464330	-3.6134576310	-0.0819472490
C	-2.6088518720	-2.4122509230	-0.5711262330
C	-1.8963721160	-1.2314810490	-0.3539404900
C	-0.6909947970	-1.2650089110	0.3501860500
C	-0.2377640670	-2.4809124090	0.8464594390
C	-0.9426136800	-3.6549025280	0.6381048430
C	0.1322352660	-0.0042208760	0.5172111350
C	-0.7214702570	1.2383546980	0.3669299450
C	-1.9259688420	1.1847772610	-0.3373871140

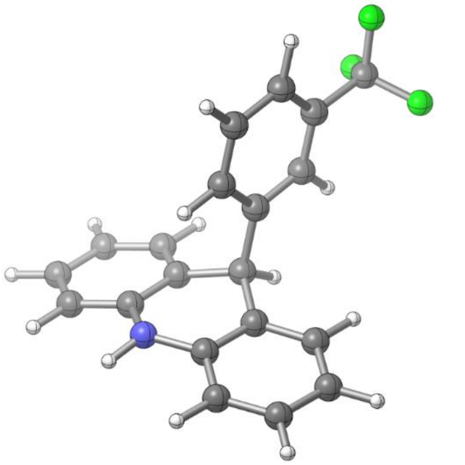

C	-2.6676653070	2.3503868570	-0.5379407580
H	-3.6003784430	2.2965914490	-1.0880382950
C	-2.2189456320	3.5563327620	-0.0330540950
C	-1.0312849900	3.6173203130	0.6867347600
C	-0.2976828140	2.4583005600	0.8790097810
H	-2.6937926820	-4.5221406550	-0.2574938500
H	-3.5424657170	-2.3743966700	-1.1211413530
H	0.6966599940	-2.5019434420	1.3970033480
H	-0.5694599070	-4.5927223150	1.0290525420
H	-2.8041118000	4.4531745540	-0.1960129660
H	-0.6807101030	4.5587394700	1.0897580160
H	0.6364255110	2.4948971620	1.4292621610
N	-2.3801465840	-0.0257401970	-0.8378323650
C	1.3041665570	0.0168730450	-0.4547851300
C	2.6043263430	0.0339461390	0.0213536360
C	1.0961653380	0.0198254430	-1.8341081760
C	3.6791297950	0.0534627080	-0.8627549350
C	2.1647905320	0.0396687460	-2.7105867000
H	0.0854211840	0.0064481450	-2.2252905110
C	3.4681937920	0.0569948940	-2.2298804550
H	2.7841478810	0.0320795810	1.0903896030
H	0.5578054460	-0.0056973810	1.5227050080
H	-3.3091712370	-0.0344238590	-1.2265097620
H	4.3025852070	0.0727648770	-2.9173521100
C	5.0681264120	0.0788401430	-0.2918394530
F	5.2884487510	1.1851563440	0.4443684470
F	6.0210760700	0.0463352340	-1.2311801600
F	5.2951974470	-0.9653761810	0.5270176510
H	1.9877817040	0.0416672500	-3.7785487450

HCyA19

E(RwB97XD) = -1359.66356055

Charge = 0 Multiplicity = 1

C	-7.8072808757	-1.0477686353	-1.6093574073
C	-6.7296546400	-1.5957103697	-0.9423560452
C	-5.7975478969	-0.7719168629	-0.3069414827

C	-5.9406413892	0.6170551883	-0.3458163964
C	-7.0320079361	1.1418363498	-1.0306545074
C	-7.9644653607	0.3315591891	-1.6566617577
C	-4.9759678802	1.5333237198	0.4205885893
C	-3.6786087630	0.7884079732	0.7400287429
C	-3.6357310850	-0.6075316897	0.7527890847
C	-2.4685368686	-1.2767423814	1.1309681161
H	-2.4623656712	-2.3609725026	1.1334798684
C	-1.3382667899	-0.5670580679	1.4820199765
C	-1.3539394929	0.8220236181	1.4468534676
C	-2.5152395816	1.4767056887	1.0750643904
H	-8.5234306919	-1.6984826154	-2.0961358560
H	-6.5958202207	-2.6708576073	-0.9009481343
H	-7.1599969904	2.2166748477	-1.0708568212
H	-8.8048987336	0.7730430909	-2.1767093675
H	-0.4398388770	-1.0984897720	1.7713364179
H	-0.4702639873	1.3899402040	1.7080927810
H	-2.5215467186	2.5588820826	1.0452098577
N	-4.7466515669	-1.3417202884	0.3850573864
C	-4.6692723045	2.7256116086	-0.5031107330
C	-3.8418936996	2.5236227908	-1.6105176233
C	-5.2397077083	3.9787451624	-0.3487661673
C	-3.5820854015	3.5353080639	-2.5143358588
C	-4.9772756030	5.0001504912	-1.2576870476
H	-5.9069535693	4.1844778344	0.4760808990
C	-4.1473706706	4.7918591996	-2.3428150153
H	-3.9435251734	5.5861769252	-3.0474240447
H	-4.6284784905	-2.3379111798	0.3001138028
C	-5.6856949200	1.9701915728	1.7502186451
C	-6.1894252249	0.7863949652	2.5837711498
C	-4.8525406336	2.8815623469	2.6588317308
H	-6.5781914055	2.5256436484	1.4396321288
C	-7.0111779295	1.2603476739	3.7807351980
H	-5.3326436147	0.2031399382	2.9382609142
H	-6.8010359759	0.1208151934	1.9736564310
C	-5.6821823621	3.3919521870	3.8342718780

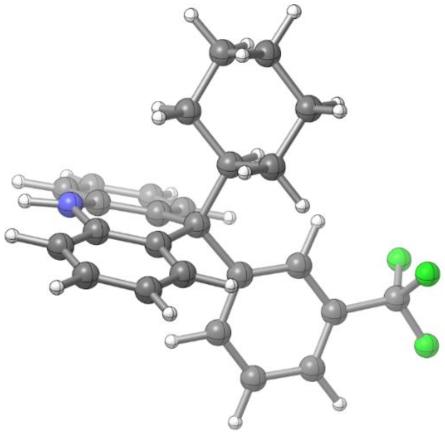

H	-4.0047959211	2.3133864443	3.0517868568
H	-4.4327684498	3.7235848232	2.1070890311
C	-6.2340180773	2.2343480465	4.6591149011
H	-7.3367566941	0.3978480832	4.3676261956
H	-7.9190213177	1.7525239322	3.4137455730
H	-5.0708881395	4.0460187577	4.4609306662
H	-6.5121985723	4.0001162335	3.4567314572
H	-6.8696104353	2.6082821235	5.4657684687
H	-5.3988372009	1.7047601341	5.1312393026
H	-3.3956028193	1.5495150608	-1.7676492825
C	-5.6311662388	6.3311025005	-1.0229102827
F	-5.2956409316	7.2488697727	-1.9374306094
F	-6.9755050521	6.2412049107	-1.0400096487
F	-5.3084896495	6.8461301650	0.1791747028
H	-2.9362765322	3.3473286755	-3.3625204125

A20

E(RwB97XD) = -1198.97263293

Charge = 0 Multiplicity = 1

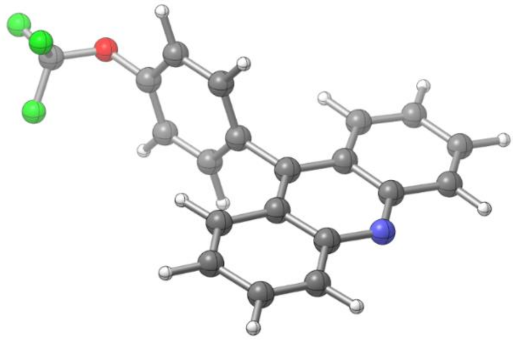

C	-7.8795658540	-1.2979659700	-0.9387636020
C	-6.8046546173	-1.7516633793	-0.2456061523
C	-5.7167863665	-0.8815742115	0.0628467300
C	-5.7794417626	0.4790594214	-0.3720099777
C	-6.9290412493	0.9143635823	-1.0979130155
C	-7.9433914015	0.0545492640	-1.3712191867
C	-4.7075022913	1.3221232334	-0.0626284544
C	-3.6241649014	0.8044099497	0.6543573800
C	-3.6644234513	-0.5718891892	1.0399075752
C	-2.5651750441	-1.1123371103	1.7715781204
H	-2.6141558943	-2.1564570291	2.0545728689
C	-1.5002697525	-0.3361721056	2.0960067477
C	-1.4587977513	1.0320068762	1.7130763523
C	-2.4845792910	1.5843642208	1.0163207346
H	-8.7000910382	-1.9667938908	-1.1677179884
H	-6.7388836045	-2.7784410318	0.0921339098
H	-6.9827528424	1.9432839310	-1.4293103941

H	-8.8105773199	0.3957028997	-1.9224852010
H	-0.6709491291	-0.7566282111	2.6514063521
H	-0.5992764683	1.6331109477	1.9817907040
H	-2.4476503277	2.6265964994	0.7270186679
N	-4.6853212987	-1.3784717016	0.7468821160
C	-4.7208314645	2.7477959683	-0.4885794638
C	-4.2094215384	3.1147553989	-1.7287109936
C	-5.2404018036	3.7259074893	0.3526355248
C	-4.2175978105	4.4409601149	-2.1301002885
C	-5.2550733225	5.0553918190	-0.0372988599
H	-5.6344649728	3.4474537698	1.3219845663
C	-4.7483900801	5.3899163329	-1.2780289757
H	-3.8154405676	4.7364699298	-3.0899700649
H	-5.6515913465	5.8239329275	0.6127321671
H	-3.7980787063	2.3588263232	-2.3858546792
O	-4.7034486687	6.7388640337	-1.6499395106
C	-5.7477039584	7.2365656551	-2.3274666543
F	-5.5012645407	8.5169904689	-2.5660301200
F	-5.9474080251	6.6245970444	-3.5005798333
F	-6.8976715367	7.1518407288	-1.6485651012

HA20

E(UwB97XD) = -1199.57029765

Charge = 0 Multiplicity = 2

C	-7.9946844780	-1.3791442970	-0.7333403910
C	-6.8201635060	-1.8230892840	-0.1515436510
C	-5.7745969840	-0.9350859340	0.0836991500
C	-5.8916894610	0.4356881300	-0.2575150530
C	-7.0983208520	0.8468002000	-0.8593700560
C	-8.1299283780	-0.0395748770	-1.0910201570
C	-4.7968445460	1.3142722750	-0.0003087040
C	-3.6011762470	0.8037355010	0.5879868140
C	-3.5211808500	-0.5747068830	0.9089467280
C	-2.3719629530	-1.1105038730	1.4830033500
H	-2.3416528480	-2.1696895700	1.7122475030
C	-1.2861876240	-0.2979846720	1.7586466010

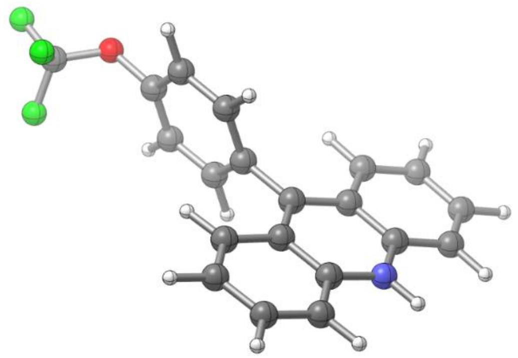

Go back to table of contents

C	-1.3444058910	1.0627006000	1.4656739960
C	-2.4793933810	1.6004505790	0.8945486460
H	-8.8023335030	-2.0776147290	-0.9123562530
H	-6.7000544730	-2.8644824750	0.1242318200
H	-7.2123265680	1.8832914270	-1.1482463290
H	-9.0440195530	0.3092834330	-1.5548332350
H	-0.3967883560	-0.7238807220	2.2056022450
H	-0.4999043670	1.7027441250	1.6883135880
H	-2.5152014970	2.6598870720	0.6774663920
N	-4.6040377720	-1.3788041210	0.6488592670
C	-4.9029151490	2.7522666000	-0.3451872020
C	-4.1863405490	3.2825400740	-1.4166243530
C	-5.7203089890	3.6028622480	0.3971829620
C	-4.2838664360	4.6246003300	-1.7472000330
C	-5.8284392290	4.9471718640	0.0796822840
H	-6.2781885720	3.2080868090	1.2373091780
C	-5.1122340590	5.4352453940	-0.9958125220
H	-3.7263940480	5.0382285390	-2.5772475090
H	-6.4560995940	5.6101303830	0.6603810410
H	-3.5424803460	2.6362994240	-2.0003210840
H	-4.5338200580	-2.3564280100	0.8835564150
O	-5.1707475180	6.8047249140	-1.2876342220
C	-6.1107742030	7.2207036760	-2.1467140350
F	-5.9831088990	8.5338754190	-2.2816196740
F	-7.3549735120	6.9649606380	-1.7243897620
F	-5.9947196330	6.6641442820	-3.3583831830

H_2A20

E(RwB97XD) = -1200.19619989

Charge = 0 Multiplicity = 1

C	-7.7162065330	-1.2017881267	-1.3203335136
C	-6.3408833074	-1.3403562293	-1.3266406937
C	-5.5452695247	-0.4855297231	-0.5616252661
C	-6.1387777487	0.5201093767	0.2042534616
C	-7.5235448095	0.6304456105	0.2080655889
C	-8.3186198817	-0.2177146882	-0.5451719828

C	-5.2782195010	1.5051823236	0.9687785832
C	-3.9058901121	0.9304656096	1.2556148468
C	-3.3886407861	-0.0877263412	0.4519760169
C	-2.0916823063	-0.5561603016	0.6692408721
H	-1.7003572215	-1.3453460605	0.0371867365
C	-1.3210909696	-0.0239653459	1.6859891414
C	-1.8343345262	0.9739573798	2.5062675191
C	-3.1199194300	1.4387100645	2.2821340670
H	-8.3222066788	-1.8717443550	-1.9180037455
H	-5.8688714434	-2.1168751196	-1.9180113374
H	-7.9817990293	1.4097821964	0.8074442005
H	-9.3957362836	-0.1114217482	-0.5320189042
H	-0.3164942228	-0.3968292474	1.8442180852
H	-1.2365228234	1.3870607453	3.3085232133
H	-3.5265436921	2.2258129764	2.9080118904
N	-4.1661831594	-0.6291643946	-0.5605595630
C	-5.1710649079	2.8327289804	0.2296305194
C	-4.6130192714	2.8999783933	-1.0455772000
C	-5.6339582242	4.0022576100	0.8189294669
C	-4.5155880360	4.1058242552	-1.7163524248
C	-5.5442659705	5.2208080655	0.1601173957
H	-6.0756063957	3.9660767047	1.8076607165
C	-4.9786385848	5.2529434778	-1.0971505282
H	-4.0893475741	4.1605573214	-2.7094053527
H	-5.9115665202	6.1312018380	0.6152801106
H	-4.2505970788	1.9997185152	-1.5273284919
H	-5.7664909590	1.7147764740	1.9227543753
H	-3.7958358939	-1.4401722572	-1.0291828275
O	-4.9408739552	6.4666030123	-1.7984620146
C	-3.8522372425	7.2341108082	-1.6600160715
F	-3.6458885796	7.6260846753	-0.3966784896
F	-4.0235488977	8.3185944767	-2.4046447964
F	-2.7291529679	6.6269270577	-2.0618153937

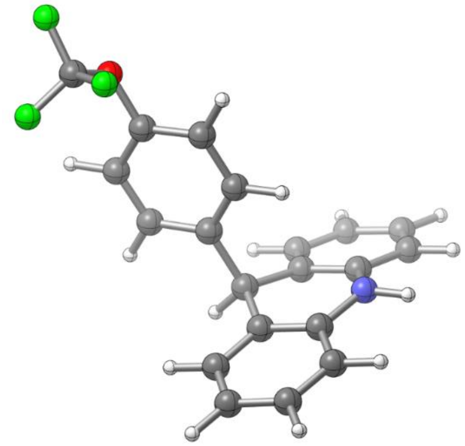

HCyA20

E(RwB97XD) = -1434.89105142

Go back to table of contents

Charge = 0 Multiplicity = 1

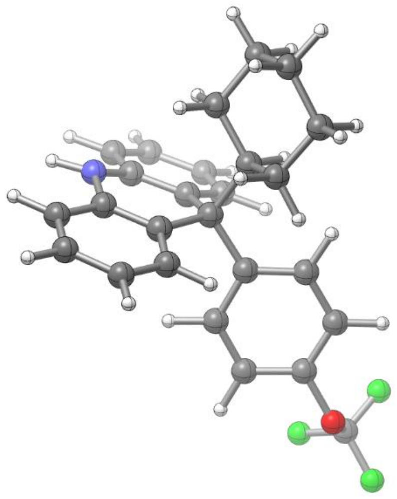

C	-7.6530318543	-0.7398596262	-1.6125610673
C	-6.4397641438	-1.2817519607	-1.2374004444
C	-5.5927037381	-0.5879256694	-0.3700372604
C	-5.9599474797	0.6666738627	0.1221696395
C	-7.1845468926	1.1915558773	-0.2784118560
C	-8.0331411946	0.5067630784	-1.1320797457
C	-5.0820548808	1.4039475936	1.1437172097
C	-3.6558492564	0.8508010183	1.1121441661
C	-3.3878377559	-0.4190881215	0.5961494042
C	-2.0947602722	-0.9471732605	0.6505827980
H	-1.9131964273	-1.9353156257	0.2433060044
C	-1.0632726363	-0.2155196988	1.2029218531
C	-1.3060889234	1.0608095812	1.6958946605
C	-2.5897158936	1.5750491313	1.6398558165
H	-8.3005519709	-1.2895775640	-2.2847332213
H	-6.1315734064	-2.2526394532	-1.6086896240
H	-7.4857651484	2.1637275590	0.0922859369
H	-8.9810666026	0.9427428973	-1.4203654533
H	-0.0657635494	-0.6363104195	1.2375549135
H	-0.5031717325	1.6497235258	2.1203562008
H	-2.7728061016	2.5721719467	2.0195479378
N	-4.3991218508	-1.1639450314	0.0201147212
C	-5.0686172279	2.8867451905	0.7287076109
C	-4.3424006760	3.2534280923	-0.4053283915
C	-5.8073710046	3.8742579454	1.3698823272
C	-4.3392303123	4.5515709421	-0.8798040087
C	-5.8167916709	5.1849969263	0.9104985293
H	-6.4000386465	3.6428275749	2.2427115541
C	-5.0850132849	5.5047172452	-0.2116527034
H	-3.7671042193	4.8237872650	-1.7569247237
H	-6.3882870133	5.9477092503	1.4228465423
H	-3.7648778701	2.5060536265	-0.9344331238
H	-4.1314484567	-2.0183073944	-0.4404460810
C	-5.7117392240	1.1816221055	2.5641285990
C	-5.9322603209	-0.2970233928	2.9038720224

C	-4.9441195064	1.8336017831	3.7199022214
H	-6.7081295791	1.6365564032	2.5198739178
C	-6.6884145426	-0.4576314587	4.2211916619
H	-4.9618215892	-0.7998687974	2.9775248300
H	-6.4915936386	-0.7936273793	2.1102652978
C	-5.7162424457	1.7122093470	5.0313504857
H	-3.9806501667	1.3297828359	3.8368786398
H	-4.7237805375	2.8814893065	3.5131219057
C	-5.9870265012	0.2513916260	5.3741760437
H	-6.8107366407	-1.5197724375	4.4475855934
H	-7.6959404489	-0.0426888349	4.1033242030
H	-5.1545693710	2.1925611334	5.8363652207
H	-6.6679013283	2.2491580615	4.9446595800
H	-6.5848078266	0.1767493424	6.2861021606
H	-5.0331884164	-0.2486498878	5.5766964323
O	-5.0392741967	6.8373594227	-0.6445386697
C	-5.9513511696	7.2339120149	-1.5411628883
F	-5.7278095448	8.5140091373	-1.8079412385
F	-5.8859094118	6.5519984115	-2.6910953727
F	-7.2085984996	7.1158889526	-1.0961407674

A21

E(RwB97XD) = -1198.97274786

Charge = 0 Multiplicity = 1

C	-7.7859873494	-1.5061150558	-0.9511519379
C	-6.6802842870	-1.8430592072	-0.2403626176
C	-5.6833832294	-0.8681424281	0.0622208933
C	-5.8657667290	0.4713097472	-0.4039673425
C	-7.0475517215	0.7833726229	-1.1418615776
C	-7.9742441005	-0.1728052331	-1.4058817214
C	-4.8795558457	1.4163015542	-0.1014344526
C	-3.7646993477	1.0173359220	0.6440411826
C	-3.6862383818	-0.3473576884	1.0637389397
C	-2.5559125664	-0.7684135677	1.8258668169
H	-2.5172166677	-1.8049882000	2.1367409867
C	-1.5689952480	0.1071382688	2.1433281643

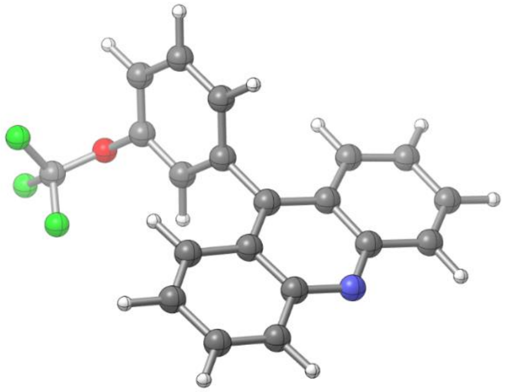

Go back to table of contents

C	-1.6406664473	1.4616743573	1.7191348732
C	-2.7001790585	1.9023958169	0.9939223940
H	-8.5373721358	-2.2535903217	-1.1739671910
H	-6.5215540487	-2.8522751874	0.1187412102
H	-7.1997632107	1.7975200546	-1.4872748709
H	-8.8672682810	0.0771214177	-1.9647628930
H	-0.7150614116	-0.2227778636	2.7220976525
H	-0.8395050323	2.1425866474	1.9774301621
H	-2.7453670227	2.9345484454	0.6721123184
N	-4.6223732690	-1.2516674343	0.7731646189
C	-5.0112077377	2.8234309841	-0.5656071102
C	-4.7439925089	3.1600688220	-1.8888345256
C	-5.3999806726	3.8152212494	0.3273337762
C	-4.8647560892	4.4727979157	-2.3177039928
C	-5.5041722244	5.1172830875	-0.1210602042
H	-5.6189491237	3.5781037184	1.3602619037
C	-5.2502167871	5.4677910167	-1.4327287302
H	-4.6583472598	4.7246840004	-3.3498497878
H	-4.4427167661	2.3875817143	-2.5852199685
O	-5.9480718869	6.1020564347	0.7695622874
C	-5.0240199513	6.7476316491	1.4958608692
F	-5.6528586651	7.6344614968	2.2538105080
F	-4.1316996732	7.3998394119	0.7416038868
F	-4.3275632410	5.9328074586	2.2970150794
H	-5.3542873112	6.4968302733	-1.7499466999

HA21

E(UwB97XD) = -1199.57067577

Charge = 0 Multiplicity = 2

C	-2.8835248776	-3.6844016699	-0.5604263558
C	-3.5257062486	-2.4617645602	-0.4777684908
C	-2.7924296241	-1.2970294221	-0.2690506672
C	-1.3808778903	-1.3348887466	-0.1471515120
C	-0.7653442548	-2.6009345841	-0.2217222861
C	-1.4983550690	-3.7514104780	-0.4270384341
C	-0.6667310424	-0.1169643469	0.0663436641

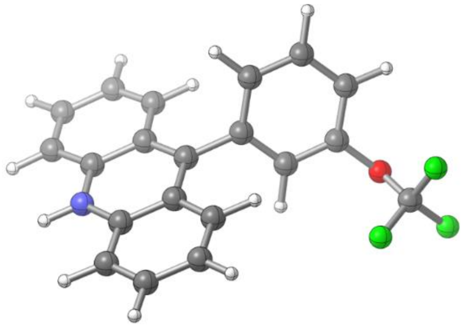

C	-1.3807139506	1.1162370029	0.1639555391
C	-2.7927831798	1.1090285799	0.0428431362
C	-3.5261963610	2.2889075115	0.1305724405
H	-4.6054036426	2.2465615809	0.0374076097
C	-2.8821036381	3.4967997913	0.3308401139
C	-1.4935635485	3.5336849689	0.4381720131
C	-0.7605640948	2.3681419961	0.3532654635
H	-3.4617791138	-4.5851692815	-0.7230239524
H	-4.6038365475	-2.3961255234	-0.5702525504
H	0.3086986462	-2.6682415999	-0.1107259369
H	-0.9931451820	-4.7075890180	-0.4803107755
H	-3.4606203498	4.4094725840	0.3979411795
H	-0.9848741042	4.4781001774	0.5854212282
H	0.3175765573	2.4130968002	0.4305273044
N	-3.4332969038	-0.0866490088	-0.1709205290
C	0.8098200068	-0.1306062309	0.1848490178
C	1.4217292273	0.1745496123	1.3986196512
C	1.6154530479	-0.4430369415	-0.9096806863
C	2.7995925943	0.1715221986	1.4846289218
C	2.9968862410	-0.4539147015	-0.7973023880
H	1.1504617671	-0.6817121385	-1.8582578554
C	3.6064174257	-0.1453109662	0.4094105163
H	3.6066960989	-0.7020861992	-1.6566306454
H	0.8283573042	0.4137625231	2.2716817532
H	-4.4369026924	-0.0749671056	-0.2611085876
H	4.6826704004	-0.1498627053	0.5185828953
O	3.3962835465	0.4311871028	2.7265497597
C	3.6875025309	1.7051015390	3.0215816270
F	4.2276039548	1.7272710907	4.2324790119
F	2.6104345298	2.4997387021	3.0311233054
F	4.5600564346	2.2525214666	2.1663955000

H_2A21

E(RwB97XD) = -1200.19680271

Charge = 0 Multiplicity = 1

C	-2.1650619280	-3.5680005312	-0.0116441584

C	-2.5025902335	-2.3968859913	-0.6639395899
C	-1.7496152547	-1.2389364083	-0.4627430272
C	-0.6437907893	-1.2663040153	0.3895959541
C	-0.3330512486	-2.4494110639	1.0478595475
C	-1.0802179288	-3.6001101907	0.8568238242
C	0.2302457455	-0.0385995172	0.5399849435
C	-0.5351923048	1.2247396116	0.2031733058
C	-1.6434930026	1.1672299103	-0.6443786549
C	-2.2926268440	2.3434959679	-1.0227197503
H	-3.1502582676	2.2875498898	-1.6835642046
C	-1.8496707885	3.5648706120	-0.5503262176
C	-0.7618950437	3.6312246770	0.3124560063
C	-0.1175326461	2.4610580017	0.6789551931
H	-2.7579795696	-4.4594268441	-0.1762679593
H	-3.3574118968	-2.3644809191	-1.3299487941
H	0.5235132002	-2.4649613704	1.7131924084
H	-0.8185946946	-4.5134889203	1.3754318456
H	-2.3628667540	4.4701739411	-0.8508529479
H	-0.4177824197	4.5854302655	0.6902146402
H	0.7409090913	2.5007049160	1.3408793674
N	-2.0932456557	-0.0602991668	-1.1078589210
C	1.4886505135	-0.1519016297	-0.3102410797
C	2.7457753266	-0.1370718463	0.2802653212
C	1.3971902400	-0.2677169027	-1.6949625305
C	3.8966314783	-0.2374834517	-0.4906207414
C	2.5516346278	-0.3545079315	-2.4427323885
H	0.4360132736	-0.2913131986	-2.1931650963
C	3.8097531474	-0.3488332289	-1.8678341675
H	2.8269099456	-0.0494120956	1.3572178667
H	0.5576123232	0.0249021013	1.5795081379
H	-2.9662890577	-0.0594990839	-1.6102780921
H	4.6924097673	-0.4317295384	-2.4876967893
H	4.8687538070	-0.2302873649	-0.0142318187
O	2.4447521561	-0.5267444798	-3.8303820836
C	2.3948838154	0.5724032799	-4.5941179987
F	1.3411017376	1.3516249929	-4.3235932547

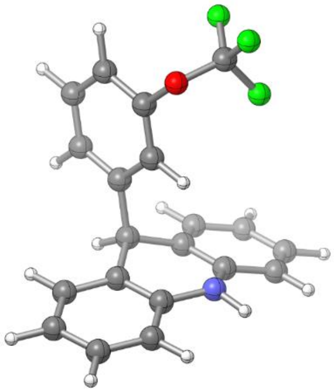

F	3.4833306894	1.3422121606	-4.4727611012
F	2.3046584315	0.1823682911	-5.8589507174

HCyA21

E(RwB97XD) = -1434.89188294

Charge = 0 Multiplicity = 1

C	-7.8109566070	-1.1771843885	-1.5234483754
C	-6.6819494483	-1.6629167860	-0.8942955668
C	-5.7707354117	-0.7865893777	-0.3002328983
C	-5.9868809461	0.5928512488	-0.3430709752
C	-7.1288858084	1.0545203863	-0.9897837600
C	-8.0409591212	0.1917792610	-1.5742498329
C	-5.0421381912	1.5637290237	0.3797697092
C	-3.6951257556	0.8894455922	0.6487909158
C	-3.5791577985	-0.5020448890	0.6676599772
C	-2.3624222525	-1.1070612892	0.9951643844
H	-2.2991228390	-2.1894416378	1.0033446275
C	-1.2556836597	-0.3370597137	1.2891266721
C	-1.3453607644	1.0490490946	1.2473954984
C	-2.5548251557	1.6397081721	0.9262142190
H	-8.5099530384	-1.8681613230	-1.9786005614
H	-6.4906825347	-2.7292475904	-0.8518575832
H	-7.3128418780	2.1210555909	-1.0328486658
H	-8.9218547434	0.5845653668	-2.0656526232
H	-0.3182130526	-0.8190277283	1.5384771763
H	-0.4811178303	1.6640355976	1.4630043196
H	-2.6179438184	2.7198356622	0.8886228197
N	-4.6664428941	-1.2954778693	0.3551973728
C	-4.8349245316	2.7662110839	-0.5594757793
C	-4.0301899678	2.5895223106	-1.6842261094
C	-5.4673619391	3.9943639872	-0.4020402469
C	-3.8582542267	3.6195535512	-2.5815504562
C	-5.2929333909	5.0188114566	-1.3246827861
H	-6.1199103794	4.1729128195	0.4402912527
C	-4.4796855511	4.8448557906	-2.4297518725
H	-4.3307509071	5.6299339919	-3.1588666264

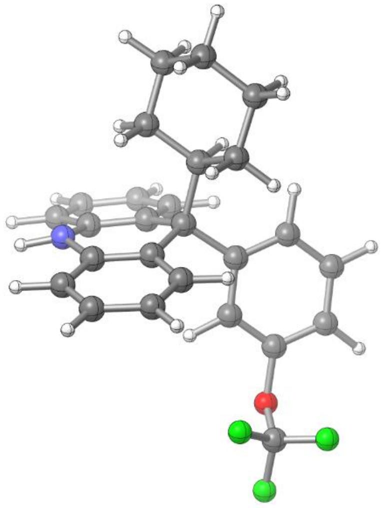

H	-4.4989697662	-2.2844493208	0.2679719594
C	-5.7193797146	1.9720917775	1.7349184116
C	-6.1264663920	0.7692270795	2.5937369518
C	-4.8970284333	2.9310257542	2.6030657856
H	-6.6504836408	2.4798282026	1.4583117395
C	-6.9181654857	1.2073902675	3.8241437137
H	-5.2270650661	0.2309949582	2.9120703712
H	-6.7299706674	0.0704975007	2.0133627395
C	-5.6993593843	3.4039881440	3.8126648142
H	-4.0037154804	2.4110439325	2.9602337031
H	-4.5481751967	3.7898217375	2.0284563002
C	-6.1535776504	2.2238357802	4.6645828825
H	-7.1740764348	0.3326968618	4.4272535303
H	-7.8648995102	1.6513956185	3.4958589642
H	-5.0958415331	4.0920323105	4.4098563777
H	-6.5760845238	3.9661575933	3.4709135937
H	-6.7707440337	2.5685964896	5.4981141979
H	-5.2720587516	1.7397954718	5.0998758718
H	-3.5347214277	1.6450827736	-1.8665417934
O	-3.0804637832	3.3924892779	-3.7267777315
C	-1.7659368605	3.6334511990	-3.6430742562
F	-1.4871991737	4.9079853185	-3.3407579977
F	-1.1508053154	2.8785821374	-2.7249573783
F	-1.2282104400	3.3636396017	-4.8253994814
H	-5.8005878914	5.9634931382	-1.1765934953

A22

E(RwB97XD) = -634.215991726

Charge = 0 Multiplicity = 1

C	-7.9076740302	-1.1754078317	-0.5901023832
C	-6.7625079890	-1.6783641716	-0.0648757438
C	-5.6171029217	-0.8478334884	0.1233214477
C	-5.6832642878	0.5270610800	-0.2639352215
C	-6.9123425009	1.0115201354	-0.8087076232
C	-7.9843511716	0.1925777556	-0.9640124552
C	-4.5496163158	1.3345896927	-0.0815597962

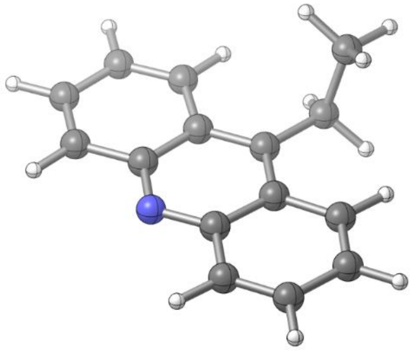

C	-3.4162342261	0.7531138154	0.5082099441
C	-3.4585871999	-0.6328151283	0.8582441780
C	-2.3084589023	-1.2349801360	1.4514009577
H	-2.3665194197	-2.2875889737	1.6993889199
C	-1.1906793938	-0.5067271512	1.6971431243
C	-1.1470969178	0.8734269555	1.3649555830
C	-2.2180826600	1.4791667588	0.7904240966
H	-8.7719699316	-1.8139770869	-0.7255469929
H	-6.6816718409	-2.7170245978	0.2309653612
H	-6.9956715418	2.0506010421	-1.0953641238
H	-8.9074975749	0.5811790666	-1.3753683268
H	-0.3247829121	-0.9731081599	2.1508021767
H	-0.2503883702	1.4431965569	1.5741636564
H	-2.1639604328	2.5322406734	0.5518138154
N	-4.5297042608	-1.4021383313	0.6605154151
C	-4.5653985573	2.7899088504	-0.4641739459
H	-3.5688775738	3.1027100749	-0.7733137462
H	-5.2052248607	2.9371591560	-1.3333650547
C	-5.0478305558	3.6800686188	0.6823350660
H	-6.0615461115	3.4115152118	0.9859323070
H	-4.3981884235	3.5806958526	1.5541332240
H	-5.0495920254	4.7274606898	0.3757871305

HA22

E(RwB97XD) = -634.215991726

Charge = 0 Multiplicity = 1

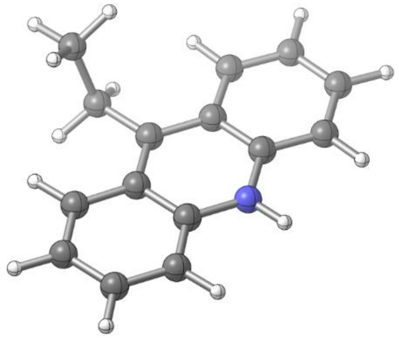

C	-7.9076740302	-1.1754078317	-0.5901023832
C	-6.7625079890	-1.6783641716	-0.0648757438
C	-5.6171029217	-0.8478334884	0.1233214477
C	-5.6832642878	0.5270610800	-0.2639352215
C	-6.9123425009	1.0115201354	-0.8087076232
C	-7.9843511716	0.1925777556	-0.9640124552
C	-4.5496163158	1.3345896927	-0.0815597962
C	-3.4162342261	0.7531138154	0.5082099441
C	-3.4585871999	-0.6328151283	0.8582441780
C	-2.3084589023	-1.2349801360	1.4514009577

H	-2.3665194197	-2.2875889737	1.6993889199
C	-1.1906793938	-0.5067271512	1.6971431243
C	-1.1470969178	0.8734269555	1.3649555830
C	-2.2180826600	1.4791667588	0.7904240966
H	-8.7719699316	-1.8139770869	-0.7255469929
H	-6.6816718409	-2.7170245978	0.2309653612
H	-6.9956715418	2.0506010421	-1.0953641238
H	-8.9074975749	0.5811790666	-1.3753683268
H	-0.3247829121	-0.9731081599	2.1508021767
H	-0.2503883702	1.4431965569	1.5741636564
H	-2.1639604328	2.5322406734	0.5518138154
N	-4.5297042608	-1.4021383313	0.6605154151
C	-4.5653985573	2.7899088504	-0.4641739459
H	-3.5688775738	3.1027100749	-0.7733137462
H	-5.2052248607	2.9371591560	-1.3333650547
C	-5.0478305558	3.6800686188	0.6823350660
H	-6.0615461115	3.4115152118	0.9859323070
H	-4.3981884235	3.5806958526	1.5541332240
H	-5.0495920254	4.7274606898	0.3757871305

H_2A22

E(RwB97XD) = -635.440236995

Charge = 0 Multiplicity = 1

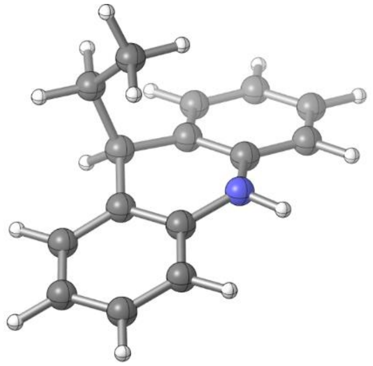

C	-3.4223594171	-0.8021316101	-0.3756744264
C	-2.2022494692	-1.3566787043	-0.7176673747
C	-1.0207833955	-0.6512894692	-0.4849141881
C	-1.0653086693	0.6239930513	0.0851926047
C	-2.3012485005	1.1509491444	0.4377171695
C	-3.4793053667	0.4550208224	0.2131310382
C	0.2038463994	1.4290557441	0.2474577836
C	1.4160090559	0.5289142757	0.3206594780
C	1.3826047404	-0.7434045119	-0.2566134578
C	2.5295397831	-1.5382927122	-0.2677831497
H	2.4889654757	-2.5222434917	-0.7215682177
C	3.7020905325	-1.0758985786	0.3017482036
C	3.7428046633	0.1776736257	0.8994033995

C	2.6001617808	0.9631445261	0.9026925616
H	-4.3325290245	-1.3594678957	-0.5613315922
H	-2.1521687590	-2.3441528714	-1.1627839284
H	-2.3356809581	2.1366752949	0.8896235579
H	-4.4313312907	0.8883929989	0.4921173668
H	4.5855771495	-1.7025751169	0.2873181111
H	4.6555287878	0.5394616627	1.3554960809
H	2.6239793948	1.9467849014	1.3597354788
N	0.2039183858	-1.2095229234	-0.8197022721
C	0.3510393592	2.4831212443	-0.8740330371
H	1.2360931639	3.0864212124	-0.6523593910
C	0.4628688361	1.9111177023	-2.2793931378
H	0.5631082540	2.7151280438	-3.0110781450
H	1.3359579141	1.2621678254	-2.3781409596
H	-0.4223809811	1.3290685563	-2.5453526045
H	0.1364035643	1.9861109949	1.1856005546
H	0.1949370166	-2.1733609276	-1.1117769529
H	-0.5112543154	3.1538011062	-0.8190454037

HCyA22

E(RwB97XD) = -870.136002669

Charge = 0 Multiplicity = 1

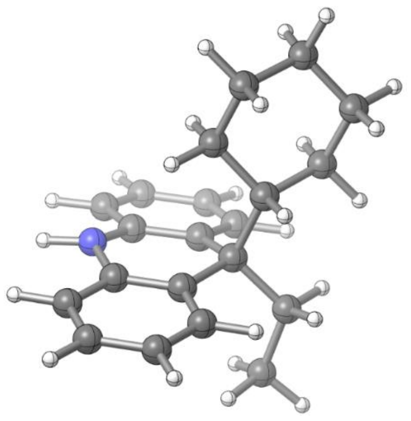

C	-3.6741684129	-0.8007297190	-0.3817825069
C	-2.4945829164	-1.4096674846	-0.0037213918
C	-1.3101828370	-0.6730202478	0.0778827977
C	-1.2958048146	0.6964341133	-0.2098741030
C	-2.4997143976	1.2737050237	-0.6091102247
C	-3.6792593629	0.5515089760	-0.6948218525
C	-0.0199396078	1.5281638520	-0.0490513451
C	1.2115098587	0.6165413945	0.0092845665
C	1.0971455058	-0.7508835144	0.2710577311
C	2.2349808867	-1.5542387290	0.3832431140
H	2.1132185197	-2.6129233867	0.5839371518
C	3.4944651149	-1.0067475001	0.2441996573
C	3.6306665479	0.3517235303	-0.0102938505
C	2.4959128892	1.1381309620	-0.1234187315

H	-4.5854961586	-1.3832711495	-0.4407406620
H	-2.4713425214	-2.4685551286	0.2287096516
H	-2.5260345704	2.3258415108	-0.8569738581
H	-4.5934594603	1.0430252544	-1.0024540214
H	4.3687422311	-1.6399801268	0.3337482872
H	4.6118532358	0.7963395089	-0.1181758006
H	2.6185481209	2.1965669805	-0.3151402180
N	-0.1482289558	-1.3187955130	0.4511319572
C	0.1271535678	2.5076415914	-1.2389393295
H	-0.7446642337	3.1579433843	-1.2822300910
C	0.3038319566	1.8422806021	-2.5965365531
H	1.2101001744	1.2353567983	-2.6370755877
H	-0.5422445251	1.1932617588	-2.8332513273
H	0.3728671374	2.5974569670	-3.3822789343
H	-0.1883699965	-2.3208322577	0.5376559738
C	-0.0474579754	2.3377896381	1.2989051273
C	-1.1112311812	3.4366795550	1.3872831744
C	-0.1560570726	1.4345959118	2.5316762459
H	0.9297128710	2.8356201559	1.3443915295
C	-0.9766095280	4.2410793741	2.6794902940
H	-2.1069948424	2.9841354751	1.3591790168
H	-1.0431876417	4.1151762071	0.5356815798
C	-0.0310440206	2.2240528828	3.8319836858
H	-1.1248496575	0.9227275803	2.5140861306
H	0.6130444172	0.6608632569	2.5039129908
C	-1.0610229245	3.3447648017	3.9093252583
H	-1.7524991042	5.0100821266	2.7162297539
H	-0.0133097406	4.7639568806	2.6795545059
H	-0.1375672773	1.5498092809	4.6856253319
H	0.9750217832	2.6545675657	3.8935810046
H	-0.9183901075	3.9322749299	4.8199615868
H	-2.0644742606	2.9076749189	3.9660954207
H	0.9702098671	3.1732356279	-1.0410451160

A23

E(RwB97XD) = -865.281367435

Charge = 0 Multiplicity = 1

C	-7.8590031456	-1.3714833206	-0.9505726895
C	-6.7497835065	-1.8136520048	-0.3054105174
C	-5.6923647090	-0.9146325503	0.0258043681
C	-5.8255291489	0.4635908966	-0.3287348162
C	-7.0081718838	0.8853413055	-1.0072377512
C	-7.9901381249	-0.0022683459	-1.3089703037
C	-4.7846612340	1.3392464301	-0.0018304808
C	-3.6583581207	0.8268151691	0.6508014069
C	-3.6263854517	-0.5686500493	0.9587423146
C	-2.4796808204	-1.0989185055	1.6221332591
H	-2.4747835426	-2.1577732811	1.8493353037
C	-1.4356324142	-0.2961012786	1.9500846215
C	-1.4642401047	1.0908917210	1.6405347568
C	-2.5391575976	1.6344554455	1.0142190536
H	-8.6550413383	-2.0629184464	-1.1983183002
H	-6.6324247272	-2.8533632411	-0.0260419958
H	-7.1106965888	1.9279668993	-1.2792799338
H	-8.8822342392	0.3288762988	-1.8255562131
H	-0.5694120624	-0.7096717136	2.4518679293
H	-0.6200689452	1.7131396419	1.9099185229
H	-2.5600158510	2.6913930279	0.7816074012
N	-4.6206032349	-1.4034622222	0.6518516888
C	-4.8733840458	2.7852738236	-0.3437399351
C	-4.3922530904	3.2623006886	-1.5693492358
C	-5.4413954892	3.6697030031	0.5623340259
C	-4.5012048807	4.6203395611	-1.8393968578
C	-5.5395110514	5.0225161497	0.2709690750
C	-5.0690054387	5.5181808348	-0.9378720991
H	-4.1301993486	4.9935584972	-2.7887716720
H	-5.9872691726	5.6955916102	0.9930031694
C	-3.7728631671	2.3287496099	-2.5701602010
H	-2.8970381846	1.8271012331	-2.1513807067
H	-4.4766490243	1.5485982550	-2.8702307282
H	-3.4607563344	2.8673848003	-3.4646253094
C	-5.1589041531	6.9794526157	-1.2763371233

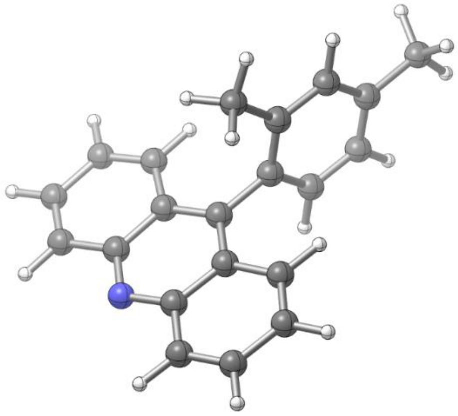

H	-4.1634702396	7.4122447228	-1.4020137720
H	-5.6986602168	7.1317555272	-2.2136971732
H	-5.6732782622	7.5367040918	-0.4930965474
H	-5.8127271090	3.2939290996	1.5087254658

HA23

E(UwB97XD) = -865.878237639

Charge = 0 Multiplicity = 2

C	-7.9265825483	-1.3383541335	-0.9103535133
C	-6.7864394957	-1.7917574628	-0.2681210017
C	-5.7541783607	-0.9088662584	0.0333799179
C	-5.8497960576	0.4644790374	-0.3046674680
C	-7.0230377085	0.8877037036	-0.9603726379
C	-8.0420750397	0.0056992942	-1.2580250592
C	-4.7738700770	1.3411963988	0.0174946022
C	-3.6109994804	0.8246894891	0.6584256499
C	-3.5478835304	-0.5541269414	0.9809810188
C	-2.4256105793	-1.0907421446	1.6045029365
H	-2.4070270322	-2.1490335044	1.8392062081
C	-1.3474843533	-0.2798625292	1.9177250918
C	-1.3844624385	1.0780982103	1.6089600345
C	-2.4952181855	1.6176789304	0.9925721638
H	-8.7243172977	-2.0331727088	-1.1409279425
H	-6.6847853436	-2.8361067901	0.0043382077
H	-7.1181246206	1.9313752148	-1.2316144475
H	-8.9317342523	0.3614059567	-1.7625425789
H	-0.4784498055	-0.7069444540	2.4021737917
H	-0.5421647566	1.7133425157	1.8530003628
H	-2.5203676596	2.6740287811	0.7568413159
N	-4.6188187681	-1.3554417049	0.6656771288
C	-4.8585079726	2.7836657608	-0.3269430395
C	-4.3604075079	3.2640858466	-1.5466711538
C	-5.4393941254	3.6777169675	0.5636717134
C	-4.4634821097	4.6211955009	-1.8245054946
C	-5.5330970465	5.0305007006	0.2677564335
C	-5.0442056415	5.5229289159	-0.9349298604

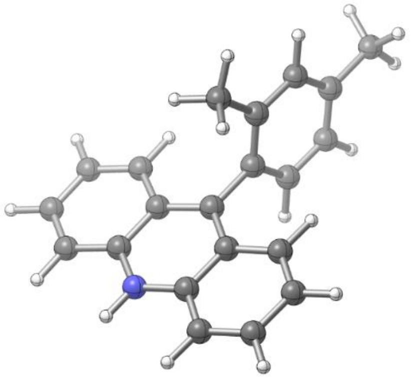

H	-4.0784371709	4.9912473140	-2.7698275140
H	-5.9913712692	5.7062840260	0.9809986758
C	-3.7260155050	2.3273062587	-2.5352436708
H	-2.8432311288	1.8445623209	-2.1084906048
H	-4.4176523507	1.5323783198	-2.8244705025
H	-3.4203206301	2.8581522031	-3.4370017541
H	-5.8258207556	3.3062125250	1.5060568041
H	-4.5621470049	-2.3338998513	0.9001581635
C	-5.1293530664	6.9833415936	-1.2798542275
H	-4.1329260584	7.4137968638	-1.4060508979
H	-5.6678269681	7.1338151042	-2.2184136345
H	-5.6435962972	7.5453207297	-0.4997332172

H_2A23

E(RwB97XD) = -866.501139450

Charge = 0 Multiplicity = 1

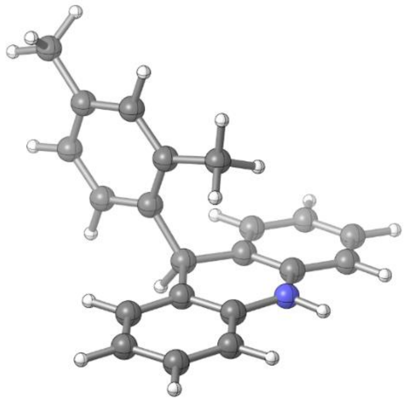

C	-8.0797757941	-1.2296057363	-0.6775266201
C	-7.0041974826	-1.6523135764	0.0799082463
C	-5.8343010043	-0.8902053917	0.1330896342
C	-5.7450507423	0.3033731914	-0.5865885157
C	-6.8428191013	0.7100683098	-1.3352124417
C	-8.0068036174	-0.0389289535	-1.3905082304
C	-4.4855071290	1.1525072484	-0.5626816538
C	-3.4570804334	0.6408540994	0.4317396009
C	-3.6365736314	-0.5659870428	1.1112067835
C	-2.6649891802	-1.0125226081	2.0105856854
H	-2.8153053151	-1.9549377857	2.5253645827
C	-1.5268684430	-0.2630455358	2.2390456187
C	-1.3413401867	0.9445337438	1.5766803281
C	-2.3072571916	1.3793101335	0.6839949732
H	-8.9798076934	-1.8315527998	-0.7090814931
H	-7.0561759077	-2.5797865183	0.6391314984
H	-6.7737947543	1.6364890626	-1.8950620610
H	-8.8466666621	0.2999745613	-1.9833762004
H	-0.7825177827	-0.6228899238	2.9389383228
H	-0.4538287696	1.5385222620	1.7533447471

H	-2.1671495250	2.3164565080	0.1562303745
N	-4.7623608907	-1.3327499946	0.8831189715
C	-3.8993621456	1.3771450363	-1.9532334080
C	-3.3951406029	0.3410181172	-2.7578256968
C	-3.8637892494	2.6754839034	-2.4456847982
C	-2.8834922995	0.6596538617	-4.0098789747
C	-3.3479936207	2.9722999057	-3.6998704762
C	-2.8473850666	1.9613627039	-4.5055293705
H	-2.4945522908	-0.1436783469	-4.6282084931
H	-3.3376141121	3.9989392420	-4.0483887429
C	-3.3907124106	-1.0956138936	-2.3152999502
H	-2.8267385497	-1.2282708221	-1.3903722330
H	-4.4032870366	-1.4599311664	-2.1319809262
H	-2.9389206586	-1.7291241275	-3.0786162872
H	-4.2509841145	3.4801212863	-1.8301078190
H	-4.8892223297	-2.1594894630	1.4420526364
H	-4.7865662369	2.1451438764	-0.2155779350
C	-2.2827265340	2.2425486002	-5.8695283461
H	-1.2456695813	1.9063339222	-5.9414966046
H	-2.8474915845	1.7167831665	-6.6432378542
H	-2.3097218782	3.3086683346	-6.0969949917

HCyA23

E(RwB97XD) = -1101.19389590

Charge = 0 Multiplicity = 1

C	-8.0597302530	-0.8462848004	-1.1288711661
C	-6.7143486467	-1.1618005778	-1.1245959228
C	-5.7900987407	-0.3045129128	-0.5250148854
C	-6.2155861540	0.8797378182	0.0819459129
C	-7.5746312886	1.1687558001	0.0695940917
C	-8.4986272089	0.3260141611	-0.5281143817
C	-5.2070949902	1.8746699259	0.6702117697
C	-3.8600583921	1.1956936499	0.9170458096
C	-3.5255769876	0.0053602515	0.2682210539
C	-2.2667252300	-0.5709385884	0.4527669481
H	-2.0312130795	-1.4964577850	-0.0606754461

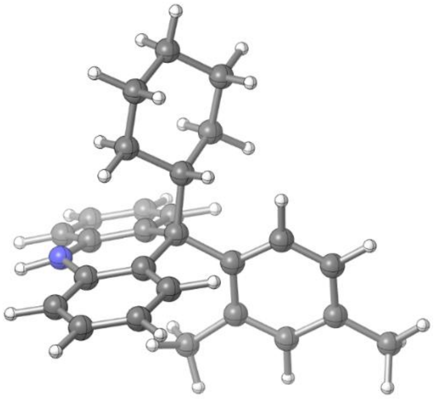

C	-1.3428273903	0.0209486890	1.2914691571
C	-1.6693434276	1.1909653569	1.9653020050
C	-2.9181549062	1.7574056954	1.7720371071
H	-8.7662540207	-1.5200412413	-1.5981474124
H	-6.3618553303	-2.0768122134	-1.5873537553
H	-7.9188782703	2.0820384414	0.5395229226
H	-9.5502972237	0.5825498267	-0.5241612865
H	-0.3714289621	-0.4385027566	1.4282551138
H	-0.9586945724	1.6574308745	2.6355169287
H	-3.1754420364	2.6627830999	2.3071272582
N	-4.4460743454	-0.6251815022	-0.5485576025
C	-5.7921843975	2.3981865146	2.0020326395
C	-5.9390437699	1.5499659761	3.1214033693
C	-6.2694231464	3.6991275189	2.1224721192
C	-6.5062952907	2.0591706712	4.2847899869
C	-6.8352561901	4.1842321474	3.2931767670
C	-6.9539286236	3.3703611176	4.4066999786
H	-6.6134697920	1.3934088444	5.1356172838
H	-7.1891672841	5.2085982420	3.3281105385
C	-5.5322598504	0.0986405440	3.1415756757
H	-5.8323901835	-0.4277784959	2.2361234979
H	-4.4509047572	-0.0146895015	3.2374979337
H	-5.9972288384	-0.4045402810	3.9897492766
H	-6.2179630388	4.3749487911	1.2822154154
H	-4.1981065069	-1.5343335358	-0.9025860439
C	-5.0096400450	3.0134562056	-0.4013673393
C	-3.9572180292	4.0713744973	-0.0434724845
C	-4.6849424293	2.4841909220	-1.8047305456
H	-5.9815281697	3.5117602836	-0.4860718805
C	-3.9744630462	5.2260876685	-1.0417837583
H	-2.9672488328	3.6076946855	-0.0672570945
H	-4.0954425803	4.4519250808	0.9682669553
C	-4.6741276275	3.6129386284	-2.8342053150
H	-3.7057015441	1.9934828587	-1.7911834577
H	-5.4147938692	1.7359632776	-2.1139099824
C	-3.7055642194	4.7279269779	-2.4572761314

H	-3.2297906450	5.9729516237	-0.7552599742
H	-4.9509057628	5.7230697329	-1.0078807234
H	-4.4225187772	3.2093298533	-3.8182619656
H	-5.6857732128	4.0271253704	-2.9135407975
H	-3.7754845682	5.5504255455	-3.1737234916
H	-2.6797766063	4.3459847069	-2.5099061784
C	-7.5380033607	3.8687050597	5.6978668776
H	-8.2773252771	3.1690716513	6.0932064300
H	-6.7606285096	3.9826305028	6.4580989886
H	-8.0202537625	4.8379581063	5.5658942115

X-Ray Crystallographic Data

N-(5-(Allylsulfonyl)pentyl)-4-chlorobenzamide (1d)

CCDC 2042726

Bond precision: C–C = 0.0021 Å Wavelength = 1.54184

Cell: a = 5.0188(1) b = 9.6241(2) c = 16.1198(4)

α = 89.487(2) β = 85.132(2) γ = 82.136(2)

Temperature: 100 K

	Calculated	Reported
Volume	768.50(3)	768.50(3)
Space group	P -1	P -1
Hall group	-P 1	-P 1
Moiety formula	$C_{15}H_{20}ClNO_3S$	$C_{15}H_{20}ClNO_3S$
Sum formula	$C_{15}H_{20}ClNO_3S$	$C_{15}H_{20}ClNO_3S$
M_r	329.83	329.83
D_x,g cm^{-3}	1.425	1.425
Z	2	2
Mu (mm^{-1})	3.554	3.554
F000	348.0	348.0
F000'	350.27	
h,k,l_{max}	6,12,20	6,12,20
N_{ref}	3246	3083
T_{min},T_{max}	0.603,0.805	0.601,1.000
T_{min}'	0.470	

Correction method = # Reported T Limits: T_{min} = 0.601 T_{max} = 1.000 AbsCorr = GAUSSIAN

Data completeness = 0.950 Theta(max) = 76.472

R(reflections) = 0.0347(2859) wR2(reflections) = 0.0883(3083)

S = 1.062 N_{par} = 199

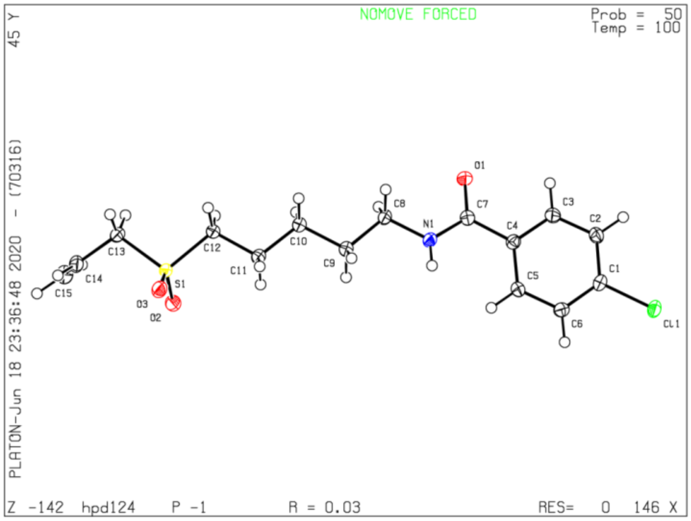

Go back to table of contents

1,4-Bis(allylsulfonyl)butane (1k)

CCDC 2042729

Bond precision:		C–C = 0.0020 Å		Wavelength = 1.54184
Cell:	a = 6.4691(1)	b = 11.4368(2)	c = 8.7387(1)	
	α = 90	β = 102.114(2)	γ = 90	
Temperature:	100 K			

	Calculated	Reported
Volume	632.143(17)	632.142(17)
Space group	P 21/n	P 1 21/n 1
Hall group	-P 2yn	-P 2yn
Moiety formula	$C_{10}H_{18}O_4S_2$	$C_{10}H_{18}O_4S_2$
Sum formula	$C_{10}H_{18}O_4S_2$	$C_{10}H_{18}O_4S_2$
M_r	266.36	266.36
D_x,g cm^{-3}	1.399	1.399
Z	2	2
Mu (mm^{-1})	3.818	3.818
F000	284.0	284.0
F000'	286.08	
h,k,l_{max}	8,14,11	8,14,11
N_{ref}	1321	1265
T_{min},T_{max}	0.540,0.660	0.732,1.000
T_{min}'	0.406	

Correction method = # Reported T Limits: T_{min} = 0.732 T_{max} = 1.000 AbsCorr = GAUSSIAN

Data completeness = 0.958 Theta(max) = 76.450

R(reflections) = 0.0290(1234) wR2(reflections) = 0.0777(1265)

S = 1.051 N_{par} = 74

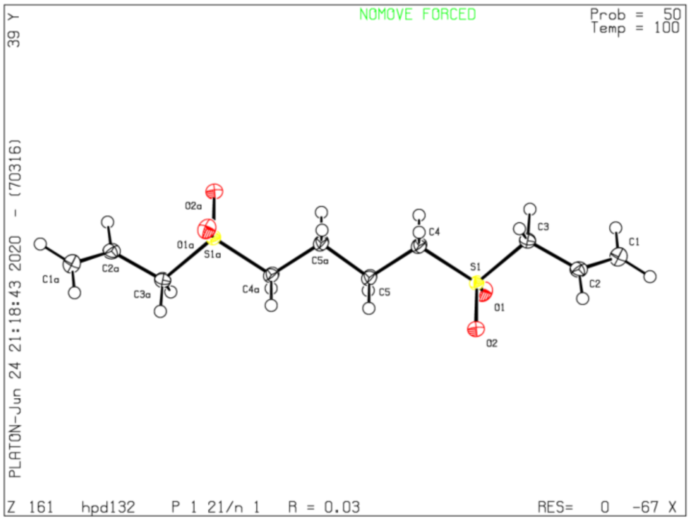

Go back to table of contents

tert-Butyl (*S*)-4-(allylsulfonyl)-2-((*tert*-butoxycarbonyl)amino)butanoate (1u)

CCDC 2042730

Bond precision:	C–C = 0.0033 Å		Wavelength = 1.54184
Cell:	a = 5.46991(9)	b = 10.15896(14)	c = 34.3303(5)
	α = 90	β = 90	γ = 90
Temperature:	100 K		

	Calculated	Reported
Volume	1907.69(5)	1907.69(5)
Space group	P 21 21 21	P 21 21 21
Hall group	P 2ac 2ab	P 2ac 2ab
Moiety formula	$C_{16}H_{29}NO_6S$	$C_{16}H_{29}NO_6S$
Sum formula	$C_{16}H_{29}NO_6S$	$C_{16}H_{29}NO_6S$
M_r	363.46	363.46
D_x,g cm^{-3}	1.265	1.265
Z	4	4
Mu (mm^{-1})	1.766	1.766
F000	784.0	784.0
F000'	787.71	
h,k,l_{max}	6,12,43	6,12,43
N_{ref}	4007[2355]	3907
T_{min},T_{max}	0.774,0.911	0.652,1.000
T_{min}'	0.653	

Correction method = # Reported T Limits: T_{min} = 0.652 T_{max} = 1.000 AbsCorr = GAUSSIAN

Data completeness = 1.66/0.98 Theta(max) = 76.533

R(reflections) = 0.0303(3787) wR2(reflections) = 0.0773(3907)

S = 1.025 N_{par} = 224

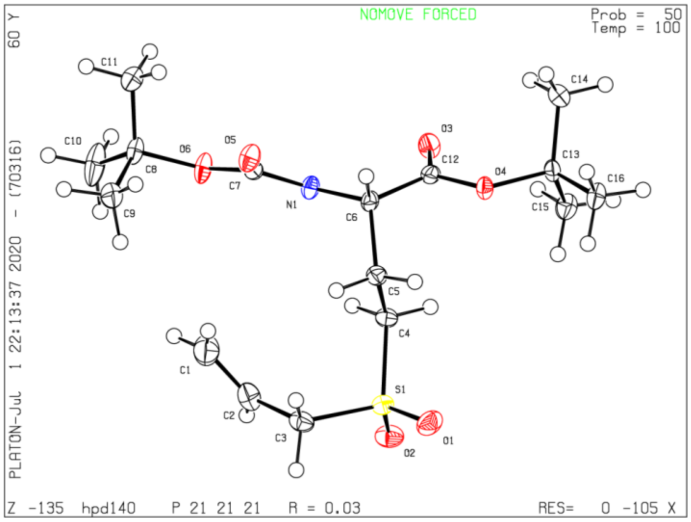

Go back to table of contents

((1*S*,2*S*,4a*R*,4b*R*,7*S*,9a*S*,10*S*,10a*R*)-10-(Allylsulfonyl)-1-methyl-8-methylene-13-oxo-1,2,5,6,8,9,10,10a-octahydro-4a,1-(epoxymethano)-7,9a-methanobenzo[*a*]azulene-2,7(4b*H*)-diyl diacetate (1z)

CCDC 2042731

Bond precision:	C–C = 0.0033 Å		Wavelength = 1.54184
Cell:	a = 10.04584(4)	b = 19.87397(12)	c = 12.32816(6)
	α = 90	β = 95.9688(4)	γ = 90
Temperature:	100 K		

	Calculated	Reported
Volume	2447.98(2)	2447.98(2)
Space group	P 21	P 1 21 1
Hall group	P 2yb	P 2yb
Moiety formula	$C_{25}H_{30}O_8S$	$C_{25}H_{30}O_8S$
Sum formula	$C_{25}H_{30}O_8S$	$C_{25}H_{30}O_8S$
M_r	490.55	490.55
D_x,g cm^{-3}	1.331	1.331
Z	4	4
Mu (mm^{-1})	1.580	1.580
F000	1040.0	1040.0
F000'	1044.62	
h,k,l_{max}	12,25,15	12,24,15
N_{ref}	10287[5298]	9458
T_{min},T_{max}	0.786,0.883	0.555,1.000
T_{min}'	0.766	

Correction method = # Reported T Limits: T_{min} = 0.555 T_{max} = 1.000 AbsCorr = GAUSSIAN

Data completeness = 1.79/0.92 Theta(max) = 76.528

R(reflections) = 0.0293(9233) wR2(reflections) = 0.0779(9458)

S = 1.045 N_{par} = 620

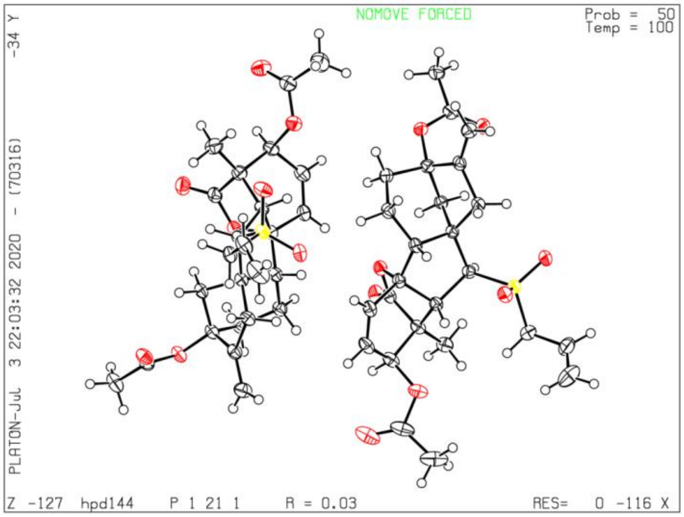

Go back to table of contents

1-Methyl-4-(((4-phenylbutyl)sulfonyl)methyl)benzene (2d)

CCDC 2042732

Bond precision:	C–C = 0.0040 Å		Wavelength = 1.54184
Cell:	a = 9.3374(1)	b = 6.0661(1)	c = 28.5106(4)
	α = 90	β = 90	γ = 90
Temperature:	100 K		

	Calculated	Reported
Volume	1614.89(4)	1614.89(4)
Space group	P n a 21	P n a 21
Hall group	P 2c -2n	P 2c -2n
Moiety formula	$C_{18}H_{22}O_2S$	$C_{18}H_{22}O_2S$
Sum formula	$C_{18}H_{22}O_2S$	$C_{18}H_{22}O_2S$
M_r	302.42	302.41
D_x,g cm^{-3}	1.244	1.244
Z	4	4
Mu (mm^{-1})	1.786	1.786
F000	648.0	648.0
F000'	650.97	
h,k,l_{max}	11,7,35	11,7,35
N_{ref}	3395[1732]	2879
T_{min},T_{max}	0.795,0.905	0.767,1.000
T_{min}'	0.742	

Correction method = # Reported T Limits: T_{min} = 0.767 T_{max} = 1.000 AbsCorr = GAUSSIAN

Data completeness = 1.66/0.85 Theta(max) = 76.594

R(reflections) = 0.0313(2804) wR2(reflections) = 0.0807(2879)

S = 1.043 N_{par} = 191

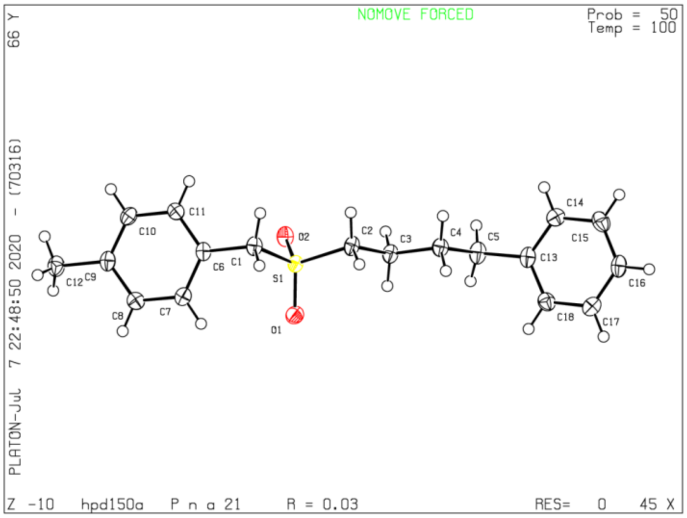

Go back to table of contents

4-(Methylsulfonyl)-1-phenylbutan-1-one (2e)

CCDC 2042733

Bond precision:	C−C = 0.0018 Å		Wavelength = 1.54184
Cell:	a = 5.72076(11)	b = 8.13039(16)	c = 11.8424(2)
	α = 91.8184(16)	β = 91.4466(15)	γ = 103.4125(17)
Temperature:	100 K		

	Calculated	Reported
Volume	535.220(18)	535.218(18)
Space group	P -1	P -1
Hall group	-P 1	-P 1
Moiety formula	$C_{11}H_{14}O_3S$	$C_{11}H_{14}O_3S$
Sum formula	$C_{11}H_{14}O_3S$	$C_{11}H_{14}O_3S$
M_r	226.28	226.28
D_x,g cm^{-3}	1.404	1.404
Z	2	2
Mu (mm^{-1})	2.571	2.571
F000	240.0	240.0
F000'	241.34	
h,k,l_{max}	7,10,14	7,10,14
N_{ref}	2243	2143
T_{min},T_{max}	0.673,0.710	0.354,1.000
T_{min}'	0.596	

Correction method = # Reported T Limits: T_{min} = 0.354 T_{max} = 1.000 AbsCorr = GAUSSIAN

Data completeness = 0.955 Theta(max) = 76.351

R(reflections) = 0.0287(2088) wR2(reflections) = 0.0780(2143)

S = 1.038 N_{par} = 138

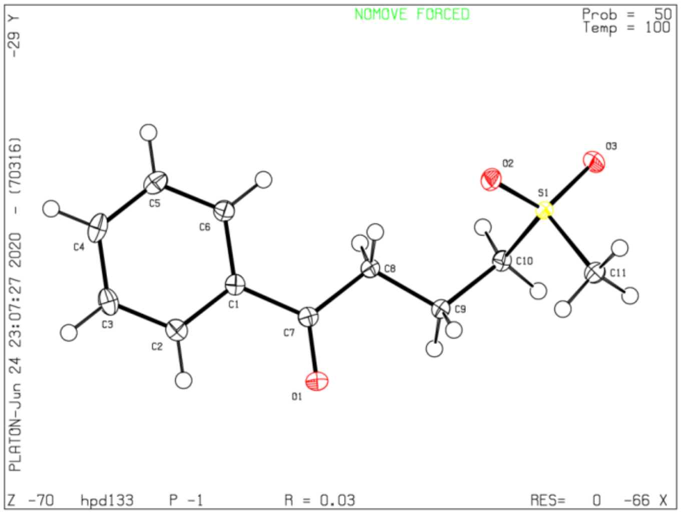

Go back to table of contents

Methyl (*tert*-butoxycarbonyl)(cyclohexylsulfonyl)-D-alaninate (2g)

CCDC 2042734

Bond precision:	C–C = 0.0024 Å		Wavelength = 1.54184
Cell:	a = 11.1742(3)	b = 16.6182(3)	c = 10.3665(3)
	α = 90	β = 116.394(3)	γ = 90
Temperature:	100 K		

	Calculated	Reported
Volume	1724.34(9)	1724.34(9)
Space group	P 21/c	P 1 21/c 1
Hall group	-P 2ybc	-P 2ybc
Moiety formula	$C_{15}H_{27}NO_6S$	$C_{15}H_{27}NO_6S$
Sum formula	$C_{15}H_{27}NO_6S$	$C_{15}H_{27}NO_6S$
M_r	349.44	349.43
D_x,g cm^{-3}	1.346	1.346
Z	4	4
Mu (mm^{-1})	1.933	1.933
F000	752.0	752.0
F000'	755.64	
h,k,l_{Max}	14,20,13	14,20,13
N_{ref}	3620	3515
T_{min},T_{max}	0.614,0.920	0.394,1.000
T_{min}'	0.474	

Correction method = # Reported T Limits: T_{min} = 0.394 T_{max} = 1.000 AbsCorr = GAUSSIAN

Data completeness = 0.971 Theta(max) = 76.519

R(reflections) = 0.0386(3130) wR2(reflections) = 0.1057(3515)

S = 1.075 N_{par} = 213

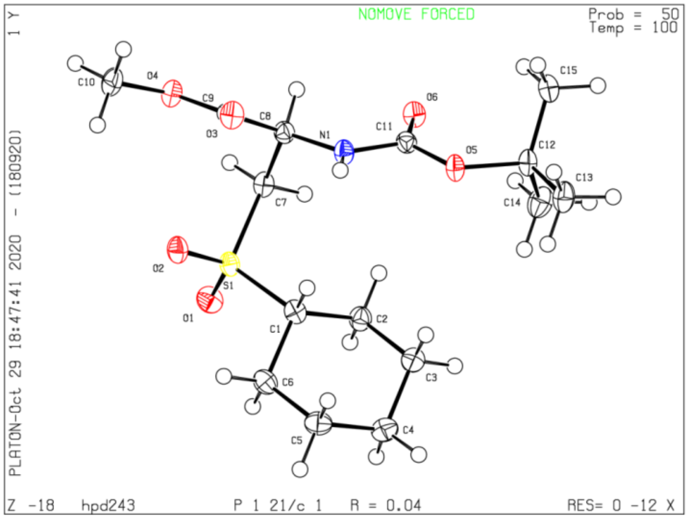

Go back to table of contents

3-((Cyclohexylsulfonyl)methyl)-1,2-oxathiolane 2-oxide (2h)

CCDC 2042735

Bond precision:	C–C = 0.0019 Å		Wavelength = 1.54184
Cell:	a = 6.09765(6)	b = 8.92648(10)	c = 22.0926(2)
	α = 90	β = 91.4743(9)	γ = 90
Temperature:	100 K		

	Calculated	Reported
Volume	1202.11(2)	1202.12(2)
Space group	P 21/c	P 1 21/c 1
Hall group	-P 2ybc	-P 2ybc
Moiety formula	$C_{10}H_{18}O_4S_2$	$C_{10}H_{18}O_4S_2$
Sum formula	$C_{10}H_{18}O_4S_2$	$C_{10}H_{18}O_4S_2$
M_r	266.36	266.36
D_x,g cm^{-3}	1.472	1.472
Z	4	4
Mu (mm^{-1})	4.015	4.015
F000	568.0	568.0
F000'	572.15	
h,k,l_{max}	7,11,27	7,11,27
N_{ref}	2529	2447
T_{min},T_{max}	0.465,0.786	0.454,1.000
T_{min}'	0.397	

Correction method = # Reported T Limits: T_{min} = 0.454 T_{max} = 1.000 AbsCorr = GAUSSIAN

Data completeness = 0.968 Theta(max) = 76.571

R(reflections) = 0.0281(2341) wR2(reflections) = 0.0761(2447)

S = 1.062 N_{par} = 146

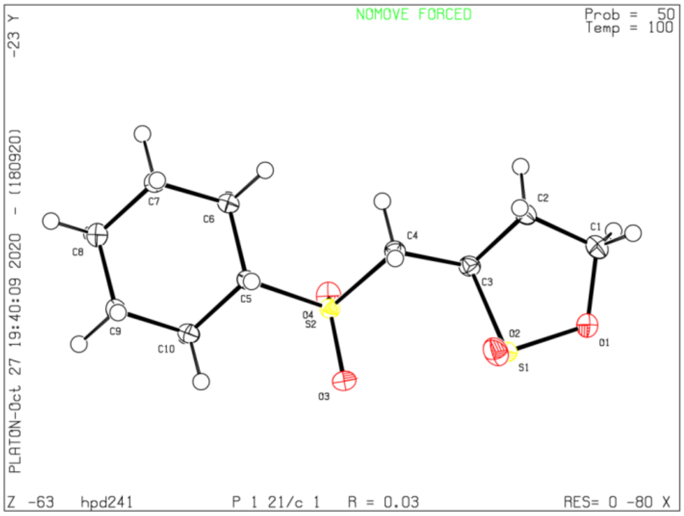

Go back to table of contents

1-(Cyclohexylsulfonyl)isoquinoline (2q)

CCDC 2042736

Bond precision:	C–C = 0.0020 Å		Wavelength = 1.54184
Cell:	a = 9.06120(12)	b = 15.82137(16)	c = 10.26220(14)
	α = 90	β = 114.8370(16)	γ = 90
Temperature:	100 K		

	Calculated	Reported
Volume	1335.12(3)	1335.12(3)
Space group	P 21/c	P 1 21/c 1
Hall group	-P 2ybc	-P 2ybc
Moiety formula	$C_{15}H_{17}NO_2S$	$C_{15}H_{17}NO_2S$
Sum formula	$C_{15}H_{17}NO_2S$	$C_{15}H_{17}NO_2S$
M_r	275.36	275.35
D_x,g cm^{-3}	1.370	1.370
Z	4	4
Mu (mm^{-1})	2.130	2.130
F000	584.0	584.0
F000'	586.87	
h,k,l_{max}	11,19,12	11,19,12
N_{ref}	2789	2694
T_{min},T_{max}	0.767,0.819	0.342,1.000
T_{min}'	0.529	

Correction method = # Reported T Limits: T_{min} = 0.342 T_{max} = 1.000 AbsCorr = GAUSSIAN

Data completeness = 0.966 Theta(max) = 76.259

R(reflections) = 0.0309(2515) wR2(reflections) = 0.0846(2694)

S = 1.076 N_{par} = 173

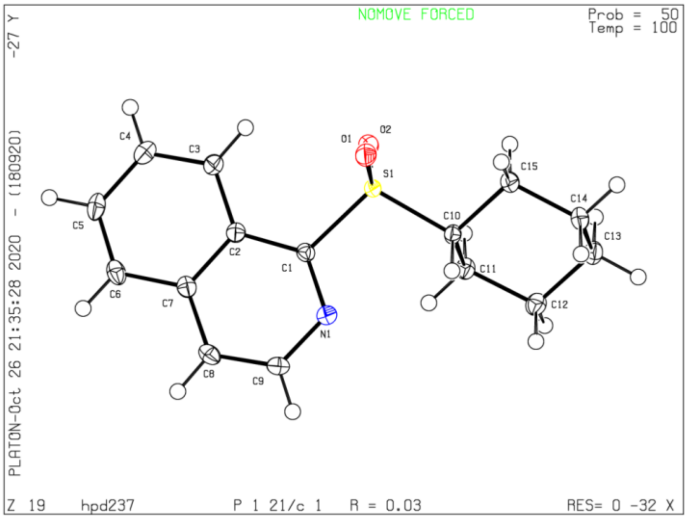

4-Oxo-4-phenylbutane-1-sulfonyl fluoride (5a)

CCDC 2042737

Bond precision: C–C = 0.0040 Å Wavelength = 1.54184

Cell: a = 5.7165(1) b = 12.1828(2) c = 14.7356(2)

α = 90 β = 91.722(1) γ = 90

Temperature: 100 K

	Calculated	Reported
Volume	1025.77(3)	1025.77(3)
Space group	P c	P 1 c 1
Hall group	P -2yc	P -2yc
Moiety formula	$C_{10}H_{11}FO_3S$	$C_{10}H_{11}FO_3S$
Sum formula	$C_{10}H_{11}FO_3S$	$C_{10}H_{11}FO_3S$
M_r	230.25	230.25
D_x,g cm^{-3}	1.491	1.491
Z	4	4
Mu (mm^{-1})	2.841	2.841
F000	480.0	480.0
F000'	482.90	
h,k,l_{max}	7,15,18	7,15,18
N_{ref}	4307[2161]	3241
T_{min},T_{max}	0.749,0.880	0.763,1.000
T_{min}'	0.594	

Correction method = # Reported T Limits: T_{min} = 0.763 T_{max} = 1.000 AbsCorr = GAUSSIAN

Data completeness = 1.50/0.75 Theta(max) = 76.622

R(reflections) = 0.0303(3185) wR2(reflections) = 0.0802(3241)

S = 1.021 N_{par} = 271

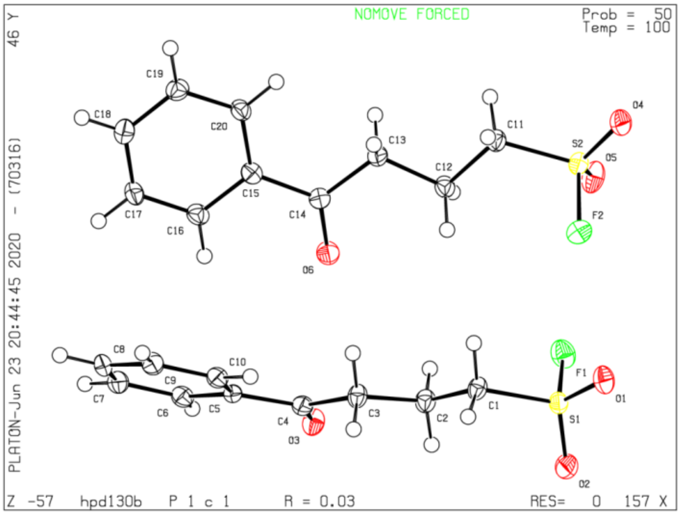

Go back to table of contents

NMR Spectroscopic Data

9-(3-(Trifluoromethyl)phenyl)acridine (A19)

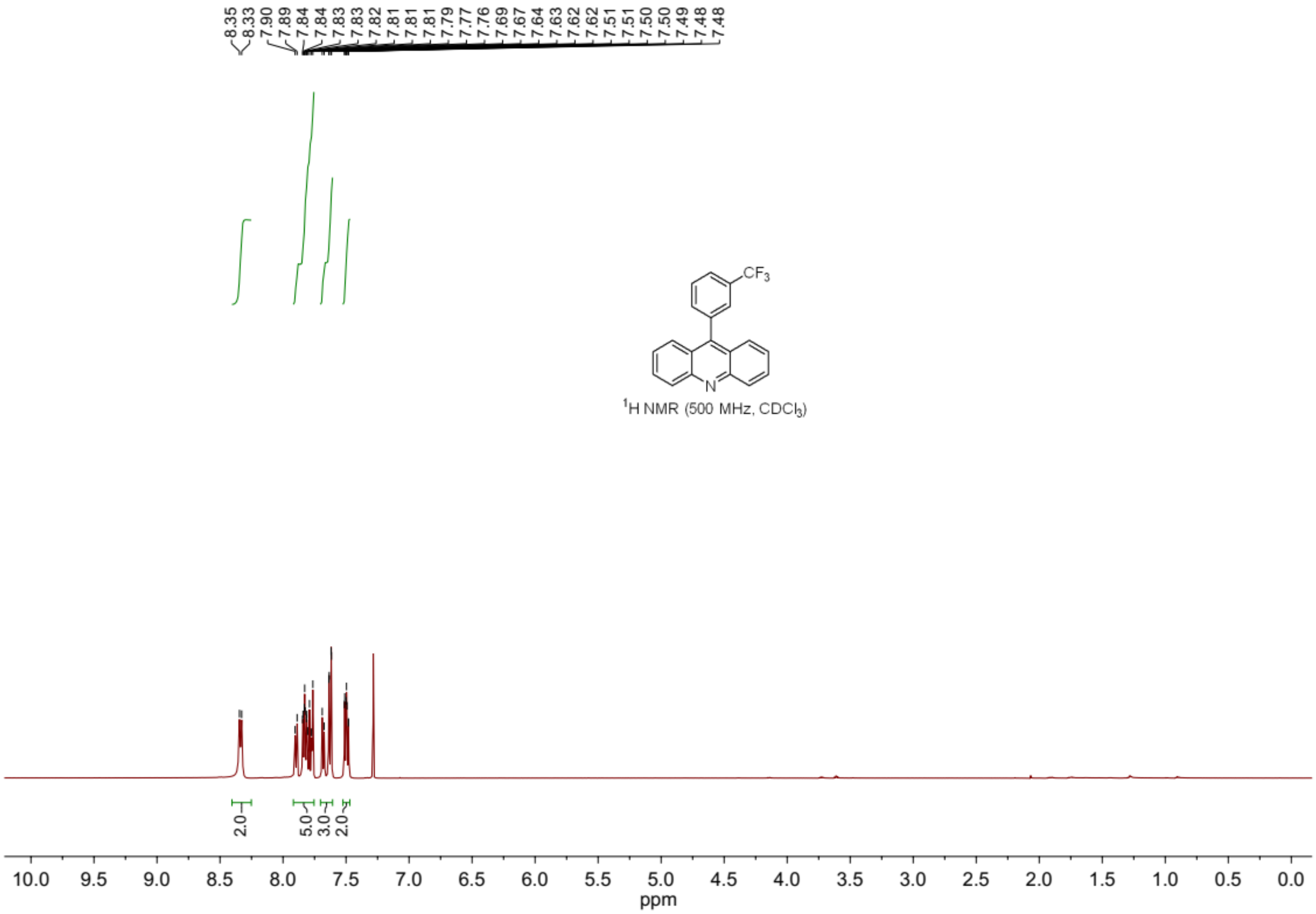

Go back to table of contents

9-(3-(Trifluoromethyl)phenyl)acridine (A19)

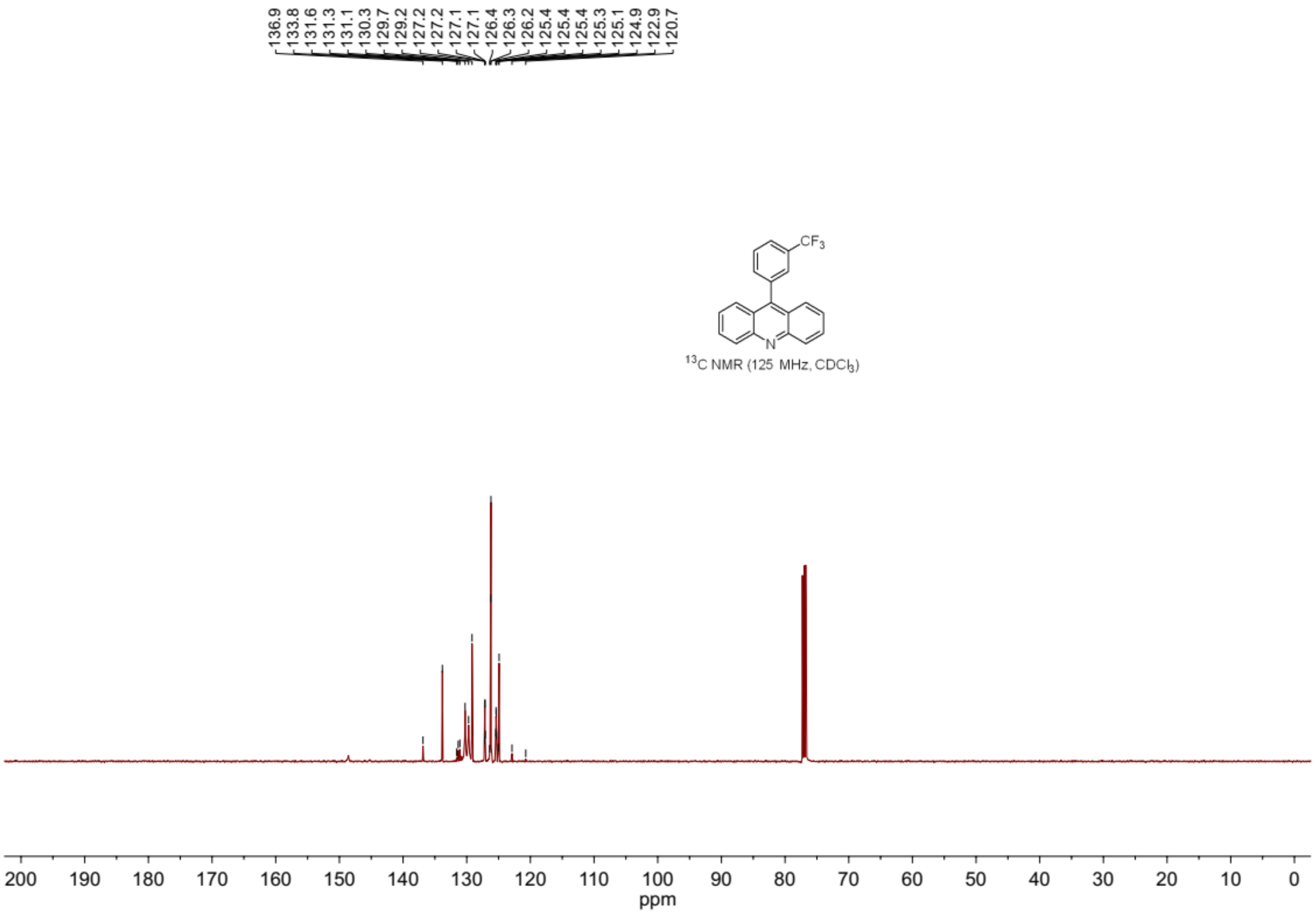

Go back to table of contents

9-(3-(Trifluoromethoxy)phenyl)acridine (A21)

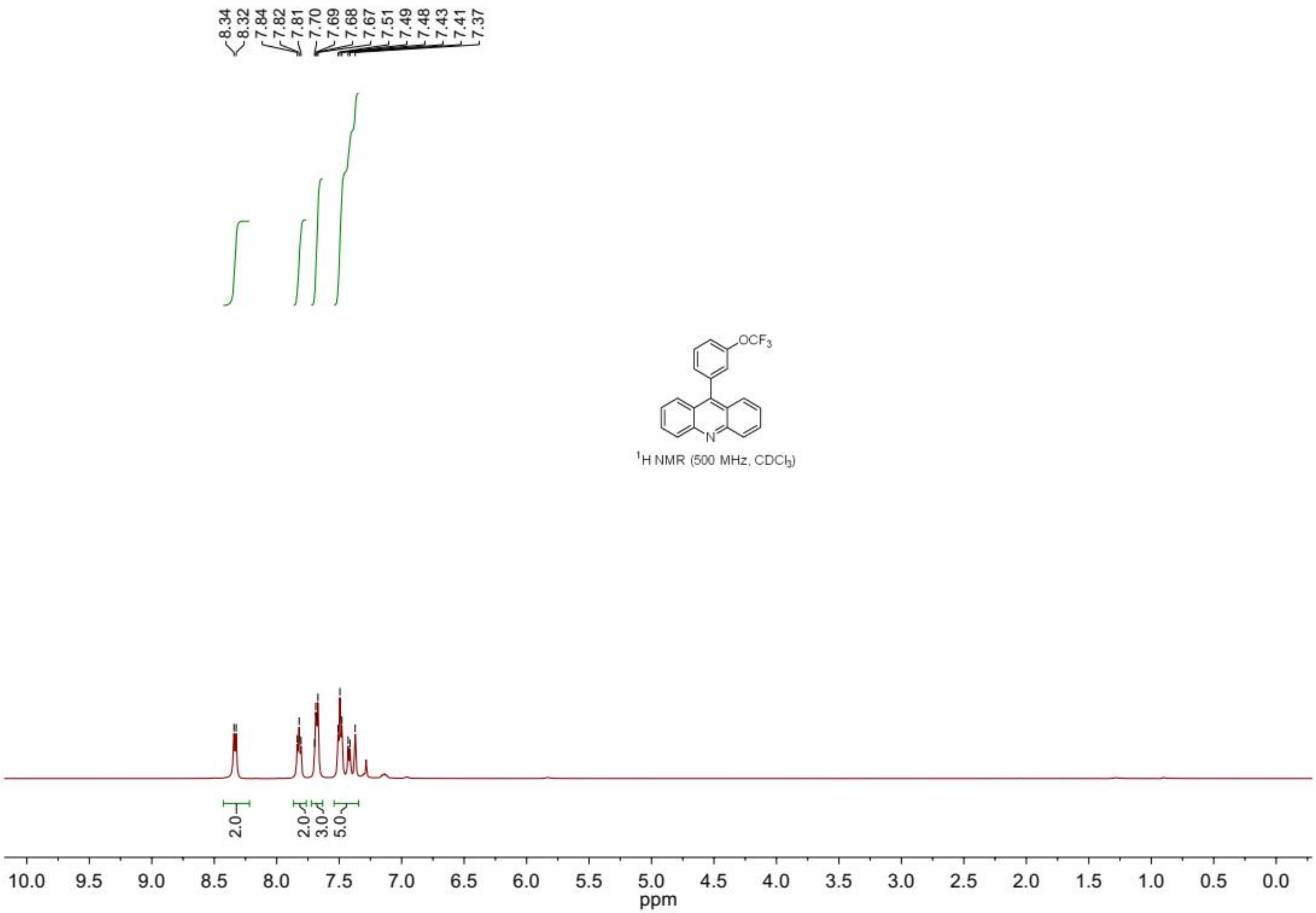

Go back to table of contents

9-(3-(Trifluoromethoxy)phenyl)acridine (A21)

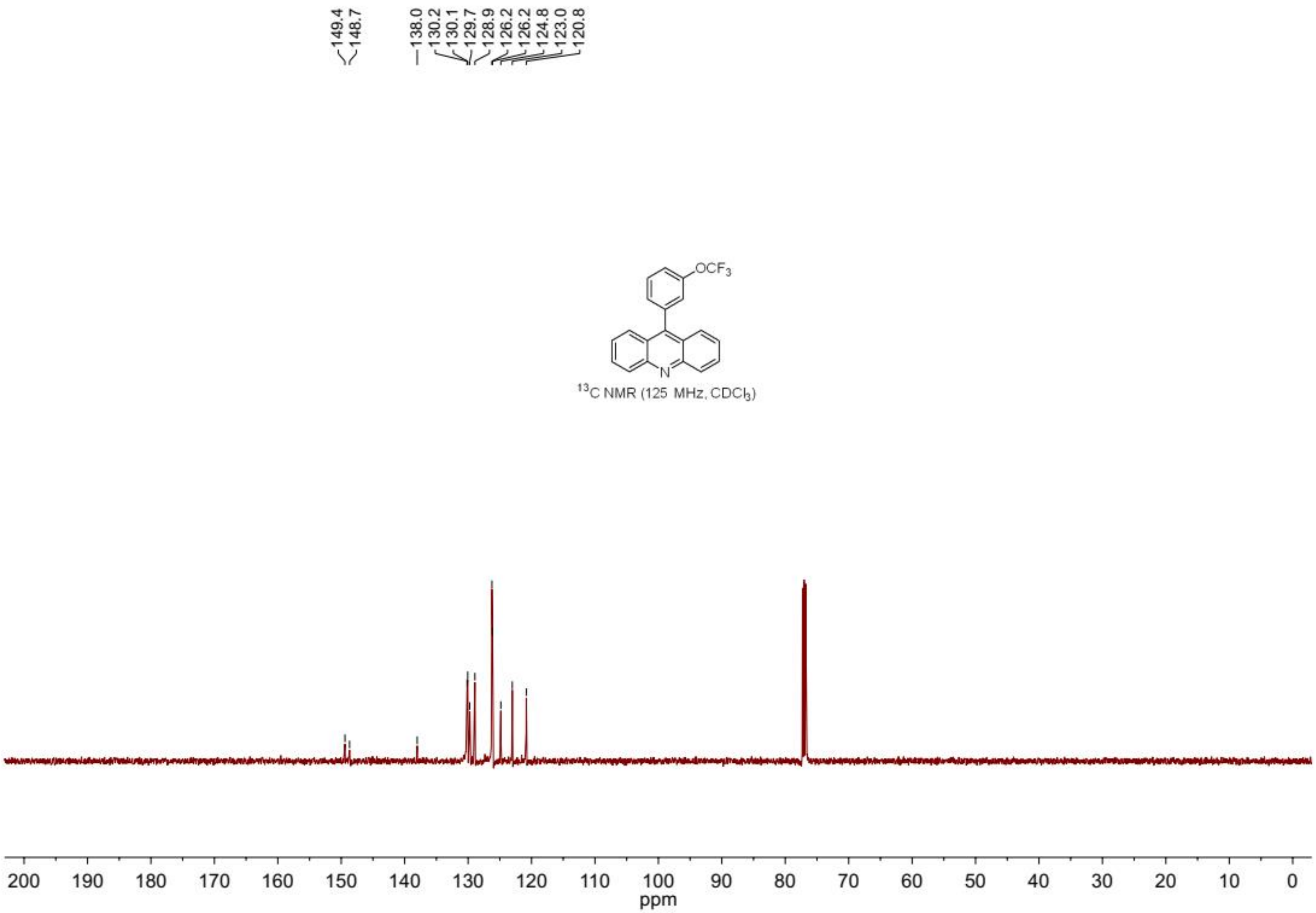

Go back to table of contents

(Allylsulfonyl)cyclohexane (1a)

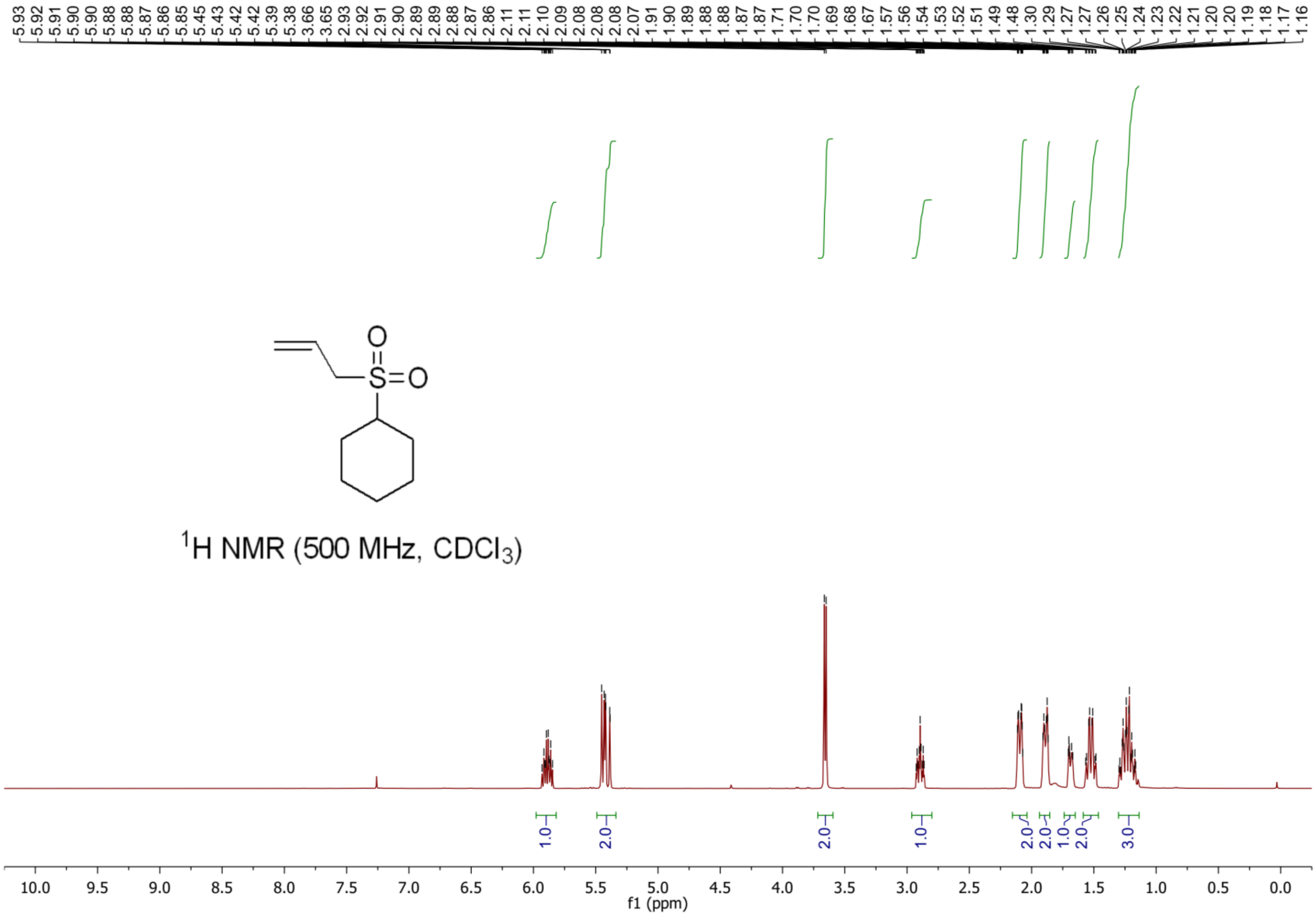

Go back to table of contents

(Allylsulfonyl)cyclohexane (1a)

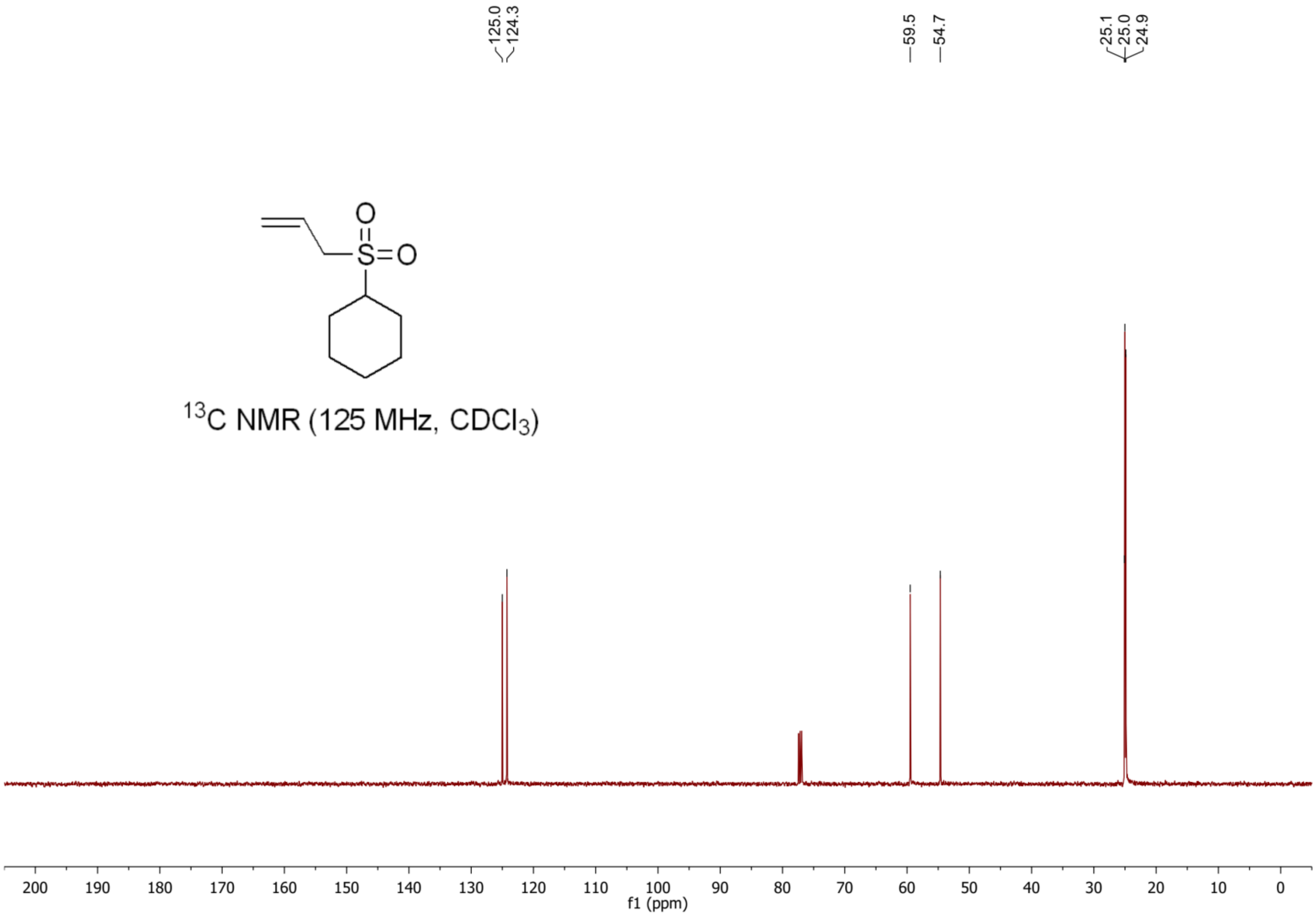

Go back to table of contents

1-(2-(Allylsulfonyl)ethyl)-4-methoxybenzene (1b)

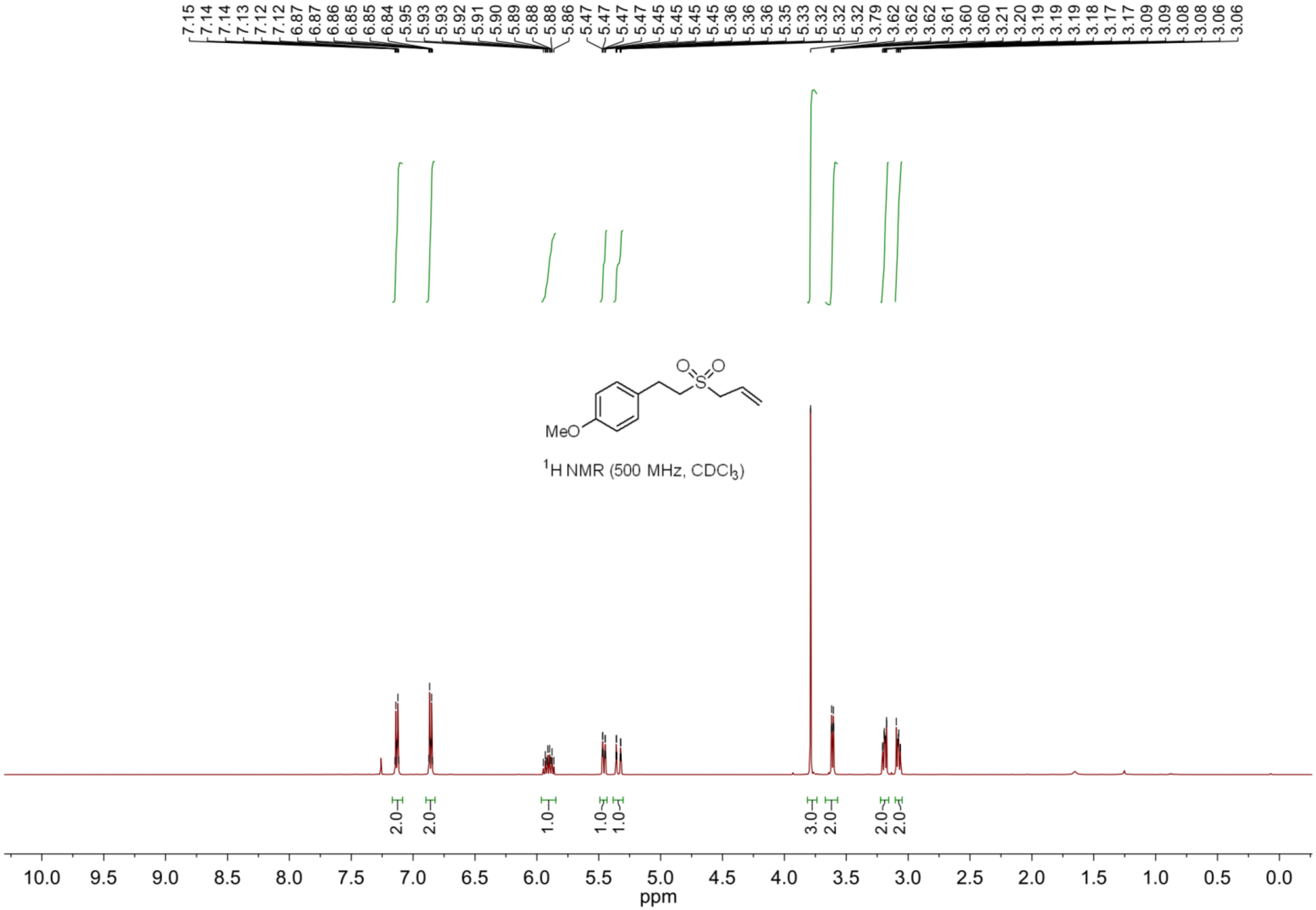

Go back to table of contents

1-(2-(Allylsulfonyl)ethyl)-4-methoxybenzene (1b)

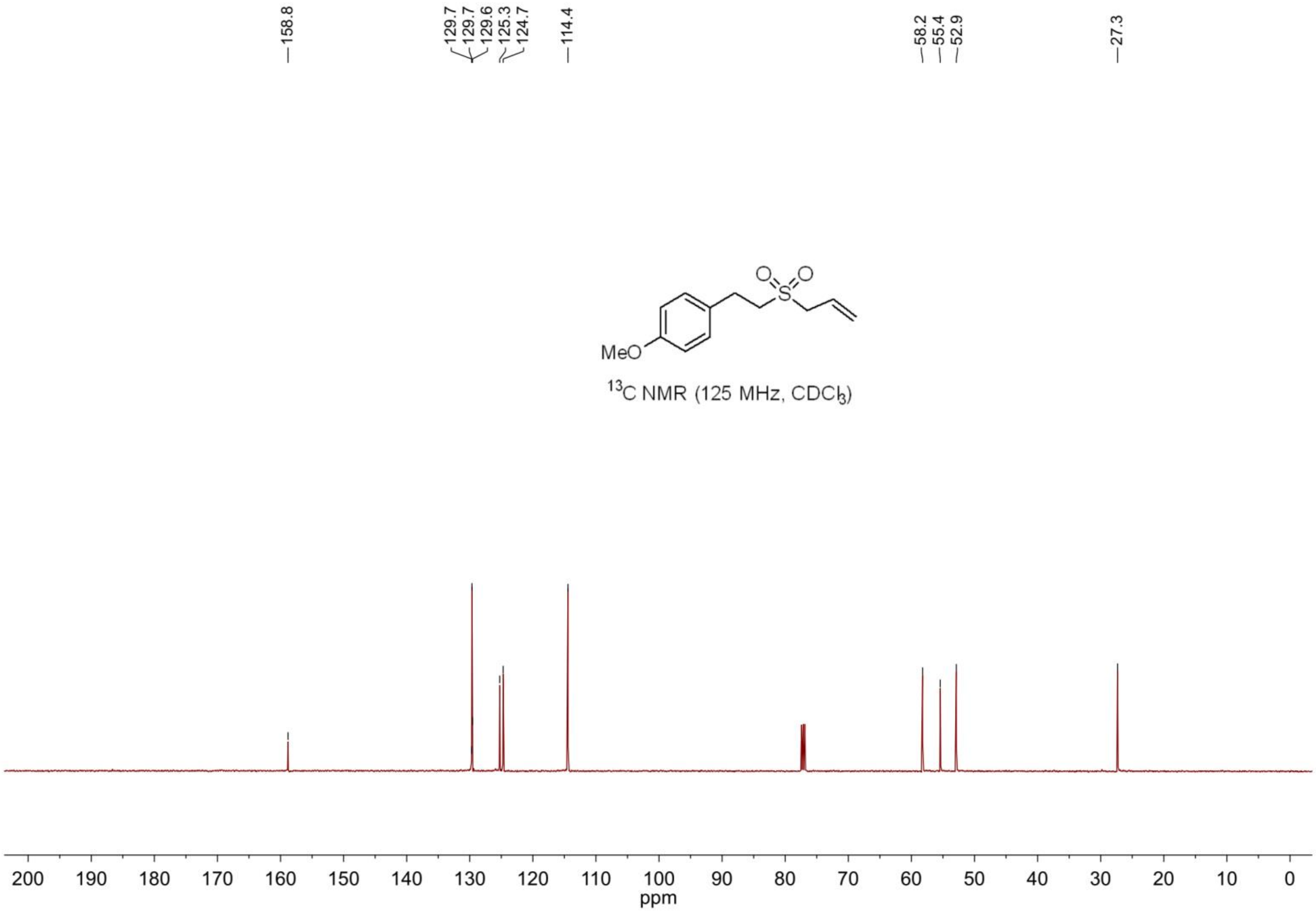

Go back to table of contents

Methyl 5-(allylsulfonyl)pentanoate (1c)

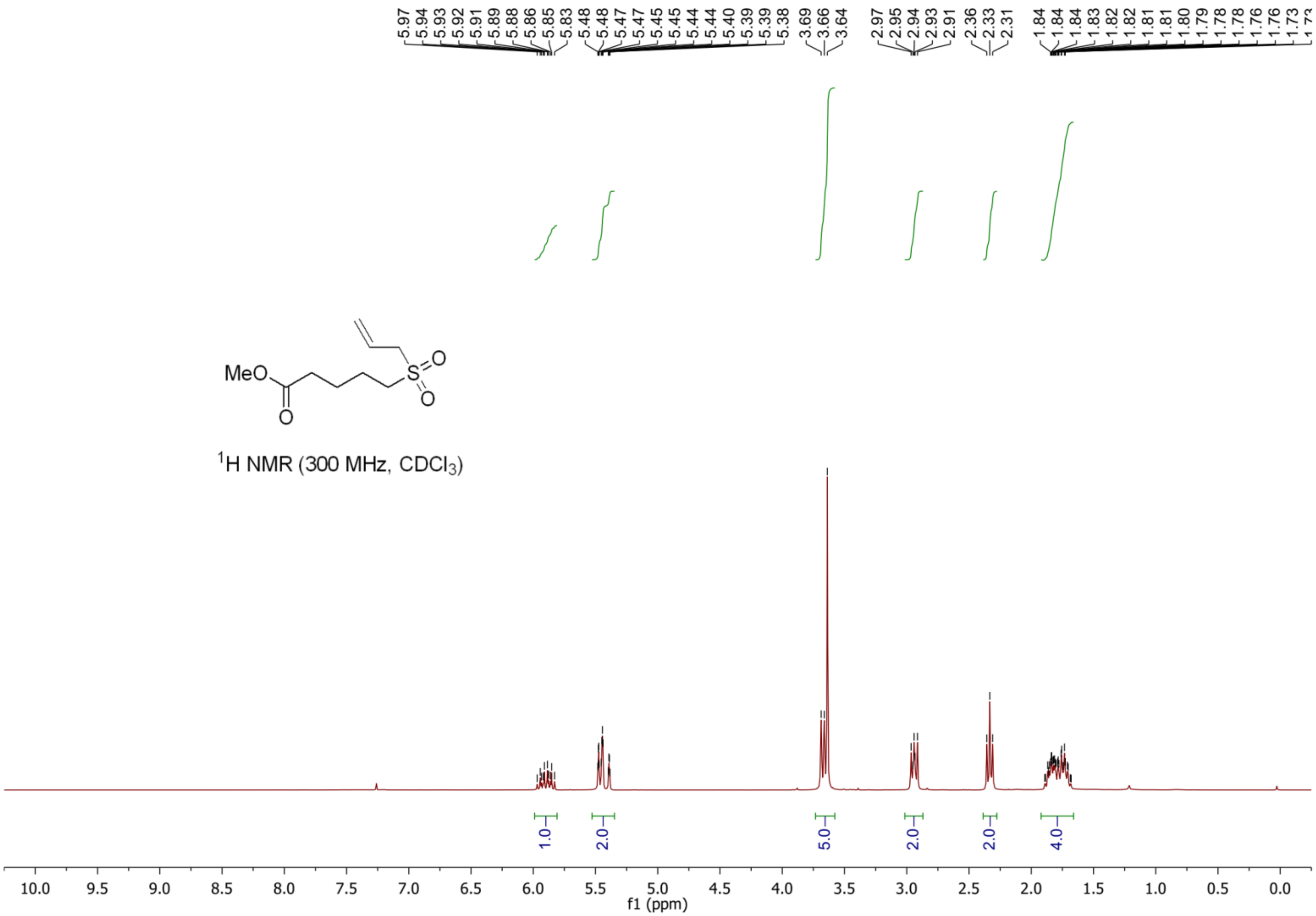

^{1}H NMR (300 MHz, $CDCl_3$)

Go back to table of contents

Methyl 5-(allylsulfonyl)pentanoate (1c)

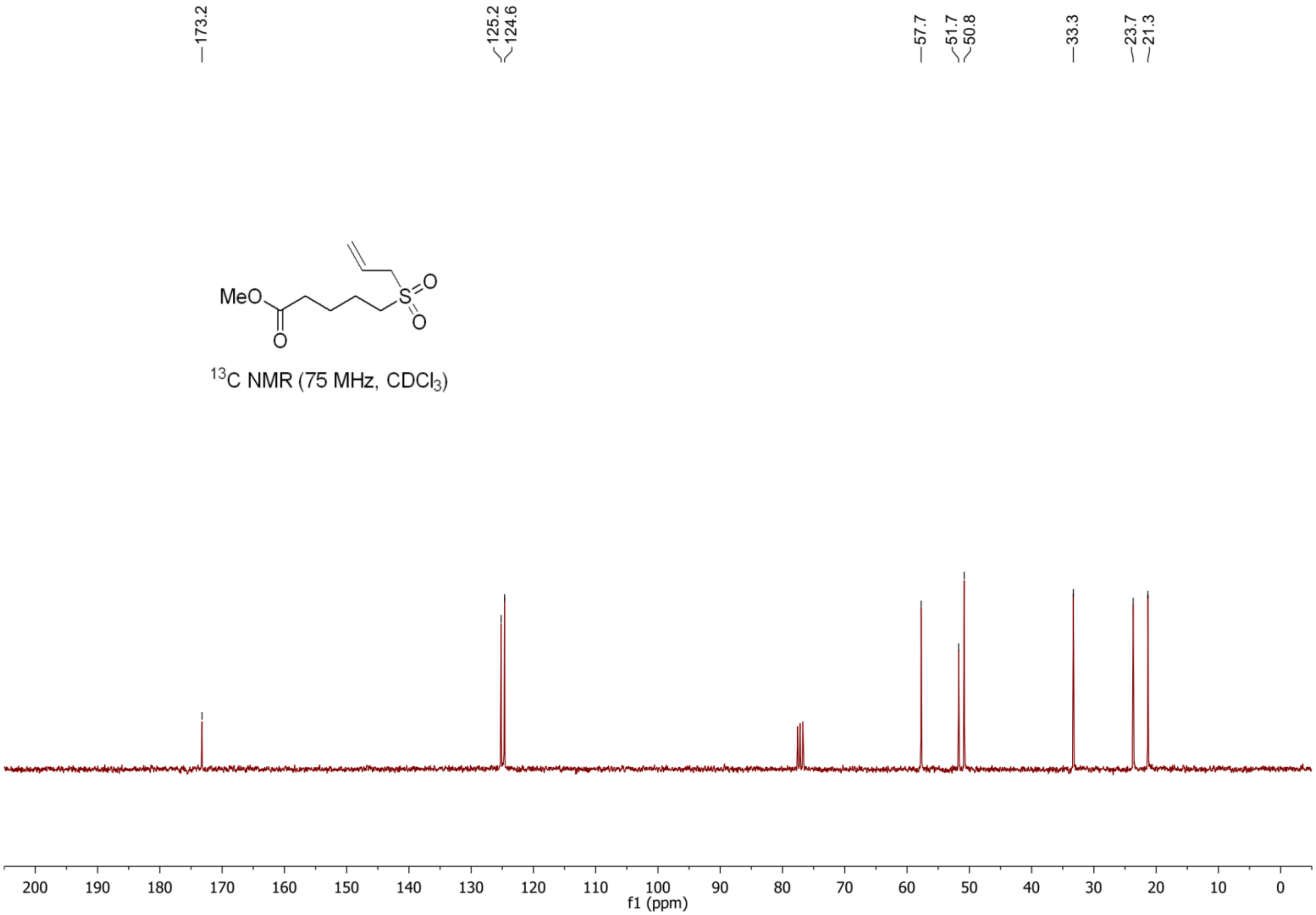

Go back to table of contents

***N*-(5-(Allylsulfonyl)pentyl)-4-chlorobenzamide (1d)**

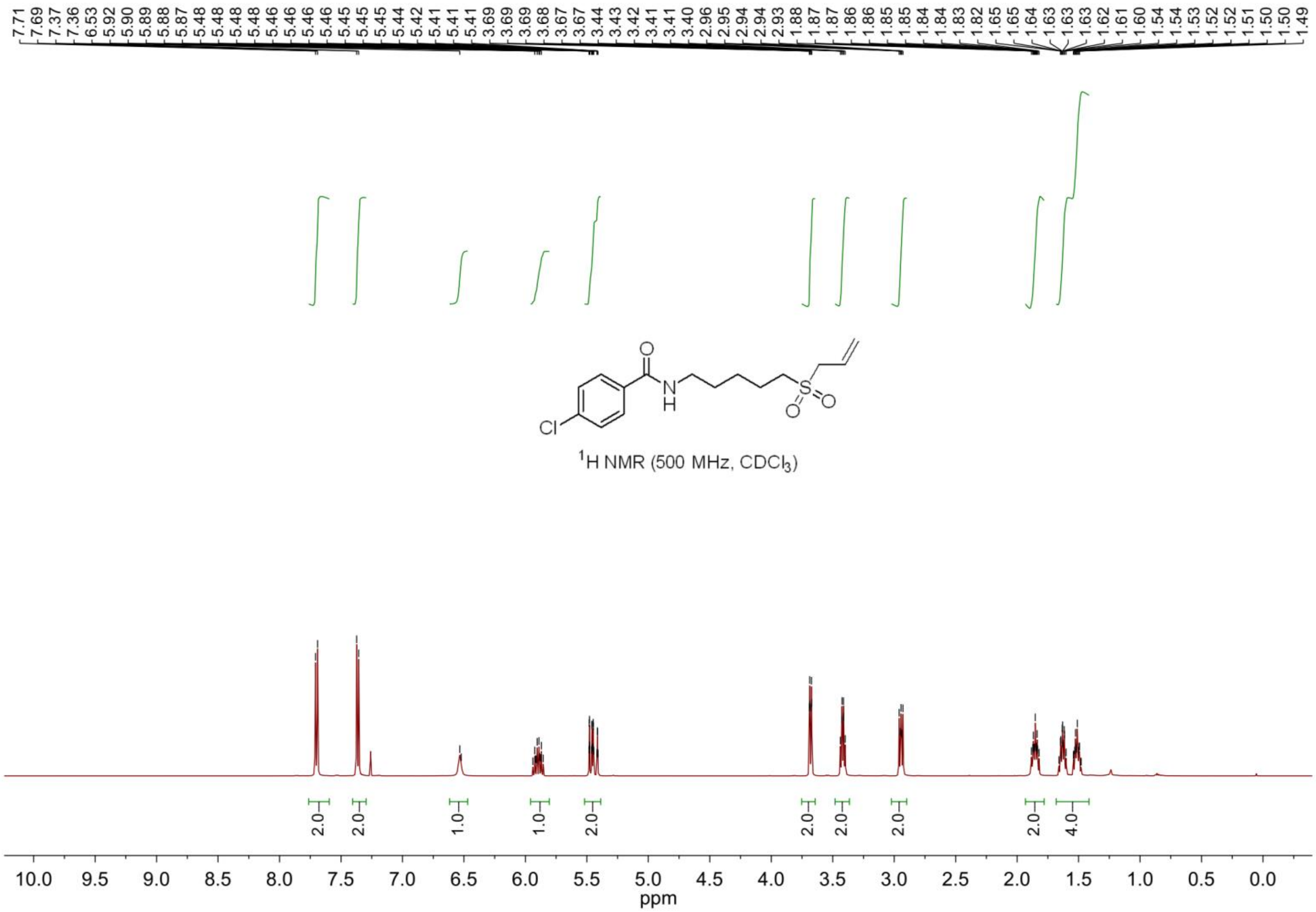

Go back to table of contents

N-(5-(Allylsulfonyl)pentyl)-4-chlorobenzamide (1d)

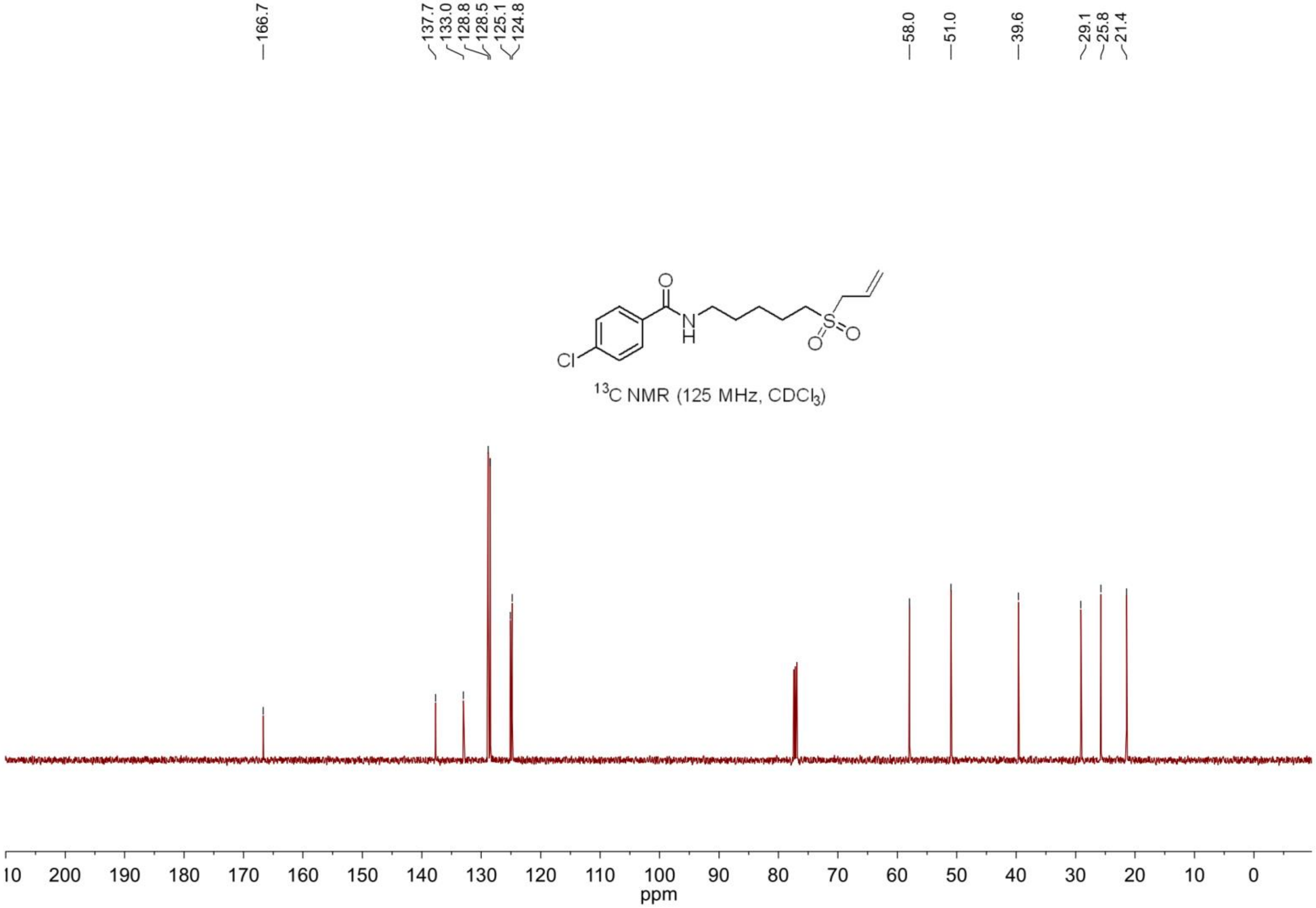

Go back to table of contents

3-(3,3,3-Trifluoropropyl)sulfonyl)prop-1-ene (1e)

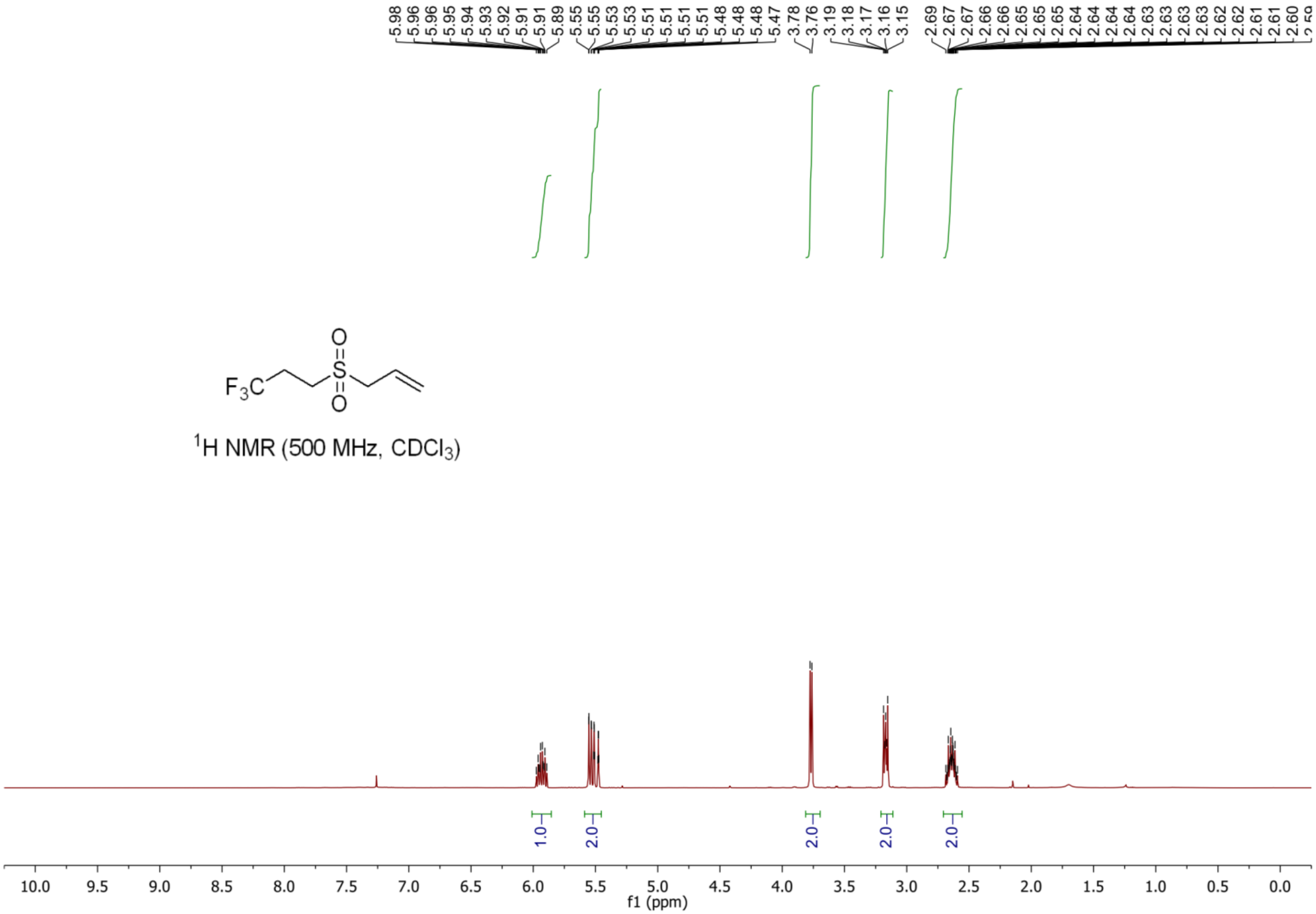

Go back to table of contents

3-(3,3,3-Trifluoropropyl)sulfonyl)prop-1-ene (1e)

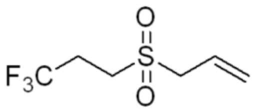

^{13}C NMR (125 MHz, $CDCl_3$)

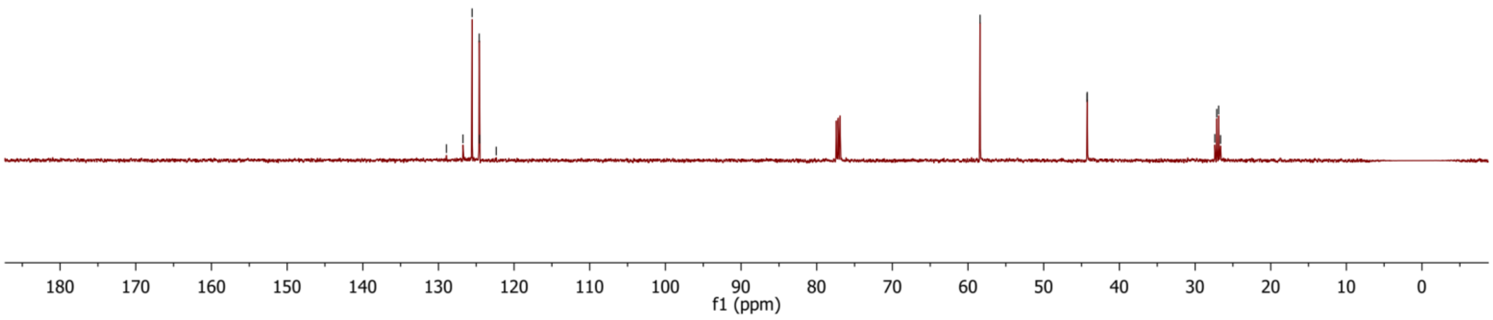

N-(5-(Allylsulfonyl)pentyl)-4-(trifluoromethyl)benzamide (1f)

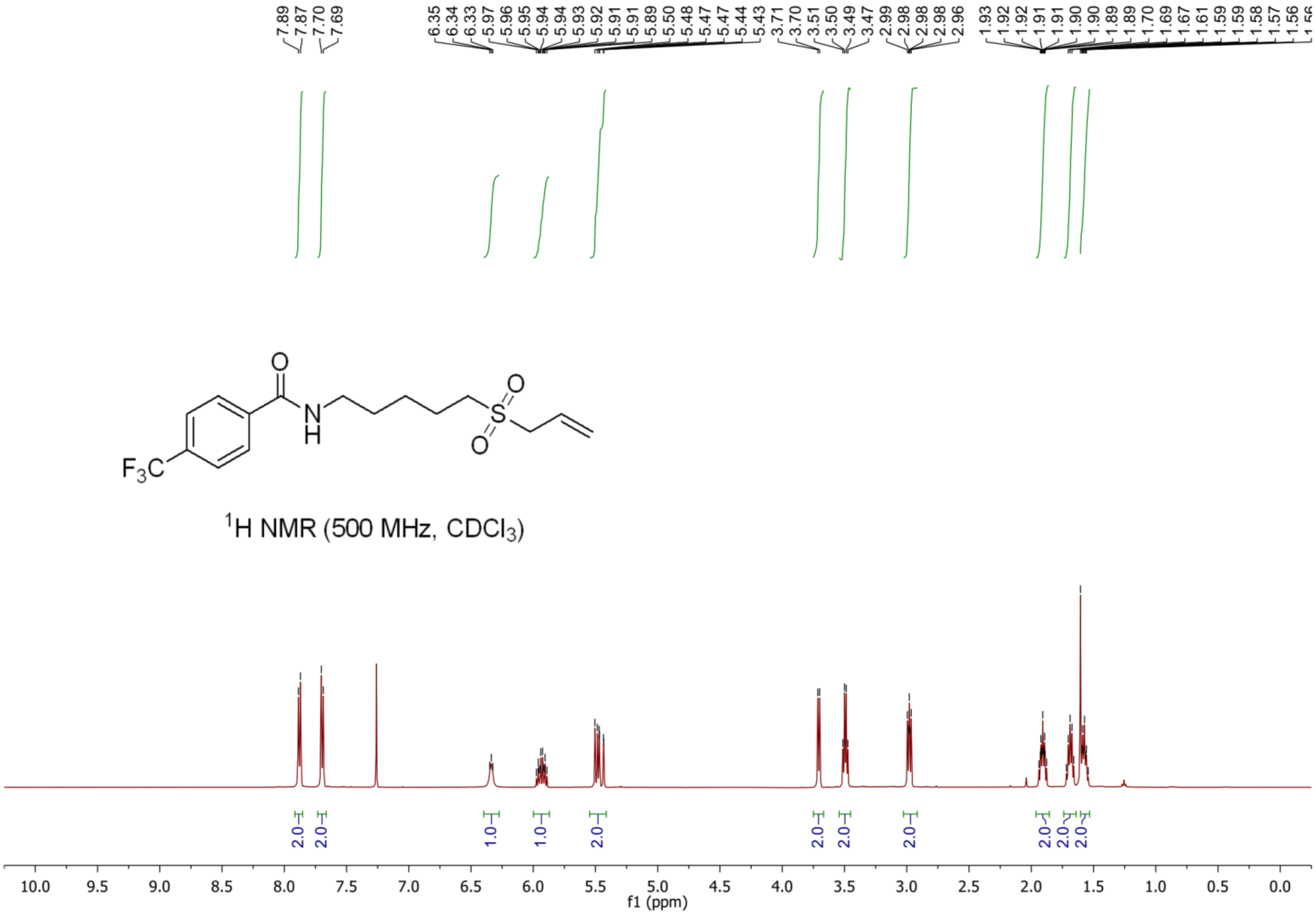

Go back to table of contents

_N_-(5-(Allylsulfonyl)pentyl)-4-(trifluoromethyl)benzamide (1f)

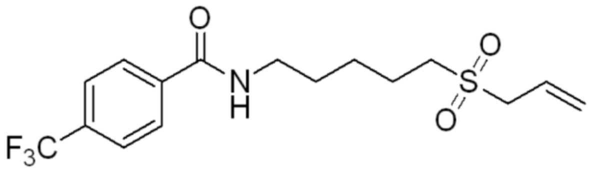

^{13}C NMR (125 MHz, $CDCl_3$)

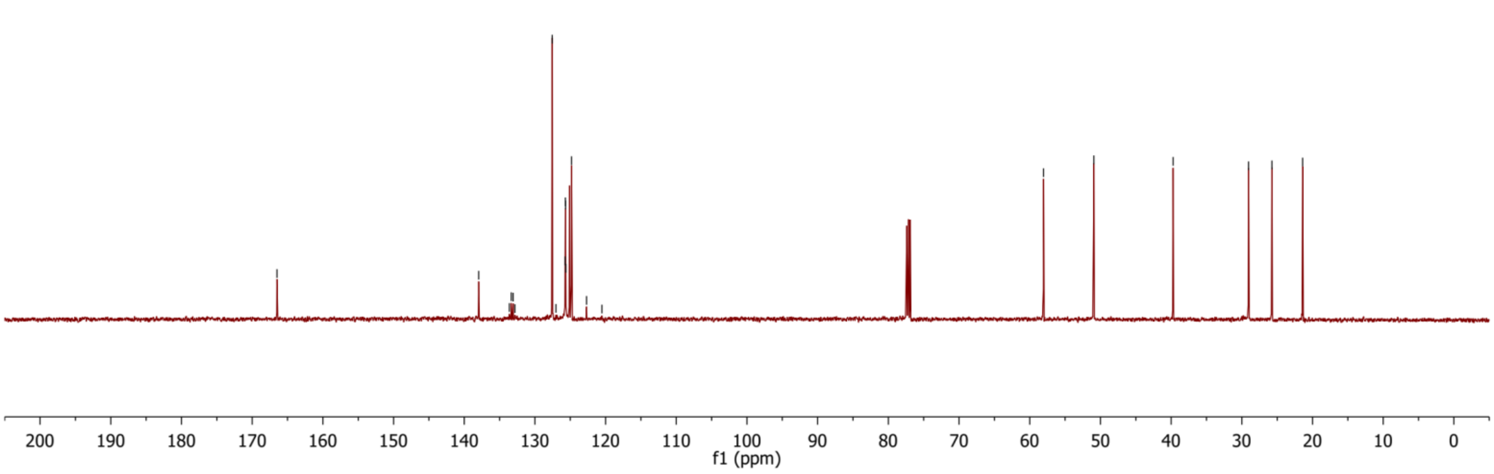

Go back to table of contents

4-(Allylsulfonyl)-*N*-(3-(4,4,5,5-tetramethyl-1,3,2-dioxaborolan-2-yl)phenyl)butanamide (1g)

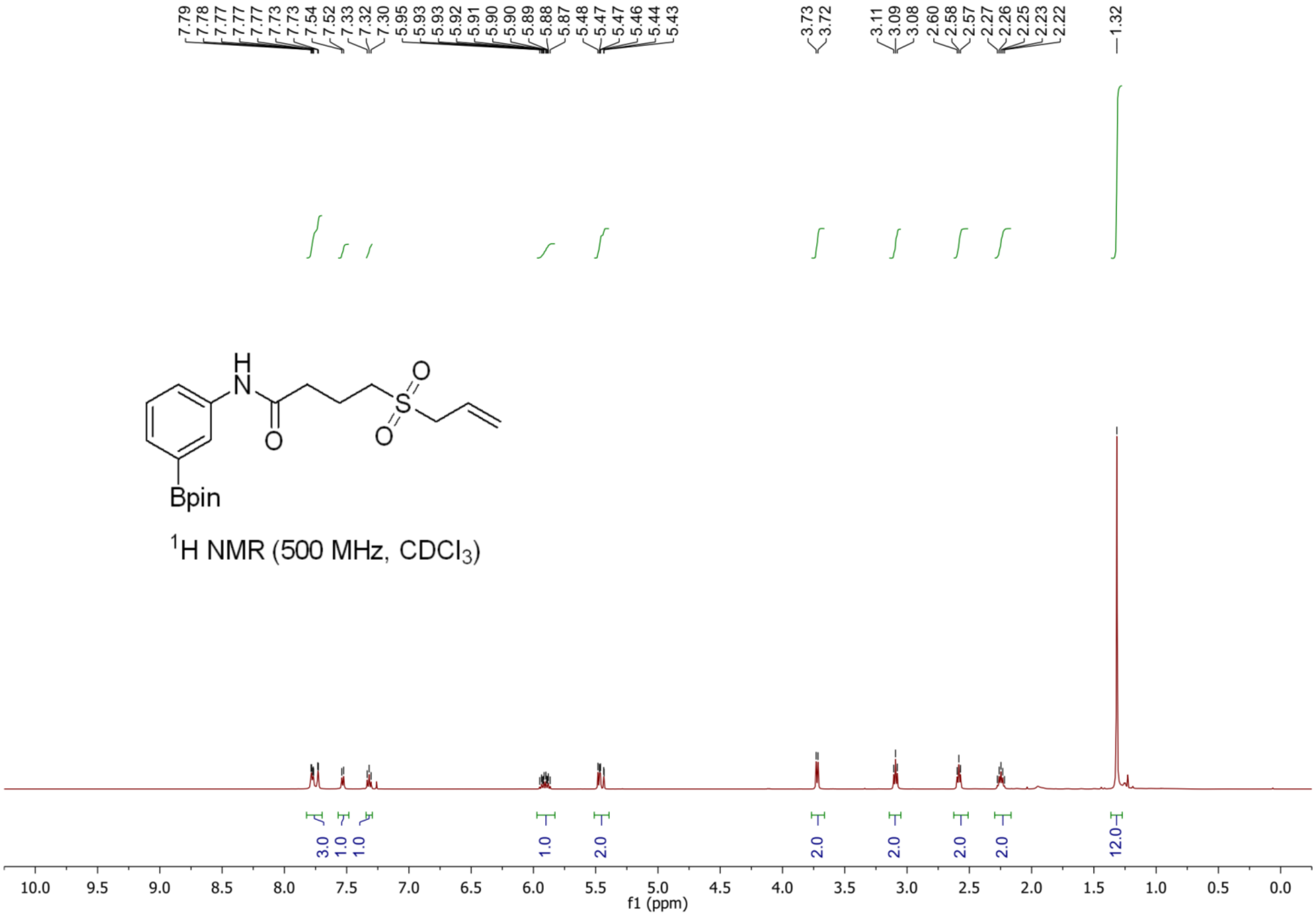

Go back to table of contents

4-(Allylsulfonyl)-*N*-(3-(4,4,5,5-tetramethyl-1,3,2-dioxaborolan-2-yl)phenyl)butanamide (1g)

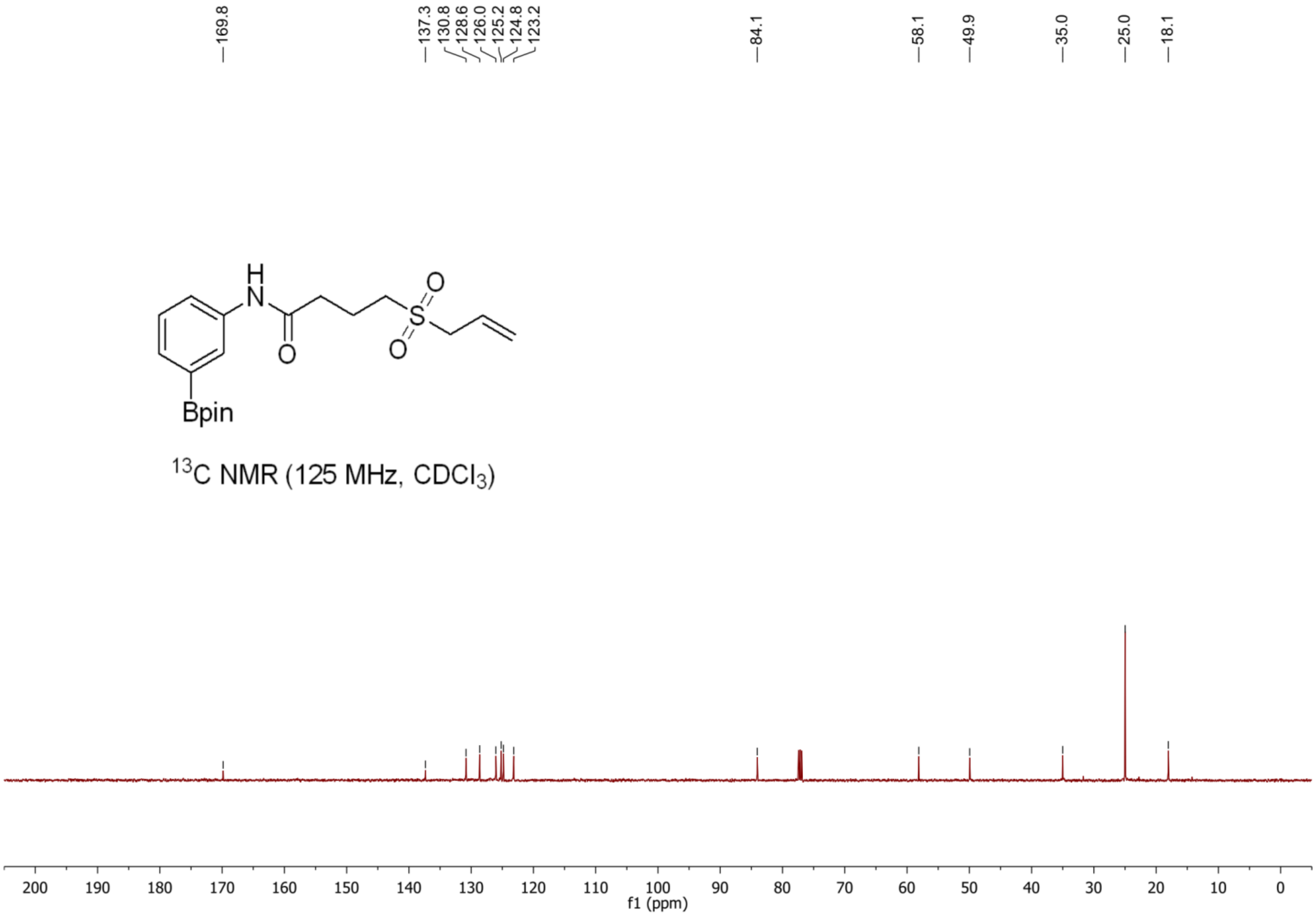

Go back to table of contents

3-(3-(Allylsulfonyl)propyl)-1*H*-indole (1h)

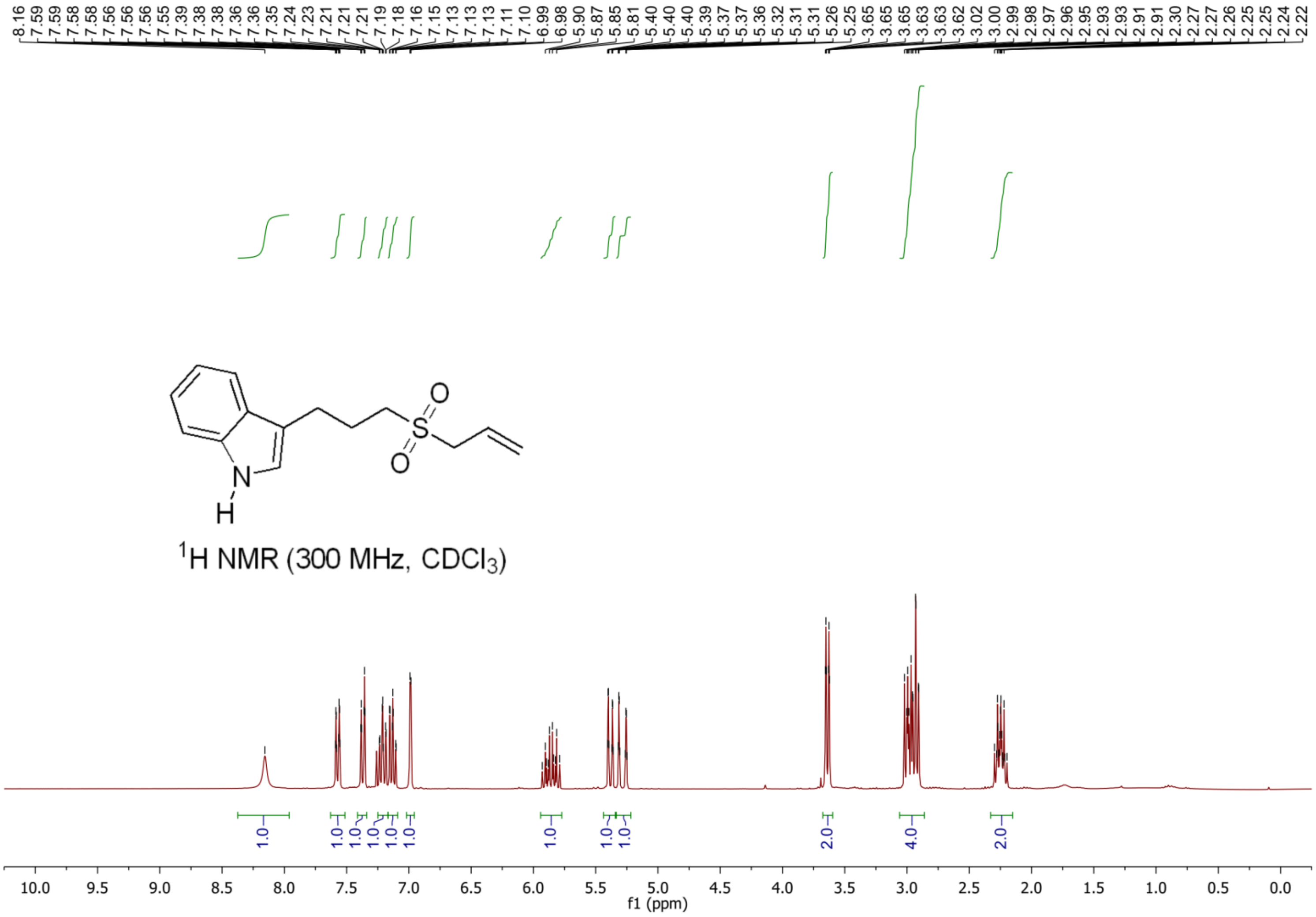

Go back to table of contents

3-(3-(Allylsulfonyl)propyl)-1*H*-indole (1h)

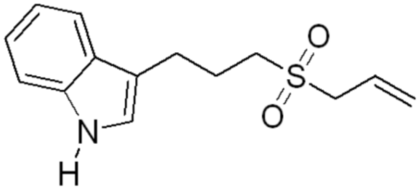

^{13}C NMR (75 MHz, $CDCl_3$)

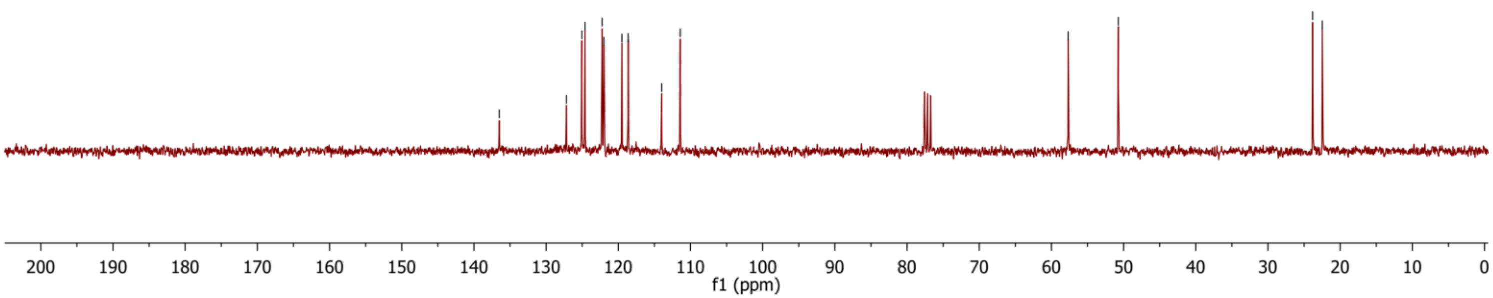

Go back to table of contents

4-(Allylsulfonyl)-1-(5-methylthiophen-2-yl)butan-1-one (1i)

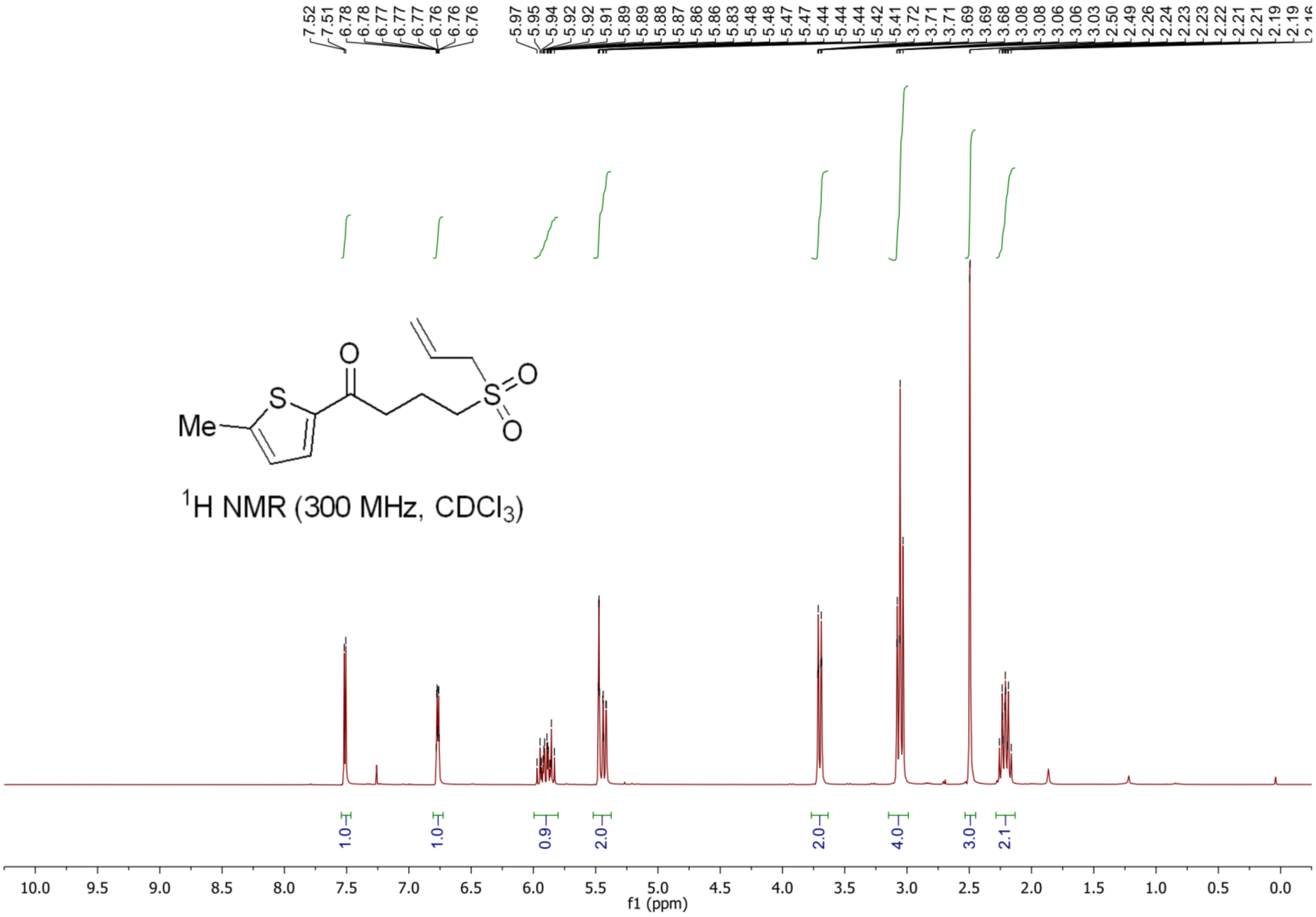

Go back to table of contents

4-(Allylsulfonyl)-1-(5-methylthiophen-2-yl)butan-1-one (1i)

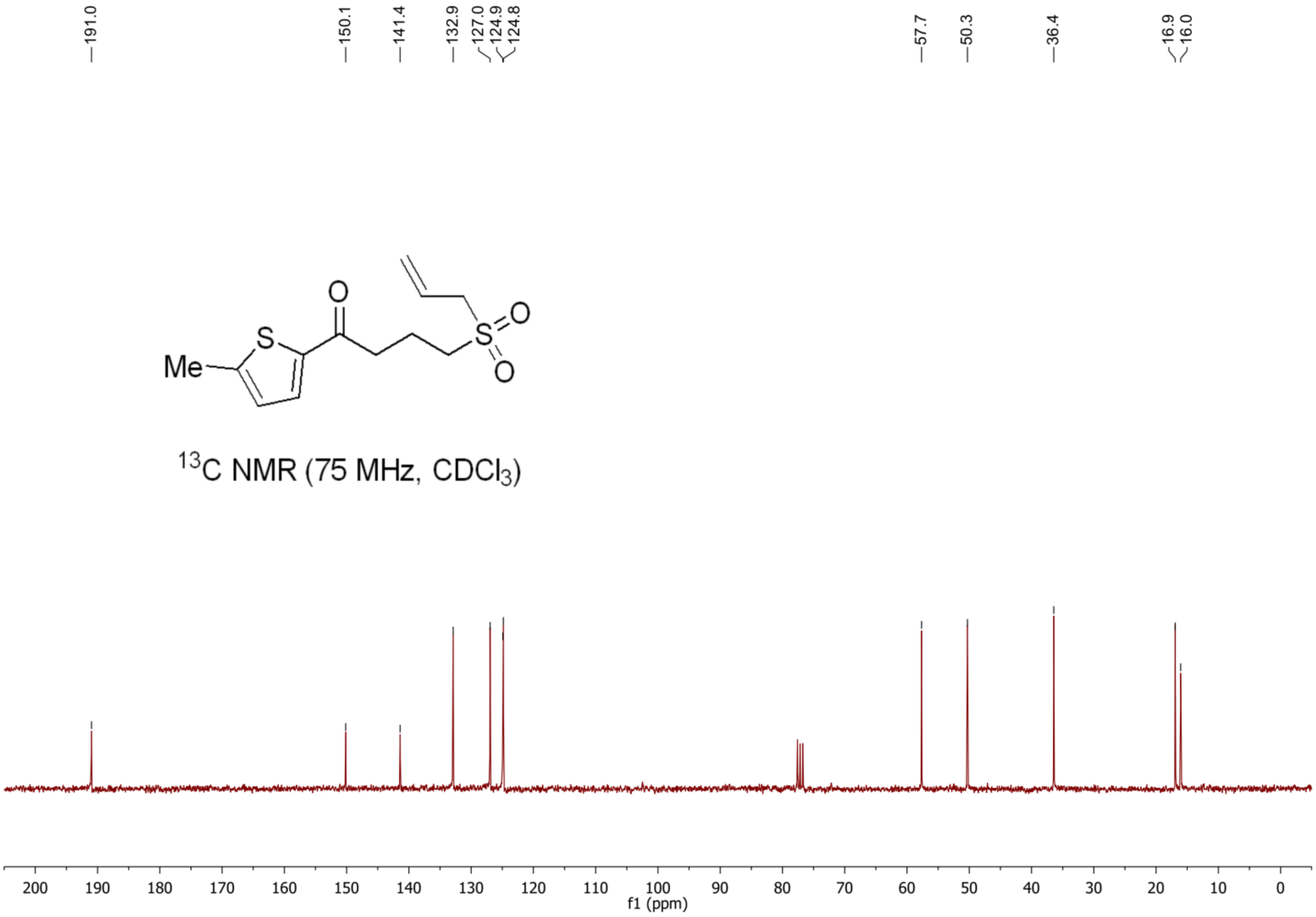

Go back to table of contents

1,7-Bis(allylsulfonyl)heptane (1j)

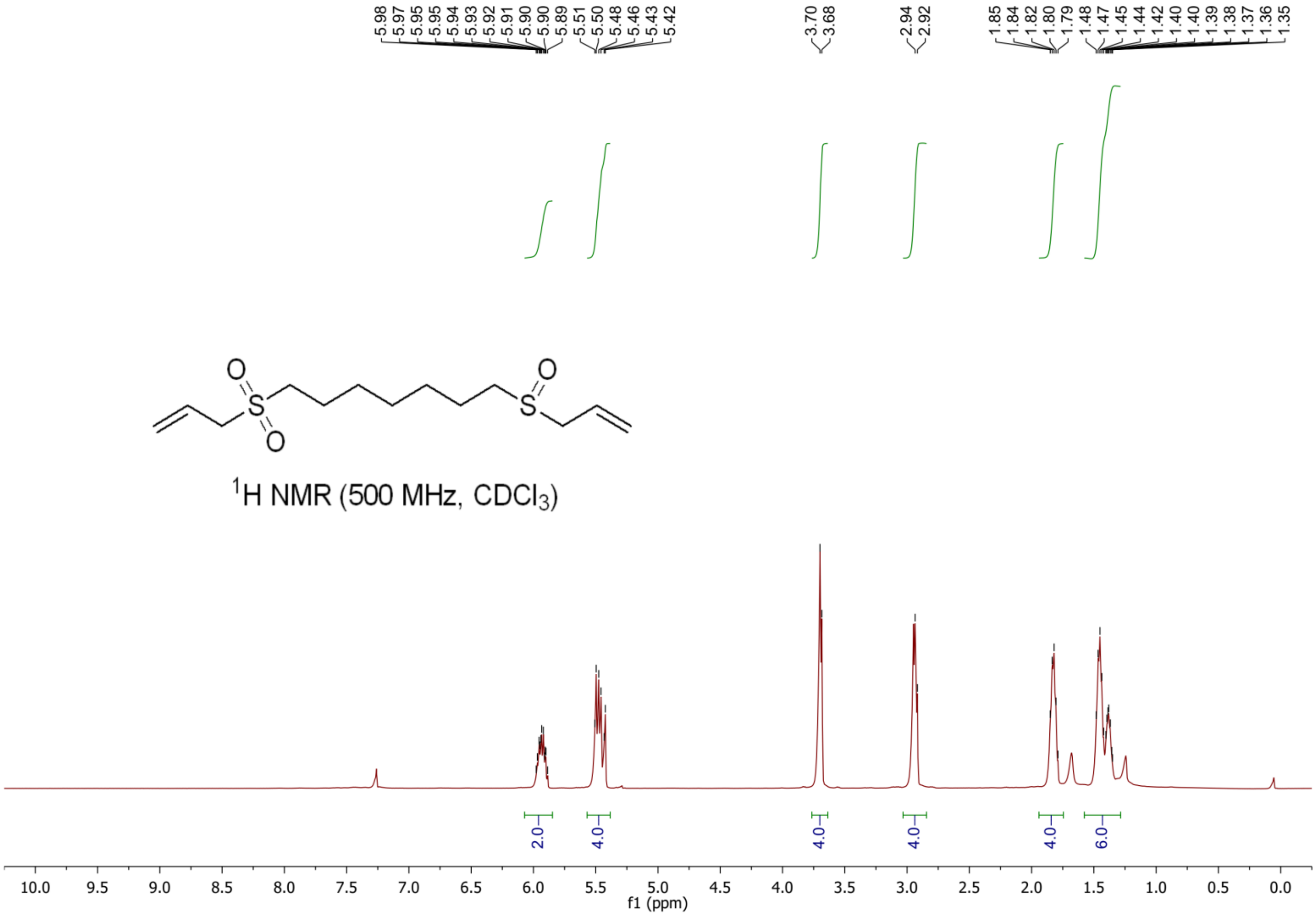

Go back to table of contents

1,7-Bis(allylsulfonyl)heptane (1j)

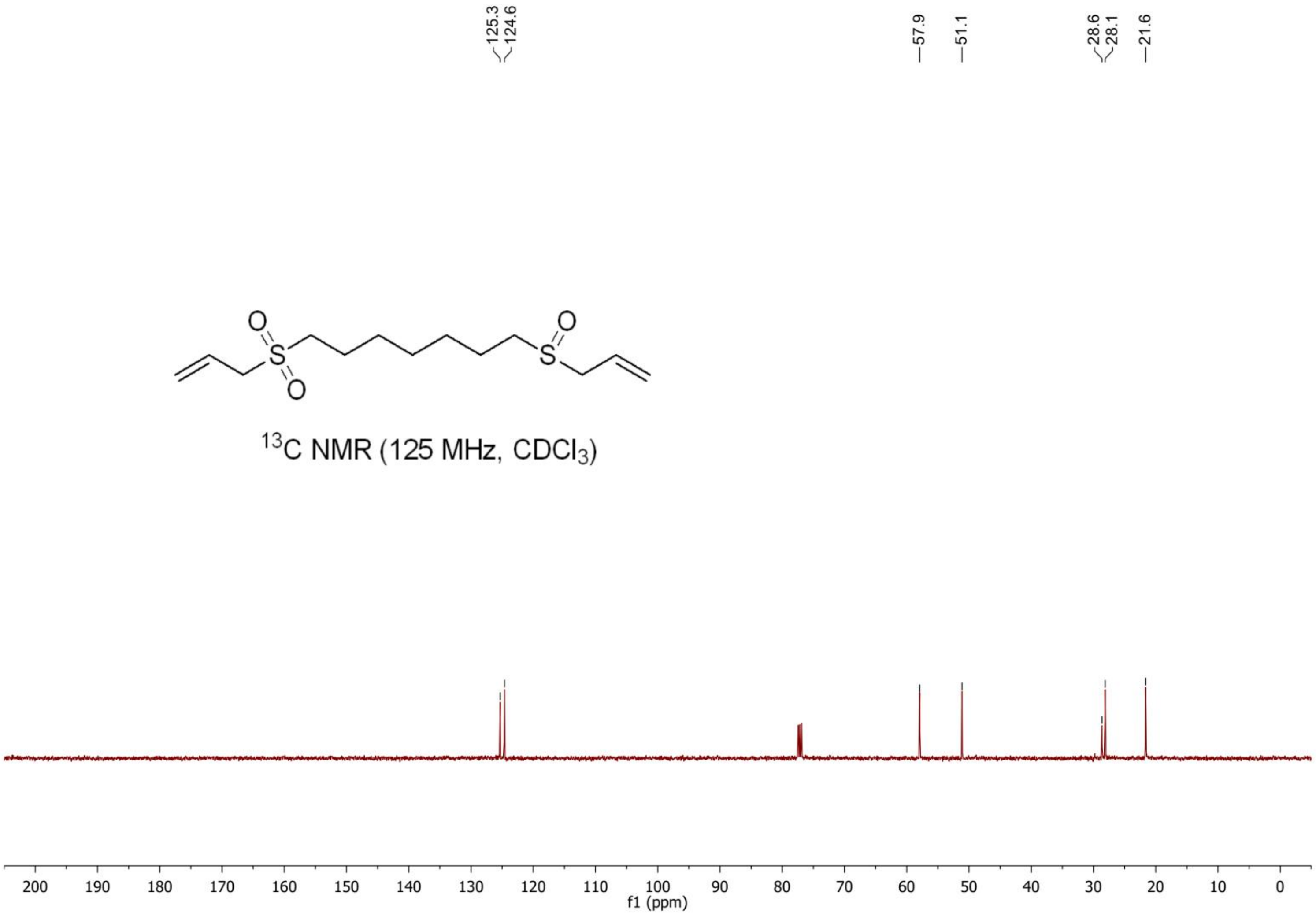

Go back to table of contents

1,4-Bis(allylsulfonyl)butane (1k)

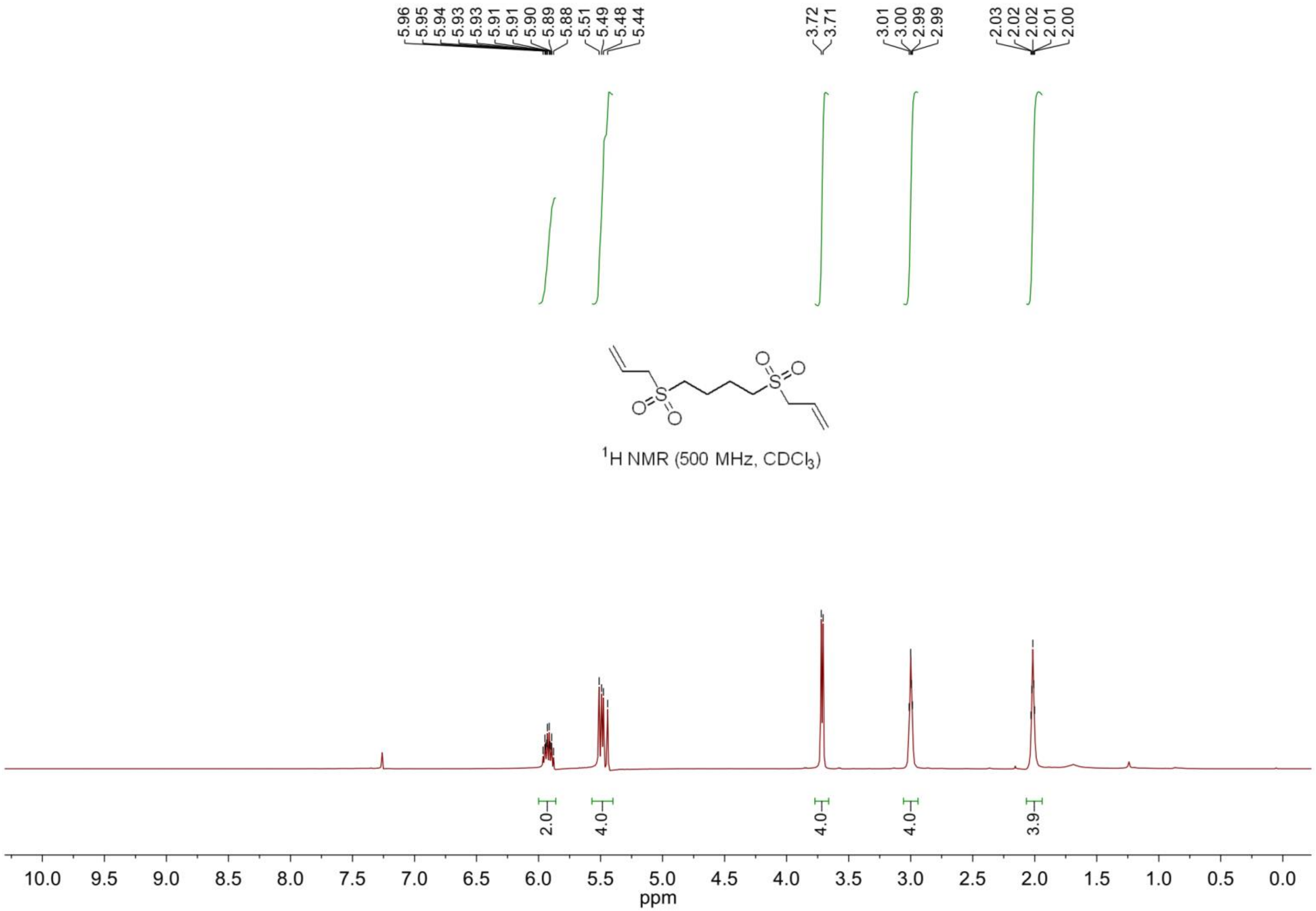

Go back to table of contents

1,4-Bis(allylsulfonyl)butane (1k)

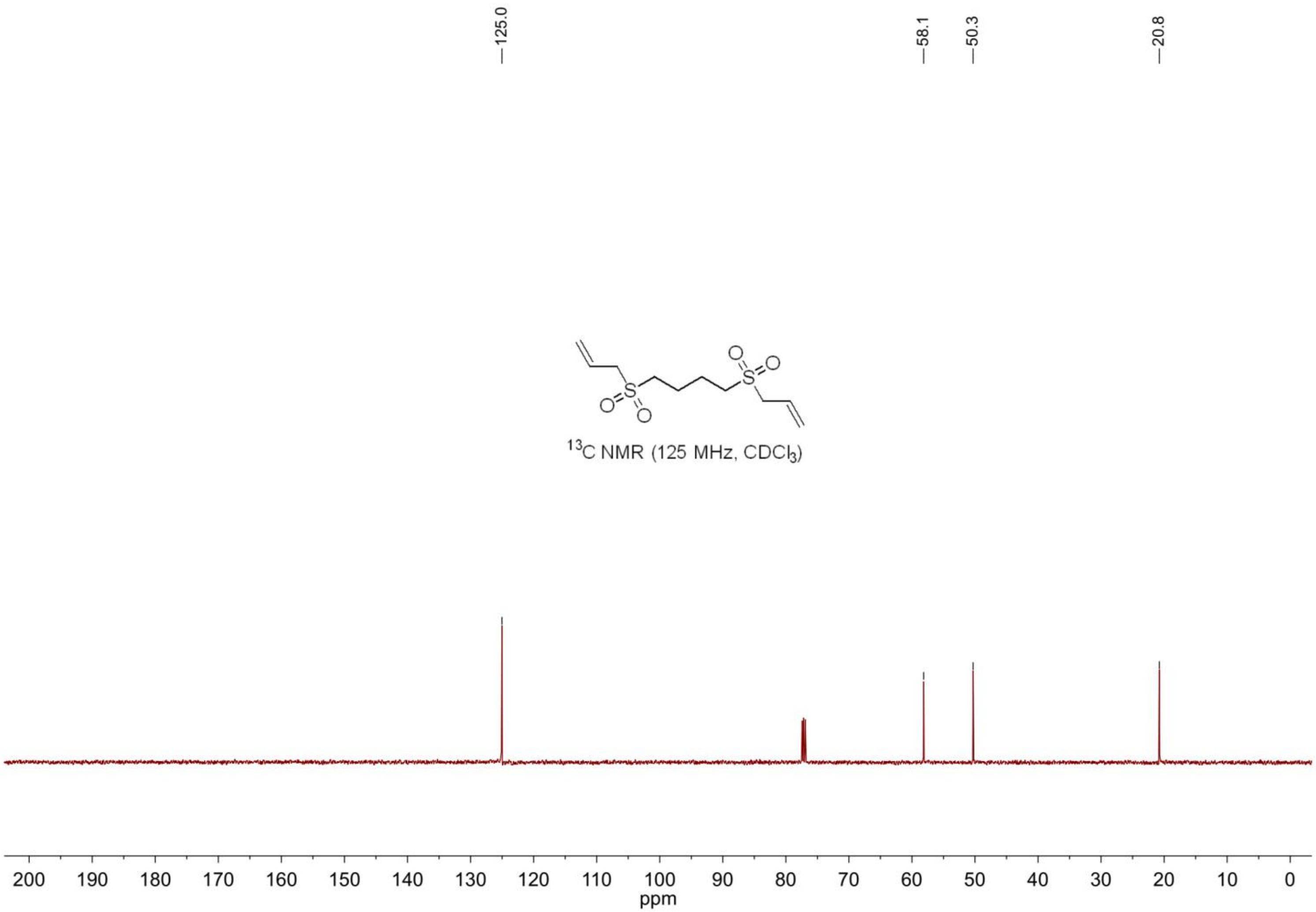

Go back to table of contents

1-(Allylsulfonyl)tetracosa-9,11-diyne (1l)

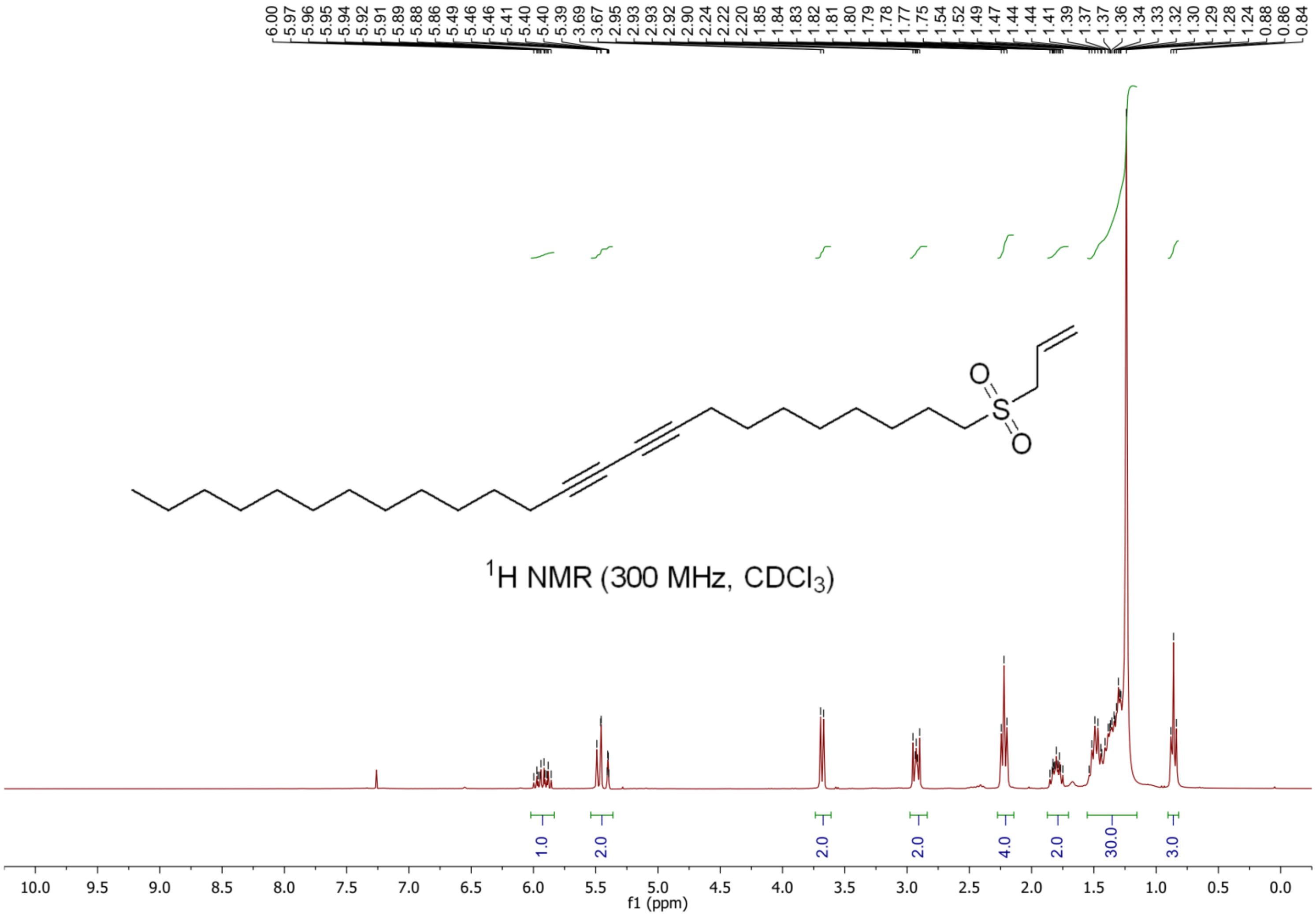

Go back to table of contents

1-(Allylsulfonyl)tetracosa-9,11-diyne (1l)

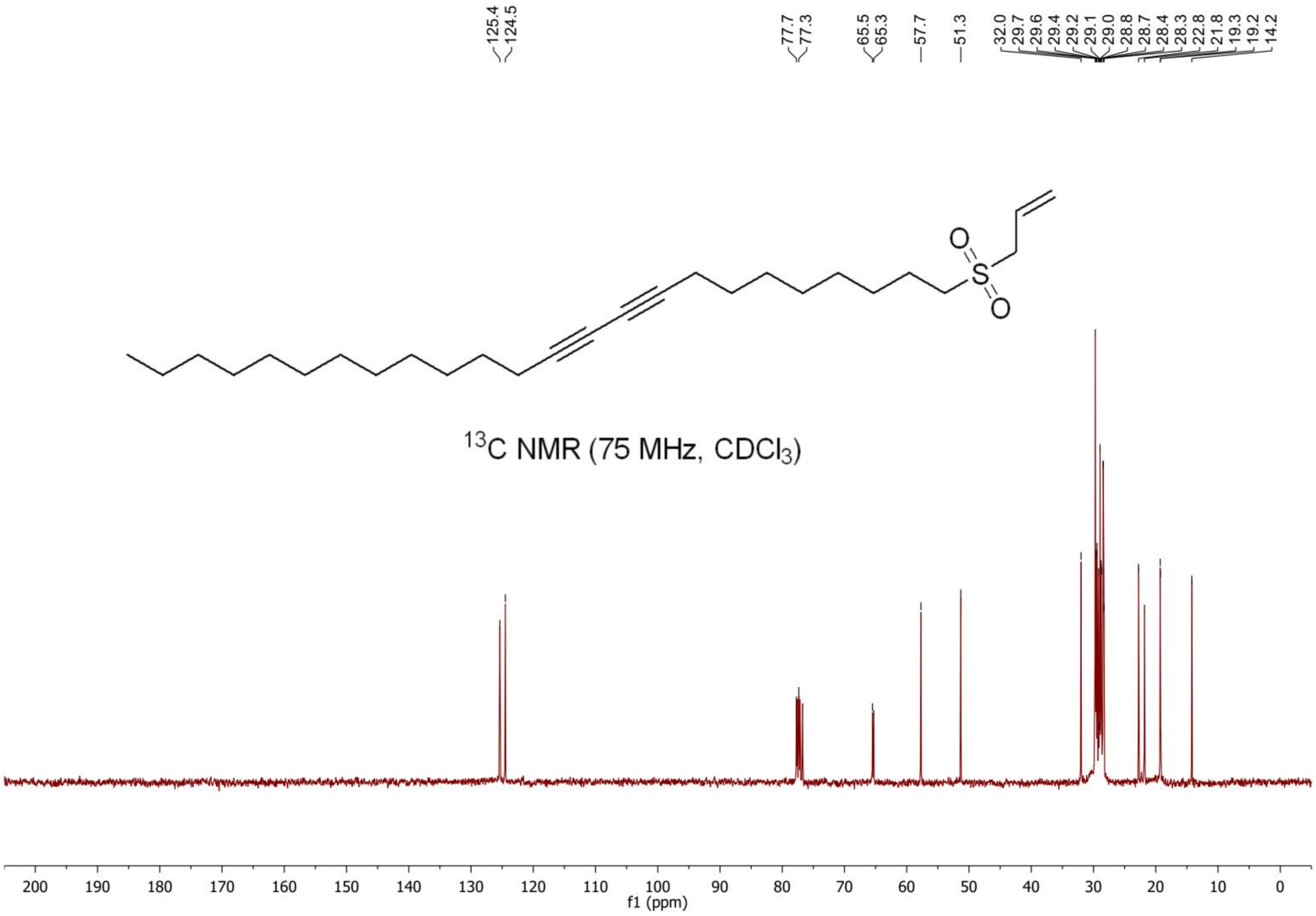

Go back to table of contents

(Allylsulfonyl)cyclobutane (1m)

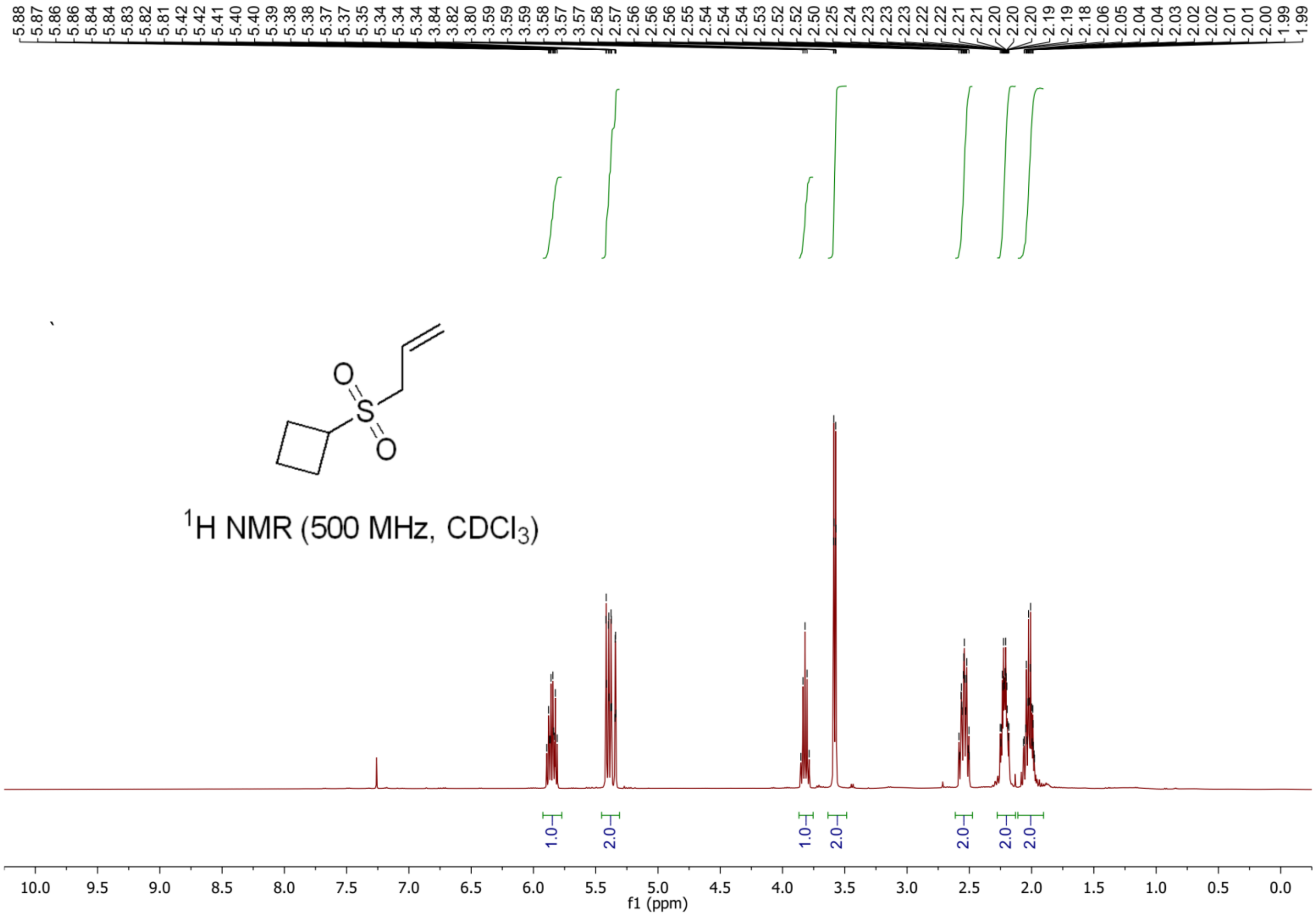

Go back to table of contents

(Allylsulfonyl)cyclobutane (1m)

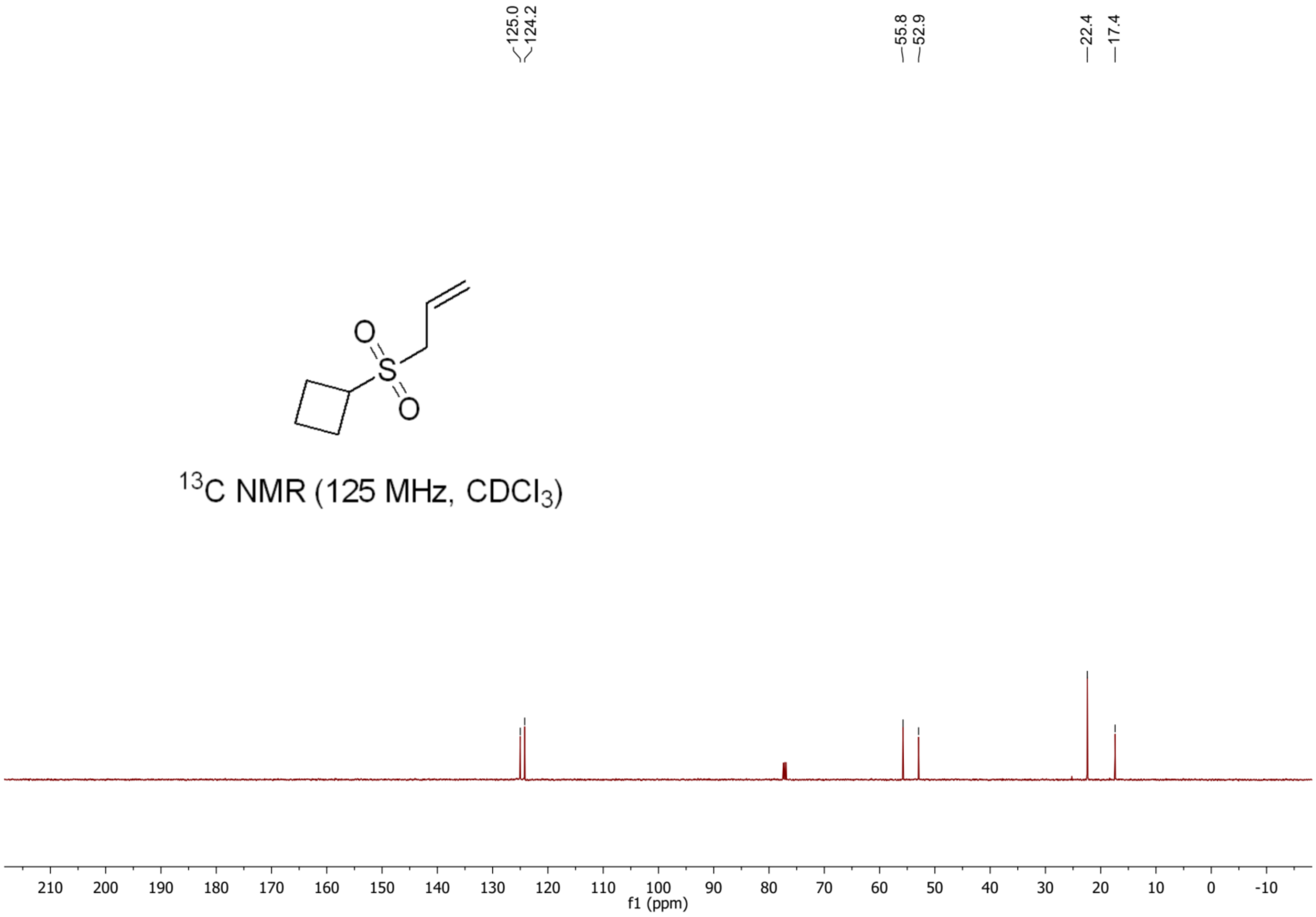

Go back to table of contents

((1*S,2*R**)-2-(Allylsulfonyl)cyclopropyl)benzene (1n)**

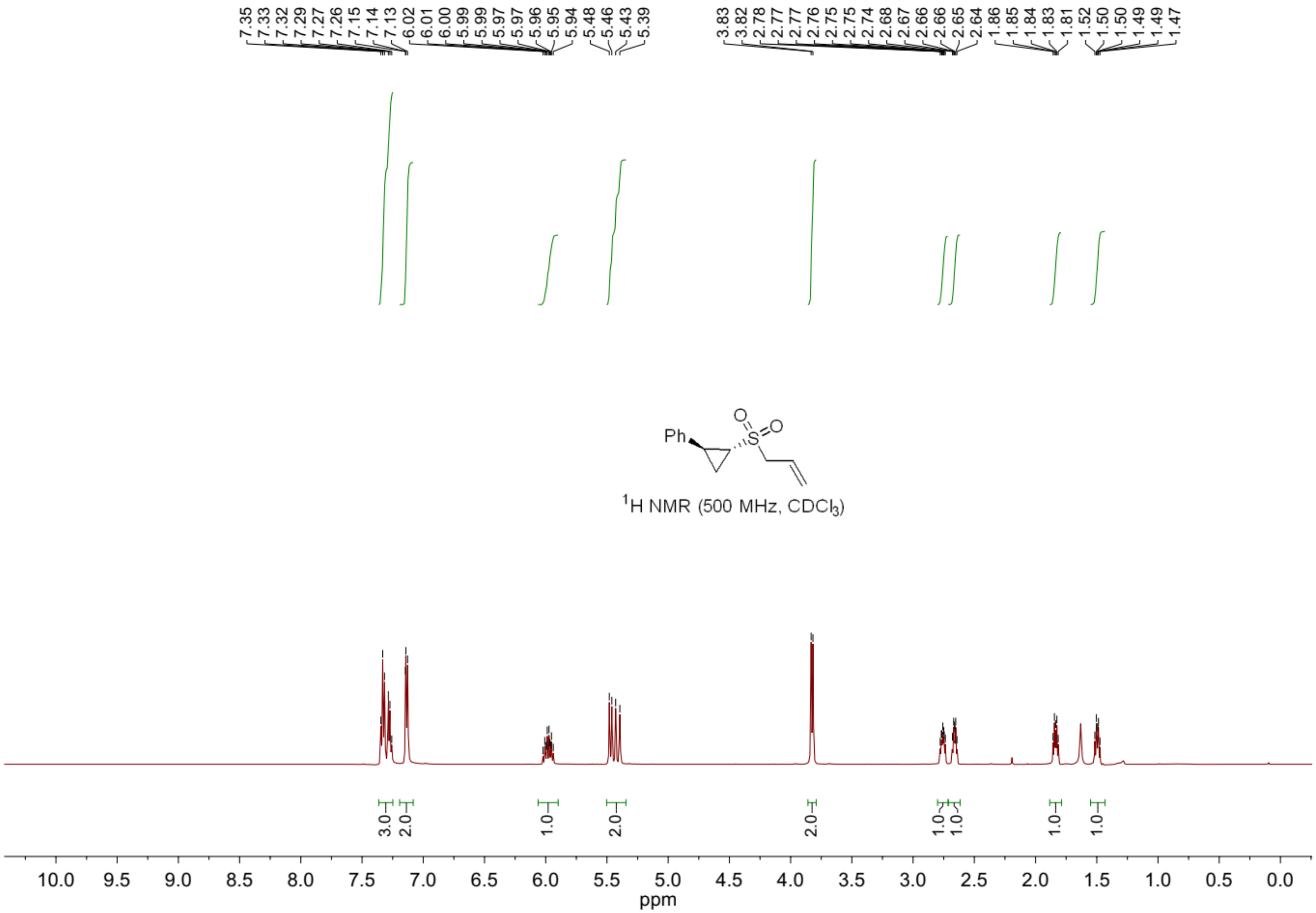

Go back to table of contents

((1*S,2*R**)-2-(Allylsulfonyl)cyclopropyl)benzene (1n)**

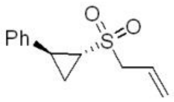

^{13}C NMR (125 MHz, $CDCl_3$)

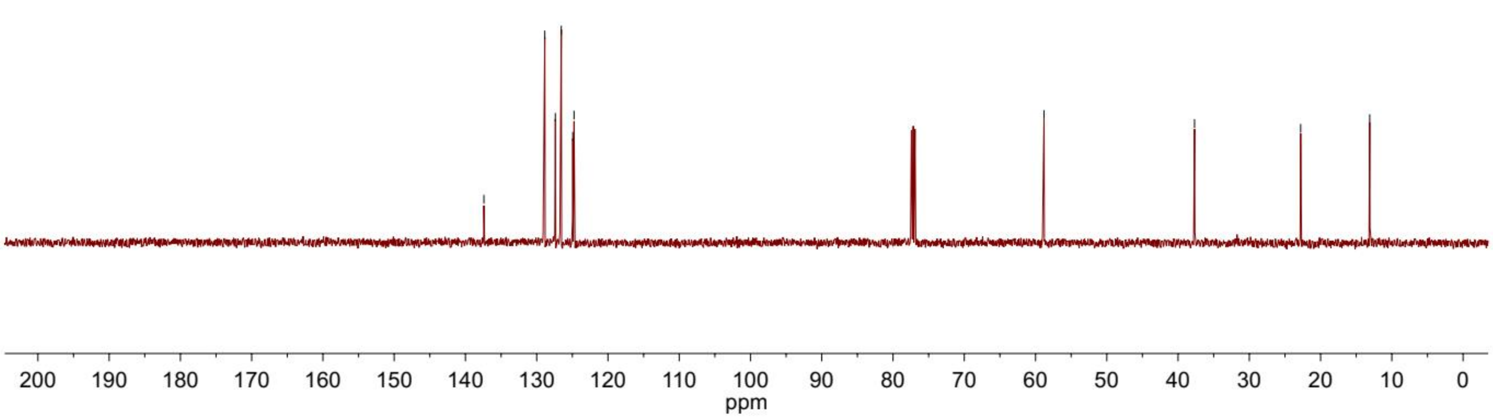

Go back to table of contents

2-(Allylsulfonyl)-2,3-dihydro-1*H*-indene (1o)

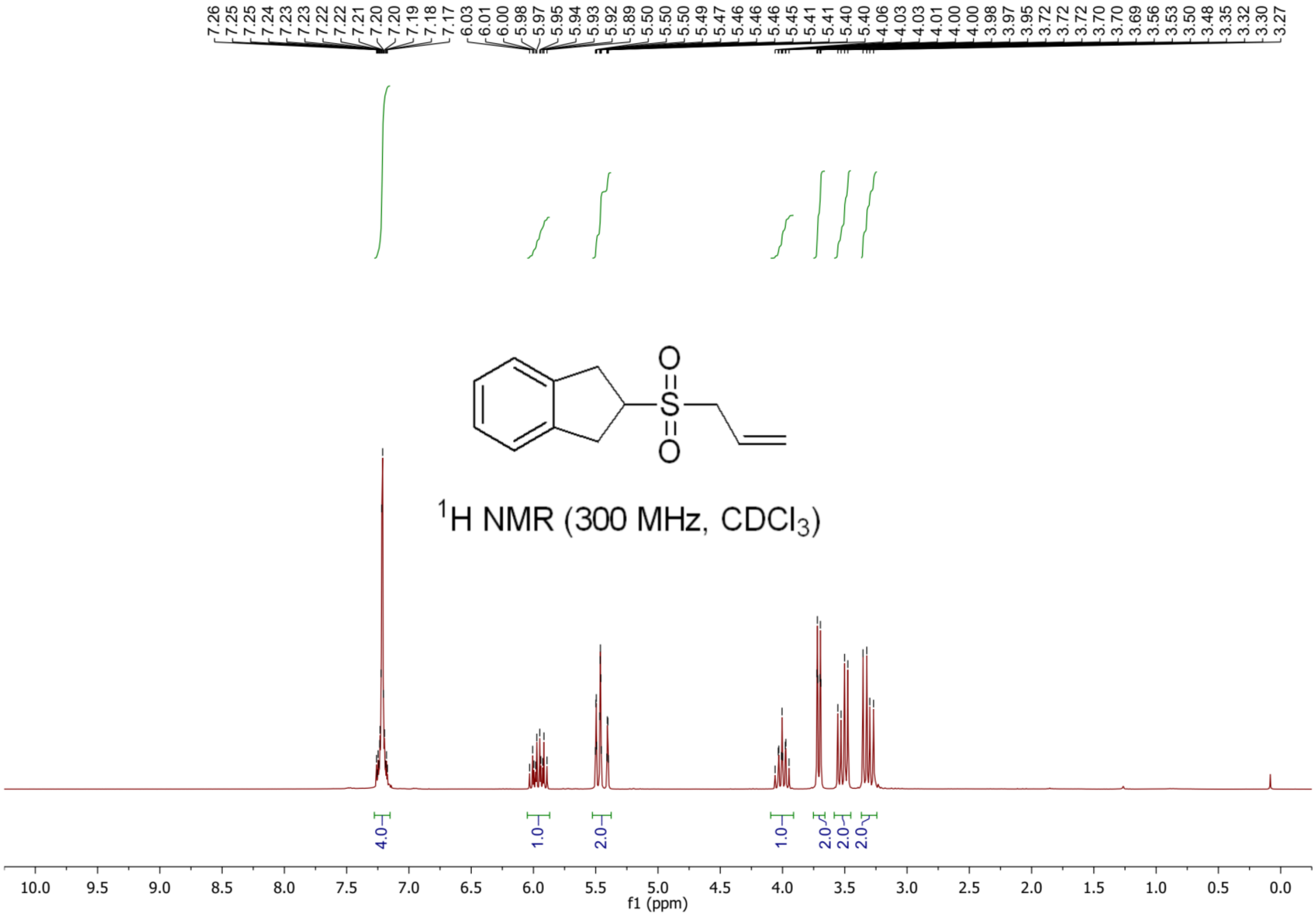

Go back to table of contents

2-(Allylsulfonyl)-2,3-dihydro-1*H*-indene (1o)

139.4
127.4
124.9
124.6
124.5
59.5
56.5
33.3

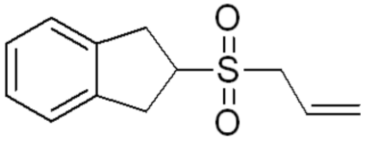

^{13}C NMR (75 MHz, $CDCl_3$)

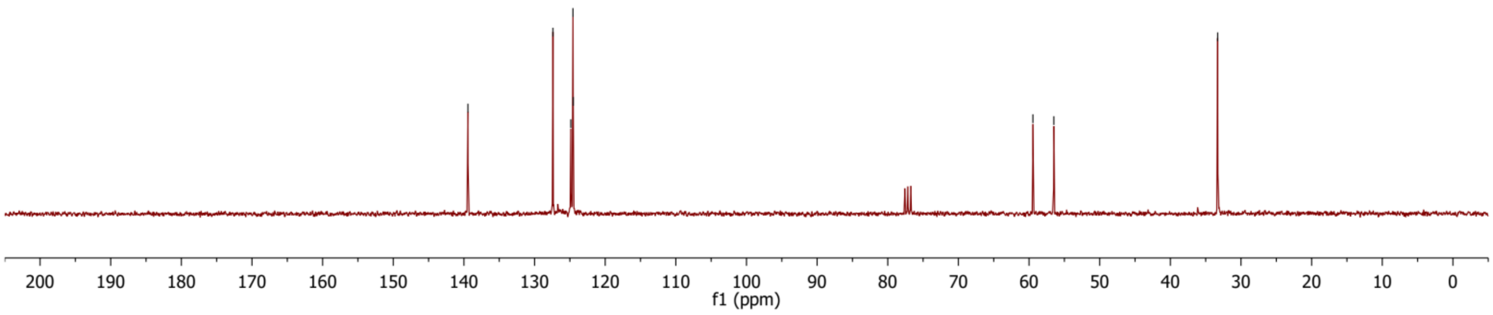

Go back to table of contents

7-(Allylsulfonyl)pentadecane (1p)

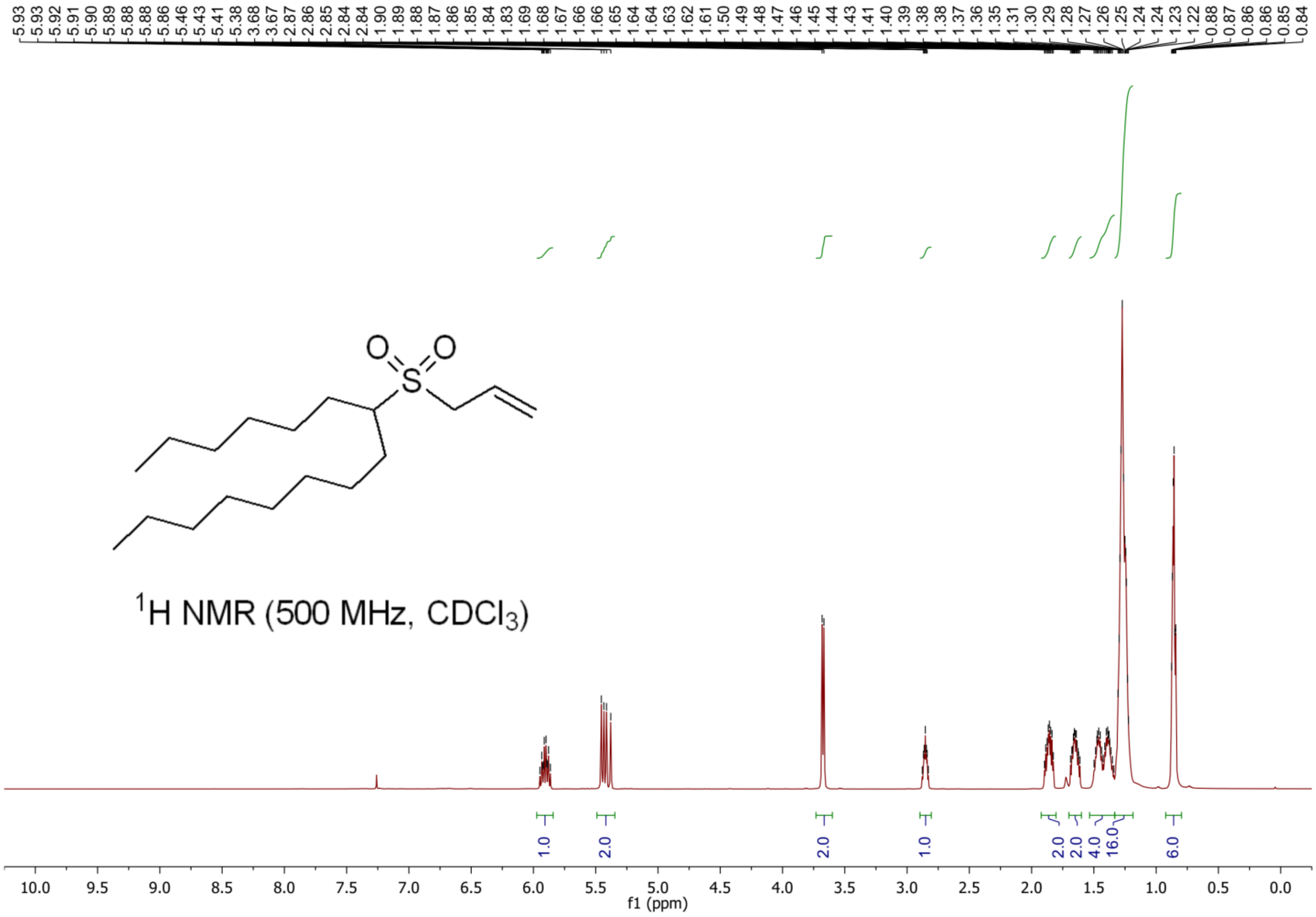

Go back to table of contents

7-(Allylsulfonyl)pentadecane (1p)

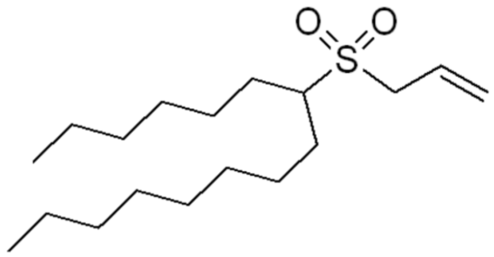

^{13}C NMR (125 MHz, $CDCl_3$)

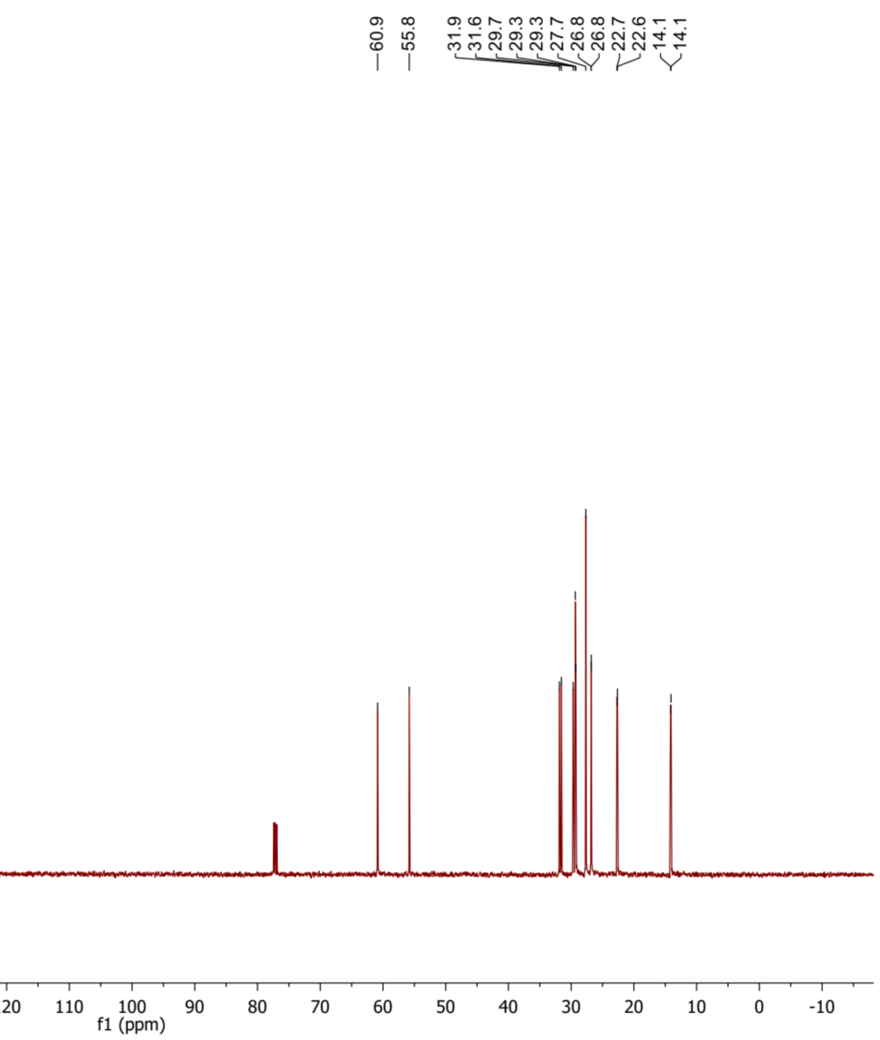

Go back to table of contents

(1-(Allylsulfonyl)cyclopropyl)benzene (1g)

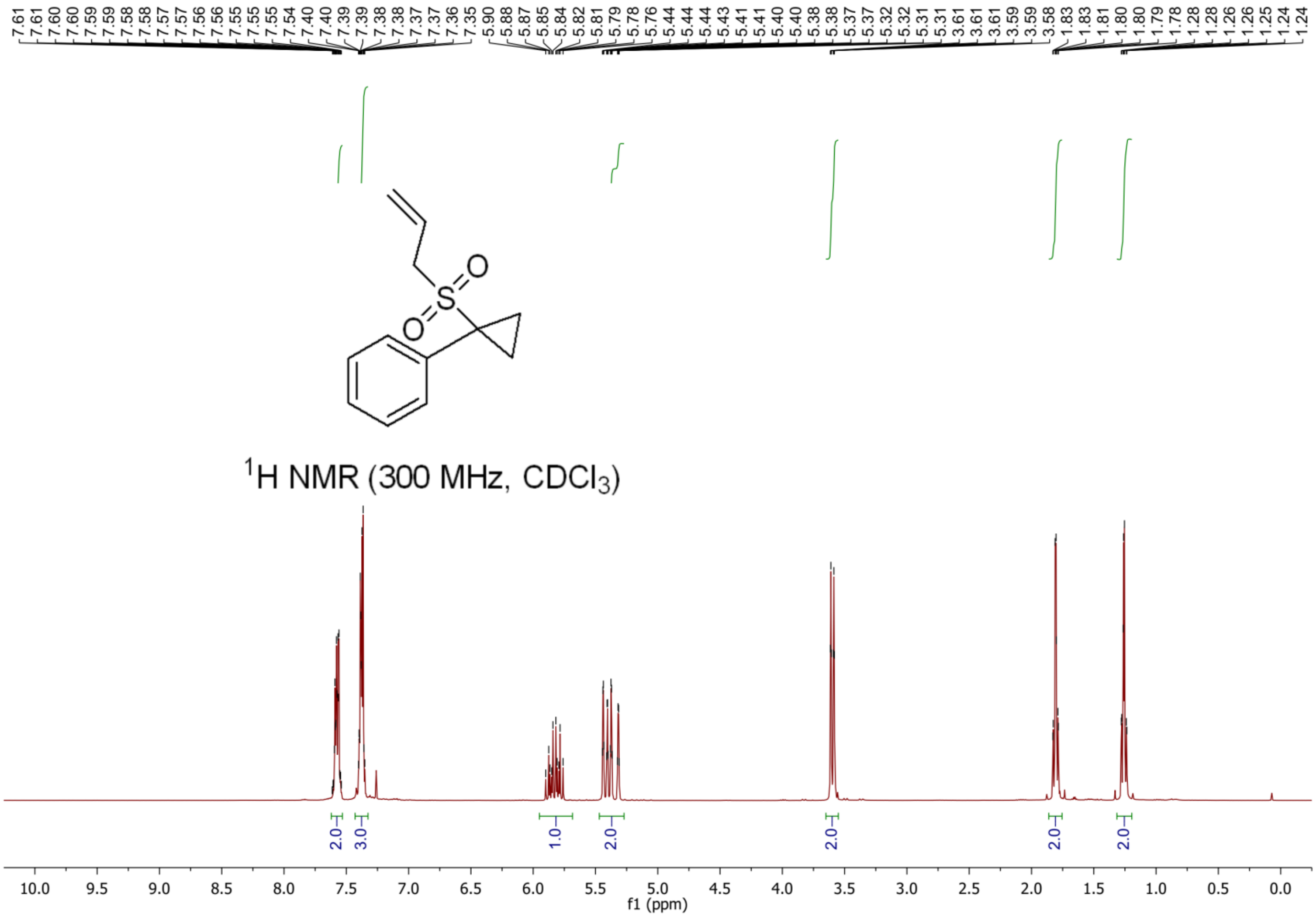

Go back to table of contents

(1-(Allylsulfonyl)cyclopropyl)benzene (1q)

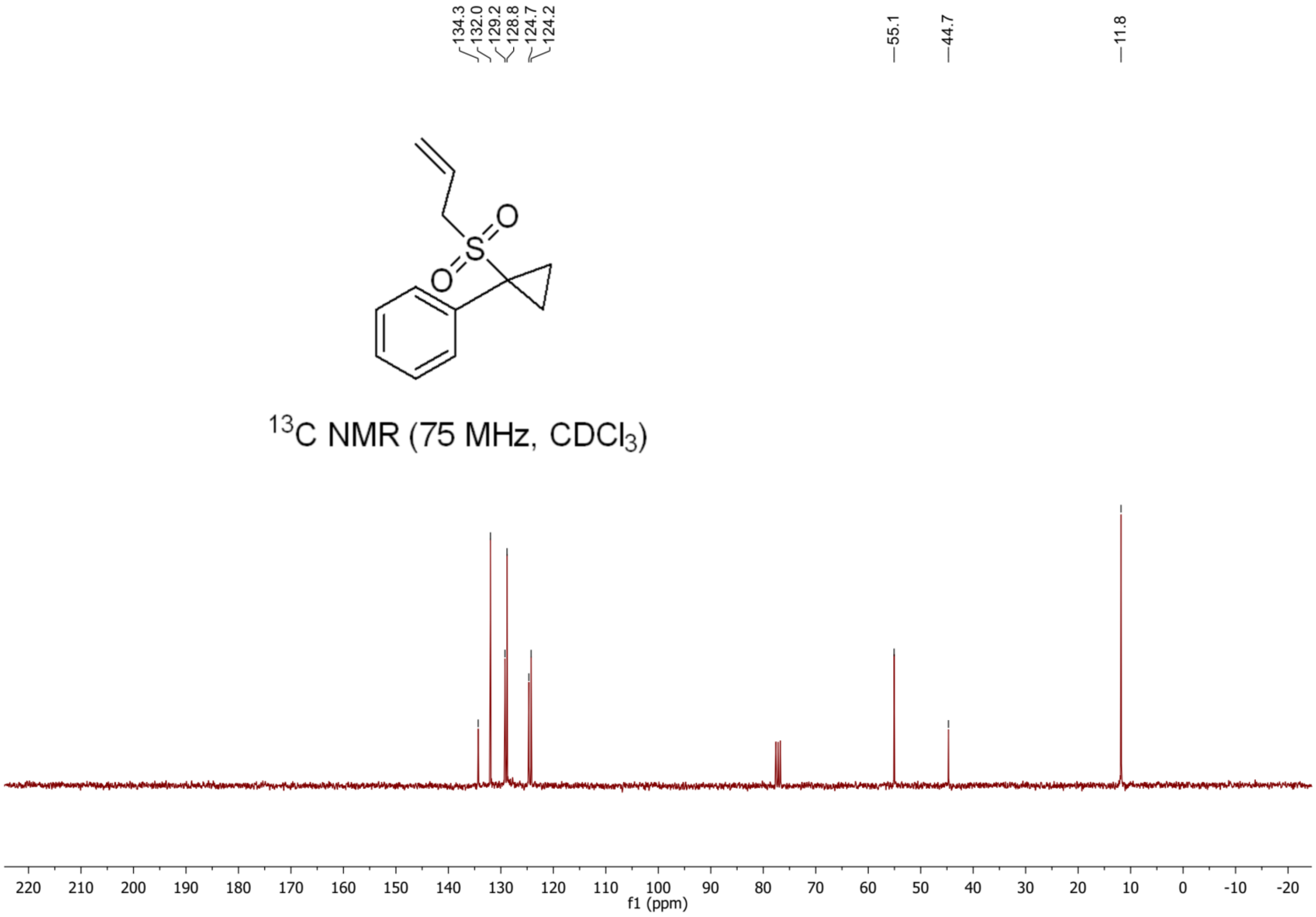

Go back to table of contents

2-(Allylsulfonyl)-2-methylbutane (1r)

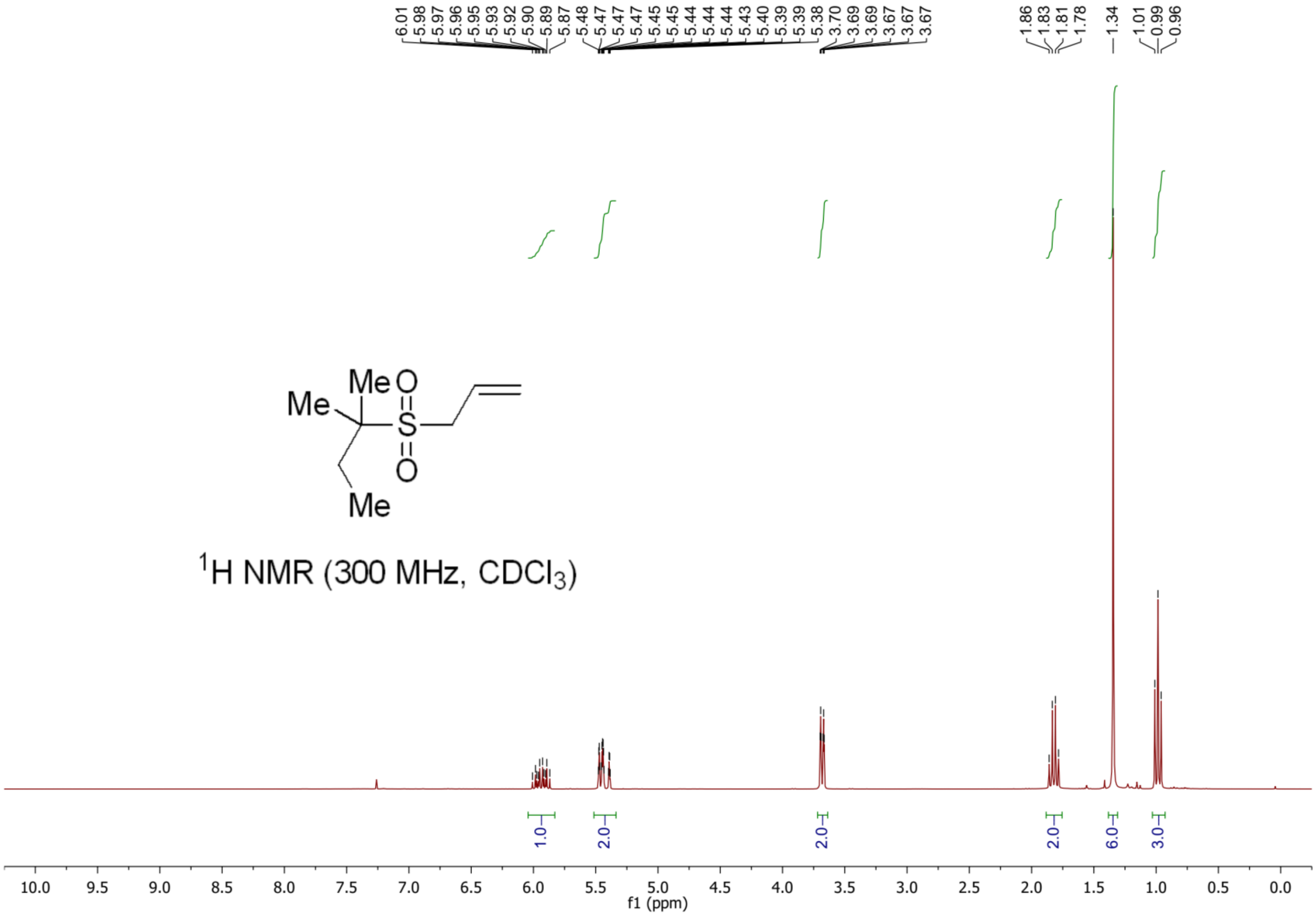

Go back to table of contents

2-(Allylsulfonyl)-2-methylbutane (1r)

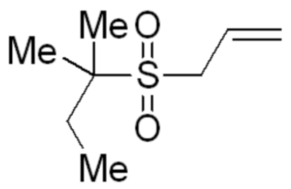

^{13}C NMR (75 MHz, $CDCl_3$)

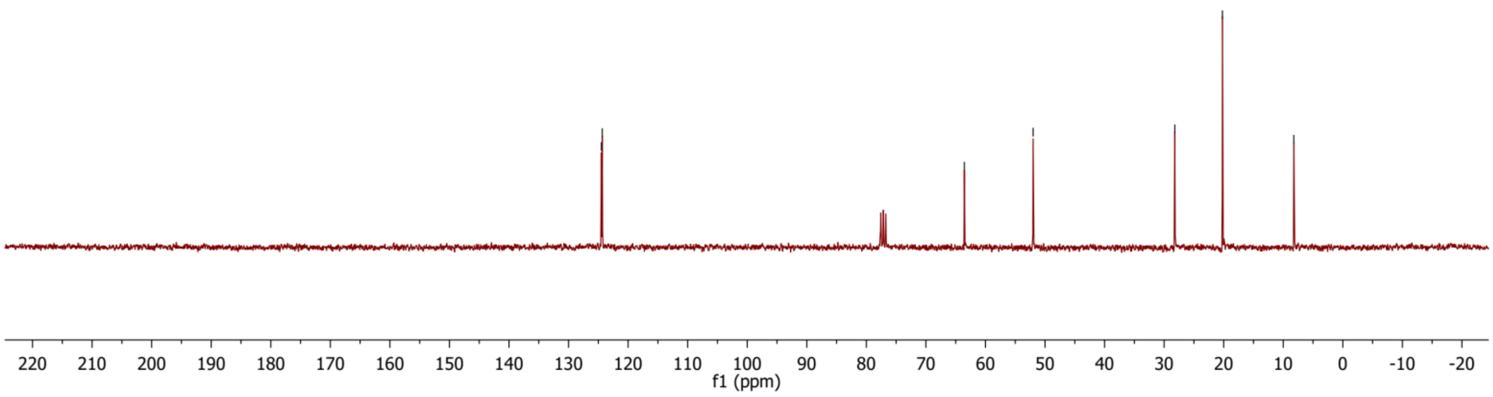

Go back to table of contents

1-(Allylsulfonyl)adamantane (1s)

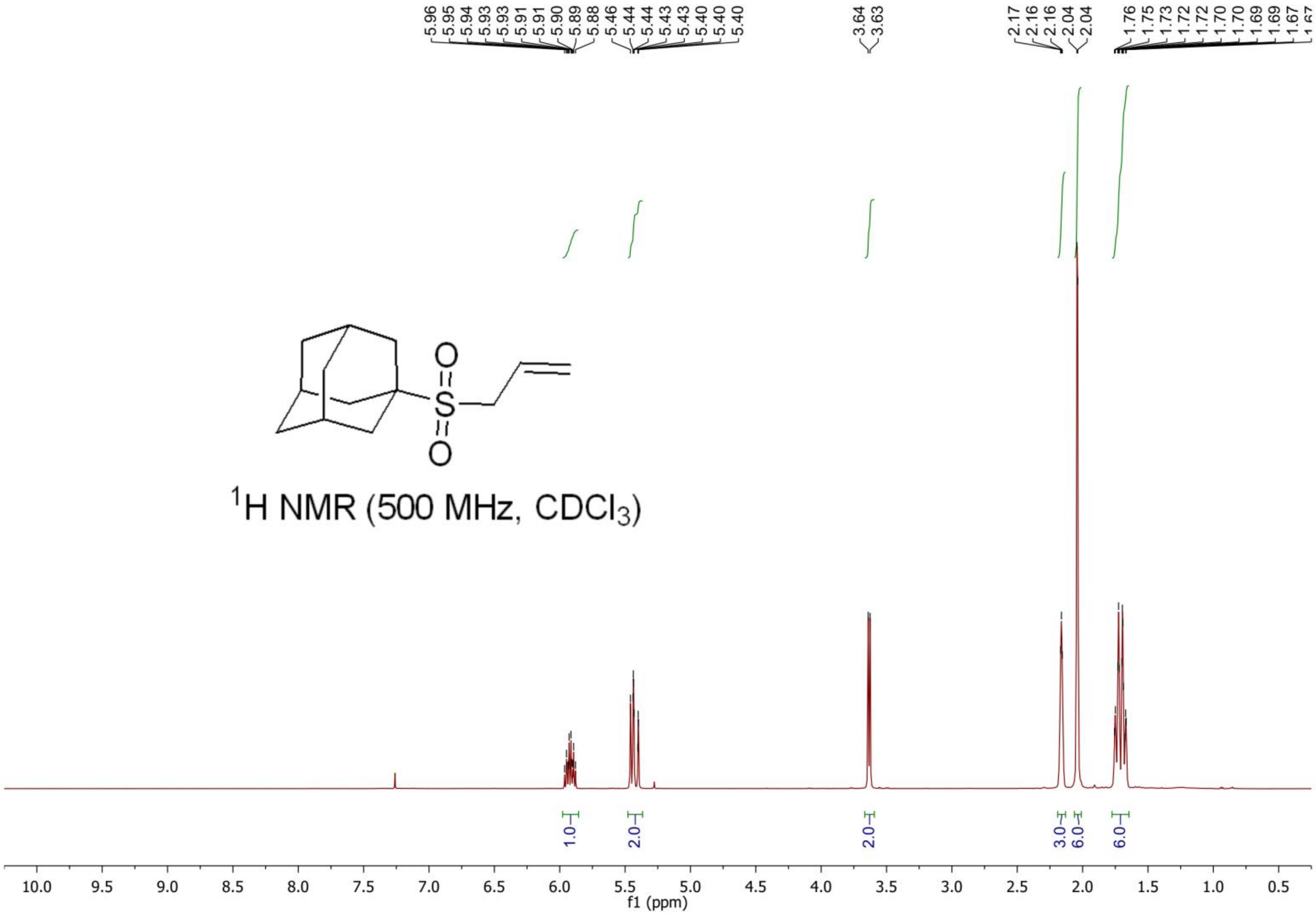

Go back to table of contents

1-(Allylsulfonyl)adamantane (1s)

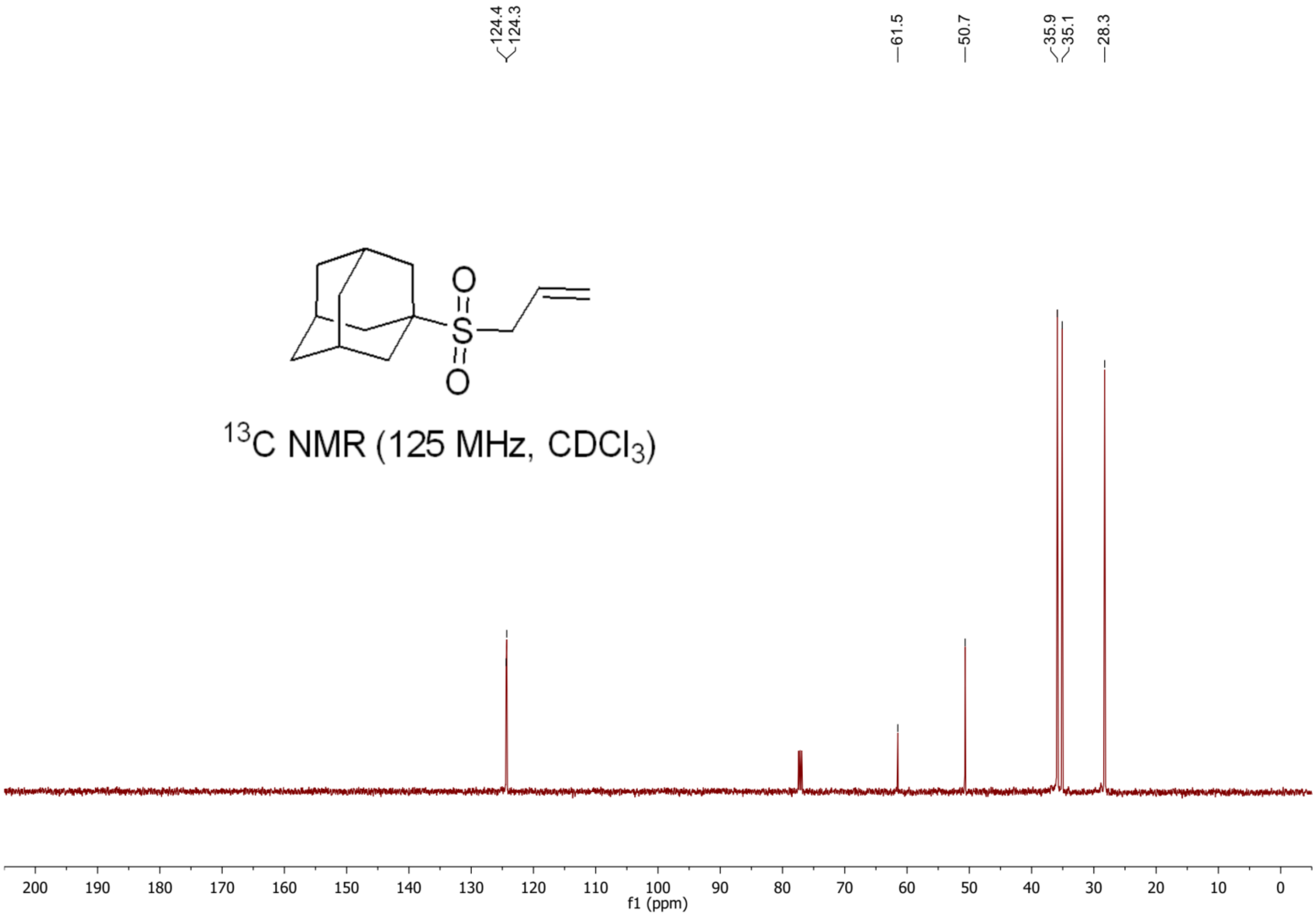

Go back to table of contents

4-(Allylsulfonyl)-4-methyltetrahydro-2*H*-pyran (1t)

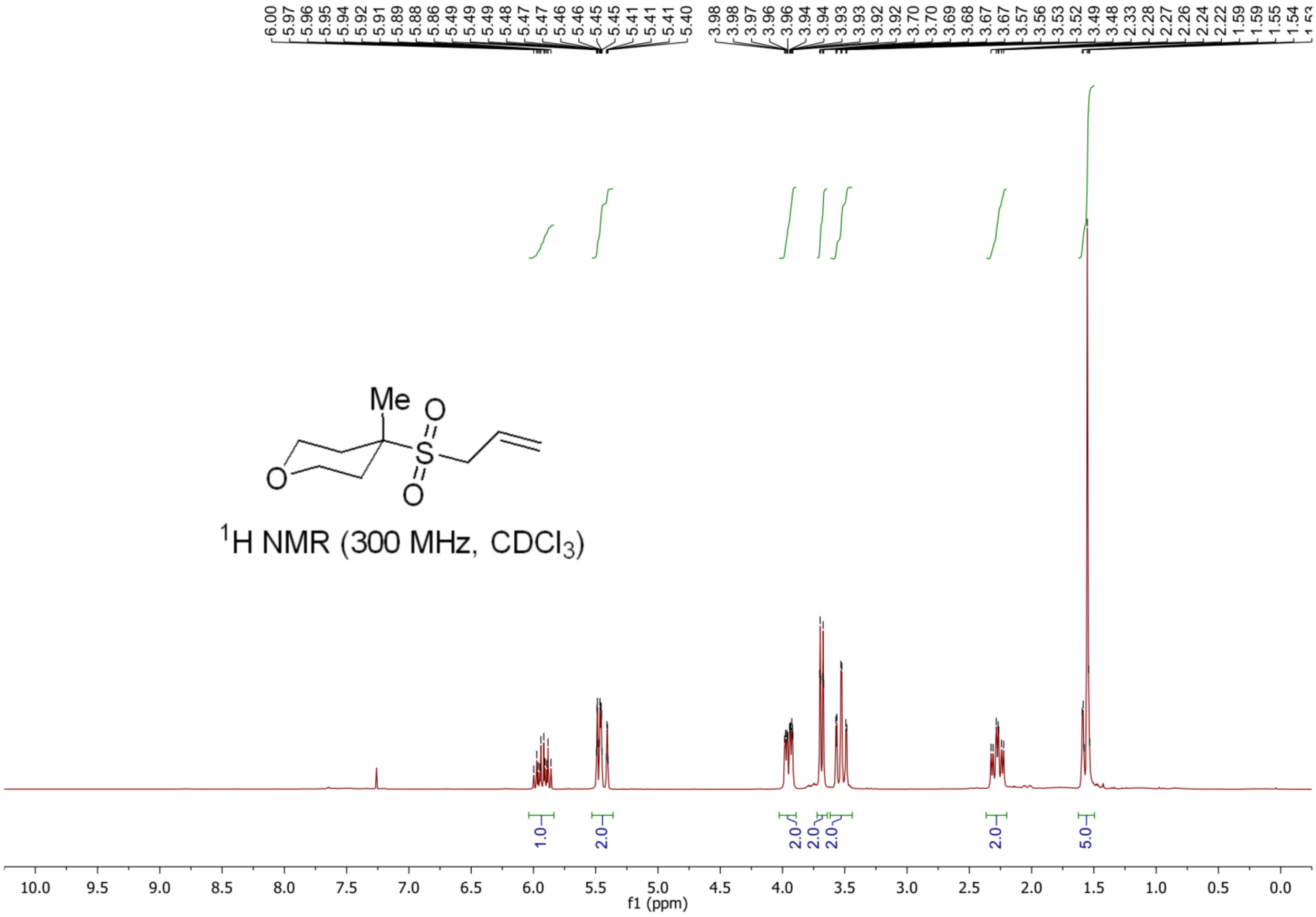

Go back to table of contents

4-(Allylsulfonyl)-4-methyltetrahydro-2*H*-pyran (1t)

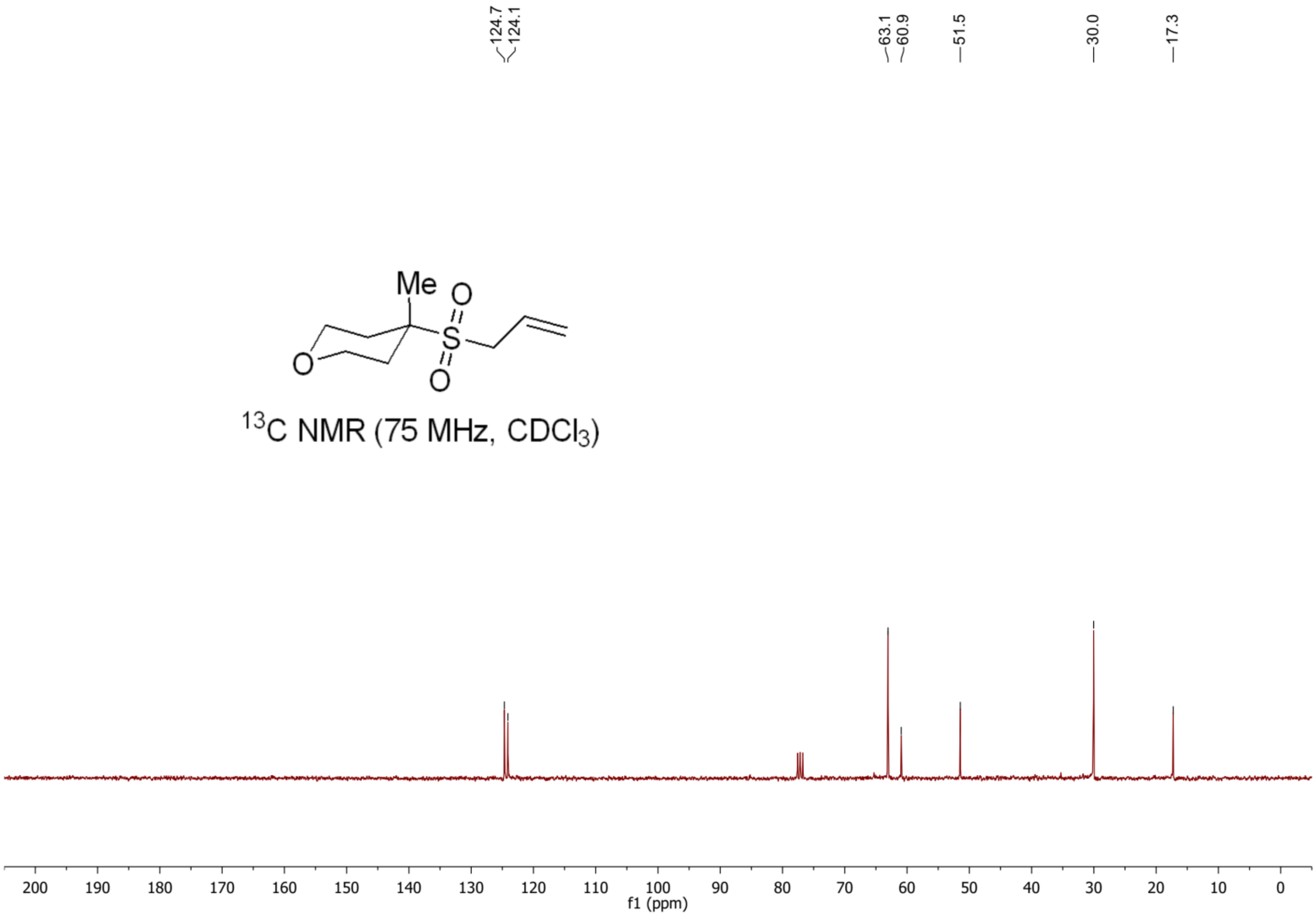

Go back to table of contents

Di-*tert*-butyl ((2*S*,3*S*,4*R*)-2-(4-(allylsulfonyl)butyl)tetrahydrothiophene-3,4-diyl)dicarbamate (1w)

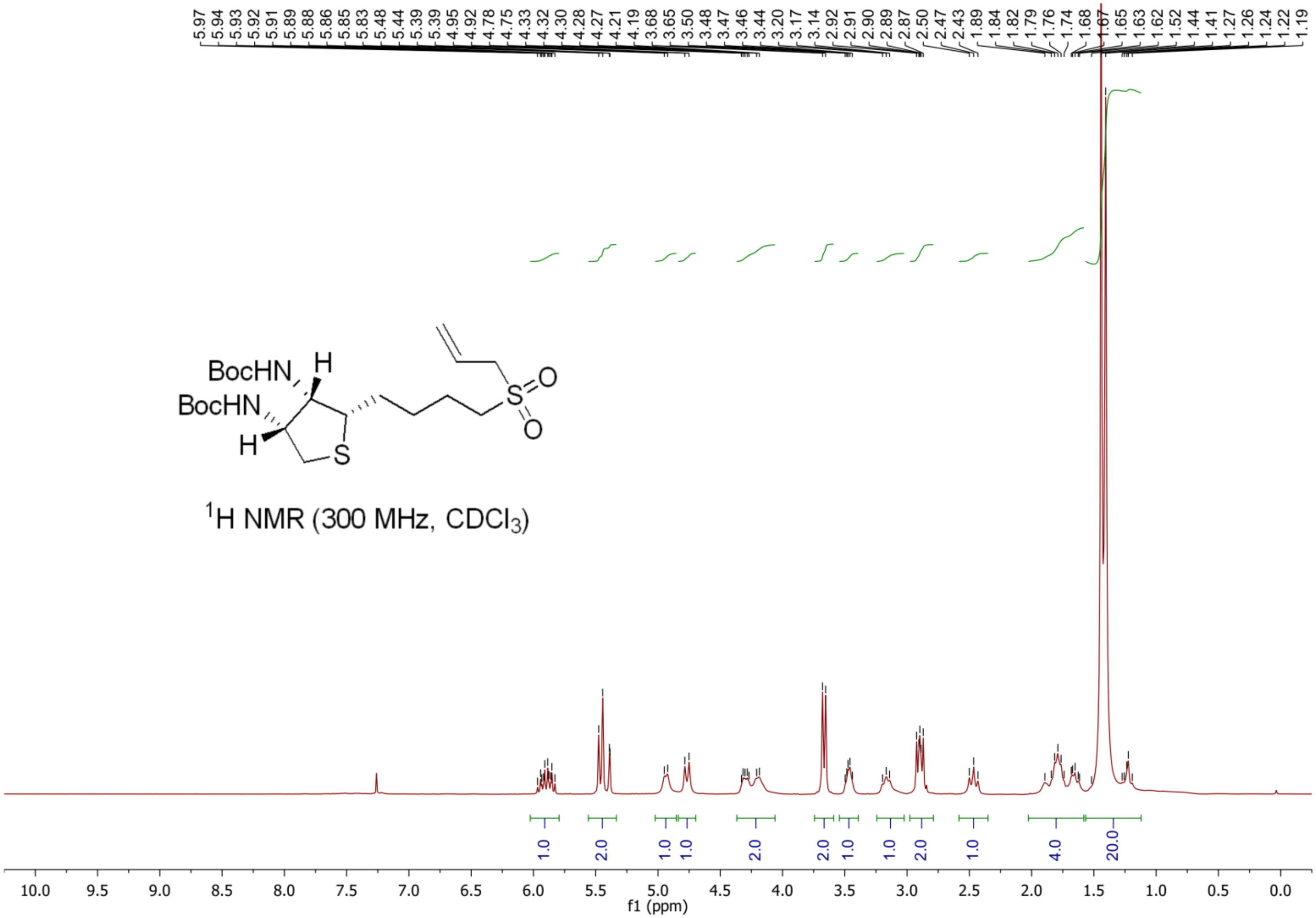

Go back to table of contents

Di-*tert*-butyl ((2*S*,3*S*,4*R*)-2-(4-(allylsulfonyl)butyl)tetrahydrothiophene-3,4-diyl)dicarbamate (1w)

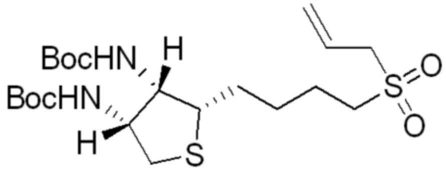

^{13}C NMR (75 MHz, $CDCl_3$)

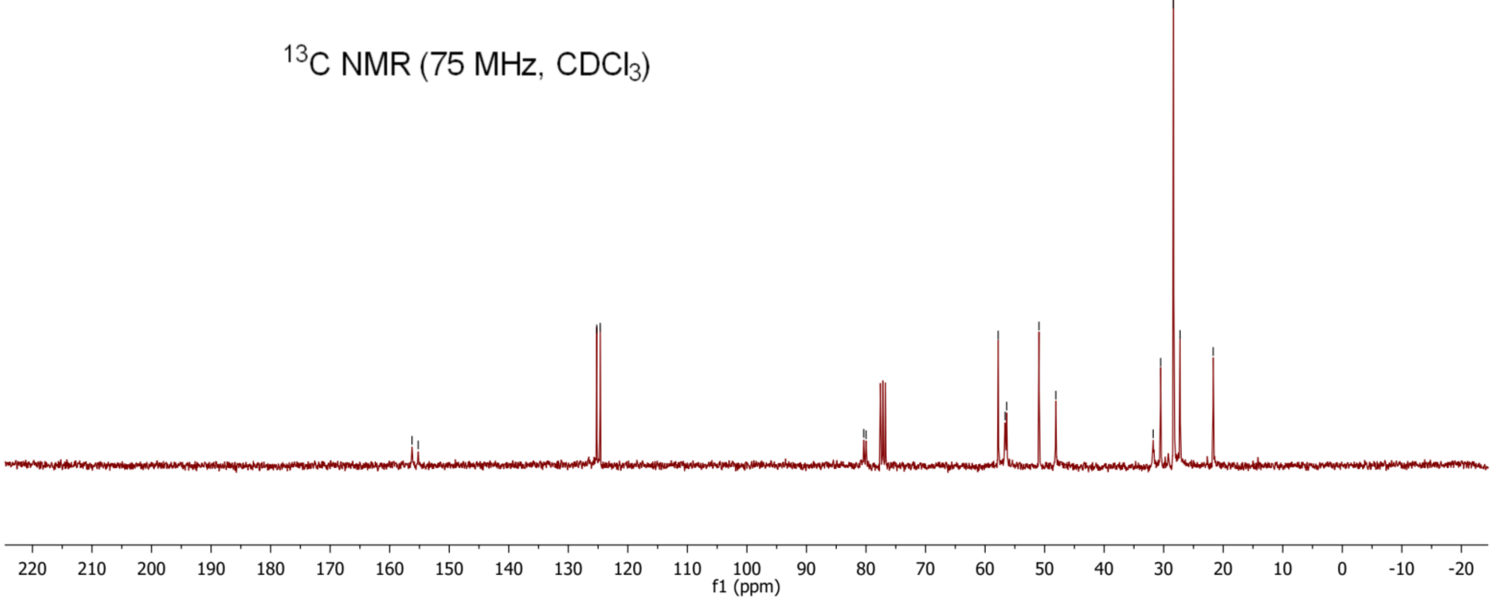

Go back to table of contents

tert-Butyl (_S_)-4-(allylsulfonyl)-2-((_tert_-butoxycarbonyl)amino)butanoate (1u)

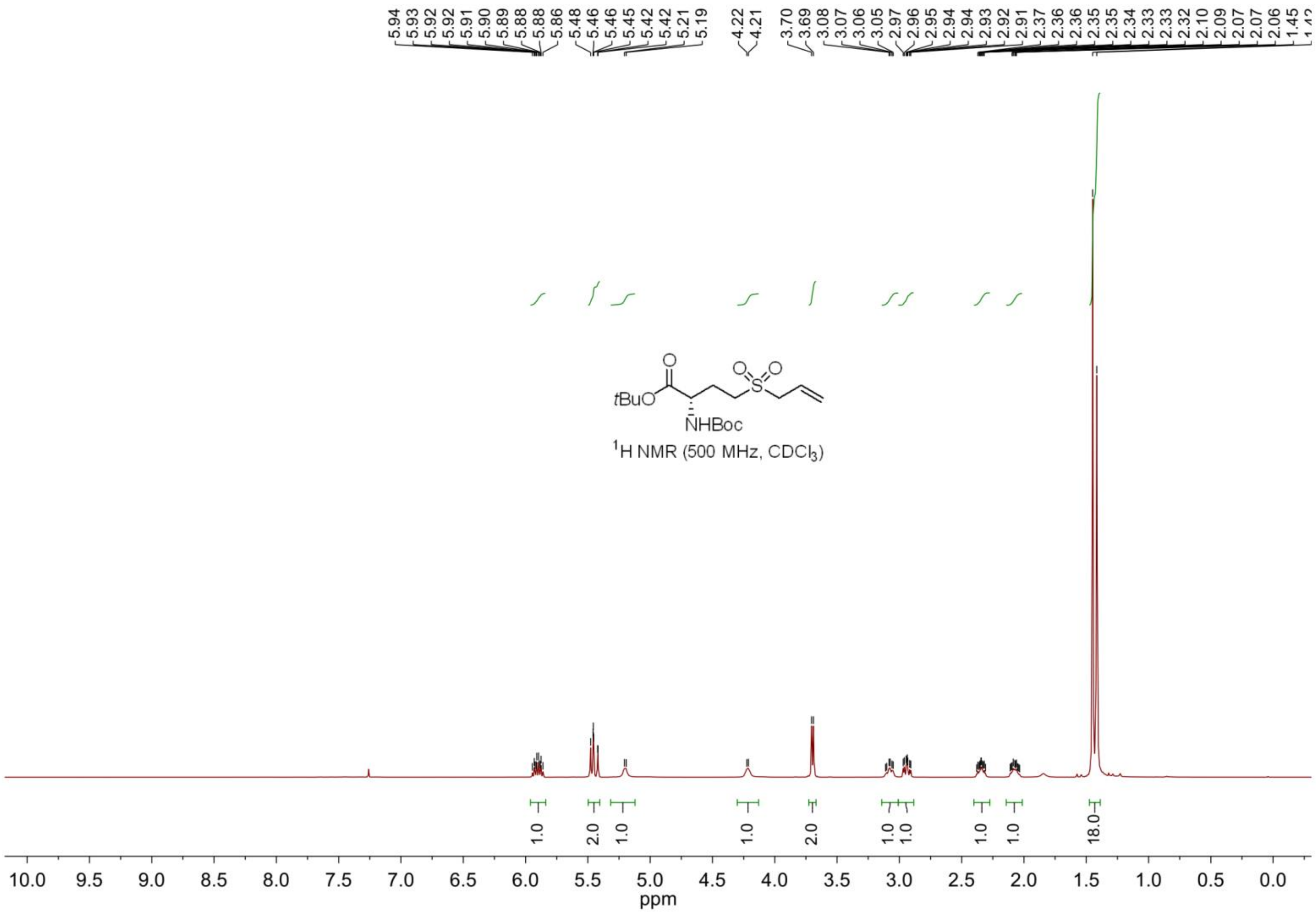

Go back to table of contents

tert-Butyl (S)-4-(allylsulfonyl)-2-((tert-butoxycarbonyl)amino)butanoate (1u)

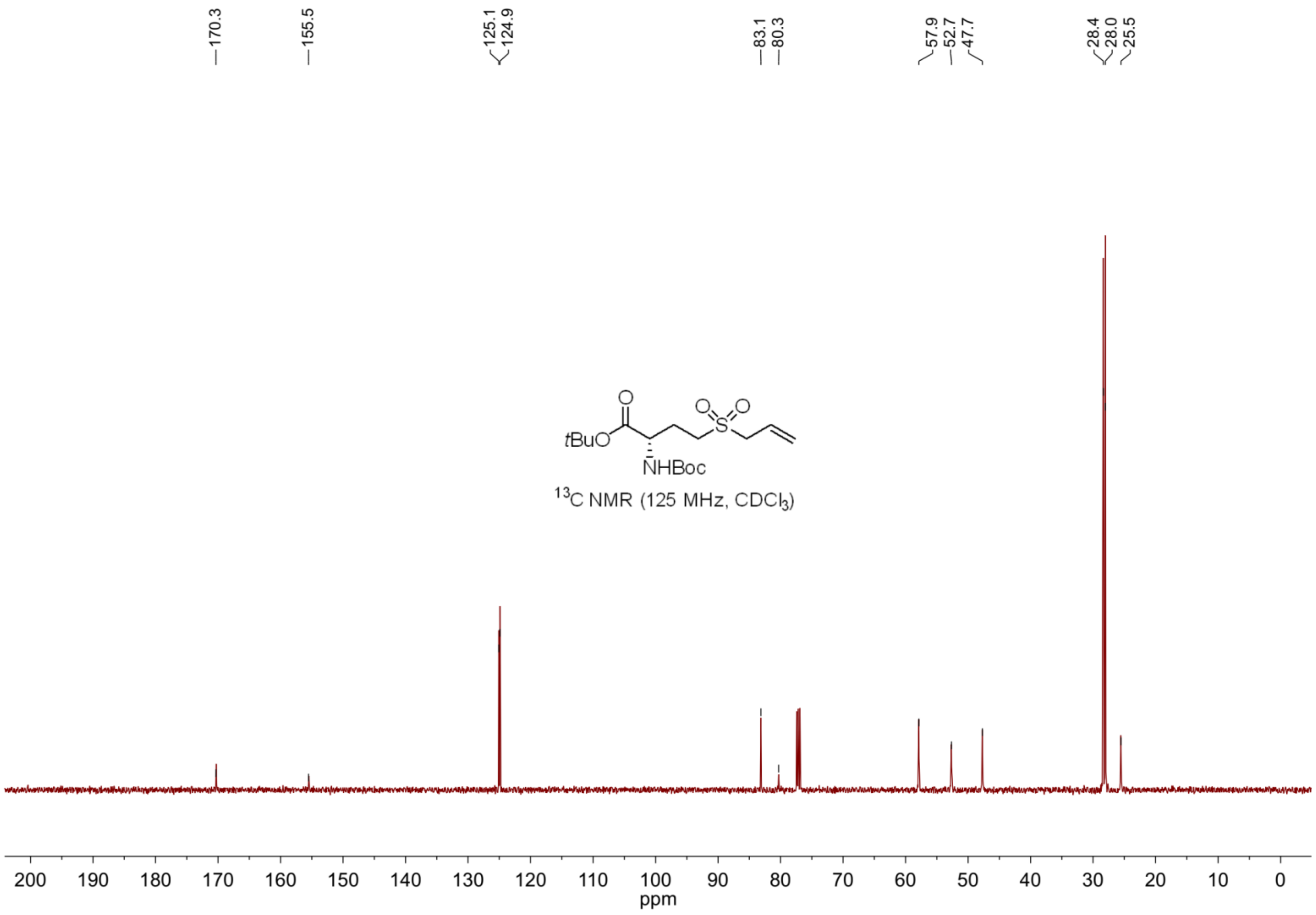

Go back to table of contents

15-(Allylsulfonyl)pentadecane-1,7,8-triyl triacetate (1v)

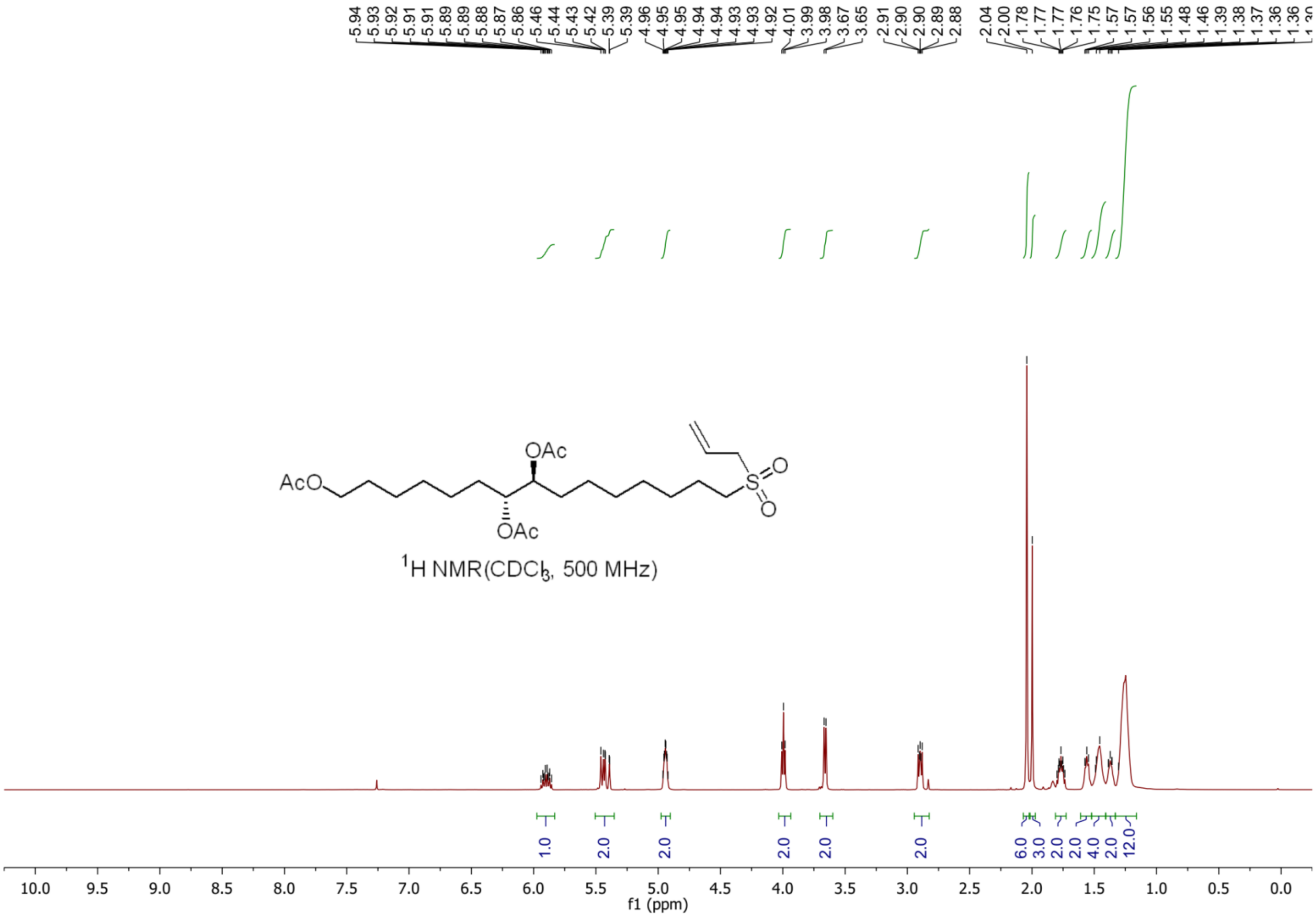

Go back to table of contents

15-(Allylsulfonyl)pentadecane-1,7,8-triyl triacetate (1v)

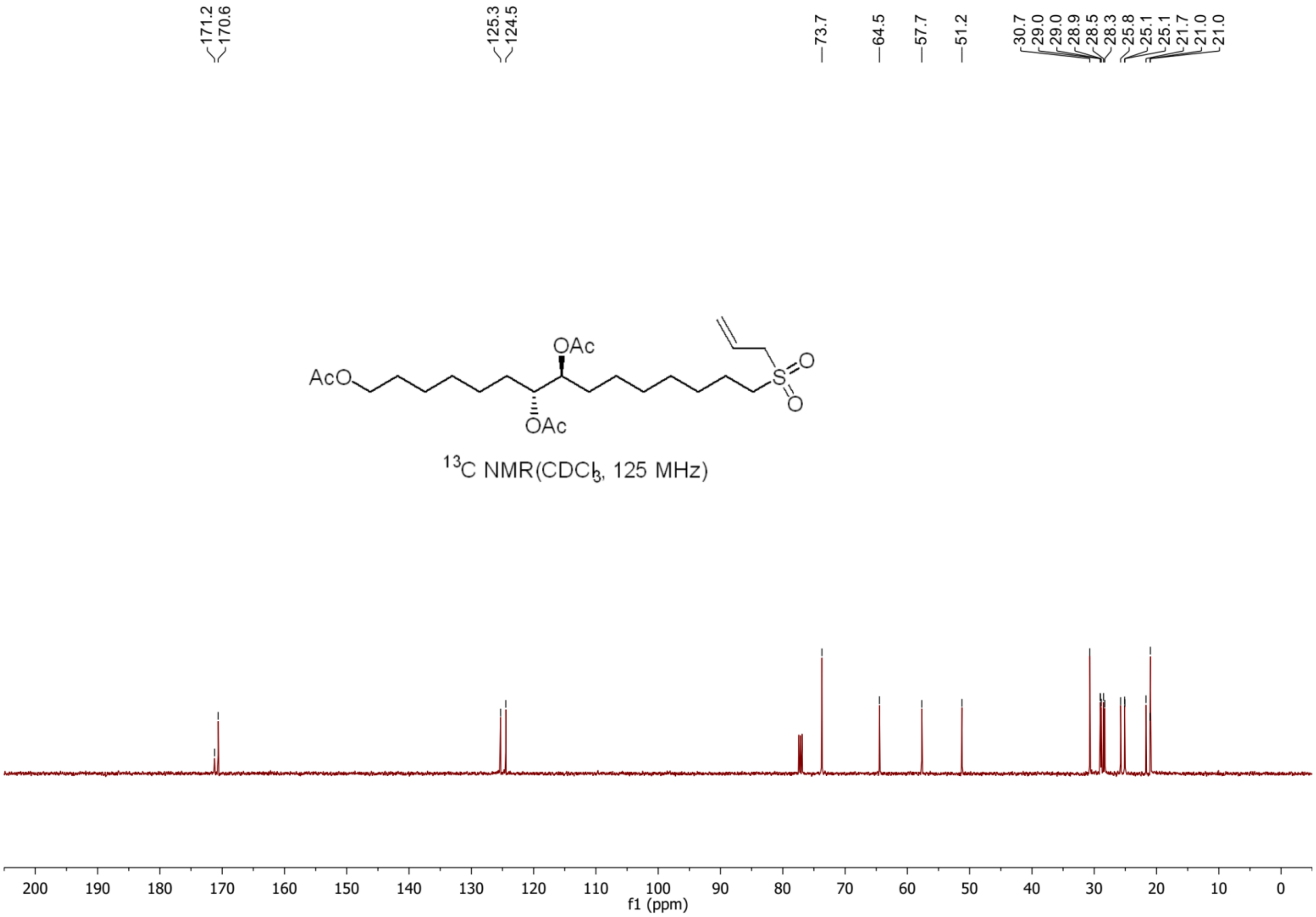

Go back to table of contents

Di-*tert*-butyl ((2*S*,3*S*,4*R*)-2-(4-(allylsulfonyl)butyl)tetrahydrothiophene-3,4-diyl)dicarbamate (1w)

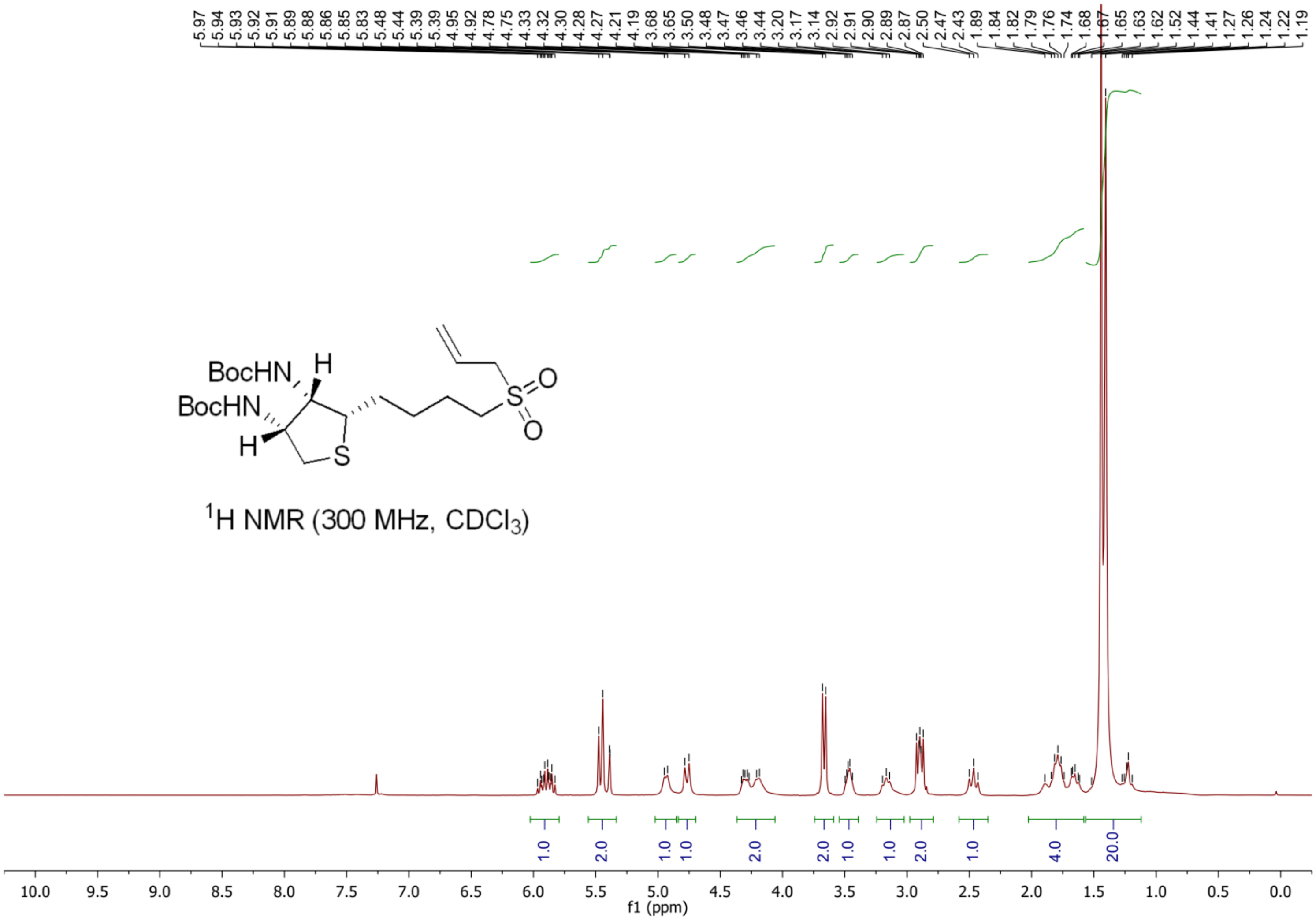

Go back to table of contents

Di-*tert*-butyl ((2*S*,3*S*,4*R*)-2-(4-(allylsulfonyl)butyl)tetrahydrothiophene-3,4-diyl)dicarbamate (1w)

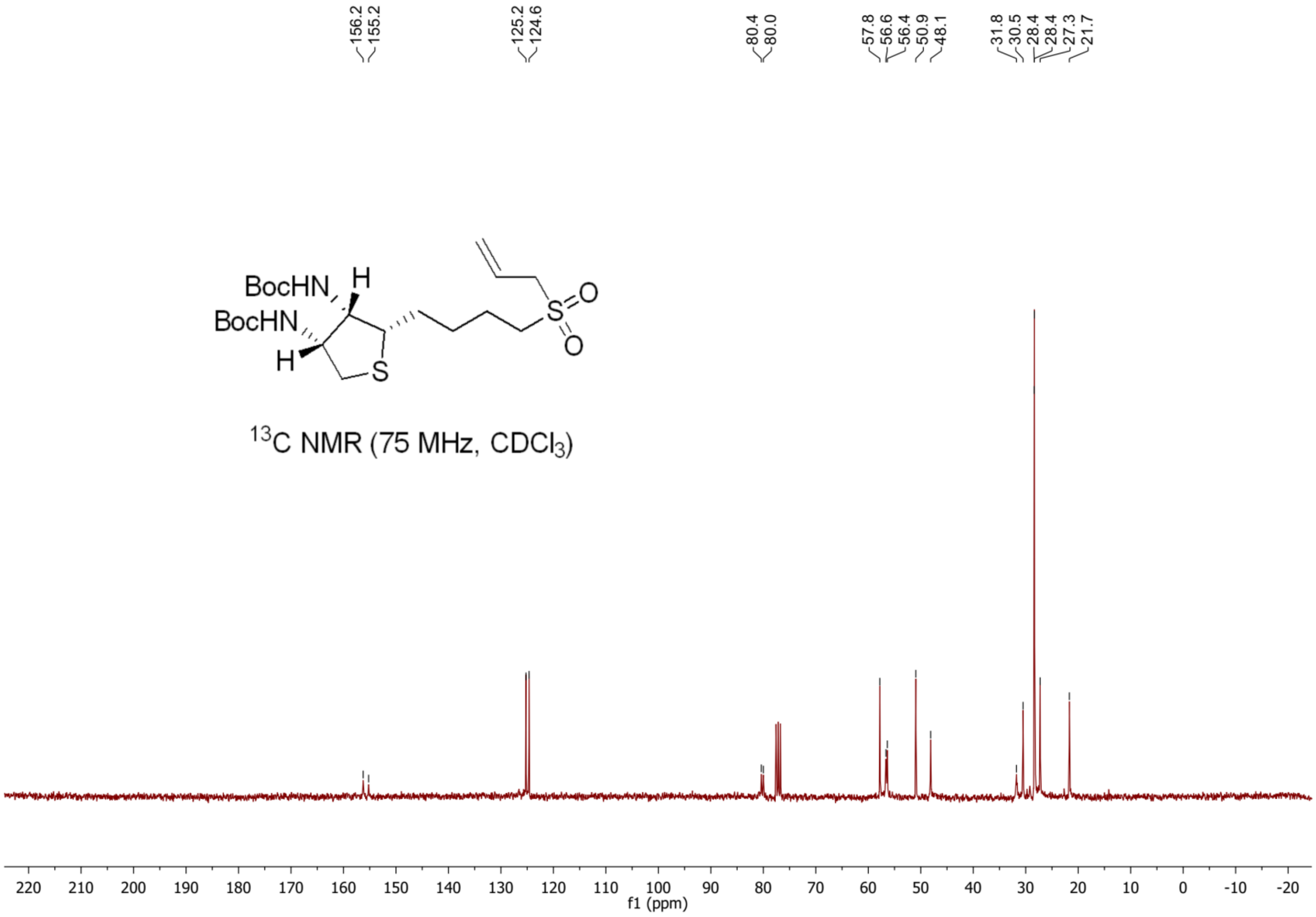

Go back to table of contents

(2*R*,4a*S*,6a*S*,6b*R*,10*S*,12a*S*,14b*R*)-2-(Allylsulfonyl)-10-hydroxy-2,4a,6a,6b,9,9,12a-heptamethyl-1,3,4,4a,5,6,6a,6b,7,8,8a,9,10,11,12,12a,12b,14b-octadecahydropicen-13(2*H*)-one (1x)

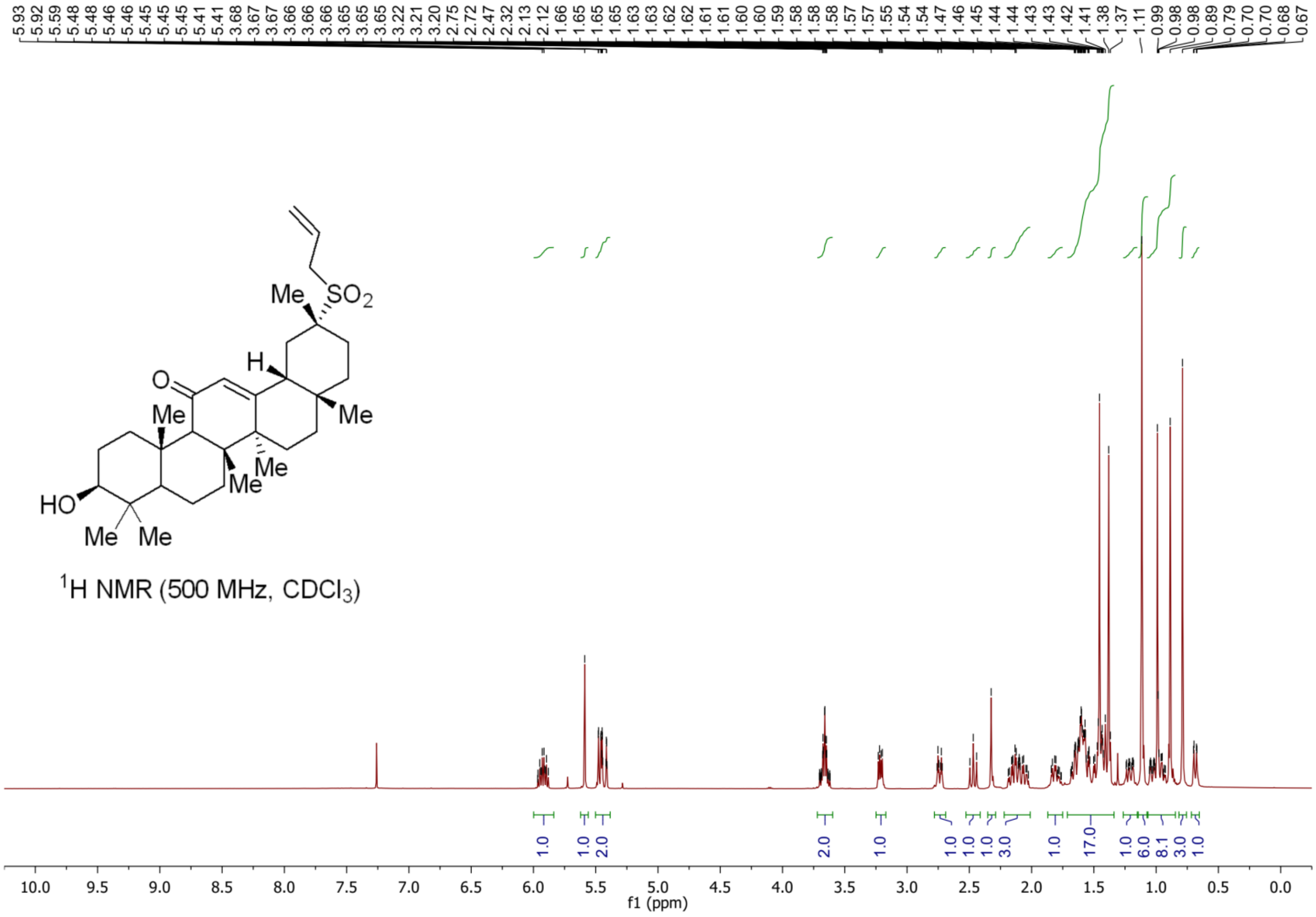

Go back to table of contents

(2*R*,4a*S*,6a*S*,6b*R*,10*S*,12a*S*,14b*R*)-2-(Allylsulfonyl)-10-hydroxy-2,4a,6a,6b,9,9,12a-heptamethyl-1,3,4,4a,5,6,6a,6b,7,8,8a,9,10,11,12,12a,12b,14b-octadecahydropicen-13(2*H*)-one (1x)

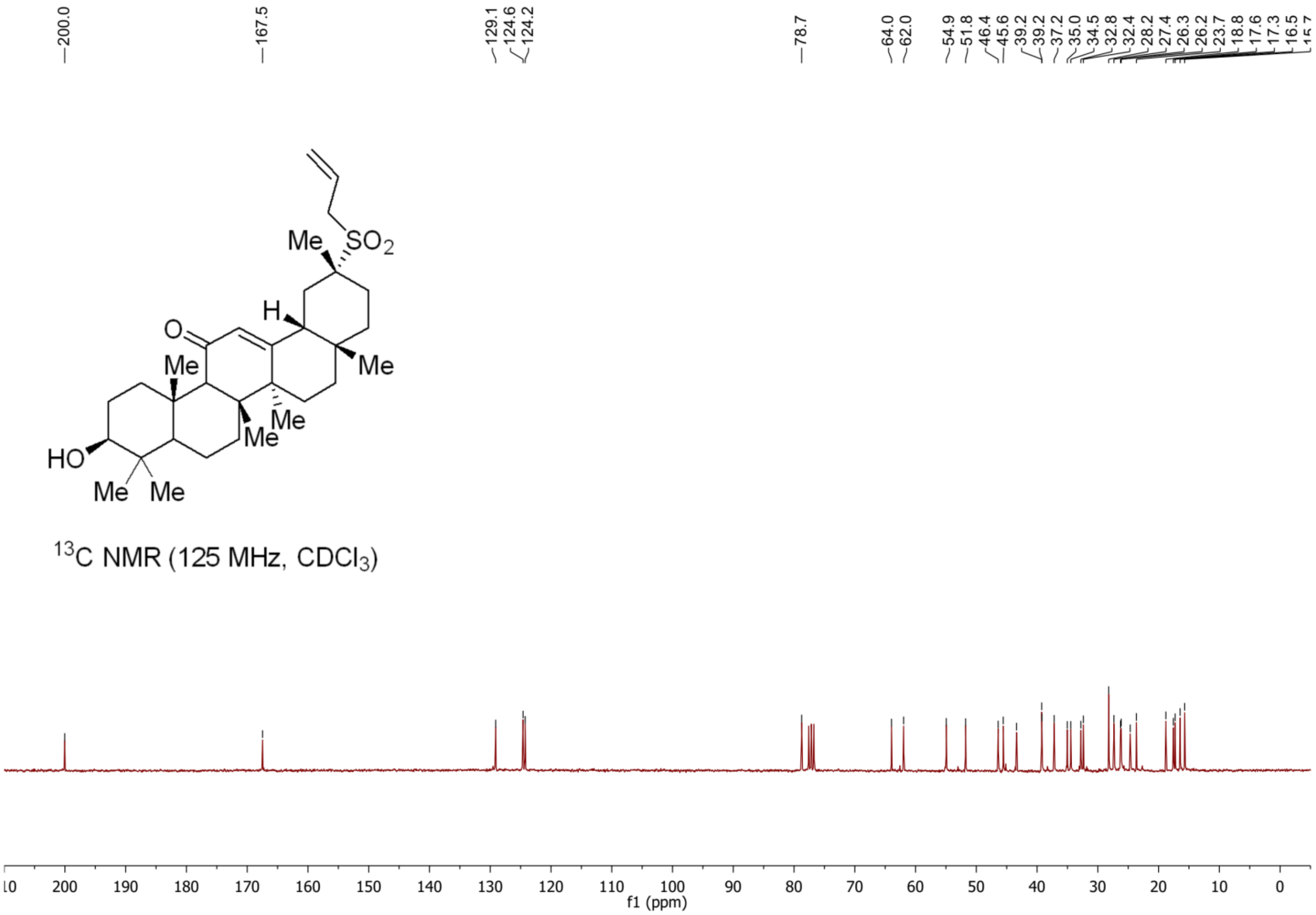

Go back to table of contents

(3*R*,7*R*,8*R*,9*S*,10*S*,13*R*,14*S*,17*R*)-17-((*R*)-4-(Allylsulfonyl)butan-2-yl)-10,13-dimethylhexadecahydro-1*H*-cyclopenta[*a*]phenanthrene-3,7-diol (1y)

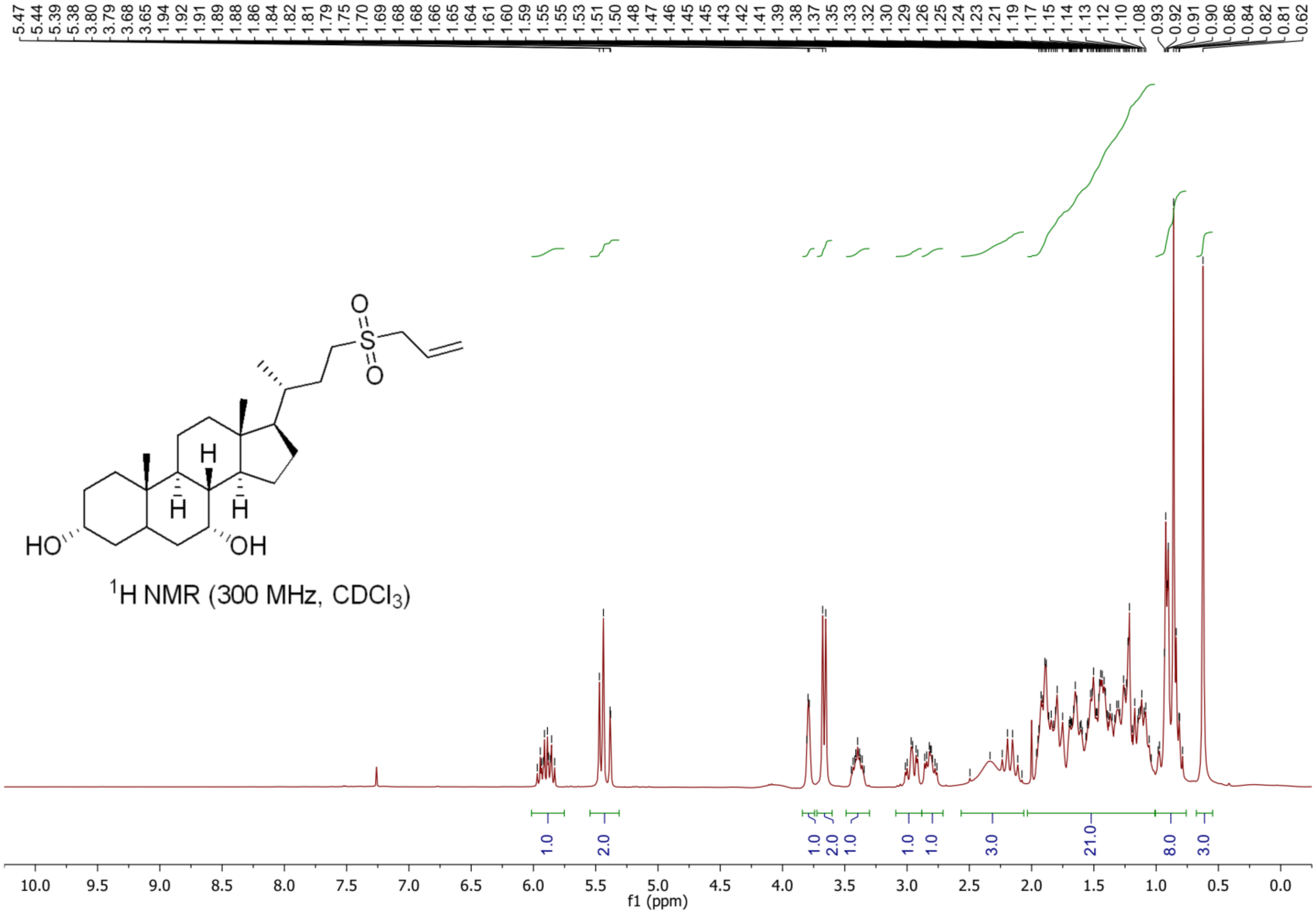

Go back to table of contents

(3*R*,7*R*,8*R*,9*S*,10*S*,13*R*,14*S*,17*R*)-17-((*R*)-4-(Allylsulfonyl)butan-2-yl)-10,13-dimethylhexadecahydro-1*H*-cyclopenta[*a*]phenanthrene-

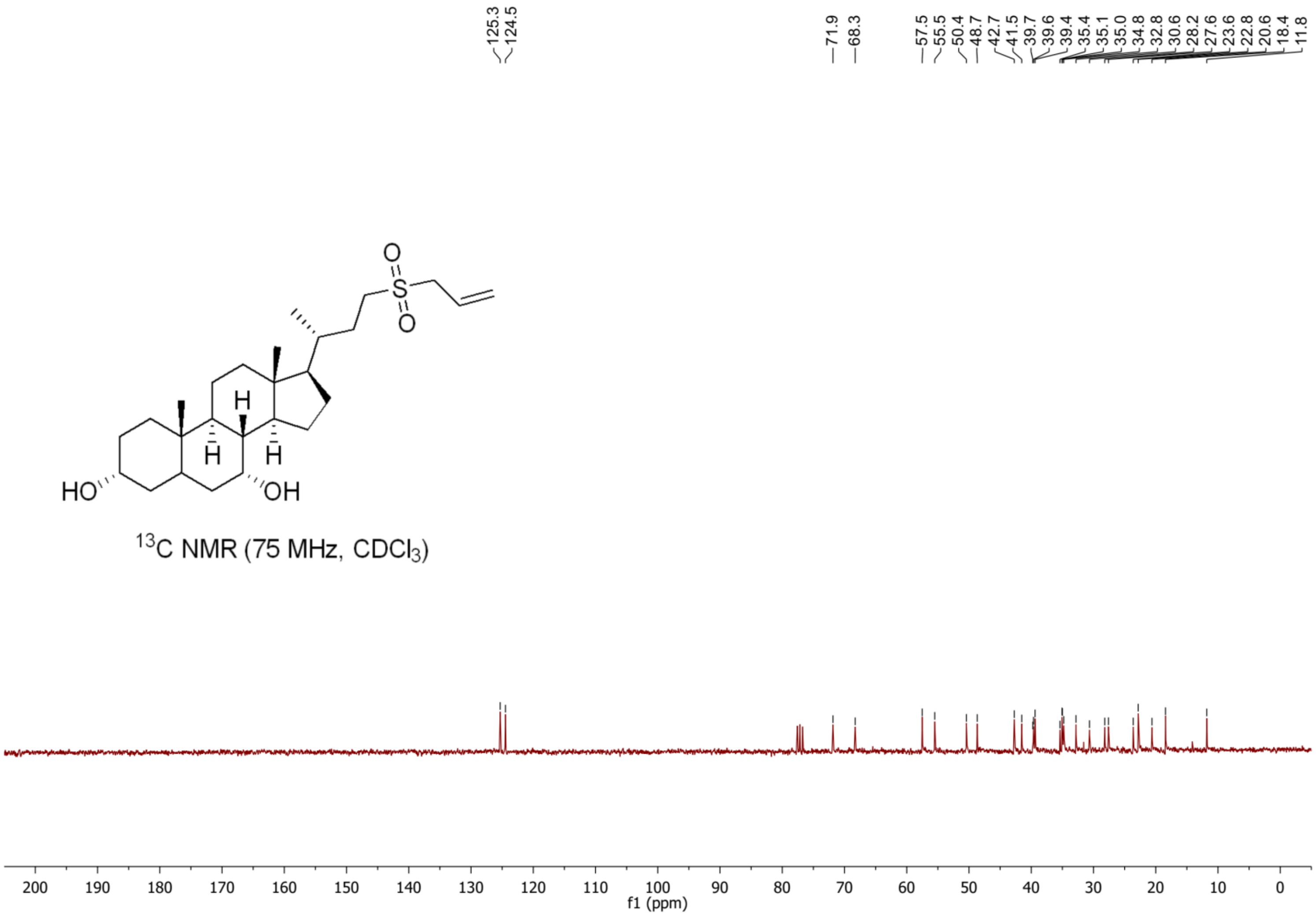

3,7-diol (1y)

Go back to table of contents

((1*S*,2*S*,4a*R*,4b*R*,7*S*,9a*S*,10*S*,10a*R*)-10-(Allylsulfonyl)-1-methyl-8-methylene-13-oxo-1,2,5,6,8,9,10,10a-octahydro-4a,1-(epoxymethano)-7,9a-methanobenzo[*a*]azulene-2,7(4b*H*)-diyl diacetate (1z)

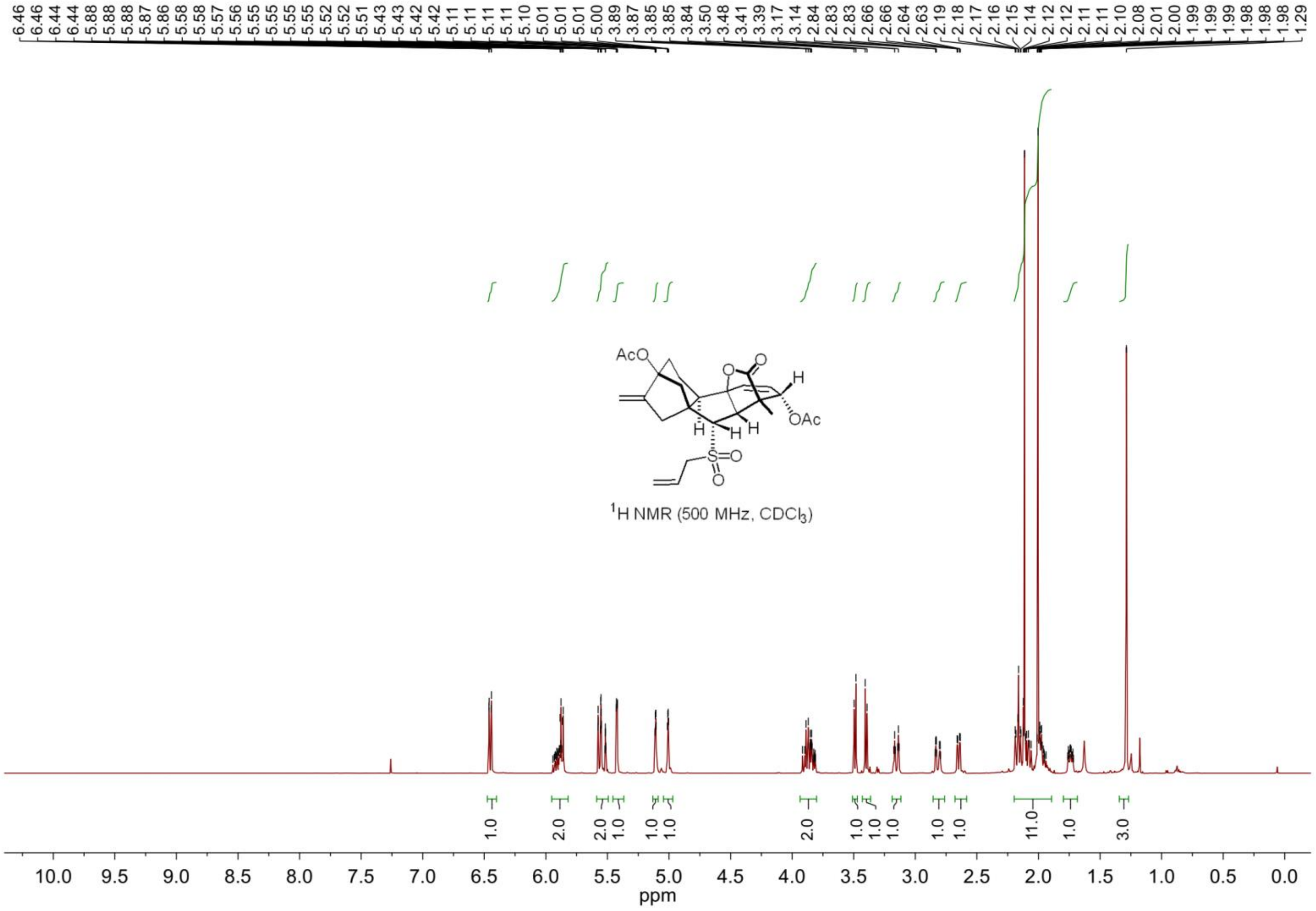

Go back to table of contents

((1*S*,2*S*,4a*R*,4b*R*,7*S*,9a*S*,10*S*,10a*R*)-10-(Allylsulfonyl)-1-methyl-8-methylene-13-oxo-1,2,5,6,8,9,10,10a-octahydro-4a,1-(epoxymethano)-7,9a-methanobenzo[*a*]azulene-2,7(4b*H*)-diyl diacetate (1z)

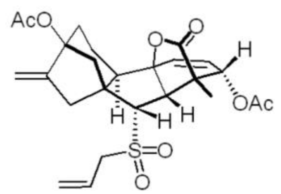

^{13}C NMR (125 MHz, $CDCl_3$)

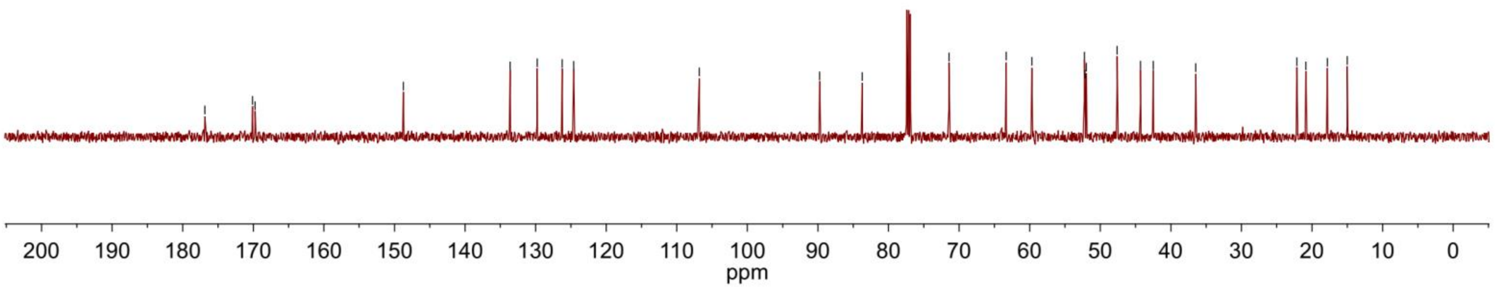

Go back to table of contents

1-((2-Bromoallyl)sulfonyl)pentadecane (2a)

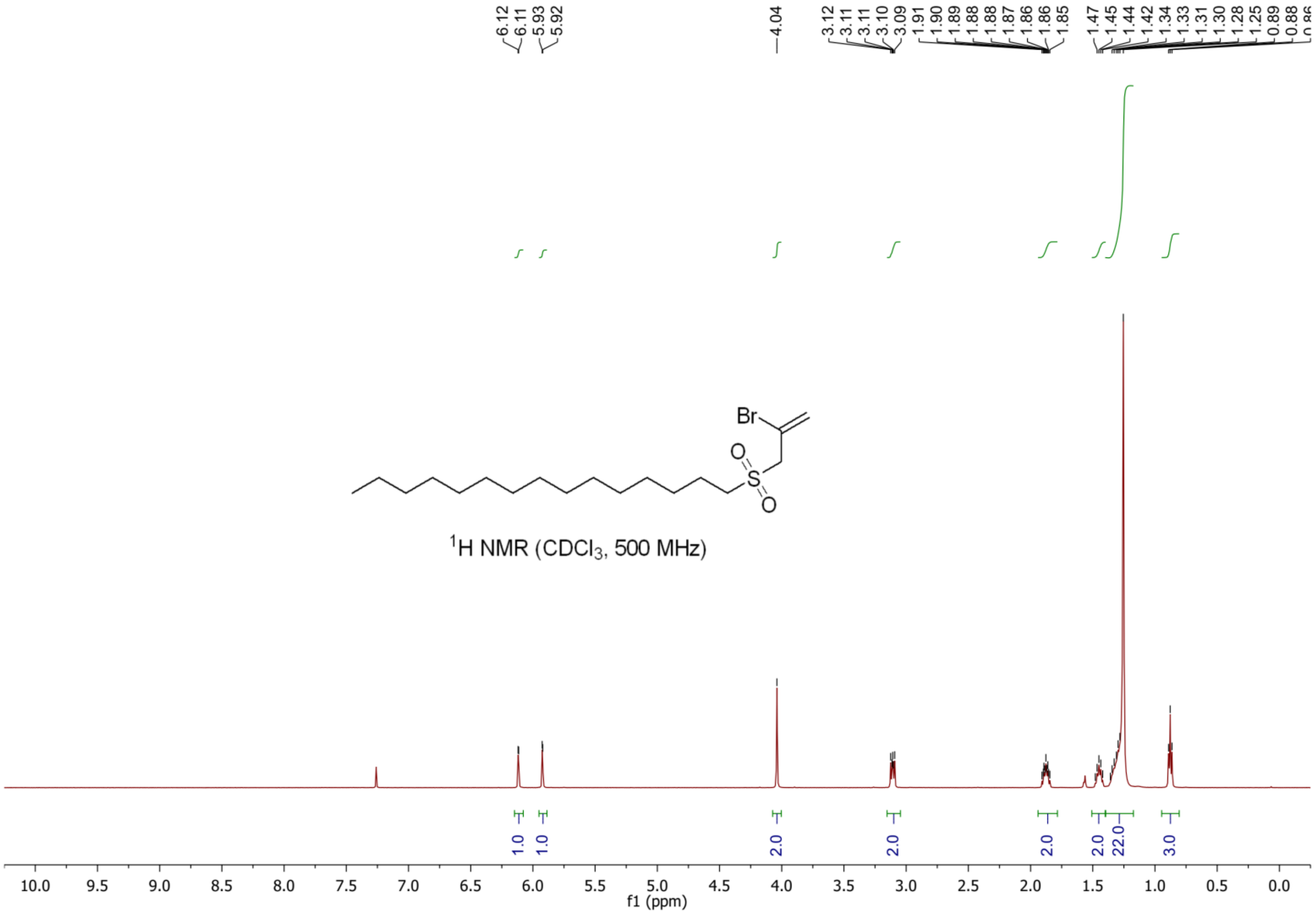

Go back to table of contents

1-((2-Bromoallyl)sulfonyl)pentadecane (2a)

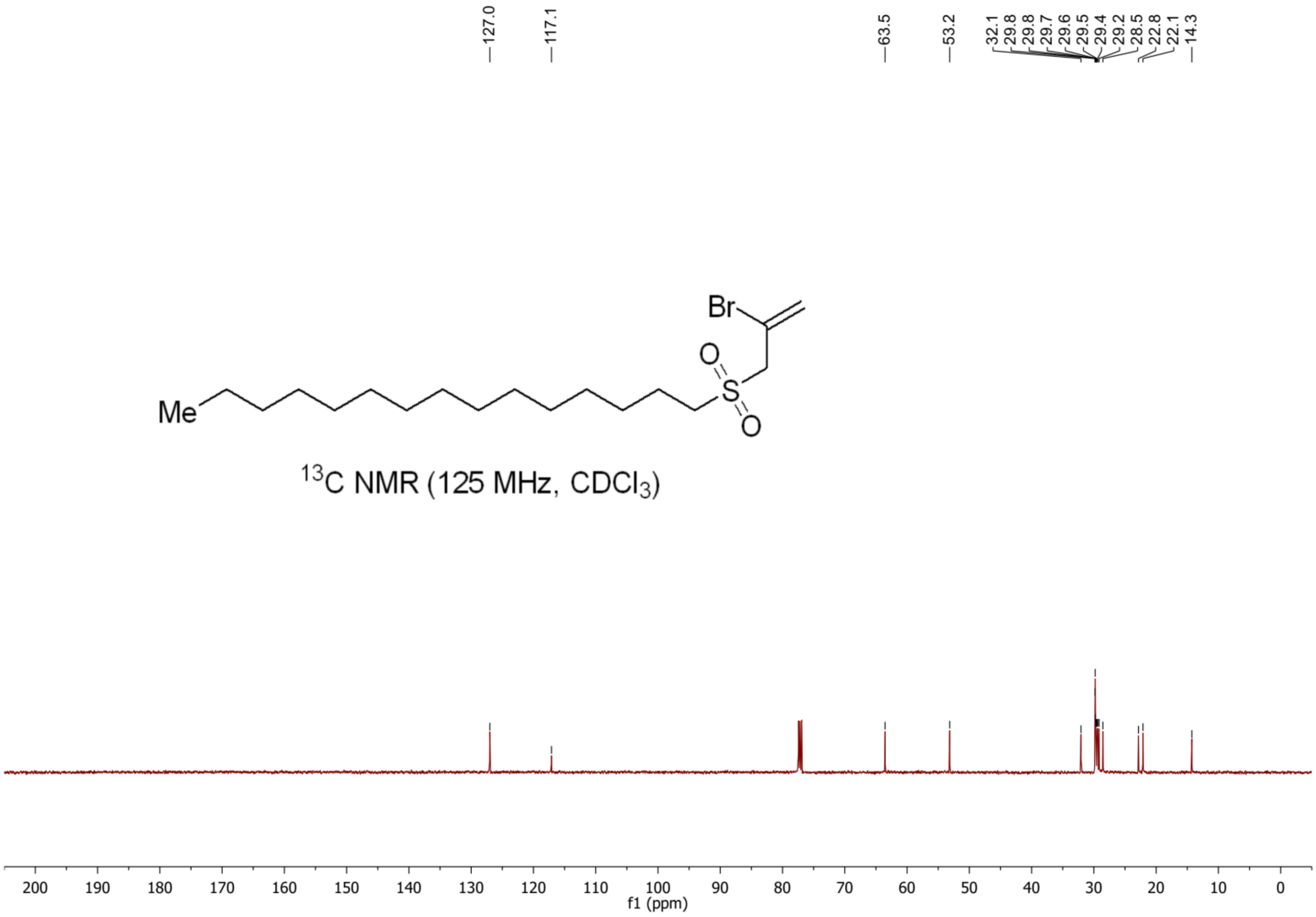

Go back to table of contents

1-((2-Methylallyl)sulfonyl)pentadecane (2b)

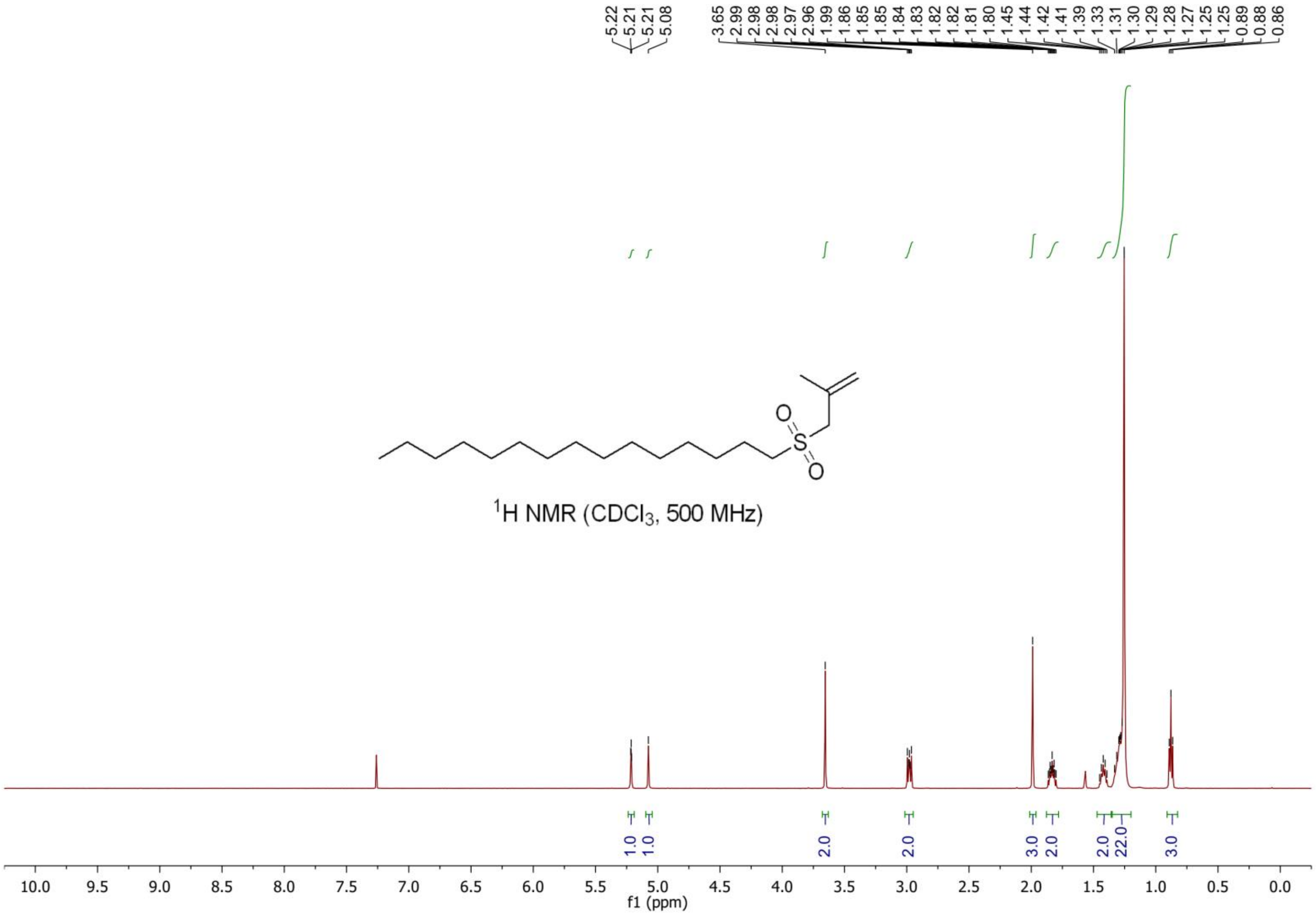

Go back to table of contents

1-((2-Methylallyl)sulfonyl)pentadecane (2b)

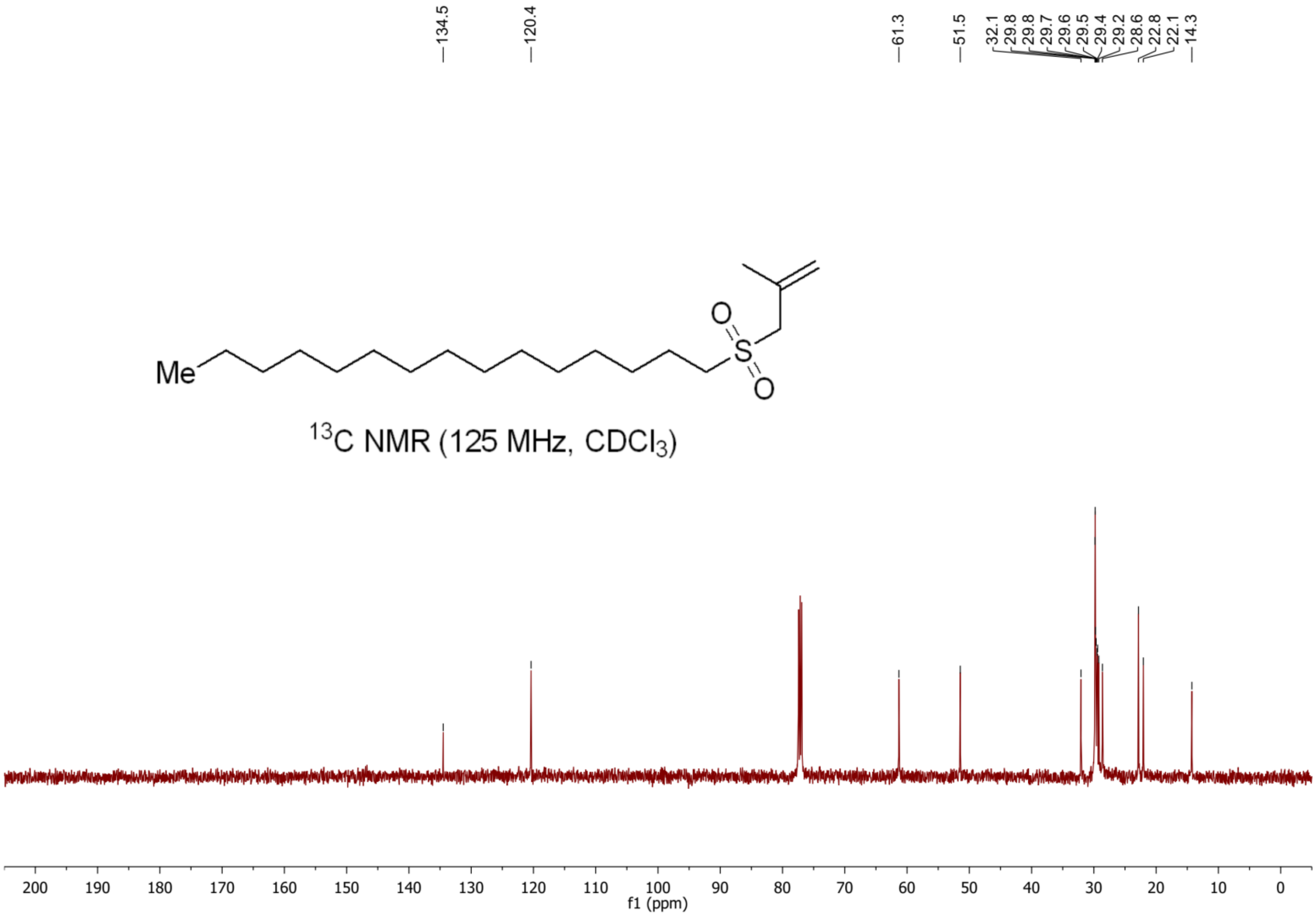

Go back to table of contents

1-Bromo-2-((cyclohexylsulfonyl)methyl)benzene (2c)

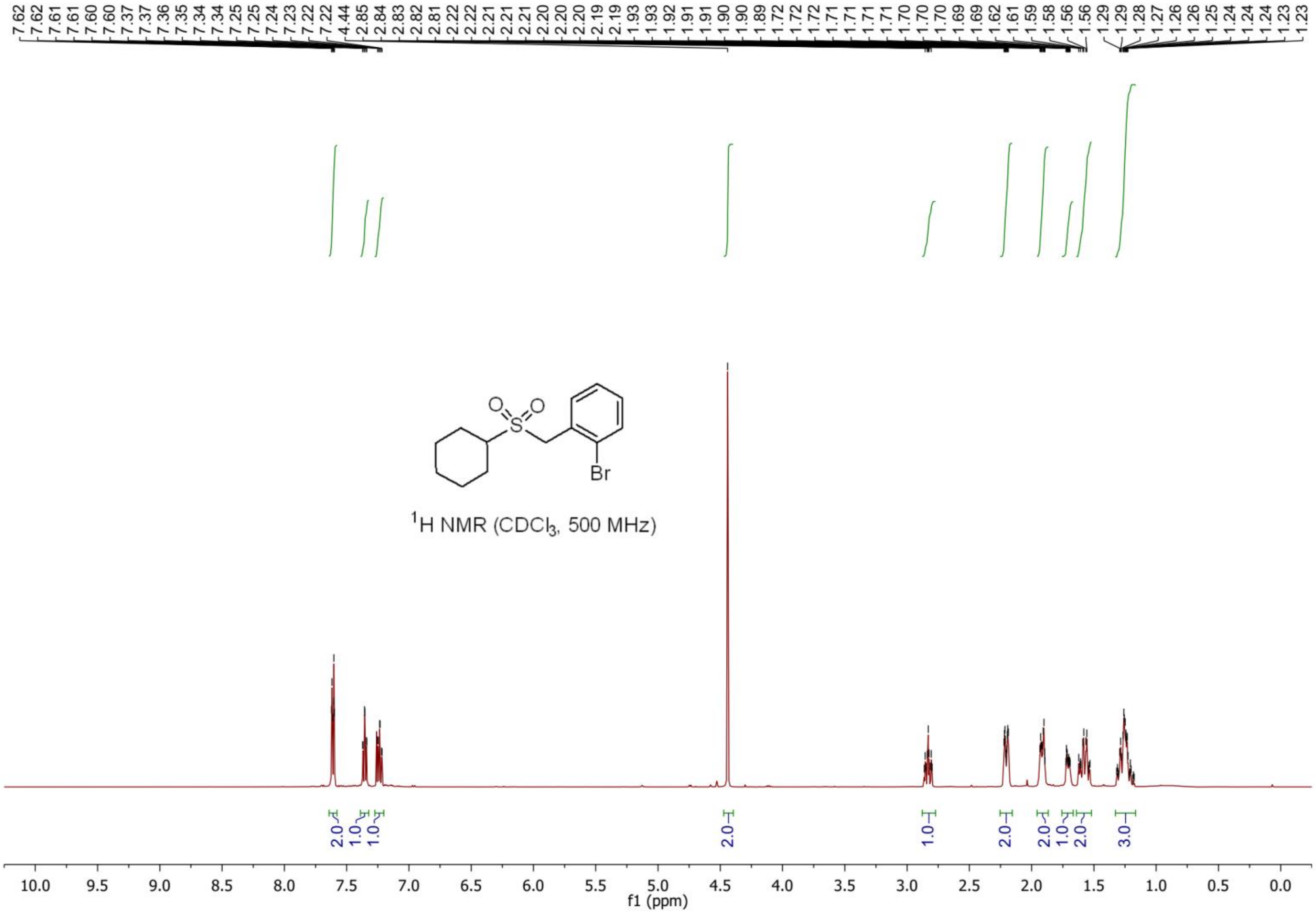

Go back to table of contents

1-Bromo-2-((cyclohexylsulfonyl)methyl)benzene (2c)

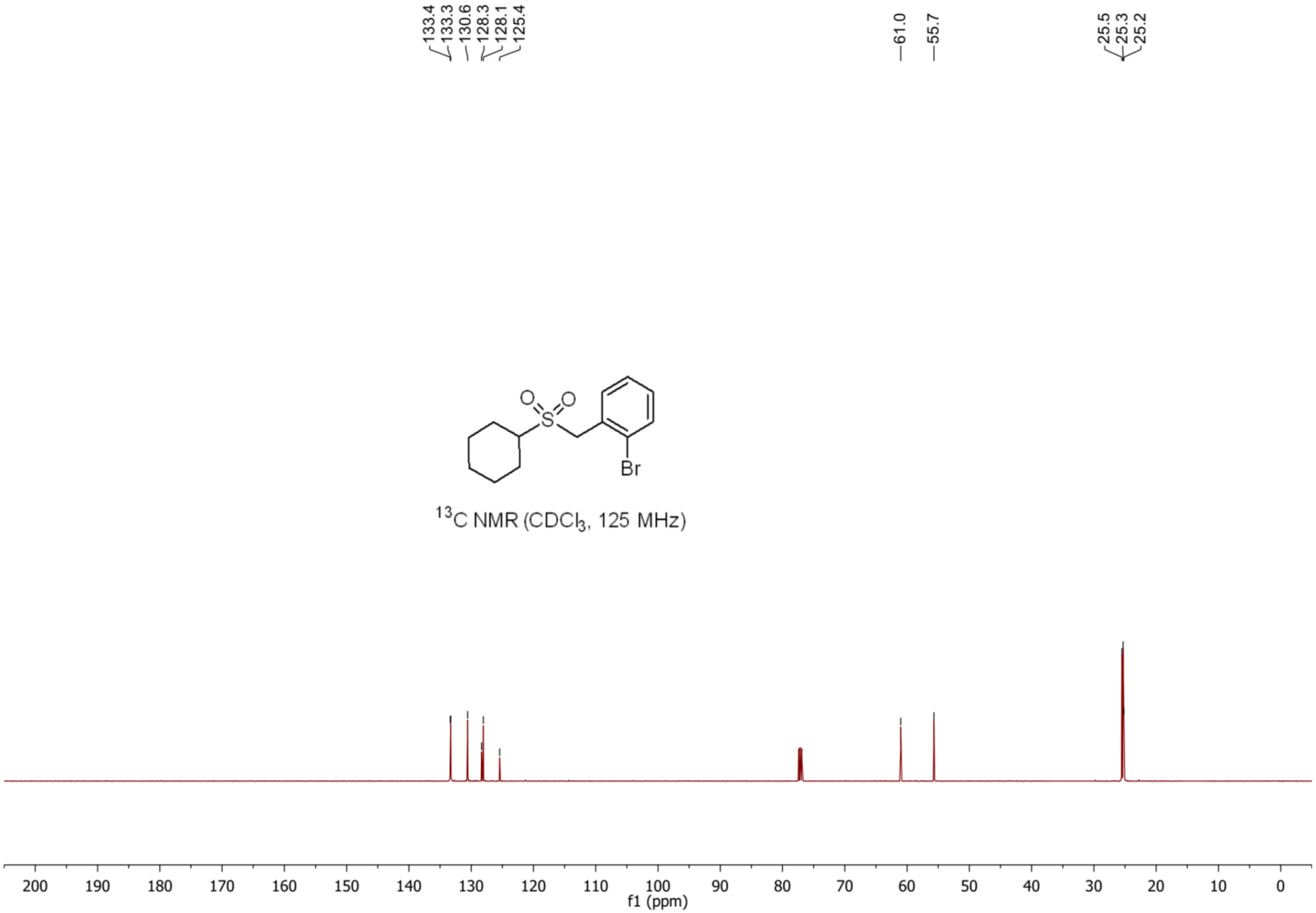

Go back to table of contents

1-Methyl-4-(((4-phenylbutyl)sulfonyl)methyl)benzene (2d)

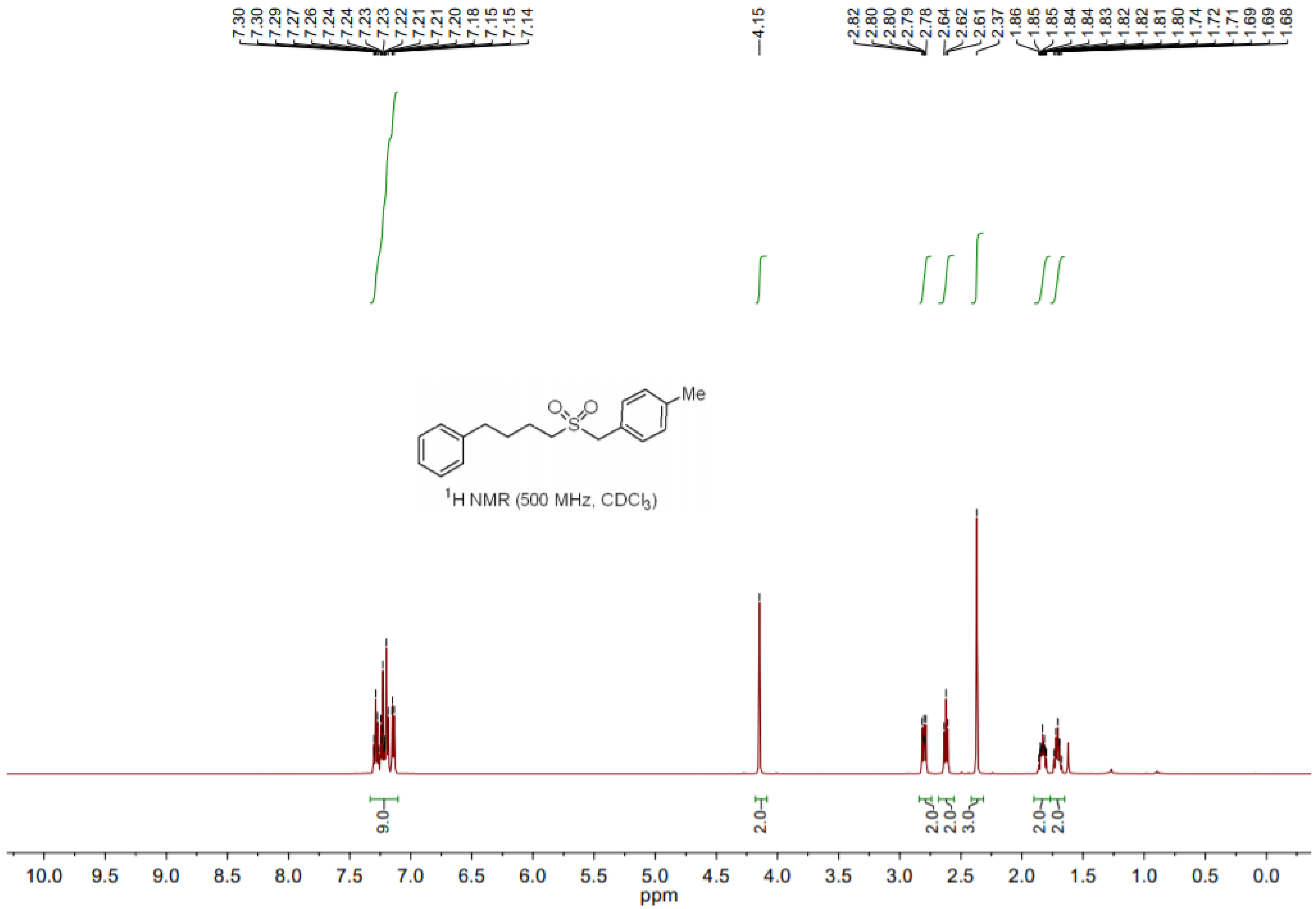

Go back to table of contents

1-Methyl-4-(((4-phenylbutyl)sulfonyl)methyl)benzene (2d)

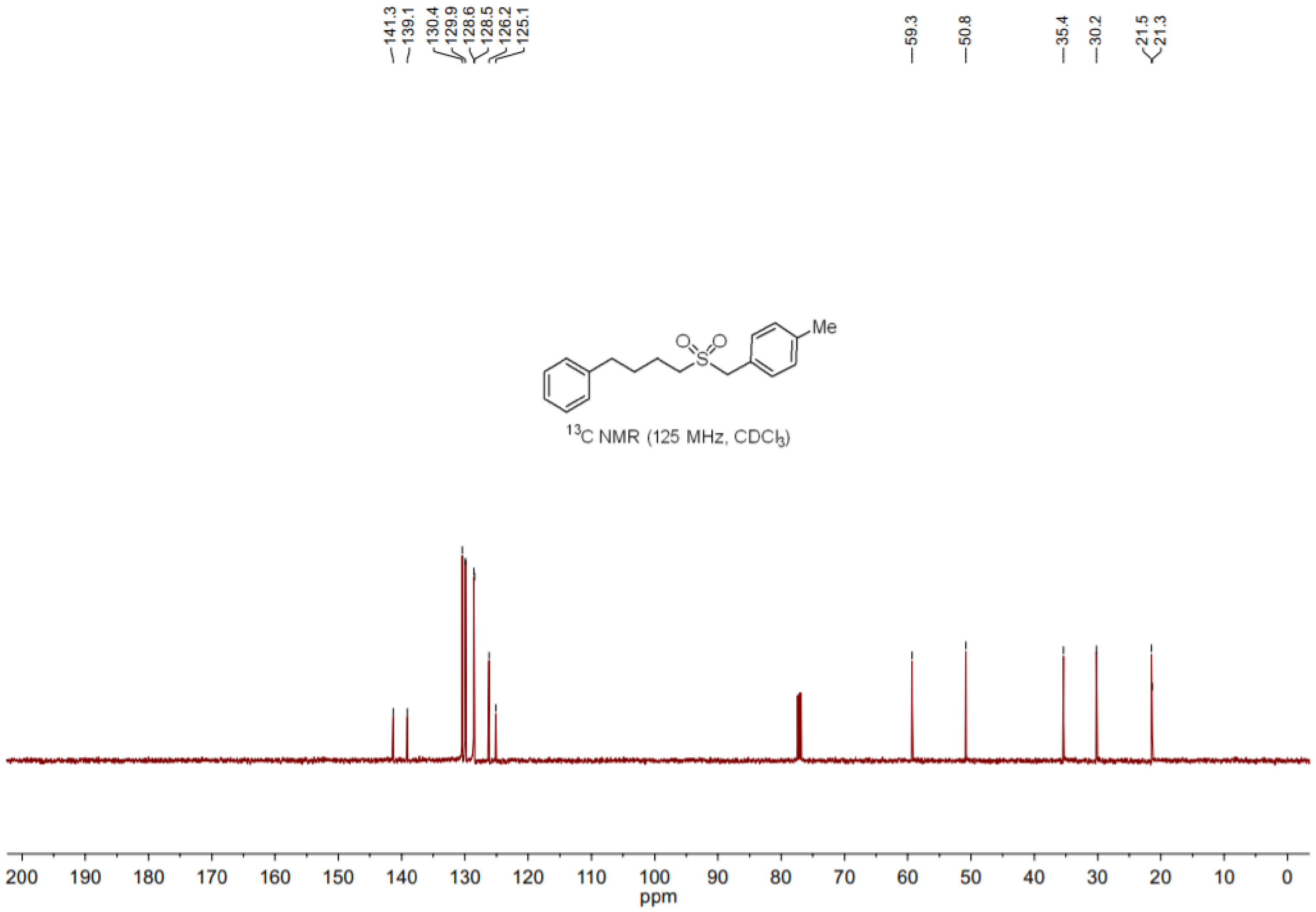

Go back to table of contents

4-(Methylsulfonyl)-1-phenylbutan-1-one (2e)

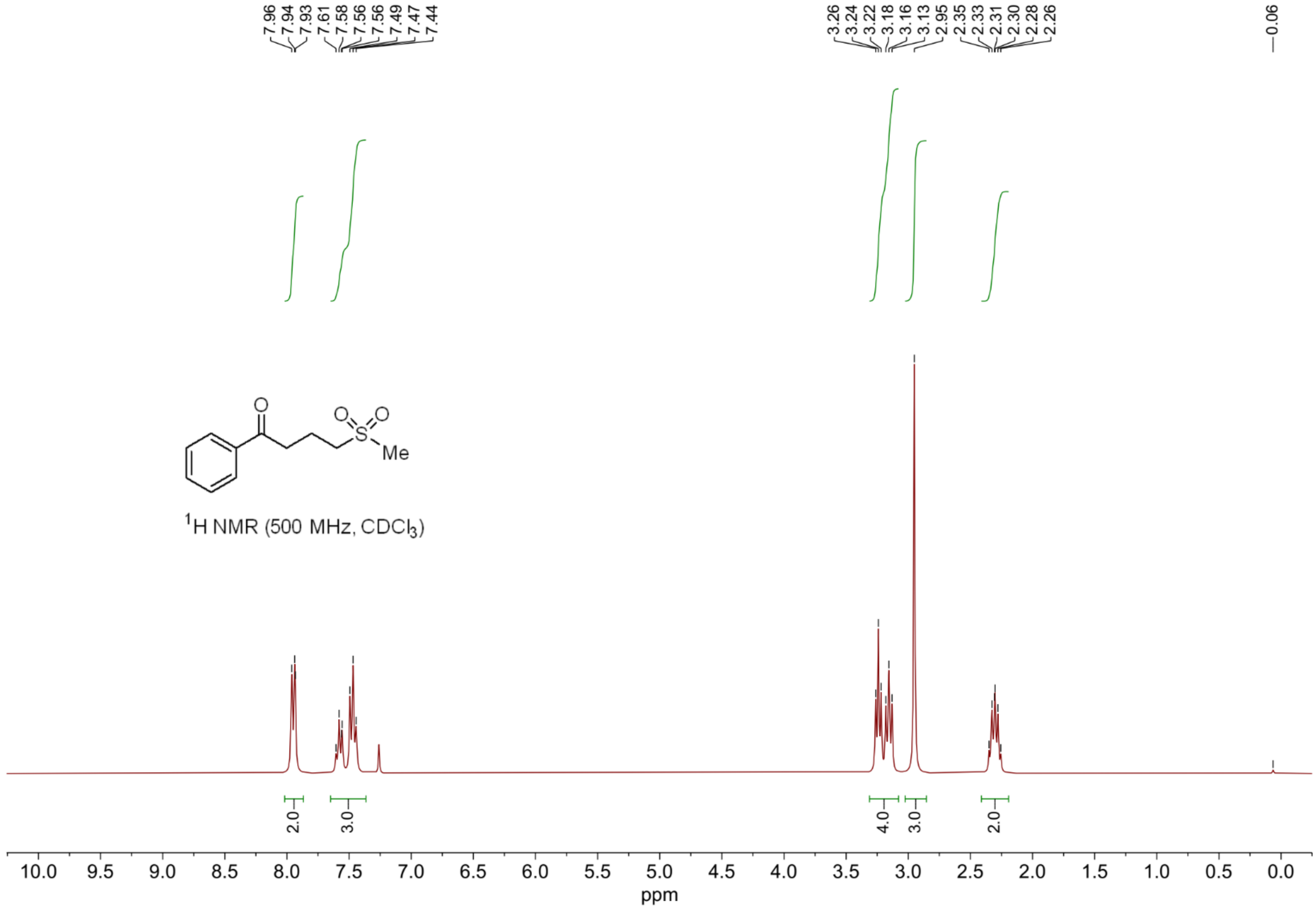

Go back to table of contents

4-(Methylsulfonyl)-1-phenylbutan-1-one (2e)

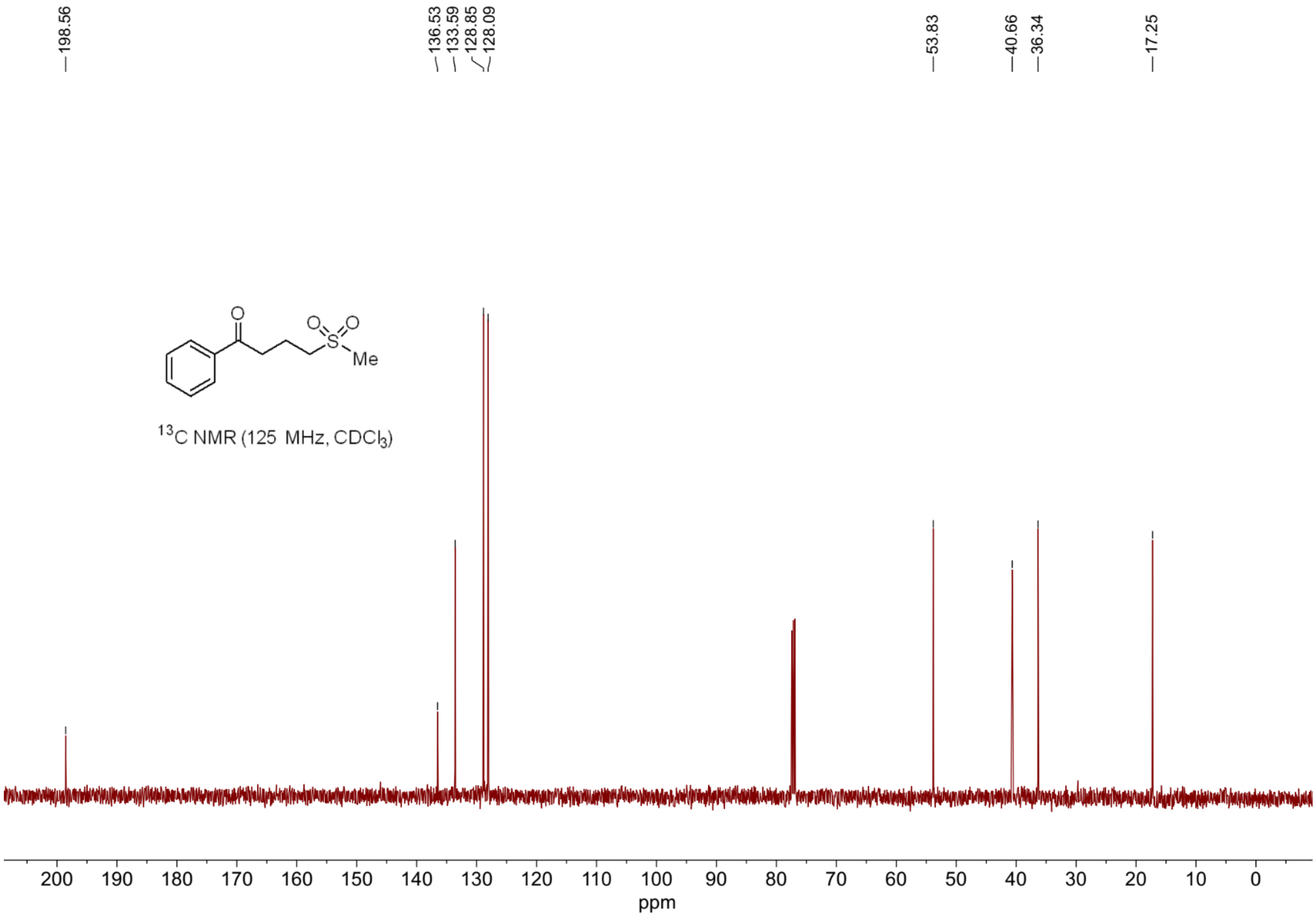

Go back to table of contents

1,4-Dimethyl-2-((4-methyl-4-(methylsulfonyl)pentyl)oxy)benzene (2f)

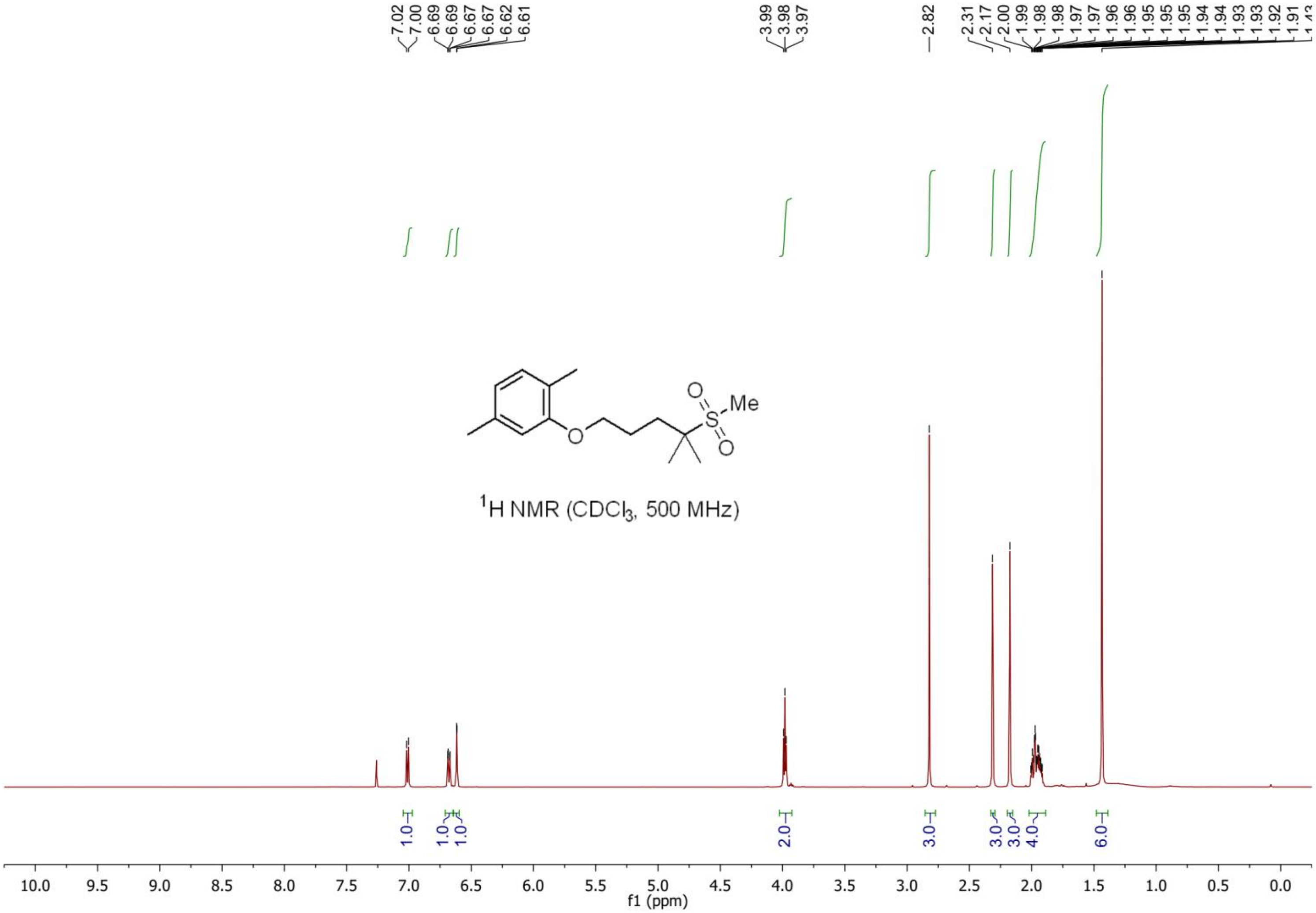

1,4-Dimethyl-2-((4-methyl-4-(methylsulfonyl)pentyl)oxy)benzene (2f)

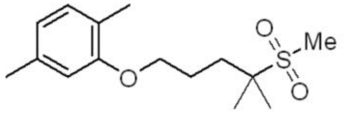

^{13}C NMR (CDCl$_3$, 125 MHz)

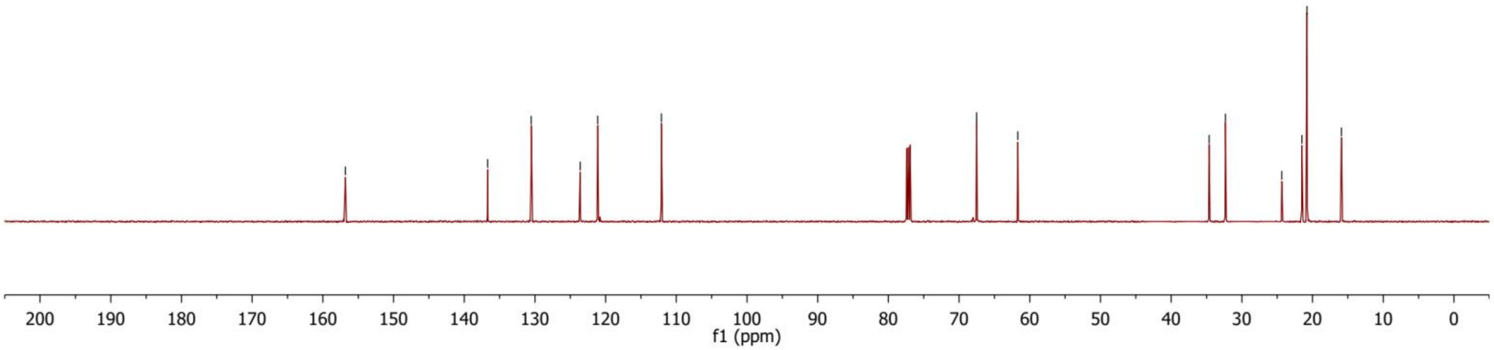

Go back to table of contents

Methyl (*tert*-butoxycarbonyl)(cyclohexylsulfonyl)-D-alaninate (2g)

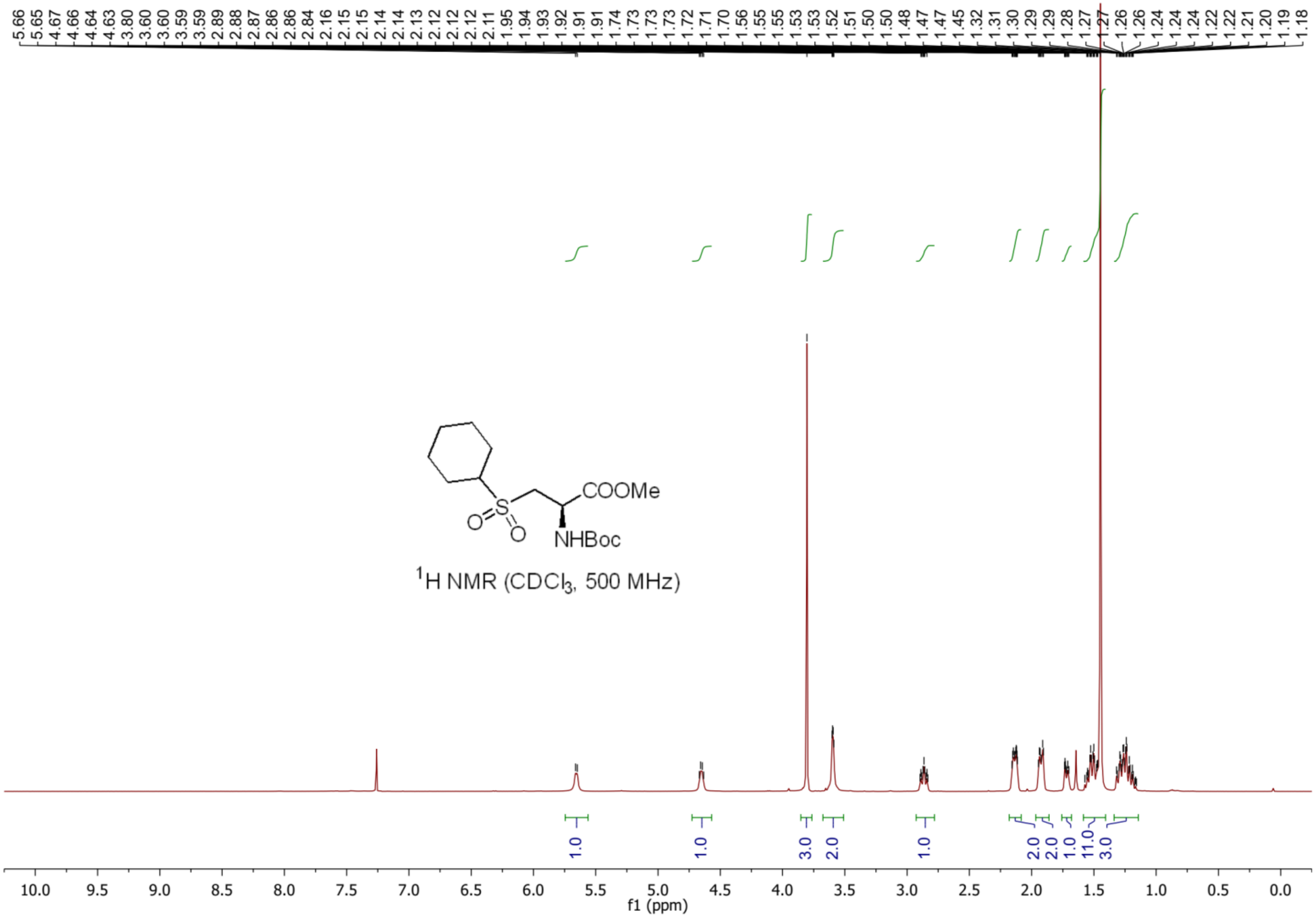

Go back to table of contents

Methyl (*tert*-butoxycarbonyl)(cyclohexylsulfonyl)-D-alaninate (2g)

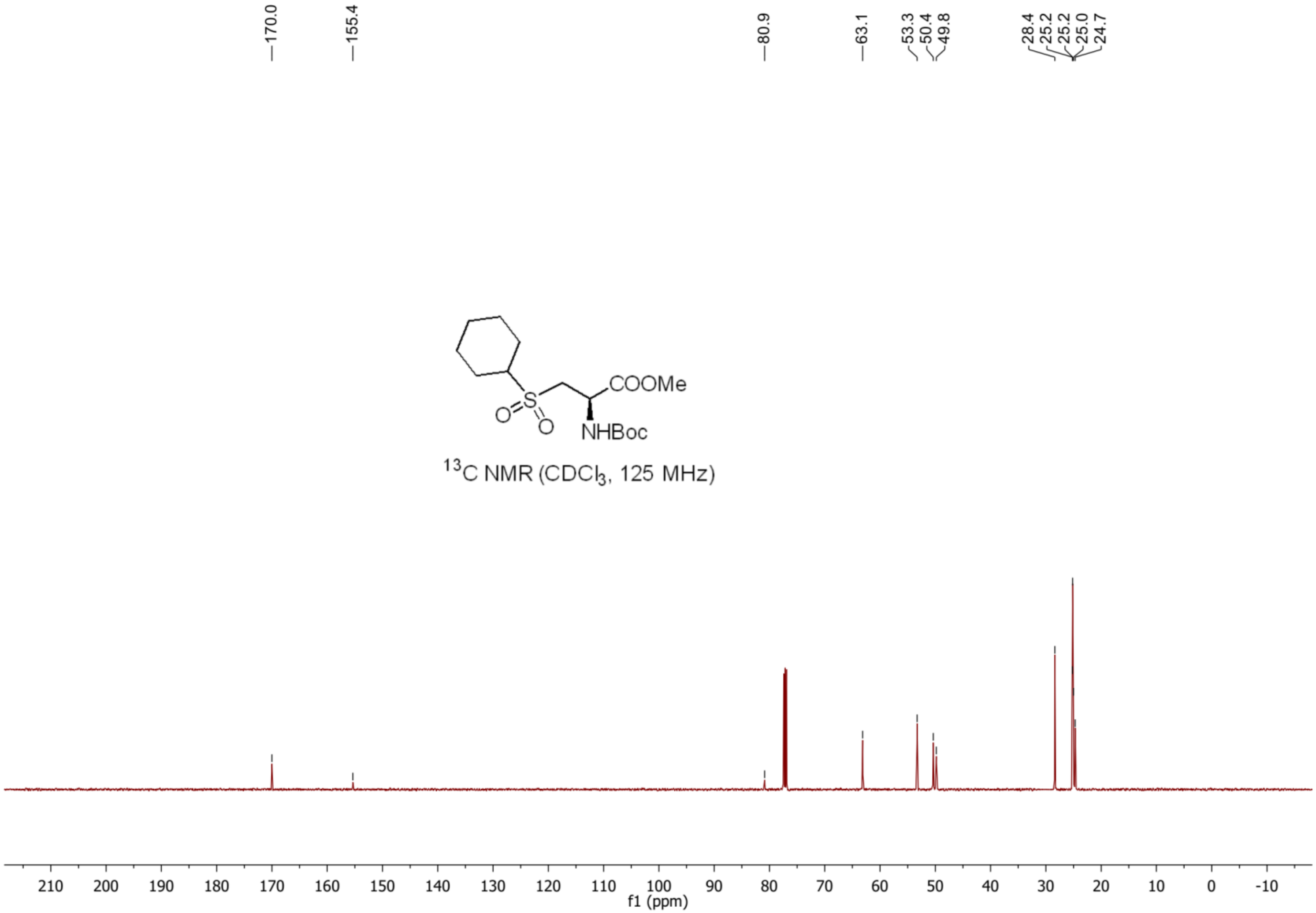

Go back to table of contents

cis-3-((Cyclohexylsulfonyl)methyl)-1,2-oxathiolane 2-oxide (2h)

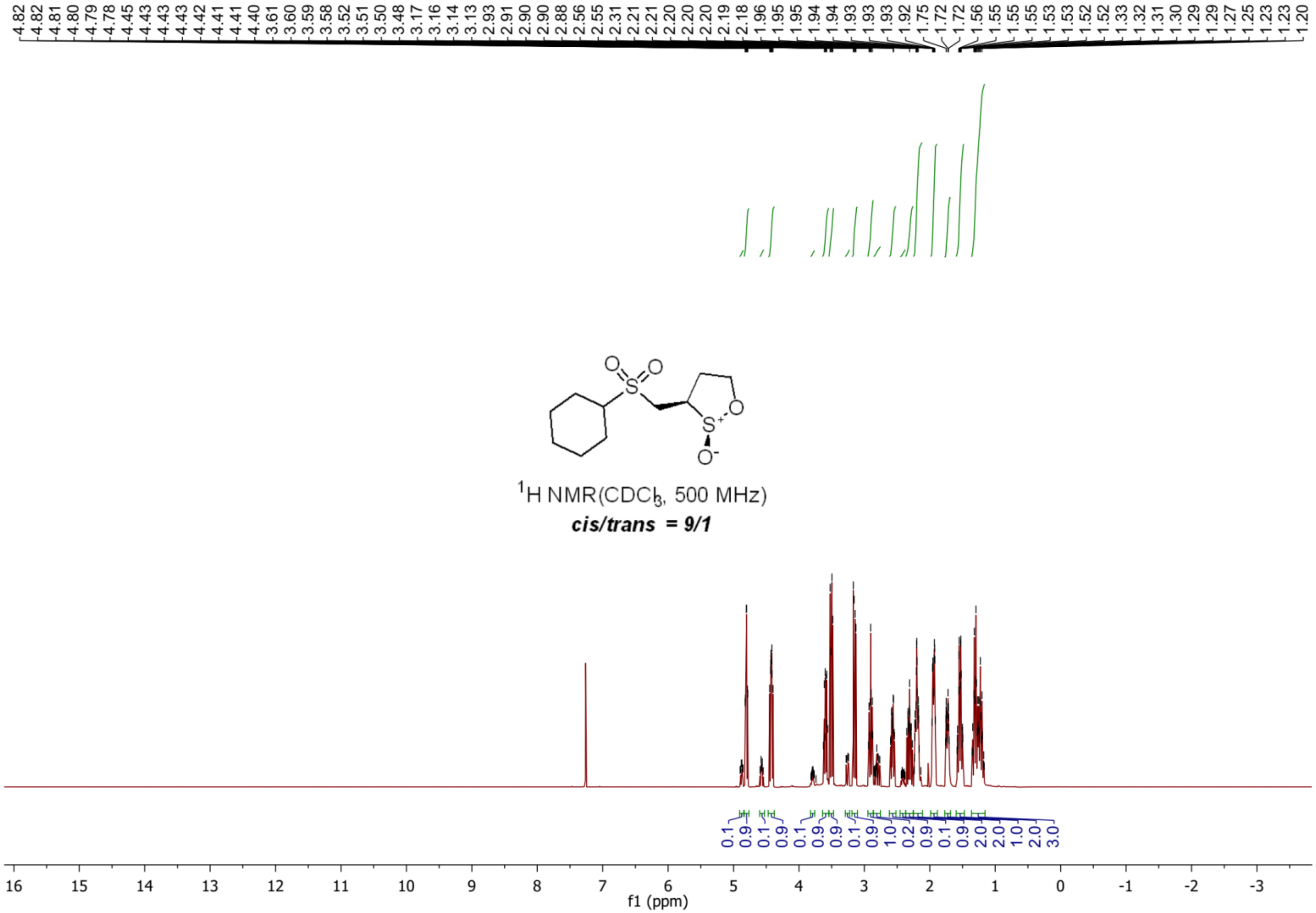

***cis*-3-((Cyclohexylsulfonyl)methyl)-1,2-oxathiolane 2-oxide (2h)**

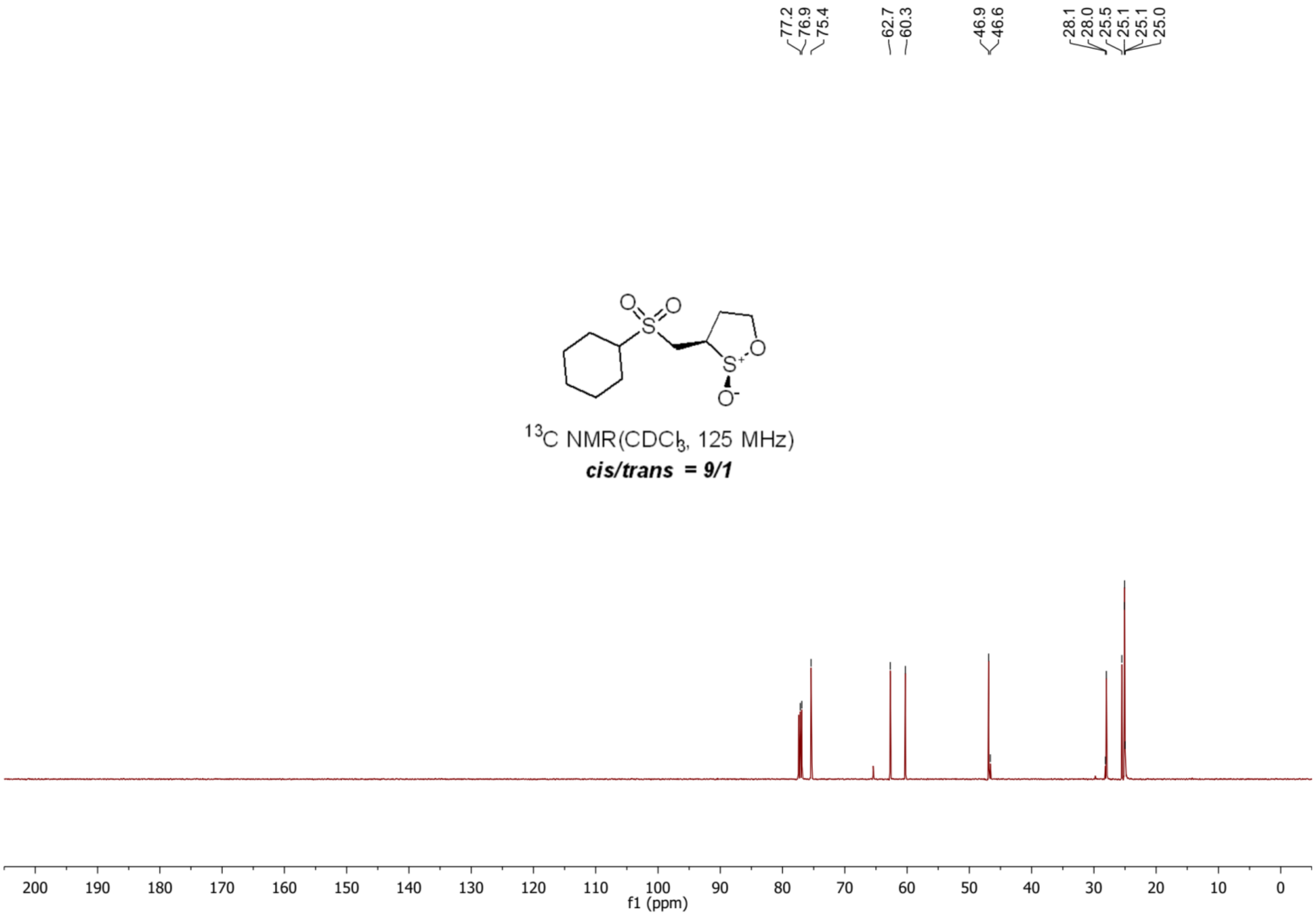

Go back to table of contents

***cis*-3-(((1-Phenylcyclopropyl)sulfonyl)methyl)-1,2-oxathiolane 2-oxide (2i)**

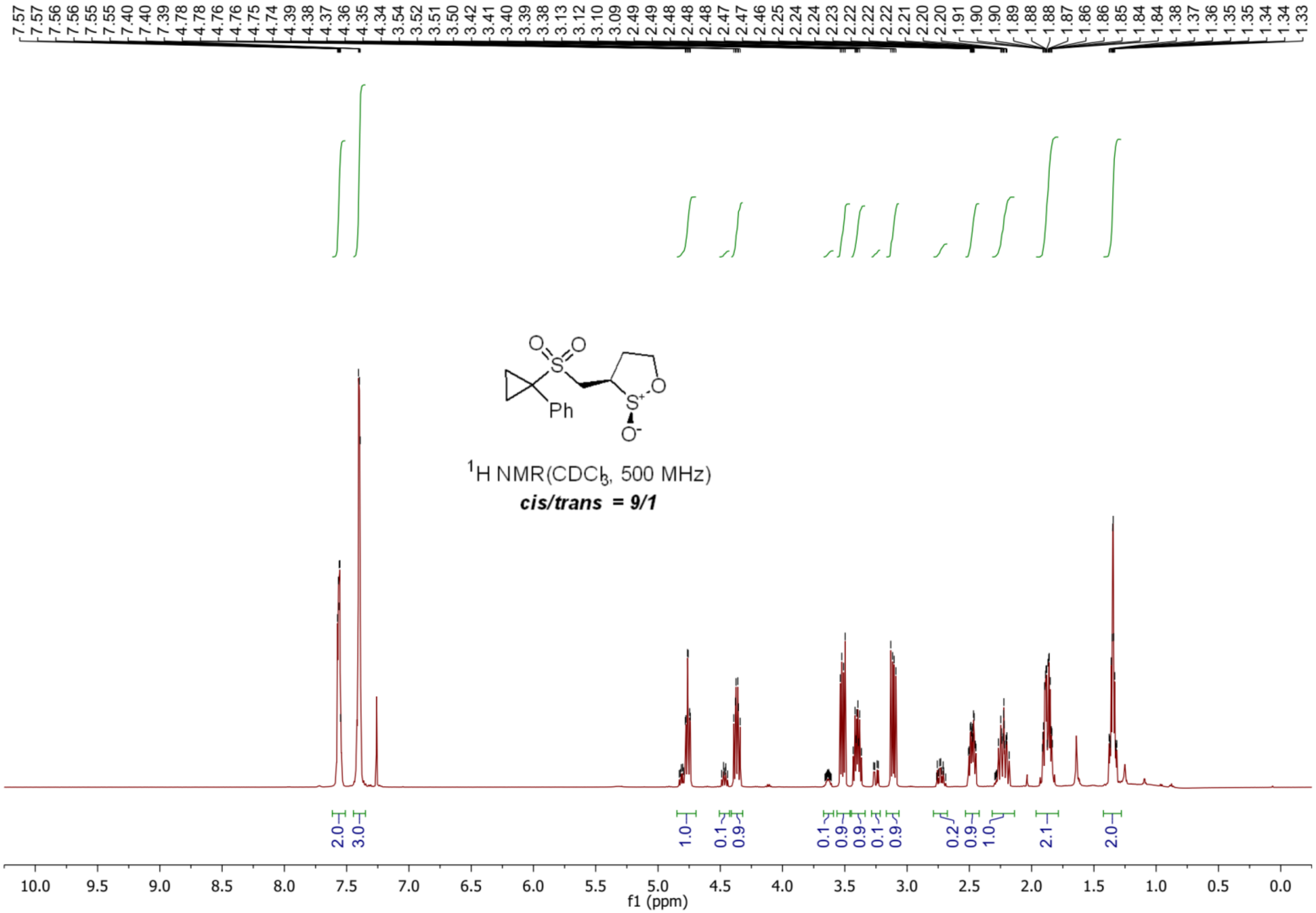

Go back to table of contents

cis-3-(((1-Phenylcyclopropyl)sulfonyl)methyl)-1,2-oxathiolane 2-oxide (2i)

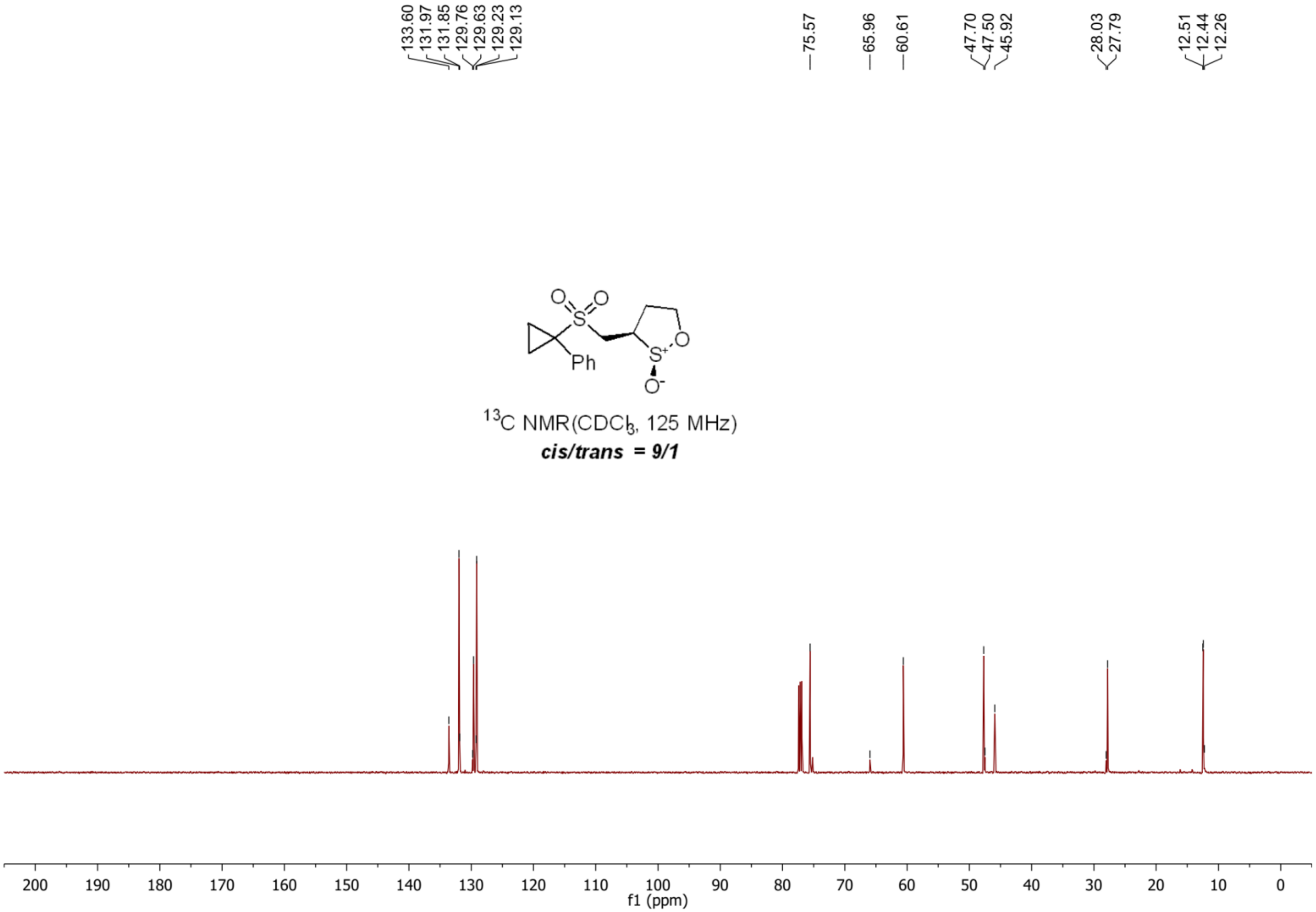

Go back to table of contents

(Ethylsulfonyl)cyclopentane (2j)

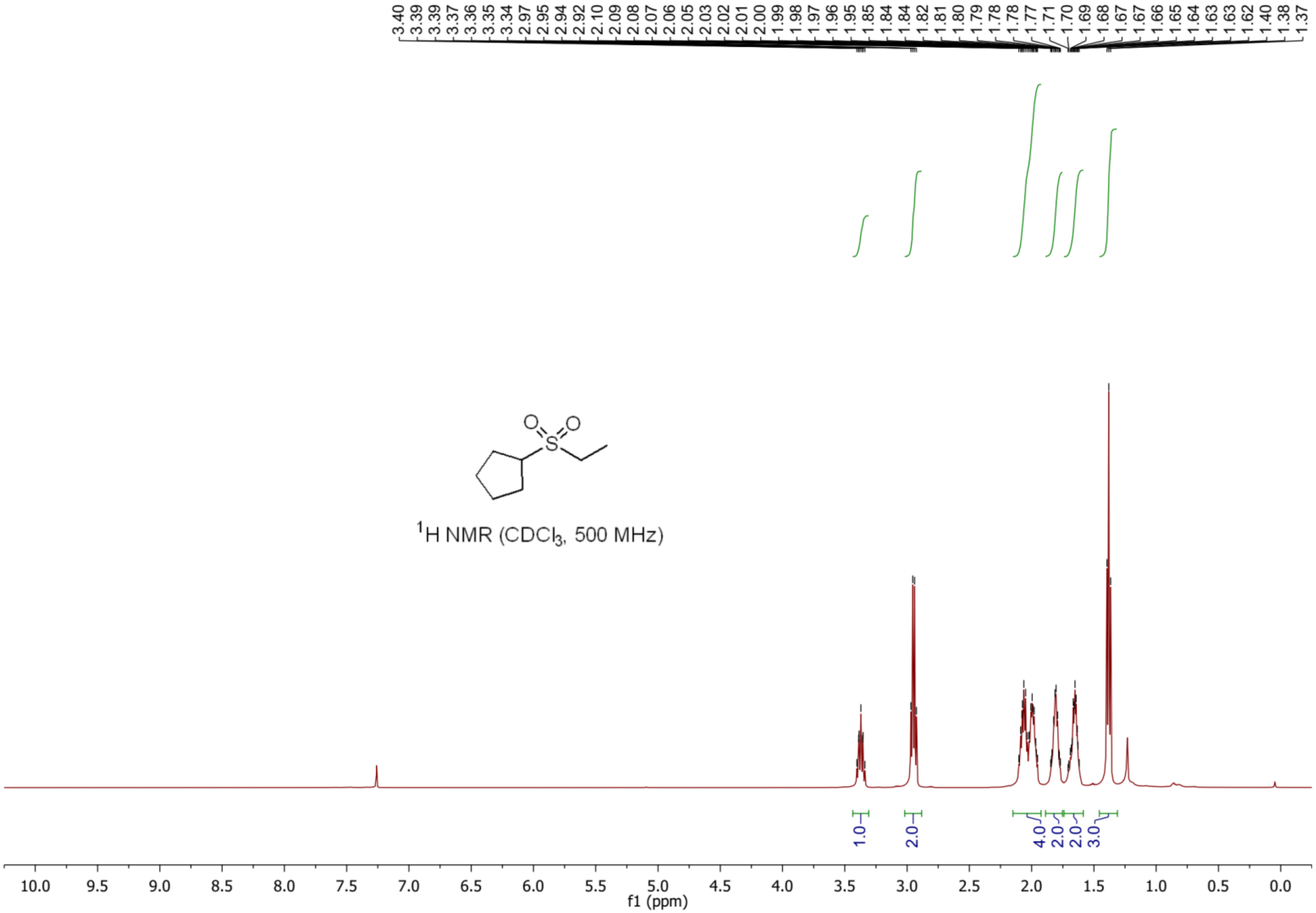

Go back to table of contents

(Ethylsulfonyl)cyclopentane (2j)

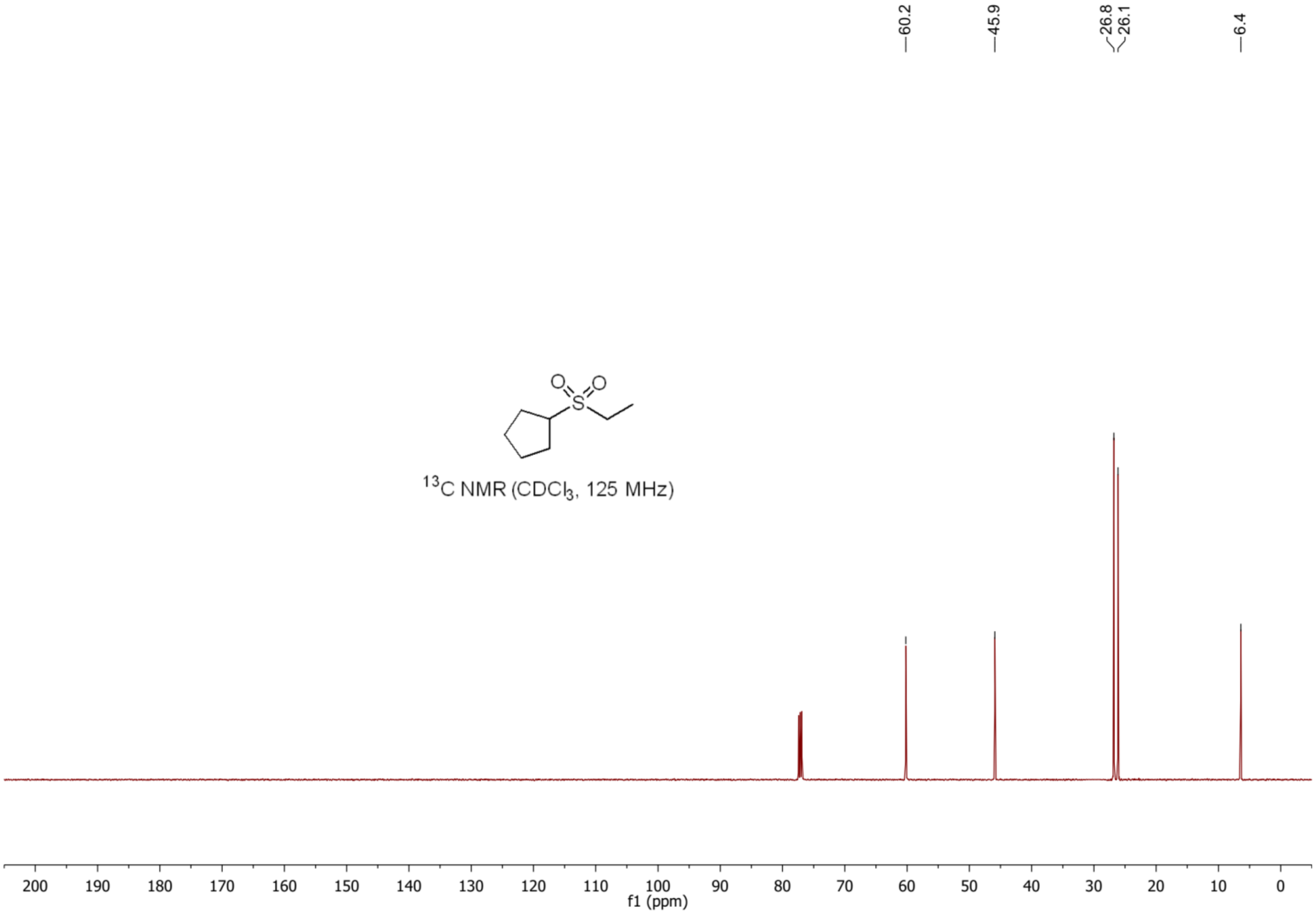

Go back to table of contents

4-((2-(4,5-diphenyloxazol-2-yl)ethyl)sulfonyl)butan-2-one (2k)

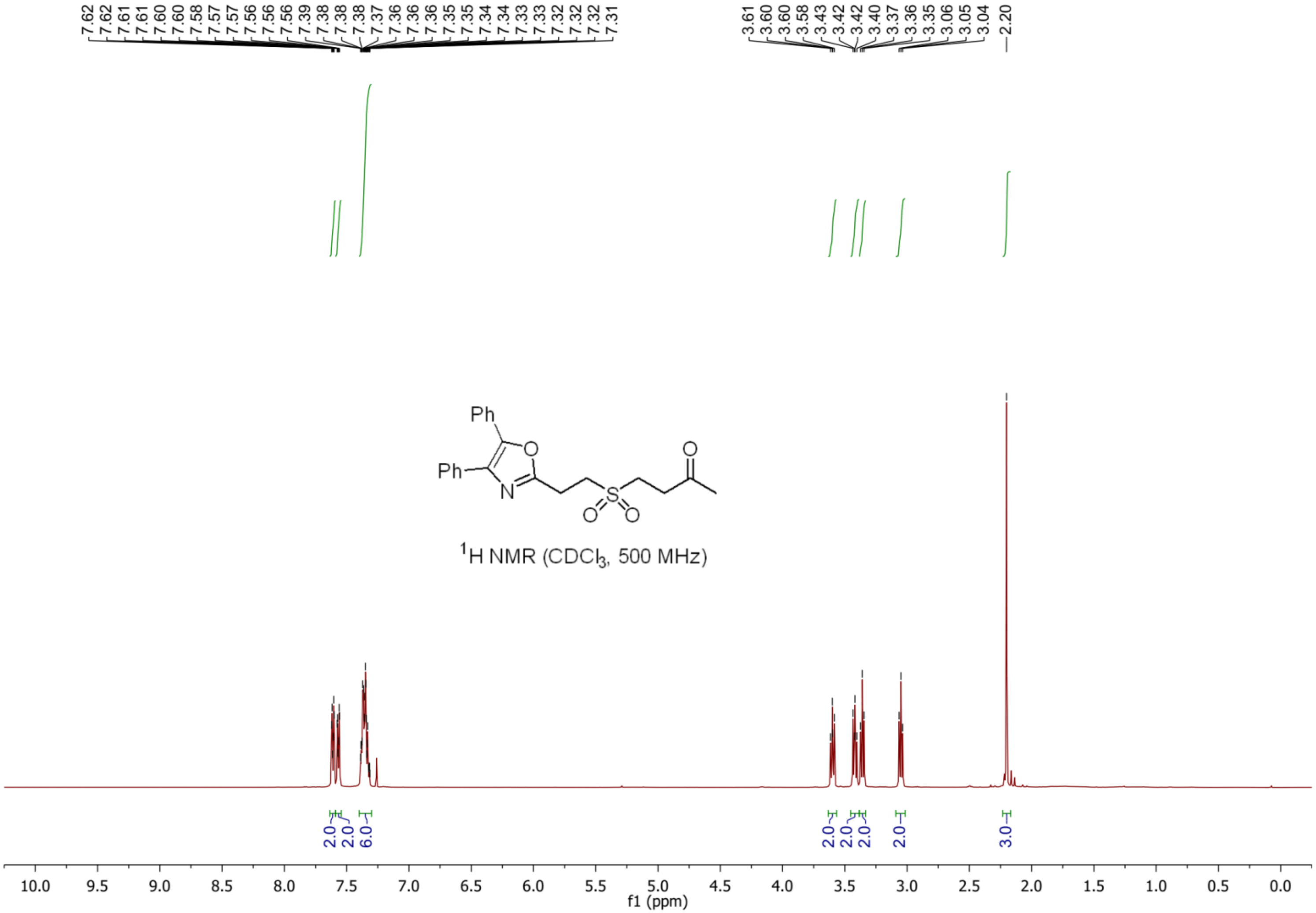

4-((2-(4,5-diphenyloxazol-2-yl)ethyl)sulfonyl)butan-2-one (2k)

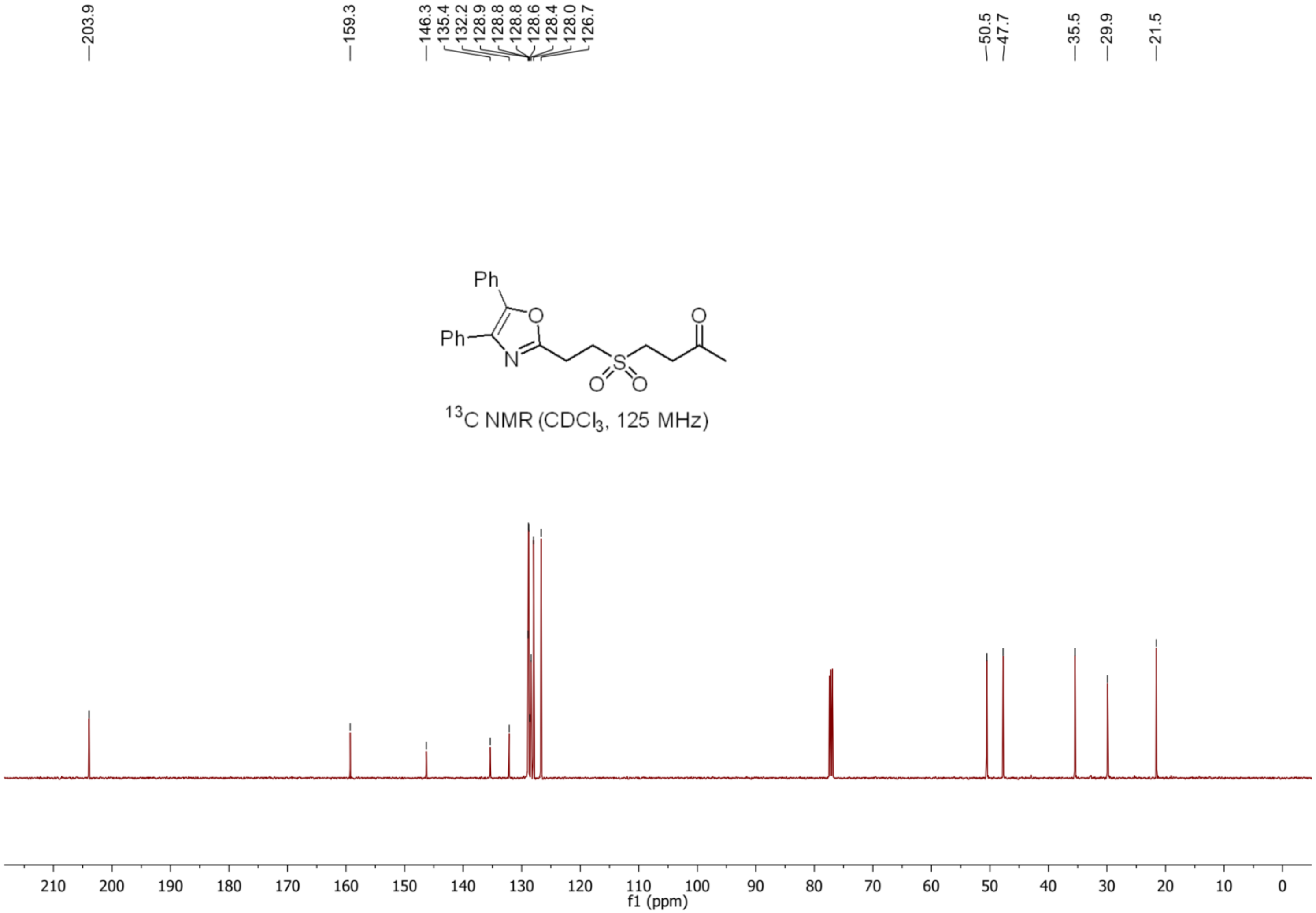

Go back to table of contents

3-((5-(5-Methylthiophen-2-yl)-5-oxopentyl)sulfonyl)cyclohexan-1-one (2l)

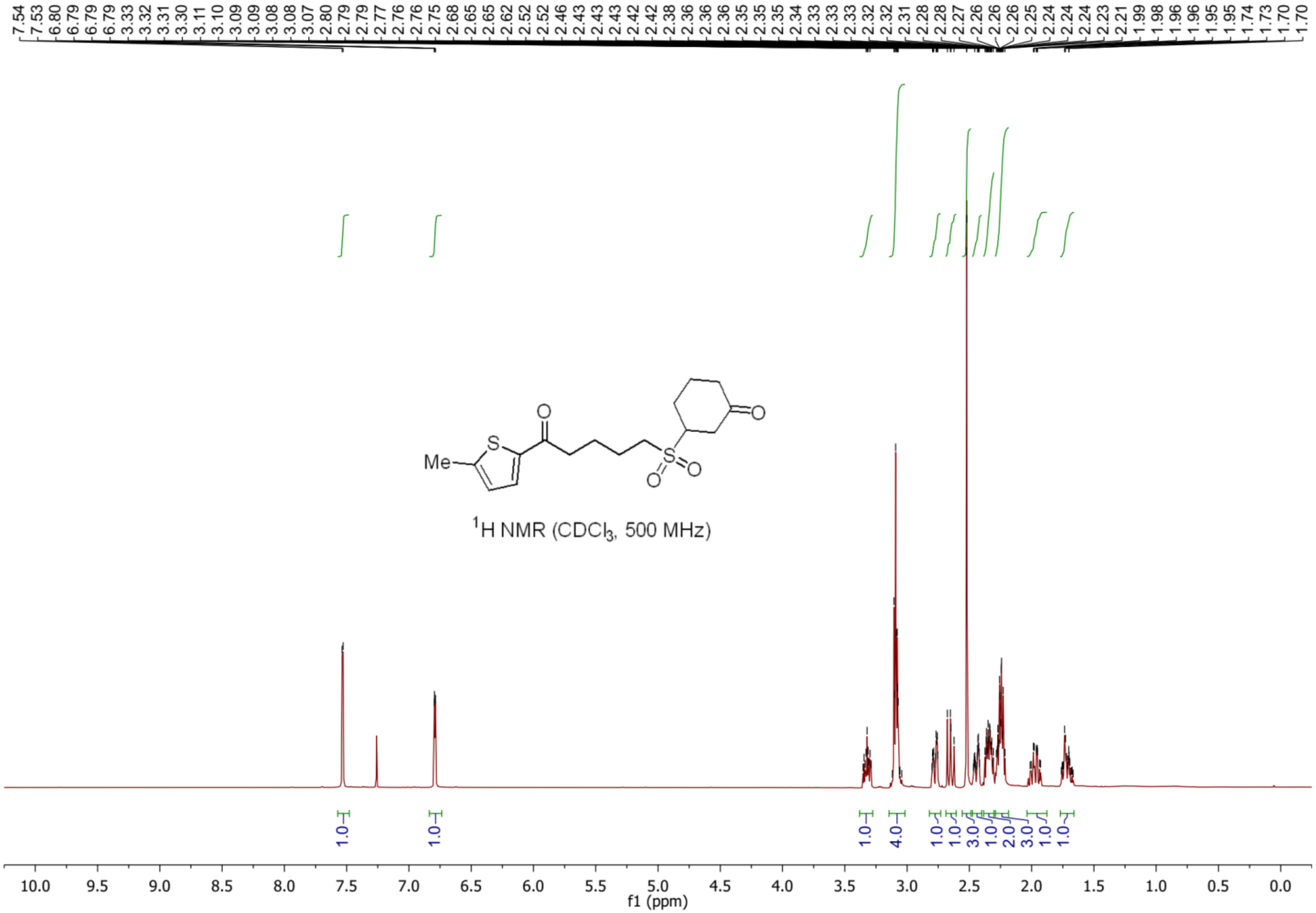

3-((5-(5-Methylthiophen-2-yl)-5-oxopentyl)sulfonyl)cyclohexan-1-one (2l)

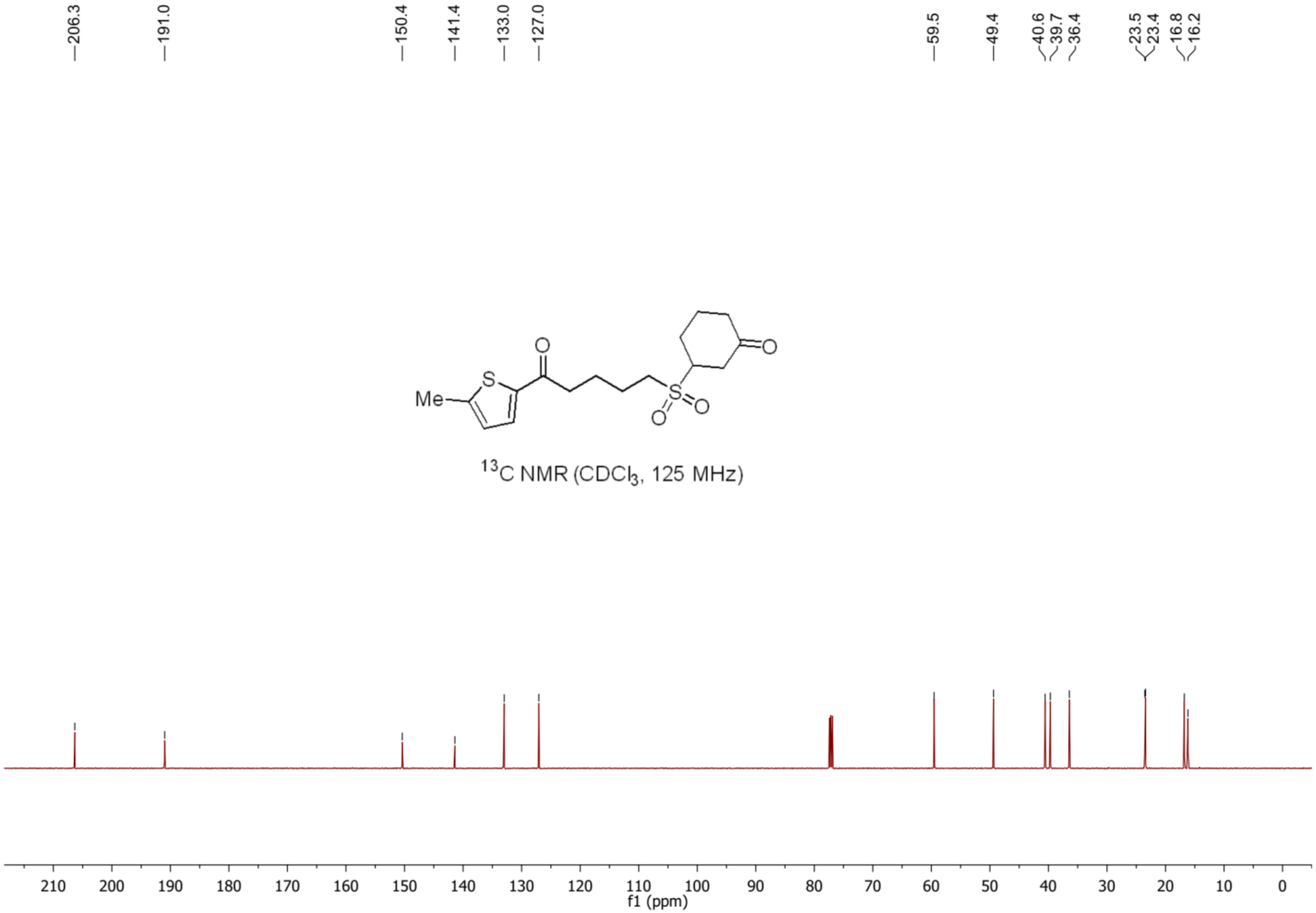

Go back to table of contents

3-((((1*S,3*R**)-3-acetyl-2,2-dimethylcyclobutyl)methyl)sulfonyl)propanal (2m)**

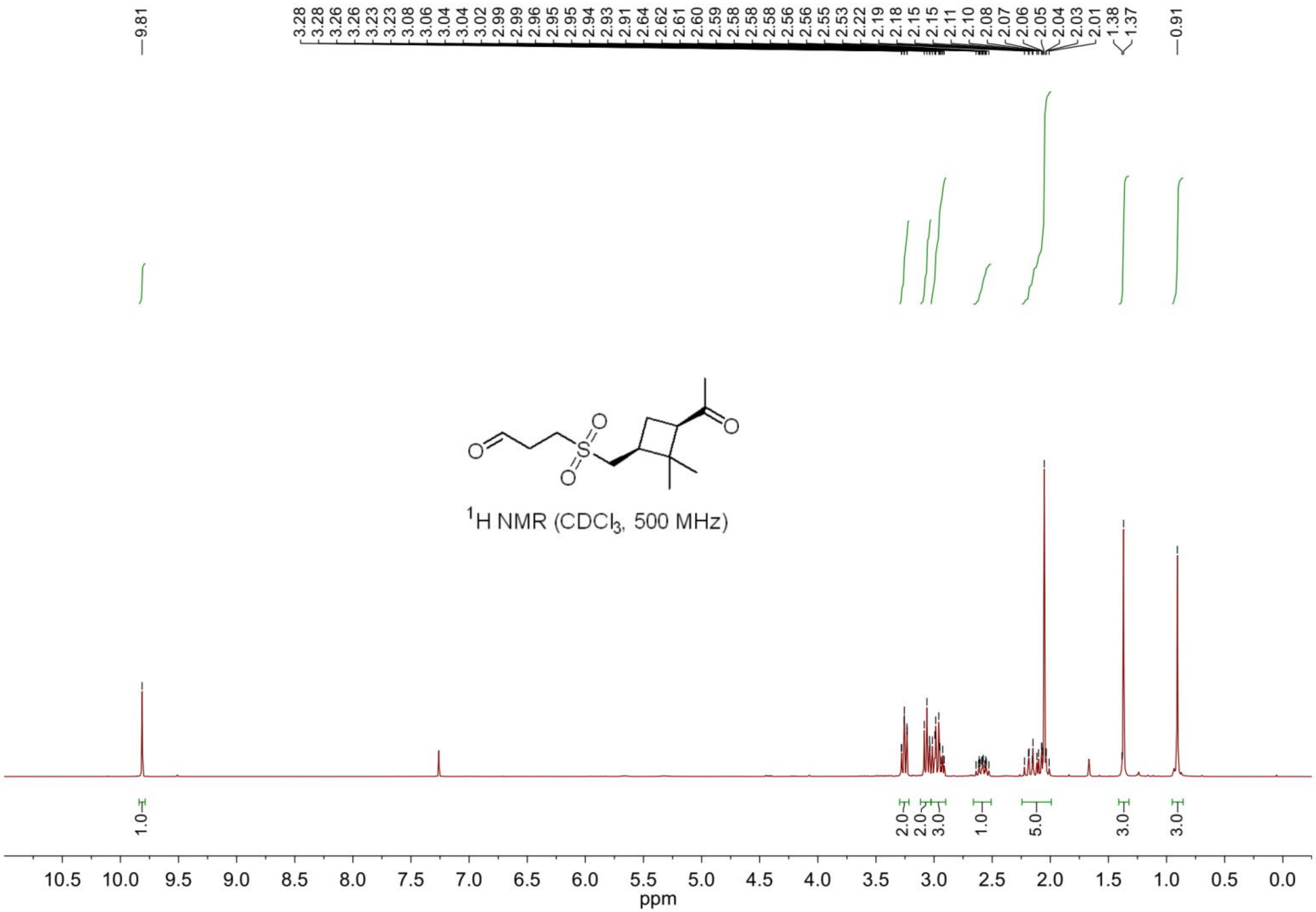

Go back to table of contents

3-((((1*S,3*R**)-3-acetyl-2,2-dimethylcyclobutyl)methyl)sulfonyl)propanal (2m)**

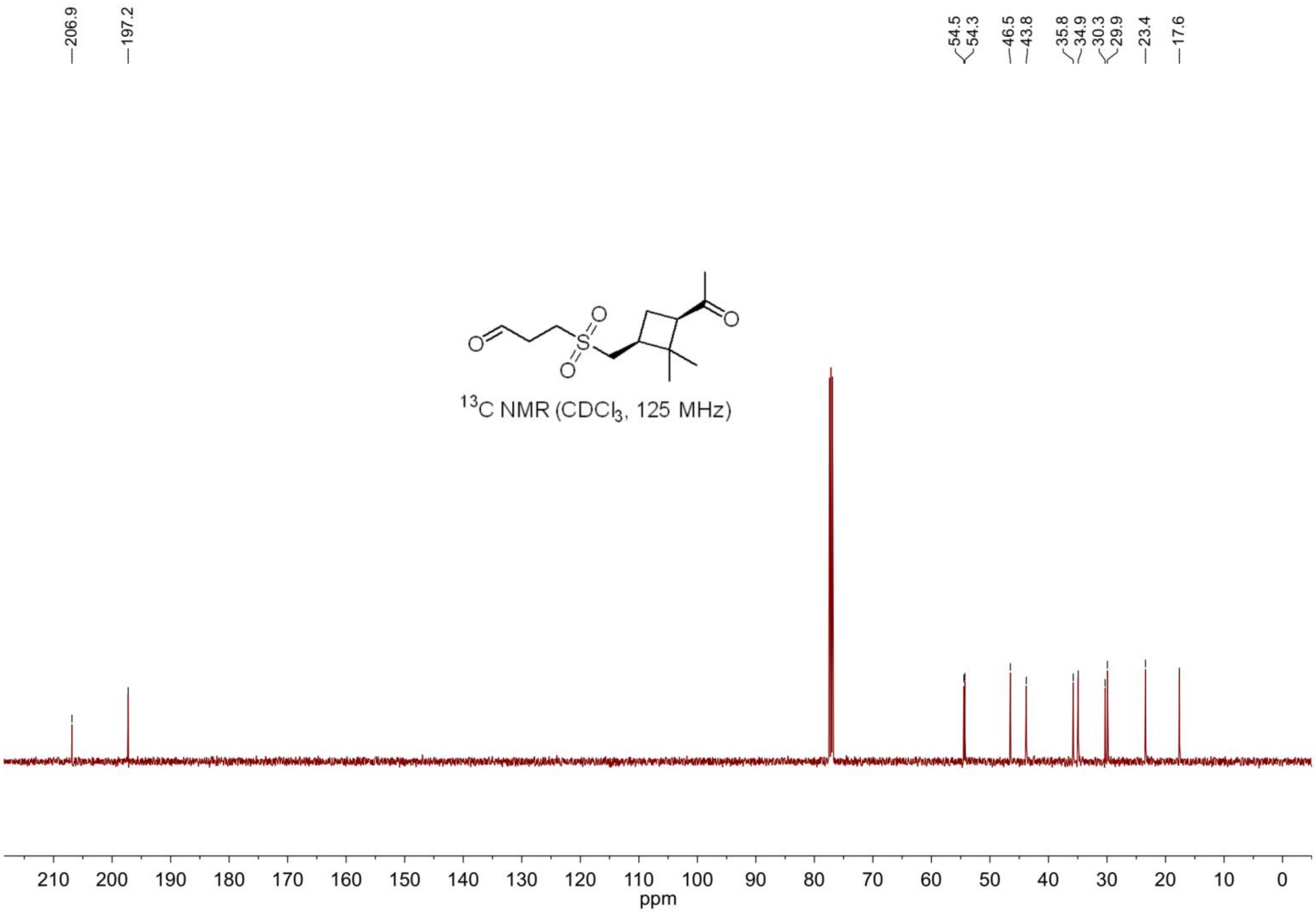

Go back to table of contents

3-((Cyclopentylmethyl)sulfonyl)pentanal (2n)

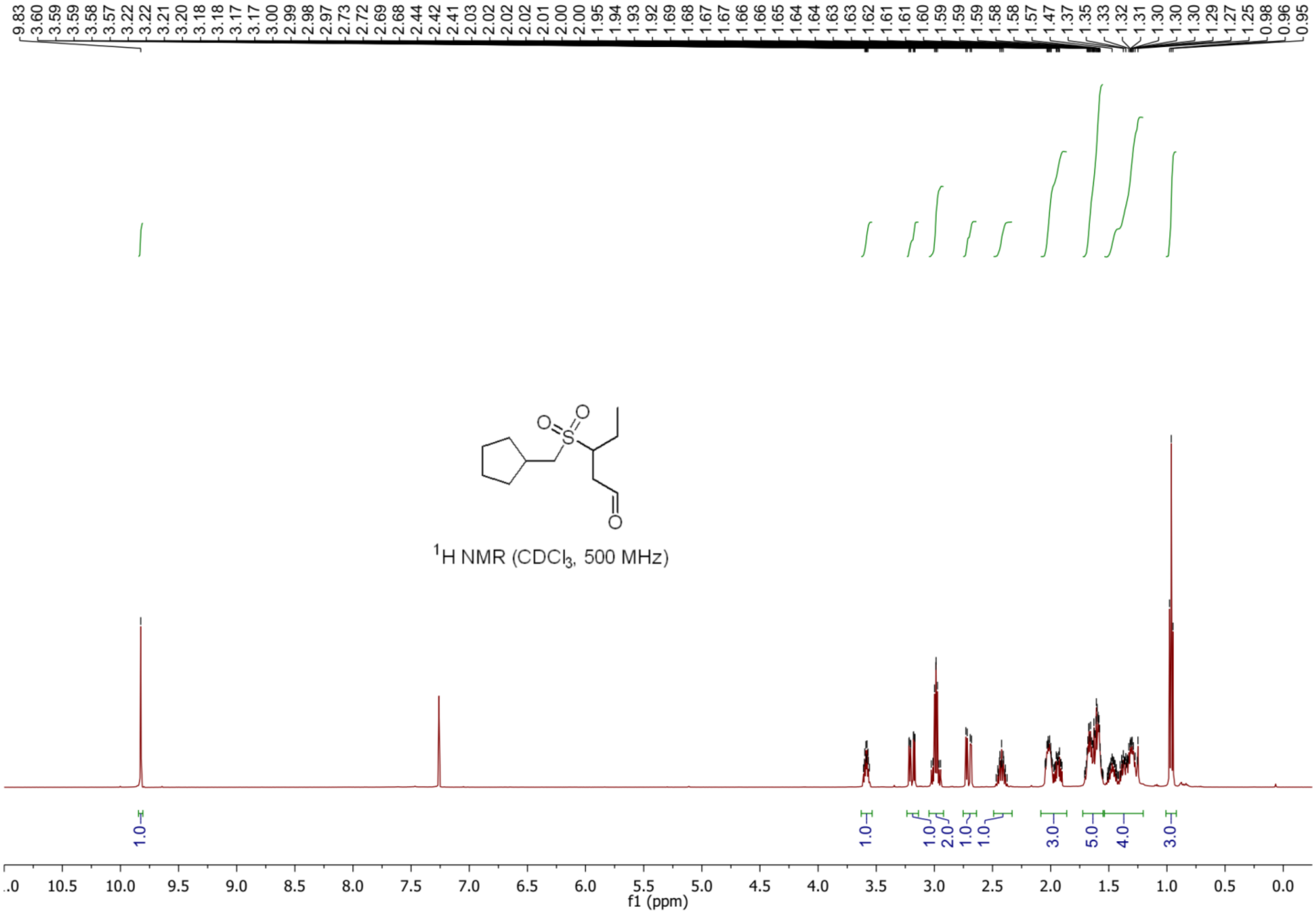

3-((Cyclopentylmethyl)sulfonyl)pentanal (2n)

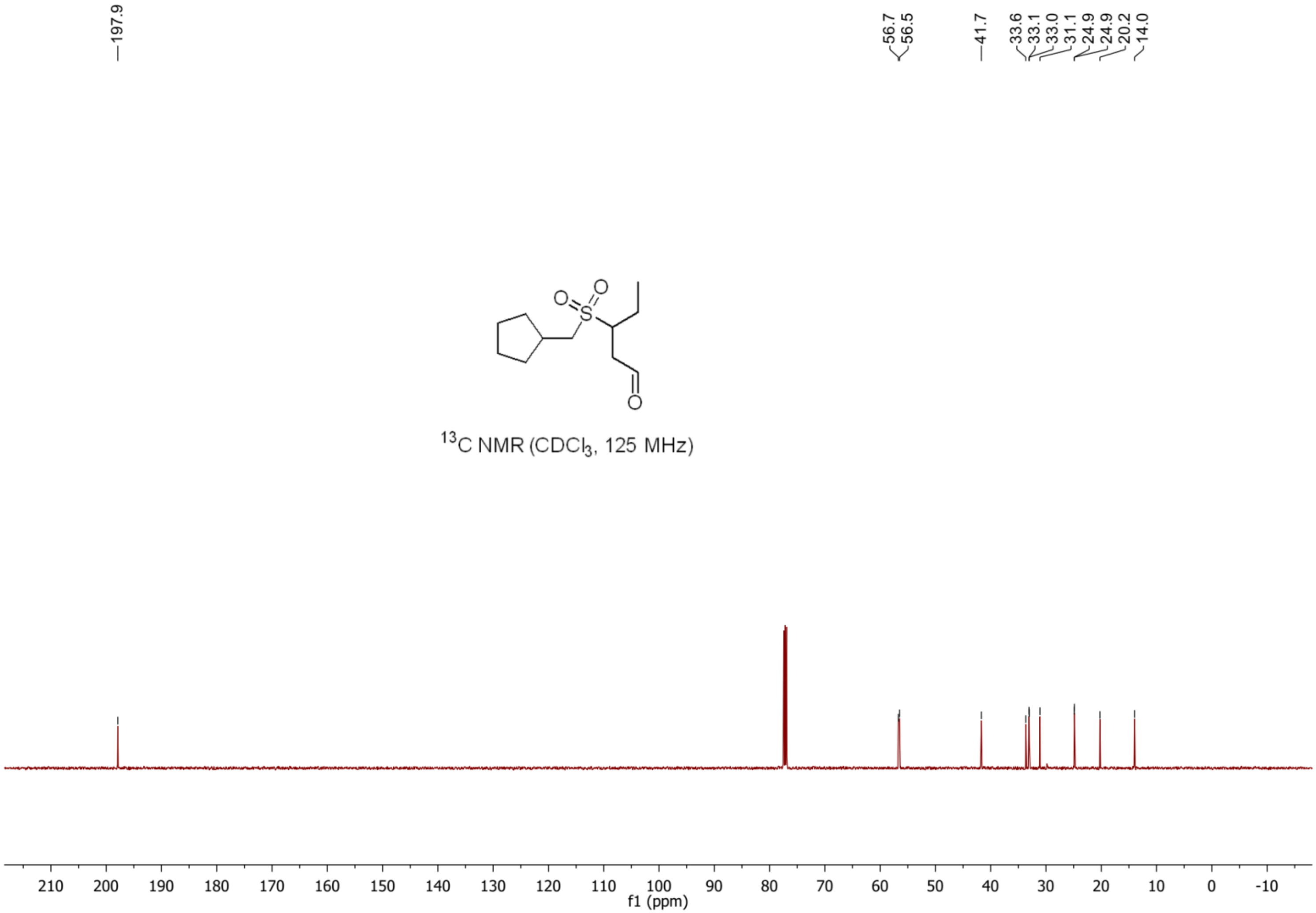

Go back to table of contents

3-(((3*R*)-3-((3*R*,7*S*,8*R*,9*S*,10*S*,13*R*,14*S*,17*R*)-3,7-dihydroxy-10,13-dimethylhexadecahydro-1*H*-cyclopenta[a]phenanthren-17-yl)butyl)sulfonyl)propanenitrile (2o)

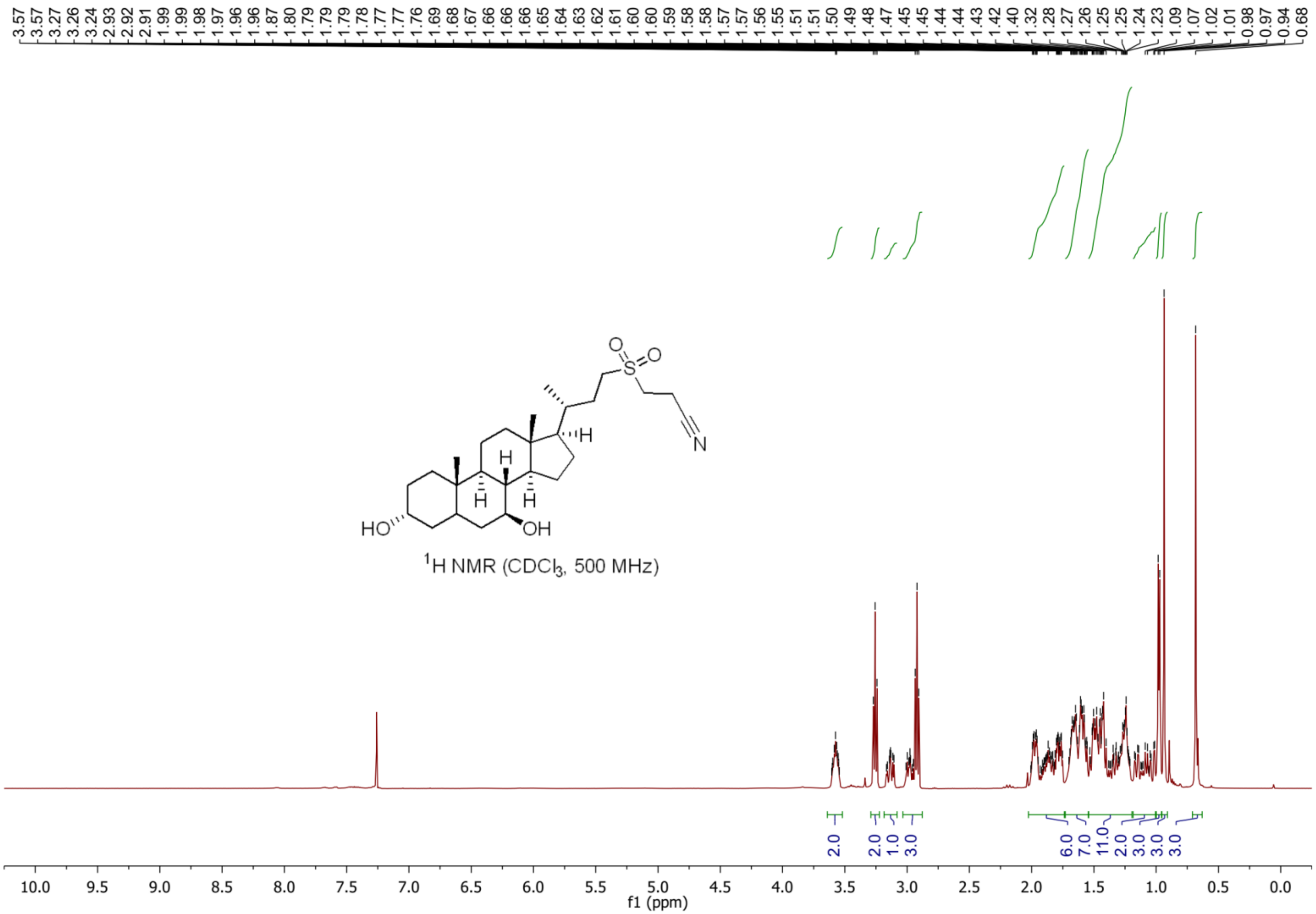

3-(((3*R*)-3-((3*R*,7*S*,8*R*,9*S*,10*S*,13*R*,14*S*,17*R*)-3,7-dihydroxy-10,13-dimethylhexadecahydro-1*H*-cyclopenta[a]phenanthren-17-yl)butyl)sulfonyl)propanenitrile (2o)

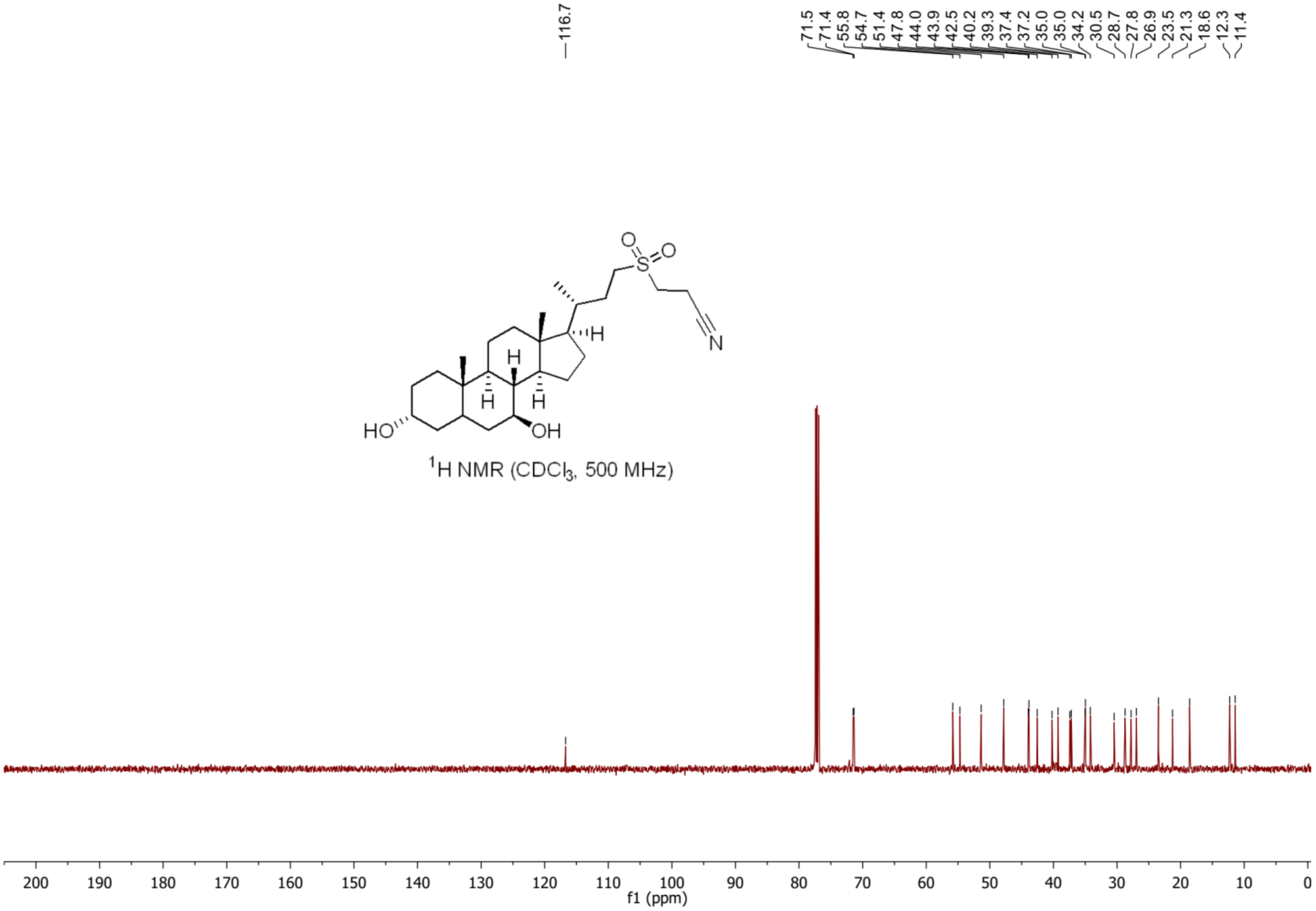

Go back to table of contents

(2*R*,4a*S*,6a*S*,6b*R*,10*S*,12a*S*,14b*R*)-10-hydroxy-2,4a,6a,6b,9,9,12a-heptamethyl-2-((2-(phenylsulfonyl)ethyl)sulfonyl)-1,3,4,4a,5,6,6a,6b,7,8,8a,9,10,11,12,12a,12b,14b-octadecahydropicen-13(2*H*)-one (2p)

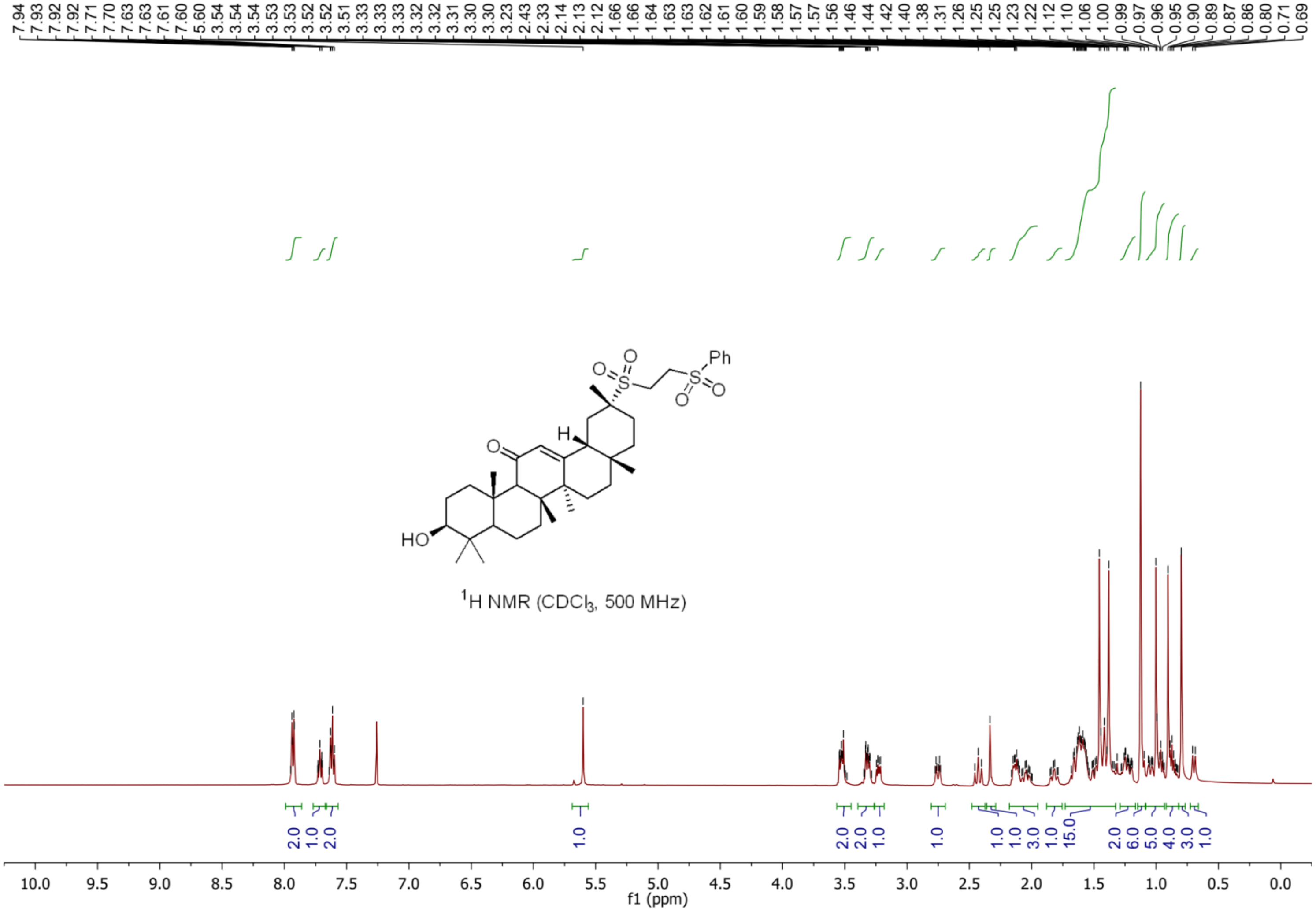

Go back to table of contents

(2*R*,4a*S*,6a*S*,6b*R*,10*S*,12a*S*,14b*R*)-10-hydroxy-2,4a,6a,6b,9,9,12a-heptamethyl-2-((2-(phenylsulfonyl)ethyl)sulfonyl)-1,3,4,4a,5,6,6a,6b,7,8,8a,9,10,11,12,12a,12b,14b-octadecahydropicen-13(2*H*)-one (2p)

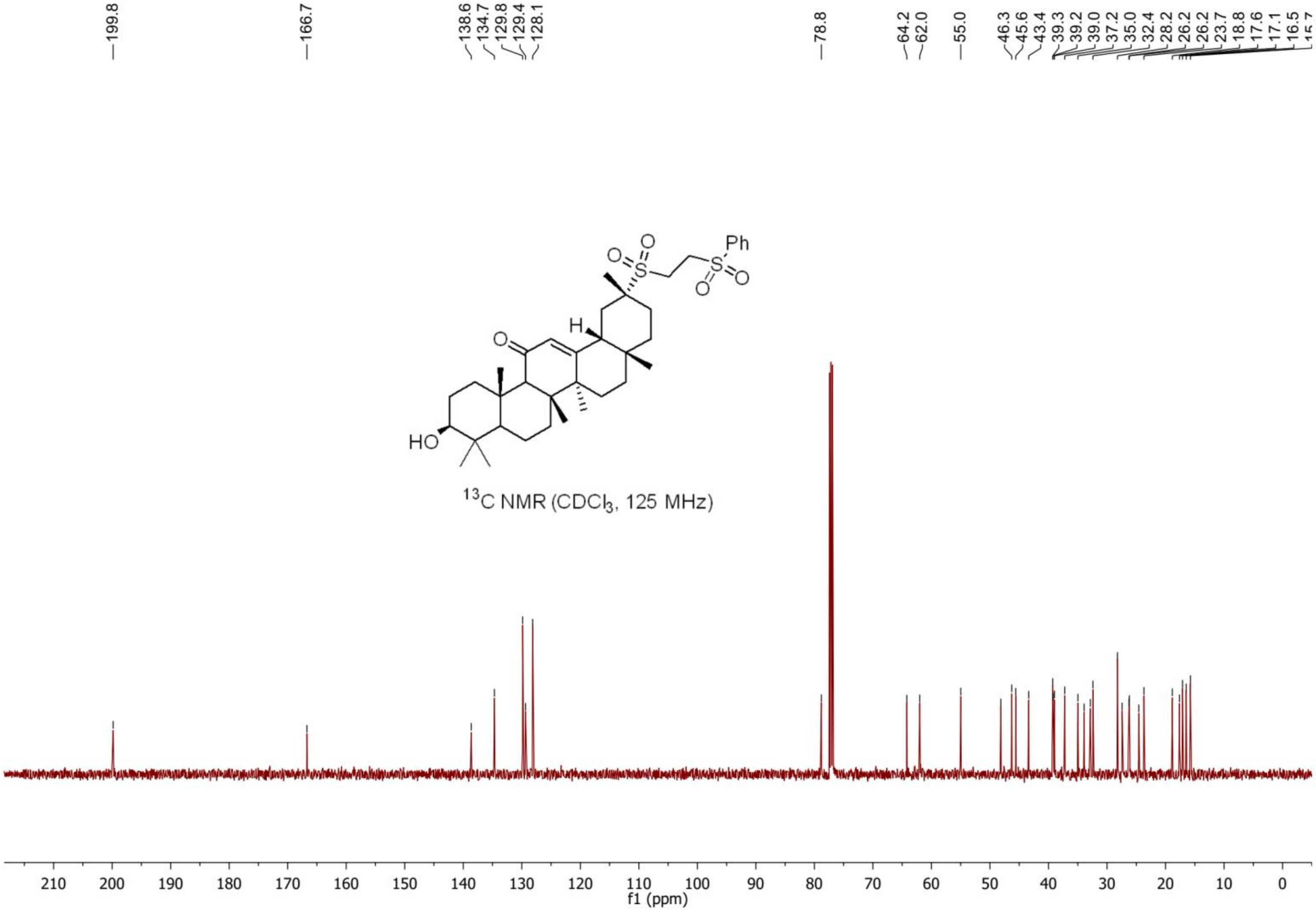

Go back to table of contents

1-(Cyclohexylsulfonyl)isoquinoline (2q)

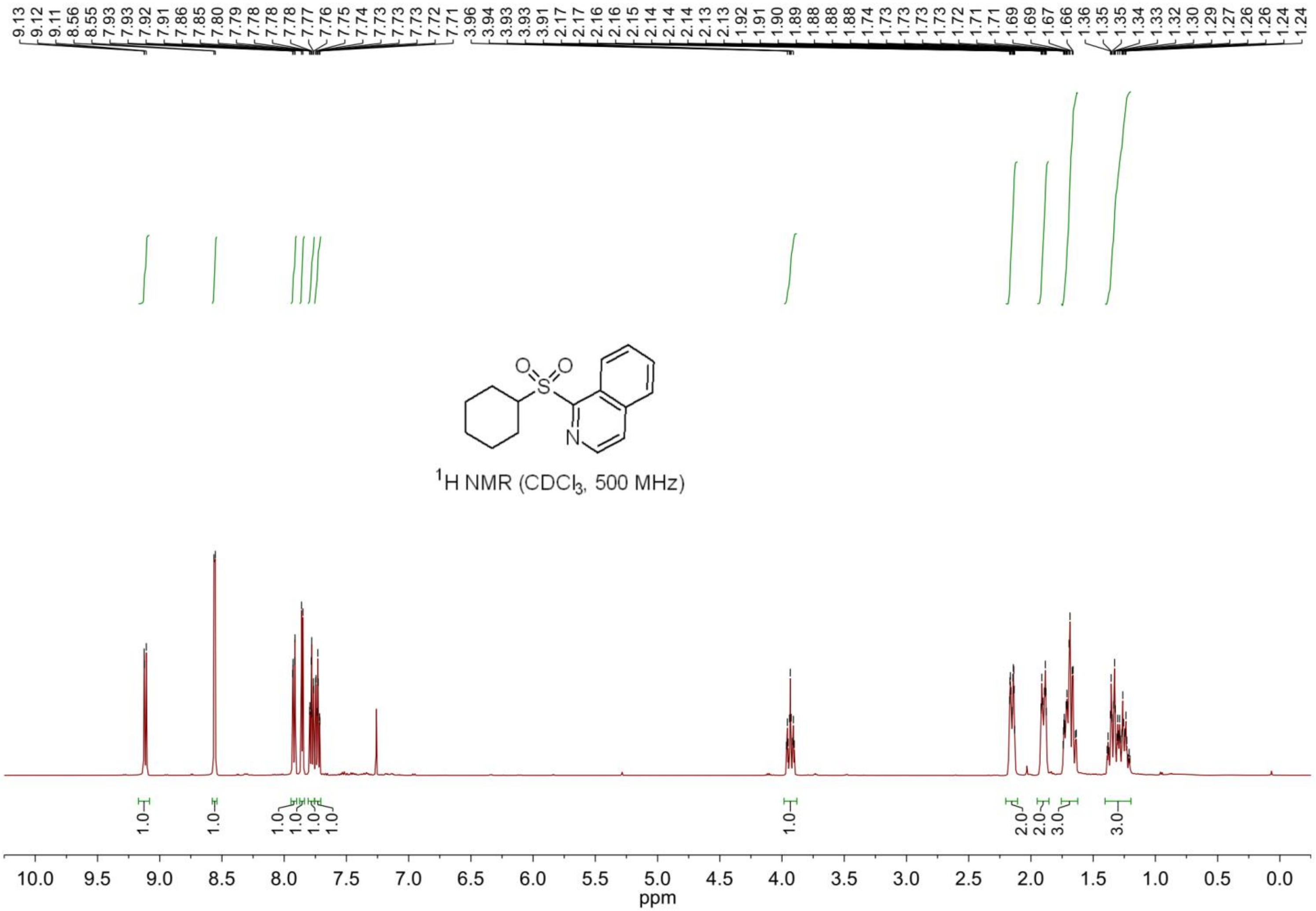

Go back to table of contents

1-(Cyclohexylsulfonyl)isoquinoline (2q)

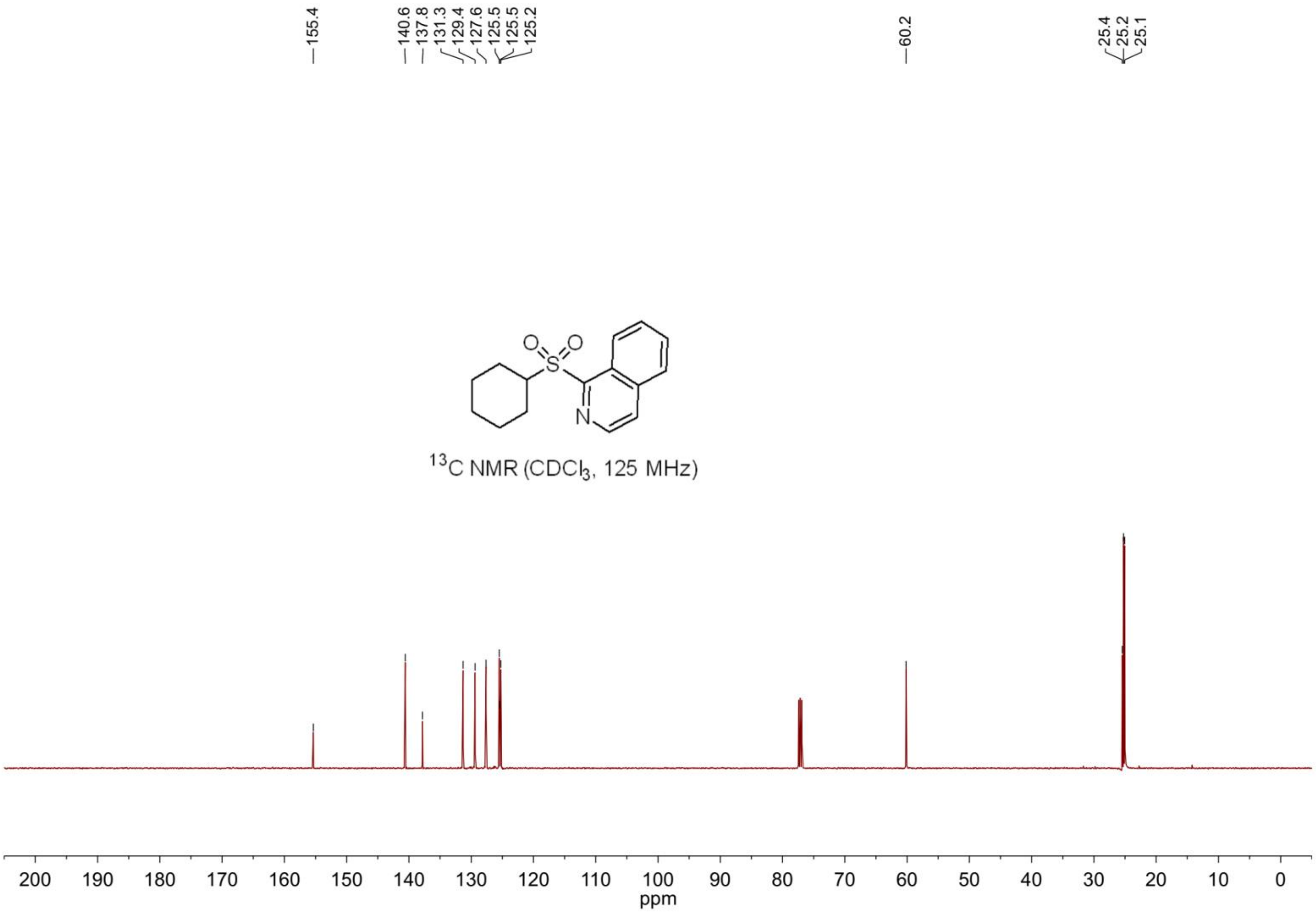

Go back to table of contents

4-(Heptan-4-ylsulfonyl)quinoline (2r)

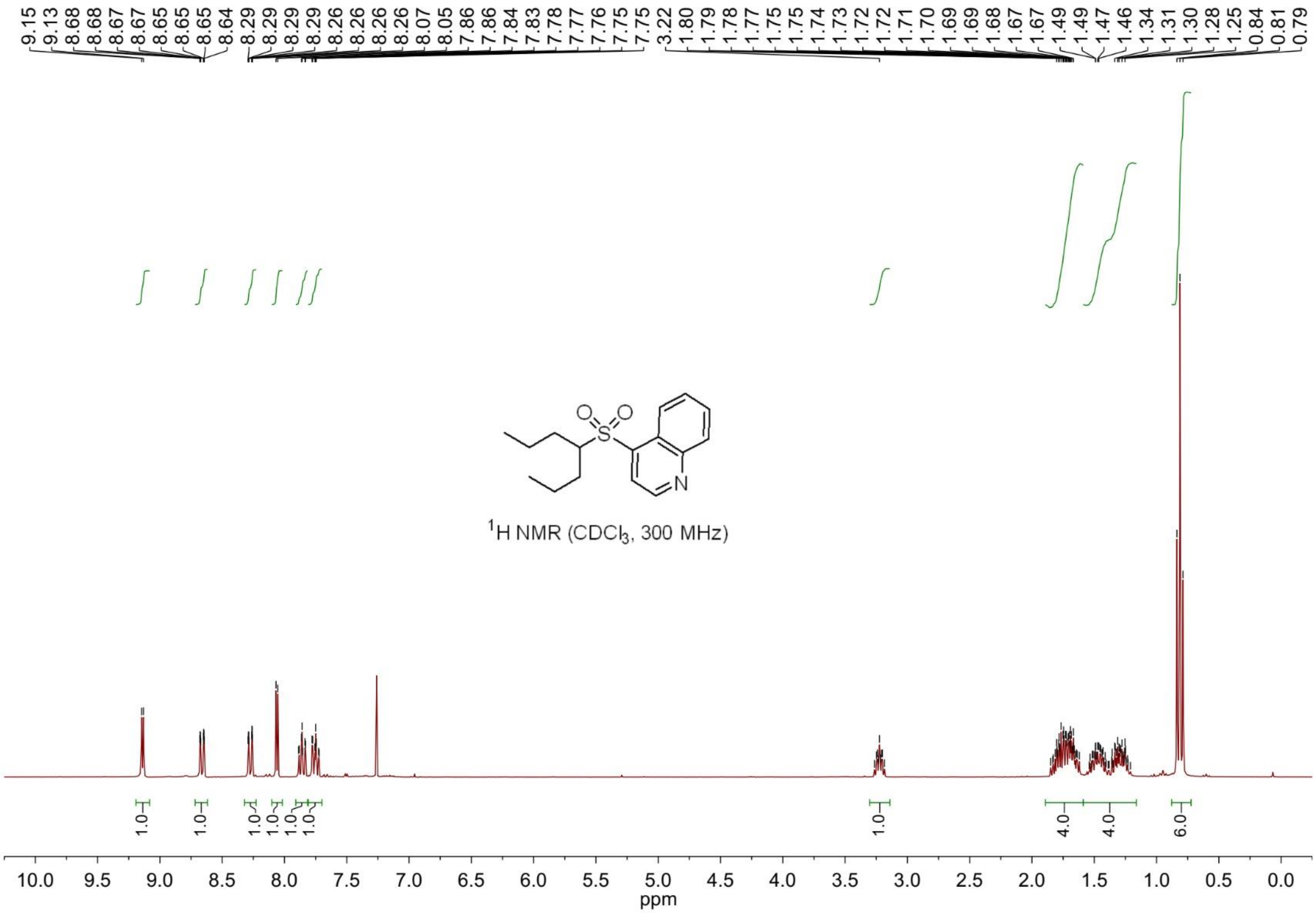

Go back to table of contents

4-(Heptan-4-ylsulfonyl)quinoline (2r)

149.6
149.5
142.4
130.9
130.5
129.1
124.2
123.1
123.1
64.1
29.6
19.9
13.9

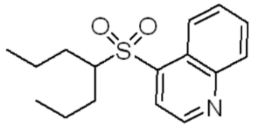

^{13}C NMR ($CDCl_3$, 125 MHz)

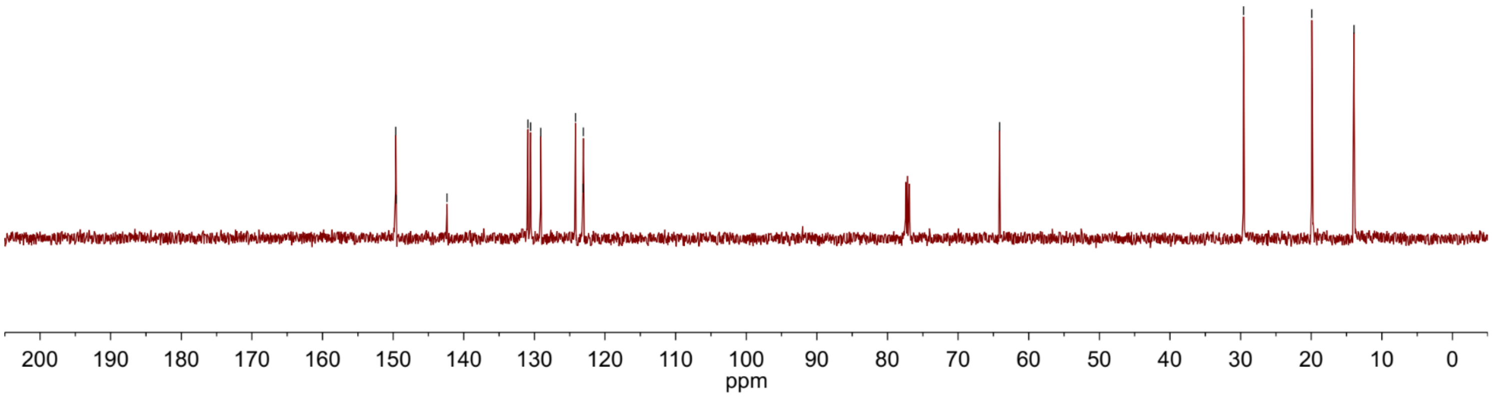

Go back to table of contents

7-Chloro-4-((4-chlorobutyl)sulfonyl)quinoline (2s)

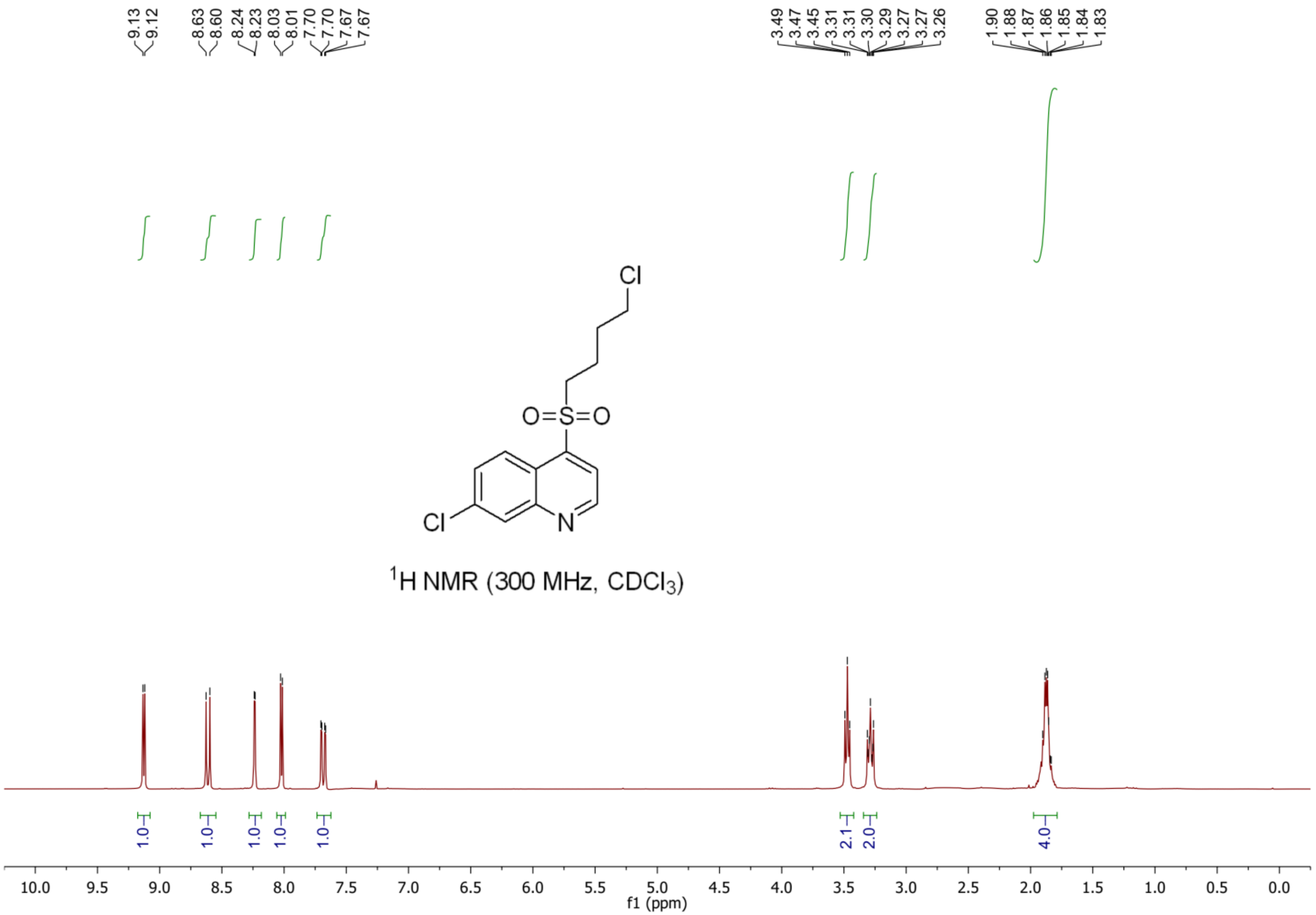

Go back to table of contents

7-Chloro-4-((4-chlorobutyl)sulfonyl)quinoline (2s)

151.0 149.9 142.8 136.9 130.3 129.9 125.3 122.5 121.1 55.4 43.8 30.7 20.1

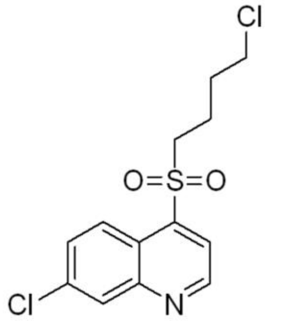

^{13}C NMR (75 MHz, $CDCl_3$)

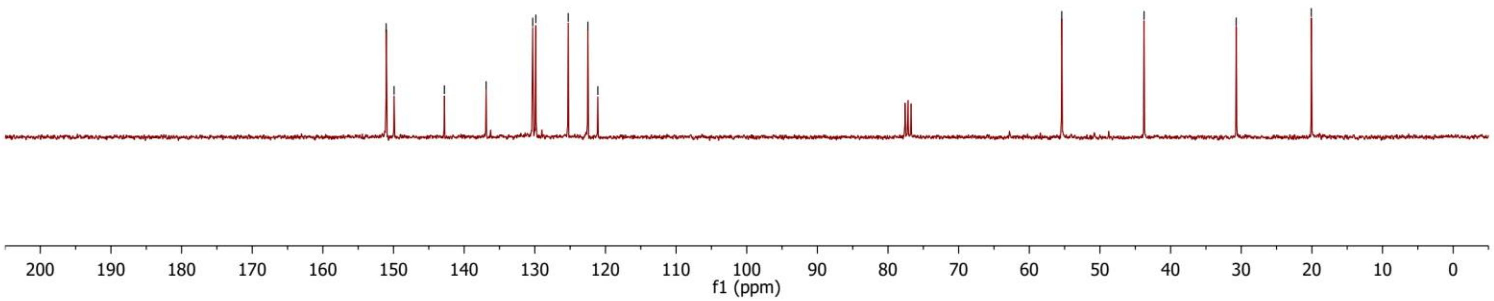

Go back to table of contents

7-Chloro-4-((1-methylcyclohexyl)sulfonyl)quinoline (2t)

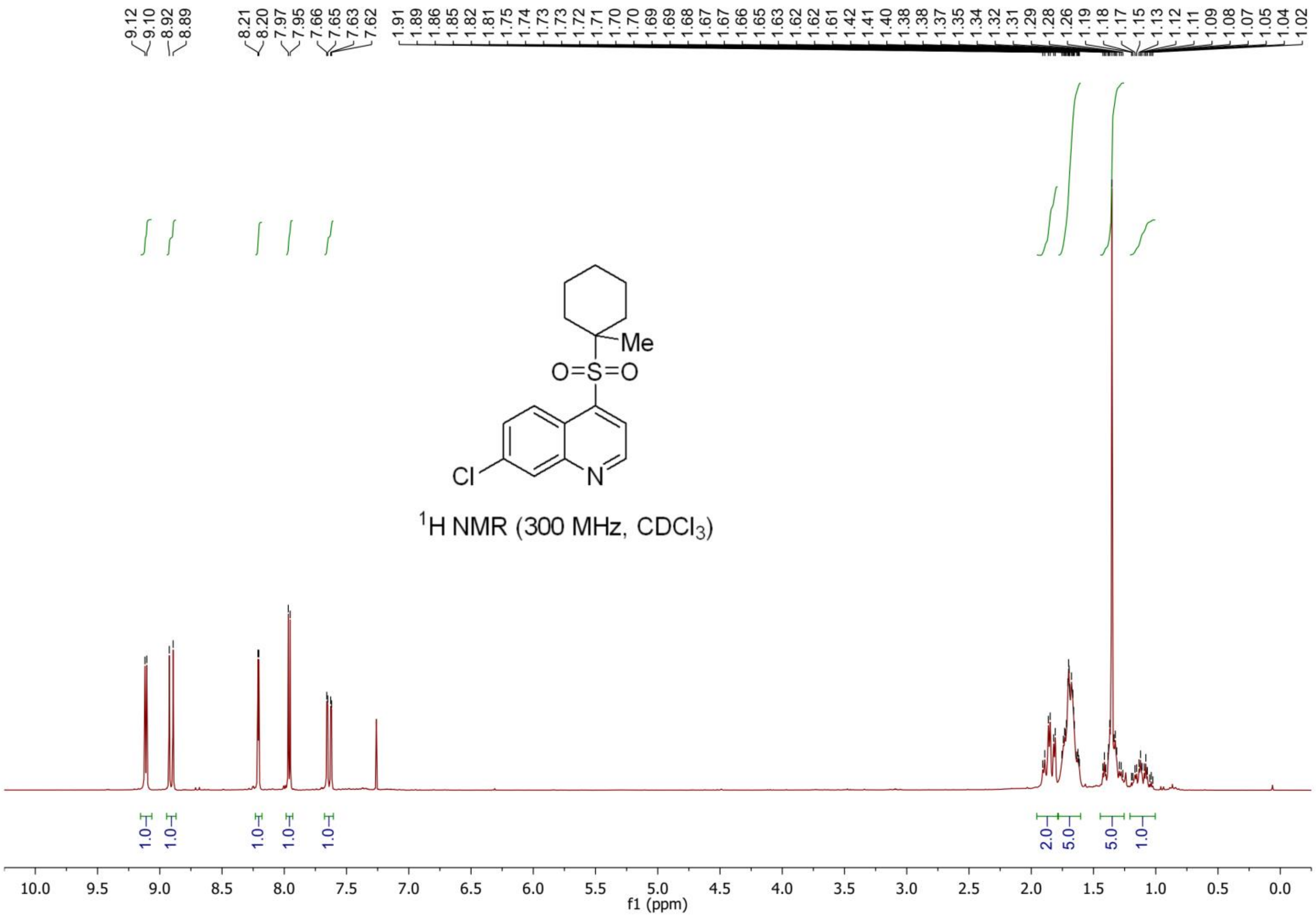

Go back to table of contents

7-Chloro-4-((1-methylcyclohexyl)sulfonyl)quinoline (2t)

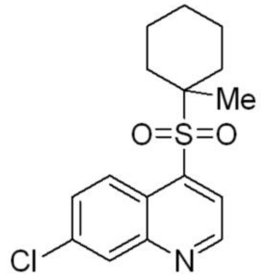

^{13}C NMR (75 MHz, $CDCl_3$)

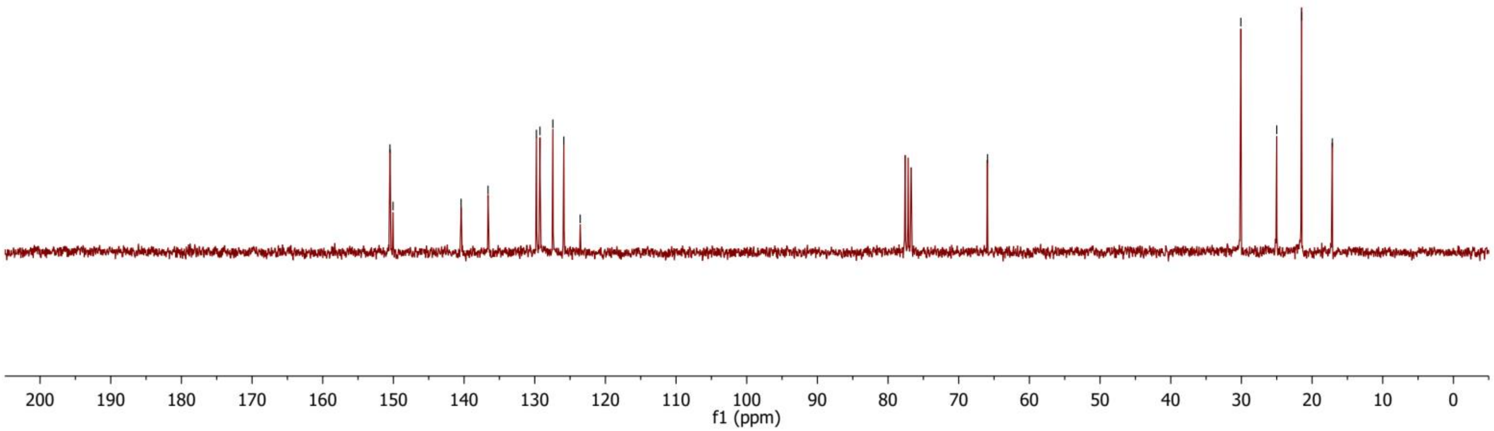

Go back to table of contents

7-Chloro-4-((3,4-dimethoxyphenethyl)sulfonyl)quinoline (2u)

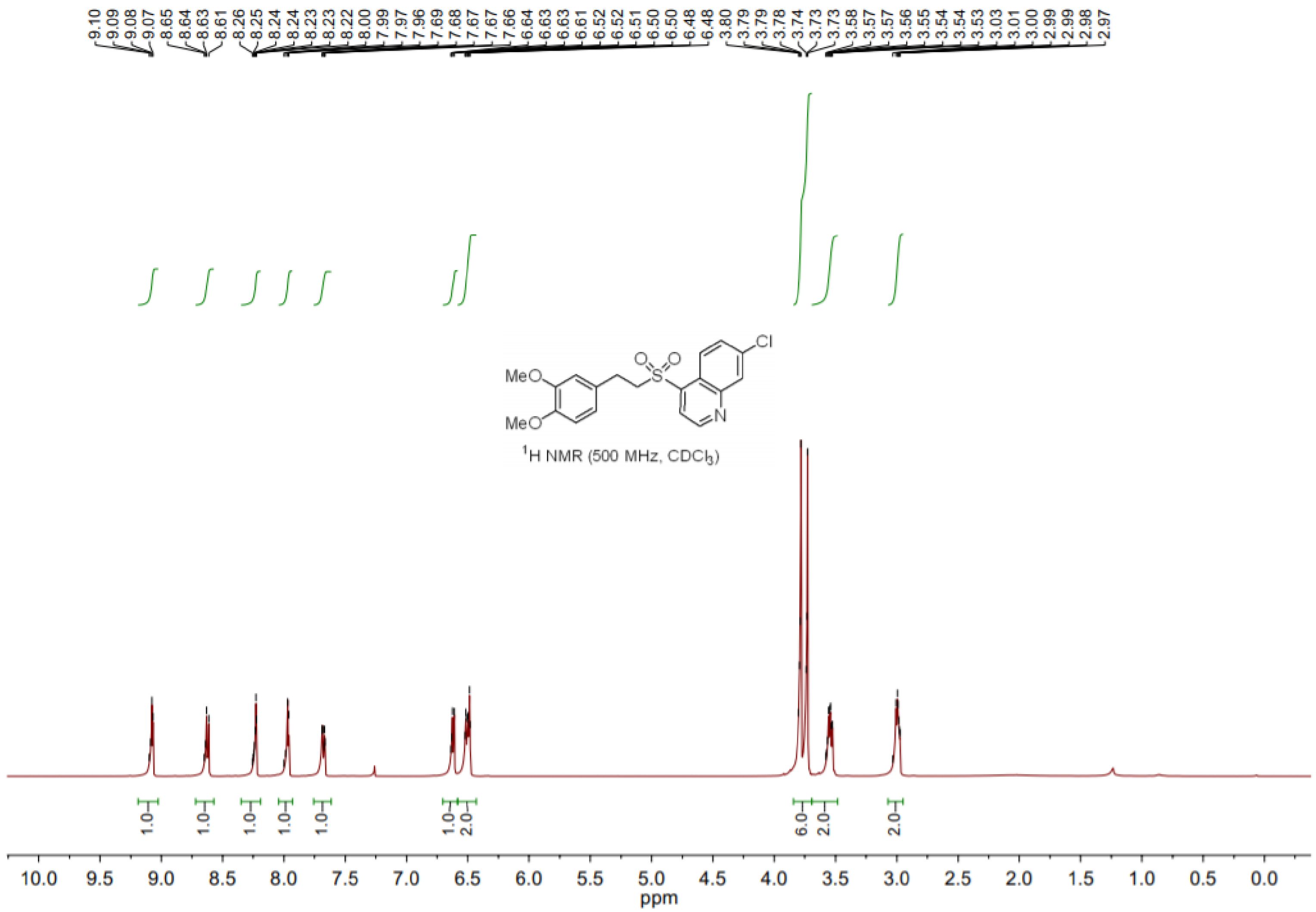

Go back to table of contents

7-Chloro-4-((3,4-dimethoxyphenethyl)sulfonyl)quinoline (2u)

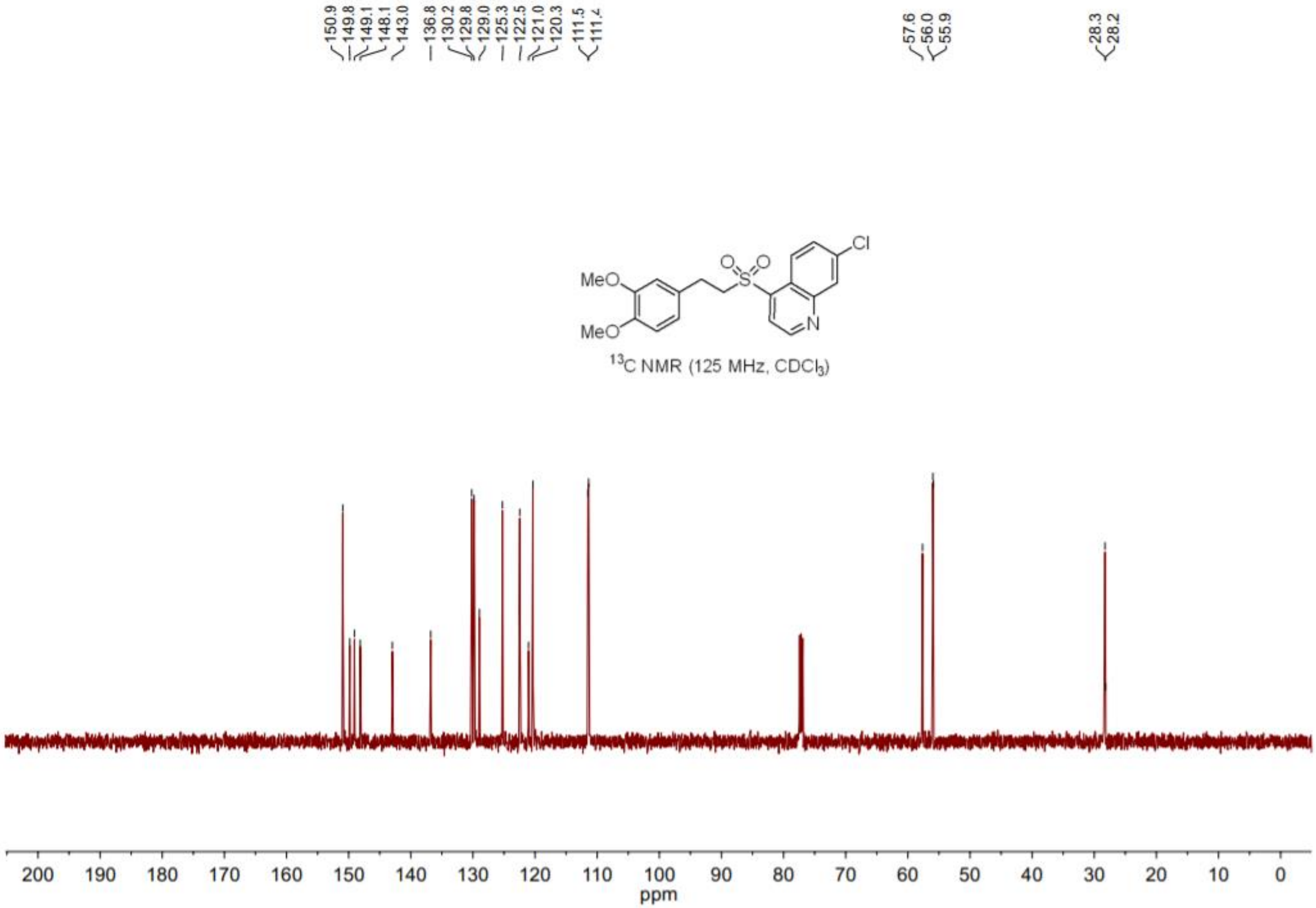

7-Chloro-4-((tetrahydro-2H-pyran-4-yl)sulfonyl)quinoline (2v)

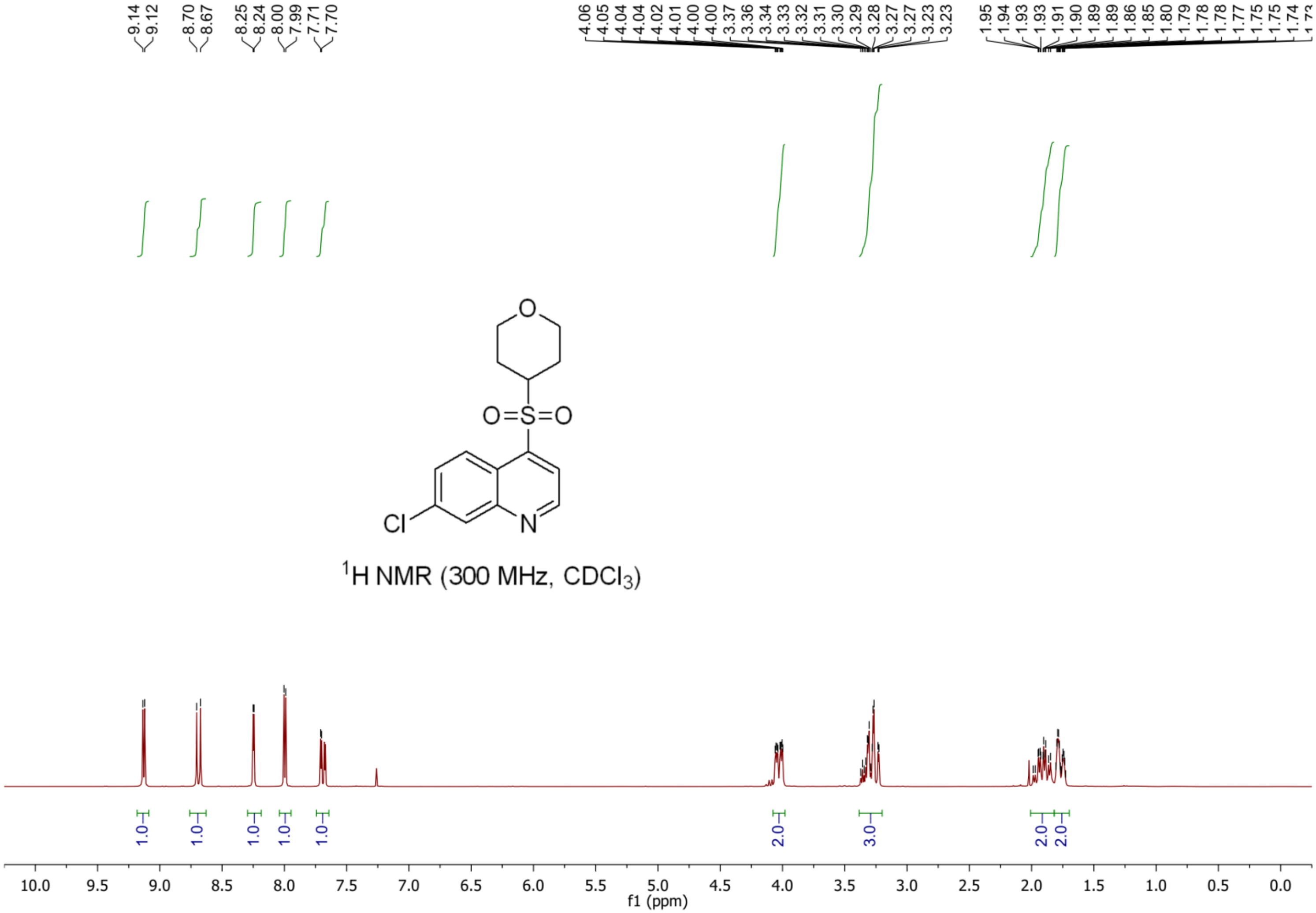

Go back to table of contents

7-Chloro-4-((tetrahydro-2H-pyran-4-yl)sulfonyl)quinoline (2v)

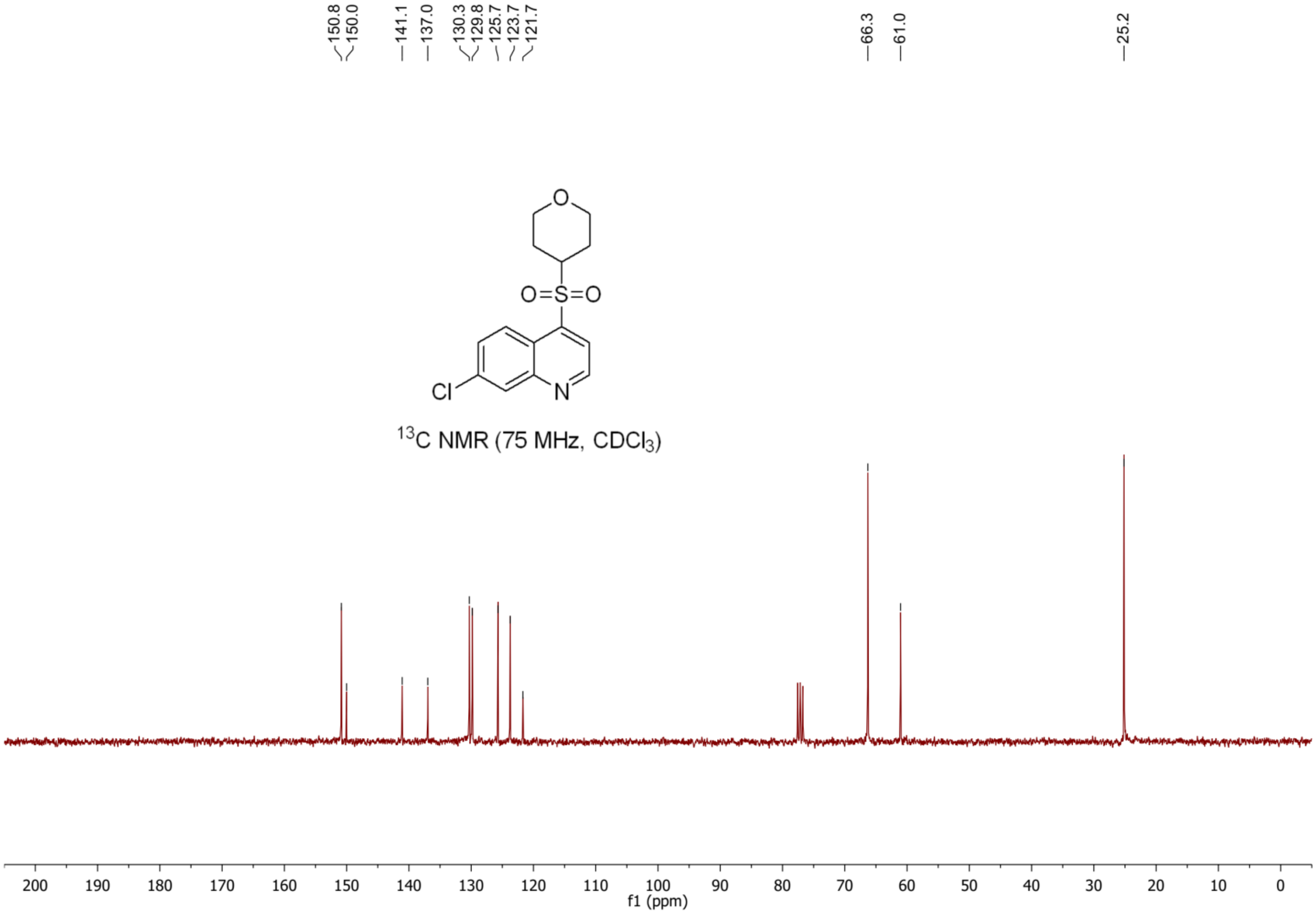

Go back to table of contents

Sodium 2-(4-fluorophenyl)ethane-1-sulfinate (3a)

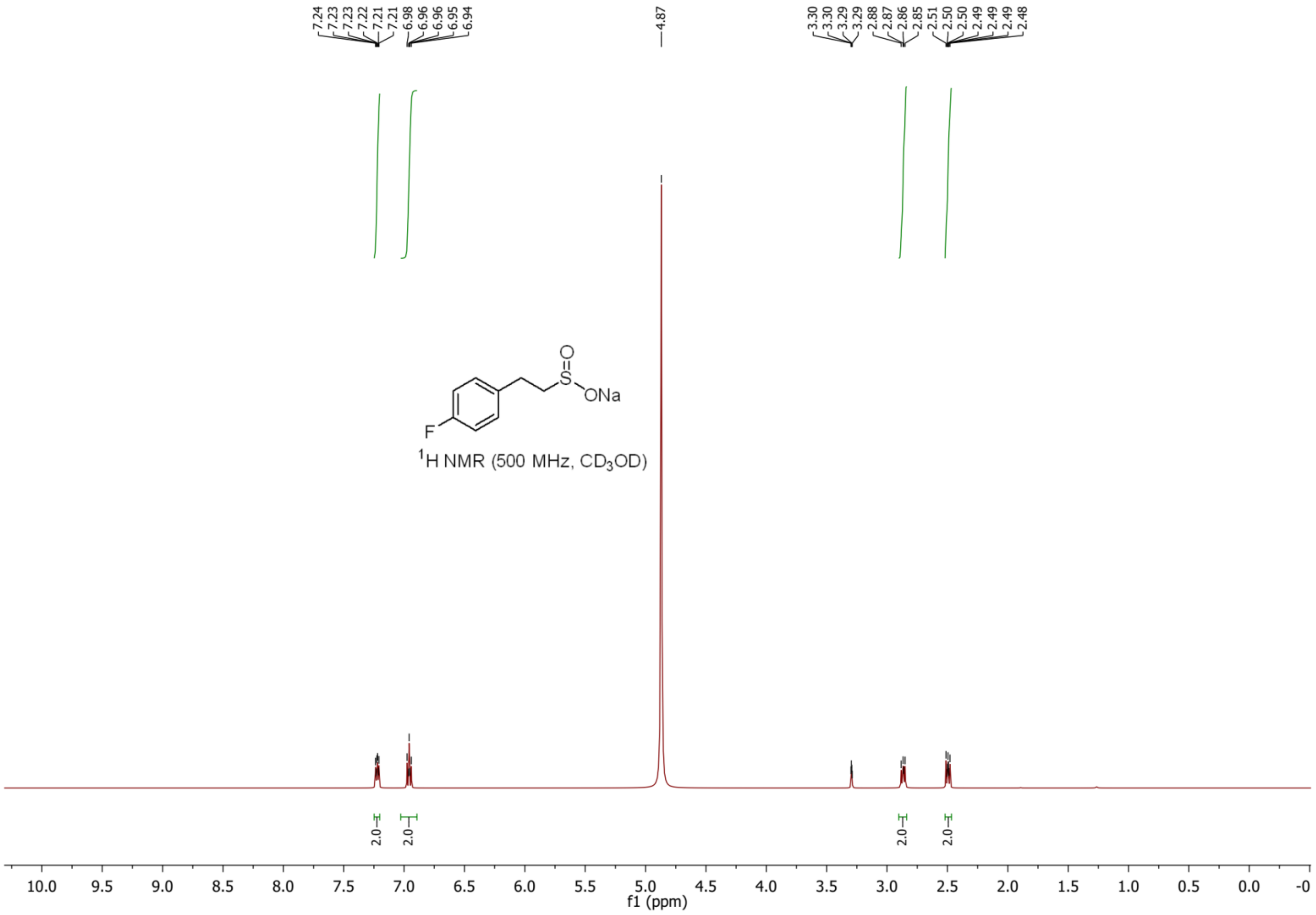

Go back to table of contents

Sodium 2-(4-fluorophenyl)ethane-1-sulfinate (3a)

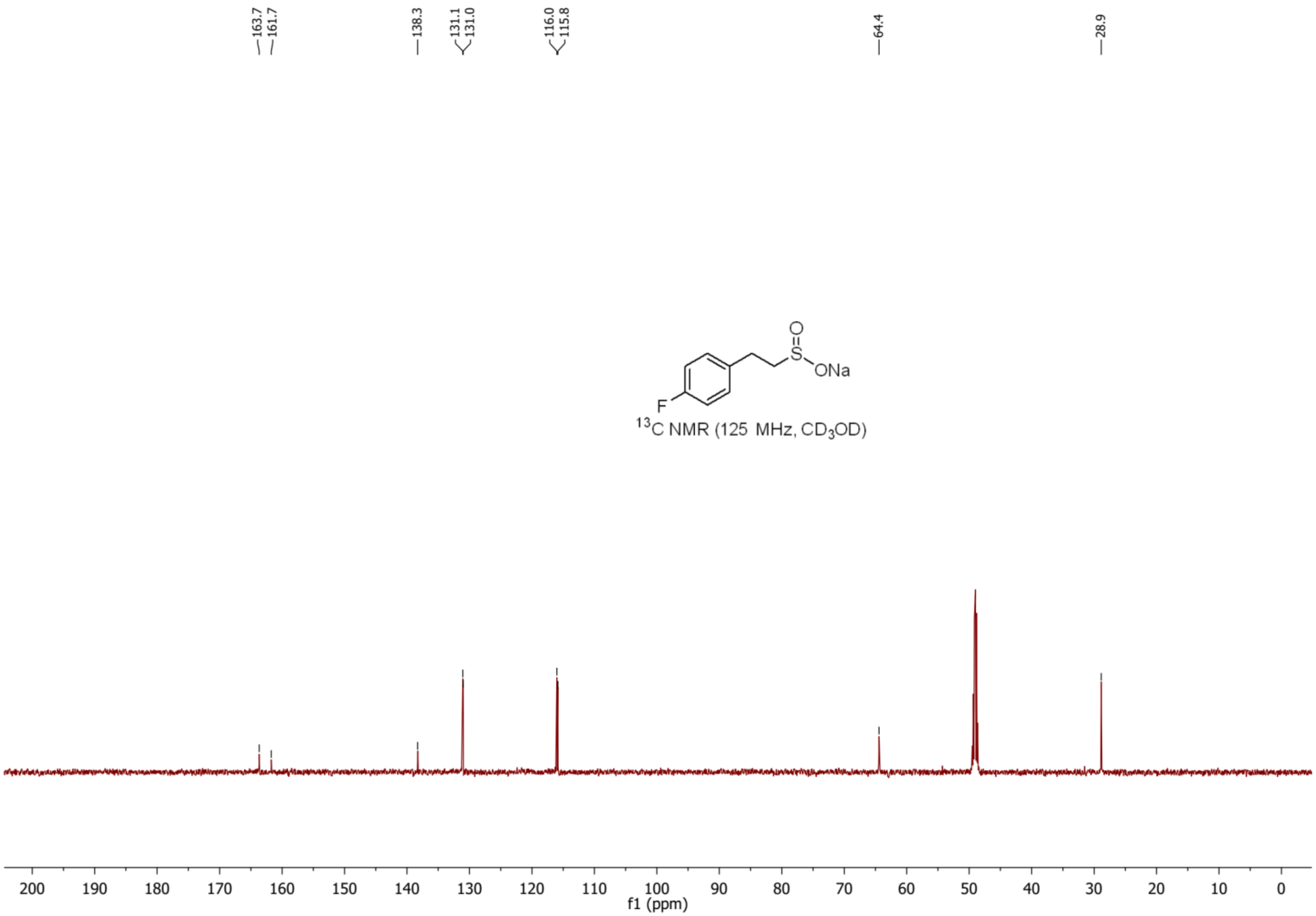

Go back to table of contents

Sodium 2-(3,4-dimethoxyphenyl)ethane-1-sulfinate (3b)

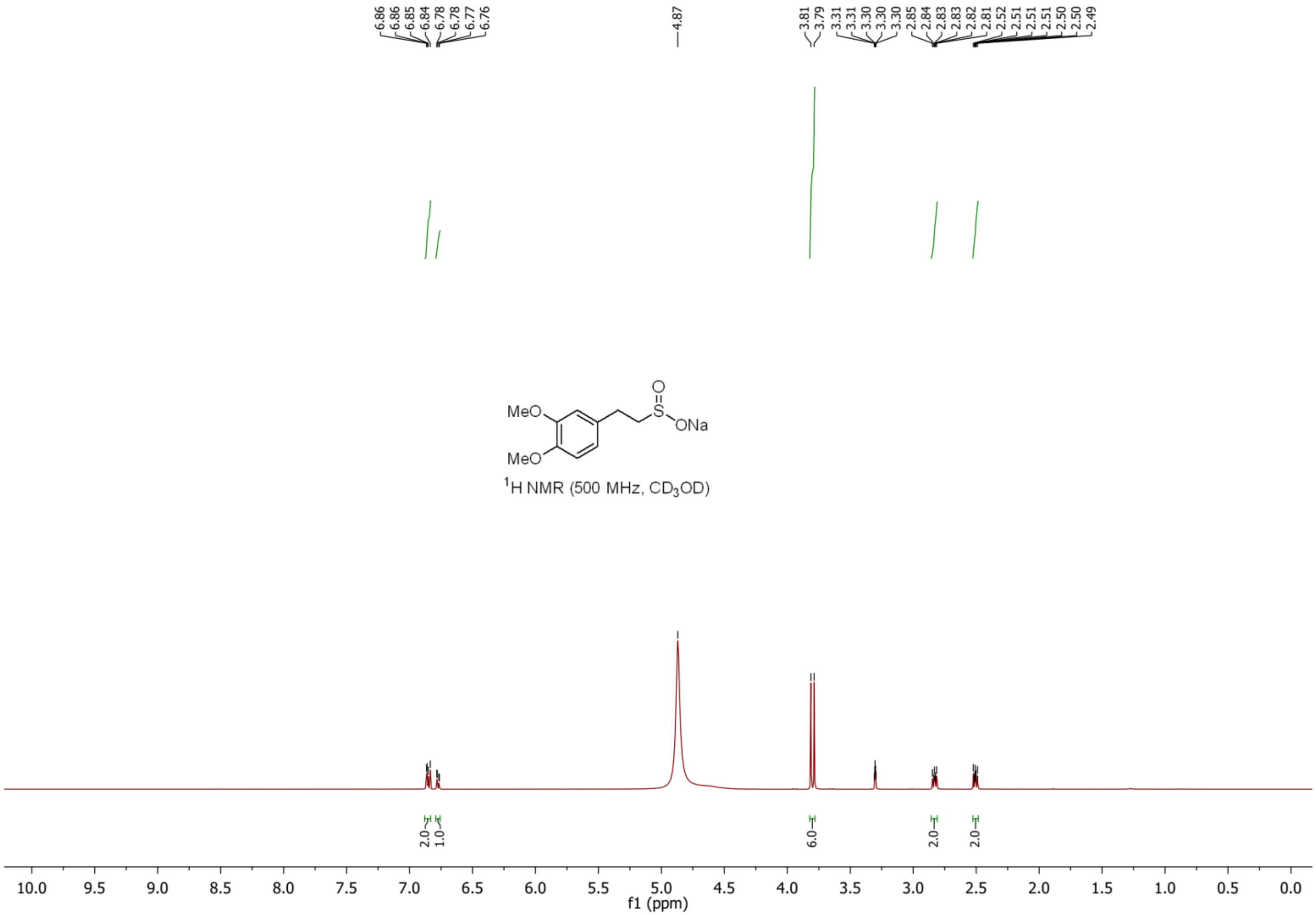

Sodium 2-(3,4-dimethoxyphenyl)ethane-1-sulfinate (3b)

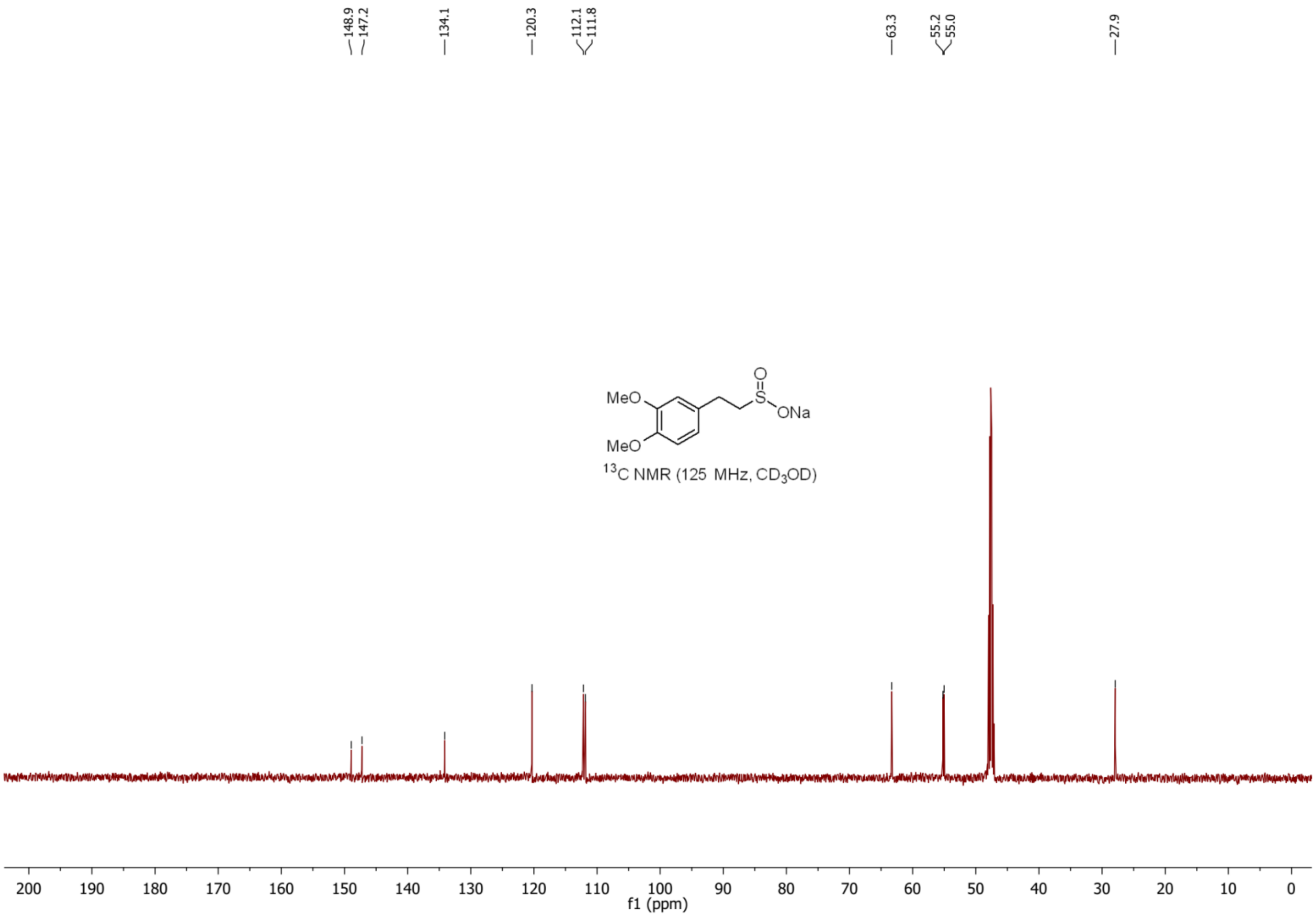

Go back to table of contents

Sodium 4-oxo-4-phenylbutane-1-sulfinate (3c)

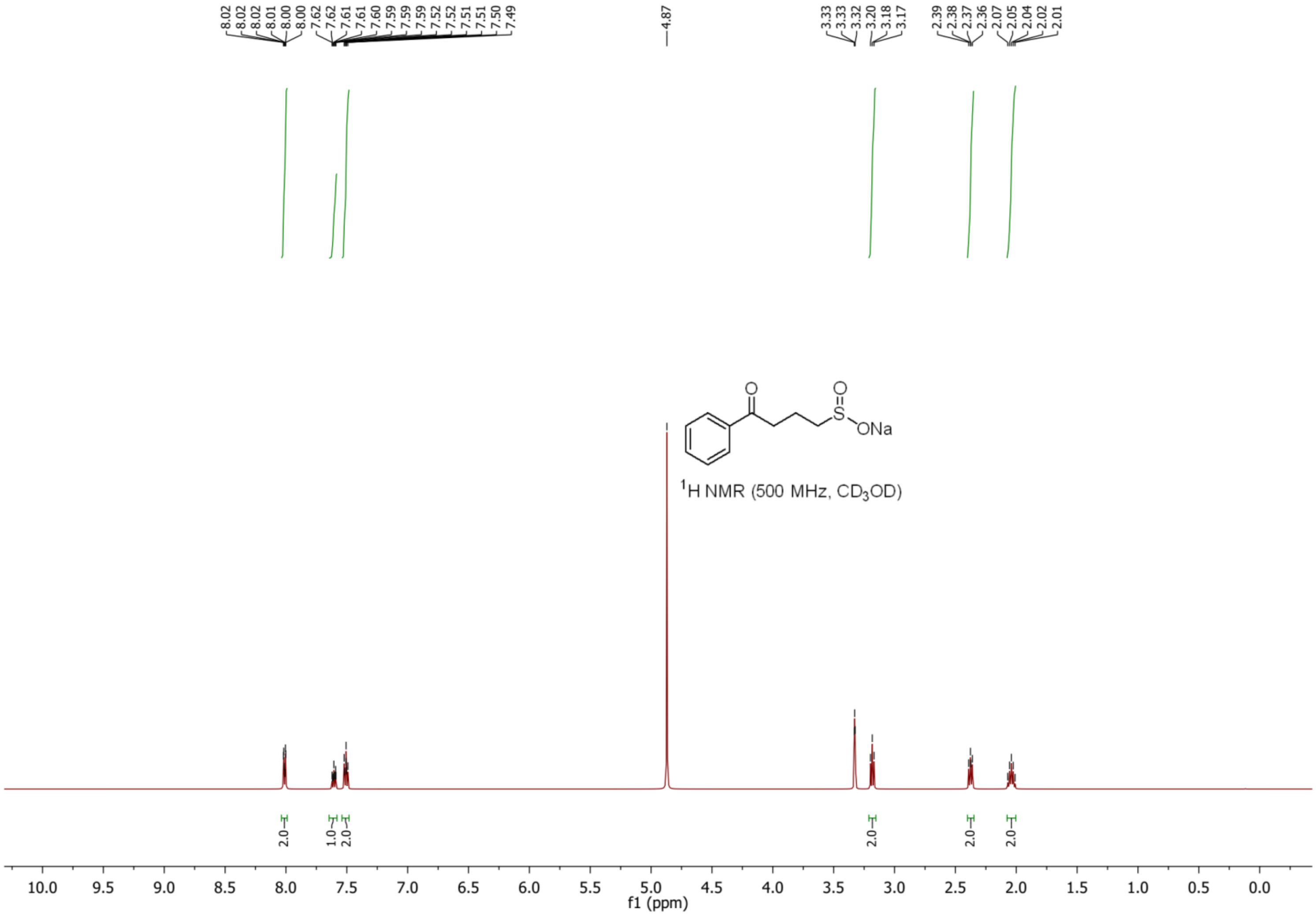

Go back to table of contents

Sodium 4-oxo-4-phenylbutane-1-sulfinate (3c)

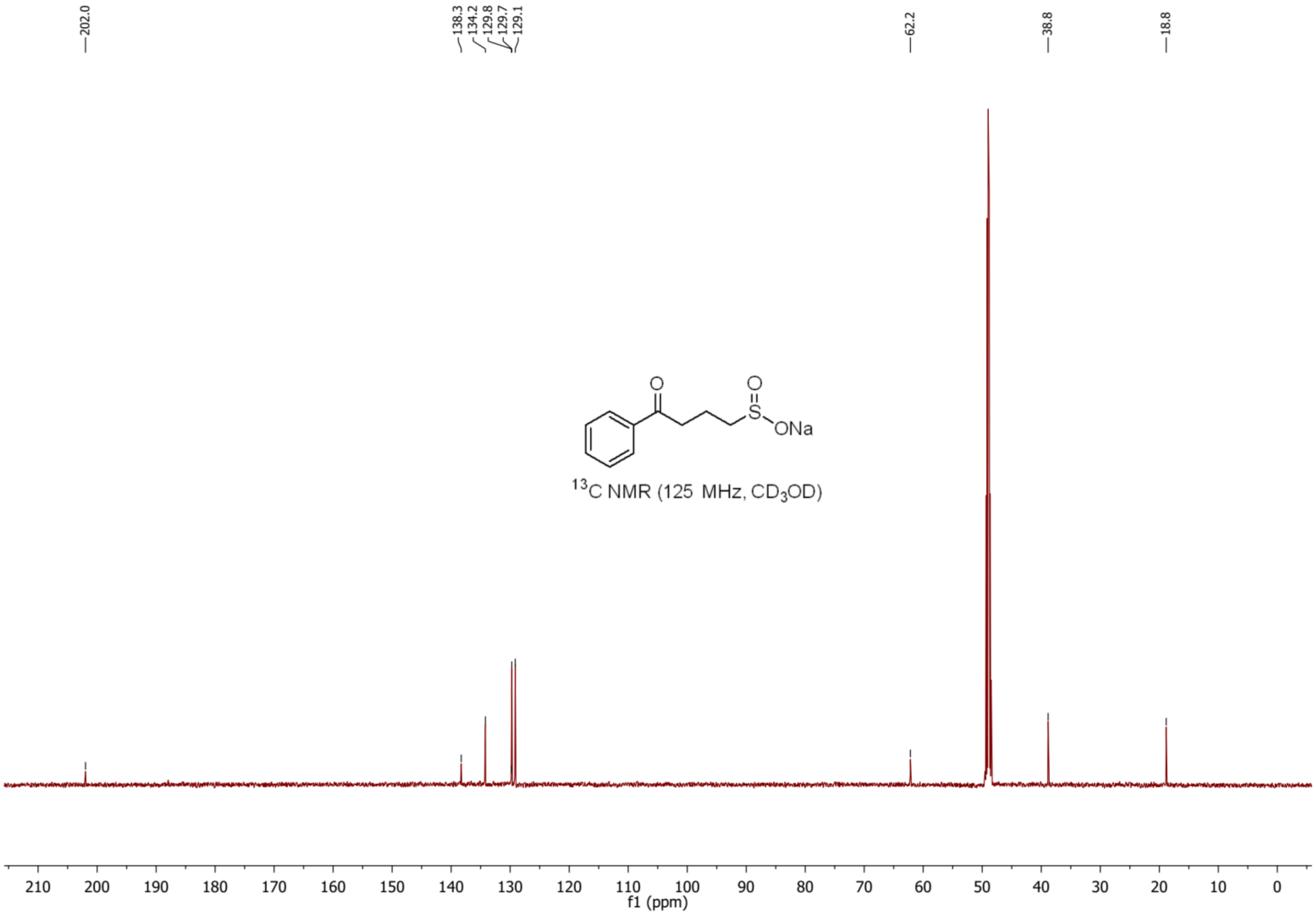

Go back to table of contents

Sodium 4-(5-methylthiophen-2-yl)-4-oxobutane-1-sulfinate (3d)

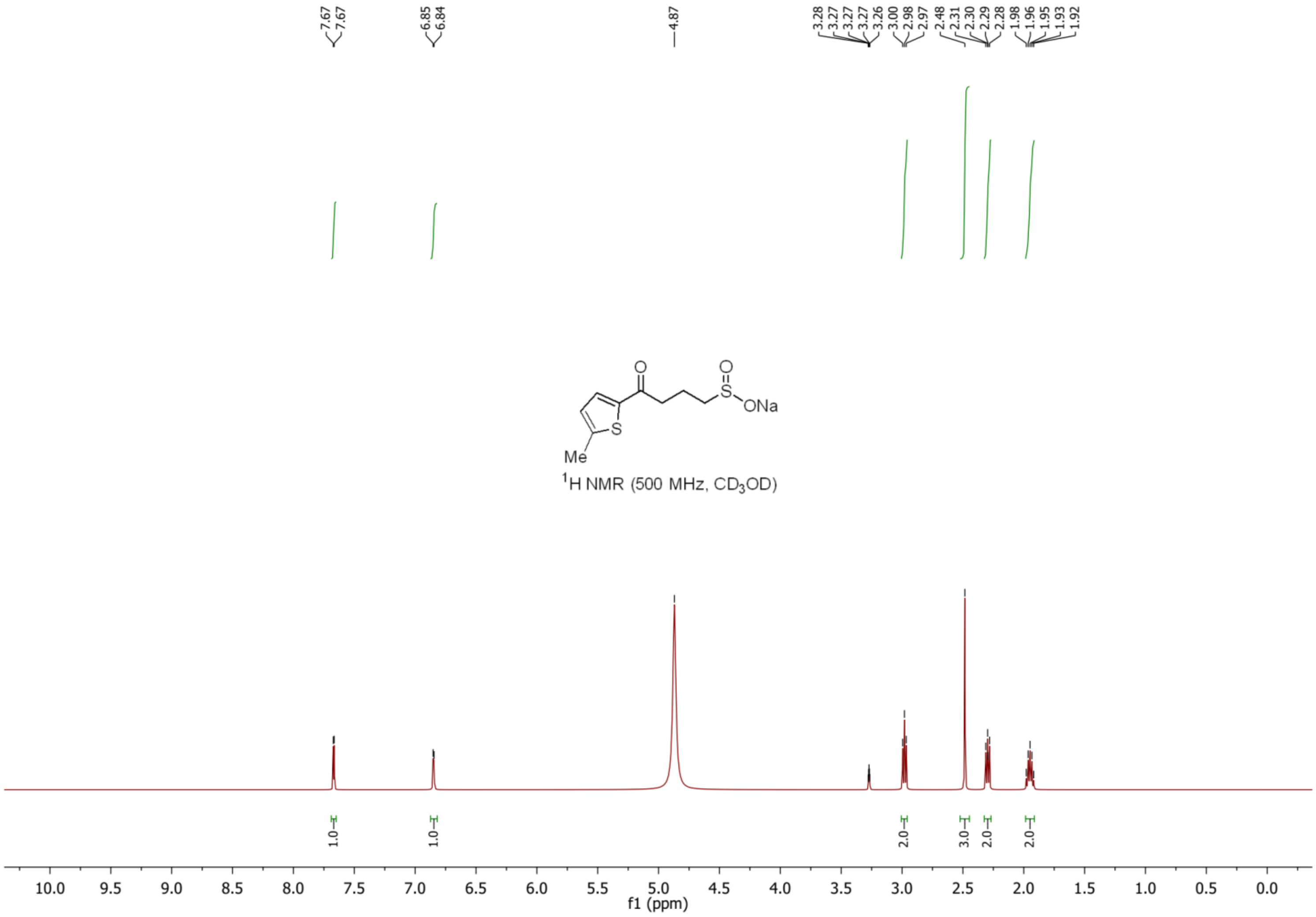

Sodium 4-(5-methylthiophen-2-yl)-4-oxobutane-1-sulfinate (3d)

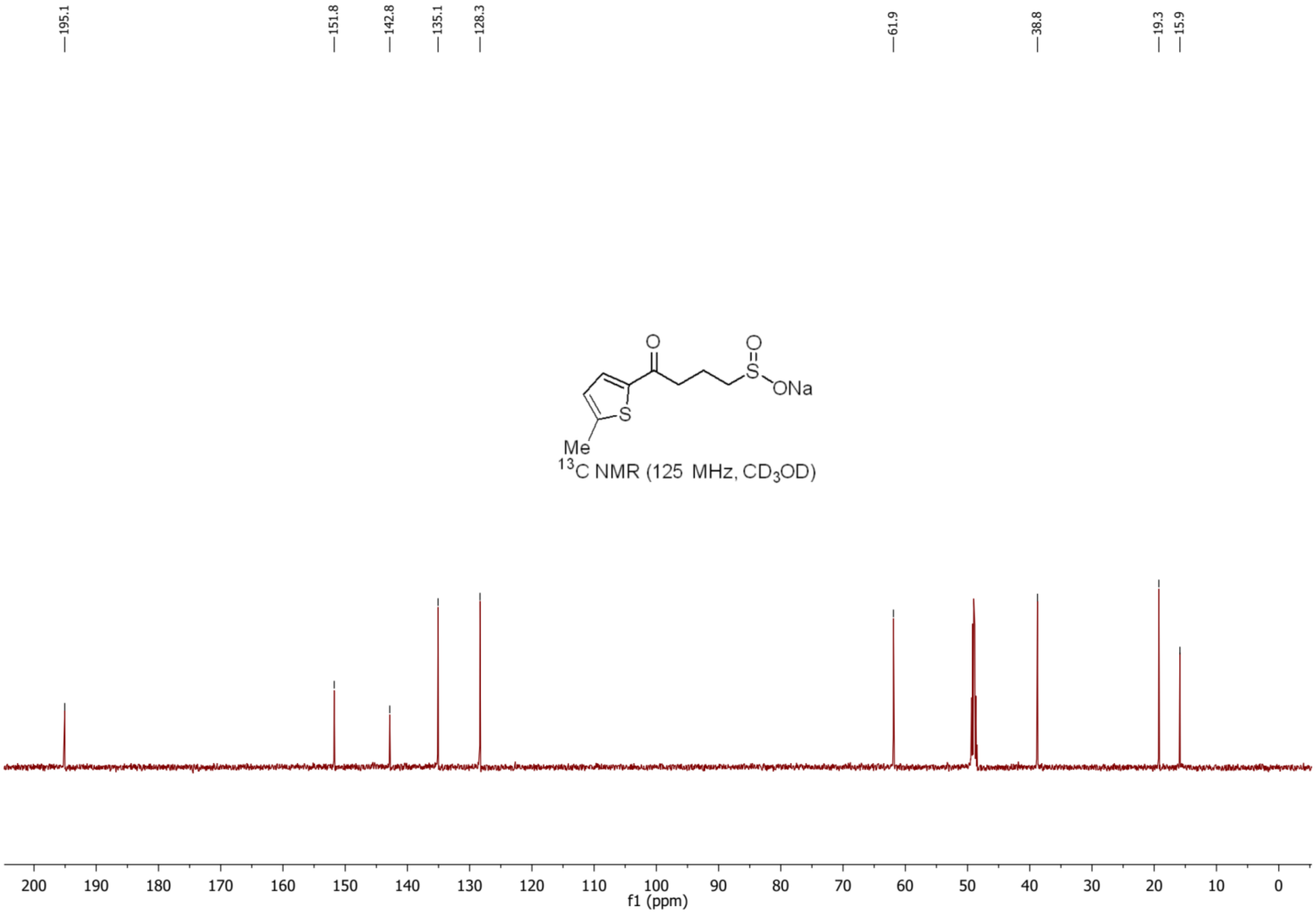

Go back to table of contents

Sodium 5-(4-chlorobenzamido)pentane-1-sulfinate (3e)

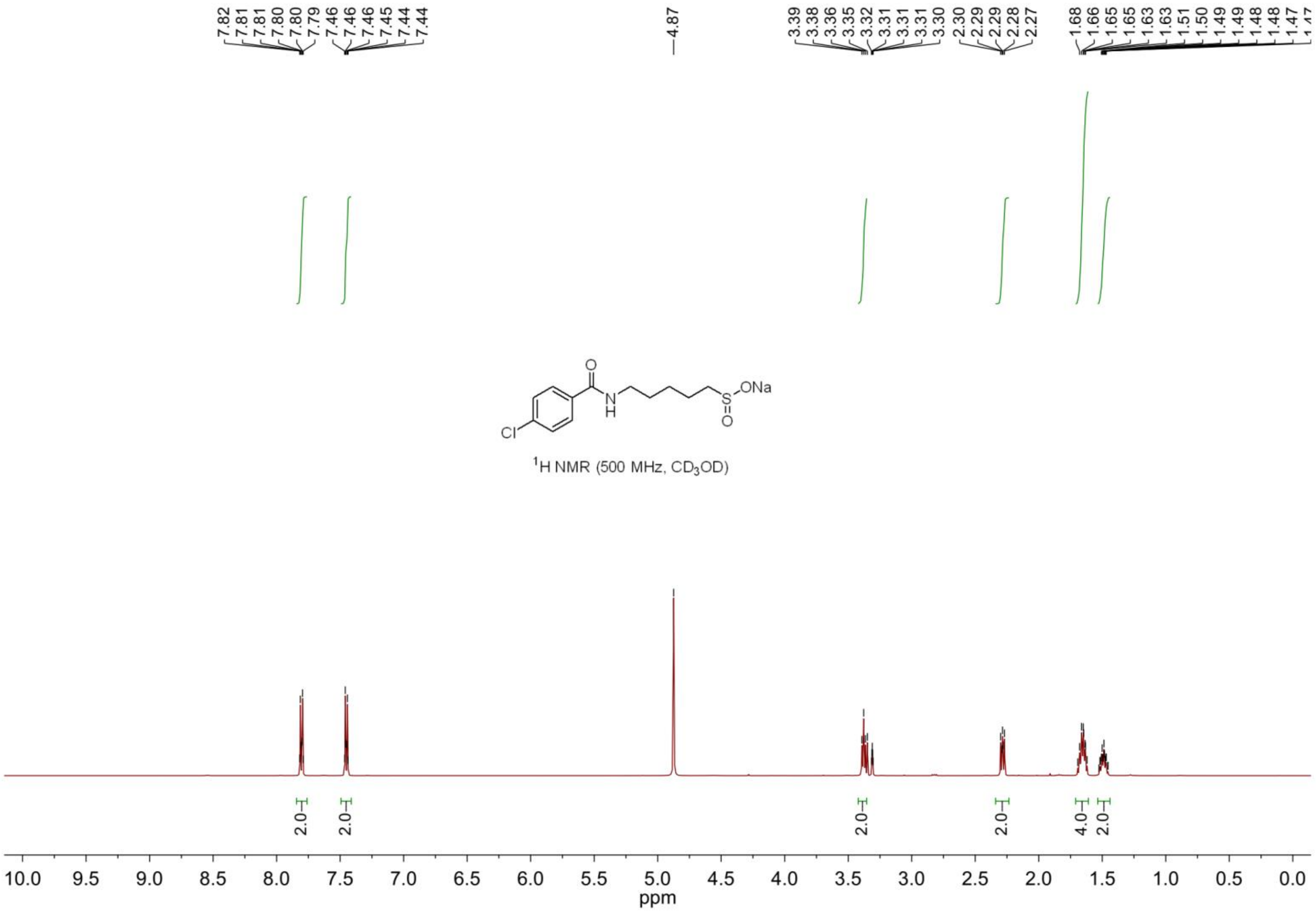

Sodium 5-(4-chlorobenzamido)pentane-1-sulfinate (3e)

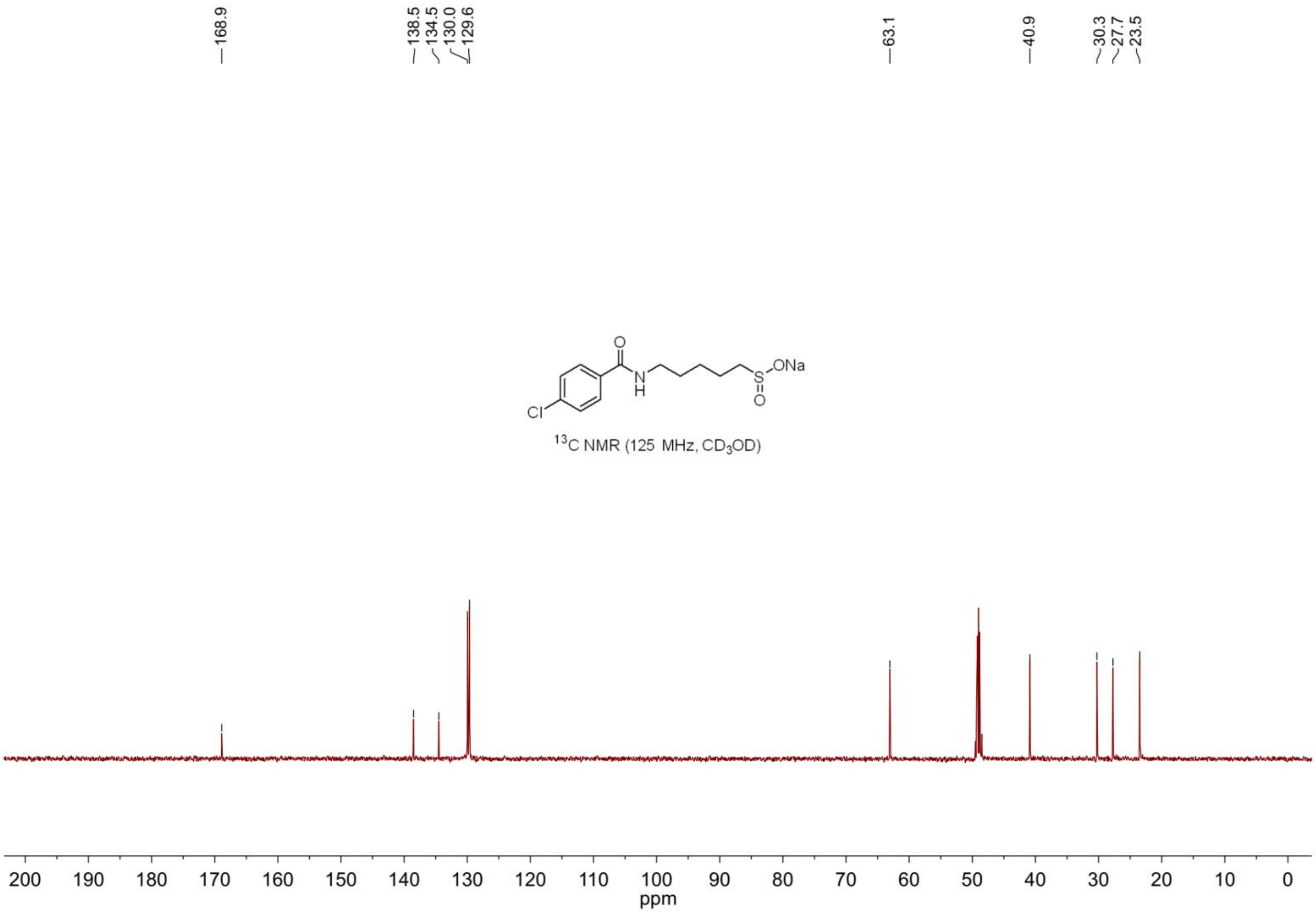

Go back to table of contents

Sodium ((1*R,3*S**)-3-acetyl-2,2-dimethylcyclobutyl)methanesulfinate (3f)**

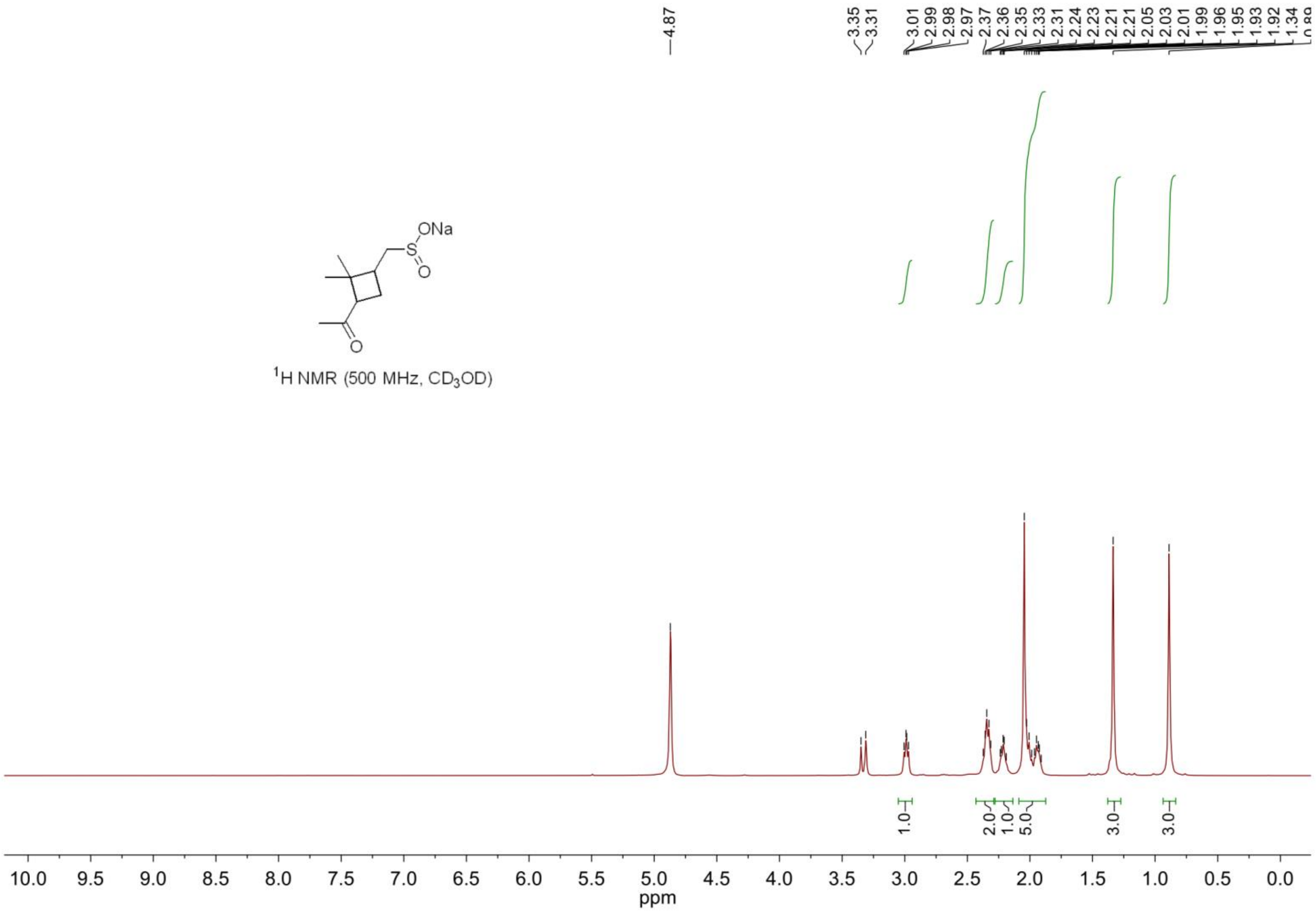

Go back to table of contents

Sodium ((1*R,3*S**)-3-acetyl-2,2-dimethylcyclobutyl)methanesulfinate (3f)**

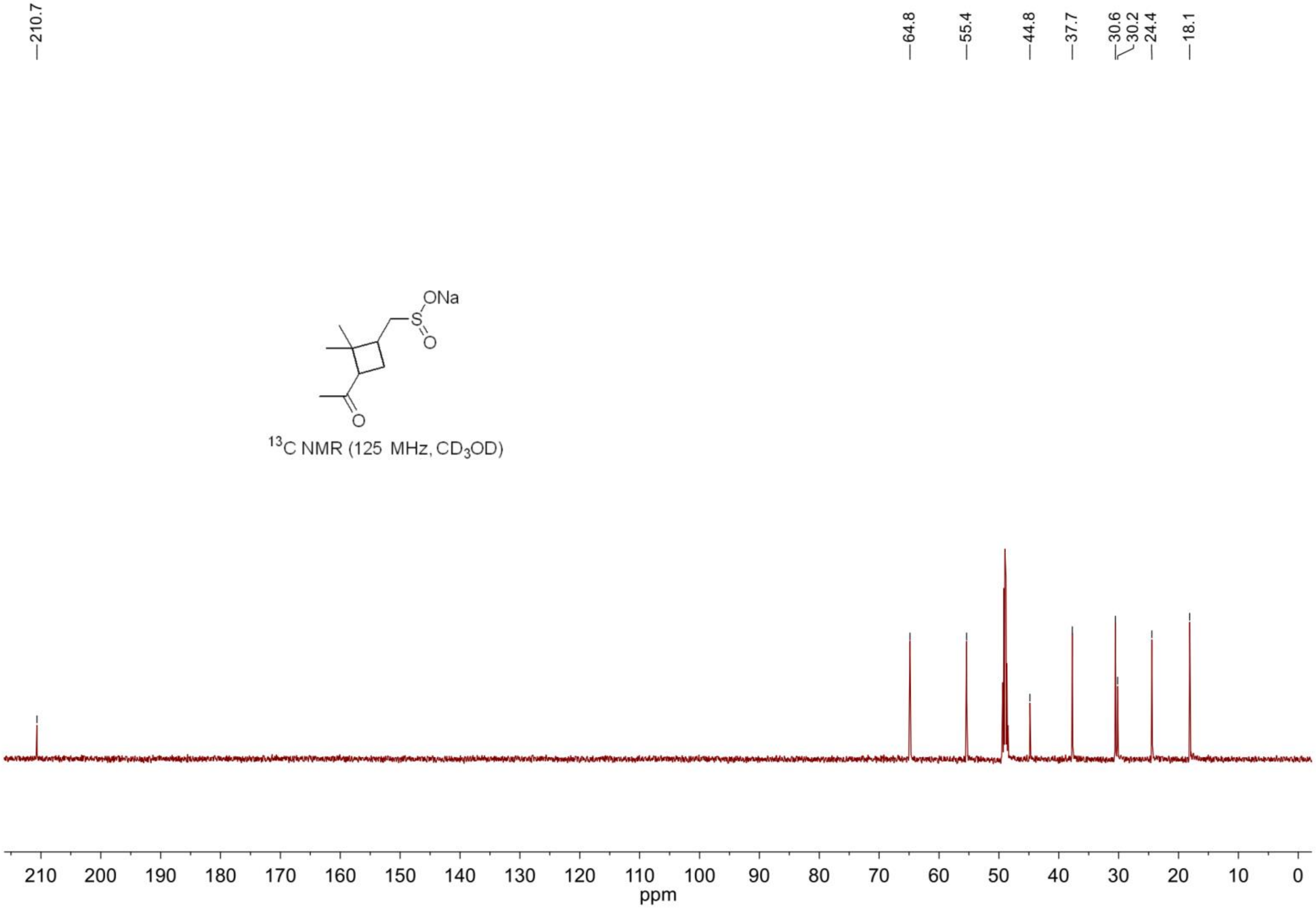

Go back to table of contents

Sodium 3-(1*H*-indol-2-yl)propane-1-sulfinate (3g)

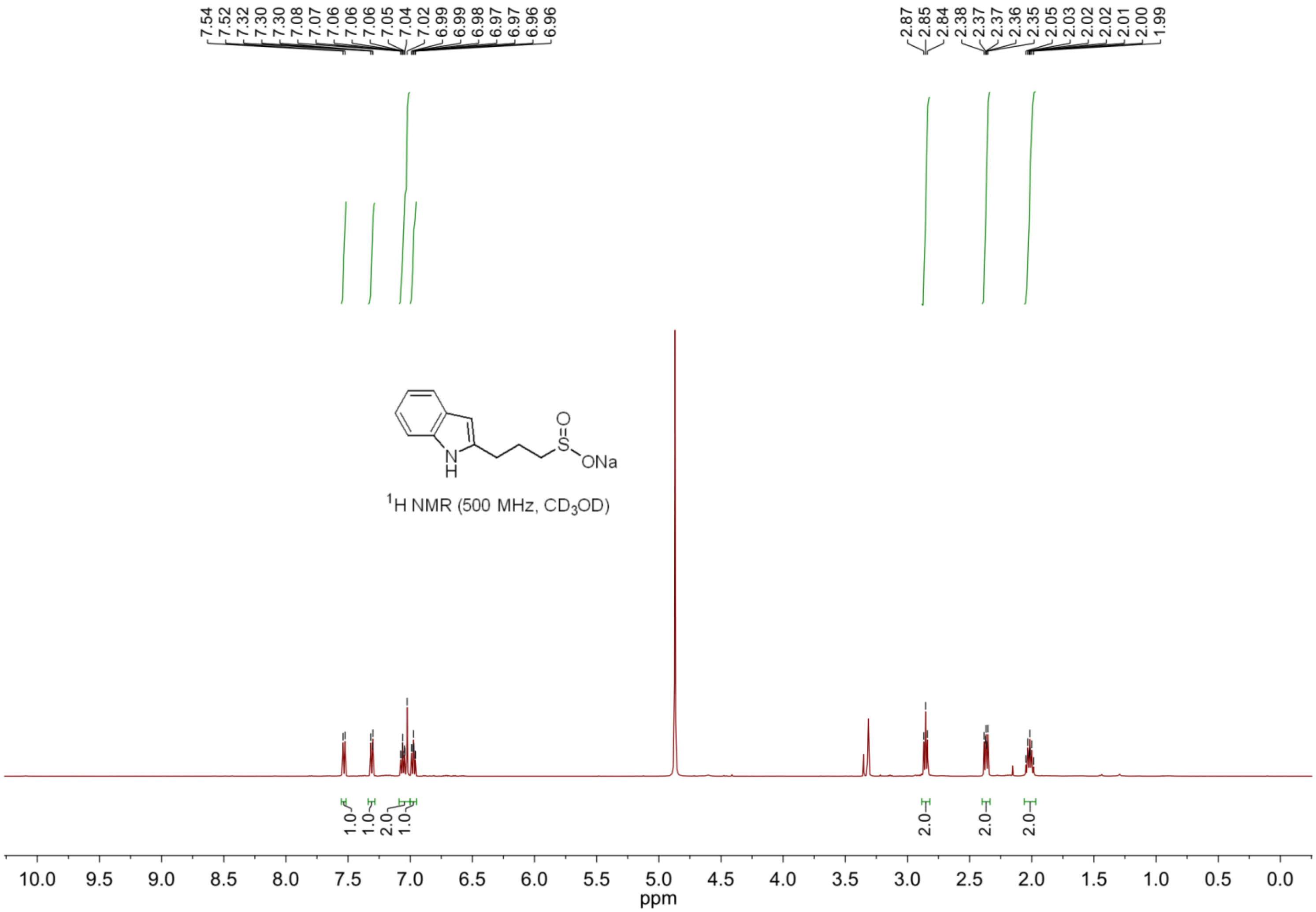

Go back to table of contents

Sodium 3-(1*H*-indol-2-yl)propane-1-sulfinate (3g)

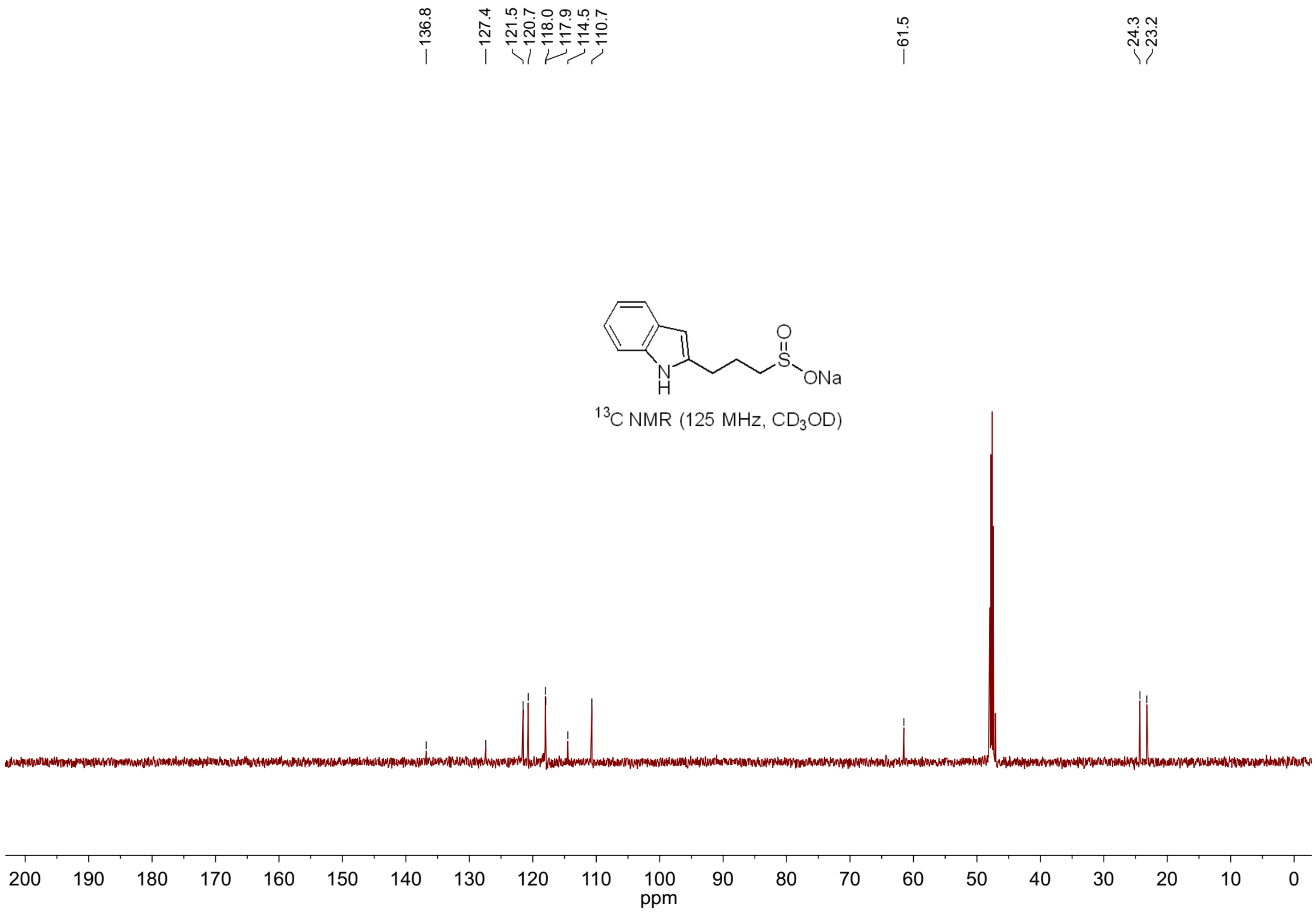

Go back to table of contents

Sodium 5-(4-(trifluoromethyl)benzamido)pentane-1-sulfinate (3h)

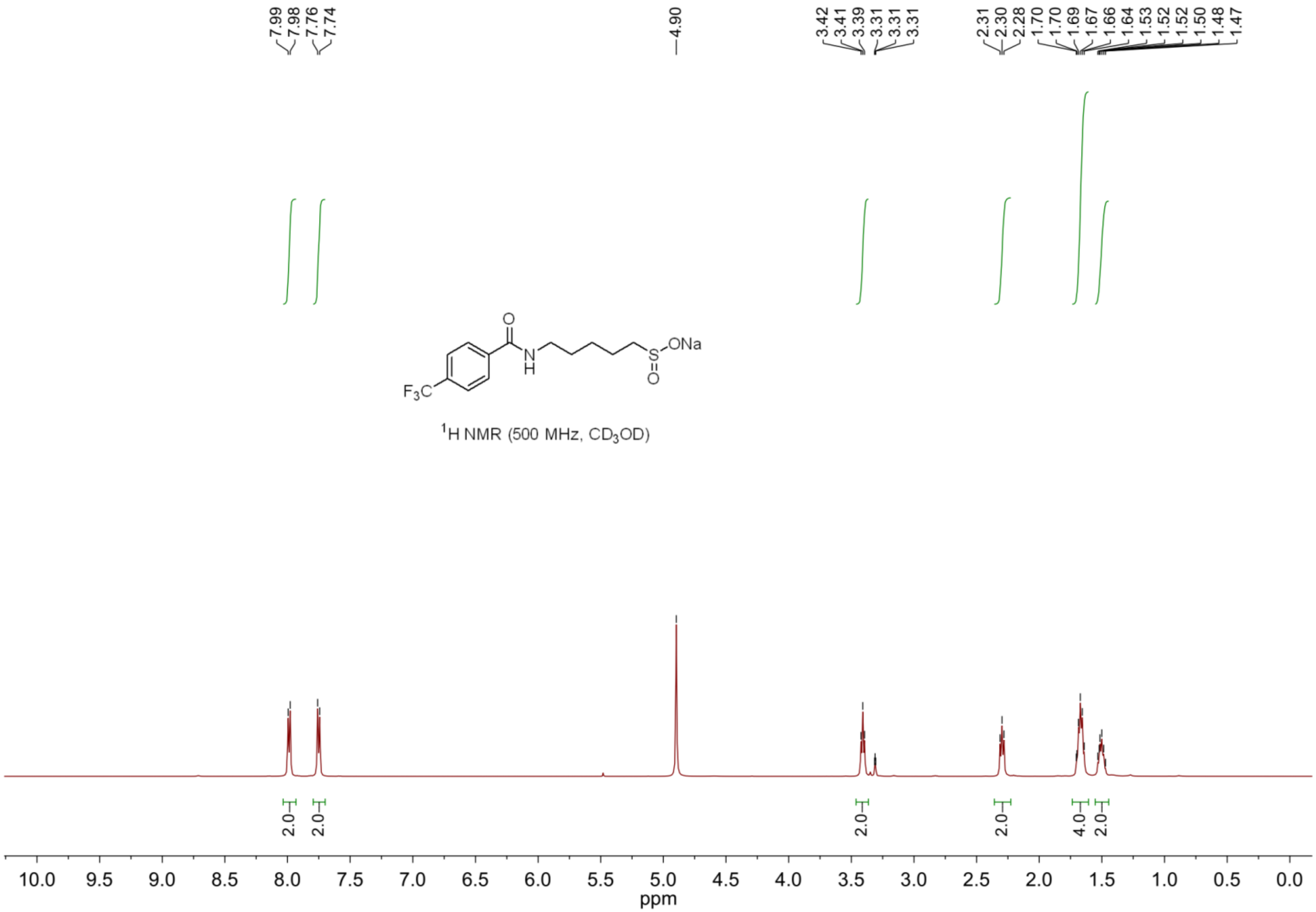

Go back to table of contents

Sodium 5-(4-(trifluoromethyl)benzamido)pentane-1-sulfinate (3h)

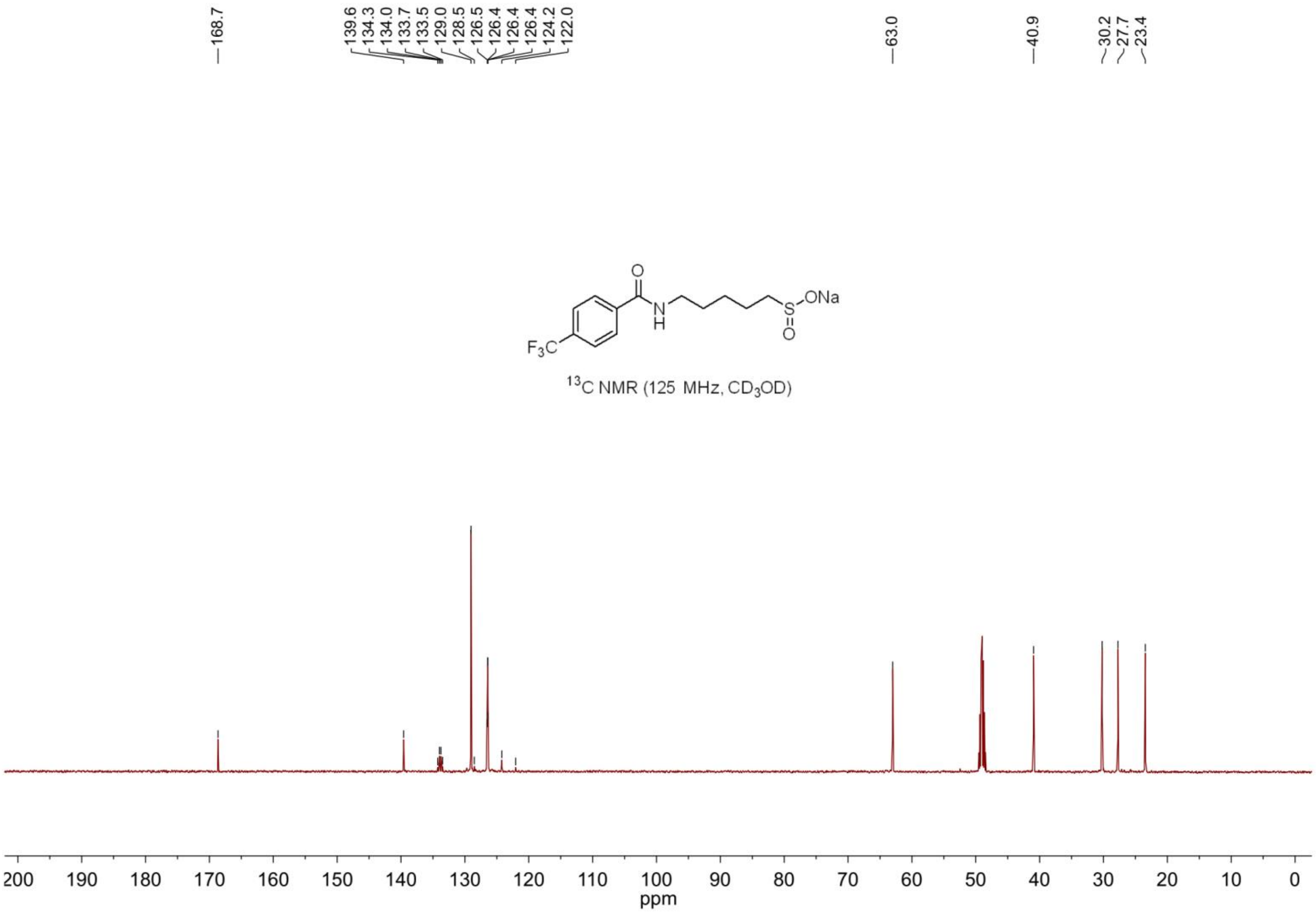

Go back to table of contents

Sodium tetrahydro-2*H*-pyran-4-sulfinate (3i)

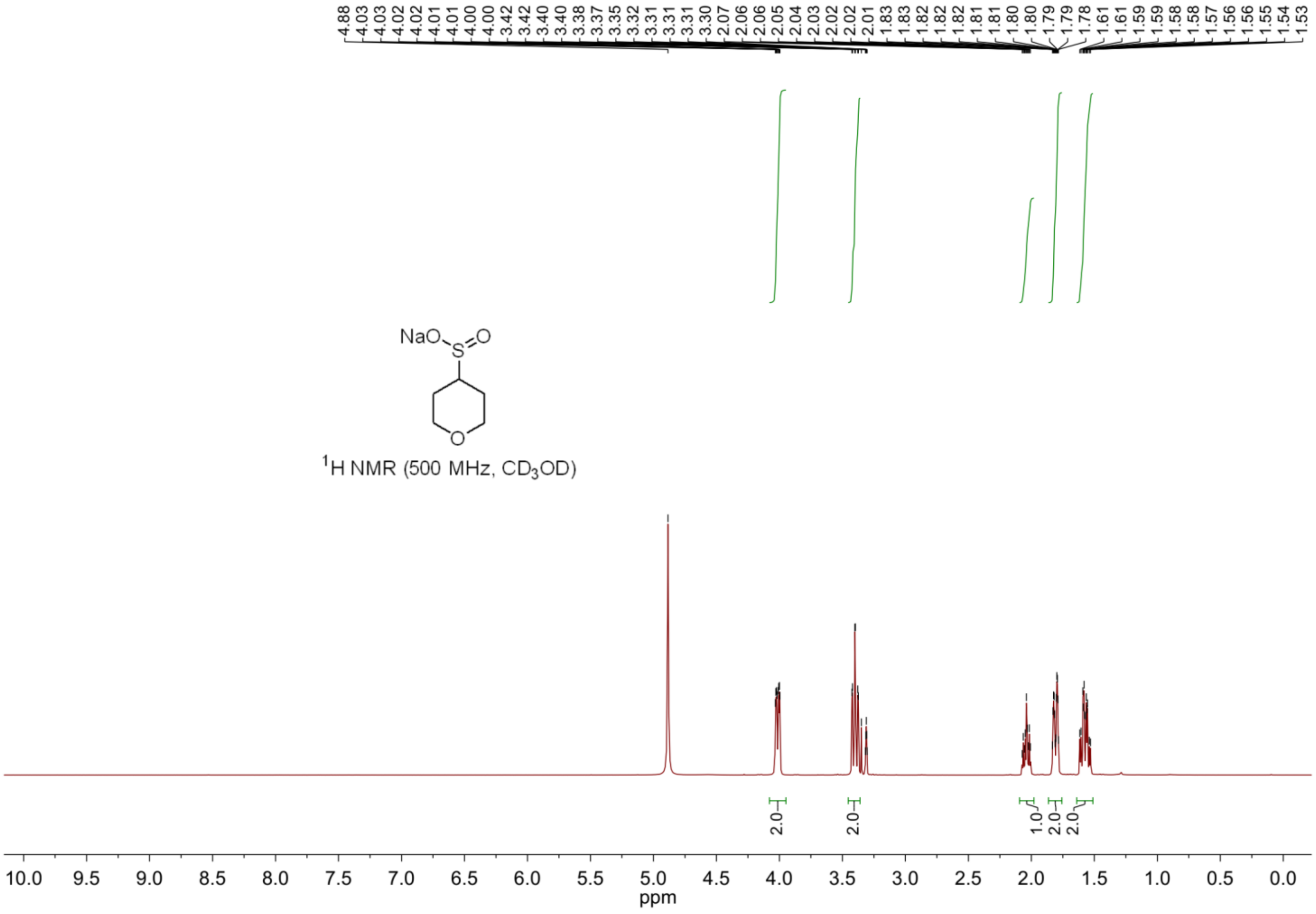

Go back to table of contents

Sodium tetrahydro-2*H*-pyran-4-sulfinate (3i)

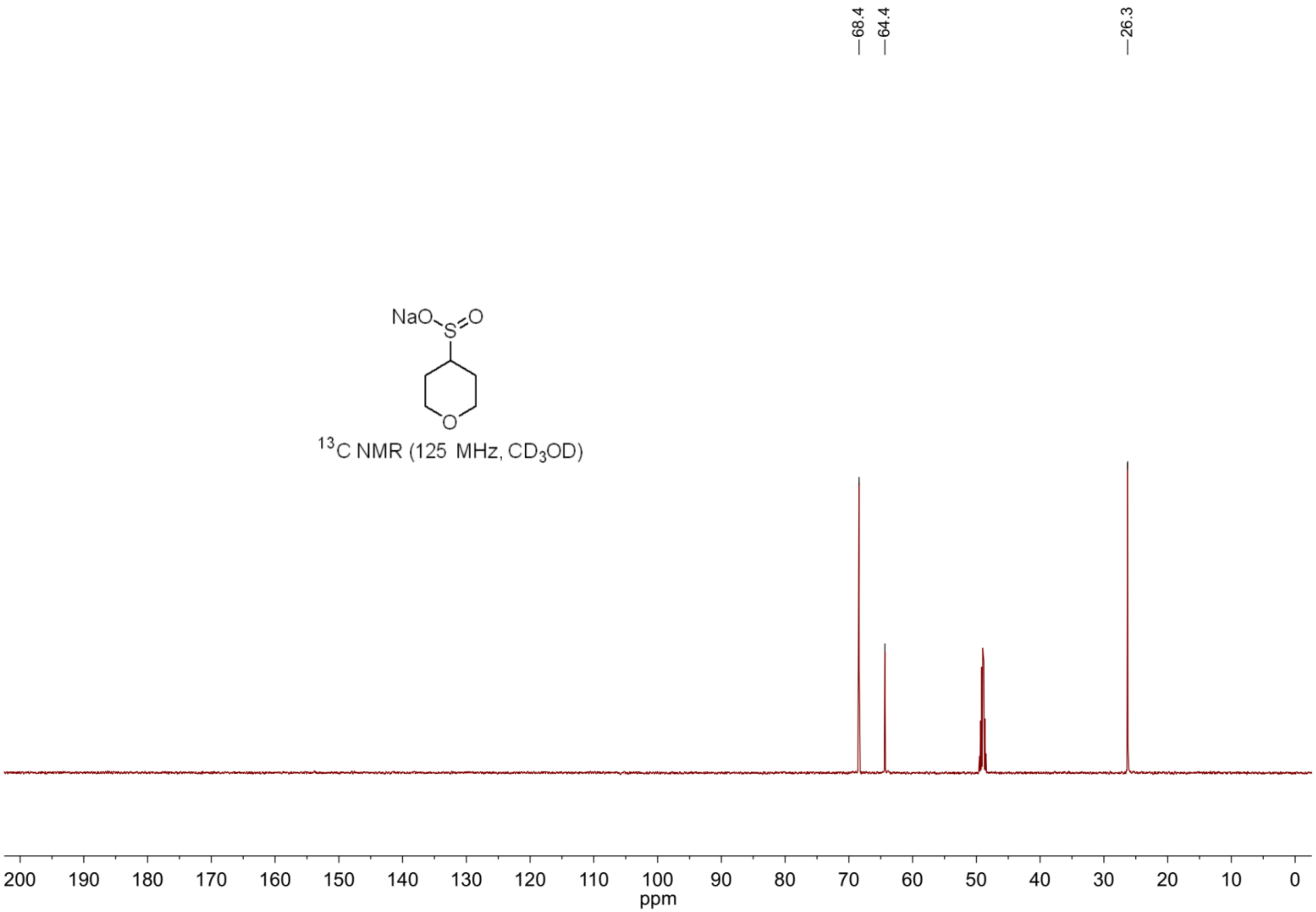

Go back to table of contents

Sodium 1-(*tert*-butoxycarbonyl)piperidine-4-sulfinate (3j)

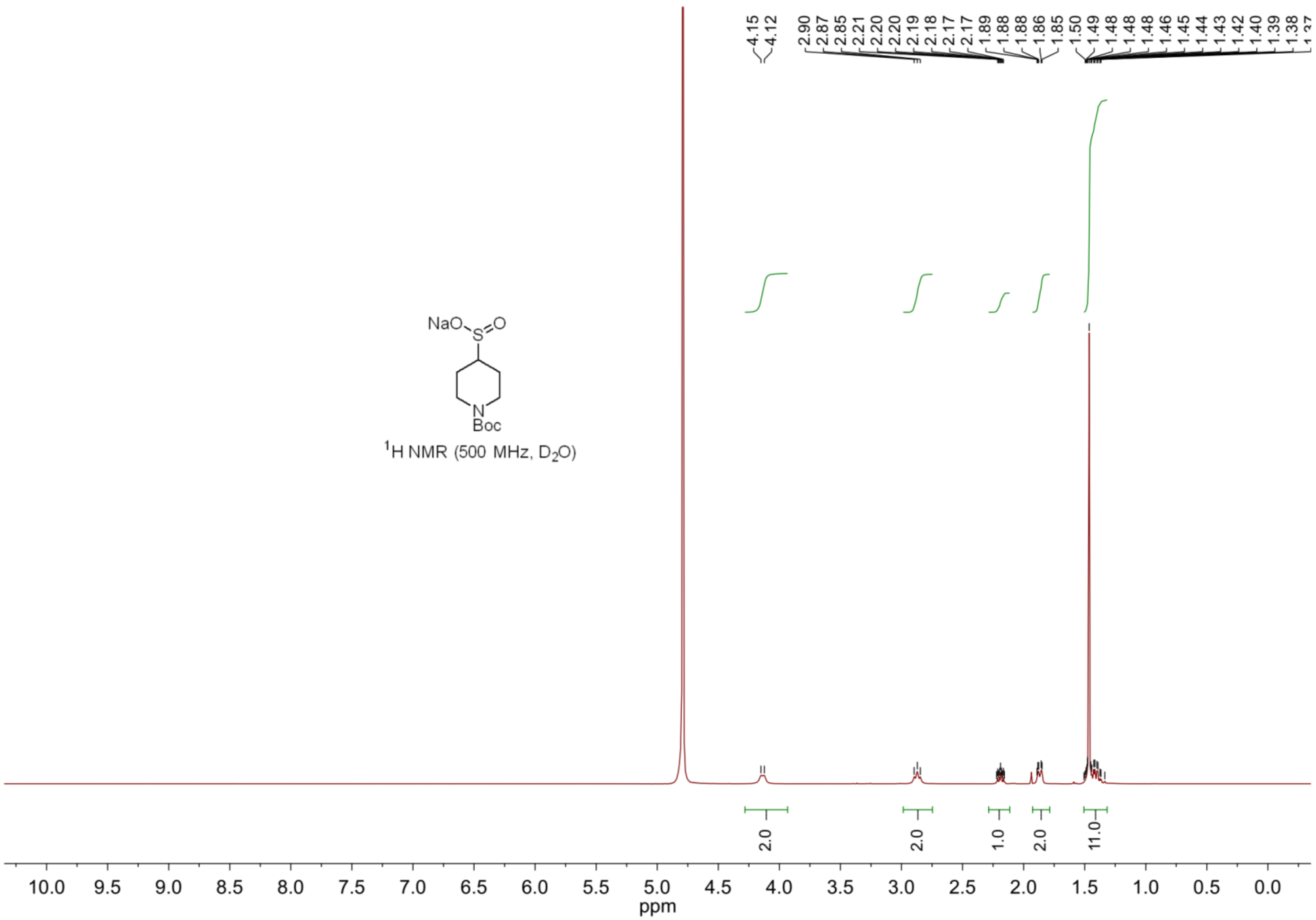

Go back to table of contents

Sodium 1-(*tert*-butoxycarbonyl)piperidine-4-sulfinate (3j)

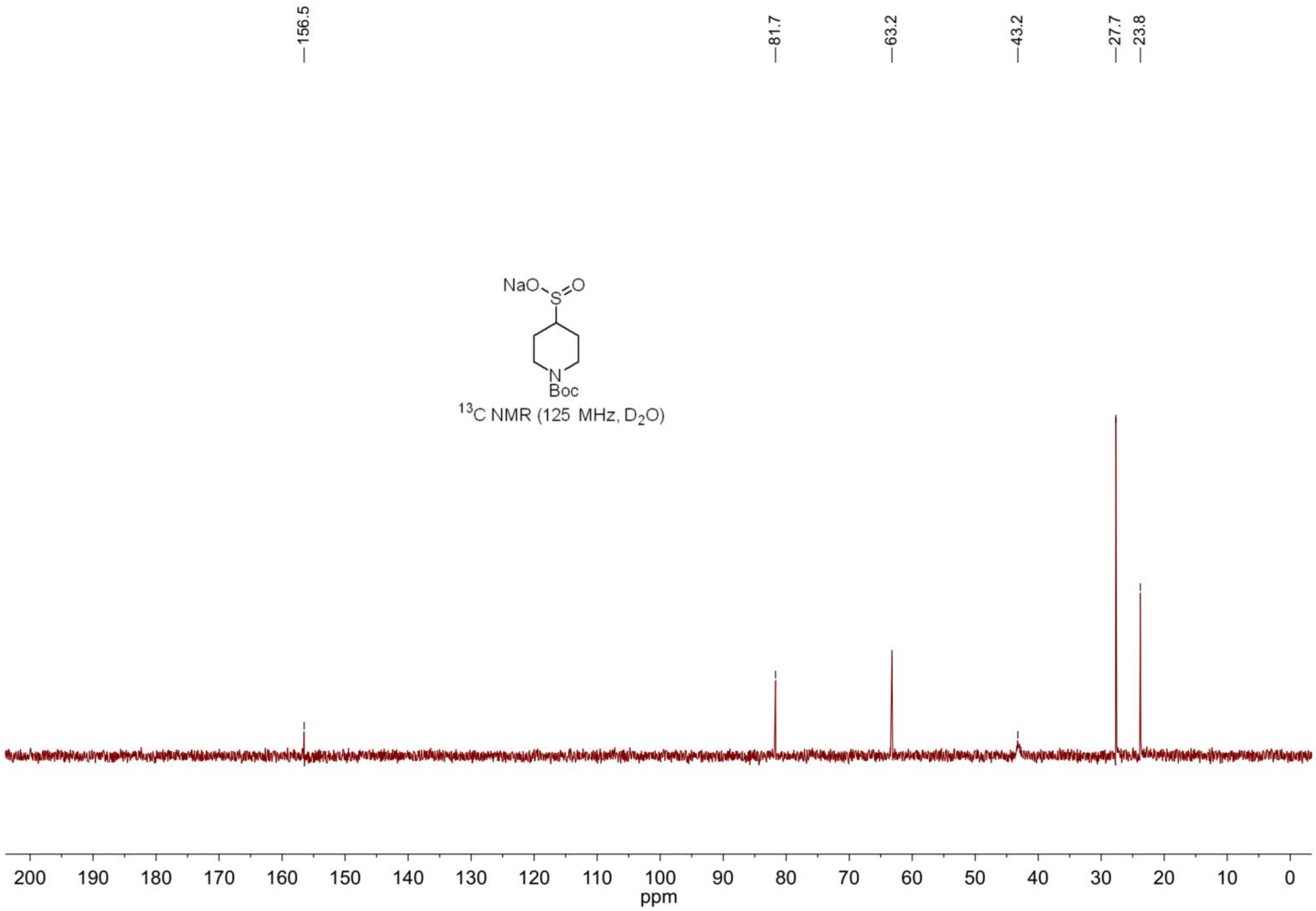

Go back to table of contents

Sodium 4,4-difluorocyclohexane-1-sulfinate (3k)

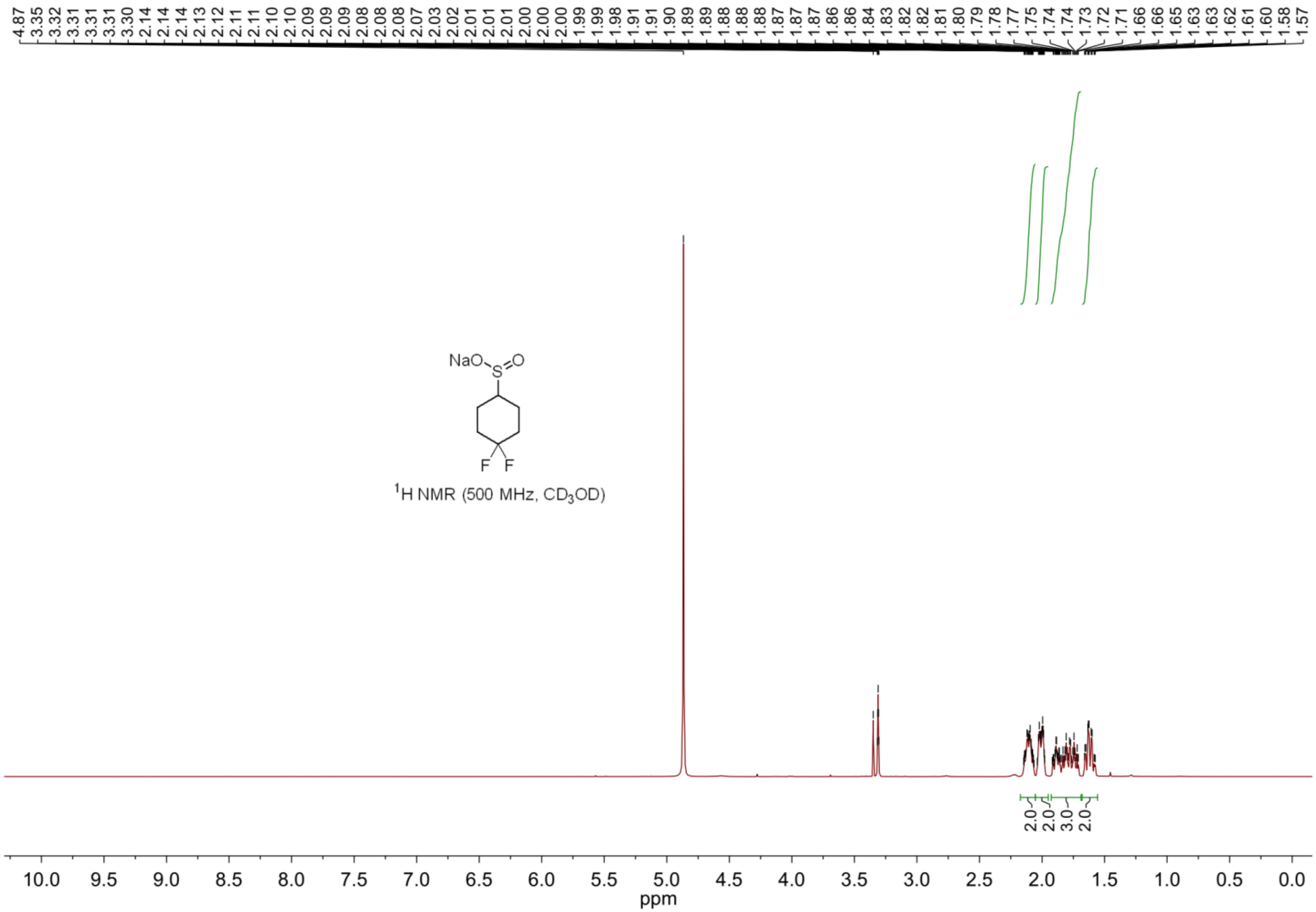

Go back to table of contents

Sodium 4,4-difluorocyclohexane-1-sulfinate (3k)

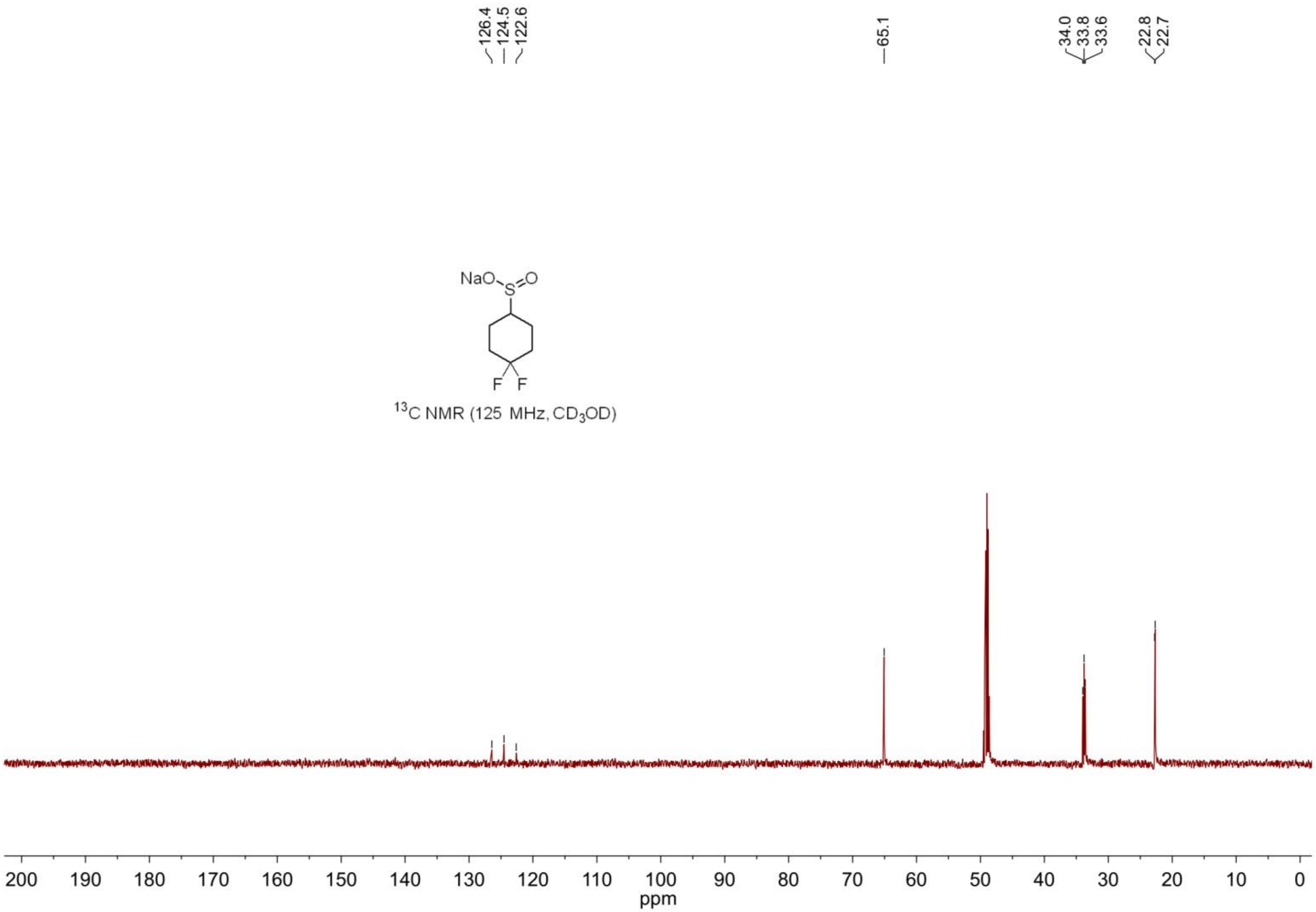

Sodium 2,3-dihydro-1*H*-indene-2-sulfinate (3l)

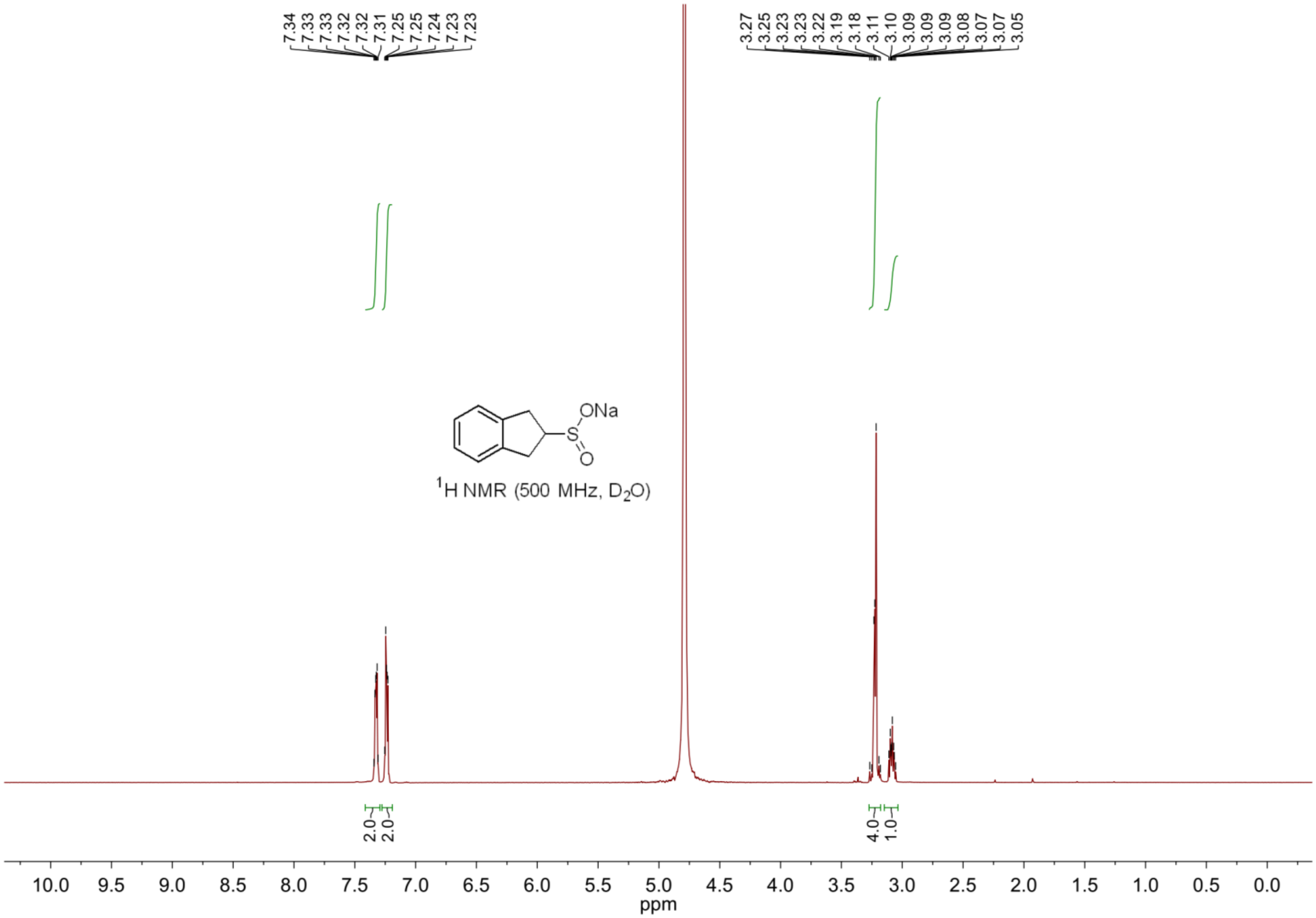

Sodium 2,3-dihydro-1*H*-indene-2-sulfinate (3l)

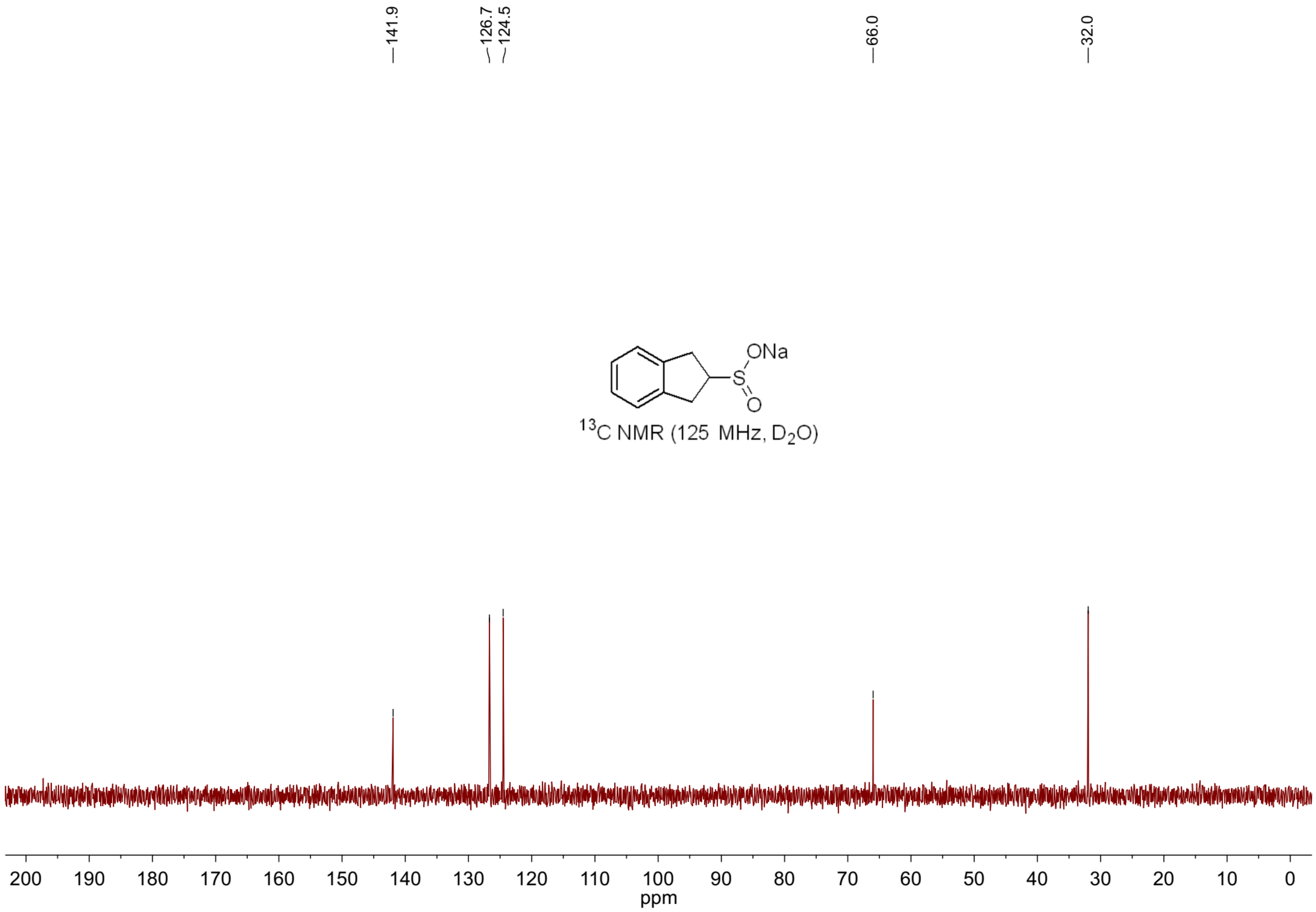

Go back to table of contents

Sodium adamantane-1-sulfinate (3m)

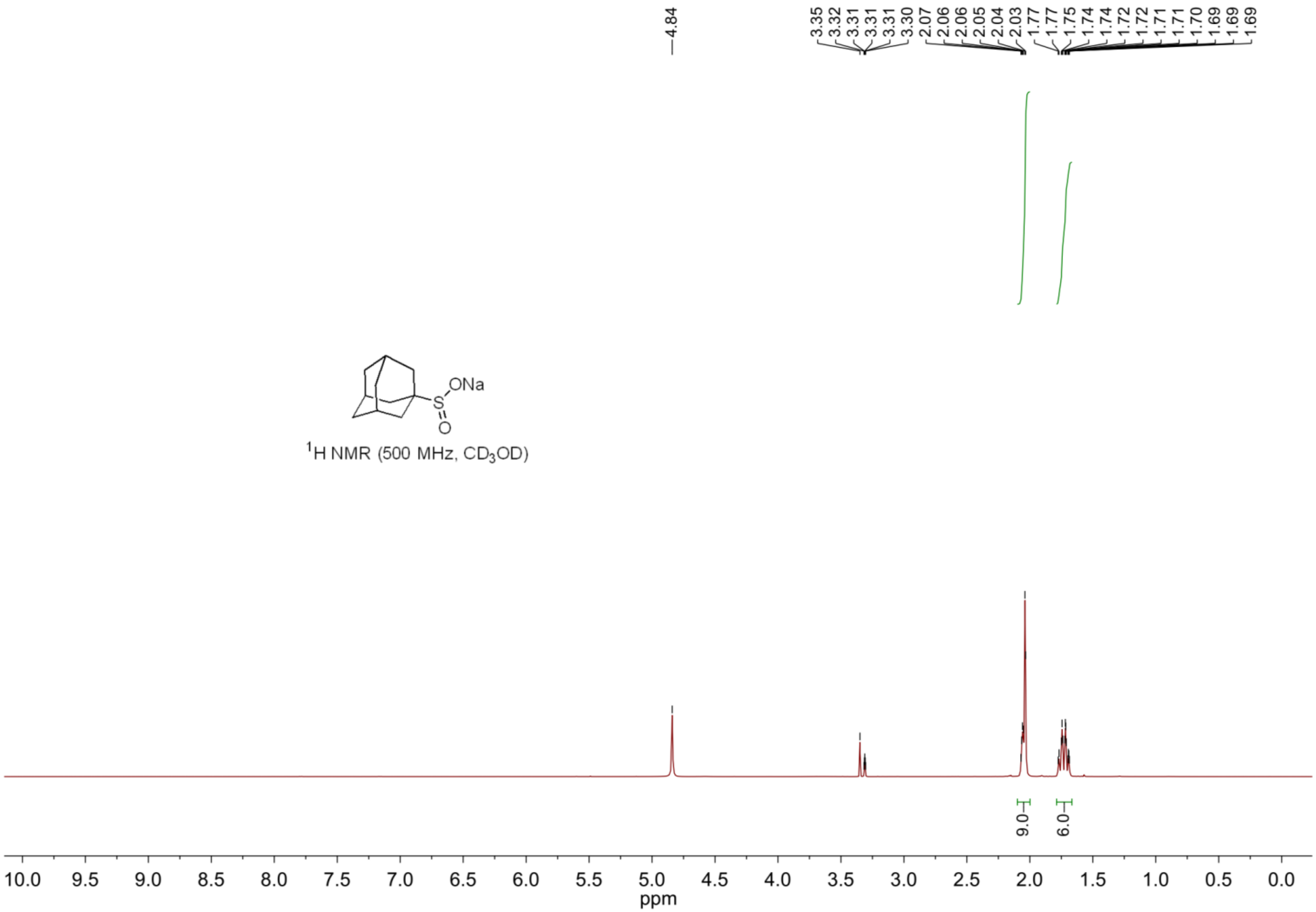

Sodium adamantane-1-sulfinate (3m)

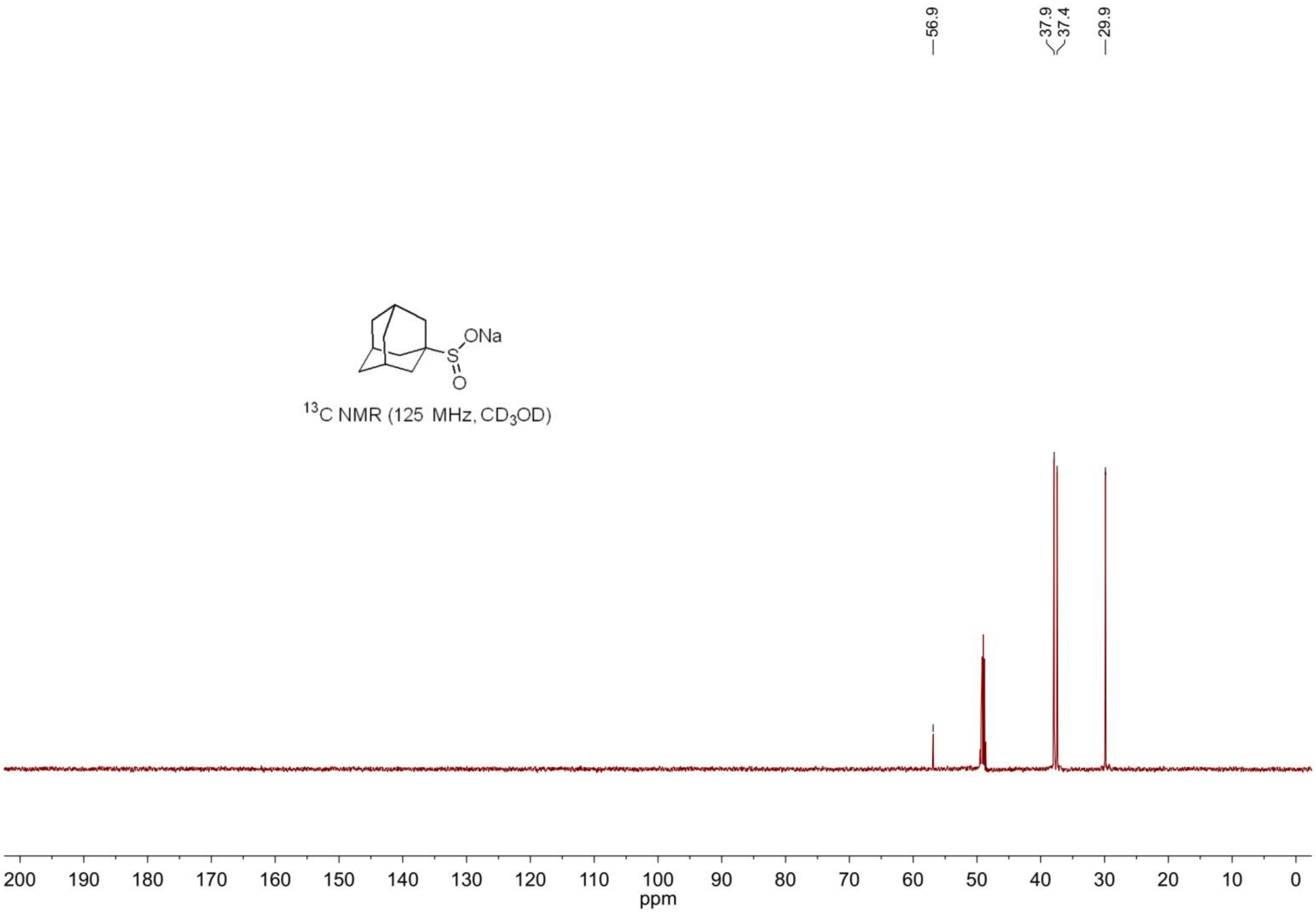

Go back to table of contents

Sodium (3*R*)-3-((3*R*,7*R*,8*R*,9*S*,10*S*,13*R*,14*S*,17*R*)-3,7-dihydroxy-10,13-dimethylhexadecahydro-1*H*-cyclopenta[*a*]phenanthren-17-yl)butane-1-sulfinate (3n)

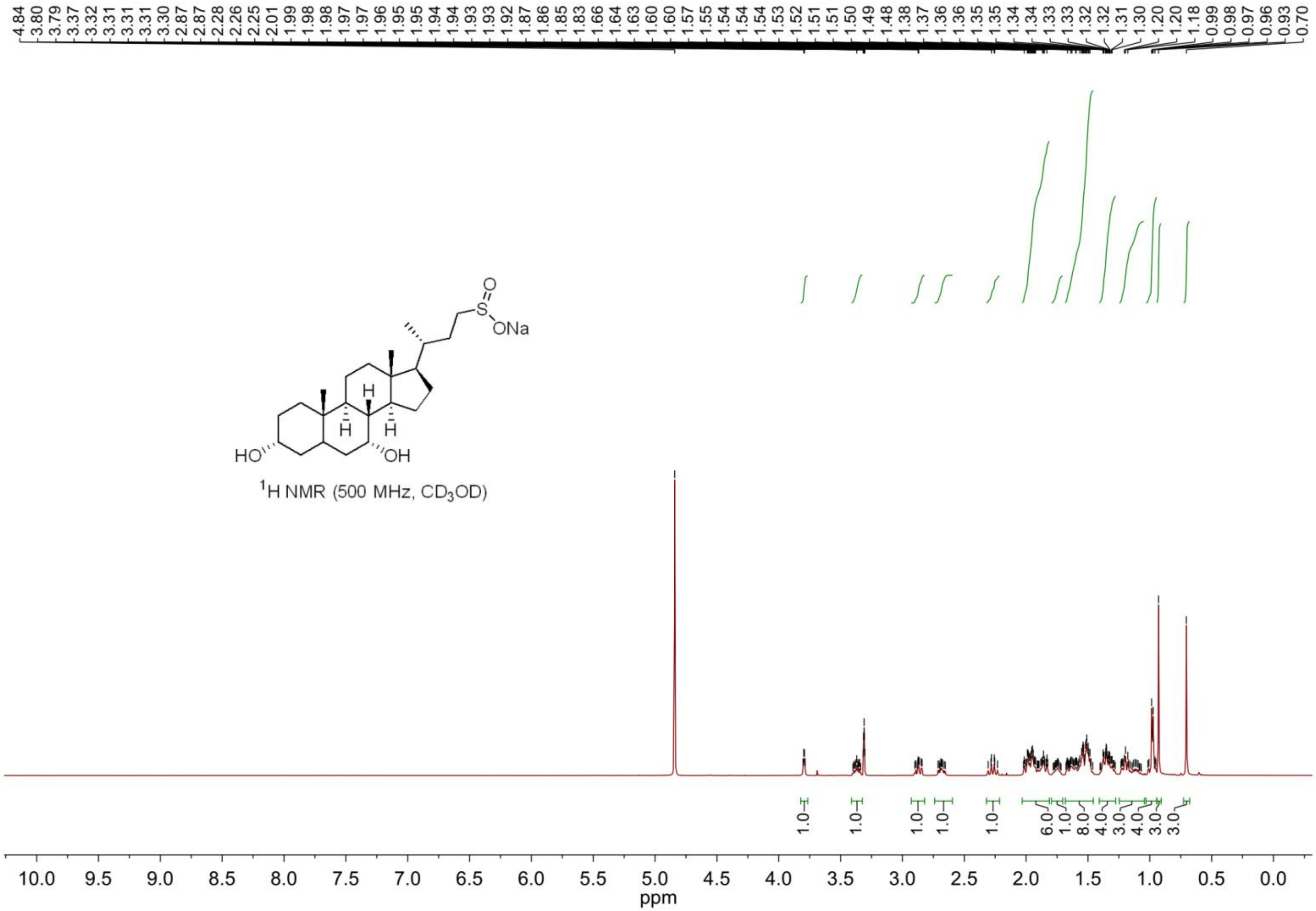

Go back to table of contents

Sodium (3*R*)-3-((3*R*,7*R*,8*R*,9*S*,10*S*,13*R*,14*S*,17*R*)-3,7-dihydroxy-10,13-dimethylhexadecahydro-1*H*-cyclopenta[*a*]phenanthren-17-yl)butane-1-sulfinate (3n)

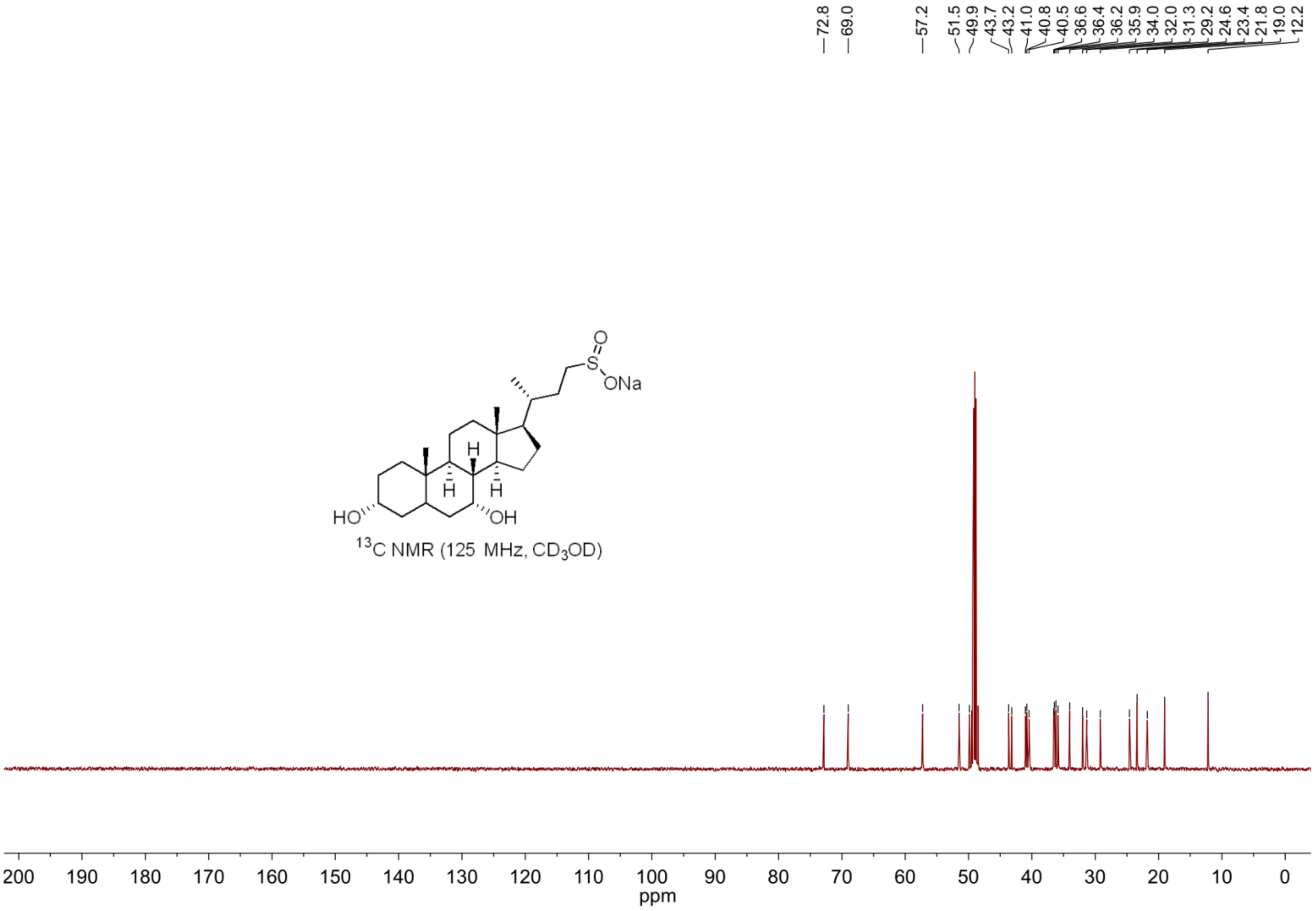

Go back to table of contents

Sodium 1-(6-methoxynaphthalen-2-yl)ethane-1-sulfinate (3o)

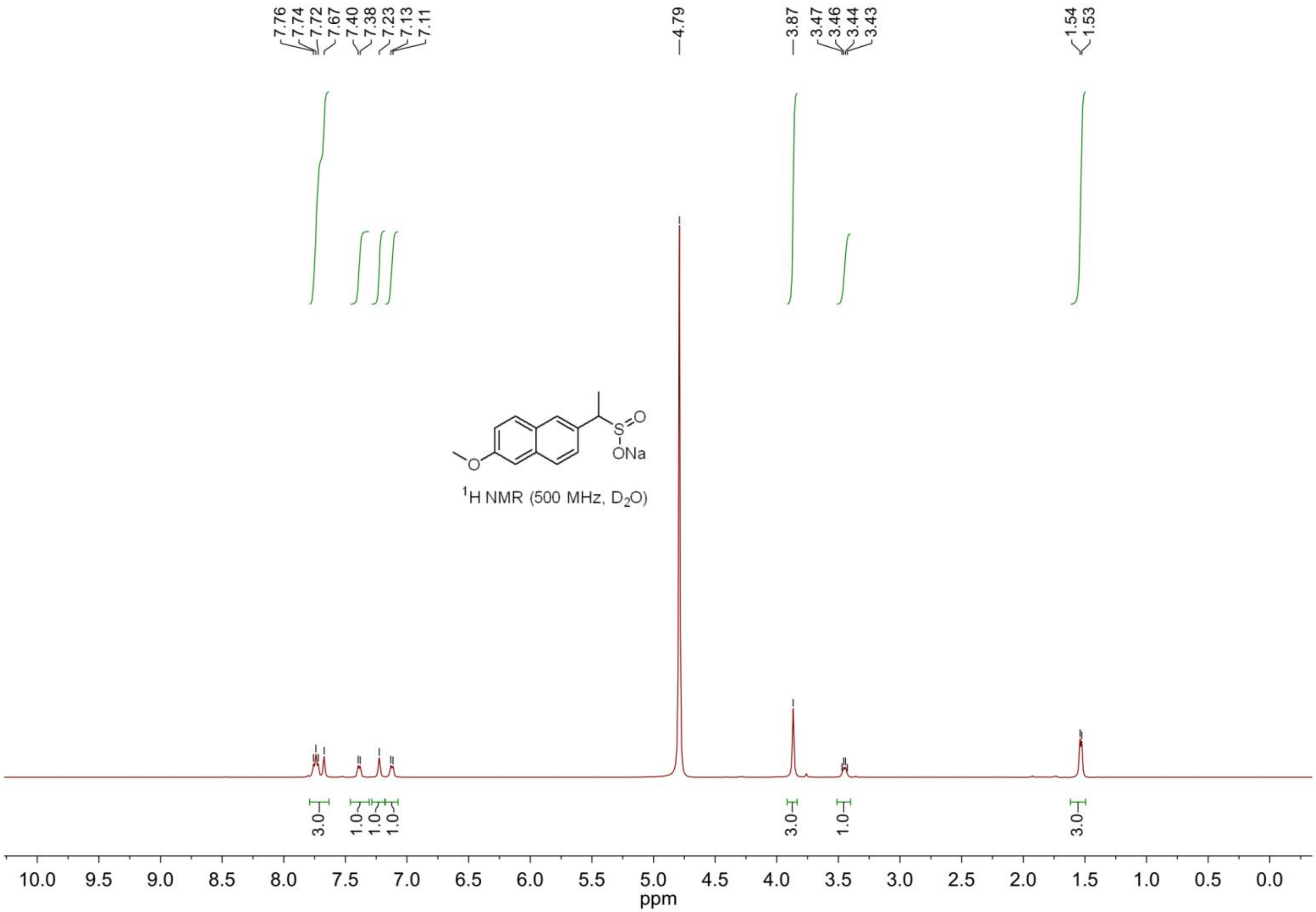

Go back to table of contents

Sodium 1-(6-methoxynaphthalen-2-yl)ethane-1-sulfinate (3o)

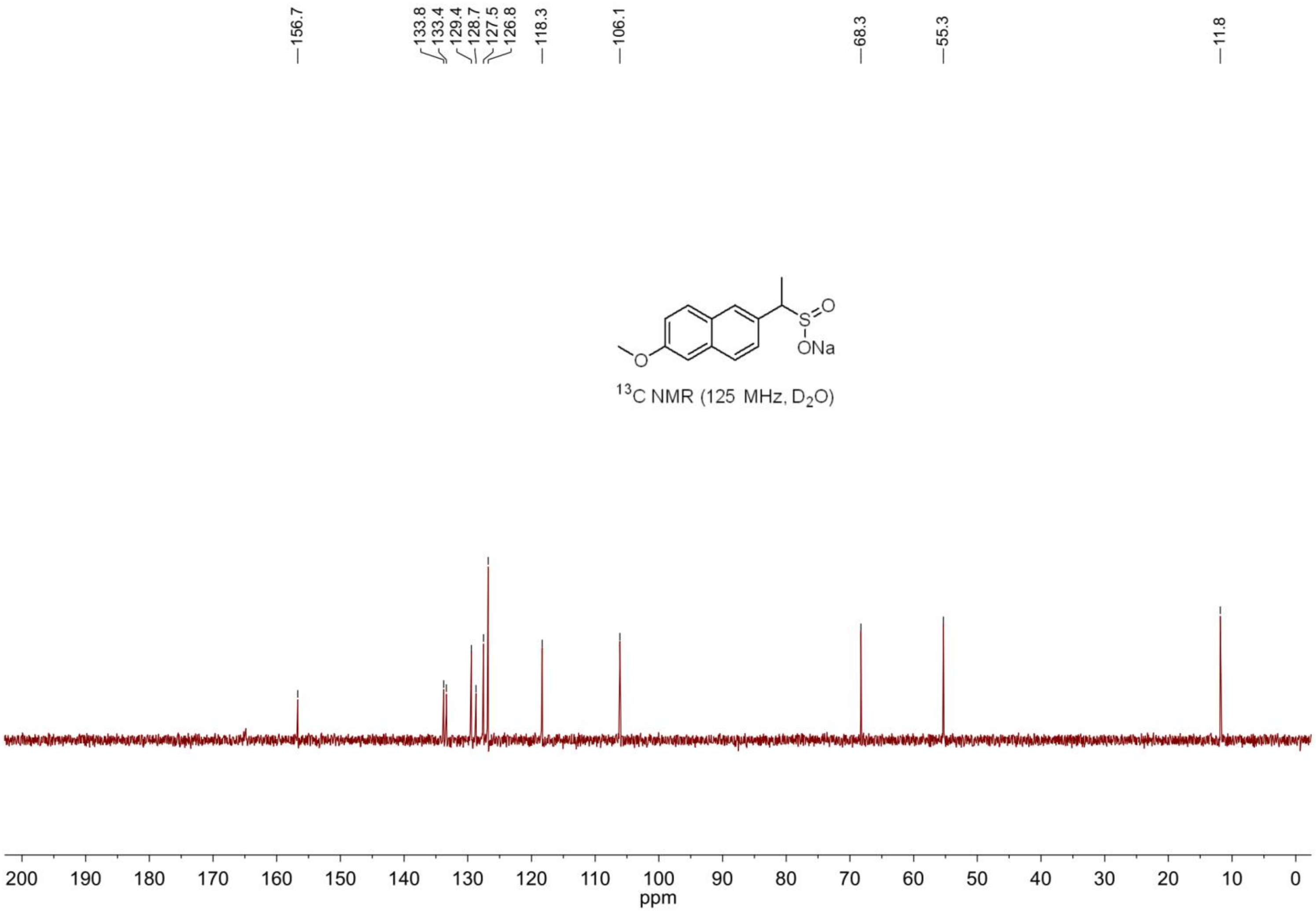

Go back to table of contents

Sodium heptane-4-sulfinate (3p)

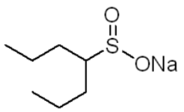

^{1}H NMR (500 MHz, CDCl$_3$)

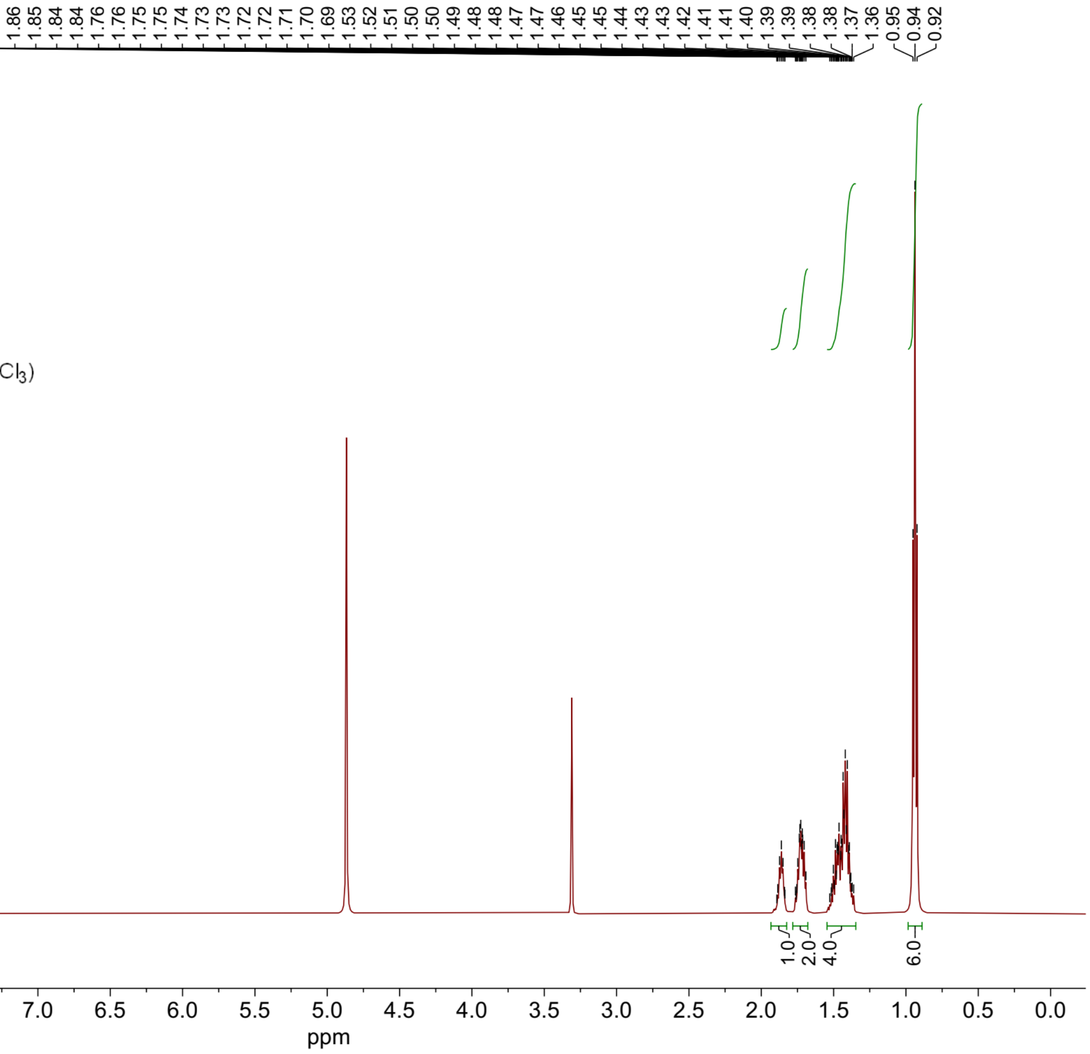

Go back to table of contents

Sodium heptane-4-sulfinate (3p)

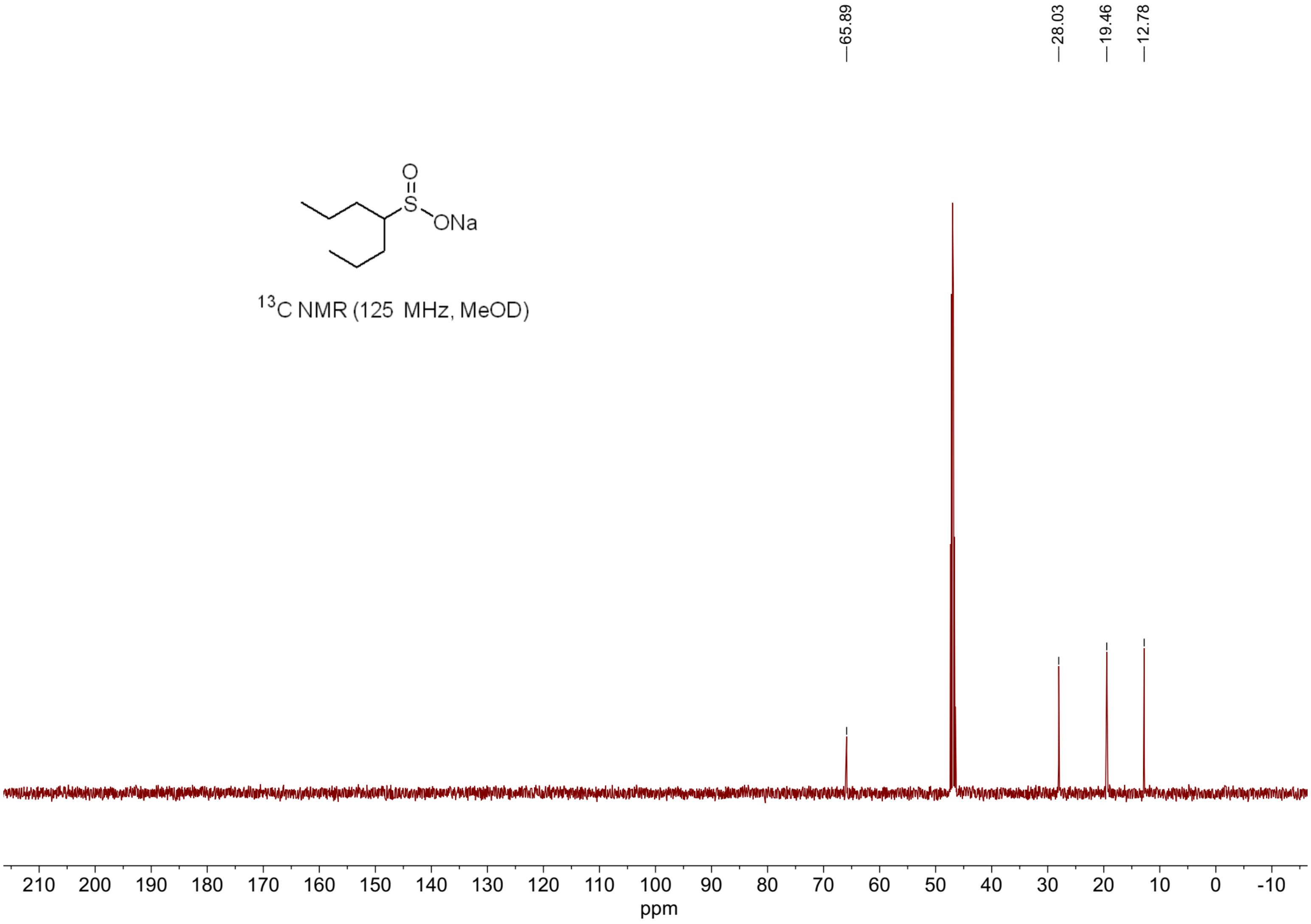

Go back to table of contents

Sodium heptane-4-sulfonate (3q)

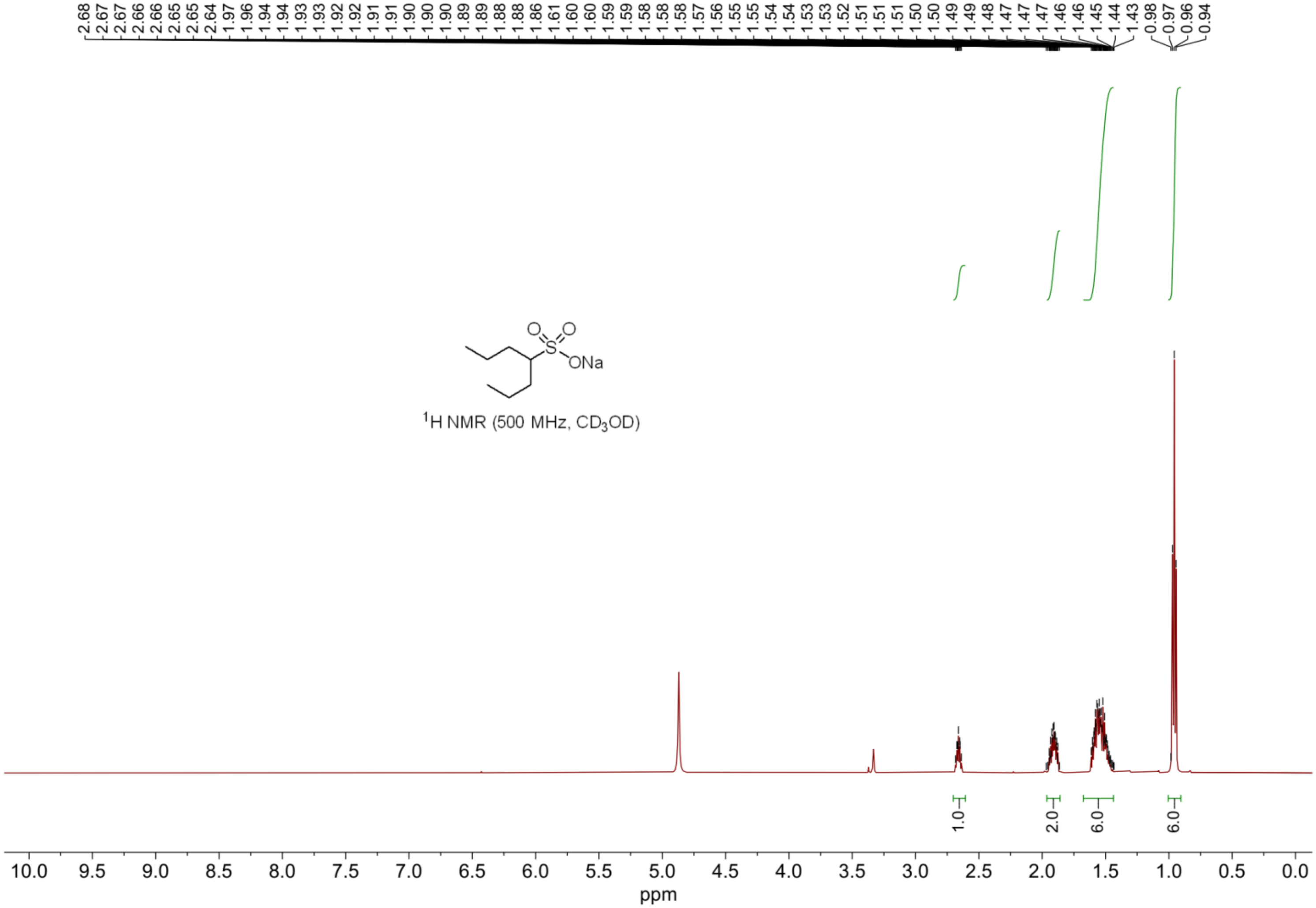

Go back to table of contents

Sodium heptane-4-sulfonate (3q)

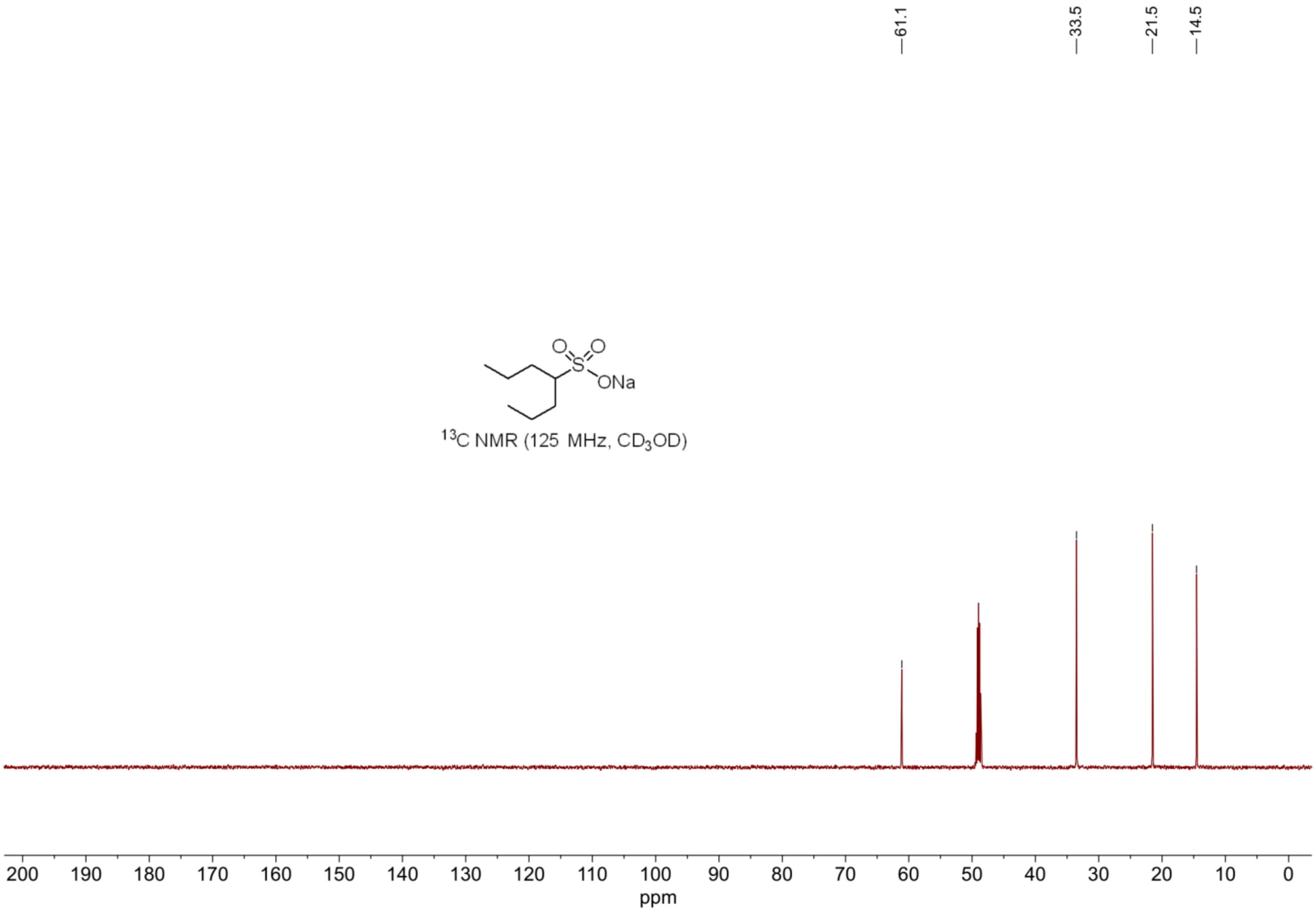

Go back to table of contents

Sodium 5-(2,5-dimethylphenoxy)-2-methylpentane-2-sulfinate (3r)

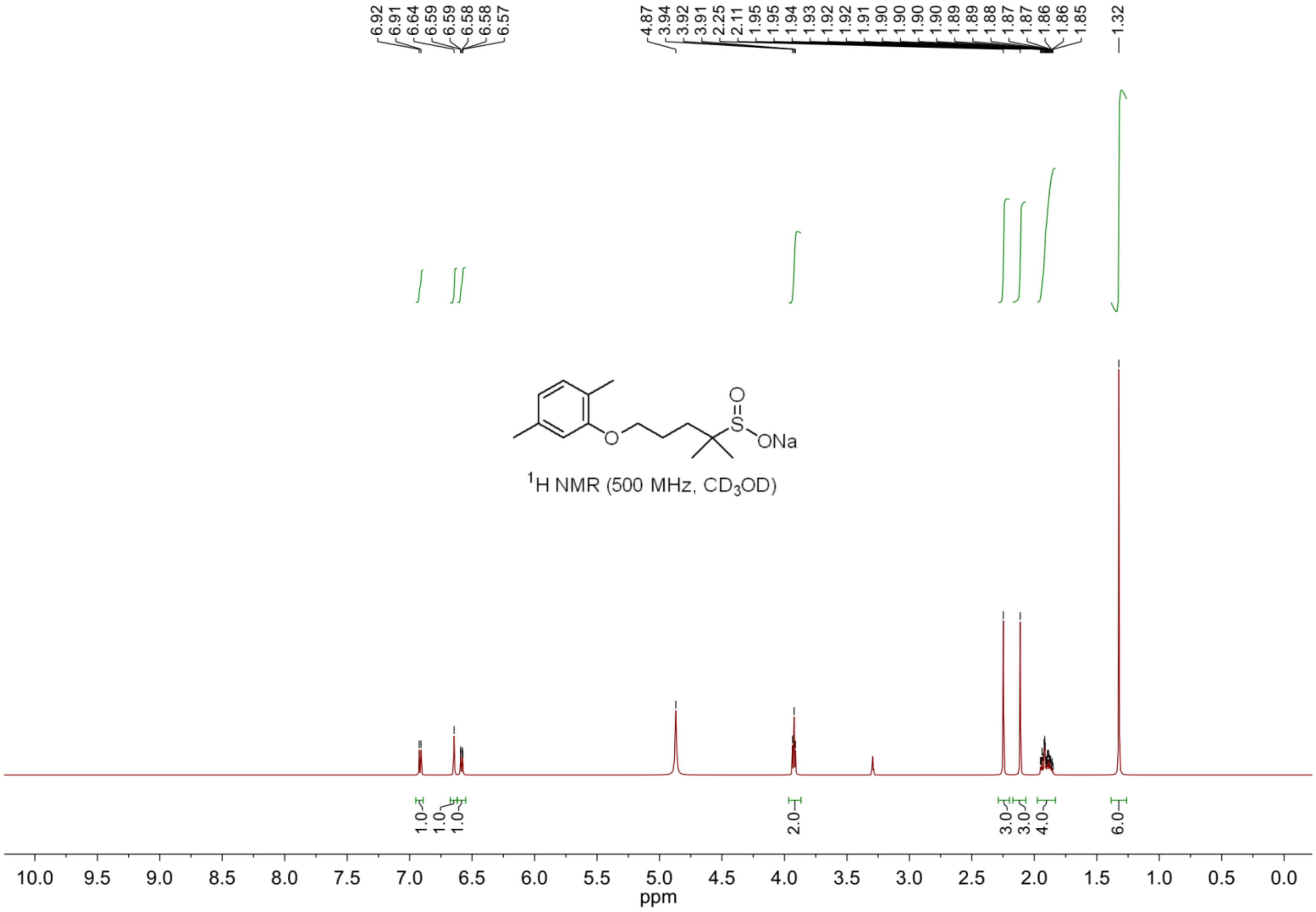

Go back to table of contents

Sodium 5-(2,5-dimethylphenoxy)-2-methylpentane-2-sulfinate (3r)

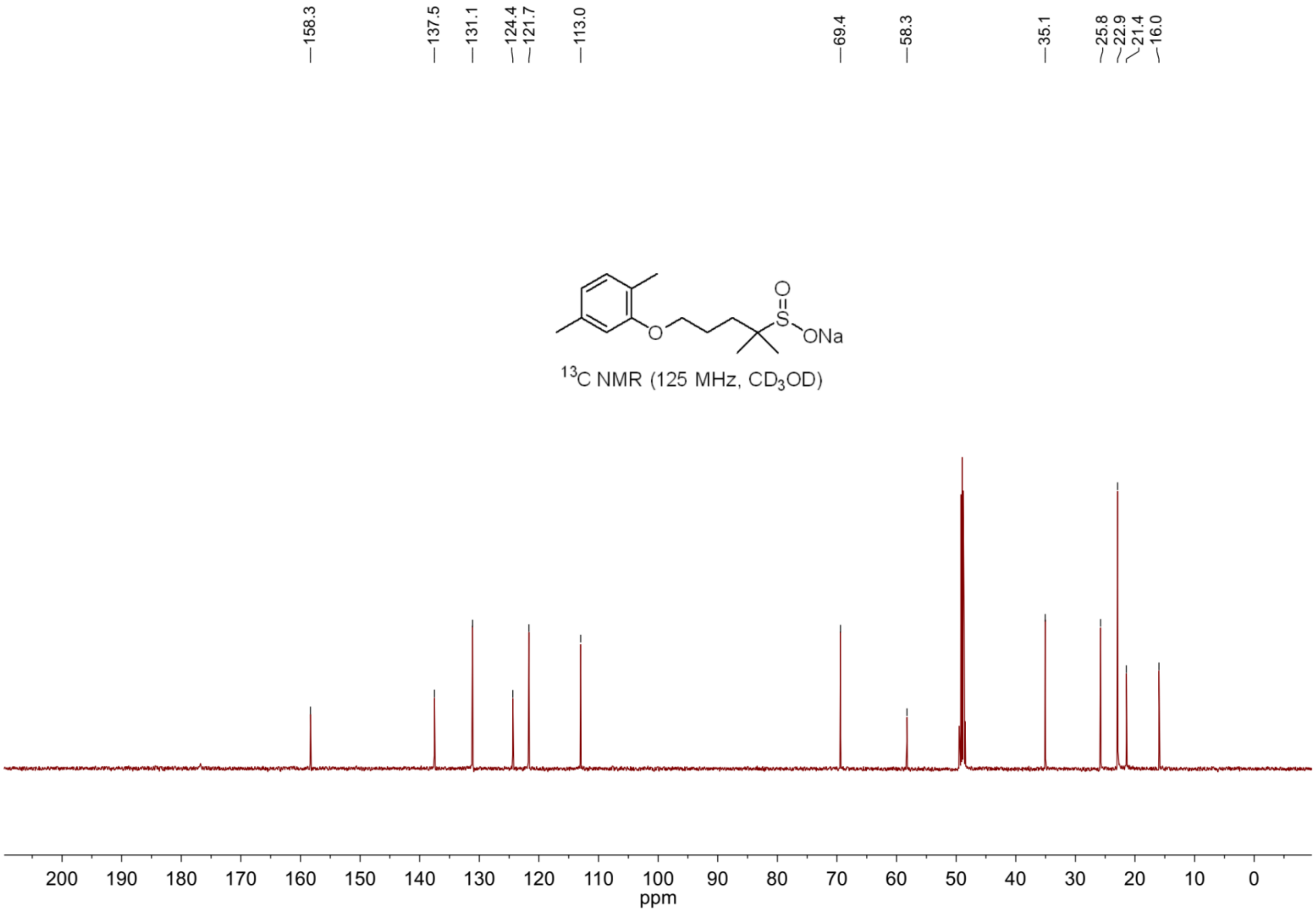

Go back to table of contents

2-(4-Fluorophenyl)ethane-1-sulfonyl chloride (4a)

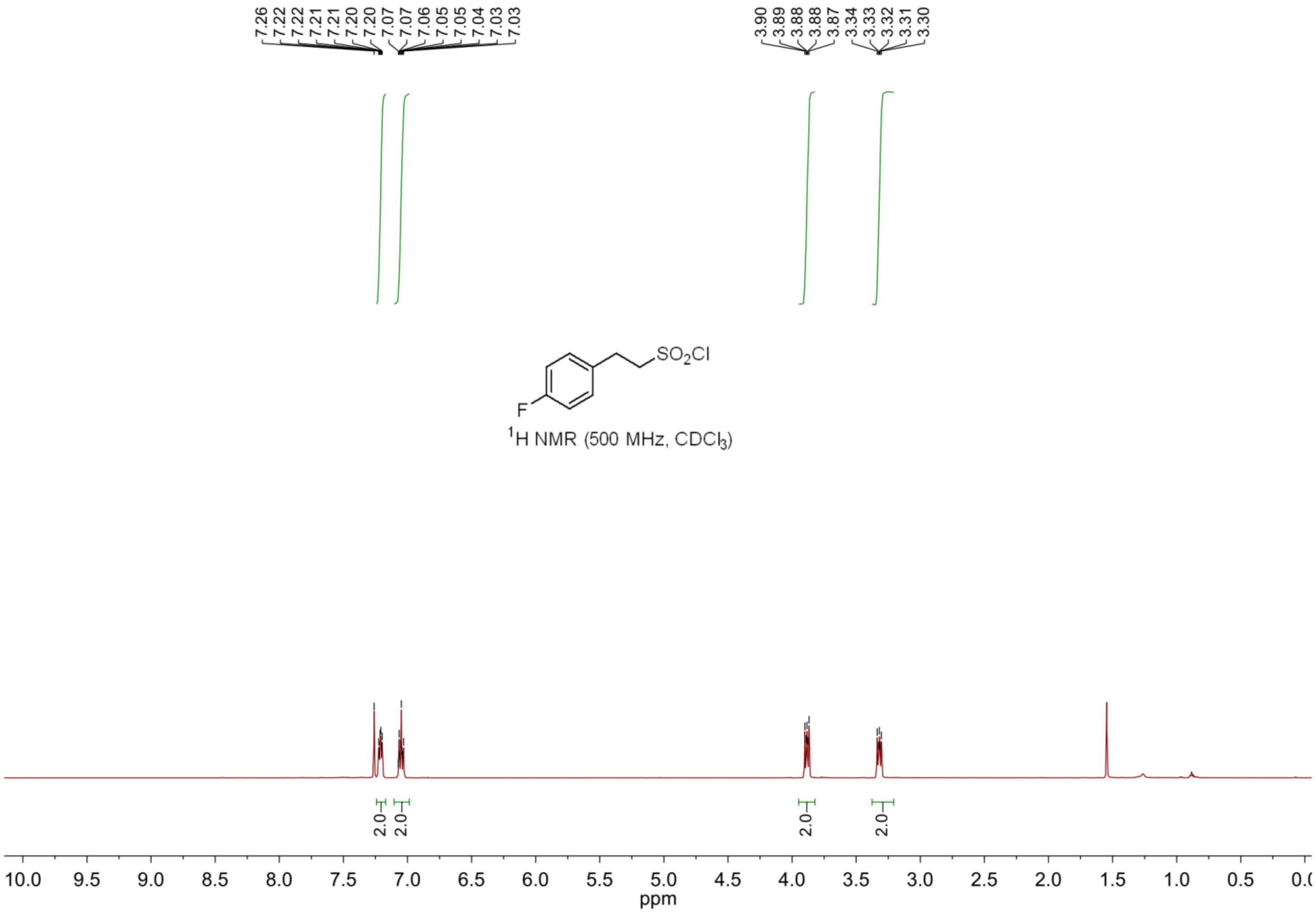

Go back to table of contents

2-(4-Fluorophenyl)ethane-1-sulfonyl chloride (4a)

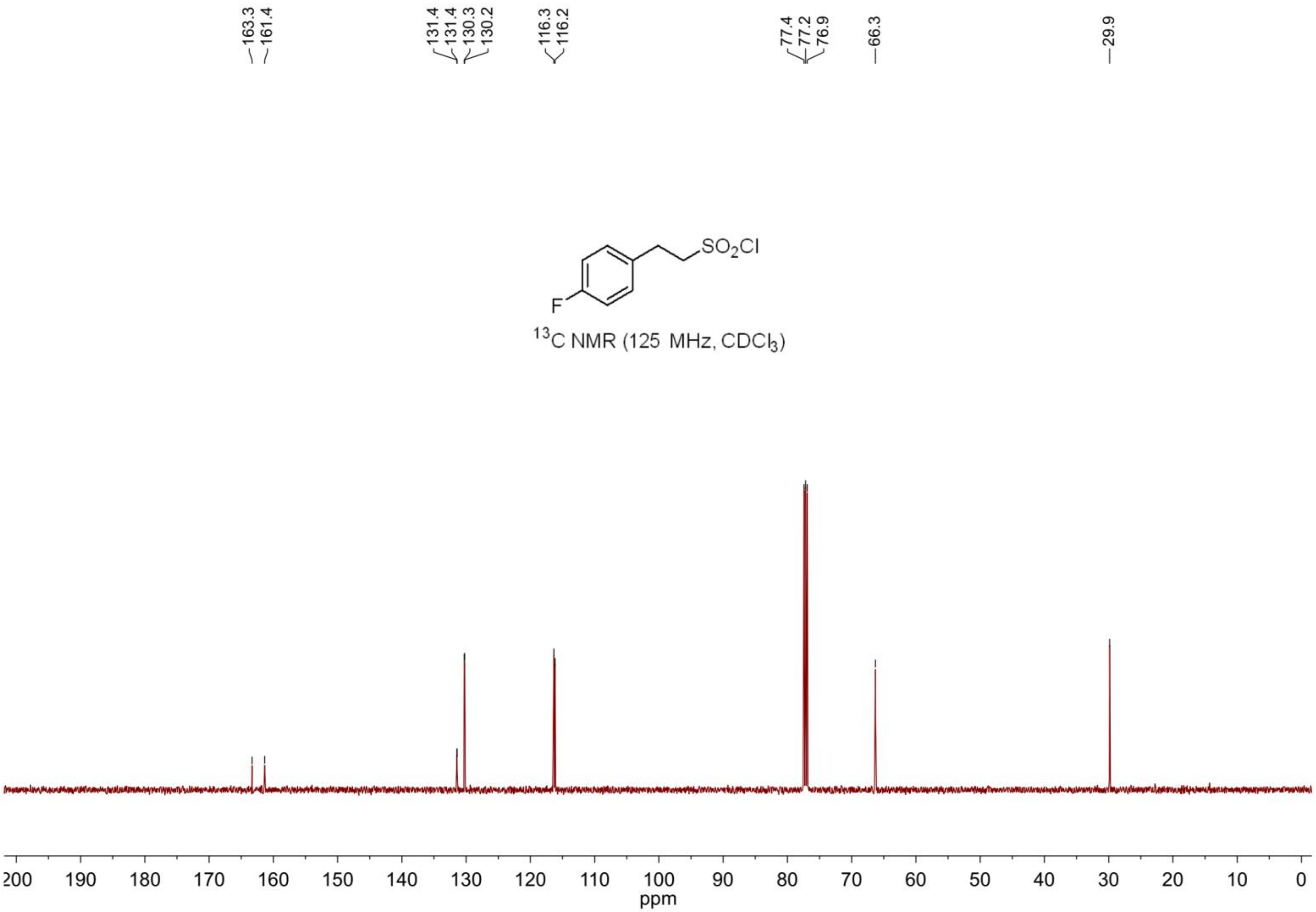

Go back to table of contents

4-Oxo-4-phenylbutane-1-sulfonyl chloride (4b)

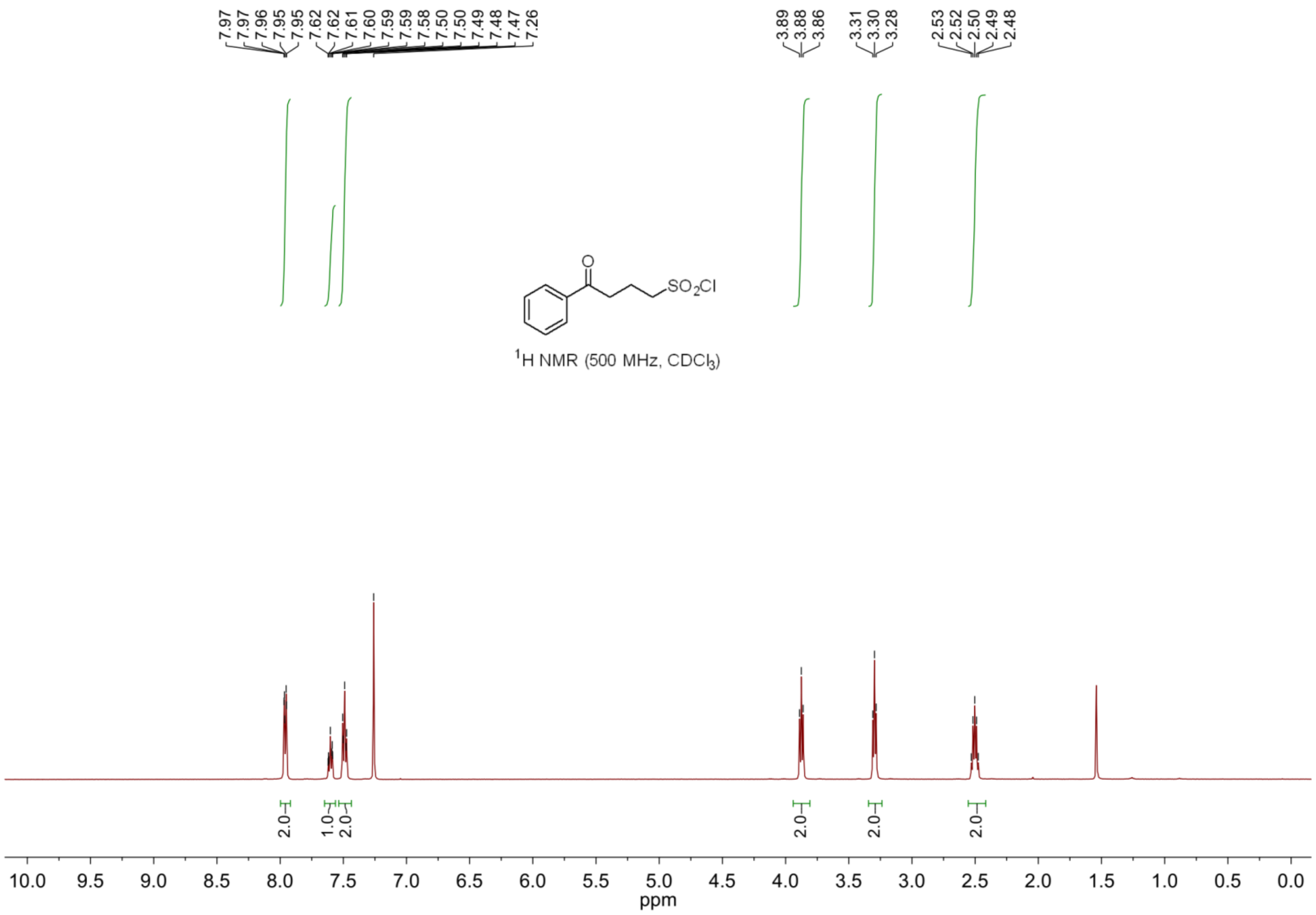

Go back to table of contents

4-Oxo-4-phenylbutane-1-sulfonyl chloride (4b)

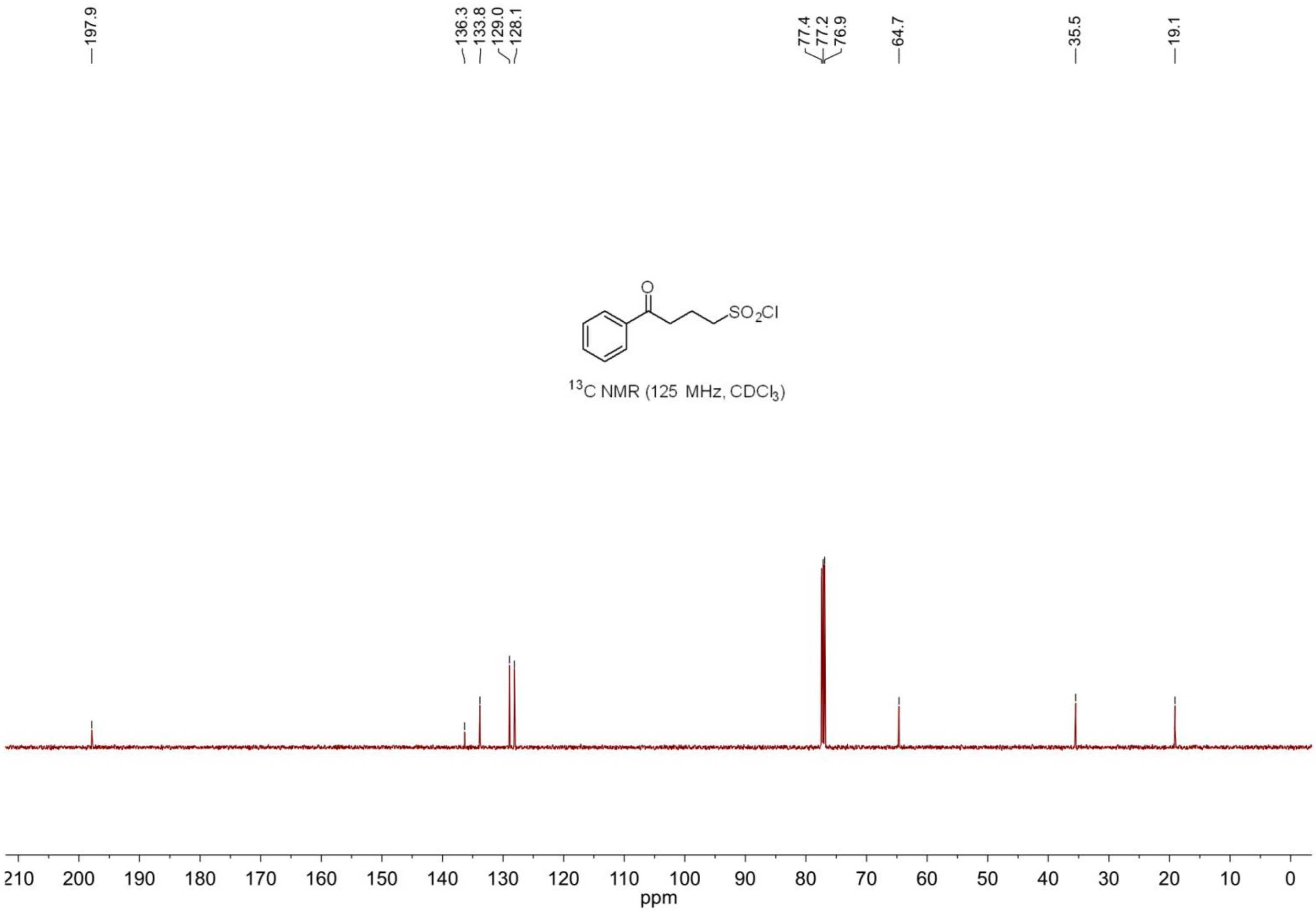

Go back to table of contents

tert-Butyl (4-(chlorosulfonyl)butyl)carbamate (4c)

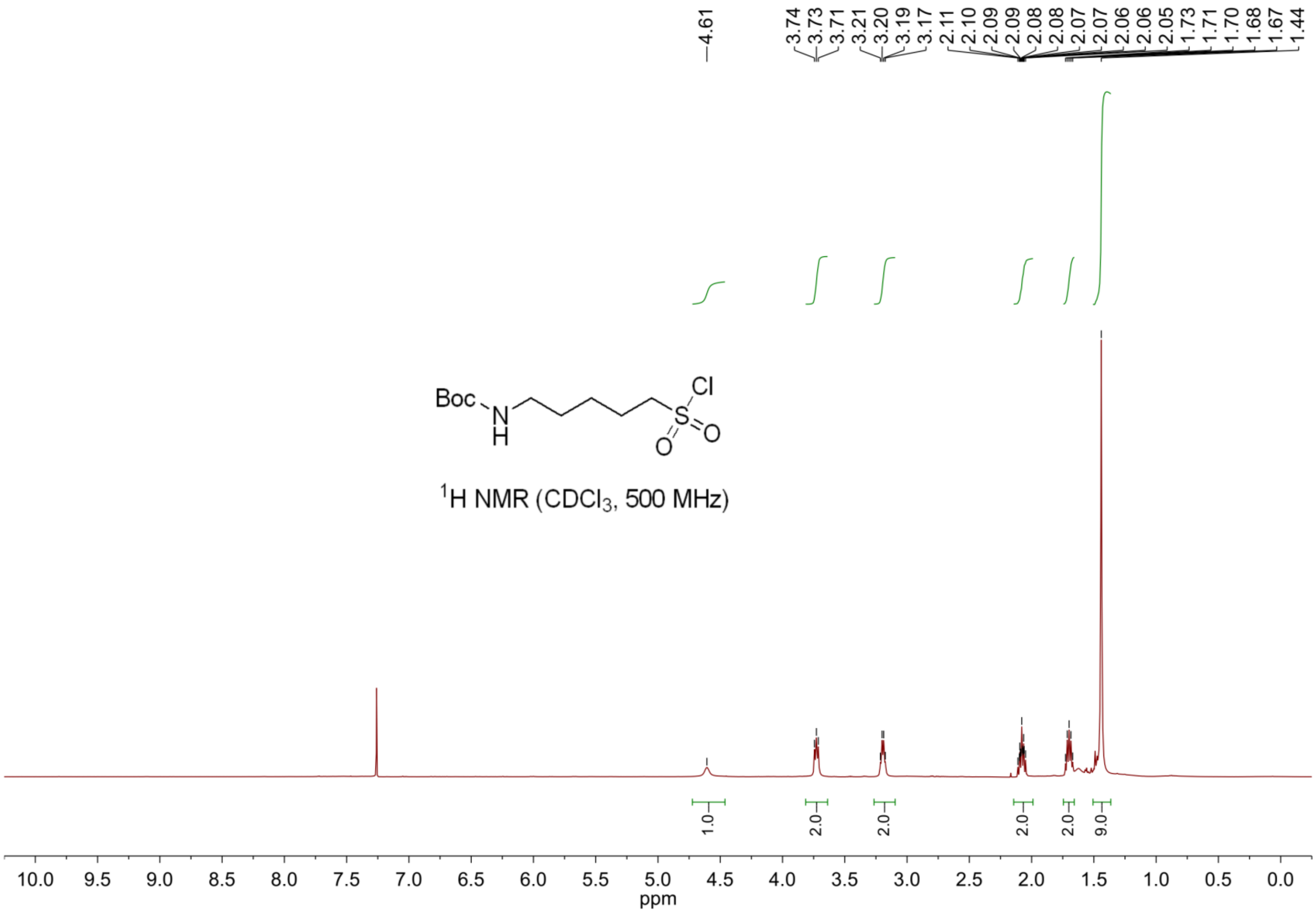

tert-Butyl (4-(chlorosulfonyl)butyl)carbamate (4c)

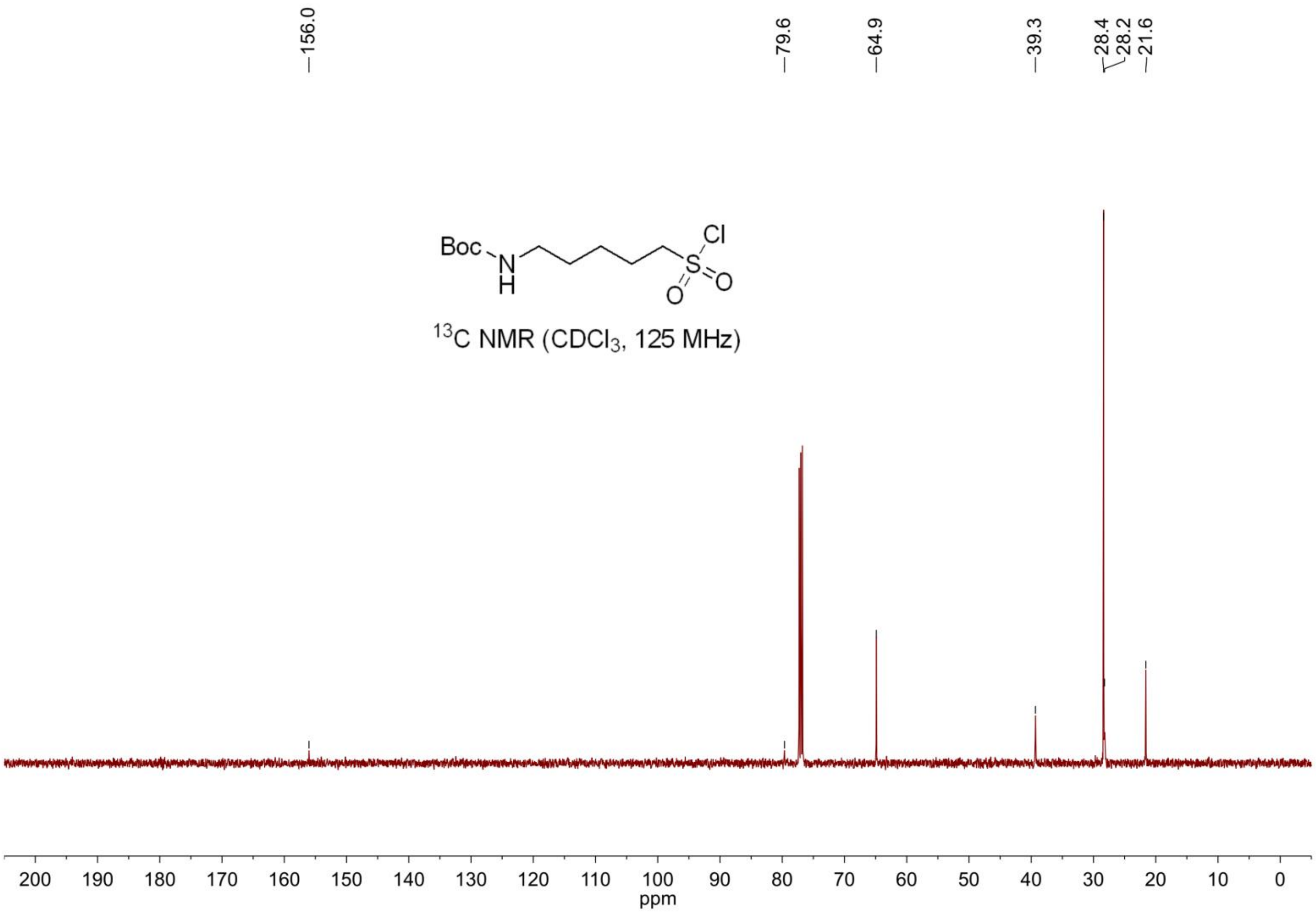

Go back to table of contents

***tert*-Butyl (5-(chlorosulfonyl)pentyl)carbamate (4d)**

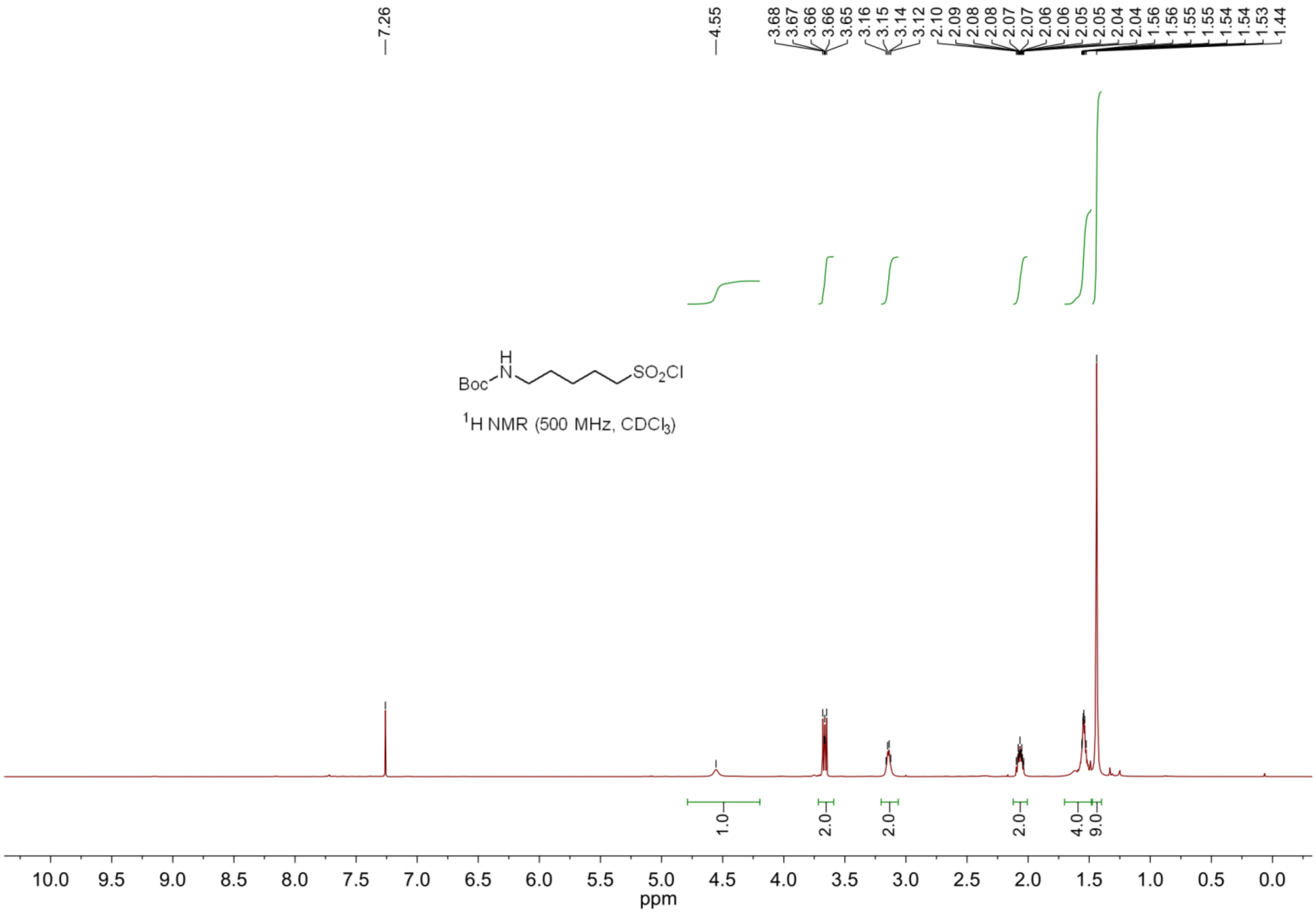

Go back to table of contents

tert-Butyl (5-(chlorosulfonyl)pentyl)carbamate (4d)

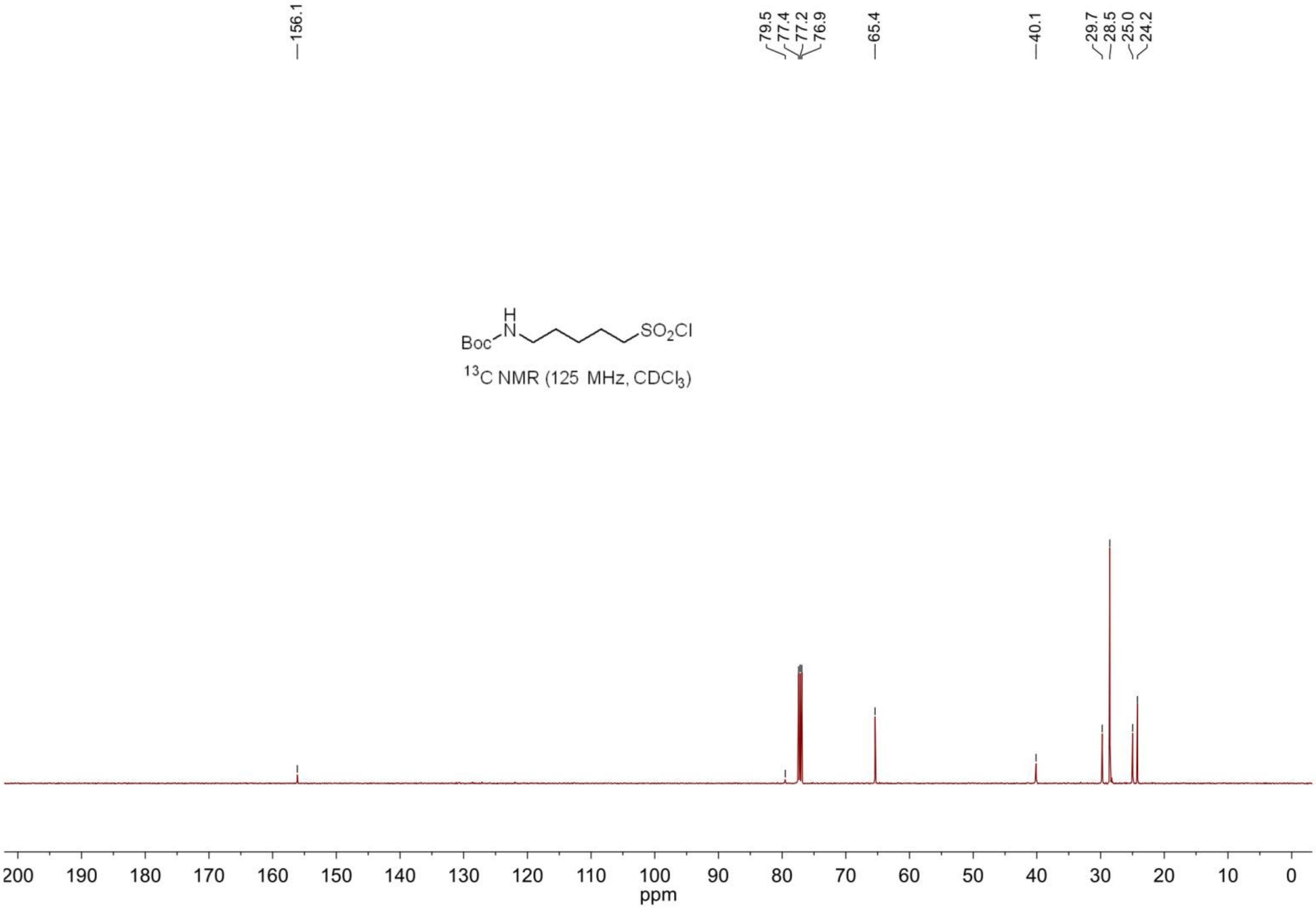

Go back to table of contents

Pentadecane-1-sulfonyl chloride (4e)

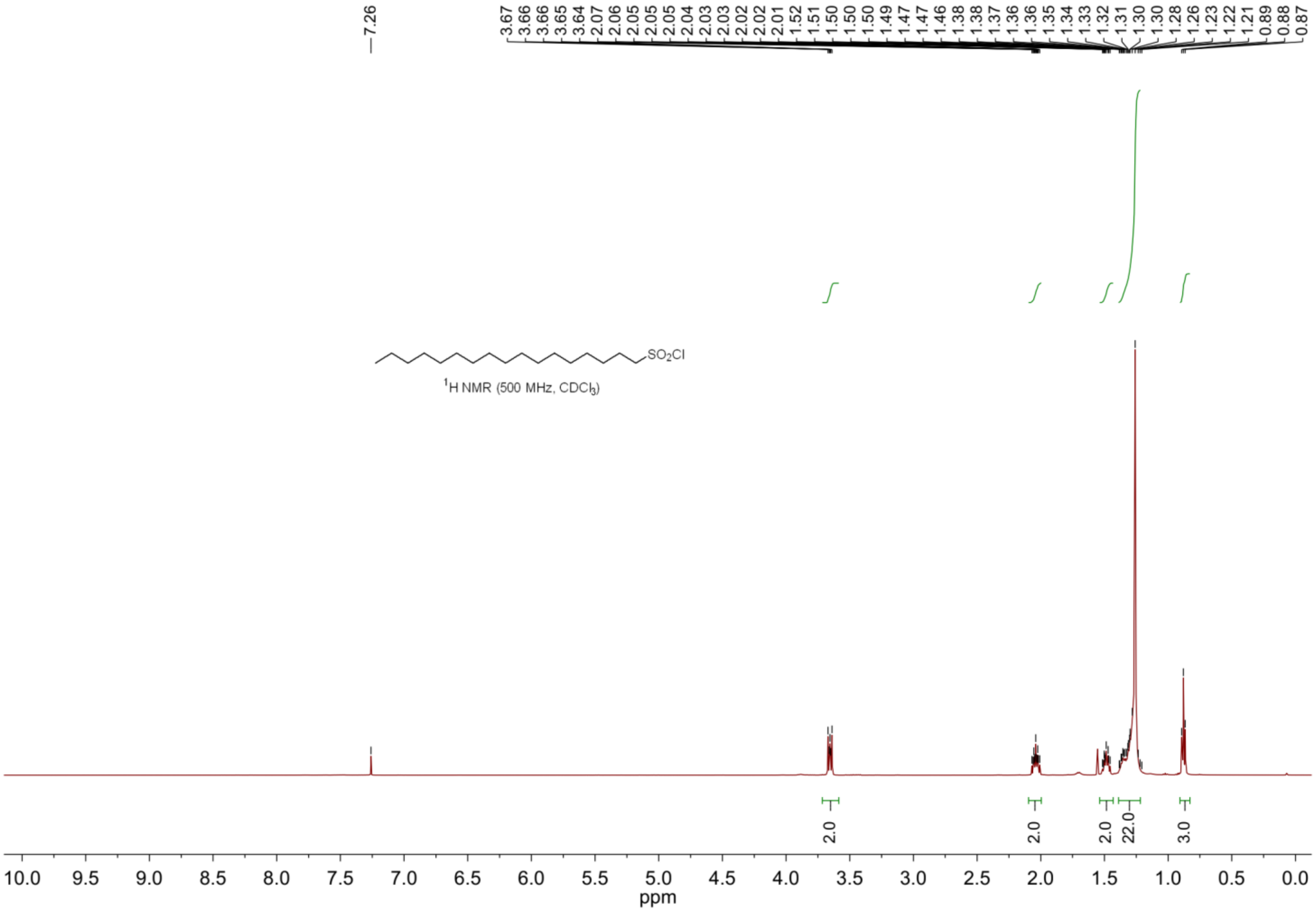

Pentadecane-1-sulfonyl chloride (4e)

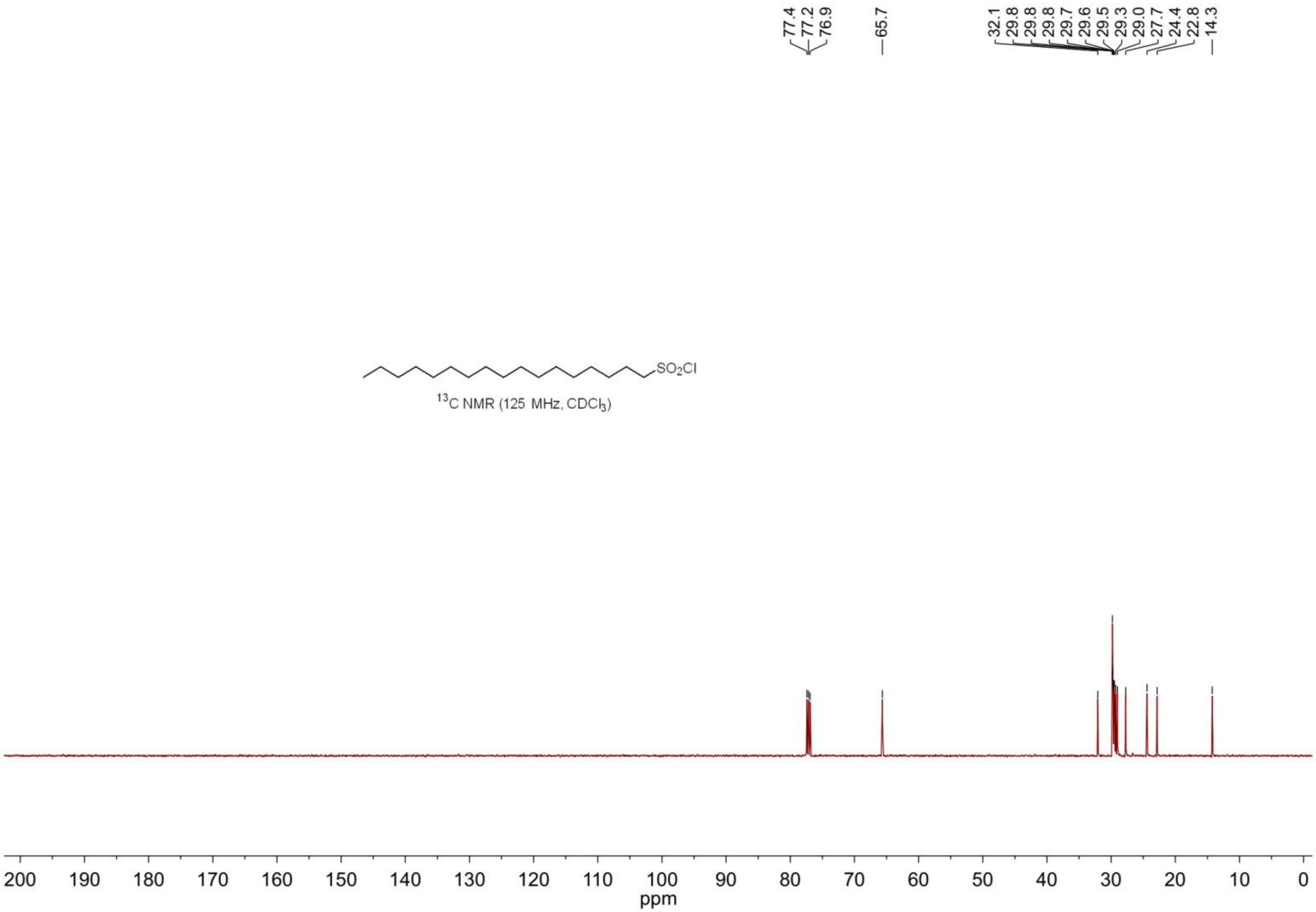

Go back to table of contents

Heptadecane-1-sulfonyl chloride (4f)

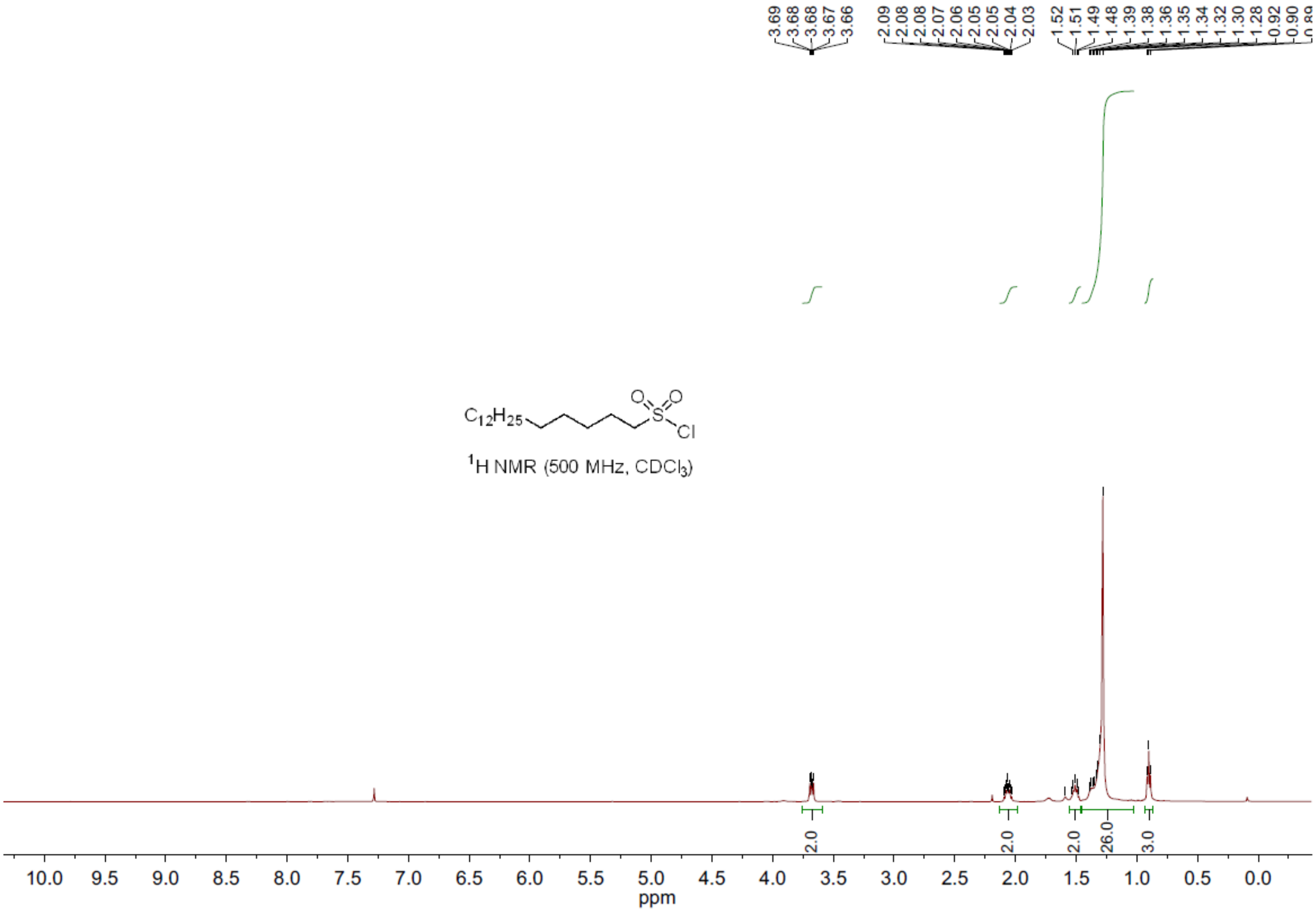

Go back to table of contents

Heptadecane-1-sulfonyl chloride (4f)

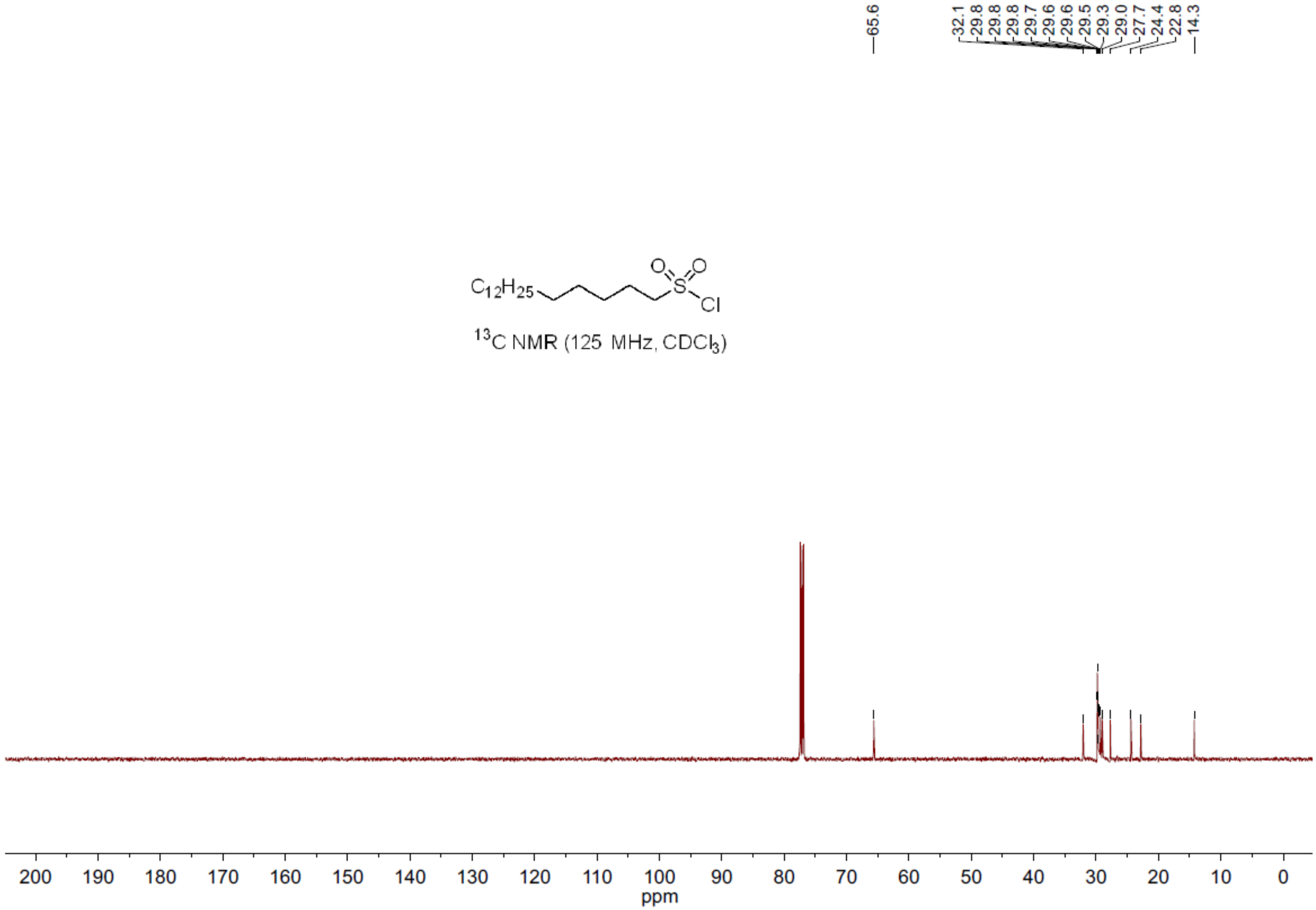

Go back to table of contents

Heptane-4-sulfonyl chloride (4g)

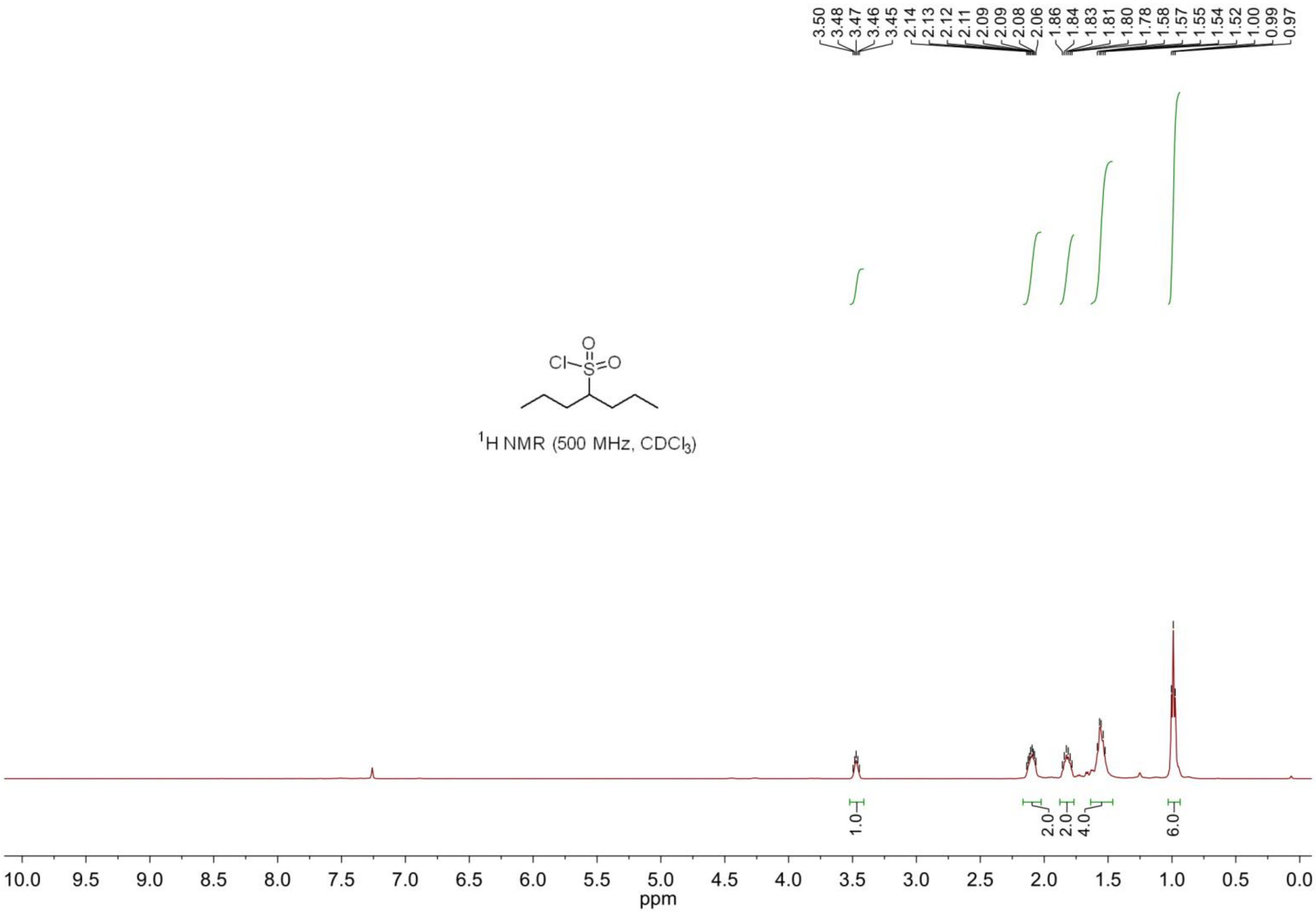

Go back to table of contents

Heptane-4-sulfonyl chloride (4g)

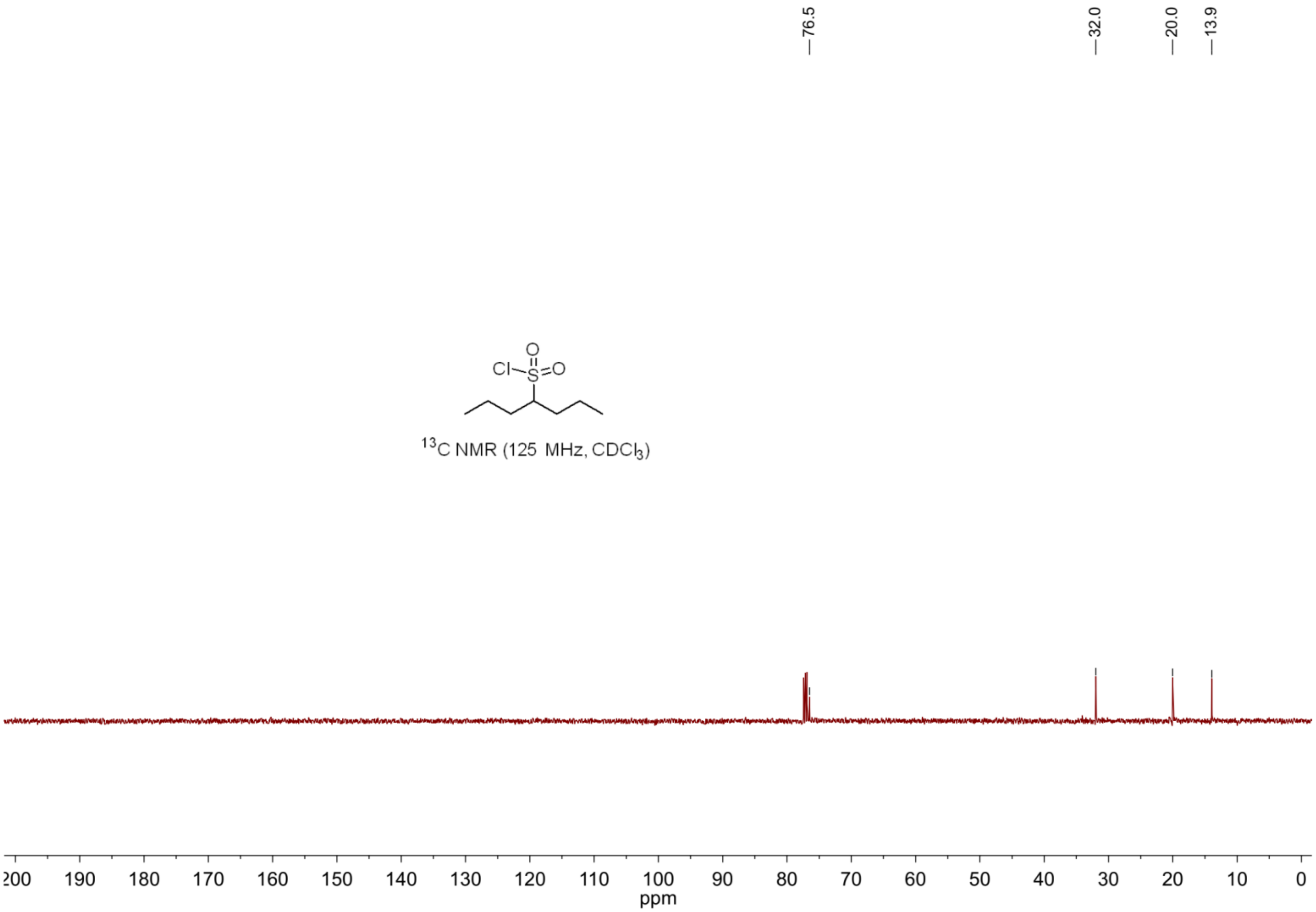

Go back to table of contents

Tetrahydro-2*H*-pyran-4-sulfonyl chloride (4h)

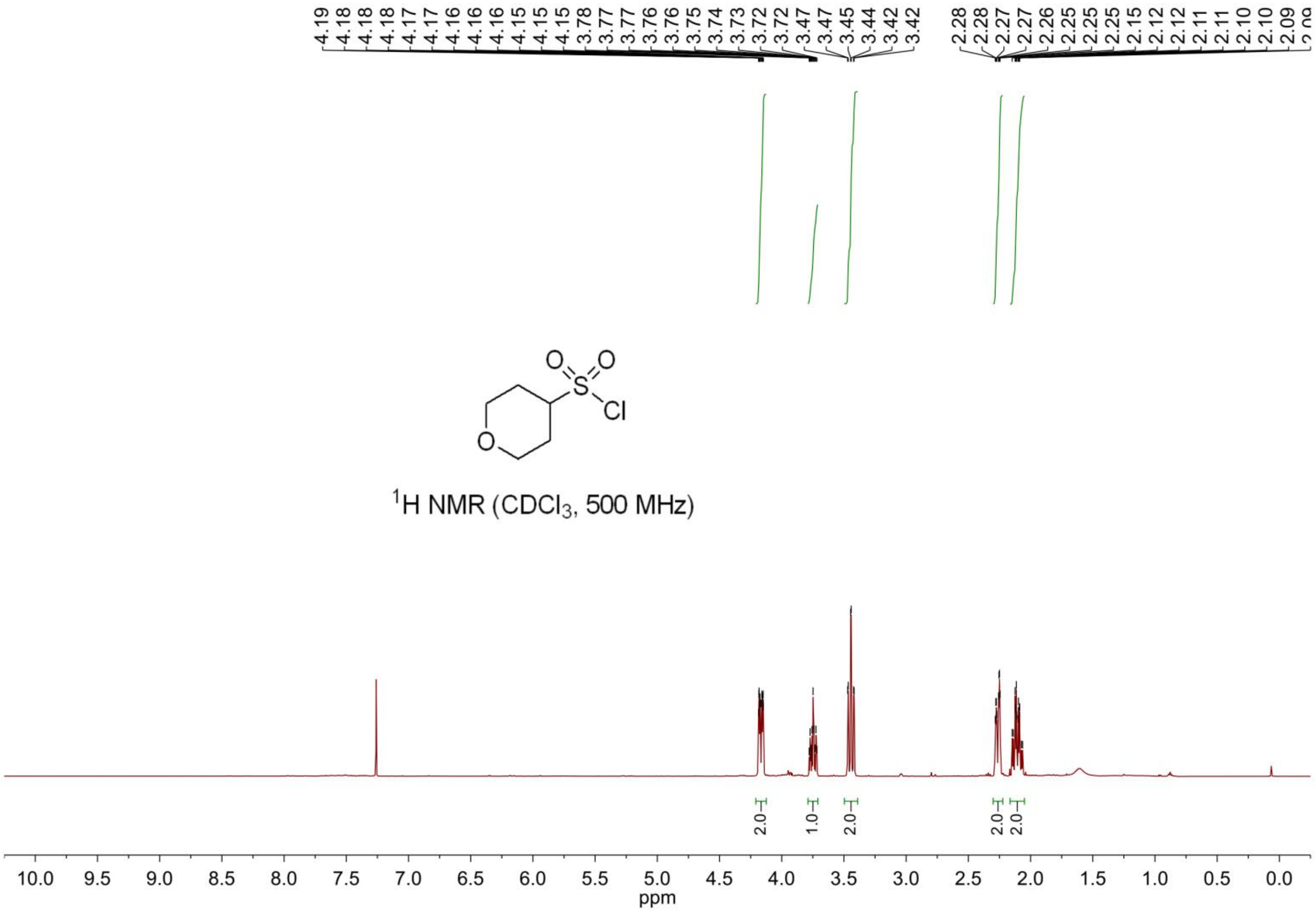

Go back to table of contents

Tetrahydro-2*H*-pyran-4-sulfonyl chloride (4h)

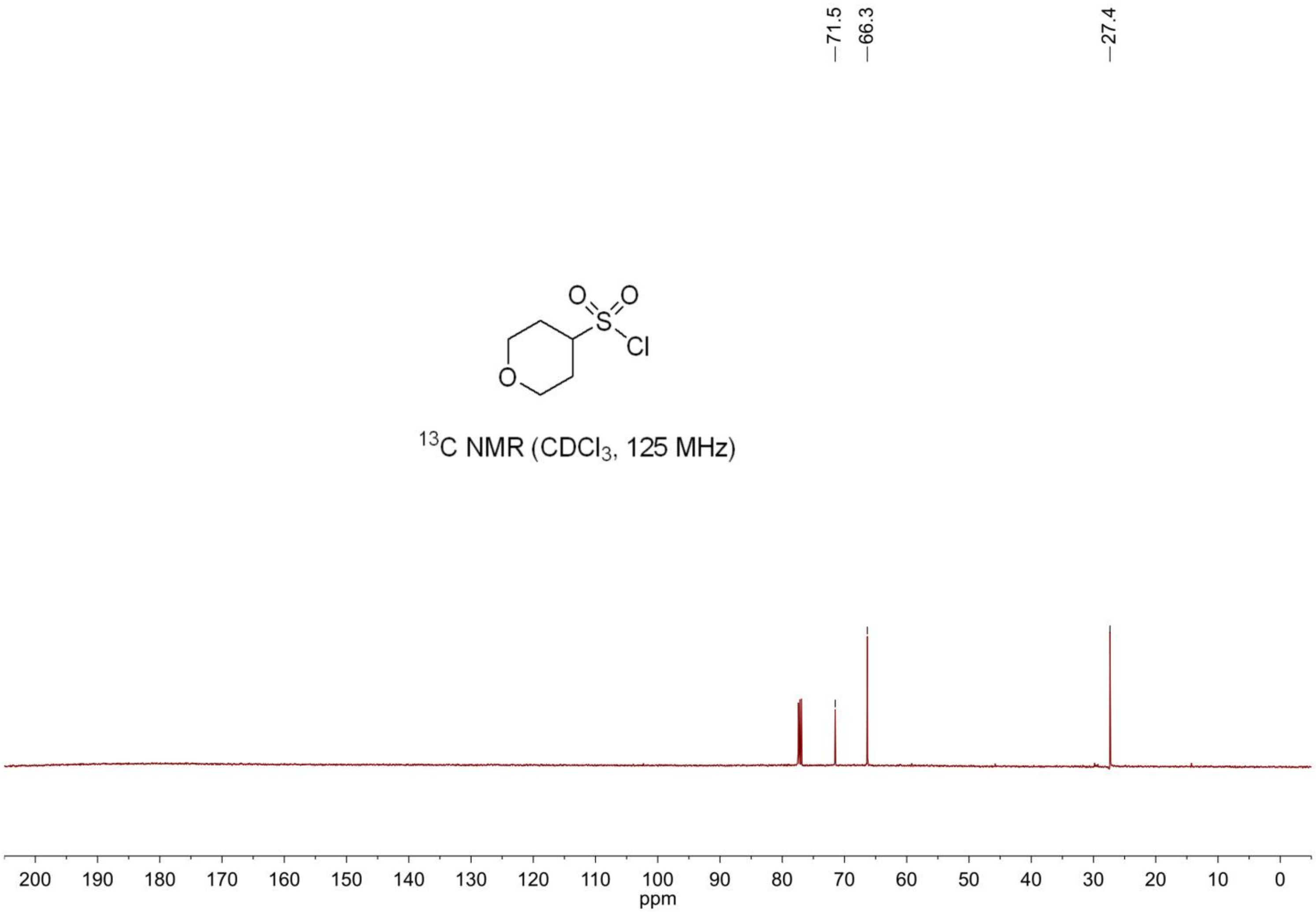

Go back to table of contents

2,3-Dihydro-1*H*-indene-2-sulfonyl chloride (4i)

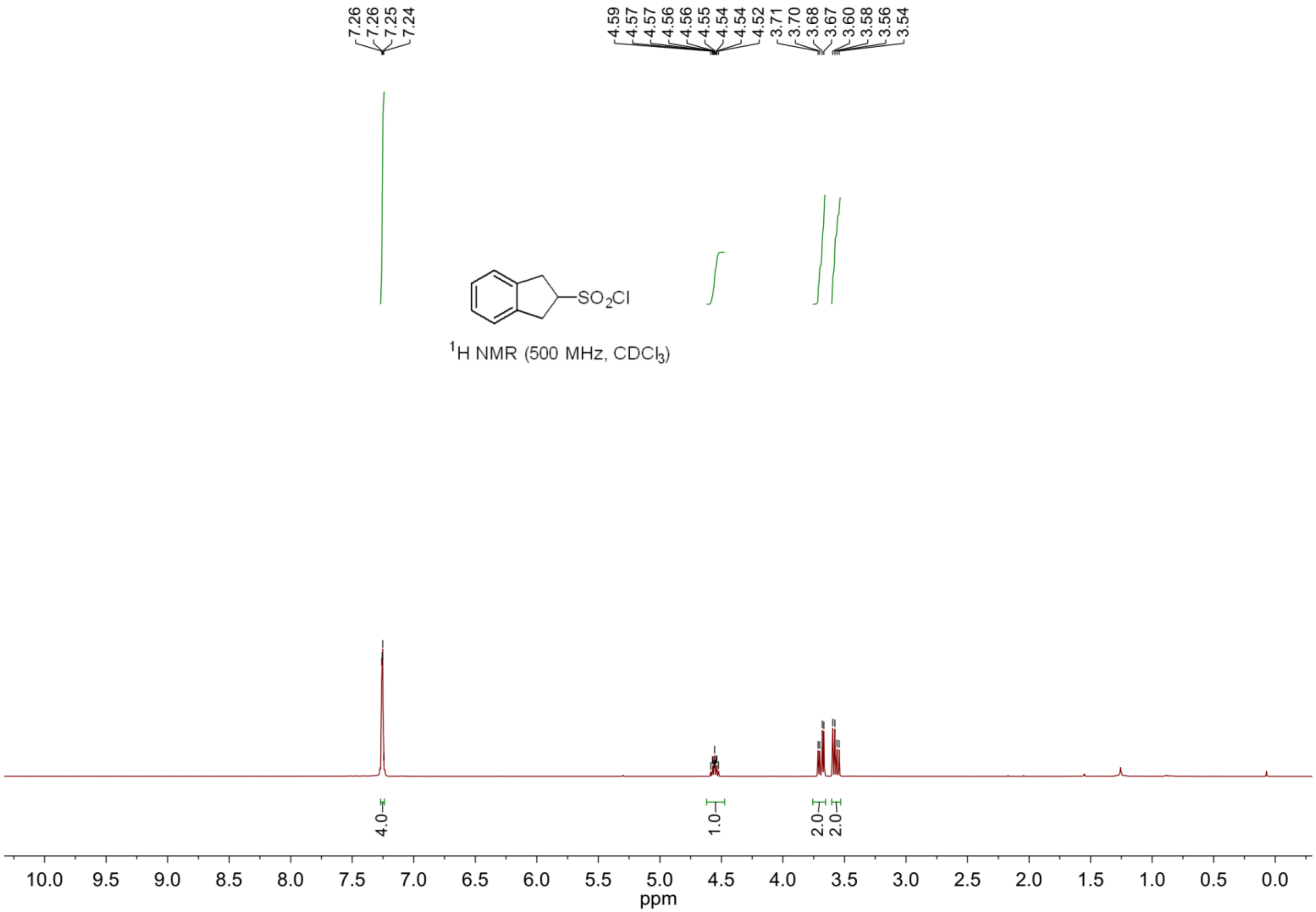

Go back to table of contents

2,3-Dihydro-1*H*-indene-2-sulfonyl chloride (4i)

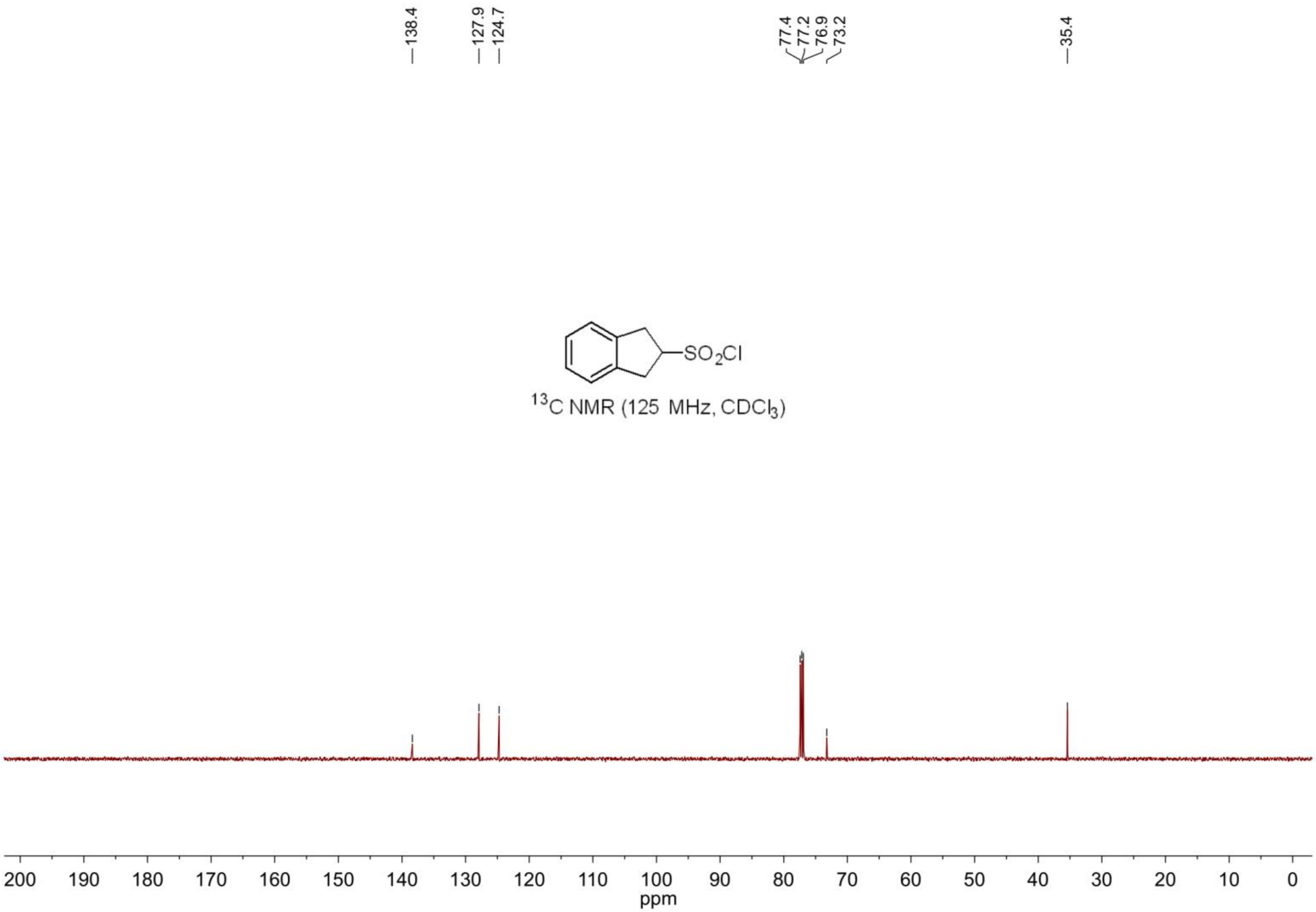

Go back to table of contents

((1*S,3*R**)-3-Acetyl-2,2-dimethylcyclobutyl)methanesulfonyl chloride (4j)**

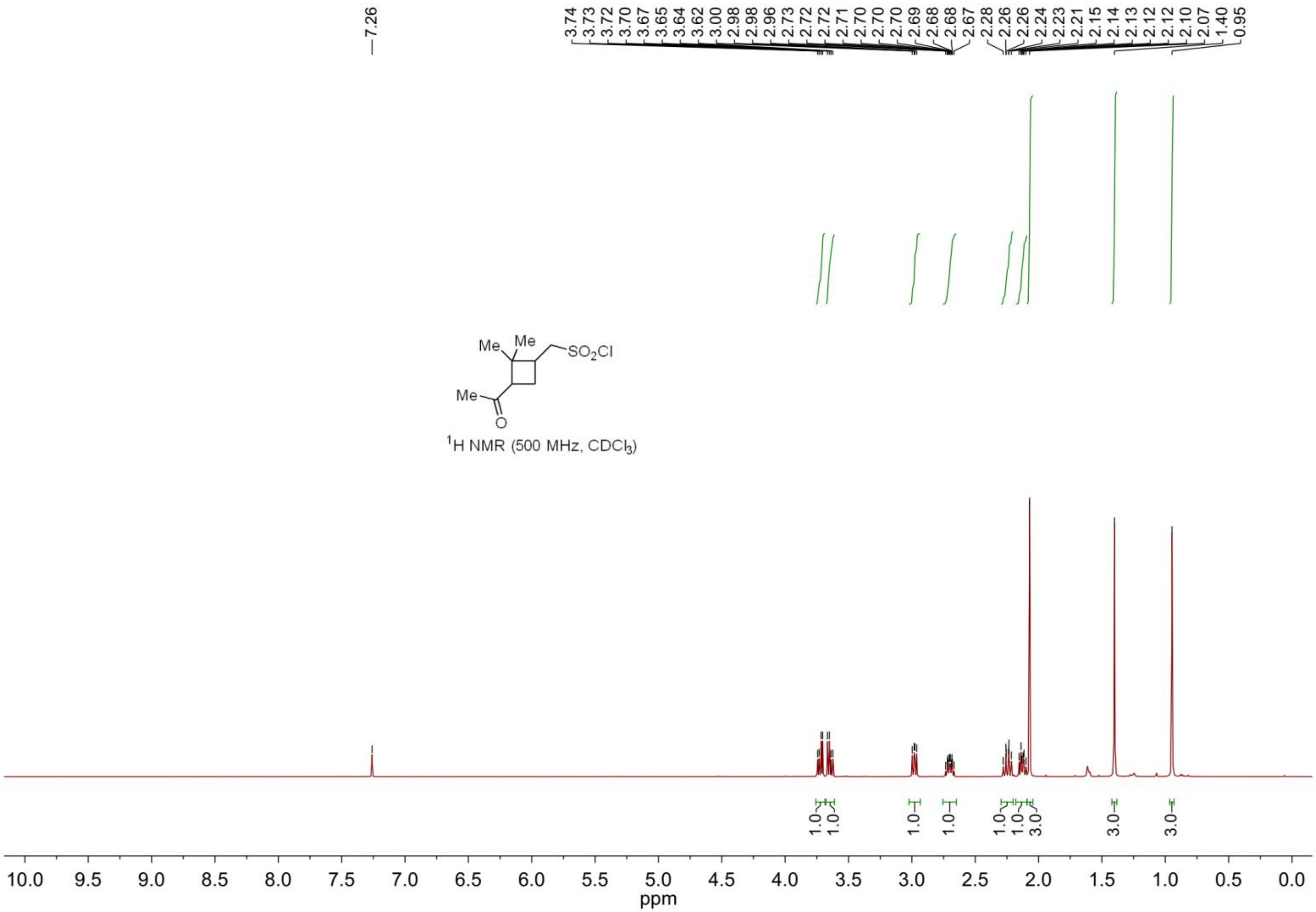

Go back to table of contents

((1*S**,3*R**)-3-Acetyl-2,2-dimethylcyclobutyl)methanesulfonyl chloride (4j)

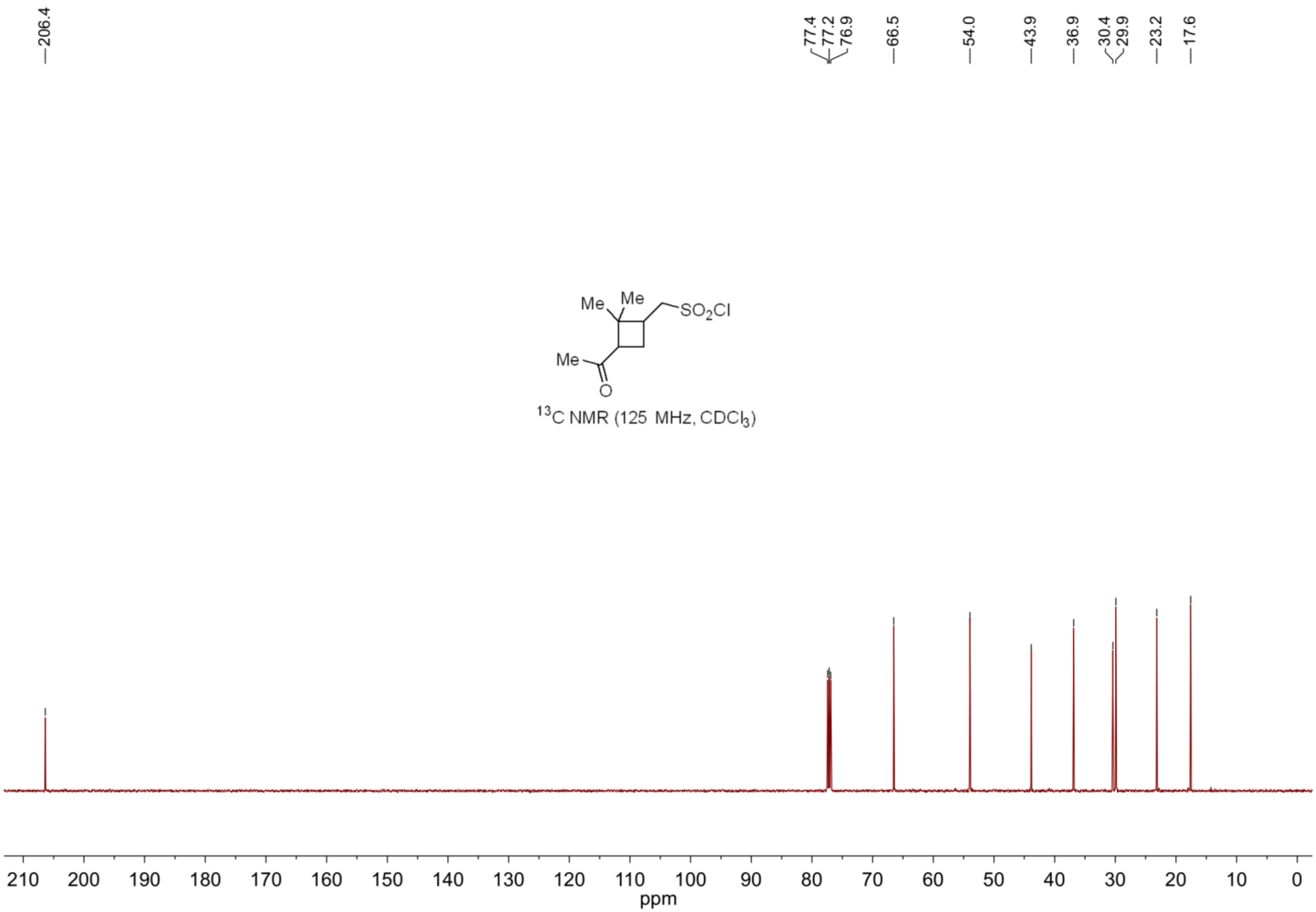

Go back to table of contents

(3*R*,7*R*,8*R*,9*S*,10*S*,13*R*,14*S*,17*R*)-17-((*R*)-4-(Chlorosulfonyl)butan-2-yl)-10,13-dimethylhexadecahydro-1*H*-cyclopenta[*a*]phenanthrene-3,7-diyl diacetate (4k)

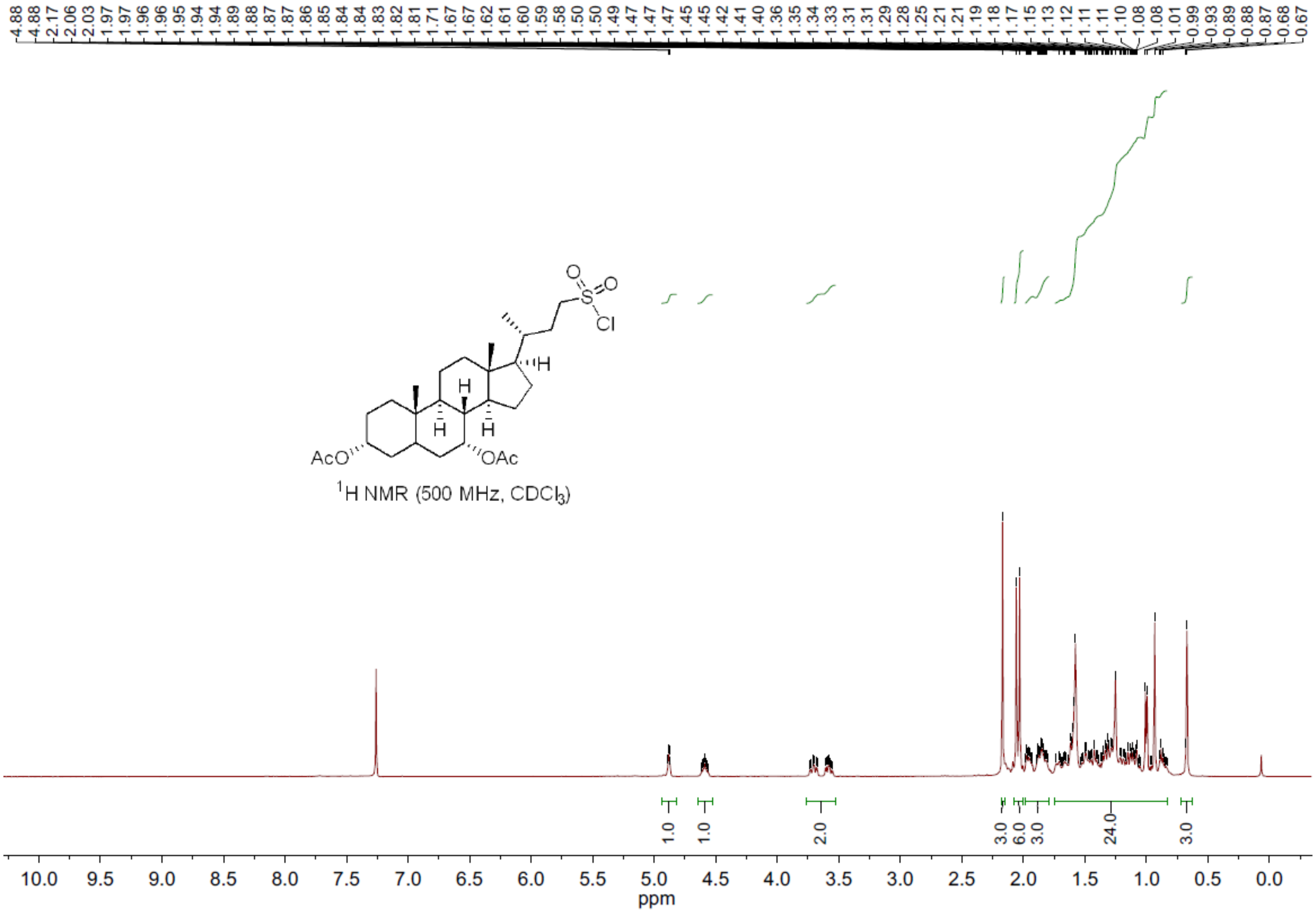

Go back to table of contents

(3*R*,7*R*,8*R*,9*S*,10*S*,13*R*,14*S*,17*R*)-17-((*R*)-4-(Chlorosulfonyl)butan-2-yl)-10,13-dimethylhexadecahydro-1*H*-cyclopenta[*a*]phenanthrene-3,7-diyl diacetate (4k)

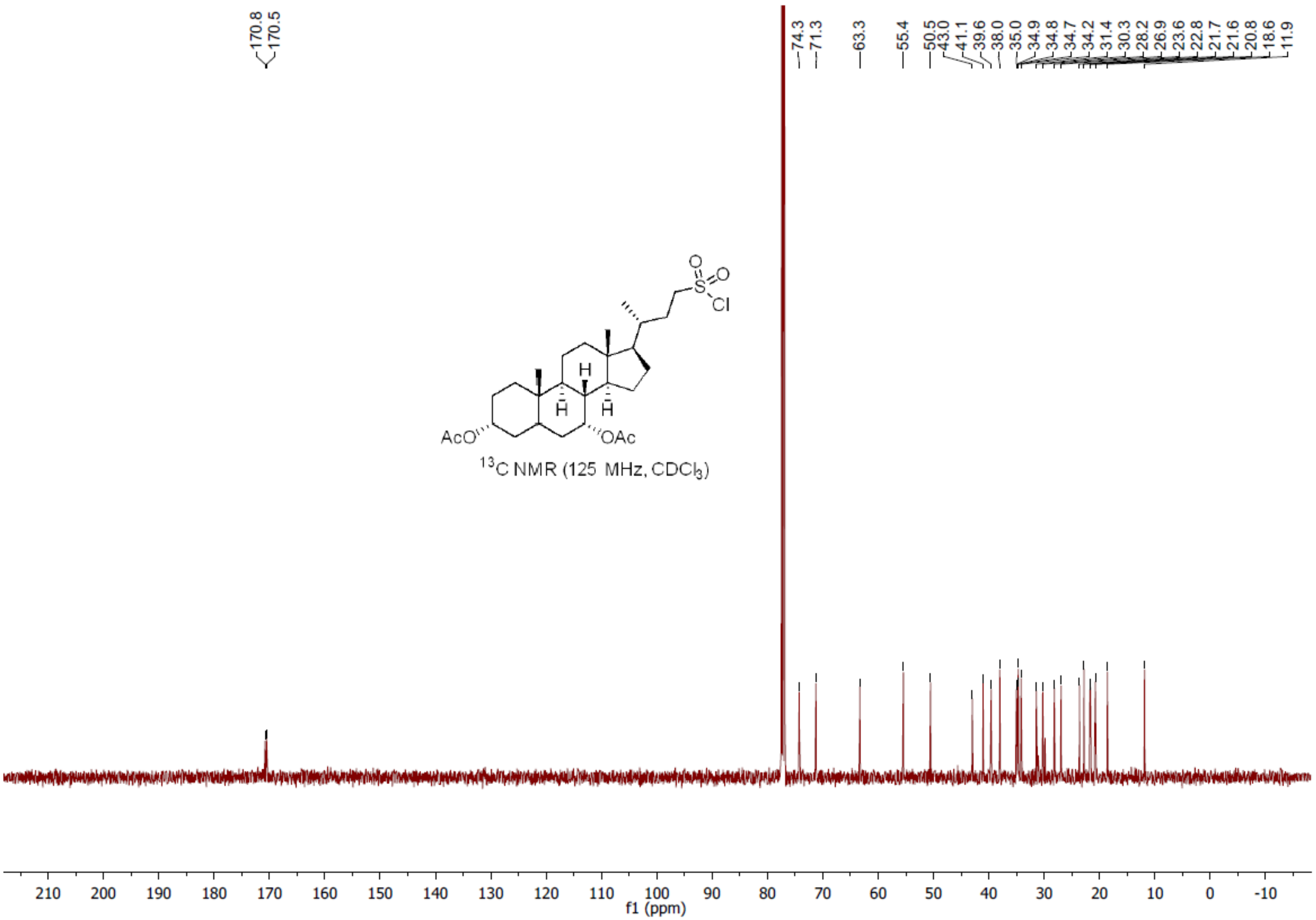

Go back to table of contents

4-Oxo-4-phenylbutane-1-sulfonyl fluoride (5a)

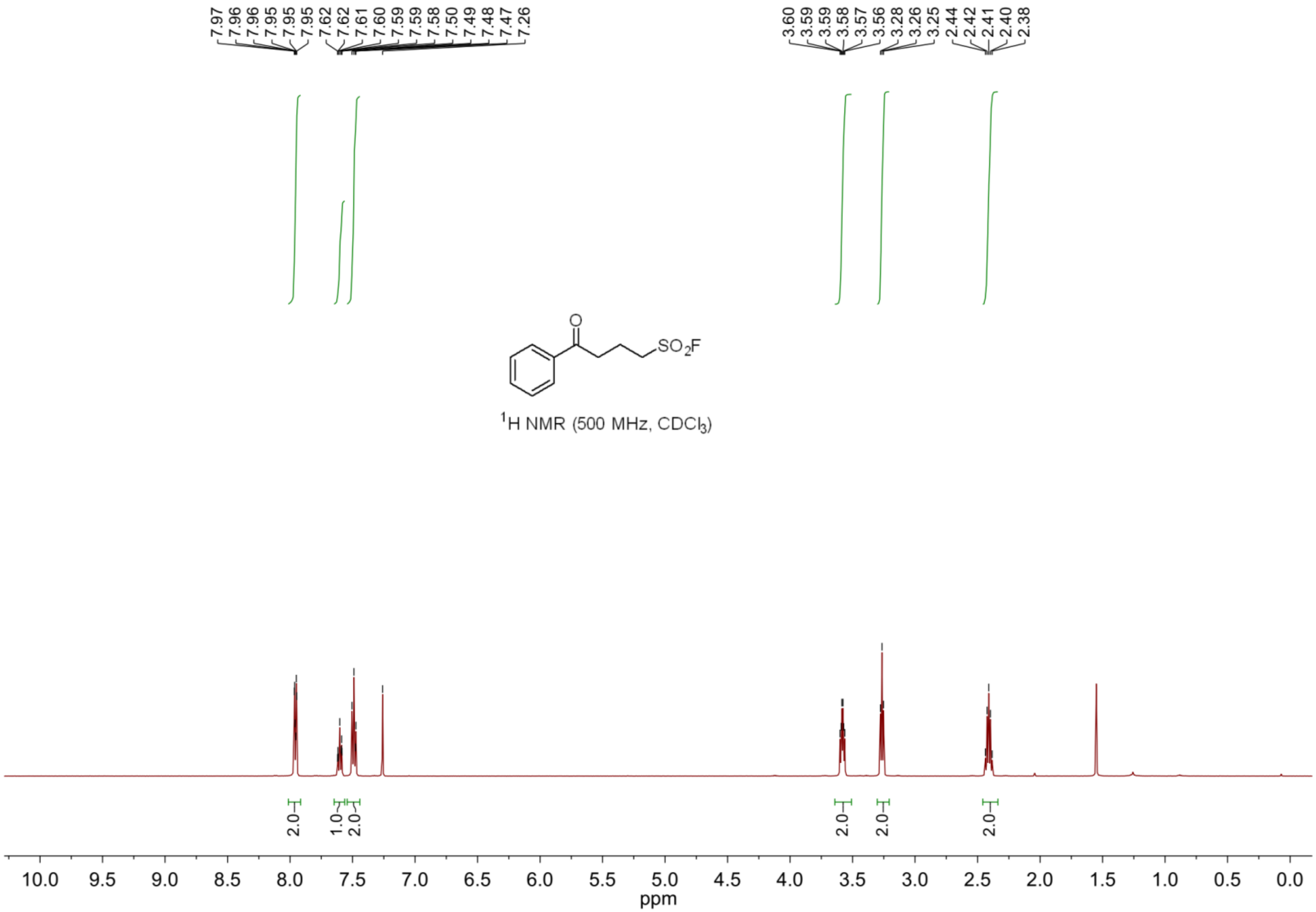

Go back to table of contents

4-Oxo-4-phenylbutane-1-sulfonyl fluoride (5a)

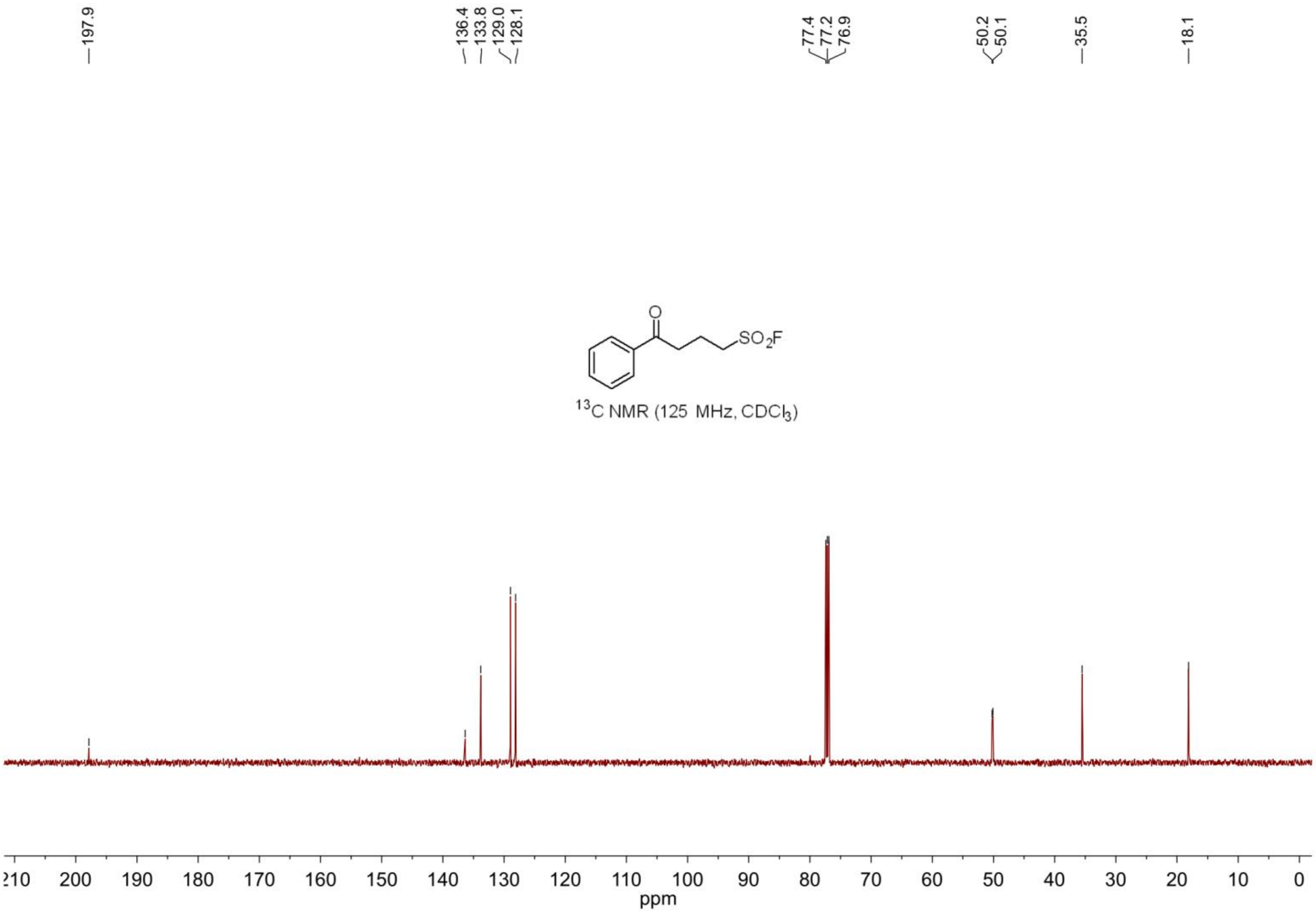

Go back to table of contents

Methyl 5-(fluorosulfonyl)pentanoate (5b)

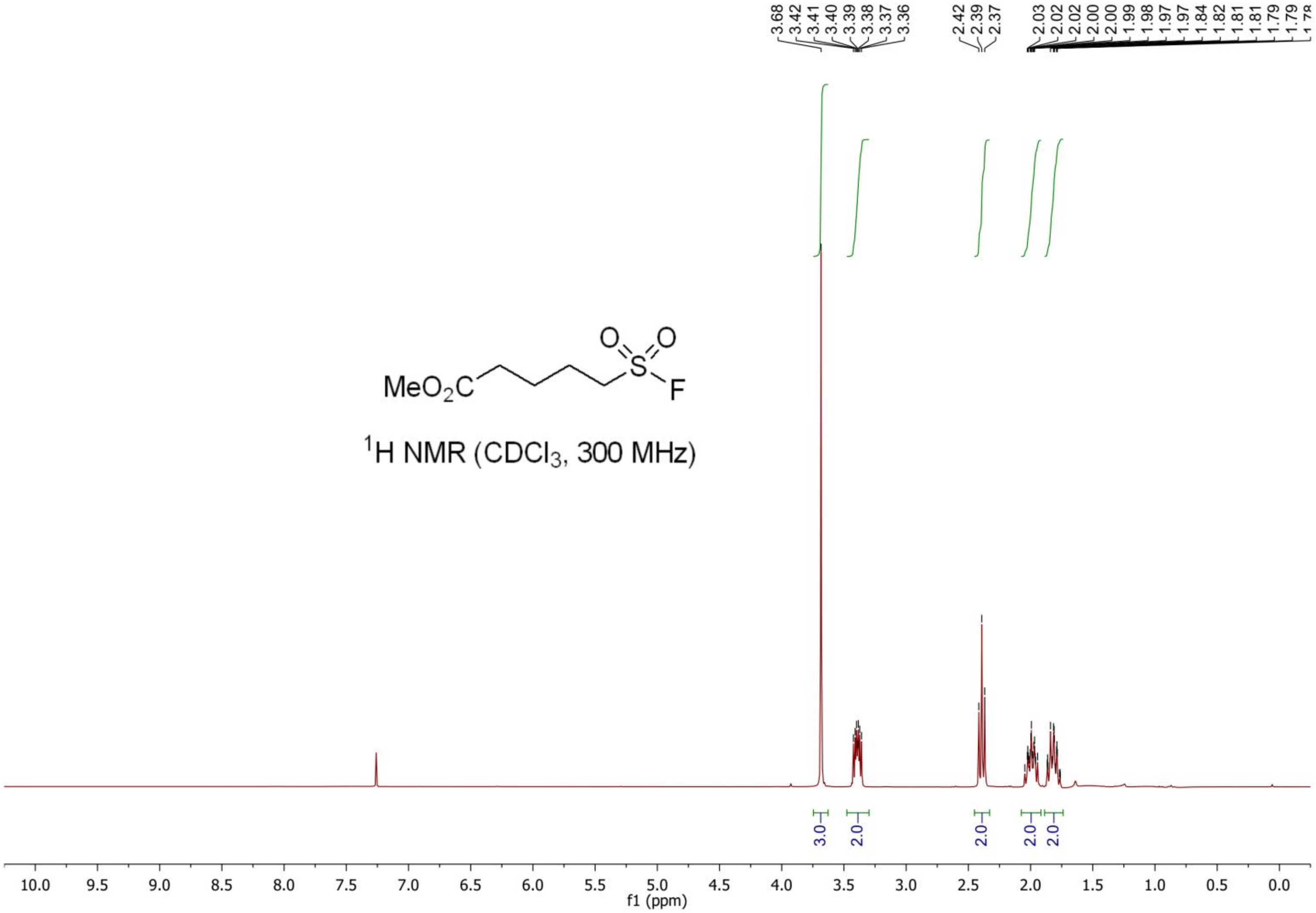

Methyl 5-(fluorosulfonyl)pentanoate (5b)

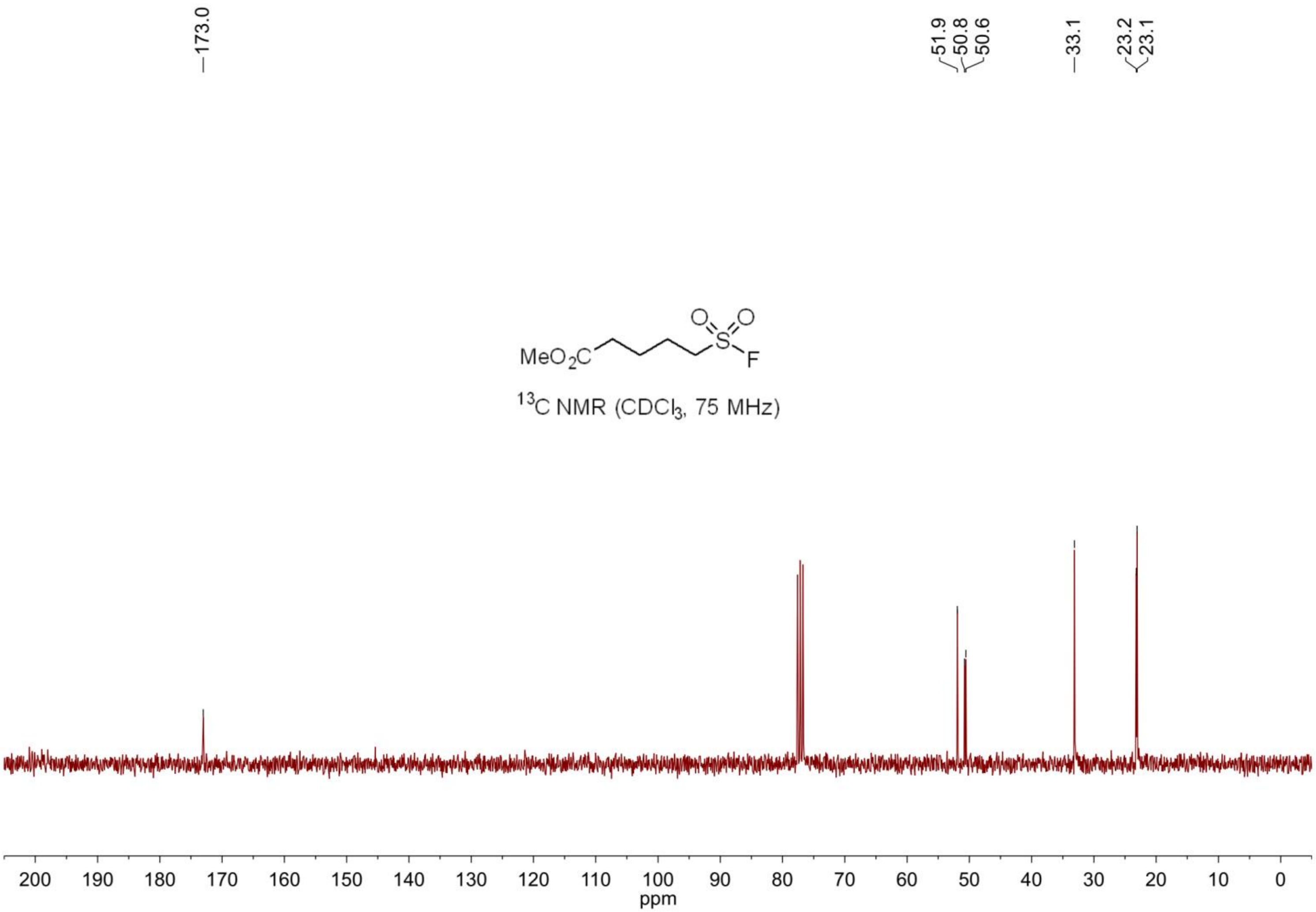

Go back to table of contents

Pentadecane-1-sulfonyl fluoride (5c)

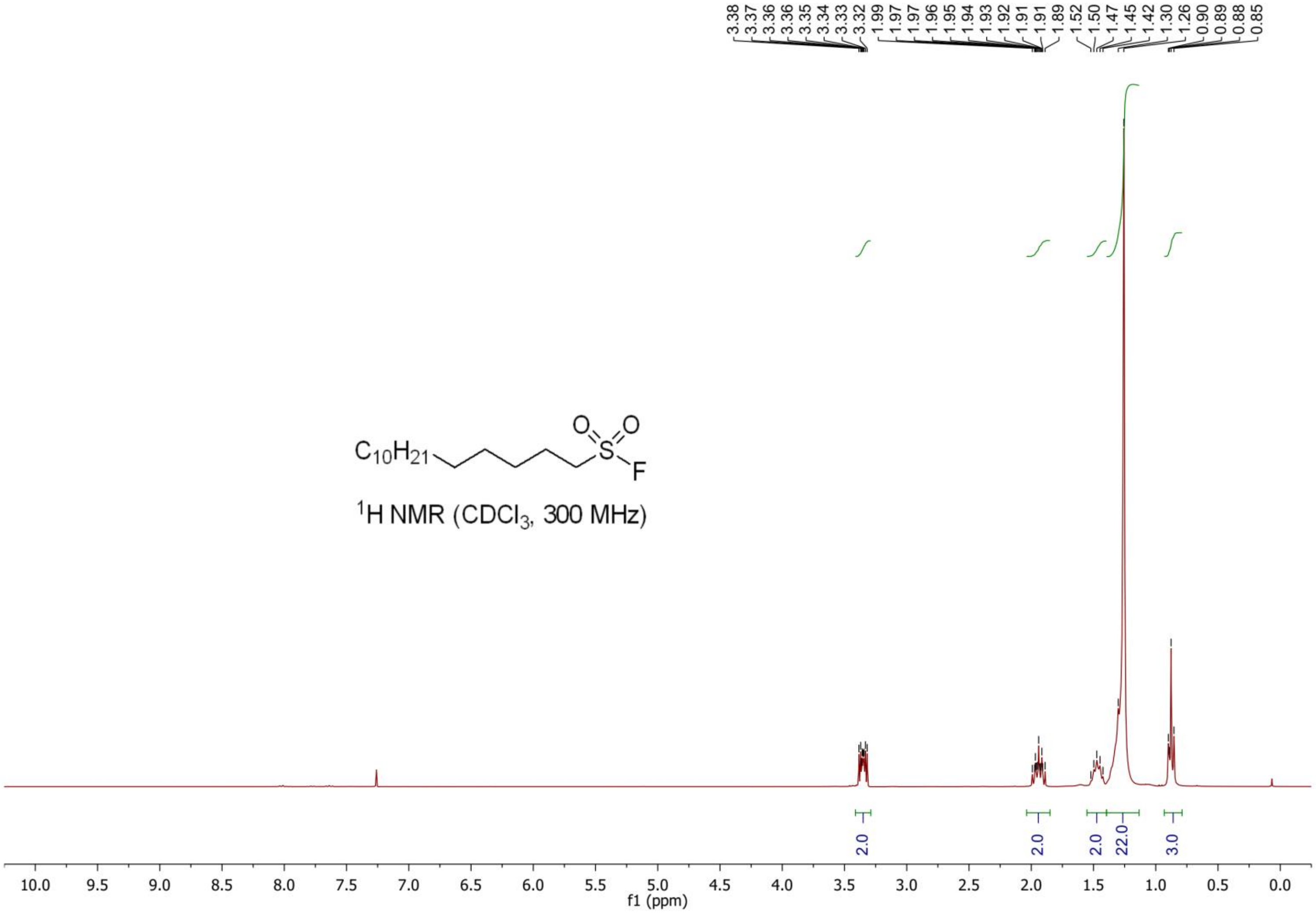

Pentadecane-1-sulfonyl fluoride (5c)

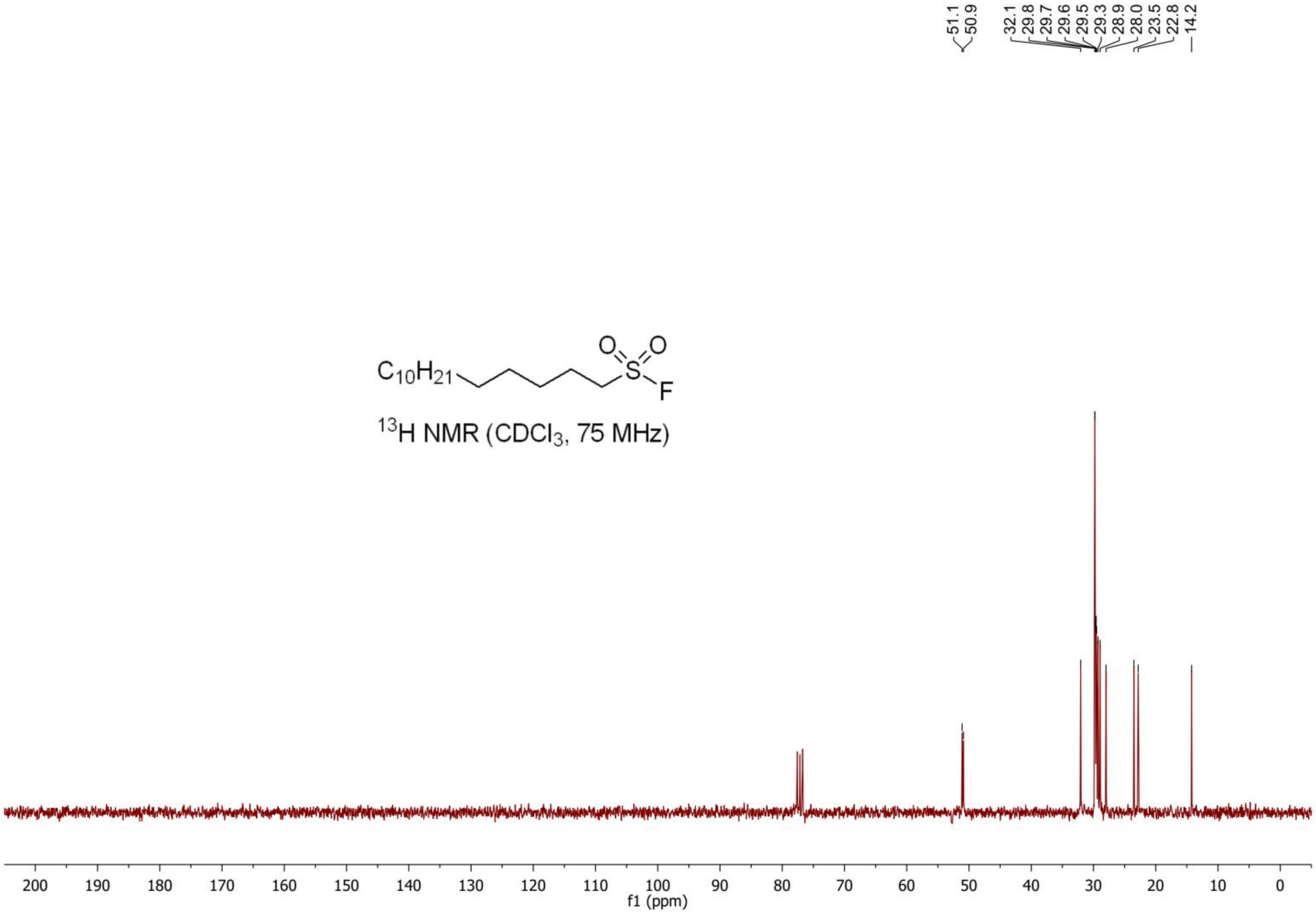

Go back to table of contents

Heptadecane-1-sulfonyl fluoride (5d)

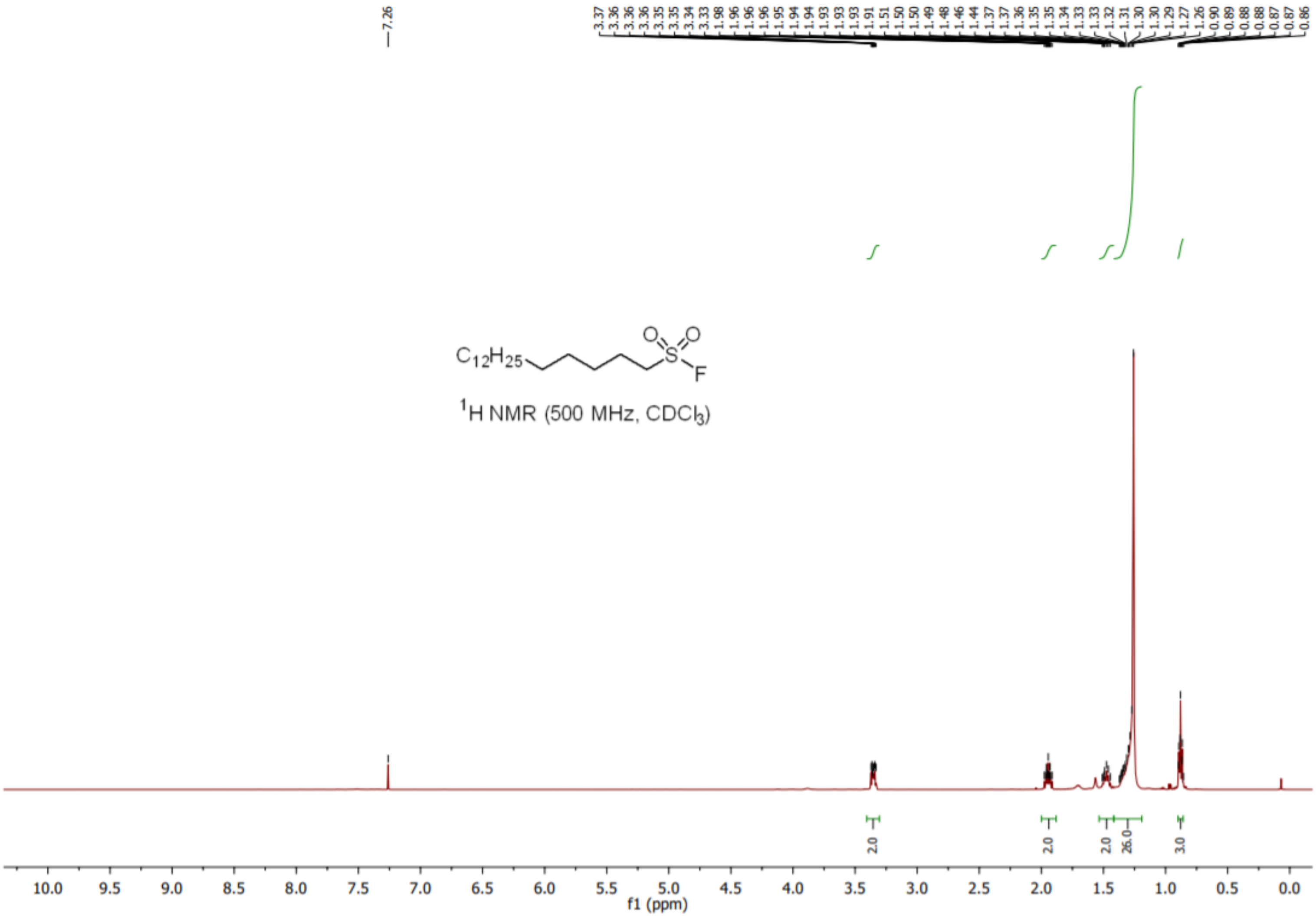

Go back to table of contents

Heptadecane-1-sulfonyl fluoride (5d)

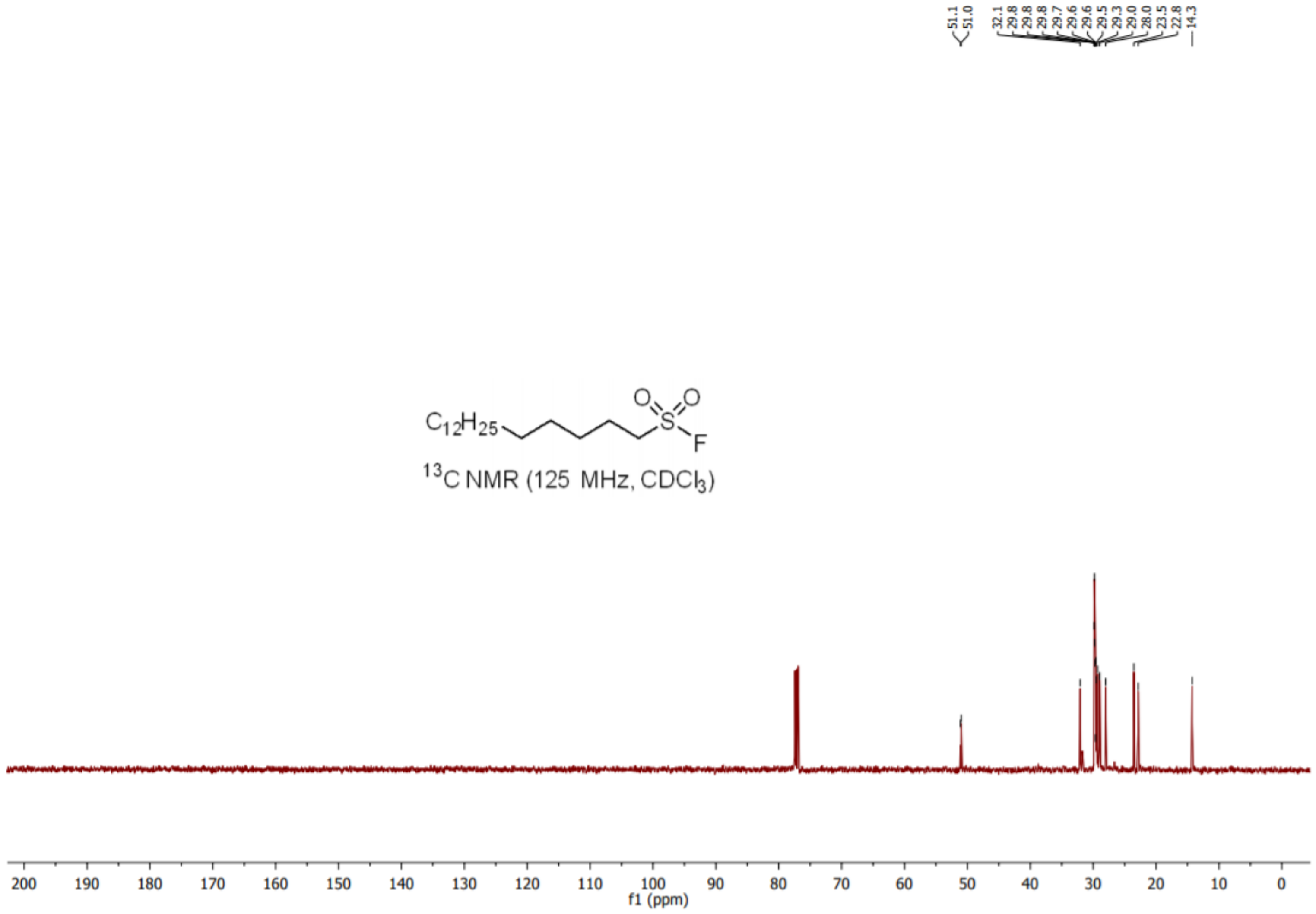

Go back to table of contents

2-(4-Fluorophenyl)ethane-1-sulfonyl fluoride (5e)

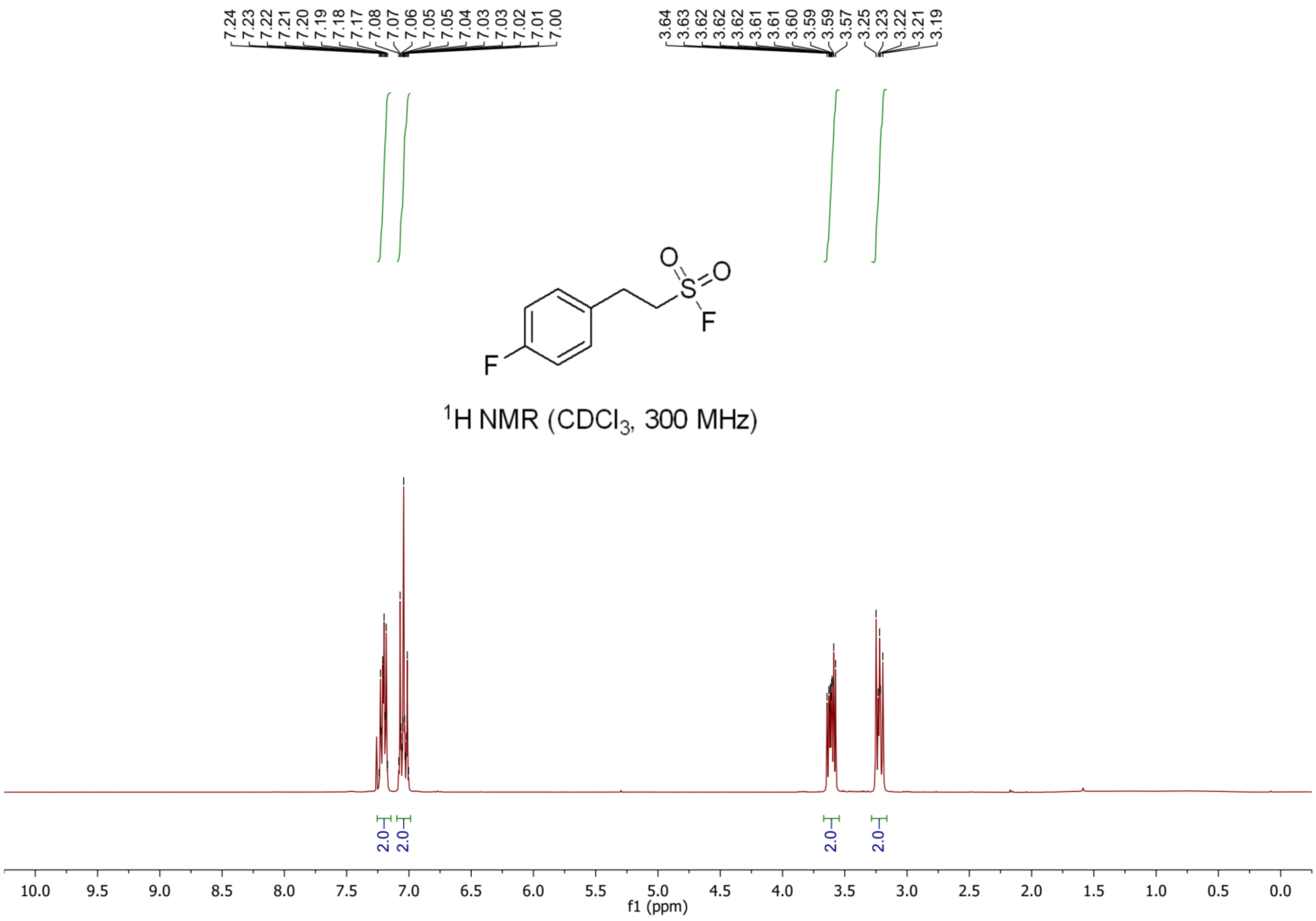

2-(4-Fluorophenyl)ethane-1-sulfonyl fluoride (5e)

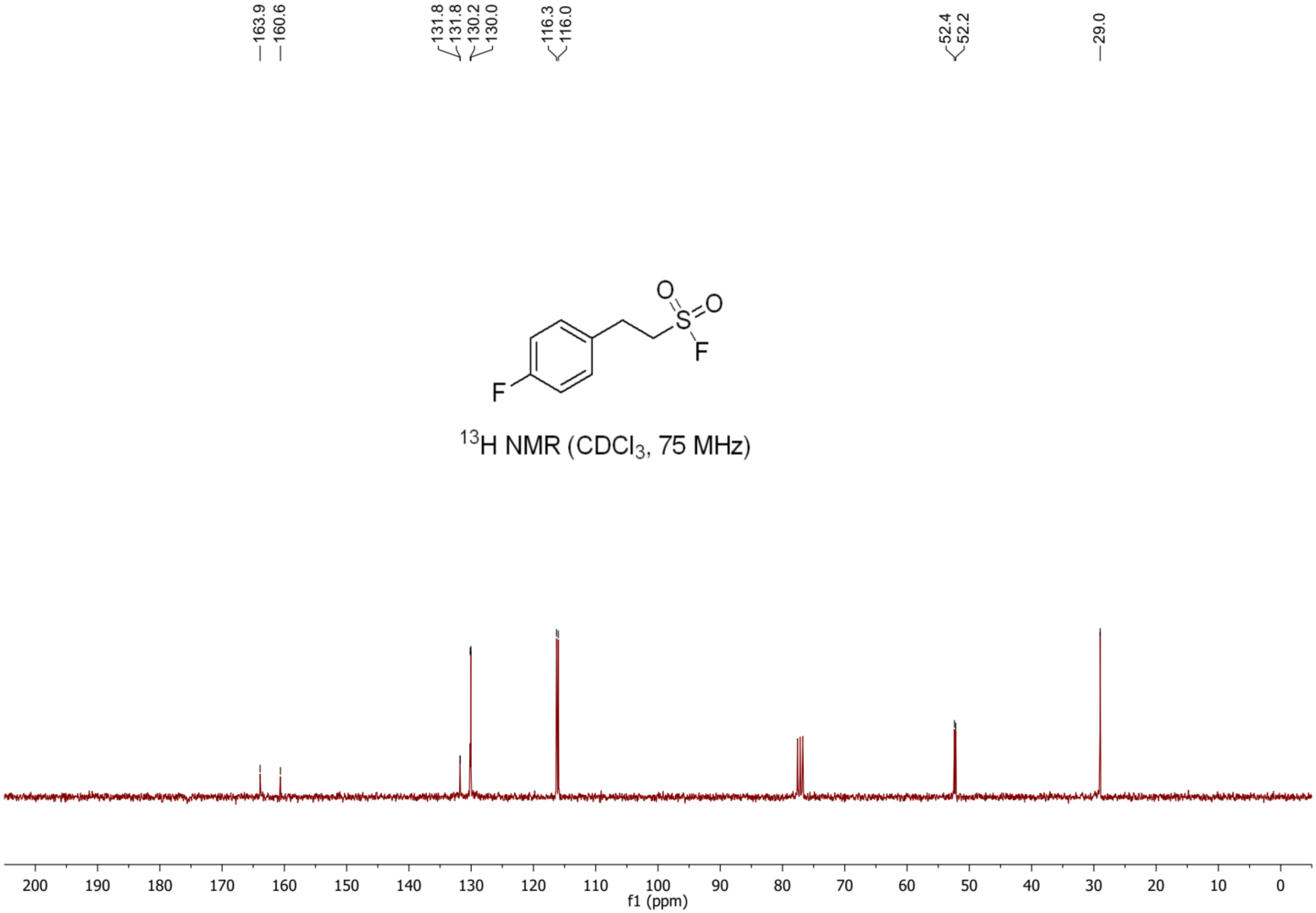

Go back to table of contents

Pentadecane-7-sulfonyl fluoride (5f)

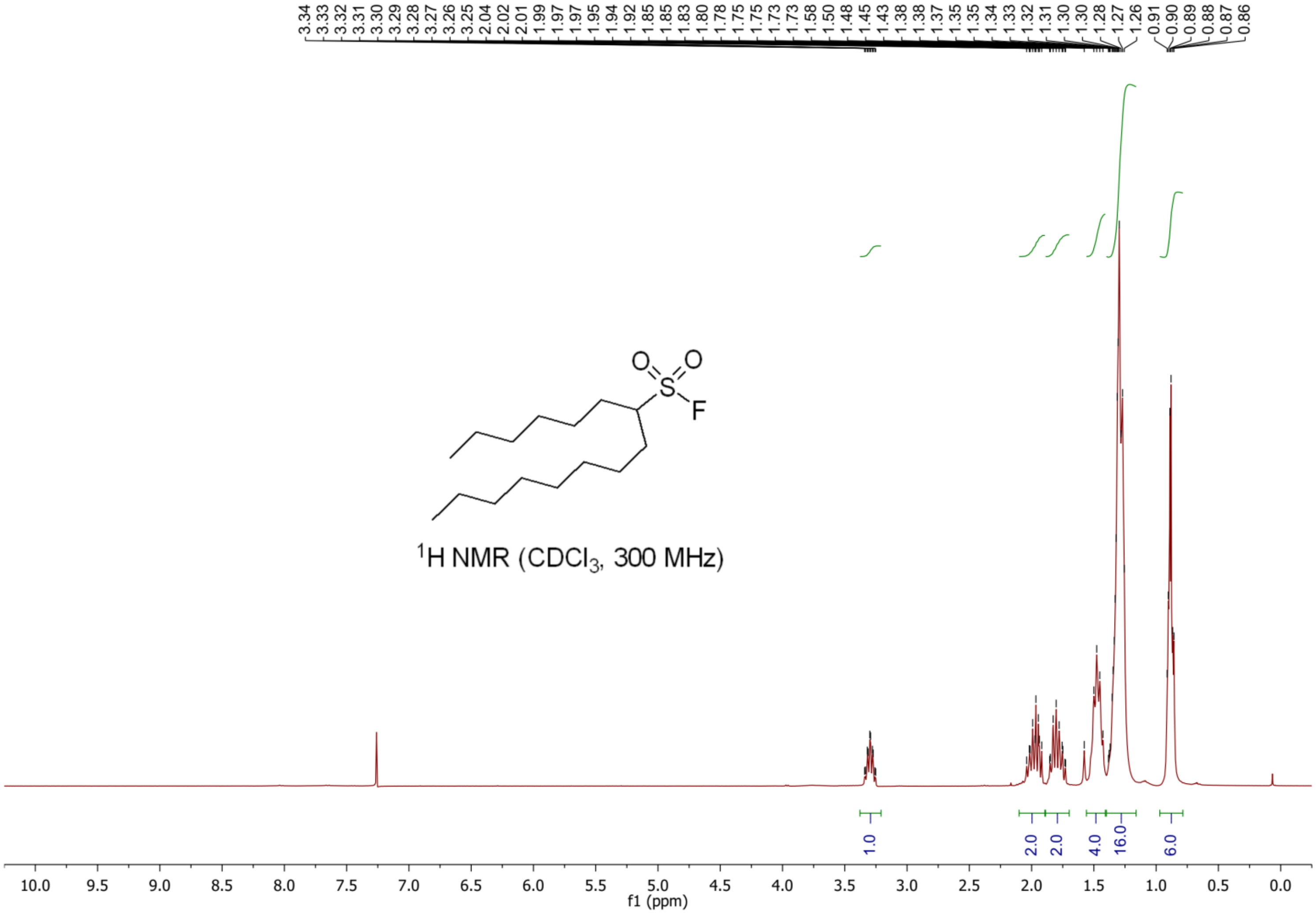

Go back to table of contents

Pentadecane-7-sulfonyl fluoride (5f)

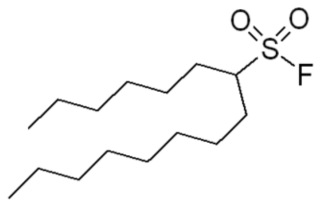

^{13}H NMR ($CDCl_3$, 75 MHz)

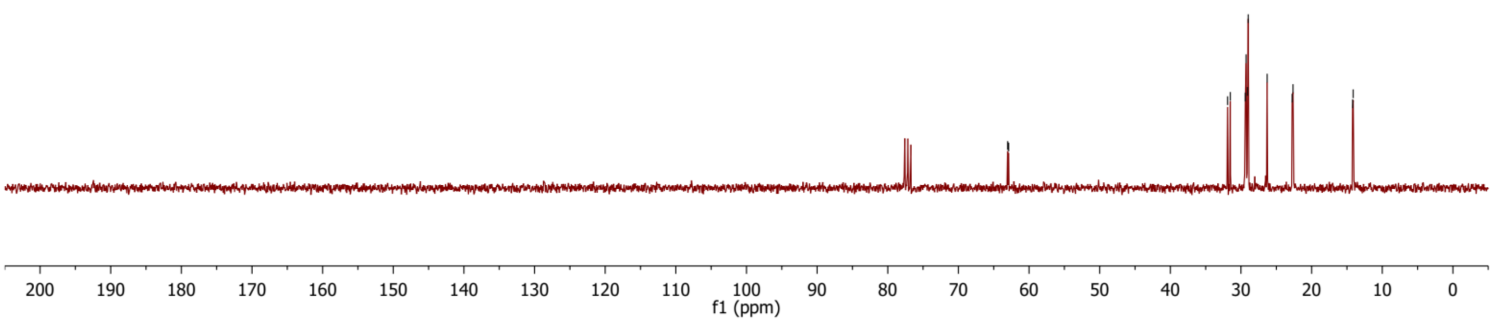

Go back to table of contents

Cyclohexanesulfonyl fluoride (5g)

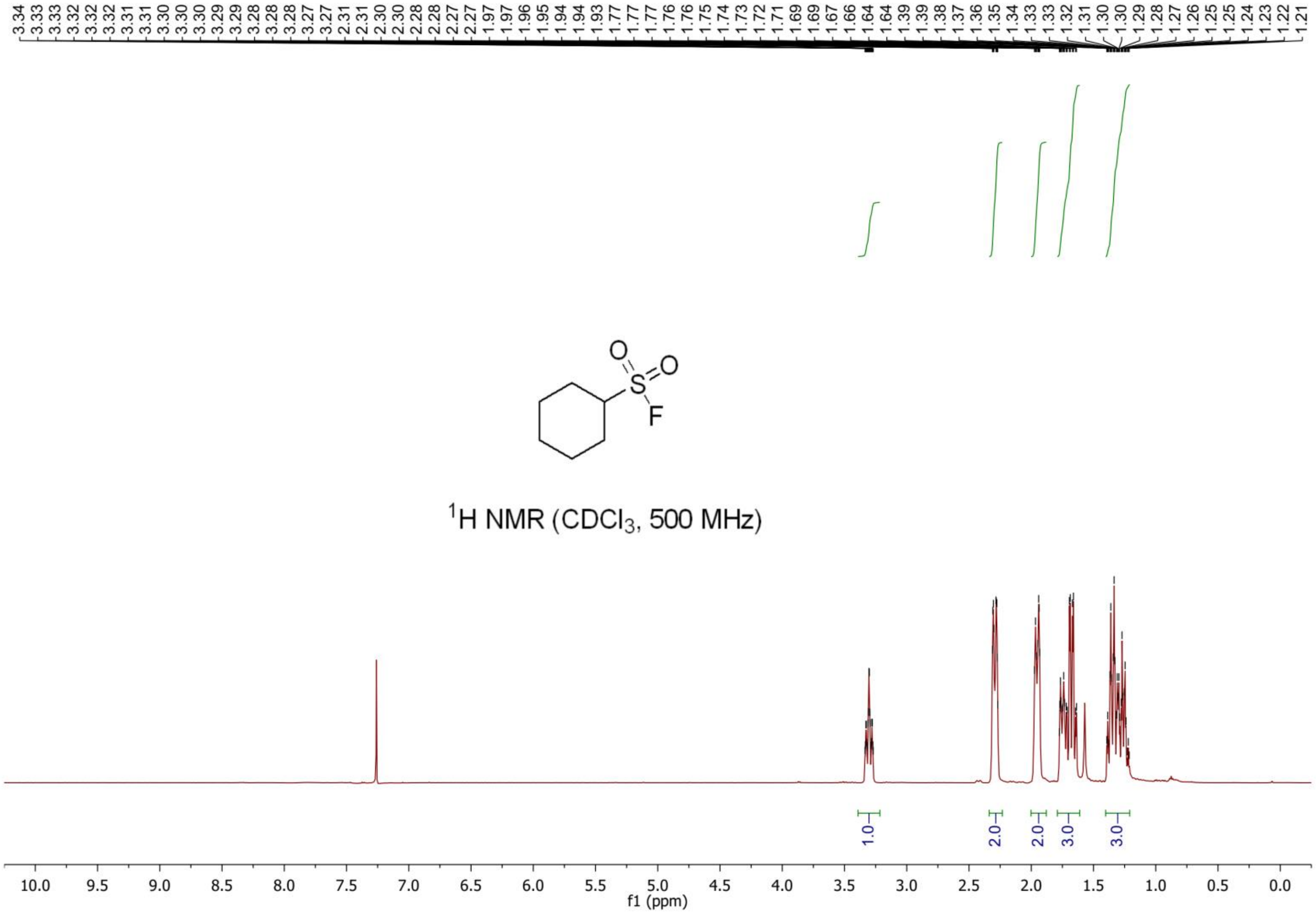

Go back to table of contents

Cyclohexanesulfonyl fluoride (5g)

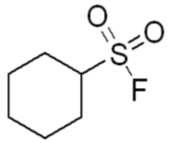

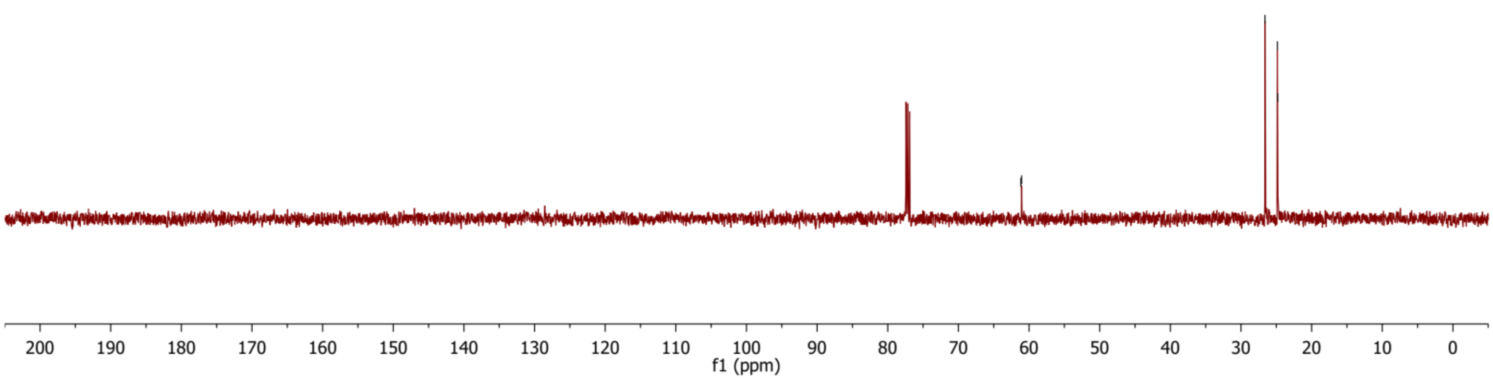

^{13}H NMR (CDCl$_3$, 125 MHz)

Go back to table of contents

Cycloheptanesulfonyl fluoride (5h)

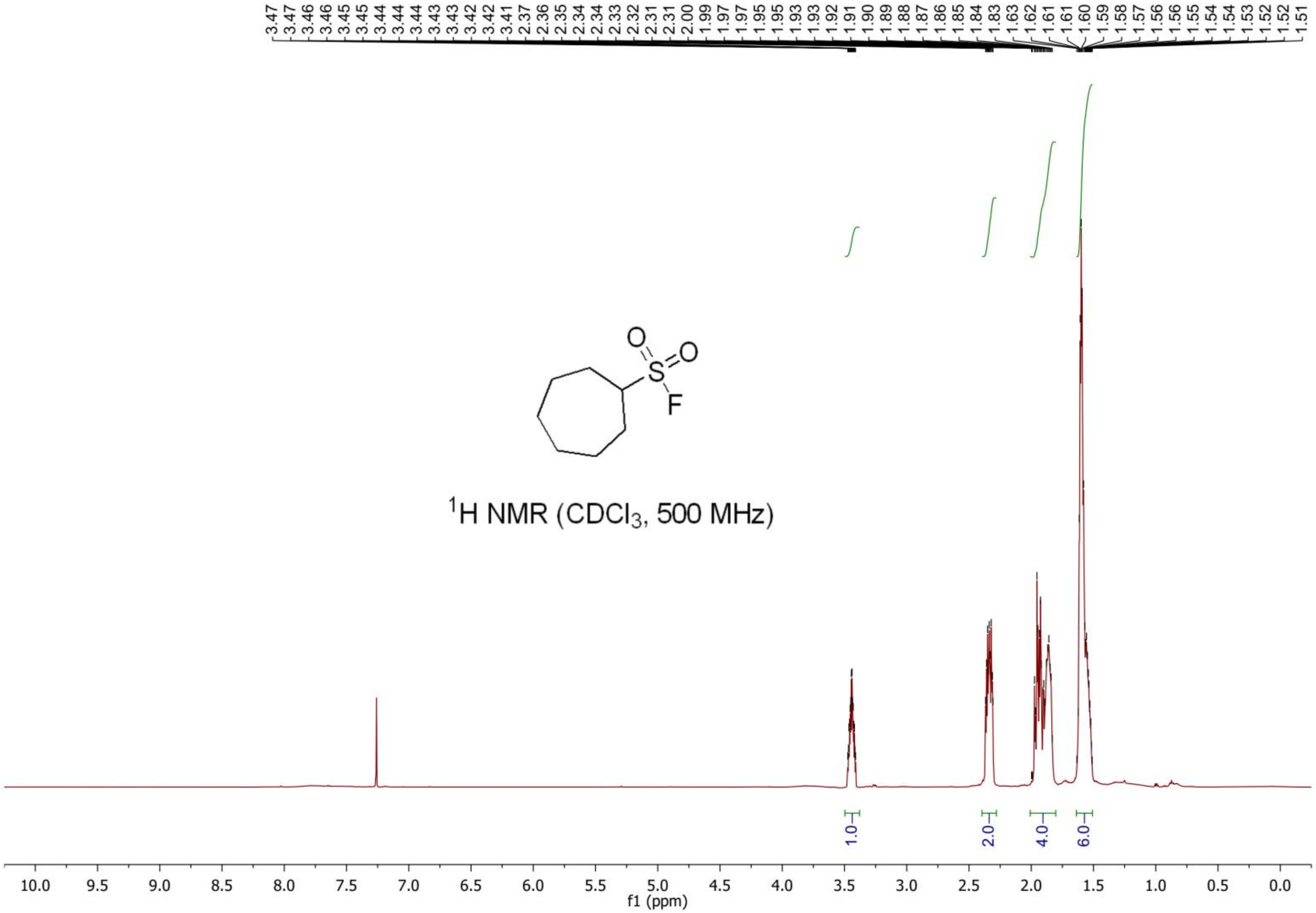

Go back to table of contents

Cycloheptanesulfonyl fluoride (5h)

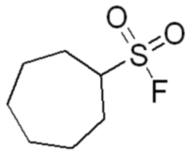

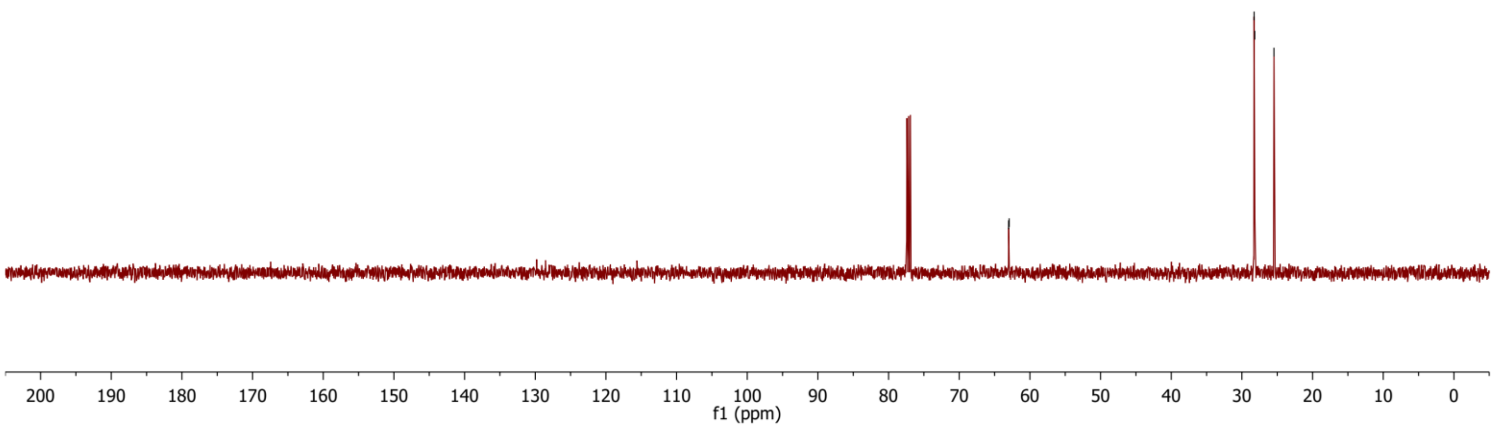

Go back to table of contents

((3a*S*,5a*R*,8a*R*,8b*S*)-2,2,7,7-Tetramethyltetrahydro-3a*H*-bis([1,3]dioxolo)[4,5-*b*:4',5'-*d*]pyran-3a-yl)methyl 4-(fluorosulfonyl)butanoate (5i)

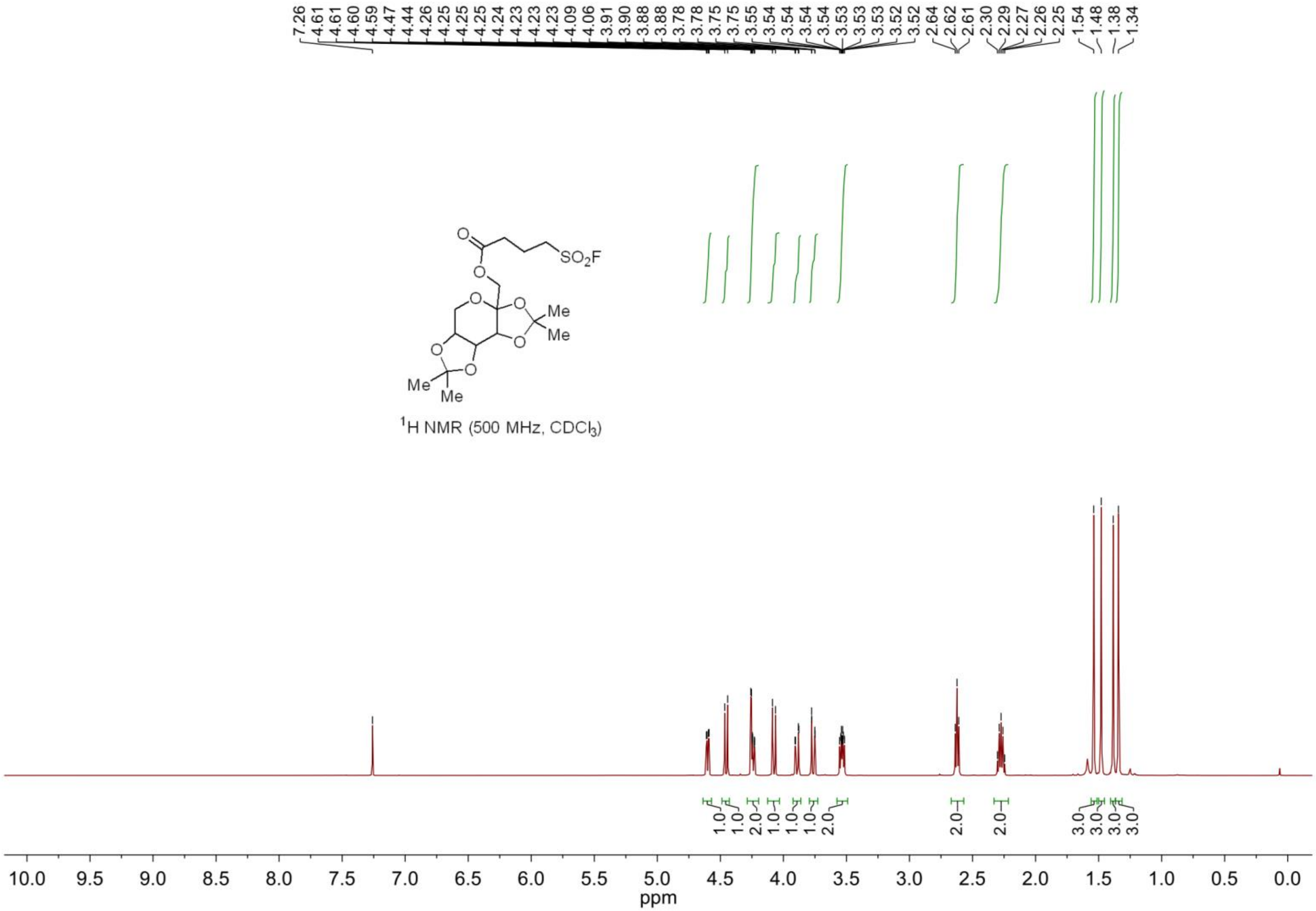

Go back to table of contents

((3a*S*,5a*R*,8a*R*,8b*S*)-2,2,7,7-Tetramethyltetrahydro-3a*H*-bis([1,3]dioxolo)[4,5-*b*:4',5'-*d*]pyran-3a-yl)methyl 4-(fluorosulfonyl)butanoate (5i)

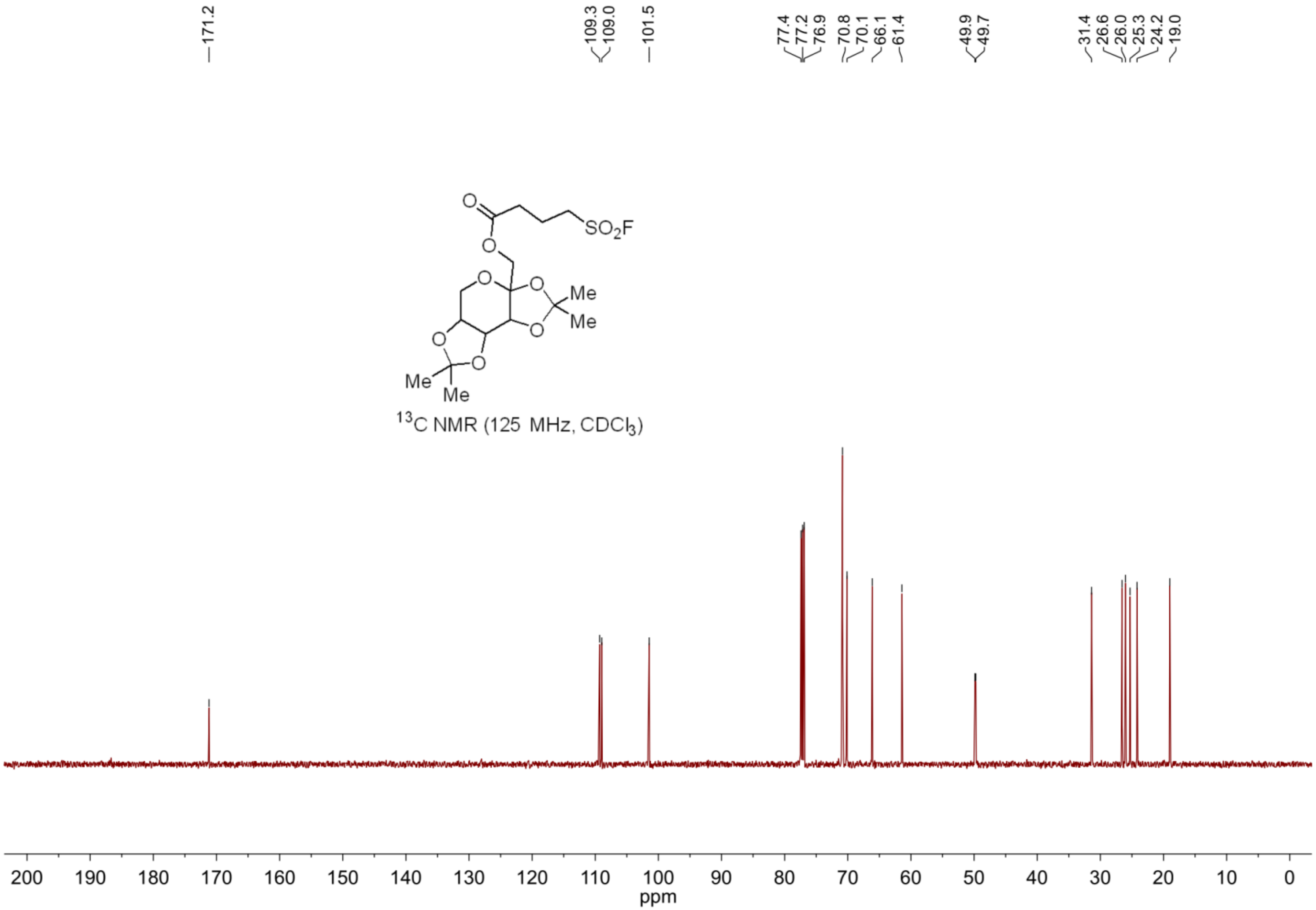

Go back to table of contents

Heptane-4-sulfonyl fluoride (5j)

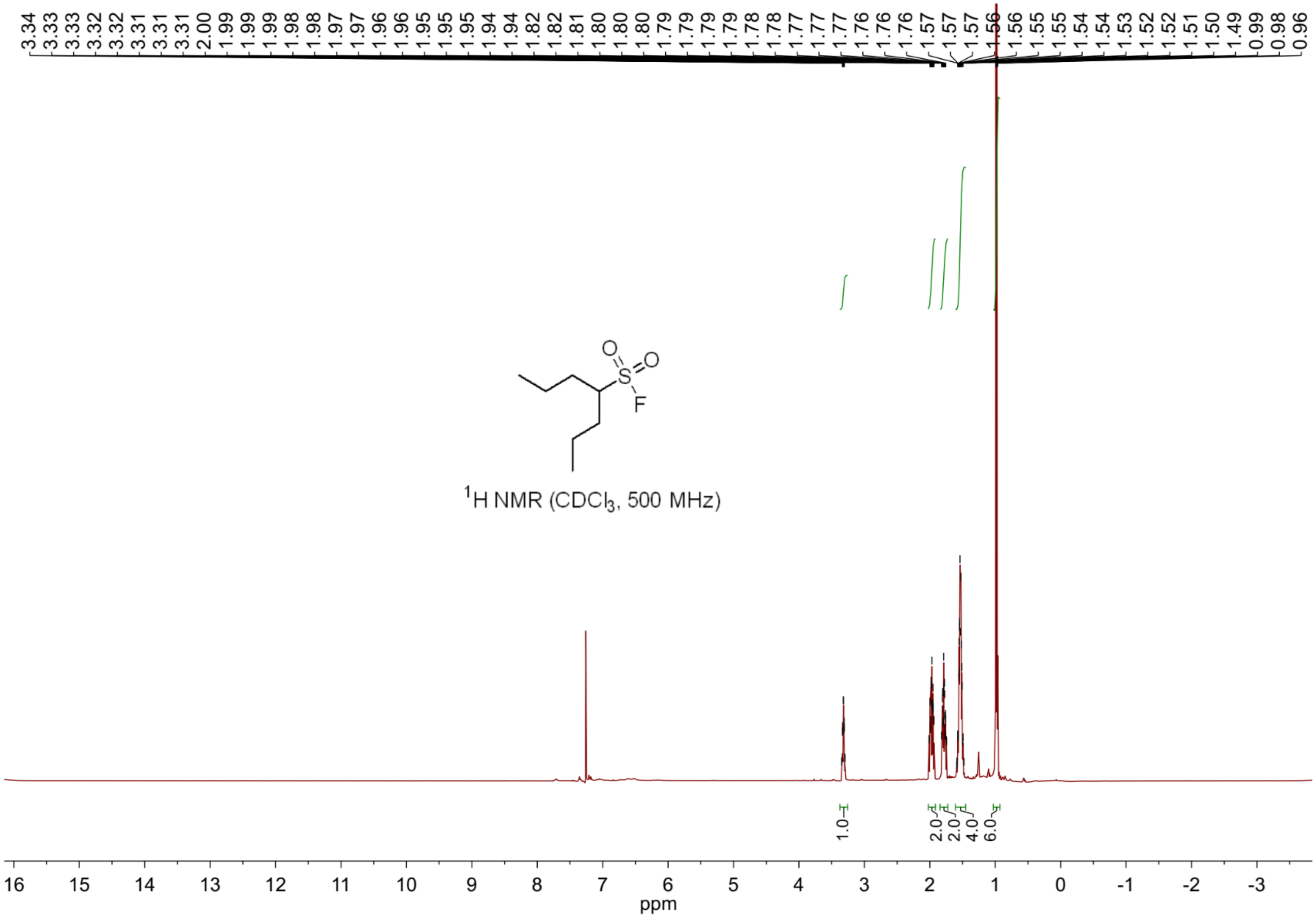

Go back to table of contents

Heptane-4-sulfonyl fluoride (5j)

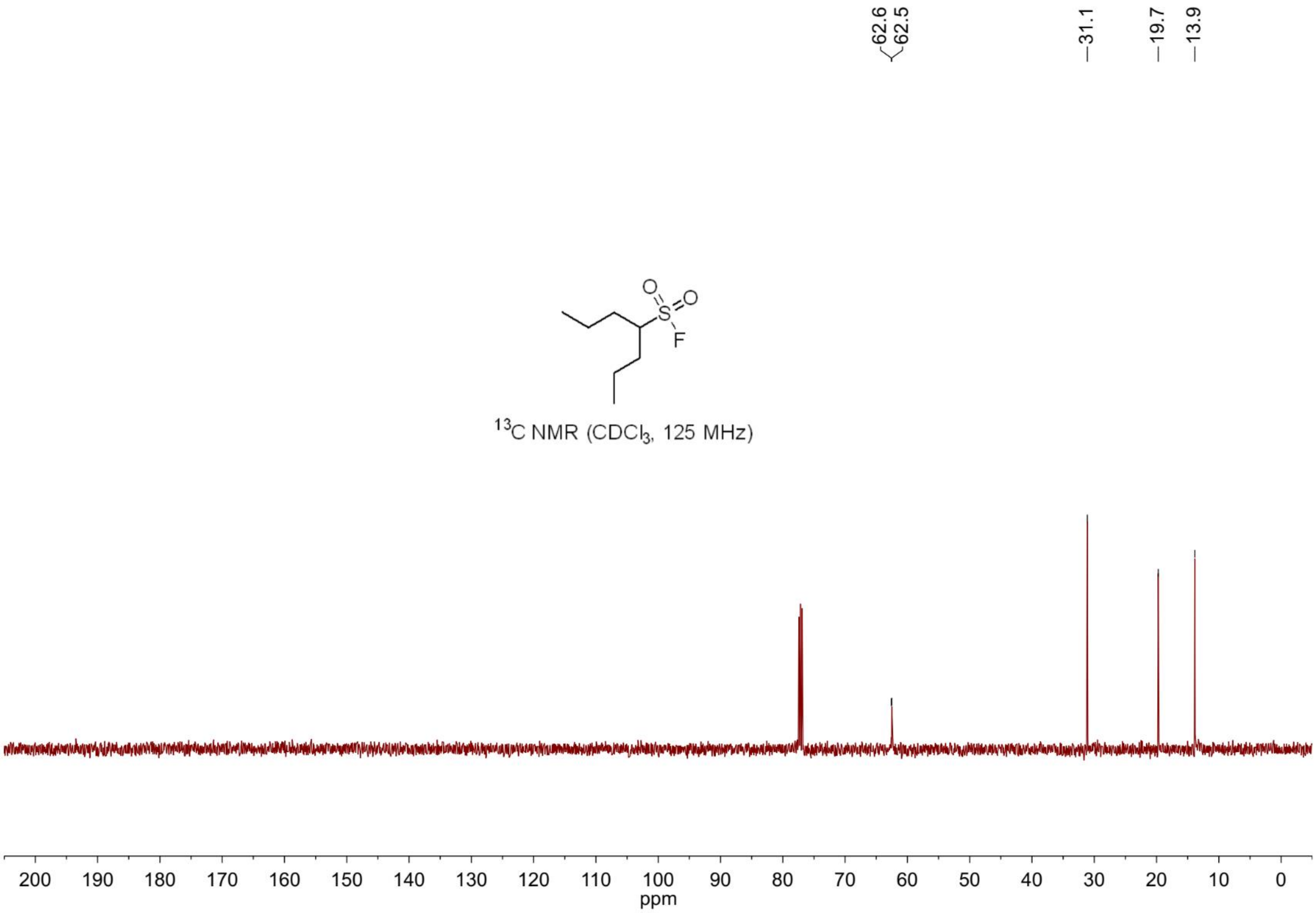

Go back to table of contents

(3*R*,5*R*,8*R*,9*S*,10*S*,12*S*,13*R*,14*S*,17*R*)-17-((*R*)-4-(Fluorosulfonyl)butan-2-yl)-10,13-dimethylhexadecahydro-1*H*-cyclopenta[*a*]phenanthrene-3,12-diyl diacetate (5k)

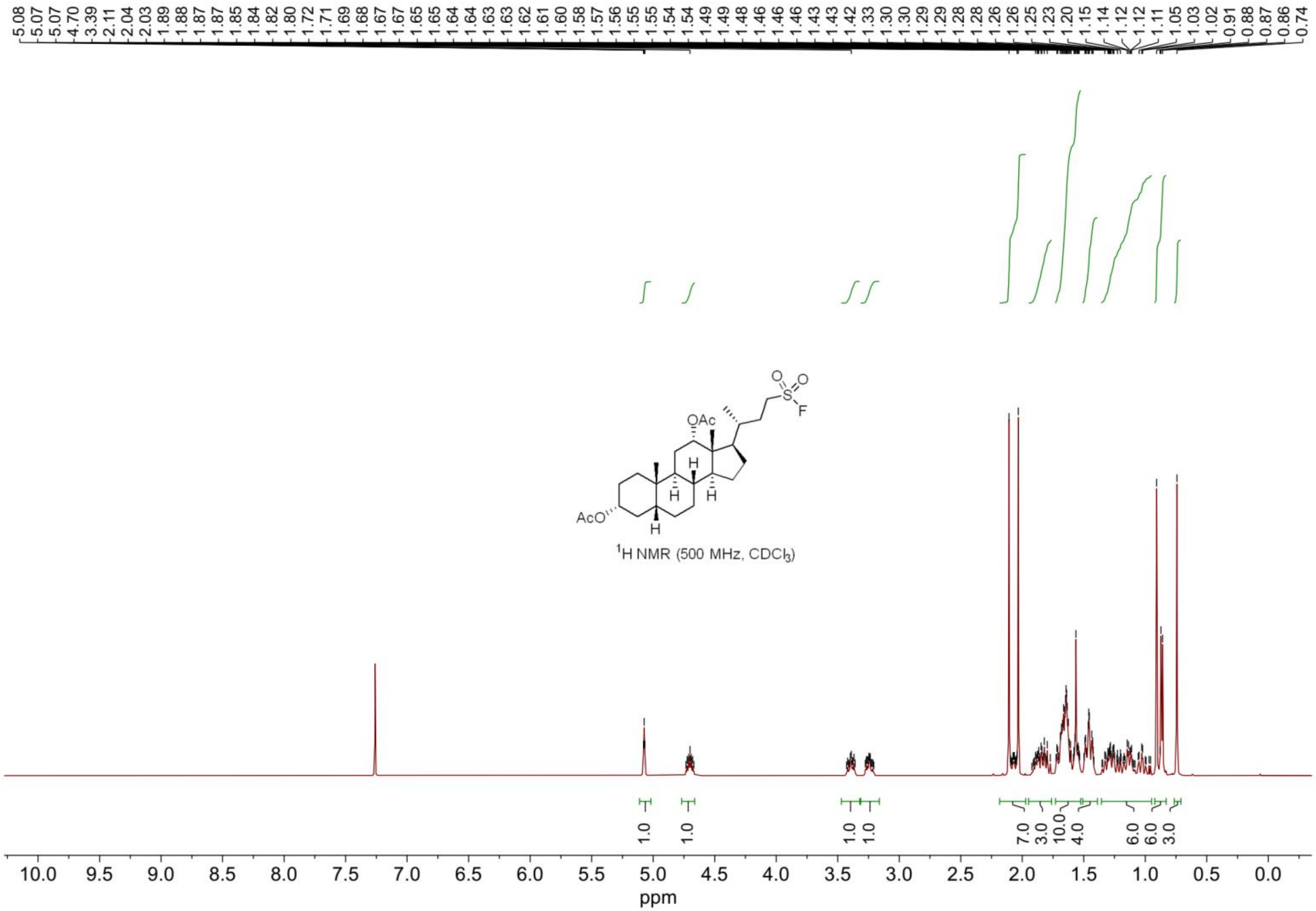

Go back to table of contents

(3*R*,5*R*,8*R*,9*S*,10*S*,12*S*,13*R*,14*S*,17*R*)-17-((*R*)-4-(Fluorosulfonyl)butan-2-yl)-10,13-dimethylhexadecahydro-1*H*-cyclopenta[*a*]phenanthrene-3,12-diyl diacetate (5k)

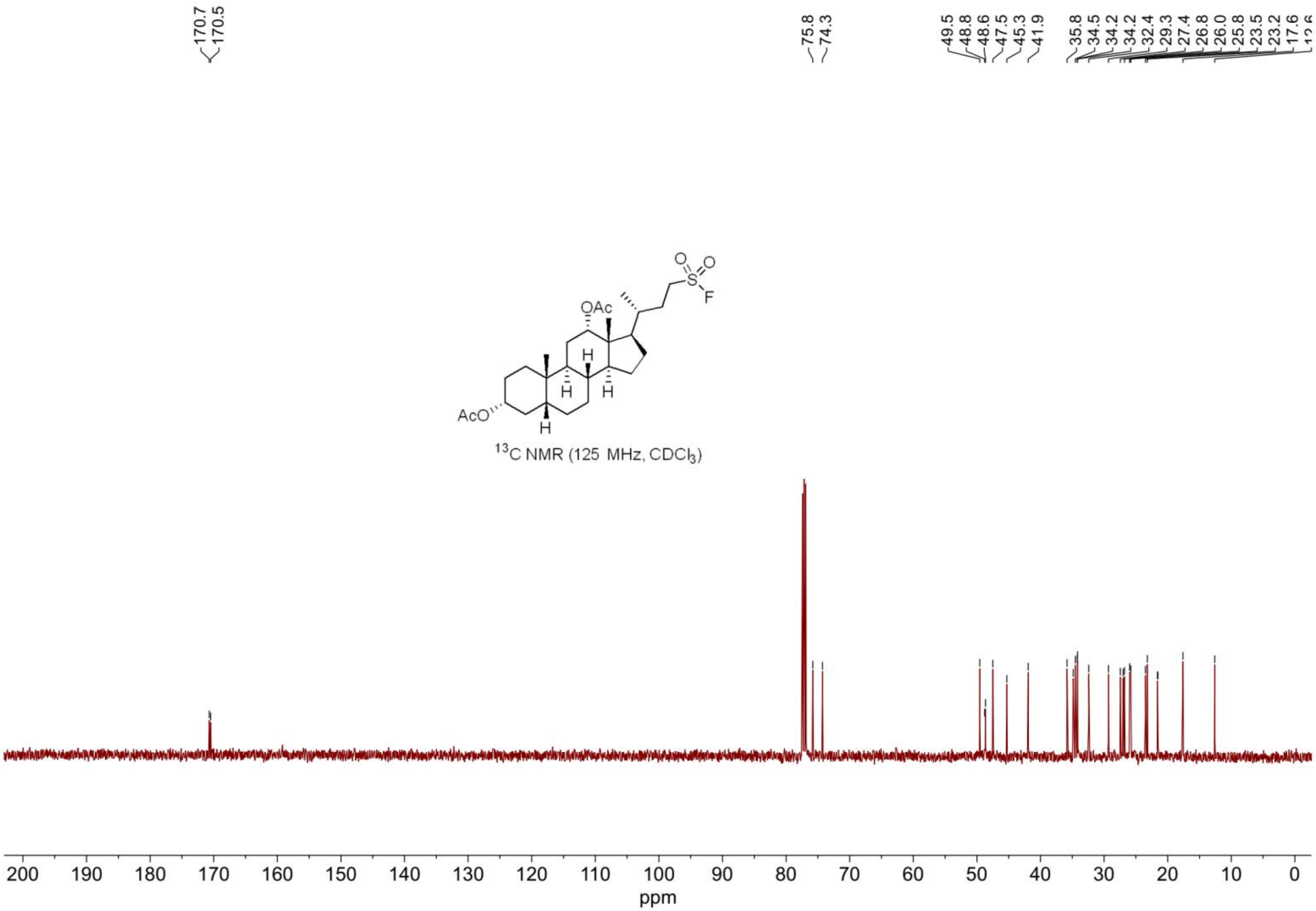

Go back to table of contents

(3*R*,7*R*,8*R*,9*S*,10*S*,13*R*,14*S*,17*R*)-17-((*R*)-4-(Fluorosulfonyl)butan-2-yl)-10,13-dimethylhexadecahydro-1*H*-cyclopenta[*a*]phenanthrene-3,7-diyl diacetate (5l)

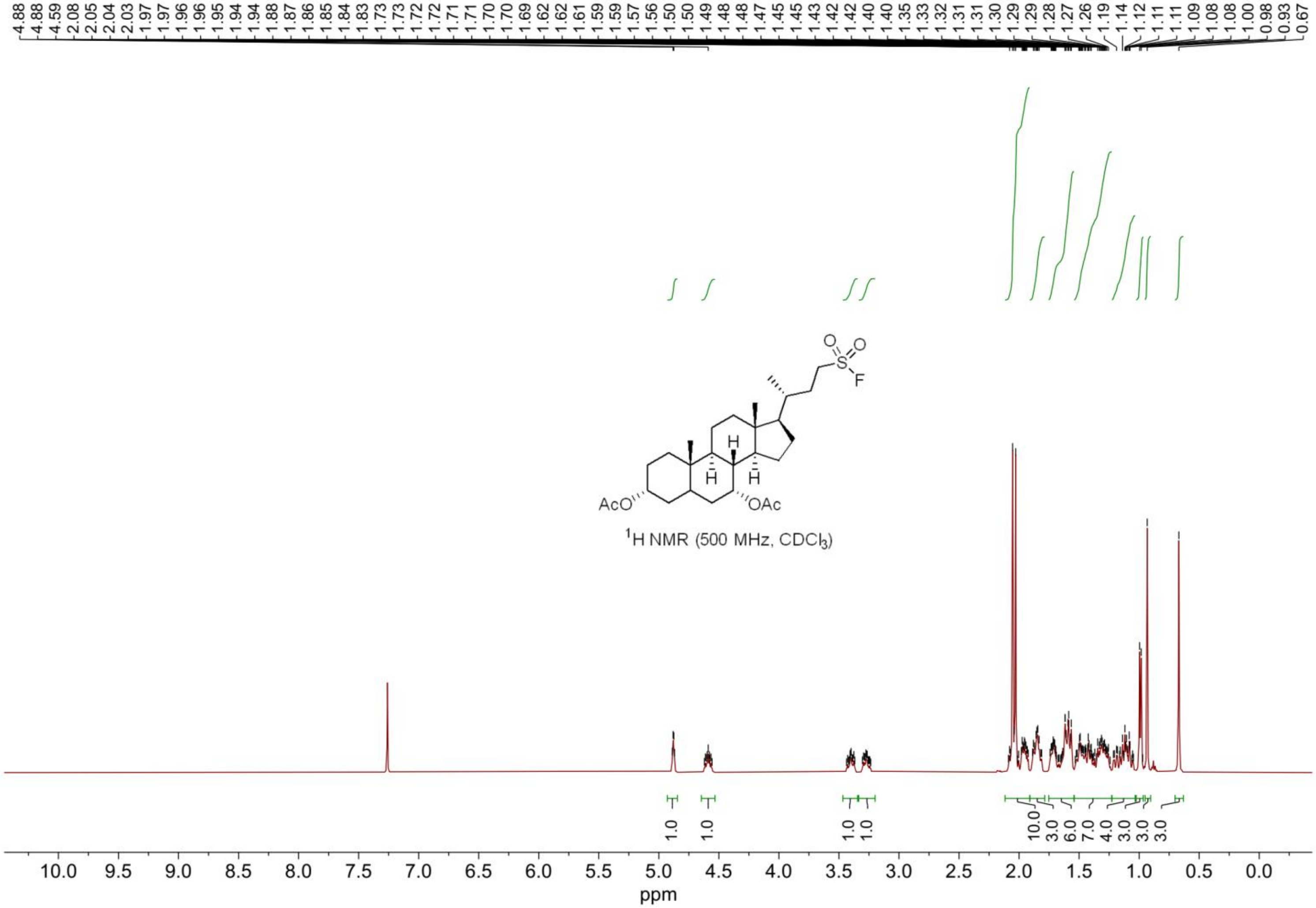

Go back to table of contents

(3*R*,7*R*,8*R*,9*S*,10*S*,13*R*,14*S*,17*R*)-17-((*R*)-4-(Fluorosulfonyl)butan-2-yl)-10,13-dimethylhexadecahydro-1*H*-cyclopenta[*a*]phenanthrene-3,7-diyl diacetate (5l)

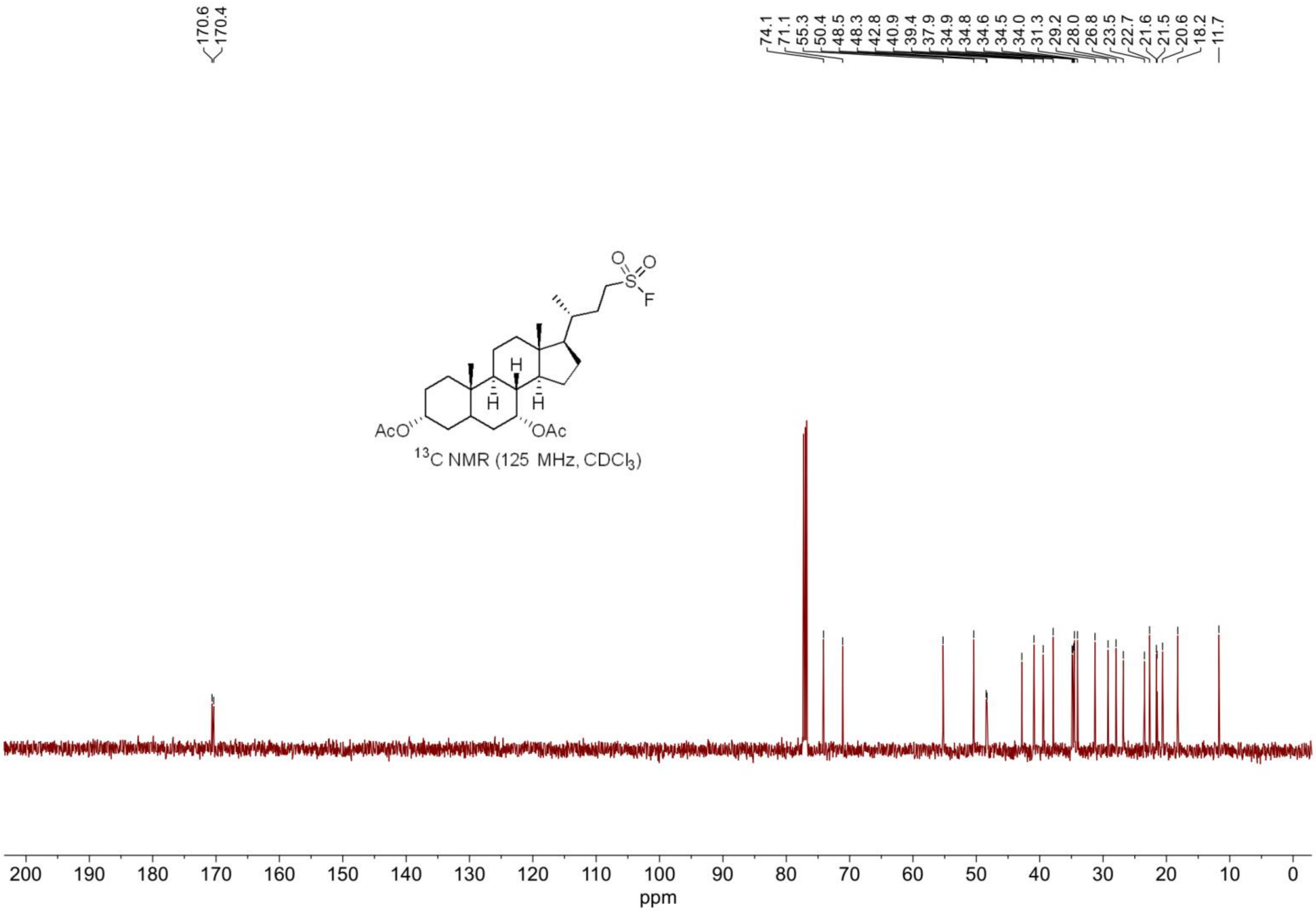

Go back to table of contents

References

1 V. T. Nguyen, V. D. Nguyen, G. C. Haug, H. T. Dang, S. Jin, Z. Li, C. Flores-Hansen, B. S. Benavides, H. D. Arman, O. V. Larionov, *ACS Catal.*, 2019, **9**, 9485–9498.

2 E. Kuruvilla, D. Ramaiah, *J. Phys. Chem. B*, 2007, **111**, 6549–6556.

3 S. Van Mileghem, W. M. De Borggraeve, *Org. Process Res. Dev.*, 2017, **21**, 785–787.

4 G. P. Crooks, S. D. Copley, *Biochemistry*, 1994, **33**, 11645–11649.

5 M. Jung, G. Brosch, D. Kölle, H. Scherf, C. Gerhäuser, P. Loidl, *J. Med. Chem.*, 1999, **42**, 4669–4679.

6 E. Brehm, R. Breinbauer, *Org. Biomol. Chem.*, 2013, **11**, 4750–4756.

7 H. Zhao, X. Guo, H. Tian, C. Li, Z. Xie, Y. Geng, F. Wang, *J. Mater. Chem.*, 2010, **20**, 3092–3097.

8 G. B. V. Subramanian, R. Sharma, *Synth. Commun.*, 1989, **19**, 1197–1202.

9 W.A. Etzel, S. Berger, *J. Label. Compd. Radiopharm.*, 1990, **28**, 977–982.

10 S. Jónsson, F. G. Odille, P. O. Norrby, K. Wärnmark, *Org. Biomol. Chem.*, 2006, **4**, 1927–1948.

11 E. C. Liu, J. J. Topczewski, *J. Am. Chem. Soc.*, 2019, **141**, 5135–5138

12 R. Thakare, H. Gao, R. E. Kosa, Y. A. Bi, M. V. Varma, M. A. Cerny, R. Sharma, M. Kuhn, B. Huang, Y. Liu, A. Yu, *Drug Metab. Dispos.*, 2017, **45**, 721–733.

13 H. T. Dang, G. C. Haug, V. T. Nguyen, N. T. Vuong, V. D. Nguyen, H. D. Arman, O. V. Larionov, *ACS Catalysis*, 2020, **10**, 11448–11457.

14 L. Pitzer, F. Schäfers, and F. Glorius, *Angew. Chem. Int. Ed.,* 2019, **58**, 8572–8576.

15 A. Defoin, R. Defoin-Straatmann, K. Hildenbrand, E. Bittersmann, D. Kreft, H. J. Kuhn, *J. Photochem.*, 1986, **33**, 237–255.

16 Texas Advanced Computing Center (TACC), The University of Texas at Austin

17 J. Towns, T. Cockerill, M. Dahan, I. Foster, K. Gaither, A. Grimshaw, V. Hazlewood, S. Lathrop, D. Lifka, G. D. Peterson, R. Roskies, J. R. Scott, N. Wilkins-Diehr, *Comput. Sci. Eng.*, 2014, **16**, 62–74.

18 Gaussian 16, Revision B.01, M. J. Frisch, G. W. Trucks, H. B. Schlegel, G. E. Scuseria, M. A. Robb, J. R. Cheeseman, G. Scalmani, V. Barone, G. A. Petersson, H. Nakatsuji, X. Li, M. Caricato, A. V. Marenich, J. Bloino, B. G. Janesko, R. Gomperts, B. Mennucci, H. P. Hratchian, J. V. Ortiz, A. F. Izmaylov, J. L. Sonnenberg, D. Williams-Young, F. Ding, F. Lipparini, F. Egidi, J. Goings, B. Peng, A. Petrone, T. Henderson, D. Ranasinghe, V. G. Zakrzewski, J. Gao, N. Rega, G. Zheng, W. Liang, M. Hada, M. Ehara, K. Toyota, R. Fukuda, J. Hasegawa, M. Ishida, T. Nakajima, Y. Honda, O. Kitao, H. Nakai, T. Vreven, K. Throssell, J. A. Montgomery, Jr., J. E. Peralta, F. Ogliaro, M. J. Bearpark, J. J. Heyd, E. N. Brothers, K. N. Kudin, V. N. Staroverov, T. A. Keith, R. Kobayashi, J. Normand, K. Raghavachari, A. P. Rendell, J. C. Burant, S. S. Iyengar, J. Tomasi, M. Cossi, J. M. Millam, M. Klene, C. Adamo, R. Cammi, J. W.

Ochterski, R. L. Martin, K. Morokuma, O. Farkas, J. B. Foresman, D. J. Fox, Gaussian, Inc., Wallingford CT, 2016.

19 P. Pracht, F. Bohle, S. Grimme, *Phys. Chem. Chem. Phys.*, 2020, **22**, 7169–7192.

20 S. Grimme, C. Bannwarth, P. Shushkov, *J Chem. Theory Comput.*, 2017, **13**, 1989–2009.

21 C. Bannwarth, S. Ehlert, S. Grimme, *J. Chem. Theory Comput.*, 2019, **15**, 1652–1671.

22 Chemcraft - graphical software for visualization of quantum chemistry computations. https://www.chemcraftprog.com

23 (*a*) R. Z. Khaliullin, E. A. Cobar, R. C. Lochan, A. T. Bell, M. Head-Gordon, *J. Phys. Chem. A*, 2007, **111**, 8753–8765; (*b*) R. Z. Khaliullin, A. T. Bell, M. Head-Gordon, *J. Chem. Phys. B*, 2008, **128**, 184112; (*c*) P. R. Horn, E. J. Sundstrom, T. A. Baker, M. Head-Gordon, *J. Chem. Phys.*, 2013, **138**, 134119; (*d*) P. R. Horn, Y. Mao, M. Head-Gordon, *Phys. Chem. Chem. Phys.*, 2016, **18**, 23067.

24 R. Z. Khaliullin, A. T. Bell, M. Head-Gordon, *J. Chem. Phys. B*, 2008, **128**, 184112.

25 Y. Shao, Z. Gan, E. Epifanovsky, A. T. B. Gilbert, M. Wormit, J. Kussmann, A. W. Lange, A. Behn, J. Deng, X. Feng, D. Ghosh, M. Goldey, P. R. Horn, L. D. Jacobson, I. Kaliman, R. Z. Khaliullin, T. Kuś, A. Landau, J. Liu, E. I. Proynov, Y. M. Rhee, R. M. Richard, M. A. Rohrdanz, R. P. Steele, E. J. Sundstrom, H. L. Woodcock, P. M. Zimmerman, D. Zuev, B. Albrecht, E. Alguire, B. Austin, G. J. O. Beran, Y. A. Bernard, E. Berquist, K. Brandhorst, K. B. Bravaya, S. T. Brown, D. Casanova, C.-M. Chang, Y. Chen, S. H. Chien, K. D. Closser, D. L. Crittenden, M. Diedenhofen, R. A. DiStasio, H. Do, A. D. Dutoi, R. G. Edgar, S. Fatehi, L. Fusti-Molnar, A. Ghysels, A. Golubeva-Zadorozhnaya, J. Gomes, M. W. D. Hanson-Heine, P. H. P. Harbach, A. W. Hauser, E. G. Hohenstein, Z. C. Holden, T.-C. Jagau, H. Ji, B. Kaduk, K. Khistyaev, J. Kim, J. Kim, R. A. King, P. Klunzinger, D. Kosenkov, T. Kowalczyk, C. M. Krauter, K. U. Lao, A. D. Laurent, K. V. Lawler, S. V. Levchenko, C. Y. Lin, F. Liu, E. Livshits, R. C. Lochan, A. Luenser, P. Manohar, S. F. Manzer, S.-P. Mao, N. Mardirossian, A. V. Marenich, S. A. Maurer, N. J. Mayhall, E. Neuscamman, C. M. Oana, R. Olivares-Amaya, D. P. O’Neill, J. A. Parkhill, T. M. Perrine, R. Peverati, A. Prociuk, D. R. Rehn, E. Rosta, N. J. Russ, S. M. Sharada, S. Sharma, D. W. Small, A. Sodt, T. Stein, D. Stück, Y.-C. Su, A. J. W. Thom, T. Tsuchimochi, V. Vanovschi, L. Vogt, O. Vydrov, T. Wang, M. A. Watson, J. Wenzel, A. White, C. F. Williams, J. Yang, S. Yeganeh, S. R. Yost, Z.-Q. You, I. Y. Zhang, X. Zhang, Y. Zhao, B. R. Brooks, G. K. L. Chan, D. M. Chipman, C. J. Cramer, W. A. Goddard, M. S. Gordon, W. J. Hehre, A. Klamt, H. F. Schaefer, M. W. Schmidt, C. D. Sherrill, D. G. Truhlar, A. Warshel, X. Xu, A. Aspuru-Guzik, R. Baer, A. T. Bell, N. A. Besley, J.-D. Chai, A. Dreuw, B. D. Dunietz, T. R. Furlani, S. R. Gwaltney, C.-P. Hsu, Y. Jung, J. Kong, D. S. Lambrecht, W. Liang, C. Ochsenfeld, V. A. Rassolov, L. V. Slipchenko, J. E. Subotnik, T. Van Voorhis, J. M. Herbert, A. I. Krylov, P. M. W. Gill, M. Head-Gordon, *Mol. Phys.*, 2015, **113**, 184–215.

26 pASDI is available at:

https://github.com/grahamhaug/LarionovLabComputationalScripts/tree/main/pASDI and should be useable on any Linux-based system.

27 Z. Liu, T. Lu, Q. Chen, *Carbon*, **2020**, *165*, 461–467.

28 T. Lu, F. Chen, *J. Comput. Chem.*, **2012**, *33*, 580–592.

29 NBO 7.0. E. D. Glendening, J. K. Badenhoop, A. E. Reed, J. E. Carpenter, J. A. Bohmann, C. M. Morales, P. Karafiloglou, C. R. Landis, F. Weinhold, Theoretical Chemistry Institute, University of Wisconsin, Madison, 2018.

30 CYLview, 1.0b, C. Y. Legault, Université de Sherbrooke, 2009 (http://www.cylview.org).

31 S. Grimme, J. Antony, S. Ehrlich, H. Krieg, *J. Chem. Phs.*, 2010, **132**, 154104.

32 S. Grimme, S. Ehrlich, L. Goerigk, *J. Comput. Chem.*, 2011, **32**, 1456–1465.

33 Y. Zhao, D. G. Truhlar, *J. Phys. Chem. A*, 2005, **109**, 5656–5667.

34 L. Goerigk, A. Hansen, C. Bauer, S. Ehrlich, A. Najibi, S. Grimme, *Phys. Chem. Chem. Phys.*, 2017, **19**, 32184.

35 F. Weigend, R. Ahlrichs, *Phys. Chem. Chem. Phys.*, 2005, **7**, 3297–3305.

36 A. V. Marenich, C. J. Cramer, D. G. Truhlar, *J. Phys. Chem. B*, 2009, **113**, 6378–6396.

37 A. Hansen, C. Bannwarth, S. Grimme, P. Petrović, C. Werlé, J.-P. Djukic, *ChemistryOpen*, 2014, **3**, 177–189.

38 (*a*) G. Luchini, J. V. Alegre-Requena, Y. Guan, I. Funes-Ardoiz, R. S. Paton, GoodVibes: GoodVibes v3.0.1 (2019); (*b*) G. Luchini, J. V. Alegre-Requena, Y. Guan, I. Funes-Ardoiz, R. S. Paton, *F1000Research*, 2020, **9**, 291.

39 B. P. Pritchard, D. Altarawy, B. Didier, T. D. Gibson, T. L. Windus, *J. Chem. Inf. Model*, 2019, **59**, 4814–4820.

40 (*a*) K. Morokuma, *J. Chem. Phys.*, *1971*, **55**, 1236–1244; (*b*) F. M. Bickelhaupt, *J. Comput. Chem.*, 1999, **20**, 114; (*c*) D. H. Ess, K. N. Houk, *J. Am. Chem. Soc.*, 2007, **129**, 10646–10647; (*d*) L. P. Wolters, F. M. Bickelhaupt, *WIREs Comput. Mol. Sci.*, 2015, **5**, 324–343; (*e*) F. M. Bickelhaupt, K. N. Houk, *Angew. Chem., Int. Ed.*, 2017, **56**, 10070–10086.

41 W.-J. van Zeist, A. H. Koers, L. P. Wolters, F. M. Bickelhaupt, *J. Chem. Theory Comput.*, 2008, **4**, 920–928.

42 J. Chai, M. Head-Gordon, *Phys. Chem. Chem. Phys*., 2008, **10**, 6615–6620.

43 L. Goerigk, S. Grimme, *J. Chem. Theory Comput.*, 2011, **7**, 291–309.

44 E. Brémond, M. Savarese, C. Adamo, D. Jacquemin, *J. Chem. Theory Comput.*, 2018, **14**, 3715–3727.

45 (*a*) A. Dreuw, J. L. Weisman, M. Head-Gordon, *J. Chem. Phys.*, 2003, **119**, 2943; (*b*) A. Dreuw, *J. Am. Chem. Soc.*, 2004, **126**, 4007–4016; (*c*) A. Dreuw, M. Head-Gordon, *Chem. Rev.*, 2005, **105**, 4009–4037.

46 (*a*) C. Adamo, D. Jacquemin, *Chem. Soc. Rev.*, 2013, **42**, 845–856; (*b*) F. Furche, D. Rappoport, *Comp. Theor. Chem.*, 2005, **16**, 93; (c) F. Maschietto, M. Campetella, J. S. Garcia, C. Adamo, I. Ciofini, *J. Chem. Phys.*, 2021, **154**, 204102.

47 L. Goerigk, S. Grimme, *Phys. Chem. Chem. Phys.*, 2011, **13**, 6670–6688.

48 Ó. Rubio-Pons, L. Serrano-Andrés, M. Merchán, *J. Phys. Chem. A.*, 2001, **105**, 9664-9673.

49 (*a*) T. Le Bahers, C. Adamo, I. Ciofini, *J. Chem. Theory Comput.*, 2011, **7**, 2498–2506; (*b*) A. Dreuw, M. Head-Gordon, *Chem. Rev.*, 2005, **105**, 4009–4037; (*c*) Z. Liu, T. Lu, Q. Chen, *Carbon*, 2020, **165**, 461–467.

50 G. Luchini, R. S. Paton, DBSTEP. https://github.com/patonlab/DBSTEP https://zenodo.org/badge/latestdoi/198946518

51 N. M. O'Boyle, A. L. Tenderhold, K. M. Langner, *J. Comp. Chem.*, 2008, **29**, 839–845.

52 (*a*) C. B. Santiago, J.-Y. Guo, M. S. Sigman, *Chem. Sci.*, 2018, **9**, 2398–2412; (*b*) T. J. DeLano, S. E. Dibrell, C. R. LAcker, A. R. Pancoast, K. E. Poremba, L. Cleary, M. S. Sigman, S. E. Reisman, *Chem. Sci.*, 2021, **12**, 7758–7762; (*c*) J. Werth, M. S. Sigman, *J. Am. Chem. Soc.*, 2020, **142**, 16382–16391.

53 (*a*) F. Pedregosa, G. Varoquaux, A. Gramfort, V. Michel, B. Thirion, O. Grisel, M. Blondel, P. Prettenhofer, R. Weiss, V. Dubourg, J. Vanderplas, A. Passos, D. Cournapeau, M. Brucher, M. Perrot, E. Duchesnay, *J. Mach. Learn. Res.*, **2011**, *12*, 2825–2830; (*b*) C. R. Harris, K. J. Millman, S. J. van der Walt, R. Gommers, P. Virtanen, D. Cournapeau, E. Wieser, J. Taylor, S. Berg, N. J. Smith, R. Kern, M. Picus, S. Hoyer, M. H. van Kerkwijk, M. Brett, A. Haldane, J. F. del Río, M. Wiebe, P. Peterson, P. Gérard-Marchant, K. Sheppard, T. Reddy, W. Weckesser, H. Abbasi, C. Gohlke, T. E. Oliphant, *Nature*, 2020, **585**, 357–362; (*c*) J. D. Hunter, *Comput. Sci. Eng.*, 2007, **9**, 90–95.
